# Posicionamento sobre o Uso do Ultrassom "Point of Care" (POCUS) na Medicina Cardiovascular – 2026

**DOI:** 10.36660/abc.20260222

**Published:** 2026-05-04

**Authors:** Monica Luiza de Alcantara, Marcos Paulo Lacerda Bernardo, Adenalva Lima de Souza Beck, Simone Nascimento Santos, Samira Saady Morhy, Edgar Bezerra de Lira, Alex dos Santos Felix, Daniel Goldwasser, Maria Estefânia Bosco Otto, Angelo Antunes Salgado, Ana Paula dos Reis Velloso Siciliano, Fernando Melo, Claudia Maria Vilas Freire, Ana Cristina Lopes Albricker, Rafael Modesto Fernandes, Frederico José Neves Mancuso, Dalton de Souza Barros, Hatem Soliman Aboumarie, Marcelo Haertel Miglioranza, Salomon Israel do Amaral, Marcelo Dantas Tavares de Melo, Marcio Mendes Pereira, Antonio Tito Paladin, Lidia Ana Zytynski Moura, Pedro Gabriel Melo de Barros e Silva, Alexandre de Matos Soeiro, Carlos Eduardo Lucena Montenegro, Luiz Cláudio Danzmann, João Ricardo Cordeiro Fernandes, Silvio Henrique Barberato

**Affiliations:** 1 Hospital Quinta D’Or RDSL Rio de Janeiro RJ Brasil Hospital Quinta D’Or RDSL, Rio de Janeiro, RJ – Brasil; 2 Universidade Estadual do Rio de Janeiro Hospital Universitário Pedro Ernesto Rio de Janeiro RJ Brasil Hospital Universitário Pedro Ernesto (HUPE) da Universidade Estadual do Rio de Janeiro, Rio de Janeiro, RJ – Brasil; 3 Hospital Copa Star Rio de Janeiro RJ Brasil Hospital Copa Star, Rio de Janeiro, RJ – Brasil; 4 Instituto de Cardiologia e Transplantes do Distrito Federal Brasília DF Brasil Instituto de Cardiologia e Transplantes do Distrito Federal, Brasília, DF – Brasil; 5 Hospital Sirio Libanês Brasília DF Brasil Hospital Sirio Libanês, Brasília, DF – Brasil; 6 Eccos Diagnóstico CardioVascular Avançado Brasília DF Brasil Eccos Diagnóstico CardioVascular Avançado, Brasília, DF – Brasil; 7 Einstein Hospital Istaelita São Paulo SP Brasil Einstein Hospital Istaelita, São Paulo, SP – Brasil; 8 Instituto Nacional de Cardiologia Rio de Janeiro RJ Brasil Instituto Nacional de Cardiologia, Rio de Janeiro, RJ – Brasil; 9 Complexo Hospitalar Americas Rio de Janeiro RJ Brasil Complexo Hospitalar Americas, Rio de Janeiro, RJ – Brasil; 10 Hospital Copa D’Or Rio de Janeiro RJ Brasil Hospital Copa D’Or, Rio de Janeiro, RJ – Brasil; 11 Universidade de Brasília Brasília DF Brasil Universidade de Brasília, Brasília, DF – Brasil; 12 Rede D’Or Rio de Janeiro RJ Brasil Rede D’Or, Rio de Janeiro, RJ – Brasil; 13 Hospital DF Star - Rede D’Or Brasília DF Brasil Hospital DF Star - Rede D’Or, Brasília, DF – Brasil; 14 Empresa Brasileira de Serviços Hospitalares Belo Horizonte MG Brasil Empresa Brasileira de Serviços Hospitalares (EBSERH), Belo Horizonte, MG – Brasil; 15 Hospital das Clínicas da Universidade Federal de Minas Gerais Belo Horizonte MG Brasil Hospital das Clínicas da Universidade Federal de Minas Gerais (HC-UFMC), Belo Horizonte, MG – Brasil; 16 Centro Universitário UniBh-Inspirali Belo Horizonte MG Brasil Centro Universitário UniBh-Inspirali, Belo Horizonte, MG – Brasil; 17 Hospital Aliança Rede D’Or Salvador BA Brasil Hospital Aliança Rede D’Or, Salvador, BA – Brasil; 18 Universidade Federal de São Paulo Escola Paulista de Medicina São Paulo SP Brasil Universidade Federal de São Paulo - Escola Paulista de Medicina (UNIFESP/EPM), São Paulo, SP – Brasil; 19 Hospital Cardio Pulmonar Salvador BA Brasil Hospital Cardio Pulmonar, Salvador, BA – Brasil; 20 Royal Brompton and Harefield Hospitals Harefield Hospital Londres UK Harefield Hospital, Royal Brompton and Harefield Hospitals, Londres, UK – Inglaterra; 21 Hospital Mae de Deus EcoHaertel Porto Alegre RS Brasil EcoHaertel - Hospital Mae de Deus, Porto Alegre, RS – Brasil; 22 Hospital Samaritano Botafogo Rio de Janeiro RJ Brasil Hospital Samaritano Botafogo, Rio de Janeiro, RJ – Brasil; 23 Universidade Federal da Paraíba João Pessoa PB Brasil Universidade Federal da Paraíba (UFPB), João Pessoa, PB – Brasil; 24 UDI Hospital Rede D'or São Luiz São Luís MA Brasil UDI Hospital Rede D'or São Luiz, São Luís, MA – Brasil; 25 Instituto Dante Pazzanese de Cardiologia São Paulo SP Brasil Instituto Dante Pazzanese de Cardiologia, São Paulo, SP – Brasil; 26 Pontifícia Universidade Católica do Paraná Curitiba PR Brasil Pontifícia Universidade Católica do Paraná (PUC-PR), Curitiba, PR – Brasil; 27 Hospital do Coração São Paulo SP Brasil Hospital do Coração, São Paulo, SP – Brasil; 28 Brazilian Clinical Research Institute São Paulo SP Brasil Brazilian Clinical Research Institute, São Paulo, SP – Brasil; 29 Centro Universitário São Camilo São Paulo SP Brasil Centro Universitário São Camilo, São Paulo, SP – Brasil; 30 Instituto do Coração do Hospital das Clínicas da Faculdade de Medicina da Universidade de São Paulo São Paulo SP Brasil Instituto do Coração do Hospital das Clínicas da Faculdade de Medicina da Universidade de São Paulo (InCor-HCFMUSP), São Paulo, SP – Brasil; 31 Hospital do Coração de Pernambuco Recife PE Brasil Hospital do Coração de Pernambuco (PROCAPE), Recife, PE – Brasil; 32 Universidade de Pernambuco Recife PE Brasil Universidade de Pernambuco, Recife, PE – Brasil; 33 Brasil Hospital Esperança Recife Recife PE Brasil Brasil Hospital Esperança Recife - Rede D'or, Recife, PE – Brasil; 34 Universidade Luterana do Brasil Porto Alegre RS Brasil Universidade Luterana do Brasil, Porto Alegre, RS – Brasil; 35 Santa Casa de Porto Alegre Porto Alegre RS Brasil Santa Casa de Porto Alegre, Porto Alegre, RS – Brasil; 36 Centro de Diagnóstico Cardiovascular Cardioeco Curitiba PR Brasil Cardioeco - Centro de Diagnóstico Cardiovascular, Curitiba, PR – Brasil

**Table:** 

Posicionamento sobre o Uso do Ultrassom "Point of Care" (POCUS) na Medicina Cardiovascular – 2026	
O relatório abaixo lista as declarações de interesse conforme relatadas à SBC pelos especialistas durante o período de desenvolvimento deste posicionamento, 2025/2026.	
Especialista	Tipo de relacionamento com a indústria
Adenalva Lima de Souza Beck	Nada a ser declarado
Alex dos Santos Felix	Nada a ser declarado
Alexandre de Matos Soeiro	Declaração financeira B - Financiamento de pesquisas sob sua responsabilidade direta/pessoal (direcionado ao departamento ou instituição) provenientes da indústria farmacêutica, de órteses, próteses, equipamentos e implantes, brasileiras ou estrangeiras: - Biomerieux.
Ana Cristina Lopes Albriker	Nada a ser declarado
Ana Paula dos Reis Veloso Siciliano	Nada a ser declarado
Angelo Salgado	Nada a ser declarado
Antonio Tito Paladino Filho	Nada a ser declarado
Carlos Eduardo Lucena Montenegro	Declaração financeira A - Pagamento de qualquer espécie e desde que economicamente apreciáveis, feitos a (i) você, (ii) ao seu cônjuge/ companheiro ou a qualquer outro membro que resida com você, (iii) a qualquer pessoa jurídica em que qualquer destes seja controlador, sócio, acionista ou participante, de forma direta ou indireta, recebimento por palestras, aulas, atuação como proctor de treinamentos, remunerações, honorários pagos por participações em conselhos consultivos, de investigadores, ou outros comitês, etc. Provenientes da indústria farmacêutica, de órteses, próteses, equipamentos e implantes, brasileiras ou estrangeiras: - Bayer: Firialta (IC); Pfizer: Vyndaquel/ Vynkella (Amiloidose); Alnylan: Amvuttra (Amiloidose) Servier: Vastarel (DAC); Novartis: Entresto, Sybrava (IC, DLP); AstraZeneca: Forxiga, Lokelma, Breztri (IC, DRC, DPOC); Viatris: Inspra (IC); Merck: Concor (IC, DAC); Novo Nordisk: Wegovy (Obesidade, IC); EMS: Vynaxa (DAC). Outros relacionamentos Financiamento de atividades de educação médica continuada, incluindo viagens, hospedagens e inscrições para congressos e cursos, provenientes da indústria farmacêutica, de órteses, próteses, equipamentos e implantes, brasileiras ou estrangeiras: - Novo Nordisk: Wegovy (Obesidade, IC).
Claudia Maria Vilas Freire	Nada a ser declarado
Dalton de Souza Barros	Nada a ser declarado
Daniel Goldwasser	Nada a ser declarado
Edgar Bezerra de Lira Filho	Nada a ser declarado
Fernando Mello Netto	Declaração financeira A - Pagamento de qualquer espécie e desde que economicamente apreciáveis, feitos a (i) você, (ii) ao seu cônjuge/ companheiro ou a qualquer outro membro que resida com você, (iii) a qualquer pessoa jurídica em que qualquer destes seja controlador, sócio, acionista ou participante, de forma direta ou indireta, recebimento por palestras, aulas, atuação como proctor de treinamentos, remunerações, honorários pagos por participações em conselhos consultivos, de investigadores, ou outros comitês, etc. Provenientes da indústria farmacêutica, de órteses, próteses, equipamentos e implantes, brasileiras ou estrangeiras: - St. Jude – Abbott: MitraClip (TEER); Edwards Lifesciences: PASCAL (TEER); Abbott: Amplatzer (oclusor de FOP, oclusor de CIA); Amulet (oclusão do apêndice atrial esquerdo – OAAE).
Frederico José Neves Mancuso	Nada a ser declarado
Hatem Soliman Aboumarie	Nada a ser declarado
João Ricardo Cordeiro Fernandes	Nada a ser declarado
Lidia Ana Zytynski Moura	Declaração financeira A - Pagamento de qualquer espécie e desde que economicamente apreciáveis, feitos a (i) você, (ii) ao seu cônjuge/ companheiro ou a qualquer outro membro que resida com você, (iii) a qualquer pessoa jurídica em que qualquer destes seja controlador, sócio, acionista ou participante, de forma direta ou indireta, recebimento por palestras, aulas, atuação como proctor de treinamentos, remunerações, honorários pagos por participações em conselhos consultivos, de investigadores, ou outros comitês, etc. Provenientes da indústria farmacêutica, de órteses, próteses, equipamentos e implantes, brasileiras ou estrangeiras: - Bayer, Merck, Novartis, Novo Nordisk, Lilly, Viatris. Outros relacionamentos Participação societária de qualquer natureza e qualquer valor economicamente apreciável de empresas na área de saúde, de ensino ou em empresas concorrentes ou fornecedoras da SBC: - Novo Nordisk, Astra.
Luiz Claudio Danzmann	Declaração financeira A - Pagamento de qualquer espécie e desde que economicamente apreciáveis, feitos a (i) você, (ii) ao seu cônjuge/ companheiro ou a qualquer outro membro que resida com você, (iii) a qualquer pessoa jurídica em que qualquer destes seja controlador, sócio, acionista ou participante, de forma direta ou indireta, recebimento por palestras, aulas, atuação como proctor de treinamentos, remunerações, honorários pagos por participações em conselhos consultivos, de investigadores, ou outros comitês, etc. Provenientes da indústria farmacêutica, de órteses, próteses, equipamentos e implantes, brasileiras ou estrangeiras: - AstraZeneca: Lokelma; Bristol Myers Squibb: Camzyos; Bayer: Firialta. Outros relacionamentos Financiamento de atividades de educação médica continuada, incluindo viagens, hospedagens e inscrições para congressos e cursos, provenientes da indústria farmacêutica, de órteses, próteses, equipamentos e implantes, brasileiras ou estrangeiras: - Astra Zeneca: Lokelma. Possui qualquer outro interesse (financeiro ou a qualquer outro título) que deva ser declarado tendo em vista o cargo ocupado na SBC, ainda que não expressamente elencado anteriormente: - Area de saúde: ensino médico.
Marcelo Dantas Tavares de Melo	Nada a ser declarado
Marcelo Haertel Miglioranza	Nada a ser declarado
Marcio Mendes Pereira	Declaração financeira A - Pagamento de qualquer espécie e desde que economicamente apreciáveis, feitos a (i) você, (ii) ao seu cônjuge/ companheiro ou a qualquer outro membro que resida com você, (iii) a qualquer pessoa jurídica em que qualquer destes seja controlador, sócio, acionista ou participante, de forma direta ou indireta, recebimento por palestras, aulas, atuação como proctor de treinamentos, remunerações, honorários pagos por participações em conselhos consultivos, de investigadores, ou outros comitês, etc. Provenientes da indústria farmacêutica, de órteses, próteses, equipamentos e implantes, brasileiras ou estrangeiras: - Abbott: MitraClip. Outros relacionamentos Financiamento de atividades de educação médica continuada, incluindo viagens, hospedagens e inscrições para congressos e cursos, provenientes da indústria farmacêutica, de órteses, próteses, equipamentos e implantes, brasileiras ou estrangeiras: - Abbott: MitraClip
Marcos Paulo Lacerda Bernardo	Nada a ser declarado
Maria Estefânia Bosco Otto	Nada a ser declarado
Monica Luiza de Alcantara	Declaração financeira A - Pagamento de qualquer espécie e desde que economicamente apreciáveis, feitos a (i) você, (ii) ao seu cônjuge/ companheiro ou a qualquer outro membro que resida com você, (iii) a qualquer pessoa jurídica em que qualquer destes seja controlador, sócio, acionista ou participante, de forma direta ou indireta, recebimento por palestras, aulas, atuação como proctor de treinamentos, remunerações, honorários pagos por participações em conselhos consultivos, de investigadores, ou outros comitês, etc. Provenientes da indústria farmacêutica, de órteses, próteses, equipamentos e implantes, brasileiras ou estrangeiras: - Boston Medical: proctoria Watchman; Edwards Lifesciences: proctoria PASCAL; Vitória Hospitalar: proctoria MitraClip (Abbott), Amplatzer (Abbott)
Pedro Gabriel Melo de Barros e Silva	Declaração financeira B - Financiamento de pesquisas sob sua responsabilidade direta/pessoal (direcionado ao departamento ou instituição) provenientes da indústria farmacêutica, de órteses, próteses, equipamentos e implantes, brasileiras ou estrangeiras: - Bayer, Novartis, Idorsia, Roche Diagnostics.
Rafael Modesto Fernandes	Outros relacionamentos Financiamento de atividades de educação médica continuada, incluindo viagens, hospedagens e inscrições para congressos e cursos, provenientes da indústria farmacêutica, de órteses, próteses, equipamentos e implantes, brasileiras ou estrangeiras: - Edwards
Salomon Israel do Amaral	Nada a ser declarado
Samira Saady Morhy	Nada a ser declarado
Silvio Henrique Barberato	Declaração financeira A - Pagamento de qualquer espécie e desde que economicamente apreciáveis, feitos a (i) você, (ii) ao seu cônjuge/ companheiro ou a qualquer outro membro que resida com você, (iii) a qualquer pessoa jurídica em que qualquer destes seja controlador, sócio, acionista ou participante, de forma direta ou indireta, recebimento por palestras, aulas, atuação como proctor de treinamentos, remunerações, honorários pagos por participações em conselhos consultivos, de investigadores, ou outros comitês, etc. Provenientes da indústria farmacêutica, de órteses, próteses, equipamentos e implantes, brasileiras ou estrangeiras: - Pfizer: Amiloidose; AstraZeneca: ICFEP. Outros relacionamentos Financiamento de atividades de educação médica continuada, incluindo viagens, hospedagens e inscrições para congressos e cursos, provenientes da indústria farmacêutica, de órteses, próteses, equipamentos e implantes, brasileiras ou estrangeiras: - Pfizer: Amiloidose.
Simone Nascimento Santos	Nada a ser declarado

## Sumário

**1. POCUS: Metodologia, Conceito, Treinamento e Certificação** 9
  **1.1. Metodologia** 9
  **1.2. Conceito e Área de Abrangência** 9
  **1.3. Equipamentos** 10
  **1.4. Treinamento, Certificação, Manutenção da Competência** 10
  **1.5. Aspectos Ético-Legais do Uso do POCUS Cardiovascular no Brasil** 10
**2. POCUS Cardíaco** 13
  **2.1. Principais Janelas no POCUS Cardíaco** 13
  **2.2. Análise da Função Sistólica Global do Ventrículo Esquerdo** 15
    **2.2.1. Aspectos Gerais** 15
    **2.2.2. Formas de Avaliação da Função Sistólica do Ventrículo Esquerdo** 15
    **2.2.3. Outras Técnicas de Avaliação da Função Sistólica do Ventrículo Esquerdo** 15
  **2.3. Pesquisa de Sobrecarga e Função Sistólica do Ventrículo Direito** 15
    **2.3.1. Embolia Pulmonar** 16
    **2.3.2. POCUS para Avaliação de Infarto do Ventrículo Direito** 17
    **2.3.3. Outras Aplicações** 17
    **2.3.4. Limitações** 17
  **2.4. POCUS no Tamponamento Cardíaco** 17
  **2.5. POCUS Avançado** 17
    **2.5.1. Avaliação da Integral Velocidade-Tempo da Via de Saída do Ventrículo Esquerdo, Determinação do Volume Sistólico e Débito Cardíaco** 17
    **2.5.2. Relação E/e’ como Medida da Pressão Atrial Esquerda e Fluido-Tolerância** 19
  **2.6. POCUS Cardíaco Pediátrico** 20
**3. Ultrassonografia Pulmonar** 21
  **3.1. Introdução** 21
  **3.2. Aspectos Técnicos** 21
    **3.2.1. Equipamentos e Transdutores** 21
  **3.3. Protocolo de Estudo** 21
  **3.4. Quantificação de Linhas B** 22
  **3.5. Principais Aplicações da Ultrassonografia Pulmonar** 22
    **3.5.1. Congestão Pulmonar** 22
    **3.5.2. Derrame Pleural** 22
    **3.5.3. Síndromes Intersticiais** 22
    **3.5.4. Consolidação Pneumônica** 24
    **3.5.5. Atelectasia** 24
    **3.5.6. Pneumotórax** 25
    **3.5.7. Tromboembolismo Pulmonar** 25
  **3.6. Conclusão e Perspectivas Futuras** 25
**4. Ultrassom Focado na Avaliação da Congestão Venosa Sistêmica - VExUS** 25
  **4.1. Introdução** 25
  **4.2. Estudos Clínicos e Evidências Científicas** 25
  **4.3. Componentes do VExUS** 26
  **4.4. Graduação do VExUS** 29
  **4.5. Aplicações Clínicas** 29
  **4.6. Limitações e Armadilhas** 29
  **4.7. Conclusão** 30
**5. POCUS na Trombose Venosa Profunda** 30
  **5.1. Introdução** 30
  **5.2. Evidências Científicas do POCUS na Pesquisa de Trombose Venosa Profunda** 31
  **5.3. Técnica do Protocolo de Ultrassonografia Focado para Pesquisa de Trombose Venosa Profunda** 31
**6. POCUS no Paciente em Choque Circulatório** 35
  **6.1. Introdução** 35
  **6.2. Protocolos Diagnósticos** 35
  **6.3. Perguntas Chave para Identificar o Tipo de Choque** 35
  **6.4. Fluido-Responsividade e Fluido-Tolerância** 37
    **6.4.1. Estimativa do Débito Cardíaco** 37
    **6.4.2. Veia Cava Inferior/Superior** 37
    **6.4.3. Manobra de Elevação Passiva dos Membros – Passive Leg Raising** 37
    **6.4.4. Manobra da Pausa Expiratória e Inspiratória** 38
**7. POCUS no Suporte Circulatório Mecânico de Curto Prazo** 40
  **7.1. Introdução** 40
  **7.2. O Papel do POCUS no Suporte Circulatório Mecânico de Curto Prazo** 40
  **7.3. Avaliação Inicial do Paciente** 40
  **7.4. Colocação e Confirmação da Posição do Dispositivo** 40
  **7.5. Balão Intra-Aórtico** 40
    **7.5.1. Introdução** 40
    **7.5.2. Sequência de Exames de Ecocardiografia Transesofágica para Orientar a Inserção do Balão Intra-Aórtico** 40
    **7.5.3. Lista de Verificação Pós-Implantação** 41
  **7.6. Oxigenação por Membrana Extracorpórea Venoarterial (ECMO-VA)** 41
    **7.6.1. Introdução** 41
    **7.6.2. Seleção de Pacientes para ECMO** 41
    **7.6.3. Orientação para Canulação** 41
    **7.6.4. Efeitos Imediatos da ECMO-VA Periférica Observados pela Ecocardiografia à Beira do Leito** 43
    **7.6.5. Avaliação do Alívio e Desmame** 44
    **7.6.6. Monitoramento Durante Testes de Desmame** 45
  **7.7. Desafios e Direções Futuras** 45
  **7.8. Conclusão** 45
**8. POCUS na Unidade de Emergência** 46
  **8.1. POCUS na Dor Torácica** 46
    **8.1.1. Avaliação de Pericardiopatias na Unidade de Emergência** 46
    **8.1.2. Avaliação da Pleura e Pulmão na Unidade de Emergência** 46
  **8.2. Avaliação do Paciente com Dispneia** 46
    **8.2.1. Congestão Pulmonar, Pleuropatias e Outros Diagnósticos Avaliados pela Ultrassonografia Pulmonar** 46
    **8.2.2. Tromboembolismo Pulmonar** 47
  **8.3. Avaliação do Paciente com Instabilidade Hemodinâmica e/ou Disfunção Orgânica** 47
  **8.4. Avaliação do Paciente com Edema Periférico** 48
**9. POCUS na Parada Cardiorrespiratória** 49
  **9.1. Introdução** 49
  **9.2. Aspectos Técnicos** 49
  **9.3. Execução do POCUS-PCR** 50
  **9.4. Confirmação da Parada Cardiorrespiratória, Avaliação da Eficácia das Compressões Torácicas e da Função Prognóstica da Presença da Atividade Cardíaca na Atividade Elétrica sem Pulso** 50
  **9.5. Diagnósticos de Causas Reversíveis pelo POCUS-PCR** 50
    **9.5.1. Tamponamento Cardíaco** 50
    **9.5.2. Tromboembolismo Pulmonar** 50
    **9.5.3. Pneumotórax Hipertensivo** 50
    **9.5.4. Hipovolemia** 50
**10. Procedimentos Guiados pela Ultrassonografia** 50
  **10.1. Acesso Vascular Guiado pelo Ultrassom** 50
    **10.1.1. Técnica de Punção** 52
    **10.1.2. Passo a Passo da Punção (Utilizar Material Estéril)** 52
    **10.1.3. Punção em Corte Transversal (Eixo Curto)** 52
    **10.1.4. Punção em Corte Longitudinal (Eixo Longo)** 53
    **10.1.5. Locais de Punção Guiada por Ultrassonografia** 53
    **10.1.6. Outras Punções Vasculares Guiadas por Ultrassonografia** 54
  **10.2. Pericardiocentese Guiada por Ultrassom** 54
    **10.2.1. Técnica de Punção Guiada por Ultrassonografia** 54
    **10.2.2. Contraindicações** 55
    **10.2.3. Complicações** 55
  **10.3. Avaliação do Estômago Cheio** 55
    **10.3.1. Técnica de Avaliação** 55
    **10.3.2. Achados Sonográficos** 55
**11. Considerações Finais sobre este Posicionamento** 56
  **11.1. Realidade Atual e Perspectivas Futuras** 56
  **11.2. Contextualização do POCUS Cardiovascular no Âmbito da Saúde Pública no Brasil** 56
**Referências** 57
**Suplemento 1** 63
**Suplemento 2** 64

## 1. POCUS: Metodologia, Conceito, Treinamento e Certificação

### 1.1. Metodologia

Este posicionamento foi elaborado a partir de reuniões *online* com um grupo multidisciplinar de especialistas. Cada participante redigiu sua seção com base em sua experiência clínica e na revisão da literatura relevante. As discussões em grupo permitiram a consolidação das recomendações apresentadas neste documento.

### 1.2. Conceito e Área de Abrangência

O termo POCUS, do inglês *point of care ultrasound* (ultrassonografia no ponto de atendimento), se refere ao uso da ultrassonografia como método auxiliar ao exame clínico, focado em responder uma questão clínica urgente. Neste documento, o termo "POCUS" é aplicado à avaliação anatômica e funcional do coração, pulmão e vasos, à beira do leito, para auxiliar o diagnóstico e a tomada de decisões terapêuticas, em pacientes internados ou em ambiente ambulatorial (desde neonatos até adultos).

O POCUS é importante devido à sua portabilidade, baixo custo e acessibilidade. Pode ser usado por médicos da linha de frente, cardiologistas e não cardiologistas, em salas de pronto atendimento, unidades de terapia intensiva, centros cirúrgicos e ambientes de difícil acesso, onde exames de imagem nem sempre estão prontamente disponíveis. Por outro lado, a adoção do POCUS exige o cumprimento de diversos pré-requisitos (Figura 1).

**Figura 1:**
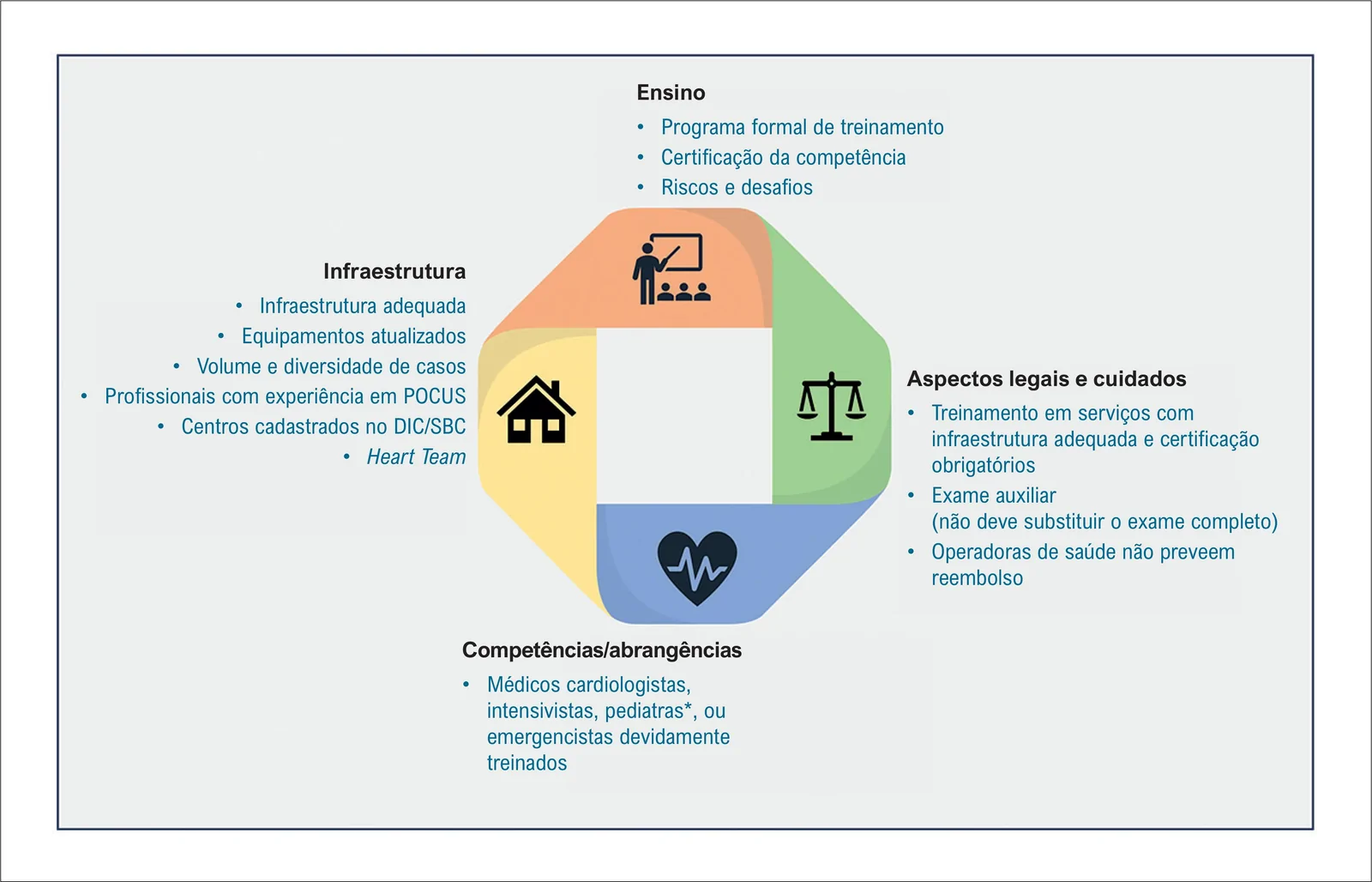
Pré-requisitos para o uso do POCUS em um serviço. *Alterações cardíacas nessa faixa etária devem alertar para a possibilidade de cardiopatia congênita, e um ecocardiografista pediátrico deve realizar um exame completo assim que possível. DIC: Departamento de Imagem Cardiovascular; POCUS: point-of-care ultrasound; SBC: Sociedade Brasileira de Cardiologia.

**Figure:**
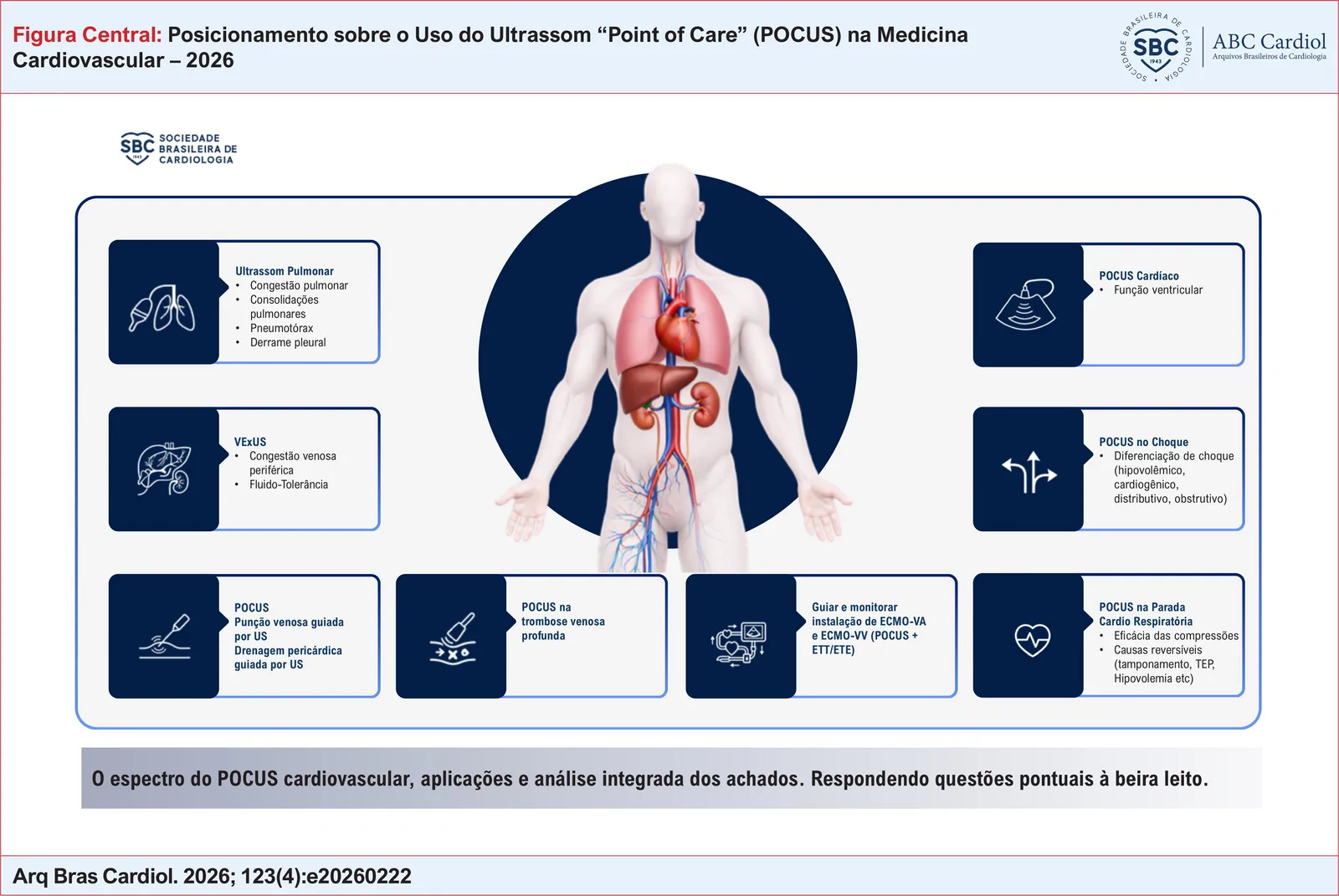
ECMO-VA: Oxigenação por membrana extracorpórea venoarterial; ECMO-VV: Oxigenação por membrana extracorpórea venovenosa; ETE: Ecocardiografia transesofágica; ETT: Ecocardiografia transtorácica; POCUS: Point-of-care ultrasound; US: Ultrassonografia; TEP: Tromboembolismo pulmonar; VExUS: Venous excess ultrasound.

### 1.3. Equipamentos

Apesar de poder ser realizado em diversos equipamentos, o ideal é que o POCUS permita a aquisição de imagens nas modalidades uni e bidimensional, Doppler espectral e mapeamento de fluxo em cores. Também deve contar com transdutores de frequências variáveis (para o coração de adultos e crianças, pulmão e vasos), ajuste de imagem padronizado ou modificável de acordo com a estrutura a ser examinada (*preset*) e por fim, a capacidade de armazenar imagens em formato compatível com os padrões DICOM (*Digital Imaging and Communications in Medicine*). A miniaturização dos equipamentos, que hoje possuem telas do mesmo tamanho das de um *smartphone*, e a inteligência artificial (IA) (para guiar o posicionamento do transdutor) desempenham um importante papel no uso crescente do POCUS.

### 1.4. Treinamento, Certificação, Manutenção da Competência

A metodologia de ensino do POCUS é bastante variada, mas seus principais objetivos, de acordo com o nível desejado, estão resumidos na Tabela 1,^1–8^ e um acrônimo é proposto na Figura 2.^1,2^ O tempo de treinamento e o número de exames necessários podem variar de acordo com a experiência pregressa, o nível de capacitação desejado e a avaliação do supervisor. A Figura 3 apresenta uma sugestão de protocolo que, idealmente, deve ser testado, validado e endossado por sociedades de especialidades afins. As etapas de treinamento propostas pelo grupo estão descritas na Figura 2 e incluem, ao final do processo, uma avaliação e certificação interna da competência (Suplemento 1). Por fim, para minimizar os riscos e superar os desafios envolvidos na realização do POCUS (Figura 4), é necessário atentar para os aspectos legais e para os cuidados apresentados na sequência (Figura 1).

**Tabela 1:** Principais objetivos do treinamento em POCUS para aquisição, interpretação e documentação dos resultados de acordo com o nível desejado (1, 2 e 3)

Habilidades	Nível 1	Nível 2	Nível 3
**Aquisição**			
**1.** Saber identificar as principais estruturas cardíacas ao ultrassom (pericárdio, câmaras cardíacas, valvas, aorta e veia cava inferior).	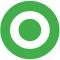	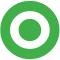	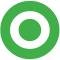
**2.** Aprender conceitos básicos de ultrassom, princípios físicos, geração de imagens e ajuste dos equipamentos (ganho, foco e profundidade) com o objetivo de melhorar a resolução da imagem ao mudar de janela.	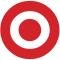 **(1)**	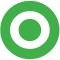	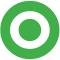
**3.** Adquirir as janelas do POCUS cardíaco (paraesternal longitudinal eeixo curto, apical quatro câmaras, subsostal e veia cava inferior longitudinal e transversa), pulmão (tórax anterior e posterior), aorta transversa abdominal em toda sua extensão, veias femoral e poplítea, e veias viscerais (hepática, porta e renais).	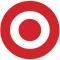 **(2)**	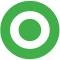	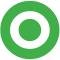
**Interpretação**			
**4.** Supervisão assistida de um profissional treinado e revisão.	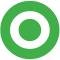	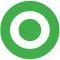	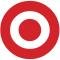
**5.** Reconhecer a presença ou ausência de patologias de acordo com a suspeita clínica visando responder a questões básicas: tamanho das câmaras, espessamento das paredes, disfunção de VE, disfunção de VD, derrame pericárdico, hipovolemia, congestão pulmonar (linhas B), congestão venosa sistêmica, trombose venosa profunda, pneumotórax ou derrame pleural.	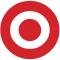 **(3)**	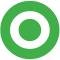 **(5)**	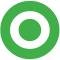 **(6)**
**6.** Conhecer os protocolos de aplicação do POCUS cardiovascular no diagnóstico diferencial do choque, insuficiência respiratória e PCR (SHOCK, RUSH, EGLS).	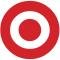	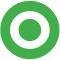	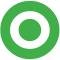
**7.** Avaliar responsividade a fluidos e congestão sistêmica.	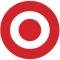	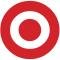	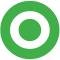
**8.** Saber realizar avaliação básica de valvas, débito cardíaco e pressão de enchimento.	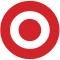	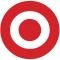	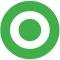
**9.** Em neonatos, identificar a patência do forame oval, persistência do canal arterial e estimar pressão sistólica pulmonar.	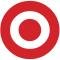	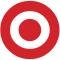	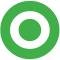
**10.** Utilizar o POCUS na punção ecoguiada, drenagem pericárdica, entubação orotraqueal, avaliação do volume vesical e resíduo gástrico. Reconhecer as limitações do POCUS, riscos envolvidos e cuidados.	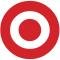	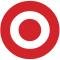	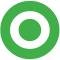
**Resultados**			
**11.** Saber arquivar as imagens no equipamento e relatar os achados do ultrassom em prontuário, assim como integrar os achados do ultrassom com a clínica do paciente.	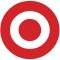 **(4)**	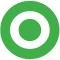	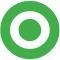
**12.** Desenvolver a habilidade de interpretar criticamente os achados do ultrassom no contexto das situações clínicas de emergência.	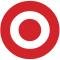	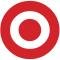	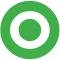
**13.** Registrar a tomada de decisão no cuidado direto ao paciente baseado na análise integrada.	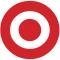	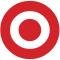	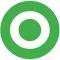
**14.** Arquivar as imagens no equipamento e enviar em DICOM para o sistema PACS do hospital.	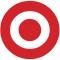	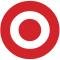	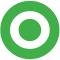
**15.** Saber reconhecer se a patologia sugerida pelo POCUS é compatível com o quadro clínico e indicar, sem demora, exame completo, se dúvida. Da mesma forma, se a suspeita clínica permanece a despeito da ausência de achados ao POCUS.	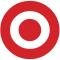	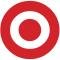	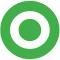

(1) Aprender apenas o posicionamento adequado do paciente, escolha do transdutor, escolha do presente e orientação correta do transdutor.

(2) Apenas janela subcostal e ao menos mais uma janela cardíaca, veia cava inferior (longitudinal), janelas pleurais anteriores, aorta transversa até região proximal da abdominal, veias femoral e poplítea e FAST.

(3) Apenas reconhecer a presença ou ausência de sinais ultrassonográficos de anormalidades cardíacas (avaliação visual do tamanho das câmaras, linhas B, derrame pleural e pericárdico).

(4) Apenas relatar os achados do ultrassom em conjunto com o exame físico.

(5) Exceto avaliação de congestão venosa visceral, todas com avaliação qualitativa do tipo SIM ou NÃO.

(6) Com avaliação semiquantitativa em discreta, moderada ou acentuada, quando aplicável.

DICOM: Digital Imaging and Communications in Medicine; FAST: Focused Assessment with Sonography for Trauma; PACS: Picture Archiving and Communication System; POCUS: point-of-care ultrasound.

**Figura 2:**
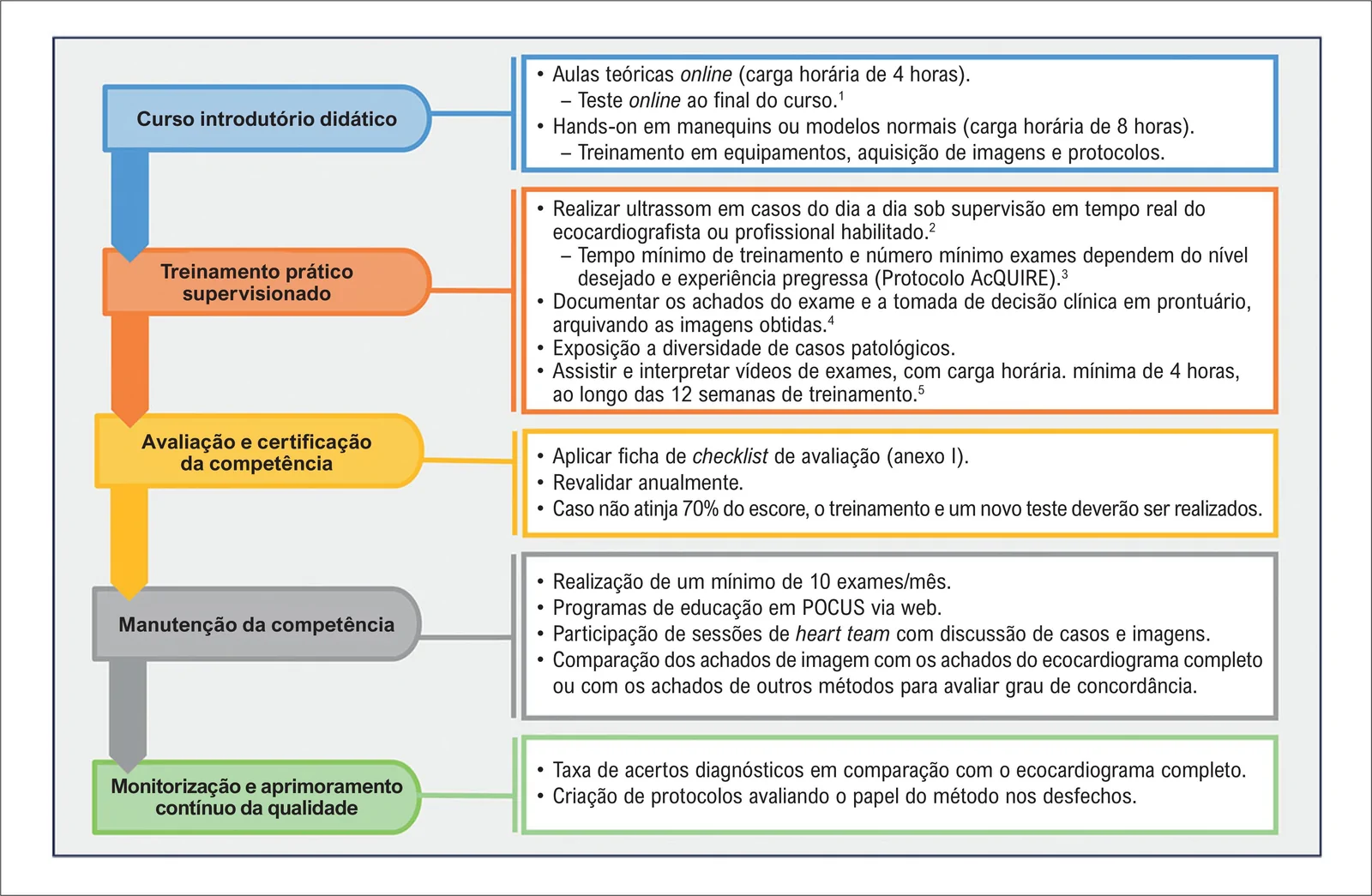
Etapas do treinamento em POCUS. O teste online não tem caráter eliminatório, mas deve ser pré-requisito obrigatório para a participação na próxima etapa, uma vez que permite ajustar o plano de ensino para a necessidade do treinando.^1^ Ao menos 25% da casuística deve ser de casos patológicos, a qual pode ser complementada com vídeos.^2^ Ver Figuras 2 e 3.^3^ O ideal é que as imagens do POCUS sejam enviadas em formato DICOM para o sistema de imagens do hospital (média de 10 imagens dinâmicas), mas não obrigatoriamente.^4^ Esses vídeos devem ser disponibilizados no site da instituição, com questões de múltipla escolha, gabarito e comentários, mas também os treinandos também devem acessar vídeos educacionais de sociedades especializadas.^5^

**Figura 3:**
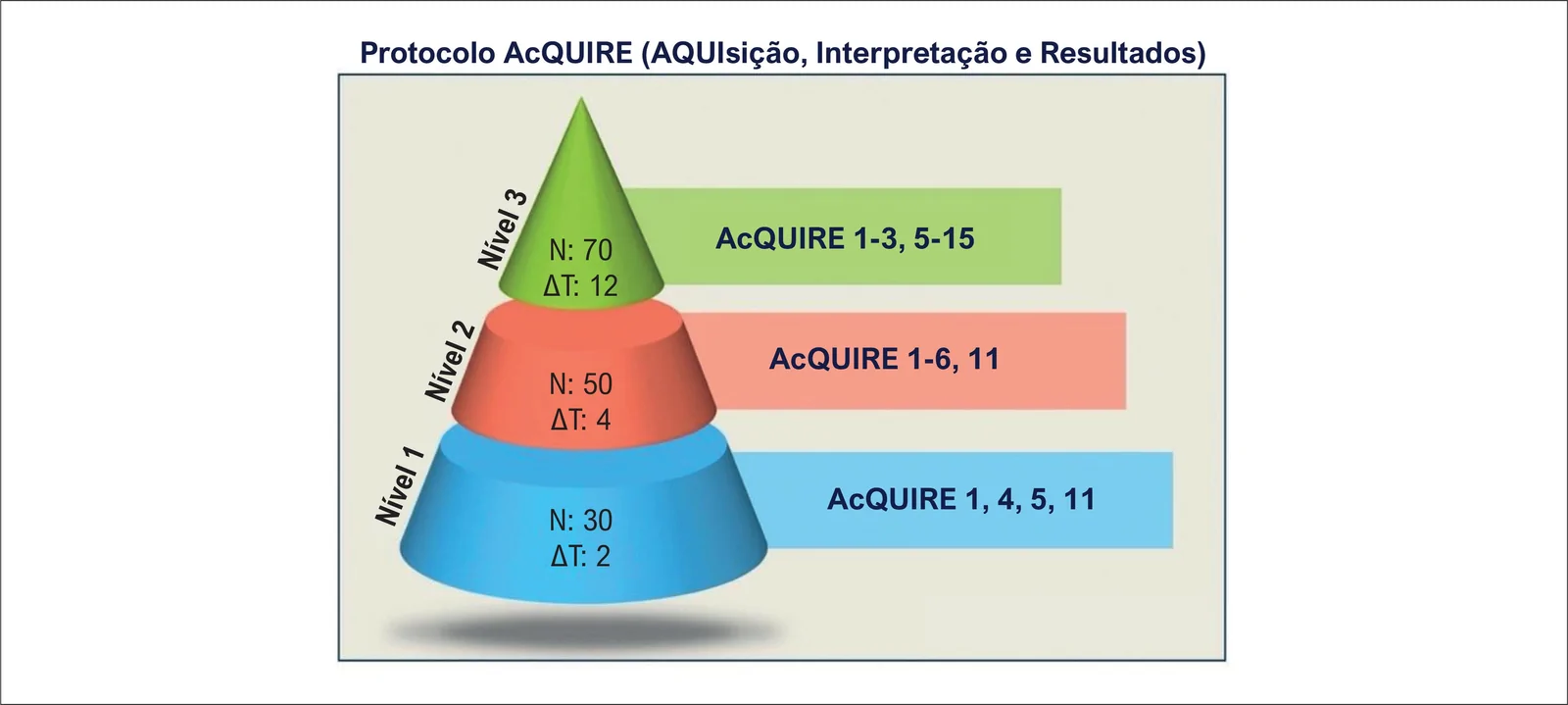
Protocolo de treinamento em POCUS por níveis, com número de exames e tempo de treinamento minimamente desejável de cada nível. Para o nível 1, por exemplo, é preciso adquirir as habilidades de AQUisição, Interpretação e documentação dos REsultados (AcQUIRE), descritos nos itens 1, 4, 5 e 11 da Tabela 1. N: número mínimo de exames; ΔT: semanas de treinamento.

**Figura 4:**
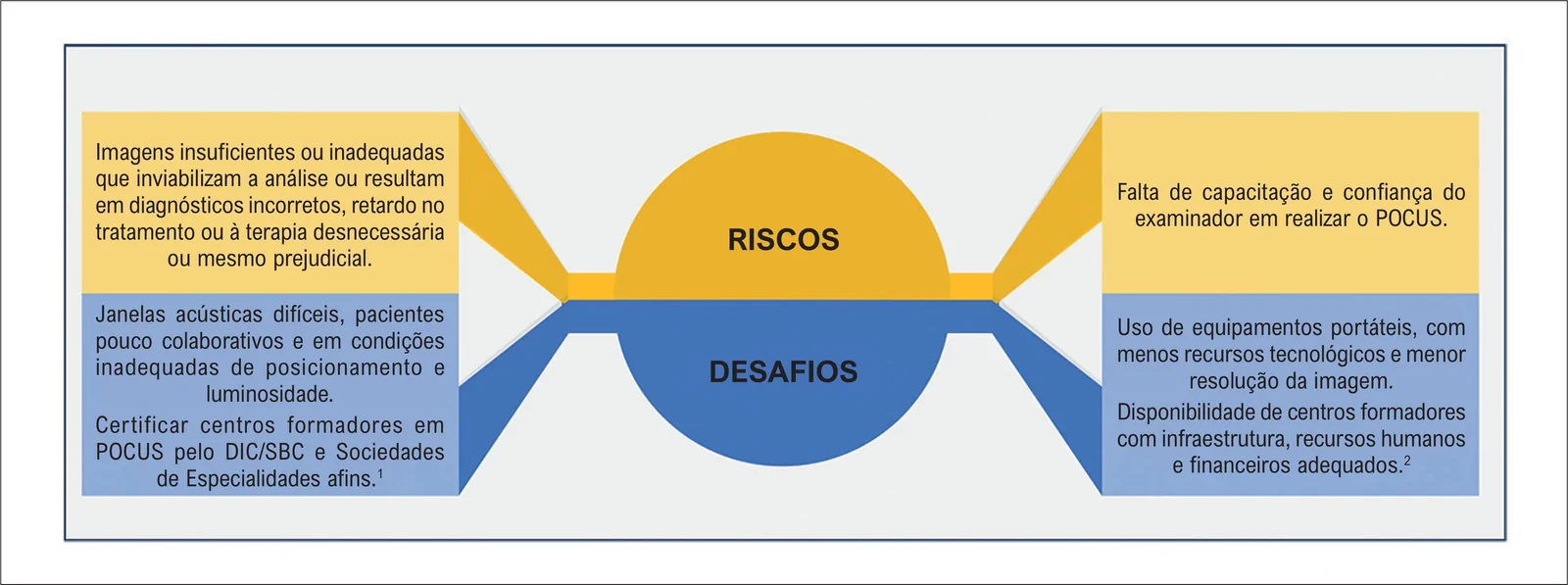
Riscos e desafios envolvidos no POCUS. O campo de ação do POCUS vai além da avaliação cardíaca e pode exigir parcerias com outros especialistas, além do ecocardiografista, tais como angiologista, radiologista, intensivista ou cardiologista pediátrico.^1^ Até o momento, não há previsão de reembolso para o POCUS nem verbas de fomento financeiro do centro formador.^2^

### 1.5. Aspectos Ético-Legais do Uso do POCUS Cardiovascular no Brasil

O POCUS cardiovascular deve ser entendido como um ato médico realizado à beira do leito, com a finalidade de responder a perguntas clínicas específicas e apoiar decisões imediatas. Ele não substitui, quando indicado, o exame ecocardiográfico diagnóstico completo.^9,10^ Nesse contexto, o médico que utiliza o POCUS assume responsabilidade civil direta pelos atos praticados, incluindo a indicação do exame, a execução técnica adequada, a interpretação correta dos achados e o devido registro em prontuário, conforme os princípios estabelecidos no Código de Ética Médica.^9,11^ A responsabilização profissional decorre não apenas de eventuais erros técnicos, mas também do uso do método fora do escopo de competência do profissional, sem capacitação adequada ou em desacordo com protocolos institucionais.^11,12^

No Brasil, a formação em POCUS ainda apresenta inserção limitada nos currículos de graduação e residência médica, o que faz com que os profissionais dependam de cursos extracurriculares e leva à heterogeneidade na qualificação.^13,14^ A implantação de programas estruturados de POCUS cardiovascular exige investimentos em equipamentos, infraestrutura digital, treinamento supervisionado e mecanismos de governança clínica.^15^ Apesar dos custos iniciais, estudos nacionais demonstram impacto positivo na eficiência diagnóstica e na racionalização do uso de exames complementares. Isso sugere uma relação custo-efetividade favorável, especialmente em cenários de urgência, terapia intensiva e em regiões com acesso restrito à ecocardiografia convencional.^13,15^ Nesse contexto, a integração com estratégias de telessaúde fortalece o uso do Tele-POCUS como ferramenta para ampliar o acesso à avaliação cardiovascular especializada no âmbito do Sistema Único de Saúde (SUS), promovendo maior equidade assistencial.^16^

Entretanto, ainda há desafios estruturais relevantes, como a ausência de codificação padronizada e de políticas claras de reembolso para o POCUS cardiovascular, tanto no SUS quanto na saúde suplementar. Isso compromete a sustentabilidade financeira de programas institucionais.^17,18^ Soma-se a isso a necessidade de atenção específica à cobertura por seguros de responsabilidade profissional, dada a inexistência de regulamentação formal que defina o escopo de atuação, os critérios mínimos de capacitação e a certificação de competência, o que gera insegurança jurídica em contextos de judicialização.^11,12^

Assim, ainda que instituições de saúde possam estabelecer normas internas de treinamento, supervisão e documentação, é prioritária a regulamentação do uso do POCUS pelo Conselho Federal de Medicina, em articulação com as sociedades médicas de especialidade. É preciso uma definição clara do escopo de atuação, dos requisitos mínimos de capacitação e certificação e da diferenciação em relação aos exames ecocardiográficos diagnósticos completos.^10,11^ Tal regulamentação é fundamental para garantir segurança jurídica aos profissionais, proteção aos pacientes, padronização da prática clínica e incorporação responsável e sustentável do POCUS cardiovascular no sistema de saúde brasileiro.

## 2. POCUS Cardíaco

### 2.1. Principais Janelas no POCUS Cardíaco

Ao contrário da ecocardiografia tradicional, o POCUS cardíaco utiliza imagens geralmente de fácil obtenção e análise, funcionando como extensão ao exame físico. O POCUS cardíaco é usado para o auxílio no diagnóstico e na conduta de diversas situações de emergência, como a investigação do choque circulatório e o manejo volêmico de pacientes críticos.^21^

São utilizadas cinco janelas ecocardiográficas principais utilizadas no POCUS (Figura 5):^22^

1. Janela paraesternal longitudinal (ou eixo longo do coração), obtida com o transdutor posicionado ao lado da linha paraesternal esquerda, entre o 3° e o 4° espaços intercostais, com o índice apontado para o ombro direito do paciente;
2. Janela paraesternal transversal (ou eixo curto do coração), obtida no mesmo local da janela paraesternal longitudinal, porém com rotação de 90° do transdutor, que passa a apontar para o ombro esquerdo do paciente. Angulações discretas do transdutor permitem mudar o plano de corte bidimensional e obter imagens "tomográficas" ao nível dos vasos da base, dos músculos papilares e dos segmentos apicais do ventrículo esquerdo;
3. Janela apical 4 câmaras (A4C), obtida com o transdutor posicionado na linha médio-clavicular esquerda, entre o 4° e 5° espaço intercostal, abaixo do mamilo ou da linha infra mamária, ao nível do *ictus cordis* (ponto de maior impulso apical), com o índice apontado para a esquerda do paciente;
4. Janela subcostal, obtida com o transdutor posicionado na linha média, abaixo do processo xifoide, formando um ângulo agudo com a parede abdominal e com o índice apontado para a esquerda do paciente;
5. Janela subcostal com foco na veia cava inferior (VCI), obtida no mesmo local da janela subcostal, mas com o transdutor perpendicular à parede abdominal e o índice apontado para a cabeça do paciente.

**Figura 5:**
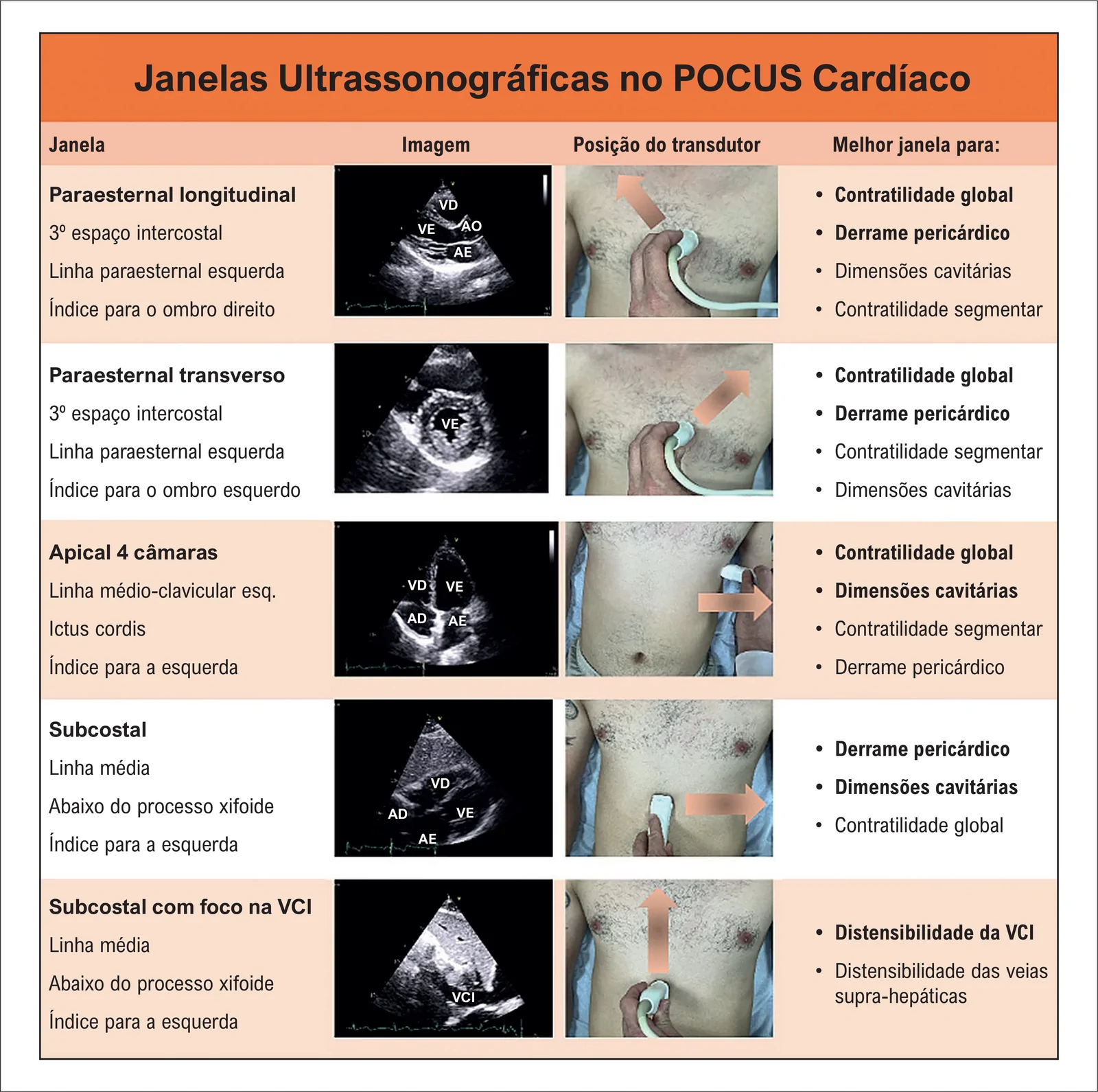
Principais janelas utilizadas no POCUS cardíaco, com respectivos posicionamentos da sonda e estruturas avaliadas.

### 2.2. Análise da Função Sistólica Global do Ventrículo Esquerdo

#### 2.2.1. Aspectos Gerais

A fração de ejeção do ventrículo esquerdo (FEVE) é a medida mais validada e comumente utilizada para avaliar a função sistólica.^23^ A disfunção do ventrículo esquerdo (VE) pode causar condições clínicas agudas ou agravá-las gravemente. Em casos de choque indiferenciado, a avaliação focada da função do VE tem apresentado aumento na acurácia diagnóstica e melhor orientação das decisões de tratamento com base em uma abordagem qualitativa.^24^ Neste documento, recomenda-se uma avaliação mais simplificada e objetiva, com impacto na tomada de decisão.

#### 2.2.2. Formas de Avaliação da Função Sistólica do ventrículo Esquerdo

A avaliação da função do VE deve ser realizada em todas as janelas ecocardiográficas disponíveis no paciente (conforme descrito no tópico 2.1. Principais janelas no POCUS cardíaco).

Métodos tradicionais de determinação da função do VE, como o Simpson biplanar ou Teicholz, geralmente não são utilizados no POCUS cardíaco. Esses métodos requerem treinamento adicional, consomem mais tempo, estão sujeitos a erros e não resultam necessariamente em maior acurácia. Assim, o POCUS cardíaco adota uma abordagem qualitativa.

Unlüer et al.*,*^25^ bem como outros autores, demonstraram uma boa correlação entre a avaliação visual do emergencista em comparação com o exame realizado por especialistas.

Nessa avaliação qualitativa, é preciso considerar a excursão do endocárdio (movimento das paredes em direção ao centro do VE) e o espessamento miocárdico (aumento da espessura a cada contração). Isso permite uma classificação mais ampla da função do VE em 4 categorias: 1) normal, 2) hiperdinâmico, 3) função sistólica reduzida, 4) disfunção grave (Tabela 2). A classificação de função sistólica reduzida inclui os pacientes com disfunção leve ou moderada ao ecocardiograma.

**Tabela 2:** Avaliação qualitativa da função sistólica do ventrículo esquerdo

**Normal**	Espessamento da maior parte dos segmentos do VE, com deslocamento do endocárdio em direção ao centro do VE.
**Hiperdinâmico**	Excursão endocárdica e espessamento miocárdico aumentado, levando a uma obliteração sistólica do VE parcial ou total.
**Função sistólica reduzida**	Redução da excursão endocárdica e espessamento miocárdico.
**Disfunção grave**	Grave redução da excursão endocárdica e espessamento miocárdico.

Veja exemplos nos vídeos 1 a 13 no Suplemento 2.

O objetivo dessa classificação é ser mais intuitiva e clinicamente relevante na assistência ao paciente.^26^

#### 2.2.3. Outras Técnicas de Avaliação da Função Sistólica do Ventrículo Esquerdo

Outras formas semiquantitativas, como MAPSE (*mitral annular plane systolic excursion*) e EPSS (*E-point septal separation*), podem ser utilizadas, mas cada uma possui limitações inerentes.^27,28^ Para informações mais detalhadas sobre cada um desses itens, consulte as referências bibliográficas.

Estudos demonstram uma boa correlação da medida automatizada da fração de ejeção feita por IA, desde que haja boa aquisição das imagens. A IA representa uma ferramenta promissora nesse campo.^29^

### 2.3. Pesquisa de Sobrecarga e Função Sistólica do Ventrículo Direito

Os achados ao POCUS cardíaco podem auxiliar no diagnóstico rápido de condições patológicas que acometem de forma primária ou secundária o ventrículo direito (VD). Esses dados são valiosos para a instituição de terapia de suporte hemodinâmico adequado e/ou tratamentos específicos mais rapidamente.

Na abordagem de pacientes com dor torácica e/ou dispneia aguda, sobretudo na presença de sinais de instabilidade hemodinâmica ou insuficiência cardíaca (IC), é importante afastar diagnósticos diferenciais como hipovolemia, disfunção sistólica grave do VE, tamponamento cardíaco, disfunção valvar aguda ou mesmo dissecção aórtica.^30^

Em um paciente instável com sobrecarga aguda e/ou disfunção do VD, os seguintes diagnósticos devem ser considerados: (1) tromboembolismo pulmonar (TEP) agudo; (2) infarto agudo do miocárdio (IAM) de VD; e (3) disfunção do VD em pacientes com IC aguda causada por etiologias diversas, como IAM, miocardite, sepse, cardiomiopatia periparto, valvopatia aguda e cardiomiopatia adrenérgica (Takotsubo).

O aumento do VD pode ser evidenciado no corte paraesternal longitudinal (Figura 6a) ou na janela A4C (Figura 6b). Na janela apical, a área do VD normalmente corresponde a ≤ 2/3 da área VE; quando essa relação ultrapassa 1,0, o achado é sugestivo de importante aumento e sobrecarga do VD.^31^

**Figura 6:**
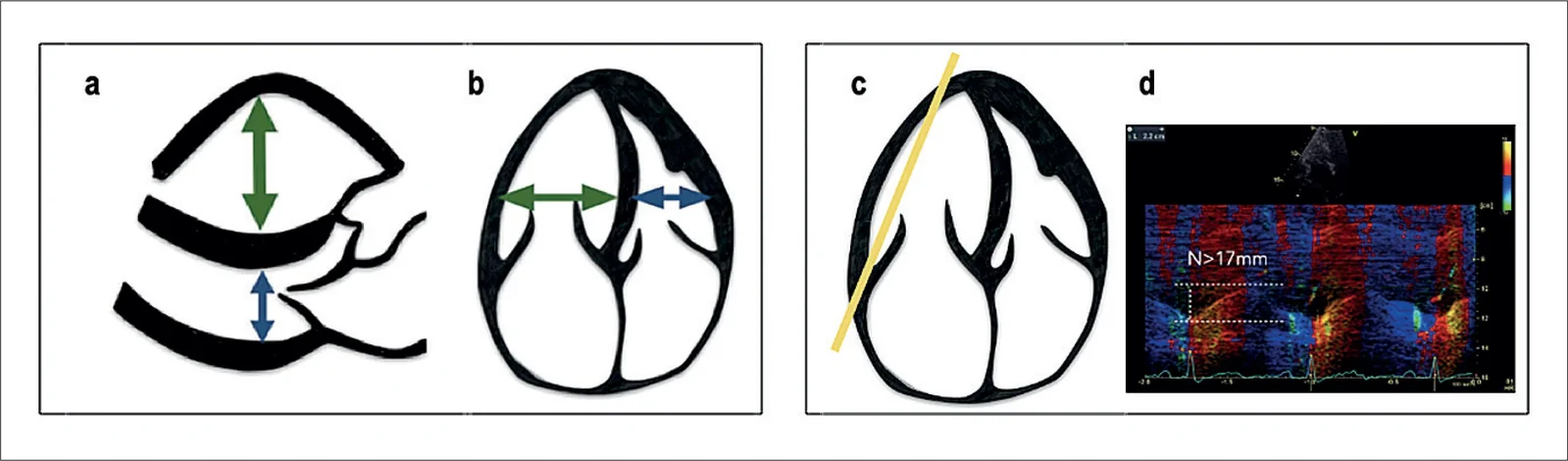
a e b: Pesquisa da sobrecarga por meio da relação VD/VE; c e d: função sistólica do VD por meio da excursão sistólica do anel tricúspide lateral (TAPSE) medida ao modo-M. VD: ventrículo direito; VE: ventrículo esquerdo.

A avaliação da função sistólica do VD é bastante complexa, o que limita a acurácia da análise subjetiva. Isso ocorre devido à geometria singular da cavidade, em forma de crescente triangular, com vias de entrada e de saída dispostas em diferentes planos, além de importante influência de pré e pós-carga e de interdependência ventricular.^32^ Além da análise subjetiva, recomenda-se avaliar a função sistólica do VD com medidas objetivas simples, como a excursão sistólica do anel tricúspide lateral (parede livre do VD) (TAPSE). A TAPSE se encontra reduzida em pacientes com TEP agudo com sobrecarga hemodinâmica e possui valor prognóstico.^33^ A TAPSE é medida a partir de janela A4C, sendo considerada anormal quando <1,7 cm (Figuras 6c e 6d).

#### 2.3.1. Embolia Pulmonar

A utilização do POCUS cardíaco na suspeita de TEP agudo é valiosa devido à agilidade na obtenção de informações relevantes para o diagnóstico e avaliação prognóstica desses pacientes. Isso é especialmente importante para os pacientes que apresentam instabilidade hemodinâmica, com dificuldade de deslocamento para angiotomografia (angio-TC) pulmonar ou que não podem aguardar a realização de um exame ecocardiográfico completo. É importante ressaltar que os achados ecocardiográficos indicativos de possível embolia pulmonar estão presentes apenas quando há sinais de sobrecarga aguda do VD. Isso geralmente ocorre em casos de embolia pulmonar com repercussão hemodinâmica (maciça ou submaciça). Assim, o POCUS contribui para a estratificação e auxilia na definição de estratégia terapêutica.^34^ A evidência de dilatação do VD, com aplainamento septal (septo em "D") (Figuras 7a e 7b) e, em alguns casos, com alteração contrátil evidente, mais proeminente nos segmentos médio e basal e com preservação da contratilidade apical (sinal de McConnell), pode auxiliar no diagnóstico. Apesar de ser um achado clássico, o sinal de *McConnell* está presente em apenas 12-20% dos pacientes.^35,36^ Em pacientes com TEP agudo, a presença de congestão venosa sistêmica por insuficiência ventricular direita também é um achado comum. Com frequência, a VCI encontra-se dilatada, associada a dinâmica respiratória reduzida, refletindo o aumento da pressão estimada do átrio direito.

**Figura 7:**
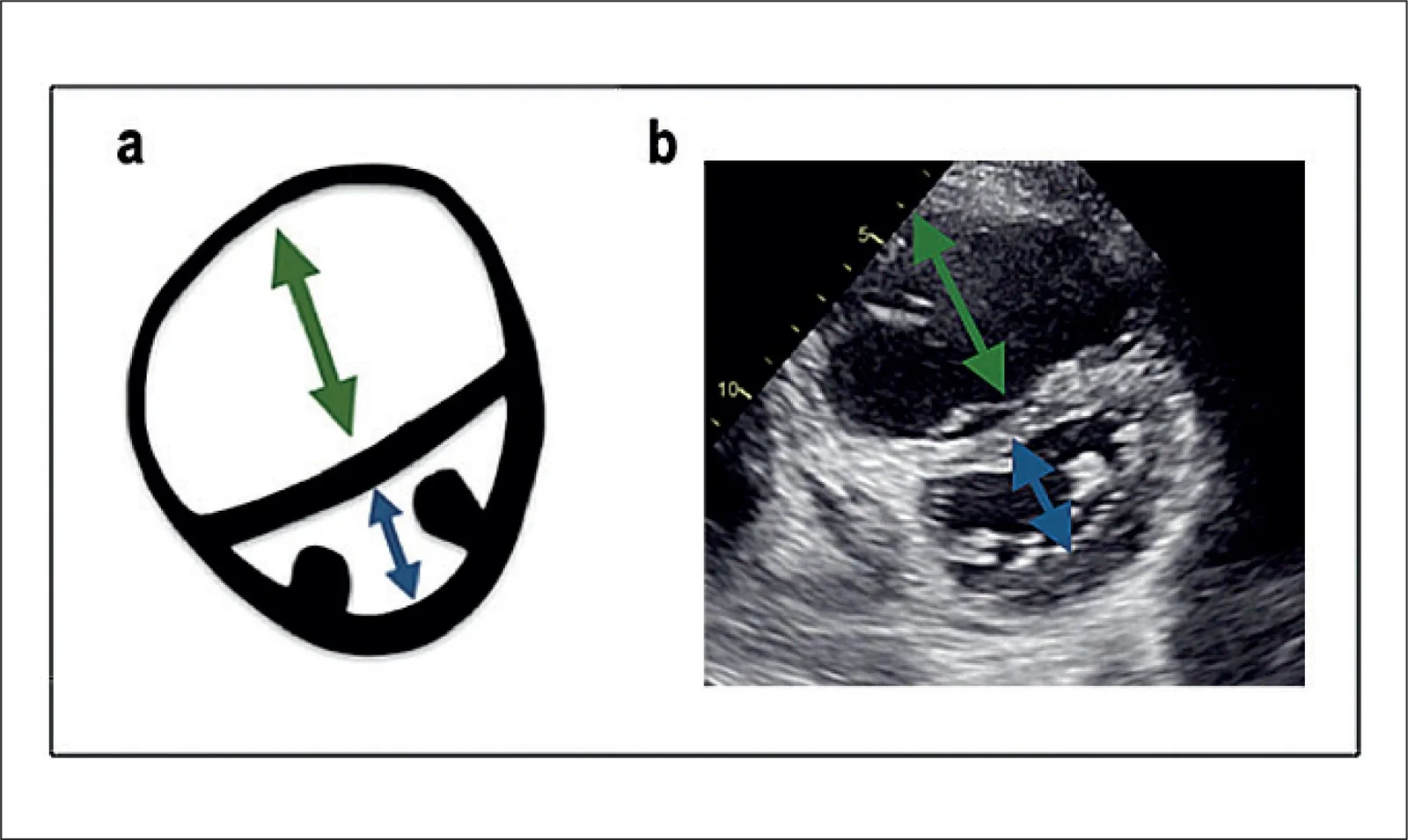
Dilatação do VD com aplainamento septal (septo em "D"). VD: ventrículo direito.

Cerca de 5% dos casos de pacientes admitidos em parada cardiorrespiratória nos serviços de emergência podem ter TEP agudo como causa subjacente. Nesses casos, o diagnóstico rápido pode proporcionar um melhor prognóstico, com indicação mais precoce de terapia trombolítica ou tromboendarterectomia pulmonar percutânea.^37^

Em casos de TEP agudo, raramente (<5%) é possível identificar trombos no interior das cavidades direitas^10^ ou impactados "em sela" na bifurcação pulmonar. Além de confirmar o diagnóstico, esses achados também estão associados a prognóstico pior, sobretudo nos pacientes que evoluem com disfunção sistólica do VD.^38–40^

A ecocardiografia tem sensibilidade reportada em torno de 60-70%, de modo que um teste negativo não exclui o diagnóstico.^41^ O uso combinado de POCUS cardíaco, ultrassonografia (US) venosa de membros inferiores e US pulmonar (USP), também denominado "triplo POCUS", aumenta a acurácia diagnóstica para a detecção de embolia pulmonar. Em um estudo multicêntrico com 357 pacientes com suspeita clínica de embolia pulmonar, Nazerian et al.^42^ compararam a acurácia do triplo POCUS com os achados da angio-TC. O triplo POCUS demonstrou sensibilidade (Sen) de 90% e especificidade (Esp) de 86%, muito superior ao uso isolado das técnicas (USP – Sen: 61% / Esp: 96%, POCUS cardíaco – Sen: 33% / Esp: 91% e US venoso de membros inferiores – Sen: 53% /Esp: 98%).

Em pacientes hemodinamicamente instáveis, o uso de POCUS cardíaco e US venoso de membros inferiores, antes mesmo da angio-TC, pode reduzir o tempo de diagnóstico e melhorar a resposta ao tratamento.^43,44^ A Sociedade Europeia de Cardiologia recomenda que, em pacientes com quadro clínico suspeito e evidências ecocardiográficas sugestivas de embolia pulmonar aguda, a terapia trombolítica pode ser justificada de imediato quando outros testes não estão prontamente disponíveis ou quando o atraso no tratamento pode significar um risco maior para o paciente.^41^

#### 2.3.2. POCUS para Avaliação de Infarto do Ventrículo Direito

O infarto do VD pode ocorrer em 30-50% dos pacientes com IAM de parede inferior e pode complicar o manejo hemodinâmico desses pacientes. O infarto do VD pode evoluir com bradicardia, necessidade de implante de marcapasso, hipotensão e morte. A presença de alterações segmentares na parede inferior inferolateral e um padrão de alteração segmentar do VD sustentam a suspeita de infarto do VD.^45^ Os principais diagnósticos diferenciais são: 1) cardiomiopatias adrenérgicas (Takotsubo), que também podem causar alteração segmentar do VD, geralmente com acinesia apical, acompanhando a alteração similar de "balonamento apical" em região análoga no VE; 2) alterações contráteis segmentares conhecidas como sinal de McConell, com contratilidade apical preservada e alterações dos segmentos médio e basal, descritas no TEP.

#### 2.3.3. Outras Aplicações

Em pacientes críticos, o POCUS cardíaco pode ser útil para avaliar a evolução da função sistólica do VD e facilitar o manejo hemodinâmico dos pacientes. Por exemplo: (1) quando submetidos a recrutamento pulmonar como estratégia de ventilação mecânica (acoplamento ventrículo-arterial),^46^ (2) em pacientes com insuficiência ventricular direita para avaliação evolutiva de dimensões cavitárias e de débito cardíaco; ou (3) no acompanhamento de pacientes em uso de suporte circulatório com oxigenação por membrana extracorpórea (ECMO) e sinais de instabilidade, em cujo caso auxilia a avaliar a descompressão de cavidade, o posicionamento das cânulas e a possibilidade de obstrução.^47^

#### 2.3.4. Limitações

O uso de POCUS cardíaco para a avaliação dos diâmetros e função sistólica do VD tem limitações. A obtenção de uma janela adequada pode ser desafiadora, com grande variabilidade de acordo com a experiência e habilidade do examinador. Além disso, o VD é extremamente sensível à pré e pós-carga e à interdependência com o a função do VE, fatores que devem ser considerados em cada análise evolutiva.

### 2.4. POCUS no Tamponamento Cardíaco

Embora raro, o tamponamento cardíaco, condição na qual o acúmulo de líquido no espaço pericárdico comprime o coração e impede seu enchimento adequado, configura uma emergência médica.^48^ Em seus estágios mais avançados, essa restrição leva à redução substancial do débito cardíaco e da perfusão sistêmica e coronariana e pode culminar em queda abrupta da pressão arterial e da frequência cardíaca.^49^

Os sinais clínicos clássicos do tamponamento cardíaco, conhecidos como tríade de Beck (hipotensão arterial, hipofonese de bulhas cardíacas e turgência jugular) nem sempre estão presentes, ou surgem tardiamente, de modo que o POCUS é uma ferramenta valiosa para a detecção precoce da condição.^50^

A quantificação do volume pericárdico com base na distância entre o pericárdio parietal e o visceral ao fim da diástole (Tabela 3), embora simples de realizar pelas janelas paraesternal e subcostal, não permite o diagnóstico de tamponamento cardíaco, uma vez que demais fatores, como velocidade de instalação e natureza do fluido (transudato, exsudato, sangue) podem levar ao aumento rápido da pressão intrapericárdica mesmo com pequenos volumes.^49^

**Tabela 3:** Semiquantificação do derrame pericárdico de acordo com a medida telediastólica e volume do líquido

Medida telediastólica	Volume aproximado de líquido
5-10 mm	100-250 mL (pequeno)
10-20 mm	250-500 mL (moderado)
>20 mm	>500 mL (importante)
>25 mm	Muito importante

Adaptado de Klein et al.^49^

A distensão da VCI e o colabamento das cavidades direitas na presença de derrame pericárdico são achados fundamentais para a confirmação da fisiologia do tamponamento cardíaco quando há suspeita clínica (Figura 8), embora variem substancialmente em relação à sensibilidade e à especificidade.^50^ A distensão da VCI e das veias supra-hepáticas e o colabamento do átrio direito na sístole ventricular são achados com alta sensibilidade, enquanto o colabamento diastólico do VD é o achado mais específico de restrição ao enchimento diastólico ao POCUS.

**Figura 8:**
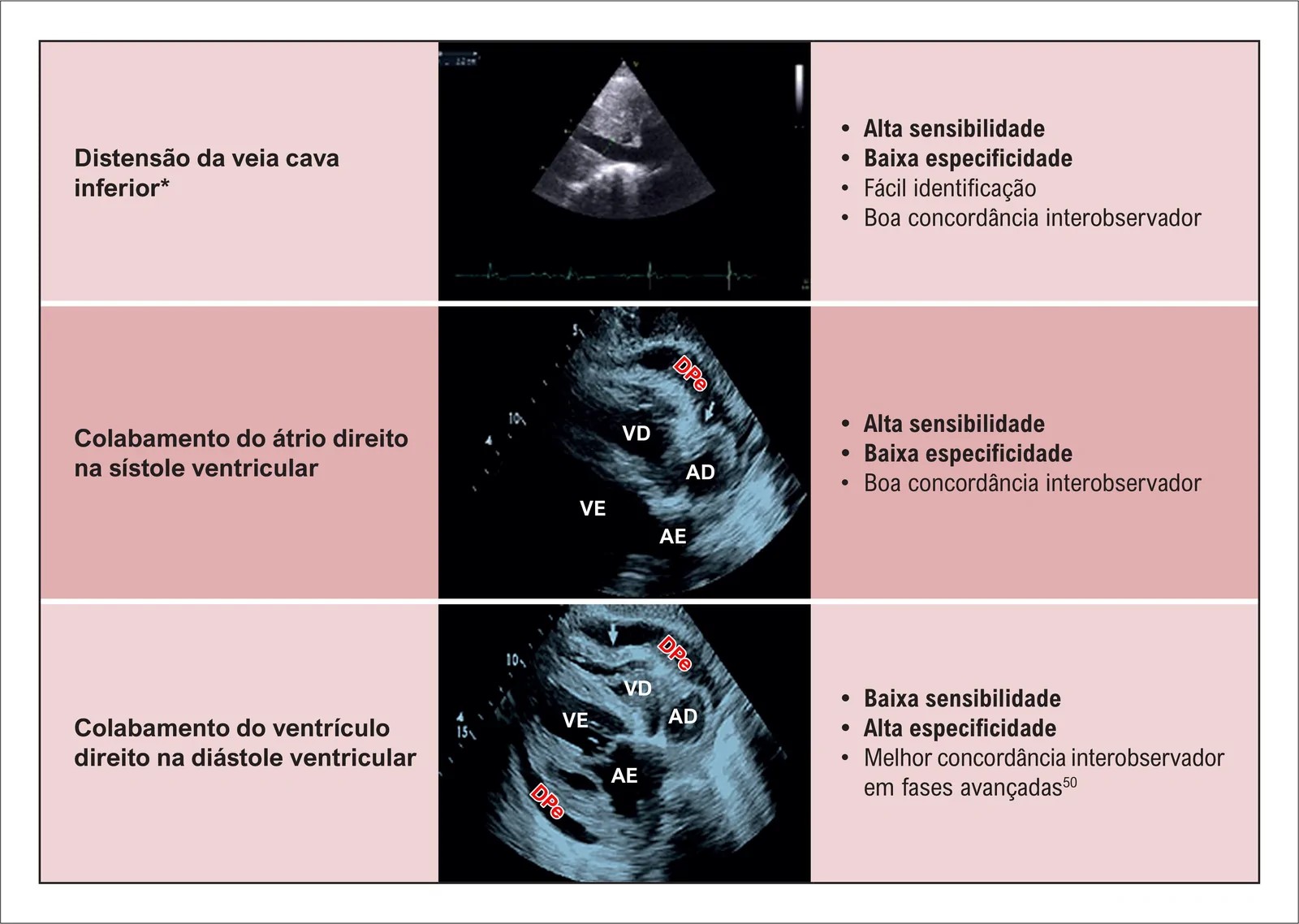
Achados fundamentais de tamponamento cardíaco ao POCUS. *A distensão da VCI pode estar ausente no tamponamento cardíaco com hipovolemia acentuada. Outros achados podem contribuir para o diagnóstico, como o desvio do septo interventricular para a esquerda durante a inspiração (parte da fisiologia do pulso paradoxal).^49^

A diferenciação entre derrame pericárdico e derrame pleural não é sempre simples e merece atenção durante o POCUS. Na janela paraesternal longitudinal, derrames anteriores à aorta descendente normalmente são pericárdicos, enquanto os posteriores ao vaso são de origem pleural (Figura 9).

**Figura 9:**
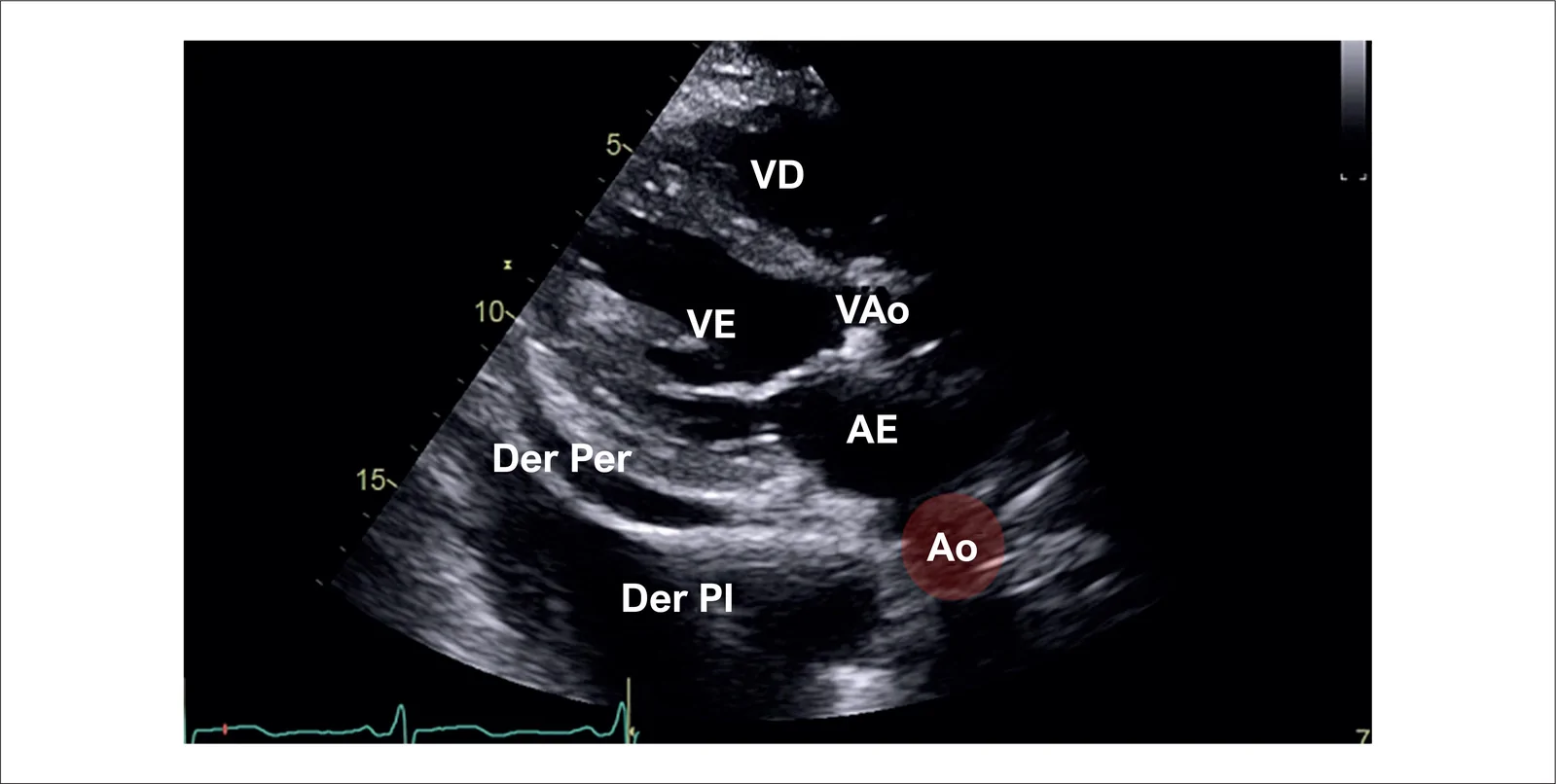
Diferenciação entre derrame pleural (posterior à aorta) e pericárdico (anterior à aorta).

### 2.5. POCUS Avançado

O POCUS avançado abrange o desenvolvimento de habilidades progressivas durante o treinamento na técnica, envolvendo a avaliação de parâmetros hemodinâmicos obtidos por ultrassonografia. É importante ressaltar que cada uma dessas habilidades possui uma curva de aprendizado. Recomenda-se que essa curva seja seguida para que o POCUS possa ser aplicado com a maior acurácia possível. Neste tópico, são abordadas a avaliação da TVI (integral velocidade-tempo) da via de saída do VE (VSVE), a determinação do volume sistólico e do débito cardíaco e a avaliação da relação E/e’ e seu papel na determinação da fluido-tolerância.

#### 2.5.1. Avaliação da Integral Velocidade-Tempo da Via de Saída do Ventrículo Esquerdo, Determinação do Volume Sistólico e Débito Cardíaco

O volume sistólico é calculado pelo produto da TVI da VSVE pela área da VSVE. A área da VSVE é derivada do seu diâmetro medido na janela paraesternal longitudinal (Figura 10). Em geral, a TVI da VSVE é obtida na janela apical de 5 câmaras (após adquirir a janela A4C, realiza-se uma anteriorização do transdutor para obtenção da imagem). Para essa medida, utiliza-se o Doppler pulsátil (PW), com volume da amostra na VSVE. Após o registro espectral, o envelopamento desse fluxo gera um valor em centímetros. Entendendo a VSVE como um cilindro, a integral velocidade-tempo representa a distância que o sangue percorre da VSVE até a aorta a cada contração cardíaca (Figura 10).

**Figura 10:**
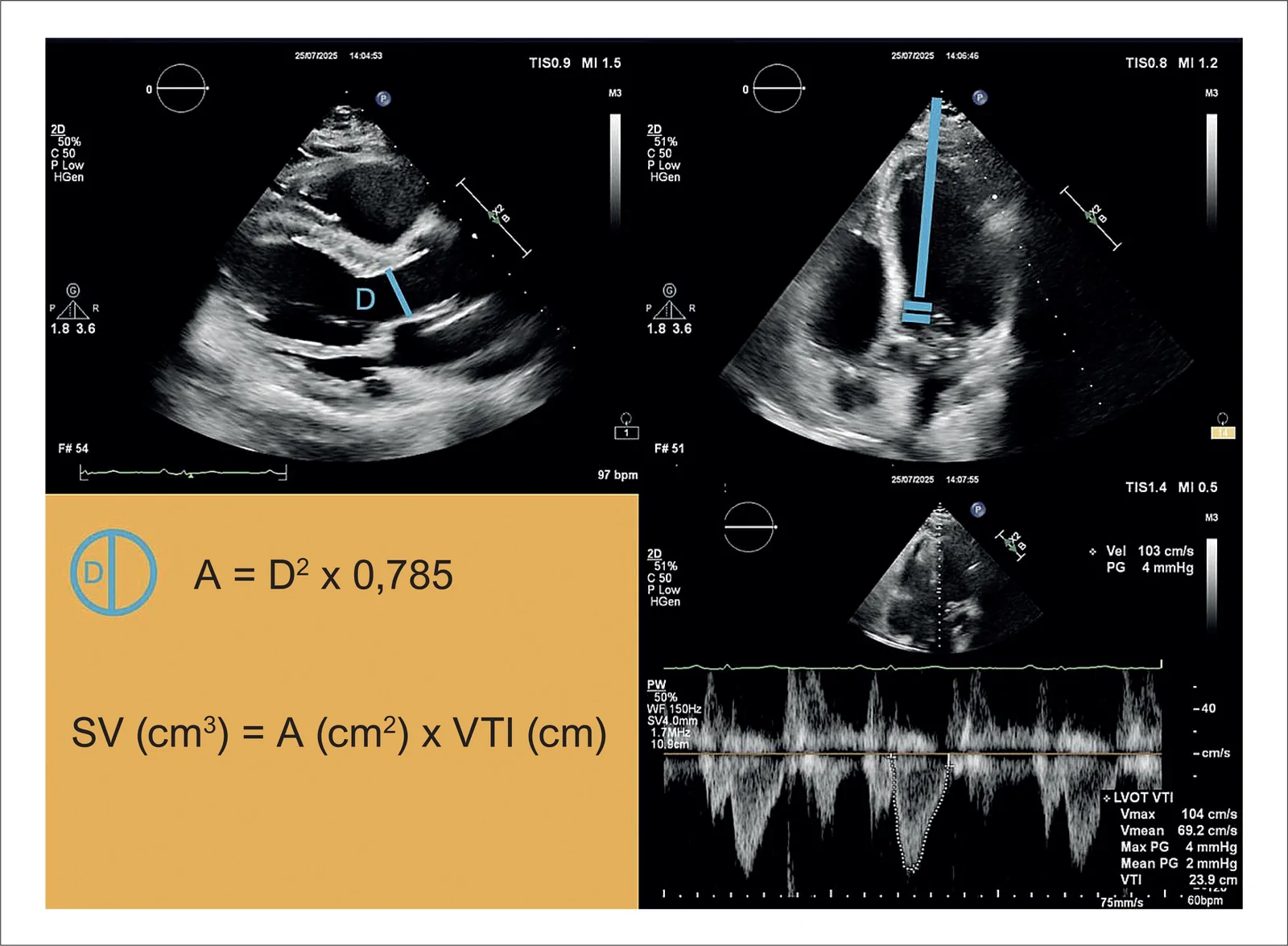
Cálculo do volume sistólico pelo produto da área da VSVE obtida na janela paraesternal pela integral do fluxo da VSVE obtido pela janela apical 5 câmaras.^51^ VSVE: via de saída do ventrículo esquerdo.

O uso da área da VSVE tem desvantagens. Como o cálculo segue a fórmula D^2^ × 0,785, onde D é o diâmetro, qualquer erro de medição é elevado ao quadrado, resultando em um erro significativo no cálculo do volume sistólico. Infelizmente, erros nessa medida não são incomuns. Logo, é necessária uma alternativa ao volume sistólico em situações de emergência.

Dado que o diâmetro da VSVE (e, logo, a área) tende a permanecer constante, variações no volume sistólico refletem mudanças da TVI da VSVE. Dessa forma, a TVI da VSVE é um possível substituto para o volume sistólico e um parâmetro hemodinâmico importante para a monitorização das respostas e orientação das terapias durante a avaliação inicial e ao longo do tratamento.

Para calcular o débito cardíaco, multiplica-se o volume sistólico pela frequência cardíaca (FC).^52^

Em indivíduos saudáveis, a TVI da VSVE normalmente varia entre 18 e 22 cm, com FC entre 55 e 95 bpm. Quando a FC é inferior a 55 bpm, a TVI obtida deve ser maior que 18 cm. De forma semelhante, em pacientes com FC acima de 95 bpm, a TVI da VSVE deve ser inferior a 22 cm, ou então deve-se supor um estado de alto débito. A monitorização hemodinâmica funcional, no qual as mudanças na função cardíaca em resposta aos tratamentos são mais importantes que medidas estáticas, se baseia no conceito de "seguir tendências".^53^ A aplicação da TVI dentro de um algoritmo de diagnóstico e tratamento é abordada no tópico 6 (POCUS no paciente em choque).

#### 2.5.2. Relação E/e’ como Medida da Pressão Atrial Esquerda e Fluido-Tolerância

O aumento da pressão atrial esquerda (PAE) tem consequências importantes nas trocas gasosas, na pós-carga da hemodinâmica pulmonar e no desempenho do ventrículo direito.^54^

A elevação da PAE pode ser causada por condições pré-existentes, como disfunção sistólica e diastólica do VE e valvopatias mitrais e aórticas; entretanto o aumento agudo da PAE pode ser observado em pacientes críticos com sepse, isquemia miocárdica, cardiomiopatia induzida por estresse e sobrecarga volêmica.^55^

A relação entre a onda E do fluxo transmitral e a onda e’ obtida no anel mitral medial e lateral (relação E/e’ média) tem sido amplamente estudada na cardiologia e ganhou interesse na literatura sobre terapia intensiva. A relação E/e’ é menos dependente de carga e pode ser usada para estimar o aumento da PAE em diversos cenários (Figura 11). Embora um valor normal de E/e’ não exclua a elevação da PAE, um valor médio >14 tem alta especificidade para aumento da PAE. Essa informação é útil na tomada de decisões sobre mais ressuscitação com fluidos à beira do leito: nesse cenário, E/e′ >14 favorece fortemente um paciente com "fluido-intolerância". No outro extremo, E/e’ <8 tem boa acurácia para predizer PAE <18 mmHg.^56^

**Figura 11:**
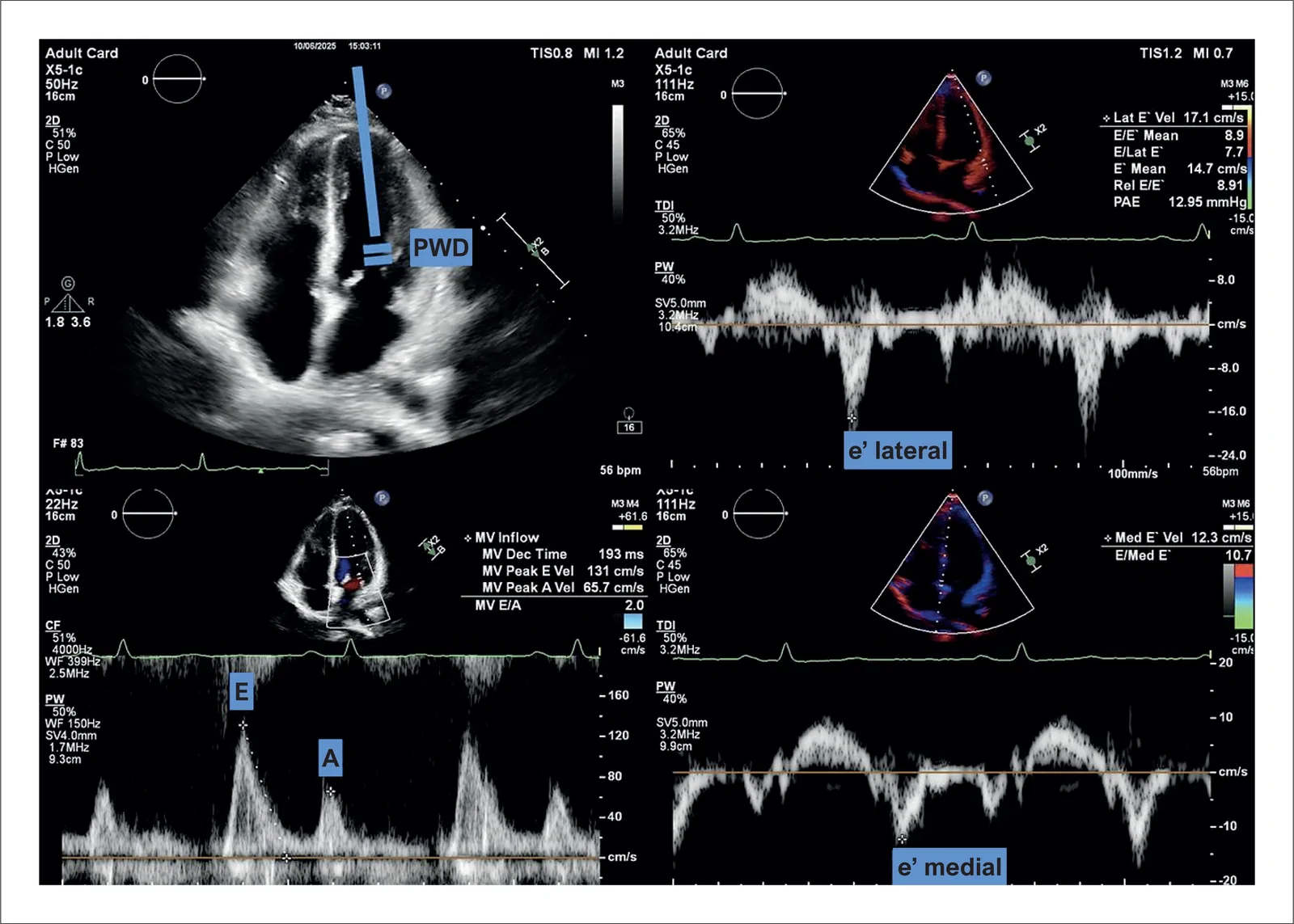
Cálculo da relação E/e’ obtido pela relação entre a onda E do fluxo mitral e a média da onda e’ obtida ao Doppler tecidual do anel mitral medial e lateral.

### 2.6. POCUS Cardíaco Pediátrico

Assim como nos pacientes adultos, o POCUS cardíaco na pediatria (≤ 18 anos) é uma extensão do exame clínico, realizado à beira do leito para auxiliar na conduta e manejo do paciente com agilidade. As principais indicações para o exame estão listadas no Quadro 1.

**Quadro 1:** Indicações principais do POCUS pediátrico

**1.** Avaliação qualitativa da função sistólica dos ventrículos
**2.** Avaliação de hipertensão pulmonar
**3.** Avaliação de derrame pericárdico e orientação para pericardiocentese
**4.** Avaliação de volemia e da responsividade à administração de fluidos
**5.** Avaliação da patência do canal arterial em neonatos

O POCUS cardíaco não inclui a avaliação da anatomia cardíaca nem o diagnóstico de cardiopatias congênitas. Também é diferente da chamada "*targeted neonatal echocardiography*" ou ecocardiografia neonatal direcionada, exame que utiliza múltiplos parâmetros ecocardiográficos para avaliar a função sistólica e diastólica e a volemia.^4,57^

## 3. Ultrassonografia Pulmonar

### 3.1. Introdução

A USP, descrita mais tardiamente em relação a outras aplicações da ultrassonografia no POCUS,^58^ tornou-se essencial para a avaliação rápida, à beira do leito e em tempo real de pacientes com queixa de dispneia. Quando associada à história clínica e ao exame físico, a USP permite acelerar o diagnóstico e o tratamento adequado do paciente.^59,60^ A USP detecta facilmente a presença de líquido extravascular pulmonar na congestão pulmonar. Seu desempenho diagnóstico é equivalente à dosagem de BNP e superior à da radiografia de tórax.^61,62^ A USP também auxilia em outros diagnósticos associados a comprometimento pulmonar, como derrame pleural, síndromes intersticiais, pneumonia, atelectasias, pneumotórax e tromboembolismo pulmonar. Essas condições serão discutidas a seguir.^63^

A USP se baseia em artefatos gerados da pleura, estrutura facilmente visível ao ultrassom. Em condições normais, o pulmão aerado reflete a energia ultrassônica e reverberações da linha pleural são projetadas em pontos equidistantes no campo ultrassônico, conhecidas como linhas A. O espessamento dos espaços interalveolares ou o preenchimento dos alvéolos por líquido ou processo inflamatório causa o borramento progressivo das linhas A e o surgimento de linhas B, que são perpendiculares e acompanham o deslizamento pleural. As linhas B se propagam por todo o campo ultrassônico.^64^

### 3.2. Aspectos Técnicos

Os aspectos técnicos do POCUS foram descritos anteriormente neste documento. No entanto, algumas observações específicas à USP se fazem necessárias.

#### 3.2.1. Equipamentos e Transdutores

Equipamentos de US simples e até mesmo os chamados dispositivos *handheld* obtêm imagens adequadas de USP. Caso o equipamento não tenha um *preset* pulmonar, o *preset* abdominal é considerado o mais apropriado para a obtenção de imagens devido à sua persistência mais elevada. Embora tenha persistência reduzida, o *preset* cardíaco adulto pode ser utilizado, sem prejuízo substancial das informações obtidas.^65^ Quatro tipos de transdutores podem ser utilizados na USP: convexo de 5 MHz, setorial de 3-5 MHz, microconvexo 5-8 MHz e linear de 5-12 MHz. O último é especialmente útil para a avaliação da linha pleural e do deslizamento pleural.^65^

### 3.3. Protocolo de Estudo

Entre os diversos protocolos de exame na literatura, recomenda-se três (Figura 12) que podem ser escolhidos de acordo com o contexto clínico. O protocolo mais utilizado é o BLUE (Figura 12a), sigla de *Bedside Lung Ultrasound in Emergency*. Nesse protocolo, cada hemitórax é dividido em três quadrantes: dois na face anterior de cada hemitórax, com foco na busca por linhas B e presença de pneumotórax, e o terceiro quadrante na região posterolateral basal, no qual se avalia a presença de derrame pleural e consolidações pulmonares.^66^

**Figura 12:**
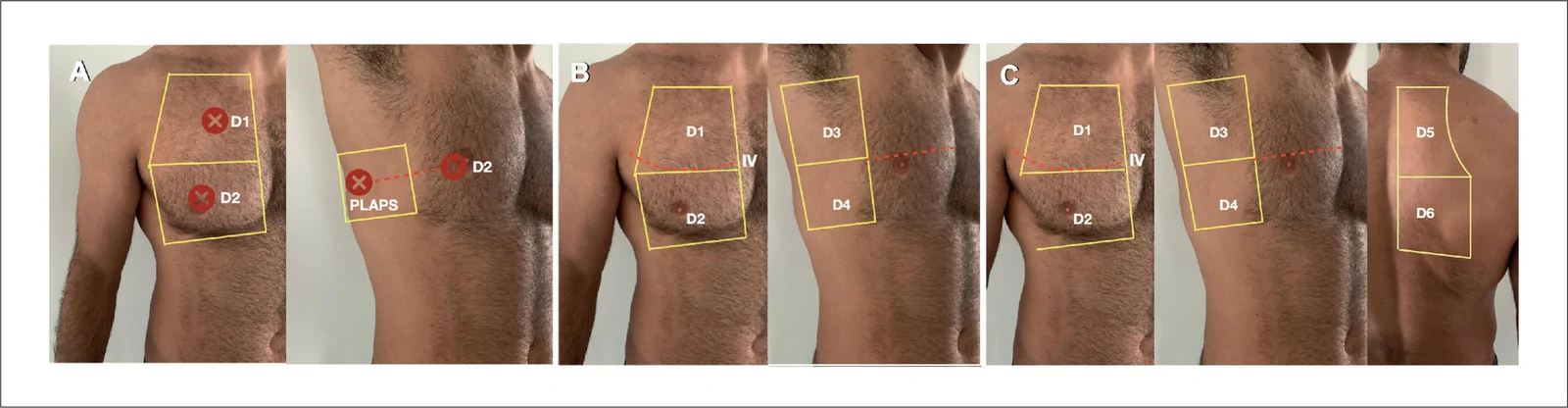
Protocolos de ultrassonografia pulmonar recomendados. A. BLUE (ref 56). B. 8 quadrantes (ref 60). C. 12 quadrantes (ref 62). Obs.: para maior detalhamento, utilizar as referências. BLUE: Bedside Lung Ultrasound in Emergency.

Com profundidade da imagem em torno de 15-18 cm, a sonda deve ser posicionada no plano sagital, com o índex em posição cranial. Com o paciente em decúbito dorsal, são realizadas varreduras cuidadosas em todo o território do quadrante, respeitando o ciclo respiratório completo e buscando o ponto com maior concentração de linhas B. Os quadrantes anteriores do tórax são chamados de pontos BLUE superior e inferior e, para fins de identificação, podem ser identificados no sentido craniocaudal como D1 e D2 no hemitórax direito e E1 e E2 no esquerdo. São nesses pontos que se busca identificar o perfil B, indicativo de congestão pulmonar, com sensibilidade de 97% e especificidade de 95%.^67^ O perfil B é caracterizado obrigatoriamente pela presença de três ou mais linhas B por quadrante na região anterior de cada hemitórax e deslizamento pleural preservado.^68,69^

O terceiro quadrante, conhecido como ponto PLAPS (*posterolateral alveolar and/or pleural syndrome)*, pode ser de difícil obtenção em pacientes impossibilitados de assumir o decúbito semilateral. A posição ideal permite a visualização do recesso costofrênico, do diafragma e do fígado ou baço. Nesse quadrante, o objetivo principal é a avaliação de derrame pleural e de possíveis consolidações pulmonares pneumônicas (Figura 13f).

**Figura 13:**
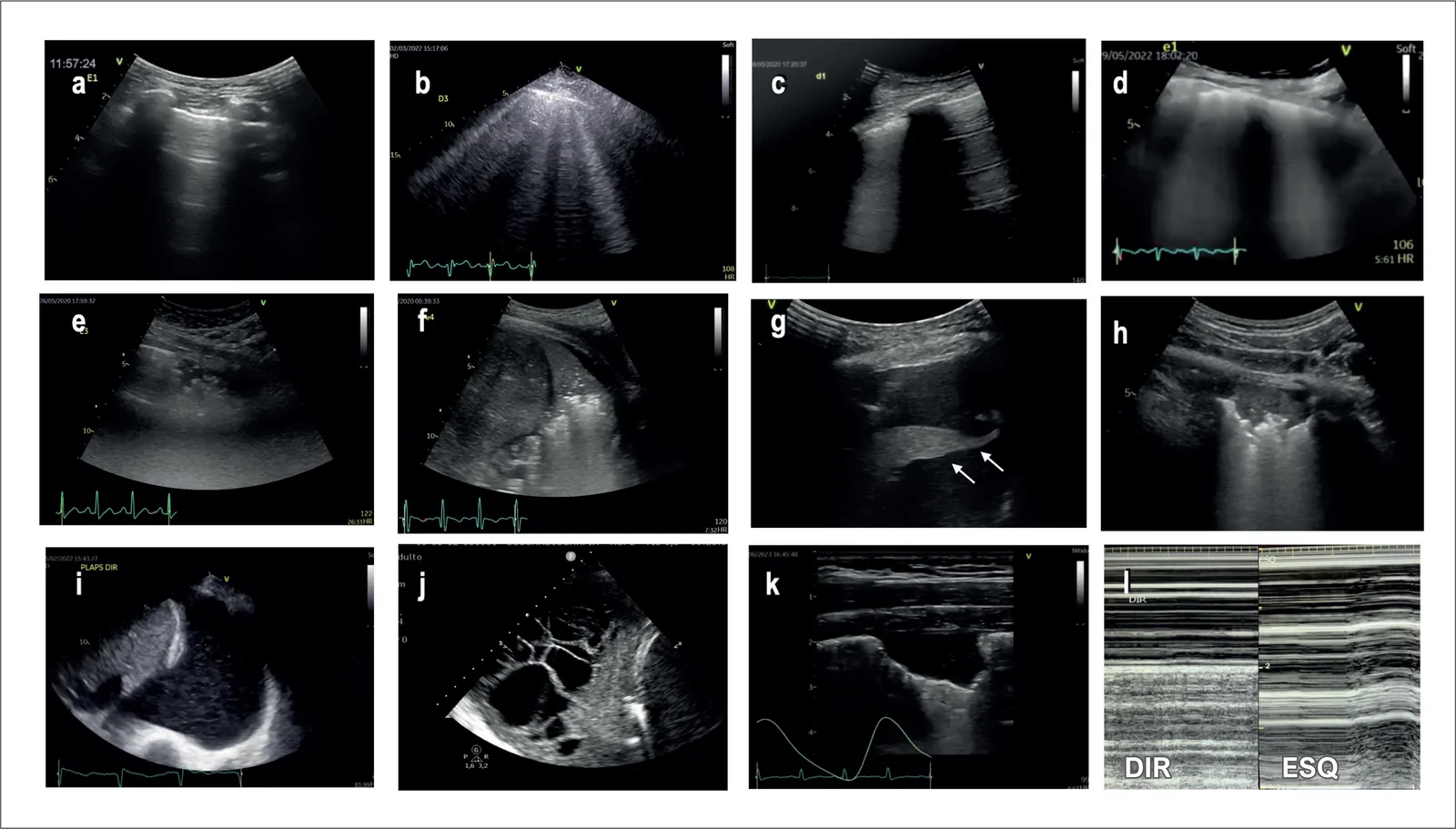
Achados à ultrassonografia pulmonar, descrição detalhada na Tabela 4. a. linhas A, b. linhas B, c. linhas B coalescentes com zonas poupadas, d. linhas B coalescentes, e. consolidação subpleural, f. consolidação translobar, g. atelectasia compressiva, h. sinal do retalho, i. sinal do plâncton, j. derrame septado, k. consolidação subpleural triangular com sonda linear (tromboembolismo pulmonar), l. código de barras (pneumotórax em pulmão esquerdo). DIR: direita; ESQ: esquerda.

Embora seja muito valorizado no POCUS, o protocolo BLUE tem caráter qualitativo e se limita à região anterior do tórax. Mais recentemente, com o objetivo de permitir uma abordagem mais quantitativa e o acompanhamento longitudinal, principalmente para pacientes com IC, seja ela com fração de ejeção reduzida (ICFEr) ou preservada (ICFEp), recomenda-se o protocolo com 8 quadrantes (Figura 12b)^70^ sem prejuízo no tempo ou na acurácia do exame.^71^ Nas patologias que acometem a região posterior do tórax, como as síndromes intersticiais, incluindo a pneumonia por SARS-CoV-2, podem ser utilizados protocolos que incluem a região posterior (Figura 12c).^72^

### 3.4. Quantificação de Linhas B

Não há um consenso em relação à melhor metodologia para a quantificação das linhas B. A tendência atual é a contagem automática e em tempo real do número de linhas B pelo uso de software específico.^73,74^

Na maioria dos casos, duas abordagens são utilizadas para quantificar as linhas B, com reprodutibilidade satisfatória:

- Escore de positividade por segmento, no qual a presença de três ou mais linhas B caracteriza uma zona positiva;^70^
- Contagem do número total de linhas B, individualmente ou pelo percentual da faixa que ocupam na tela, dividido por 10. O método é bastante útil no caso de linhas B coalescentes (por exemplo, se 80% da tela é ocupada por linhas B, considera-se a presença de 8 linhas B) (Figura 13b);^71^
- Embora ainda sem validação na literatura, para reduzir a variabilidade intra e interobservador e não prejudicar o tempo de exame, recomenda-se como terceira alternativa, no acompanhamento longitudinal do paciente, o uso de faixas de número de linhas B (por exemplo: 0, <3, 3-5, 5-7 e ≥8 linhas B). Atribui-se a cada faixa um escore de 0-4 para cada quadrante estudado.^54^ Ao final, soma-se o escore de todos os quadrantes para obter a pontuação total (por exemplo: escore 20 de um máximo de 32 em um protocolo com 8 quadrantes). Dessa forma, obtém-se uma análise semiquantitativa das linhas B que pode ser utilizada para fins de comparação durante o processo de descongestão do paciente.

### 3.5. Principais Aplicações da Ultrassonografia Pulmonar

Para facilitar a compreensão ao longo do texto, a Tabela 4 apresenta os termos mais utilizados na USP. A Figura 13 apresenta exemplos.

**Tabela 4:** Descrição dos achados mais frequentes à ultrassonografia pulmonar e seu significado. As letras em negrito se correlacionam com a Figura 13

Achado	Descrição	Significado
Linha pleural	Linha curvilínea hiperecoica abaixo das costelas.	Interface entre as pleuras parietal e visceral.
Deslizamento pleural	Deslizamento da pleural visceral sobre a parietal com a respiração.	Exclui pneumotórax e aderência pleural.
Linhas A **(a)**	Artefatos horizontais de reverberação a partir da linha pleural.	Pulmão aerado (normal) ou DPOC ou pneumotórax (com perda do deslizamento pleural).
Linhas B **(b)**	Artefatos verticais a partir da linha pleural que se estendem ao longo de todo o campo ultrassônico.	Acúmulo de líquido hidrostático e/ou inflamatório ou fibrose nos septos interlobulares e alvéolos.
Linhas Z	Artefatos verticais estáticos que não se estendem até o final do campo.	Sem significado patológico. Devem ser diferenciadas das linhas B.
Ponto pulmonar	Ponto de transição entre o deslizamento pleural e a perda de seu movimento.	Patognomônico de pneumotórax. Quanto mais lateral o ponto pulmonar, maior o pneumotórax.
Pulso pulmonar	Movimento rítmico da pleura sincronizado com os batimentos cardíacos.	Exclui pneumotórax na ausência de deslizamento pleural.
Código de barras (sinal da estratosfera)	Linhas horizontais em todo o campo ultrassônico ao modo M.	Indicativo de pneumotórax.
Sinal do plâncton	Ecos puntiformes móveis no interior do líquido.	Podem indicar infecção, hemotórax ou estar presente após terapia agressiva com diuréticos.
Derrame septado (loculado)	*Debris*, fibrinas e septações.	Derrame complexo, sugestivo de infecção, abcesso, empiema ou fibrose, dificultando a drenagem pleural.
Broncograma aéreo **(f)**	Ecos puntiformes hiperecogênicos represados em brônquios e circundados por consolidação.	Quando móveis (dinâmicos), sugerem consolidação pneumônica; quando estáticos, sugerem atelectasia.
Sinal do retalho **(h)**	Linha hiperecoica, irregular, subpleural e profunda que divide a zona consolidada da zona parcialmente aerada.	Presente nas consolidações não translobares mais frequentemente associadas a pneumonia.
Sinal da cortina	Sobreposição de pulmão aerado durante a respiração, apagando a imagem do fígado ou baço.	Bases pulmonares areadas no ponto de análise.
Sinal da medusa **(g)**	Segmento de pulmão colapsado com movimento ondulatório em meio a derrame pleural volumoso.	Atelectasia compressiva.
Consolidação subpleural **(e) (k)**	Pequena área hipoecoica subpleural (triangular, circular ou poligonal).	Embolia pulmonar, atelectasia, infecção, hemorragia ou neoplasia.
Consolidação translobar **(f)**	Ausência de ar no pulmão, que apresenta textura de órgão sólido (hepatização).	Pneumonia ou atelectasia.

DPOC: doença pulmonar obstrutiva crônica.

#### 3.5.1. Congestão Pulmonar

A congestão pulmonar de origem cardiogênica só pode considerada quando associada ao quadro clínico de ICFEp ou ICFEr em forma aguda ou crônica. Nessa última, a congestão pode ser subclínica, dada a possibilidade de mecanismos adaptativos como o espessamento da membrana alvéolo-capilar, o aumento da drenagem linfática e a hipertensão pulmonar.^75^ Na IC descompensada, os sinais de congestão pulmonar à USP costumam anteceder os sinais clínicos em dias, como aparecimento de estertores, terceira bulha, edema periférico, turgência jugular e dispneia.^75^ A Diretriz Brasileira de Insuficiência Cardíaca (2018) recomenda como classe I e nível de evidência C, o uso integrado da USP e da ecocardiografia para a avaliação da congestão pulmonar em pacientes admitidos com IC aguda. Esse procedimento acrescenta poucos minutos ao tempo de exame, com ganho substancial de informações.^76^

Pela sua simplicidade e nível de validação, recomenda-se utilizar o protocolo BLUE no serviço de emergência.^66^ A identificação do perfil B indica a presença de líquido extravascular. Quando agregado ao quadro de IC (ICFEr ou ICFEp) e com evidências ecocardiográficas de congestão hemodinâmica, confirma o diagnóstico de congestão pulmonar cardiogênica. Quando o objetivo é graduar e acompanhar evolutivamente a congestão, recomenda-se o protocolo de 8 quadrantes.^71^ No terceiro quadrante (ponto PLAPS), verifica-se a presença de derrame pleural e eventual consolidação pneumônica associada como possível causa da descompensação.

#### 3.5.2. Derrame Pleural

A USP apresenta alta acurácia para detecção de derrame pleural (93%) em comparação à radiografia de tórax (47%),^67^ possibilitando o diagnóstico de pequenos derrames a partir de 20 mL. A técnica ideal consiste em posicionar o paciente em decúbito semilateral, discretamente elevado (45°) ou sentado em caso de punção ou drenagem, com a insonação no ponto PLAPS.

O derrame pleural está presente em 56-90% dos casos de IC descompensada e em cerca de 25% dos casos de IC direita isolada e pode persistir em até 60% dos casos na fase pré-alta^63^ sem que isso implique pior prognóstico.^77^ Na IC esquerda, o derrame pleural decorre do aumento das pressões no átrio esquerdo e somente após o aparecimento da congestão pulmonar. Na IC direita, decorre da transferência do aumento da pressão atrial direita para o ducto torácico, dificultando a drenagem linfática do espaço pleural para a veia cava superior (VCS),^63^ sendo mais prevalente a direita quando unilateral. Diversos métodos de quantificação do derrame pleural foram descritos, sendo o mais utilizado aquele proposto por Balik et al.,^78^ no qual, durante a expiração, a maior distância do derrame entre a cúpula pleural e o tecido pulmonar em centímetros é multiplicada por 200, resultando em uma estimativa do volume do derrame em mililitros (Figura 14). Com base na ecogenicidade do líquido, a USP permite uma análise qualitativa da probabilidade de ser transudato, exsudato (sinal do plâncton, Figura 13i) ou hemorrágico. A presença de *debris* e septações indica derrame complexo de origem infecciosa, devendo levar à continuidade da investigação^79^ (Figura 13j).

**Figura 14:**
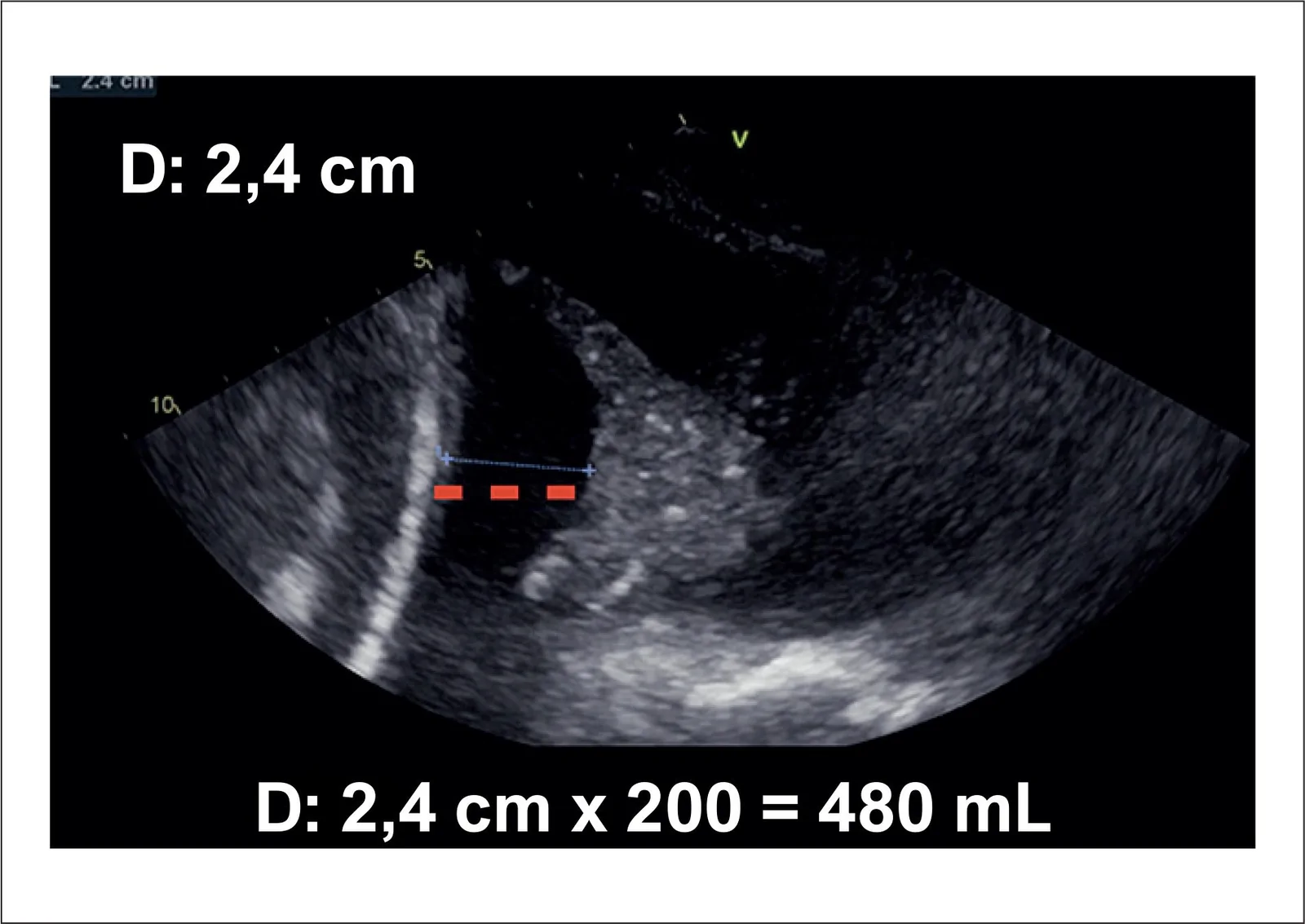
Exemplo de estimativa do volume de derrame pleural obtido pela estimativa da distância entre a cúpula diafragmática e o tecido pulmonar na expiração.

#### 3.5.3. Síndromes Intersticiais

A síndrome do desconforto respiratório agudo (SDRA) é uma forma de edema pulmonar de origem inflamatória, secundária a diversas doenças pulmonares ou sistêmicas. As causas mais frequentes são pneumonia viral ou bacteriana, sepse, broncoaspiração e trauma grave, incluindo barotrauma secundário a ventilação mecânica. A SDRA pode coexistir com IC, o que dificulta ainda mais a análise da USP.^80^ Alguns achados ultrassonográficos diferenciam o edema cardiogênico do inflamatório: distribuição heterogênea e assimétrica das linhas B, áreas poupadas de linhas B no mesmo campo ultrassônico (Figura 13c), espessamento e irregularidade da linha pleural e consolidações subpleurais (Figura 13e) ou translobares, todos presentes, por exemplo, na pneumonia associada à infecção por SARS-CoV-2.^72^

A fibrose pulmonar idiopática ou secundária a toxinas, poeiras orgânicas, medicamentos ou doenças do colágeno se caracteriza pelo espessamento e fibrose progressiva do tecido pulmonar. Nas formas mais brandas, observa-se espessamento da linha pleural. Com a evolução da doença, verifica-se aumento progressivo do espessamento pleural, redução do deslizamento pleural e surgimento de linhas B (algumas coalescentes) e cistos subpleurais que, à USP, se assemelham a consolidações subpleurais.^81^

#### 3.5.4. Consolidação Pneumônica

A pneumonia bacteriana é caracterizada pela perda da aeração do tecido pulmonar, que é preenchido por fluido infeccioso e desenvolve um aspecto de estrutura sólida (hepatização). A consolidação pode variar em extensão, sendo caracterizada como translobar quando se estende por todo campo ultrassônico e não translobar, ou mesmo subpleural, quando observa-se uma linha hiperecoica e irregular que divide o segmento consolidado do parcialmente aerado^82^ (sinal do retalho) (Figura 13h).

As consolidações localizam-se preferencialmente nas bases pulmonares (ponto PLAPS) com pequeno derrame pleural restrito ao seio costofrênico, linhas B de origem inflamatória e eventuais broncogramas dinâmicos (Figura 13f). A pleura se apresenta espessada, irregular e com perda do deslizamento.^83^

#### 3.5.5. Atelectasia

A atelectasia é a perda de volume e de aeração pulmonar por obstrução brônquica por tampão mucoso ou por compressão do parênquima por derrames pleurais volumosos. Atelectasias extensas causam distúrbio de ventilação-perfusão, com *shunt* intrapulmonar, aumento da resistência pulmonar e hipoxemia. O diagnóstico diferencial entre atelectasia e consolidação pneumônica por USP é desafiador e deve considerar o quadro clínico. Achados como broncogramas aéreos estáticos e linhas B localizadas e não difusas^84^ ajudam na diferenciação. Nos casos de atelectasia compressiva do pulmão, observa-se uma lâmina de tecido pulmonar consolidado com movimentos sinusoides (sinal da medusa) em meio ao líquido pleural (Figura 13g). Vale ressaltar que a atelectasia e a consolidação pneumônica podem coexistir em um mesmo paciente.^85^

#### 3.5.6. Pneumotórax

A USP apresenta sensibilidade de 90% e especificidade de 98% para o diagnóstico de pneumotórax.^65^ O acúmulo de ar entre a pleura parietal e a visceral ocorre principalmente no tórax anterior. Dessa forma, em caso de suspeita clínica de pneumotórax (por trauma ou por ventilação mecânica com pressão positiva elevada), recomenda-se iniciar a avaliação pela face anterior do tórax.

Nesta região, o achado de maior sensibilidade diagnóstica é a ausência de deslizamento pleural (lung sliding). À ultrassonografia em Modo M, esta condição manifesta-se como o sinal do código de barras (ou sinal da estratosfera), caracterizado por múltiplas linhas horizontais paralelas que percorrem toda a profundidade da imagem. Tal padrão contrasta com o sinal da beira-mar fisiológico, no qual a granulosidade do parênquima pulmonar (areia) se distingue das linhas horizontais estáticas da parede torácica (mar) (até a linha pleural)^65^ (Figura 13l).

A ausência do deslizamento pleural, contudo, não é exclusiva de pneumotórax, e também é evidenciada na entubação seletiva, na pleurodese, em contusões pulmonares e na fibrose pulmonar.^67^

Embora possa ser difícil de demonstrar, o ponto pulmonar é o único achado específico para pneumotórax. Ele indica o local exato da transição entre pulmão colapsado (deslizamento abolido) e pulmão normal (deslizamento presente). Quanto mais lateral for sua localização no tórax, maior o pneumotórax.

#### 3.5.7. Tromboembolismo Pulmonar

Apesar de não ser considerada método de escolha para a investigação de TEP,^34^ a USP é uma boa alternativa quando a angiotomografia pulmonar é contraindicada ou está indisponível. A análise integrada da USP com o POCUS cardíaco e venoso, em busca de sinais de sobrecarga de cavidades direitas e/ou trombose venosa profunda, pode aumentar a sensibilidade e a especificidade do método.^86^ Mais frequente nas regiões posterobasais do tórax, onde o paciente costuma referir dor pleurítica, o achado sugestivo à USP é a consolidação subpleural triangular (Figura 13k), que pode também ser circular ou poligonal. Essas consolidações se devem a necrose do parênquima ou atelectasia devido à perda de integridade do surfactante com extravasamento sanguíneo local.^87^

#### 3.6. Conclusão e Perspectivas Futuras

A USP antes subutilizada, tornou-se recentemente uma ferramenta essencial para o acompanhamento ambulatorial e para o manejo de pacientes com dispneia em unidades de terapia intensiva e serviços de emergência. A US é um método de imagem rápido, simples e altamente informativo, capaz de fornecer informações diagnósticas cruciais. Além disso, é custo-efetiva e adequada para aplicação à beira do leito.

Com o advento da IA para a interpretação de imagens, a contagem automática de linhas B e o desenvolvimento de algoritmos integrados, sua utilização ganhará mais espaço na medicina de urgência, terapia intensiva, cardiologia e outras especialidades em que o modelo agrega valor à tomada de decisão. Contudo, é preciso mencionar que a USP não substitui uma avaliação clínica criteriosa e deve sempre ser interpretada à luz do histórico do paciente.

## 4. Ultrassom Focado na Avaliação da Congestão Venosa Sistêmica – VExUS

### 4.1. Introdução

Historicamente, o tratamento de pacientes críticos se concentra em assegurar uma pressão de perfusão adequada, com forte foco no lado arterial do sistema vascular e na resposta do organismo à fluidoterapia. No entanto, o sistema venoso, muitas vezes negligenciado, desempenha um papel crucial. Os pacientes com insuficiência ventricular direita, hipertensão pulmonar ou sobrecarga hídrica são propensos a desenvolver de congestão venosa sistêmica, o que leva a redução do gradiente arteriovenoso, elevação da pressão hidrostática capilar e edema intersticial, muitas vezes exacerbado por disfunção endotelial.^88,89^ Esses fatores causam impactos adversos na perfusão tecidual e levam à disfunção orgânica, especialmente à renal. A lesão renal, por sua vez, acentua a retenção hídrica, perpetuando um ciclo vicioso e causando um forte aumento da morbimortalidade.^90–93^

Assim, a detecção precoce da congestão venosa é de extrema importância no desenvolvimento de estratégias adequadas para o acompanhamento clínico do paciente. Contudo, o exame físico e o balanço hídrico acumulado apresentam limitações importantes.^94–96^ Ademais, medidas da pressão venosa central (PVC), além de invasivas, são propensas a erros e nem sempre refletem diretamente a pré-carga, como em pacientes sob ventilação mecânica.^97–101^

Dada a escassez de sinais confiáveis, informações adicionais mais precisas e objetivas são necessárias para orientar as decisões terapêuticas. Nesse contexto, o POCUS tem se mostrado uma ferramenta útil e eficaz para avaliar o estado volêmico e quantificar a congestão venosa.

### 4.2. Estudos Clínicos e Evidências Científicas

Os marcadores ultrassonográficos de congestão venosa sistêmica não são novos. A pulsatilidade da veia porta foi descrita no contexto de IC há mais de 20 anos,^102^ embora nunca tenha sido totalmente incorporada à prática clínica.

Recentemente, o VExUS (*venous excess ultrasound*) tem demonstrado ser um método confiável e reprodutível, com boa concordância entre observadores treinados e correlação com outras medidas do estado volêmico, como a PVC. A técnica tem despertado interesse crescente na literatura, e alguns estudos exploram a sua aplicação em diferentes contextos clínicos (Tabela 5).

**Tabela 5:** Estudos que avaliaram o escore VExUS em diferentes contextos clínicos

Estudo	Objetivo	Resultado
**Beaubien-Souligny et al. (2020)**^103^ Estudo prospectivo unicêntrico; 145 pacientes submetidos a cirurgia cardíaca	Modelo inicial do sistema de graduação VExUS, analisando associação do desenvolvimento de LRA na população de cirurgia cardíaca	Associação com LRA subsequente: HR = 3,69; IC95% 1,65–8,24; p = 0,001; +LR: 6,37 (IC95% 2,19–18,50) quando detectado à admissão na UTI, superando as medidas de PVC
**Bhardwaj et al. (2020)**^104^ Estudo coorte prospectivo em UTI; 30 pacientes	Estudo prospectivo sobre aplicação do escore VExUS no estadiamento da LRA em pacientes com síndrome cardiorrenal	A resolução da LRA estava significativamente correlacionada com a melhora no grau VExUS (p = 0,003). Houve associação significativa entre alterações no grau VExUS e balanço hídrico (p = 0,006).
**Varudo et al. (2022)**^105^ Relato de caso de paciente em UTI com hiponatremia	Aplicação do escore VExUS em relato de caso como ferramenta rápida para auxiliar na avaliação do estado volêmico em paciente com hiponatremia complexa	VExUS geral grau 2, orientando estratégia diurética com melhora clínica
**Rolston et al. (2022)**^106^ Estudo observacional unicêntrico; 150 pacientes sépticos	Escore VExUS de pacientes sépticos no pronto-socorro antes de fluidoterapia (revisão de prontuários para determinar associação com piores desfechos)	Desfecho composto (mortalidade, admissão em UTI ou ativação de resposta rápida): VExUS grau 0: 31,6% dos pacientes VExUS grau 1: 47,6% dos pacientes VExUS grau > 1: 67,7% dos pacientes (p = 0,0015)
**Guinot et al. (2022)**^107^ Estudo observacional prospectivo em UTI; 81 pacientes que iniciaram terapia com diurético de alça	Avaliação de múltiplos escores (índice de pulsatilidade portal, índice de impedância venosa renal, VExUS) para predizer a depleção hídrica adequada induzida por diuréticos	O índice basal de pulsatilidade portal e o índice de impedância venosa renal foram considerados preditores superiores ao VExUS. O escore VExUS basal (AUC 0,66; IC95% 0,53–0,79; p = 0,012) não foi um bom preditor de resposta apropriada à depleção de líquidos induzida por diuréticos
**Menéndez-Suso et al. (2023)**^108^ Estudo piloto transversal em UTI pediátrica; 33 crianças	Associação do escore VExUS com PVC em UTI pediátrica	Forte correlação entre a gravidade do escore VExUS e a PVC (p<0,001) em crianças gravemente enfermas
**Longino et al. (2023)**^109^ Estudo prospectivo unicêntrico; 81 pacientes submetidos a cateterismo direito	Validação de associação do grau VExUS com a pressão atrial direita	VExUS teve área abaixo da curva favorável para predição de PAD > 10 mmHg (AUC 0,90; IC95% 0,83-0,97)
**Torres-Arrese et al. (2023)**^110^ Estudo prospectivo multicêntrico; 74 pacientes com insuficiência cardíaca aguda	Avaliação de diferentes parâmetros ultrassonográficos (incluindo o escore VExUS) como preditores de desfechos clínicos (morte ou reinternação relacionadas a insuficiência cardíaca)	VExUS grau 3 foi preditor de mortalidade durante a internação (AUC 0,885, Sn 80%, Sp 75%, VPP 33 % e VPN 96%)
**Magin et al. (2023)**^111^ Estudo piloto observacional prospectivo unicêntrico; 69 pacientes submetidos a cirurgia não cardíaca	Avaliação da obtenção de janelas VExUS no período perioperatório após cirurgias abdominais e avaliação da congestão venosa quanto à incidência de sobrecarga de volume	VExUS é viável e pode ser realizado para avaliar congestão venosa em cirurgias não cardíacas, incluindo cirurgias abdominais. Tendência de aumento da congestão venosa após cirurgia abdominal
**Viana-Rojas et al. (2023)**^112^ Estudo prospectivo; 77 pacientes com SCA	Avaliação da associação entre VExUS e LRA em pacientes com diagnóstico de SCA	VExUS ≥ 1 foi associado a LRA (OR: 6,15; IC95% 1,26 – 29,94; p = 0,02). VExUS ≥ 1 mais prevalente em IAM inferior quando comparado a IAM anterior ou SCASSST (48,3% vs. 25,8% e 22,5%, respectivamente; p = 0,031)

AUC: *area under the curve*; HR: *hazard ratio*; IAM: infarto agudo do miocárdio; IC95%: intervalo de confiança de 95%; +LR: positive likelihood ratio; LRA: lesão renal aguda; OR: odds ratio; PAD: pressão atrial direita; PVC: pressão venosa central; SCA: síndrome coronariana aguda; SCASSST: síndrome coronariana aguda sem supradesnível do segmento ST; Sn: sensibilidade; Sp: especificidade; UTI: unidade de terapia intensiva; VCI: veia cava inferior; VExUS: venous excess ultrasound; VPN: valor preditivo negativo; VPP: valor preditivo positivo.

### 4.3. Componentes do VExUS

Para a análise do VExUS, pode-se utilizar o transdutor setorial ou o convexo. É necessário reduzir o filtro e adequar a escala de velocidade para fluxos venosos. A sequência passo a passo a seguir é recomendada para cada um dos vasos estudados. Ela apresenta a melhor forma de obter as curvas de fluxo e mitigar possíveis erros de interpretação. A graduação final da congestão venosa sistêmica deve seguir o modelo proposto na Figura 15.

**Figura 15:**
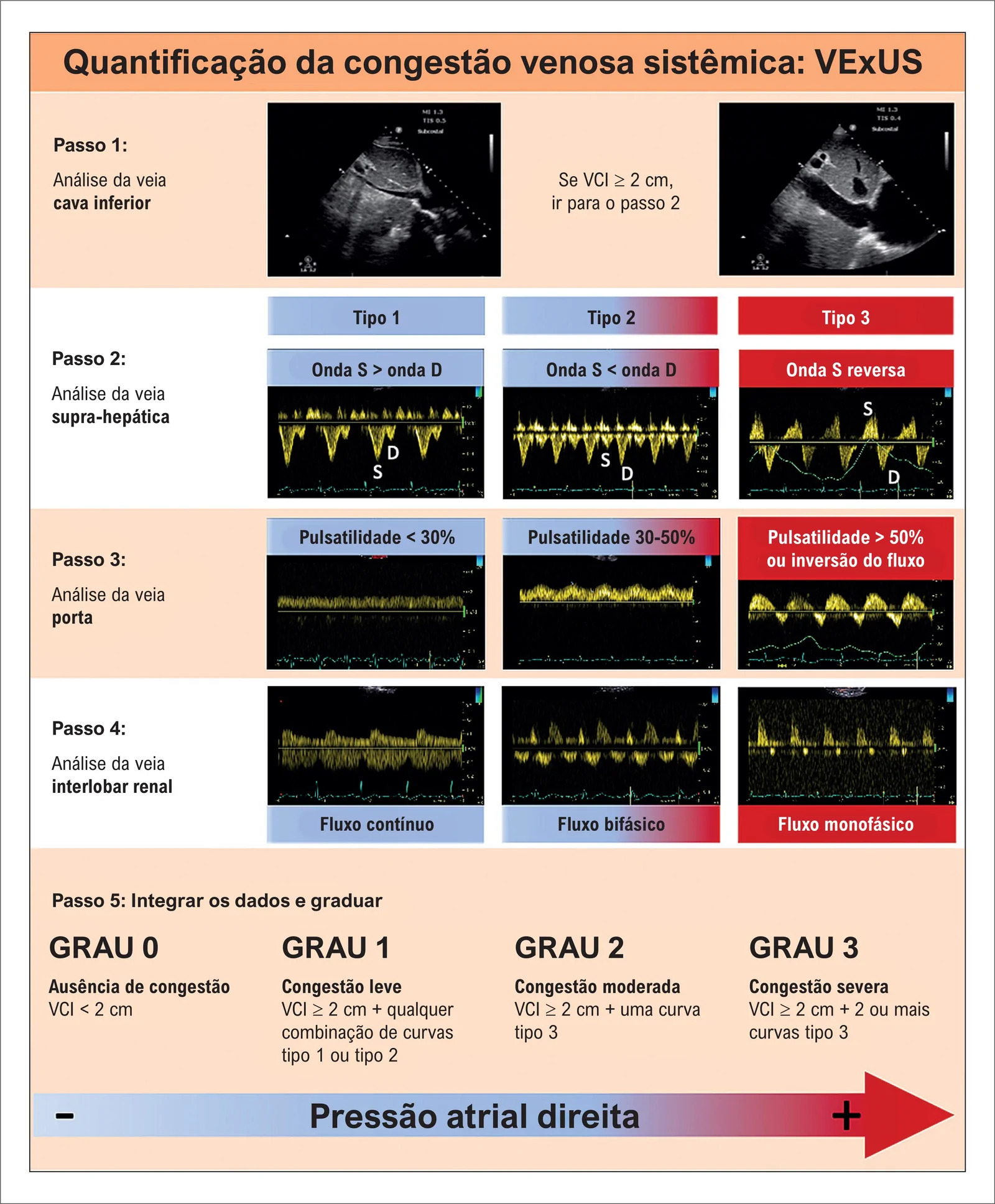
Graduação do VExUS: quando o diâmetro da VCI é maior do que 2 cm, três graus de congestão venosa são definidos com base na gravidade das alterações encontradas no fluxo das veias supra-hepática, porta e interlobar renal, que idealmente devem ser registrados em apneia expiratória. VCI: veia cava inferior; VExUS: venous excess ultrasound. Adaptado de Koratala et al.^121^

#### Passo 1: Análise da veia cava inferior

A análise da VCI é realizada preferencialmente pela janela subcostal, com o transdutor com índex direcionado para as 12 horas. Nessa projeção, a VCI deságua no átrio direito (AD) longitudinalmente. Uma alternativa menos ideal é realizar a análise da VCI pela linha axilar média, com o índex também direcionado às 12 horas (janela trans-hepática). Essa última janela superestima, com alguma frequência, as dimensões da VCI e, portanto, deve ser evitada.

A medição da VCI é feita 2 cm antes da sua desembocadura no AD, com o paciente em apneia expiratória. A veia supra-hepática esquerda geralmente deságua próximo ao local da medição, então é preciso tomar cuidado para não a incluir na medição.

Caso a VCI tenha menos de 2 cm, considera-se que não há congestão sistêmica, o VExUS é classificado em grau 0 (sem congestão) e não há necessidade de analisar os demais componentes (veia supra-hepática, veia porta e veia interlobar renal). Caso a VCI seja igual ou maior a 2 cm, pode haver evidência de congestão e deve-se prosseguir com a análise dos demais componentes. A utilização desse parâmetro ajuda a eliminar possíveis falso-positivos de congestão sistêmica.^103^

#### Passo 2: Análise da veia supra-hepática ao Doppler pulsado

A veia supra-hepática pode ser avaliada pela janela subcostal ou pela via trans-hepática (linha axilar média, com anteriorização do transdutor para melhor visualização da imagem, caso necessário). O Doppler colorido deve ser ajustado para velocidades de 30-40 cm/s. A veia analisada deve ser aquela mais alinhada verticalmente em relação ao transdutor para facilitar a obtenção dos fluxos.

A veia supra-hepática, por estar diretamente conectada a uma veia central (VCI), transmite toda a sua pulsatilidade por meio de quatro ondas ao Doppler pulsado:^113^ a **onda S**, de padrão negativo e dominante, ocorre pelo relaxamento atrial direito e repuxamento do anel tricúspide em direção ao ápice cardíaco durante a sístole ventricular; a **onda V**, nem sempre presente, em geral apresenta padrão positivo e ocorre quando o anel tricúspide volta à sua posição inicial diastólica; a **onda D**, de padrão negativo, menos ampla que a onda S, corresponde à abertura da valva tricúspide e ao influxo do sangue do AD para o ventrículo direito; e a **onda A**, de padrão positivo, resulta da contratilidade atrial.

A onda S ocorre imediatamente após a onda R do eletrocardiograma (ECG), a onda D ocorre após a onda T e a onda A tem correlação direta com a onda P do ECG, ausente em casos de fibrilação atrial.^114^ Dessa maneira, é de extrema importância utilizar o ECG para diferenciar os tipos de ondas venosas.

A morfologia das ondas pode ser dividida em três tipos, de acordo com a PAD:^115^

1. Normal (tipo 1): quando a onda S é maior que a onda D.
2. PAD aumentada (tipo 2): a onda S se torna menor que a onda D, já que o esvaziamento atrial ocorre predominantemente na diástole.^116^
3. PAD muito aumentada (tipo 3): ocorre inversão da onda S, que adquire um padrão positivo, enquanto a onda D mantém um padrão negativo.

#### Passo 3: Análise da veia porta ao Doppler pulsado

A análise da veia porta é realizada preferencialmente pela janela trans-hepática (linha axilar média), com o índex posicionado às 12 horas. A velocidade do Doppler colorido é ajustada para 20-30 cm/s. Pode também ser visualizada pela janela subcostal. Dessa maneira, a veia porta apresenta fluxo ascendente, cor vermelha ao Doppler colorido e padrão contínuo ou levemente pulsátil. A veia porta tem paredes mais espessas e hiperecogênicas (e, portanto, mais brilhosas), o que a torna fácil de diferenciar das veias supra-hepáticas, que não apresentam paredes visíveis, têm fluxo direcionado à VCI e, portanto, são visualizadas com coloração azulada.

Por estar isolada da circulação venosa sistêmica central pelos vasos sinusoidais hepáticos, seu fluxo é contínuo ou levemente pulsátil, de caráter positivo associado à contração atrial.^117^ À medida que a pressão venosa sistêmica aumenta, os vasos sinusoidais se dilatam pela congestão e a pulsatilidade venosa passa a ser transmitida de forma mais intensa para a veia porta. Assim, quanto maior a congestão sistêmica, maior é a dilatação sinusoidal e, portanto, maior a pulsatilidade, cuja variação é dada pela fração de pulsatilidade (fração de pulsatilidade = velocidade de pico – velocidade mínima / velocidade de pico × 100).

Assim como nas veias supra-hepáticas, a PAD modifica a morfologia das ondas da veia porta ao Doppler pulsado, que podem ser classificadas em três tipos:^103^

1. Normal (tipo 1): os vasos sinusoidais estão preservados, com oscilação do fluxo abaixo de 30%.
2. PAD aumentada (tipo 2): ocorre dilatação dos vasos sinusoidais, com oscilação do fluxo entre 31 e 50%.
3. PAD muito aumentada (tipo 3): há dilatação acentuada dos vasos sinusoidais, com importante transmissibilidade da pulsação venosa sistêmica para a veia porta, com oscilação acima de 50%. A velocidade pode estar até mesmo abaixo da linha de base (negativa).^118^

#### Passo 4: Análise da veia interlobar renal ao Doppler pulsado

A avaliação da veia interlobar renal é de extrema importância na graduação do VExUS, pois, de forma isolada, apresenta a maior correlação com a evolução para insuficiência renal em pacientes com congestão sistêmica acentuada.

Por ser um órgão encapsulado, o rim não transmite a pulsatilidade das veias centrais, então o fluxo venoso observado geralmente é contínuo. Porém, à medida que a congestão sistêmica aumenta (nefropatia congestiva), ocorre edema intersticial, a complacência venosa diminui e o padrão do fluxo se torna bifásico ou, em casos mais acentuados, monofásico.^119,120^

Pela proximidade entre artérias e veias, o fluxo arterial parenquimatoso é visualizado acima da linha de base, enquanto o fluxo venoso é visualizado abaixo dela.

É importante não confundir a veia renal, presente na região do hilo renal, com as veias intrarrenais, presentes na região cortical; a veia renal é semelhante a uma veia central e, portanto, tem fluxo pulsátil. Esta é uma causa frequente de erro.

Para a identificar corretamente a veia interlobar, diminua a velocidade do Doppler colorido para 20 cm/s de modo visualizar melhor o fluxo e, assim, registrá-lo na área cortical renal.

De acordo com a pressão venosa sistêmica, o fluxo das veias interlobares pode dividido entre três tipos:

1. Normal (tipo 1): fluxo venoso contínuo durante todo o ciclo cardíaco.
2. PAD aumentada (tipo 2): fluxo venoso bifásico, com 1 pico sistólico e 1 pico diastólico.
3. PAD muito aumentada (tipo 3): com o aumento da pressão venosa sistêmica e consequente edema renal, apenas o pico diastólico é observado, resultando em fluxo monofásico.

Para a obtenção adequada dos fluxos no protocolo VExUS, recomenda-se a realização de apneia respiratória sempre que possível.

#### Passo 5: Integração dos dados e graduação do VExUS

Uma vez realizada a análise passo a passo como descrito, os dados devem ser integrados para adequada graduação do grau de congestão venosa visceral.

### 4.4. Graduação do VExUS

A quantificação do grau de congestão venosa visceral se baseia no estudo de Beaubien-Souligny et al.,^103^ no qual foram validados cinco protótipos de um sistema de gradação, denominado escore VExUS, em uma população de 145 pacientes no pós-operatório de cirurgia cardíaca com relação ao risco de desenvolver insuficiência renal. Uma análise *post hoc* desse estudo prospectivo unicêntrico identificou o protótipo com melhor desempenho diagnóstico. Isso levou à proposta da classificação em quatro graus de congestão venosa, baseada nas alterações encontradas nos componentes do VExUS (Figura 15).

O estudo incluiu pacientes em ventilação mecânica, o que expandiu a aplicabilidade prática do escore VExUS. É importante ressaltar que, por si só, esse sistema de gradação não é capaz de diferenciar a congestão venosa visceral causada por sobrecarga volêmica da sobrecarga pressórica. Os achados devem ser interpretados de forma conjunta, considerando o contexto clínico e os demais achados do POCUS.^121^

### 4.5. Aplicações Clínicas

De modo geral, o escore VExUS não deve ser interpretado de forma isolada ou considerado um substituto de uma anamnese cuidadosa e de um exame físico convencional. Ainda assim, ele pode fornecer informações valiosas para o manejo do paciente crítico, sobretudo em patologias ou estados associados à elevação da pressão venosa, que aumentam os riscos de congestão venosa sistêmica substancial, má perfusão tecidual e consequente disfunção renal.

O VExUS desempenha um papel relevante no equilíbrio de fluidos. Embora não apresente dados a respeito da necessidade de reposição volêmica, é indicador fundamental de tolerância à fluidoterapia, identificando os casos em que a descontinuidade de ressuscitação volêmica ou a remoção de fluidos provavelmente será benéfica, especialmente em abordagens complexas associadas à lesão renal aguda (Figura 16).

**Figura 16:**
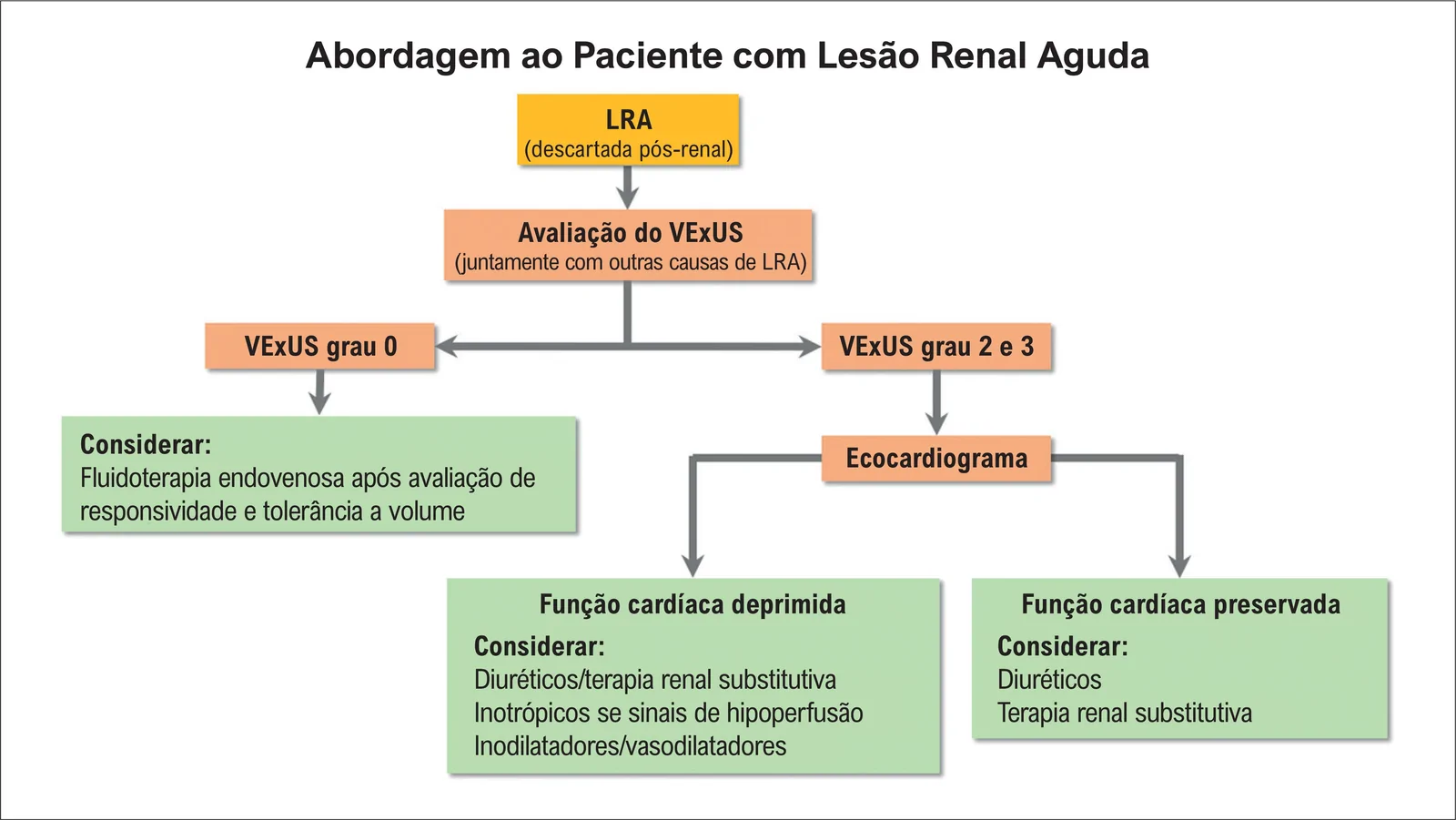
Algoritmo incorporando o POCUS ao manejo do paciente crítico. LRA: lesão renal aguda; VExUS: volume excess ultrasound. Adaptado de Bhardwaj et al.^104^ e Argaiz et al.^122^

Em diversos contextos clínicos, ensaios recentes têm demonstrado resultados promissores para o uso dessa ferramenta (Tabela 5): predição de risco de lesão renal aguda no perioperatório de cirurgias cardíacas; monitoramento de terapia descongestiva em pacientes com síndrome cardiorrenal; avaliação do estado volêmico em hiponatremia complexa; avaliação do risco de lesão renal aguda em síndromes coronarianas isquêmicas; estimativa de PVC em crianças gravemente enfermas; avaliação do estado volêmico em quadros de hipertensão pulmonar, IC direita ou pacientes sob ventilação mecânica (casos em que a PVC não reflete adequadamente a pré-carga).

### 4.6. Limitações e Armadilhas

A avaliação da congestão deve sempre iniciar com o estudo da VCI. No entanto, a avaliação isolada da VCI apresenta inúmeras limitações, a começar pela correlação modesta com a PAD aferida em estudos de cateterismo.^121^ Além disso, a avaliação de sua colapsibilidade depende da capacidade inspiratória do paciente e pode variar bastante com as condições clínicas. Atletas e indivíduos jovens podem apresentar uma VCI persistentemente dilatada sem aumento da PAD, enquanto pacientes com pressão intra-abdominal elevada podem ter a VCI colabada a despeito da PAD elevada. Além disso, a avaliação da VCI apenas no eixo longo está sujeita ao efeito cilindro, que ocorre quando um feixe de ultrassom 2D atravessa uma estrutura 3D na periferia e não no eixo central, o que pode levar à subestimação do calibre.

Na avaliação da veia supra-hepática, a ausência do traçado simultâneo de ECG pode levar a erros de interpretação importantes,^103^ especialmente nas arritmias. O ritmo de fibrilação atrial pode causar uma redução da onda S em pacientes com PAD normal, uma vez que a onda depende em parte do relaxamento atrial. Em pacientes com insuficiência tricúspide ou hipertensão arterial pulmonar, pode-se observar a presença crônica do padrão com onda S menor que onda D ou onda S reversa, independentemente do status volêmico. A disfunção do ventrículo direito, causada pela redução do rebaixamento do anel tricúspide durante a sístole ventricular, também pode reduzir ou até abolir a onda S.

A avaliação da veia porta também apresenta limitações.^121,123^ Indivíduos magros e com baixo índice de massa corporal podem apresentar pulsatilidade elevada, mesmo com PAD normal. Em pacientes com hepatopatia crônica, a curva espectral reflete alterações de pressão locais mais do que as pressões centrais, de modo que se observa fluxo pulsátil mesmo em pacientes euvolêmicos devido à presença de *shunts* porto-arteriais. Indivíduos com hipertensão portal podem apresentar fluxo reverso (hepatofugal), enquanto naqueles com cirrose hepática, a curva pode permanecer aparentemente normal, a despeito de insuficiência tricúspide (IT) e PAD elevada, devido ao aumento da resistência sinusoidal causado pela fibrose hepática.

Por fim, a principal limitação da avaliação da veia interlobar renal é a dificuldade técnica.^121^ Os vasos intrarrenais têm calibres pequenos e frequentemente saem do plano de insonação à medida que o rim se movimenta com a respiração. A realização da apneia ajuda, mas nem sempre é possível, pois depende das condições clínicas. Obesidade e dispneia podem impedir a obtenção do traçado ao Doppler. Além disso, fatores que não a PAD, como anormalidades renais estruturais e doença renal crônica, podem alterar a curva de fluxo.^123^

### 4.7. Conclusão

O escore VExUS tem demonstrado alta especificidade para o diagnóstico de congestão venosa sistêmica e valor prognóstico para lesão renal aguda após cirurgia cardíaca. É um método de fácil execução por profissionais treinados e pode ser útil no manejo clínico de pacientes críticos.

## 5. POCUS na Trombose Venosa Profunda

### 5.1. Introdução

A trombose venosa profunda (TVP) é uma patologia vascular comum, podendo ocorrer em cerca de 30% dos pacientes hospitalizados.^124–126^ O TEP é uma complicação da TVP e representa a terceira causa mais frequente de síndrome cardiovascular aguda, atrás apenas do IAM e do acidente vascular encefálico. No Brasil, entre 2016 e 2020, ocorreram mais de 34.000 óbitos por tromboembolismo venoso.^127^ Na ausência de tratamento adequado, a mortalidade por TEP pode atingir 30%, sendo um dos achados insuspeitos de autópsias mais frequentes em pacientes críticos.^128–130^ O diagnóstico rápido e acurado é fundamental para o tratamento oportuno da TVP e a prevenção de complicações. A Diretriz Conjunta sobre Tromboembolismo Venoso, publicada em 2022, foi uma força-tarefa de sociedades médicas brasileiras (DIC/SBC, CBR, SBACV e SBM) com o intuito de orientar o diagnóstico de TVP e TEP.^131^

Na TVP ocorre a formação de trombos em áreas de fluxo venoso mais lento, com subsequente propagação ao longo do lúmen do vaso. Na fase aguda ocorre uma resposta inflamatória da parede venosa, com preenchimento total ou parcial do lúmen, acompanhado de sintomas clínicos associados, como dor, edema e rubor do membro afetado. Porém, em cerca de 50% dos casos, os sinais clínicos de TVP são inespecíficos, o que dificulta o diagnóstico. Torna-se necessário a realização de exames complementares para confirmar ou excluir o diagnóstico de TVP. Em caso de suspeita de TVP, a Diretriz Brasileira de 2022 orienta a utilização do escore de Wells simplificado como modelo de predição clínica, associado à dosagem do D-dímero e à realização da ultrassonografia vascular (USV).^131–133^

A USV é o exame de escolha na suspeita de TVP, pois fornece informações anatômicas e funcionais do sistema venoso, com as vantagens de não ser invasivo, não usar contraste nefrotóxico, ser reprodutível e ter baixo custo.^134,135^

Este capítulo apresenta os diversos protocolos de POCUS para a investigação de TVP. Com base nas orientações da Diretriz Brasileira de 2022, recomenda-se a utilização do protocolo de compressão estendido, também chamado de protocolo de compressão em três pontos (ultrassonografia de compressão [USC]).^131^ Essa orientação é corroborada pelo último Consenso Europeu da Sociedade de Medicina Intensiva e pela última Diretriz de Ultrassonografia em Emergência do Colégio Americano de Emergencistas, sendo incluída também nos critérios de certificação em ultrassonografia em medicina intensiva do CHEST (*Critical Care Ultrasonography Certificate of Completion Certificate of Completion Program - American College of Chest Physicians [chestnet.org*]).^7,136^

### 5.2. Evidências Científicas do POCUS na Pesquisa de Trombose Venosa Profunda

Com relação à acurácia da USC à beira do leito realizada por médicos emergencistas, uma meta-análise de Pomero et al.^137^ revelou sensibilidade de 96,1% (IC95%: 90,6–98,5%), e especificidade de 96,8% (IC95% 94,6–98,1%). Entretanto, os estudos incluídos apresentaram grande heterogeneidade, provavelmente devido à variação no treinamento de alguns médicos, à presença de tromboses distais ou intra-abdominais e à inclusão de pacientes com biotipos difíceis.^139^

No estudo de Garcia et al.,^139^ a acurácia da USC no diagnóstico de TVP em pacientes com suspeita clínica elevada realizada na emergência foi de 91,7% em comparação com o exame completo realizado por especialistas em até 48 horas. Com relação à investigação de TVP realizada por médicos intensivistas com menos de 2 anos de experiência de US, o estudo de Kory et al.^140^ encontrou acurácia de 95%. Além disso, os autores relataram que o diagnóstico foi estabelecido em média 13 horas antes daquele obtido por médicos especialistas. Um estudo multicêntrico que avaliou a acurácia da USC realizada por médicos emergencistas em pacientes com alta suspeita de TEP/TVP mostrou um valor preditivo negativo elevado (100%) e um valor preditivo positivo moderado (61,5%), com sensibilidade de 100% e especificidade de 95,8%, em comparação com o exame formal realizado por especialistas. Observou-se ainda uma redução de cerca de 50% no tempo entre o POCUS e a US formal.^141^

Esses estudos demonstram, em diversos cenários, a utilidade da USC no manejo de pacientes com suspeita de TVP em serviços de e em unidades de terapia intensiva. Além disso, a rapidez com a qual esses exames são realizados, em menos de 5 minutos, pode evitar ou reduzir exames completos desnecessários, atrasos diagnósticos (especialmente em exames fora do horário comercial) e a indisponibilidade de profissionais especializados. Na comparação entre a USC de 2 e de 3 pontos em relação à acurácia, em uma meta-análise recente que incluiu 17 estudos, ambos os protocolos apresentaram ótimo desempenho, com sensibilidades e especificidades de cerca de 90% e taxas de falsos positivos e negativos de cerca de 4%. A acurácia foi maior quando os exames eram realizados pelos médicos emergencistas em comparação com residentes.^142^

Por ser uma manifestação menos frequente, as publicações que analisaram o POCUS para a investigação de trombose em membros superiores nos serviços de emergência são muito escassas e utilizam protocolos menos definidos.

### 5.3. Técnica do Protocolo de Ultrassonografia Focado para Pesquisa de Trombose Venosa Profunda

A aplicação de protocolos mais concisos à beira do leito, em contraste com o protocolo completo, apresenta vantagens em serviços de urgência e emergência. A rapidez no diagnóstico possibilita o início precoce do tratamento, o monitoramento contínuo ajusta terapias conforme necessário e a redução do tempo de internação resulta em recuperação mais rápida, o que beneficia os pacientes e os serviços de saúde.

No entanto, é preciso observar que, embora o POCUS proporcione benefícios substanciais, sua aplicação deve ser integrada ao contexto clínico de forma adequada, levando em consideração as necessidades específicas de cada paciente e assegurando a confiabilidade dos resultados.

Existem diversos tipos de equipamentos de ultrassonografia, desde dispositivos portáteis até máquinas mais complexas e sofisticadas, com diferentes níveis de custo. O Quadro 2 resume as principais orientações técnicas para a realização do protocolo POCUS na investigação de TVP.^143^

**Quadro 2:** Sequência de orientações técnicas para a realização do protocolo POCUS na investigação de TVP

Orientações para o protocolo de exame POCUS na investigação de TVP
•Conheça previamente o equipamento disponível no setor.
•Escolha o transdutor: (1) linear entre 5-10 MHz; (2) convexo entre 2-5 MHz (pode ser necessário em pacientes obesos ou edemaciados)
•Escolha o preset para exames venosos, quando disponível.
•Aprenda o ajuste básico da imagem bidimensional, como os controles de ganho, profundidade e foco.
•Observe o índex do transdutor, posicionando-o sempre relacionado ao marcador da tela do equipamento.
•Tenha gel de acoplamento à mão.
•Registre as imagens para comparações evolutivas.

O paciente deve ser posicionado, preferencialmente, em decúbito no próprio leito, com a cabeceira elevada a 30° se possível. A posição mais adequada para o exame do membro inferior é a rotação externa com leve flexão do joelho, o que possibilita a avaliação da região inguinal e da fossa poplítea sem a necessidade de reposicionar o membro.^143^

Tanto no protocolo de USC de dois quanto no de três pontos, deve-se avaliar, no modo de escala de cinzas, a presença de material ecogênico intraluminal indicativo de trombo. É fundamental realizar a técnica de compressão com o transdutor posicionado de transversalmente sobre a veia.^143,144^ A Figura 17 apresenta esquematicamente os protocolos de USC.

**Figura 17:**
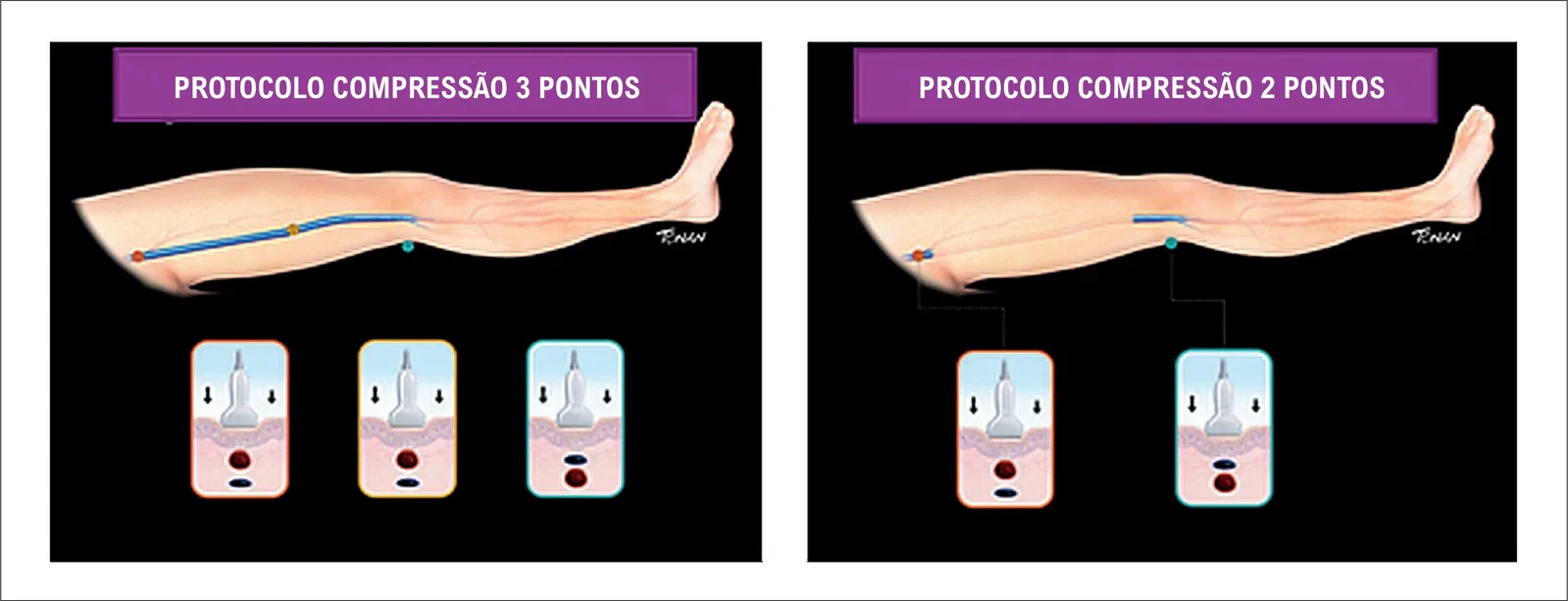
A: protocolo de exame de ultrassonografia de compressão estendido (três pontos); B: protocolo de exame de ultrassonografia de compressão (dois pontos).

O protocolo de dois pontos consiste na avaliação da veia femoral comum e da veia poplítea. O exame inicia na região inguinal, abrangendo o segmento localizado de 1 a 2 cm acima e abaixo da junção safenofemoral. Em seguida, realiza-se a avaliação na fossa poplítea, onde a veia corre paralela à artéria poplítea, até a confluência das veias da perna. O protocolo de três pontos avalia as veias femoral comum e poplítea da mesma maneira que no protocolo de dois pontos, mas incorpora a compressão da veia femoral nos segmentos proximal e médio-distal da coxa.

Independentemente do protocolo empregado, no âmbito do POCUS, o diagnóstico da TVP é feito pela visualização do trombo (material ecogênico intraluminal) ou pela identificação de uma área de incompressibilidade na parede da veia. Na presença de trombose aguda, é comum que a incompressibilidade do vaso ocorra antes que o trombo esteja visível. Portanto, a fim de evitar resultados falsos negativos, é crucial que a pressão do transdutor para colabar a parede venosa não seja excessivamente suave.^143^ A Figura 18 apresenta a sequência dos cortes ultrassonográficos no protocolo de compressão de três pontos (estendido).

**Figura 18:**
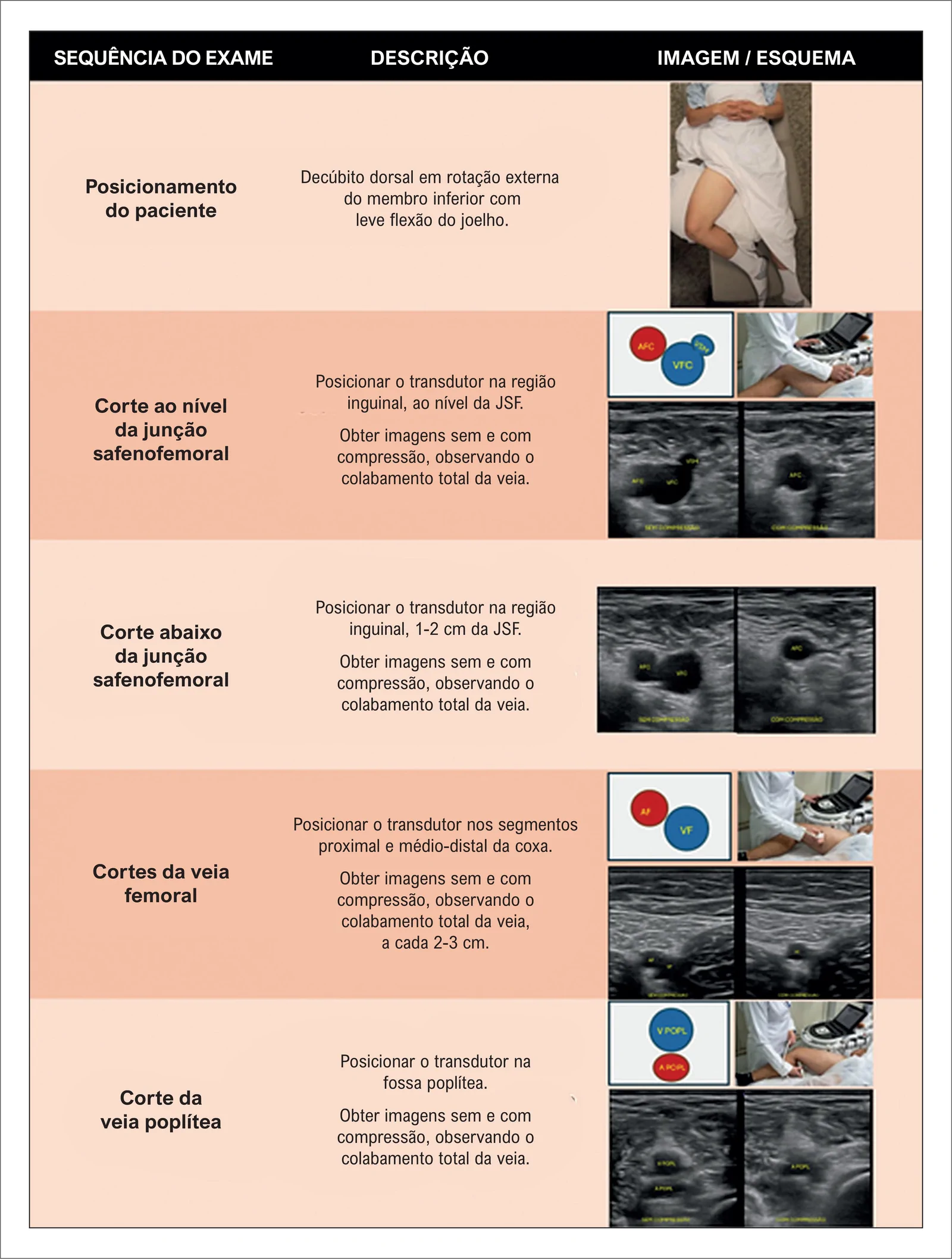
Sequência de cortes para o protocolo de ultrassonografia de compressão de três pontos (estendido).

A Figura 19 exemplifica os achados ultrassonográficos em veias normais e em veias com sinais de trombose aguda, em relação ao calibre venoso, à compressibilidade venosa, às características do lúmen venoso e à ecogenicidade do trombo.

**Figura 19:**
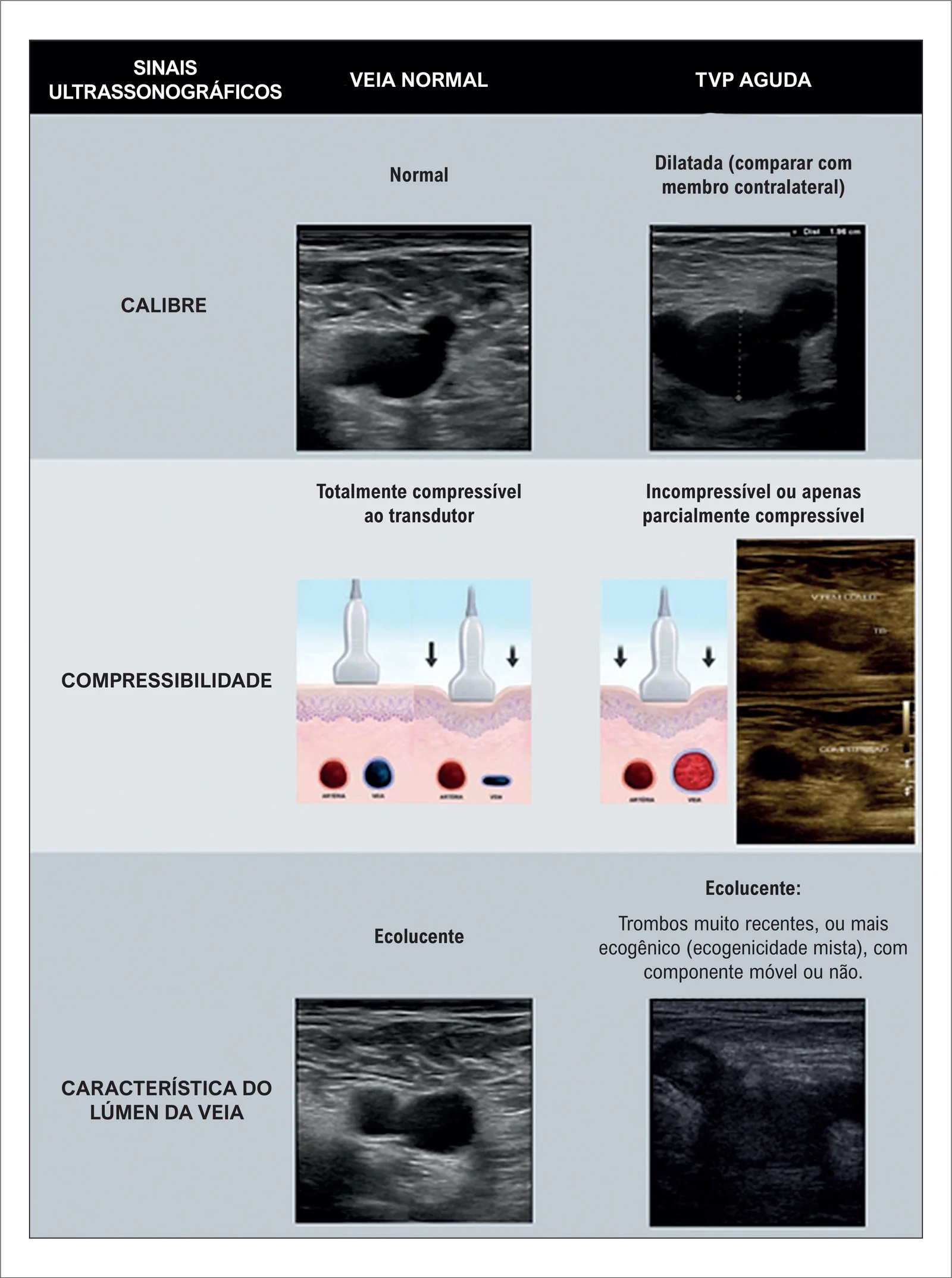
Achados ultrassonográficos nas veias normais e nas veias com sinais de trombose aguda.

Os sinais e sintomas clínicos associados ao evento trombótico podem ser comuns a diversas outras condições agudas ou crônicas. Portanto, é importante conhecer os diversos diagnósticos diferenciais, como cistos sinoviais (rotos ou não), insuficiência venosa crônica, roturas e hematomas musculares, abscessos, linfadenomegalias, tumores e cisto de Baker, entre outros.^131^

É essencial que os profissionais de saúde se familiarizem com os dispositivos e técnicas específicos empregados nos protocolos de POCUS que utilizam, garantindo a precisão e confiabilidade dos resultados obtidos no local de atendimento. Além disso, a colaboração interdisciplinar e a formação contínua representam elementos fundamentais para a implementação bem-sucedida desses protocolos de investigação da TVP.

Recomendação: O POCUS para a investigação de TVP proximal, realizado por profissionais devidamente treinados, em situações de urgência e emergência, possui Grau de recomendação I e Nível de evidência B.

## 6. POCUS no Paciente em Choque Circulatório

### 6.1. Introdução

O choque é um estado de insuficiência circulatória generalizada grave que leva à oferta inadequada de oxigênio aos tecidos. Os perfis hemodinâmicos básicos do choque podem ser divididos em: hipovolêmico, cardiogênico, obstrutivo e distributivo. Os perfis possuem tratamentos diversos e, portanto, exigem rapidez diagnóstica e terapêutica. O POCUS é o método de escolha na avaliação e monitorização hemodinâmica inicial do paciente em choque circulatório. A técnica permite o diagnóstico do perfil hemodinâmico do choque em até 2 minutos, mesmo quando realizado por não especialistas.^145^

O foco principal de quem ainda se encontra na fase inicial do treinamento deve ser a avaliação qualitativa das imagens. A tomada de decisões com base em medidas objetivas, sem proficiência suficiente para tal, pode comprometer o manejo diagnóstico e terapêutico do paciente.

Alguns autores propuseram protocolos para ultrassonografia à beira do leito em casos de parada cardíaca (protocolo FEER, PEA, CASA),^3^ choque (protocolos UHP, Trinity, RUSH, FATE, EGLS)^4,5^ ou insuficiência respiratória (protocolo BLUE).^6^

O objetivo deste capítulo é apresentar um algoritmo diagnóstico para o choque circulatório baseado no POCUS, semelhante ao protocolo EGLS (adaptado), que possa ser realizado à beira do leito por profissionais não especialistas em imagem, atuantes em serviços de emergência e/ou medicina intensiva.

### 6.2. Protocolos Diagnósticos

Este capítulo apresenta uma abordagem que integra os conceitos do protocolo BLUE, do POCUS cardíaco e dos protocolos EFAST^7^ e EGLS.^8^ Dada a relação estreita entre a água extravascular pulmonar, função ventricular esquerda e status volêmico em estados de choque, a combinação da ultrassonografia pulmonar e cardíaca com o exame EFAST oferece uma vantagem adicional em relação ao uso isolado do POCUS cardíaco ou do EFAST em pacientes com choque indiferenciado.

### 6.3. Perguntas Chave para Identificar o Tipo de Choque

Propõe-se, em conjunto com os achados ultrassonográficos correspondentes e com o tratamento implícito, uma sequência de cinco perguntas com o objetivo de identificar e tratar rapidamente patologias que apresentam uma "assinatura ultrassonográfica" característica^2,9^ (Figura 20):

- Há tamponamento?
- O paciente está hipovolêmico?
- Em caso de função ventricular esquerda deficiente, ela é a principal causa do choque?
- Há sinais de distensão do ventrículo direito?
- Há pneumotórax?

**Figura 20:**
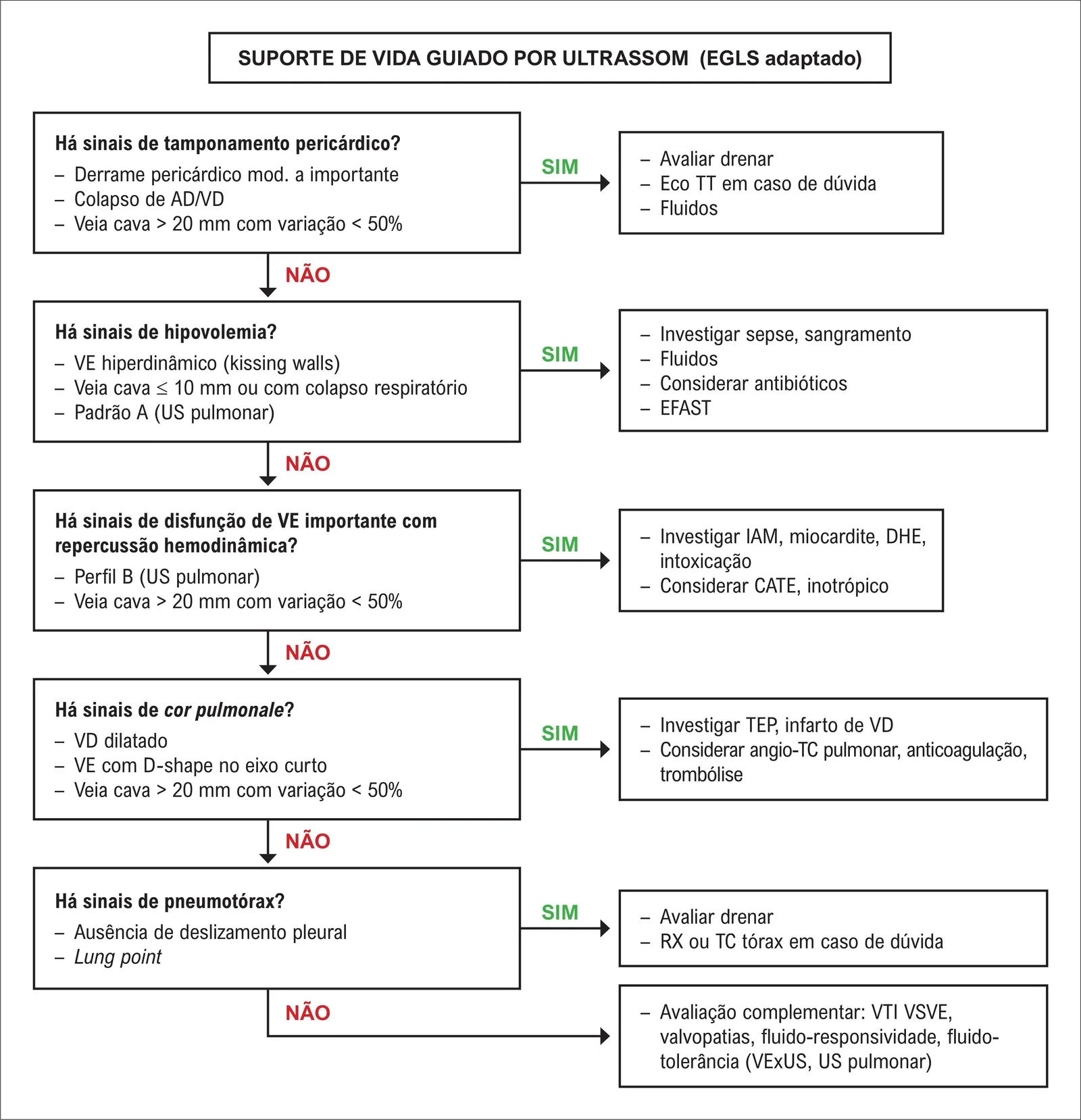
Protocolo EGLS adaptado.^8^ DHE: distúrbio hidroeletrolítico; CATE: cateterismo cardíaco.

Discutiremos abaixo cada uma das perguntas sugeridas.

#### a. Há tamponamento?

Deve-se investigar a presença de derrame pericárdico moderado a importante, VCI dilatada e com mínima variação respiratória em diâmetro, colapso diastólico das câmaras cardíacas e *swinging heart.*^9^

A fisiologia do tamponamento é sugerida por um derrame pericárdico que causa colapso atrial ou ventricular direito durante a diástole. Observa-se um movimento pendular do coração na presença de um derrame maciço (um *swinging heart* ou "coração oscilante"). O tamponamento é associado a uma pressão venosa central elevada, demonstrada na janela subcostal por uma VCI pletórica (20 mm) e sem variação respiratória.

Esses achados são discutidos em mais detalhes na Seção 4 do Capítulo 2 (POCUS no tamponamento cardíaco).

#### b. O paciente está hipovolêmico?

Deve-se investigar o ventrículo esquerdo com padrão hiperdinâmico/hipovolêmico (*kissing walls*) ou VCI fina (≤10 mm) ou com colabamento ao longo do ciclo respiratório.^146^

Em casos de choque hipovolêmico, espera-se encontrar pulmões limpos (ou seja, sem linhas B) associados a um VE hiperdinâmico e a uma VCI colapsável. Além disso, é importante investigar as possíveis etiologias do choque hipovolêmico, de modo que o exame EFAST deve ser considerado.

Estudos demonstram que uma VCI pequena (diâmetro inferior a 10 mm) está correlacionada com hipovolemia em pacientes com trauma. Logo, variações respiratórias substanciais ou colapso da VCI em pacientes em choque são sempre indicativos de possível hipovolemia,^10,11^ especialmente quando associados a um VE hiperdinâmico.

É importante lembrar que, nas fases iniciais da sepse, o paciente pode apresentar com esse perfil hiperdinâmico. Assim, é preciso considerar a sepse como etiologia.^12^

#### c. Em caso de função ventricular esquerda deficiente, ela é a principal causa do choque?

Deve-se investigar a presença de disfunção sistólica importante do VE e sinais de congestão pulmonar e venosa sistêmica.

Para que a etiologia do choque seja cardiogênica pura, é necessário que haja uma disfunção importante do VE. Normalmente, esses pacientes também apresentam sinais de congestão pulmonar (padrão B na USP) e/ou venosa sistêmica (dilatação da VCI).^13^

A síndrome intersticial pulmonar é definida pela presença de três ou mais linhas B na largura de um único espaço intercostal, o chamado perfil B. Esse padrão pode representar edema pulmonar cardiogênico,^13,14^ mas não é específico para essa patologia. Também está presente em outras doenças intersticiais,^16^ como fibrose pulmonar, SDRA e contusões pulmonares. O perfil B sugere origem cardiogênica quando apresenta distribuição homogênea, bilateral e dependente da gravidade, ou seja, é mais acentuado nas regiões basais do que nas superiores do pulmão.

É improvável que um choque cardiogênico puro seja a causa primária da instabilidade hemodinâmica na presença de um padrão pulmonar normal à ultrassonografia. Isso sugere que a administração de fluidos provavelmente é segura.^17^

O conhecimento prévio do padrão ecocardiográfico e pulmonar também pode auxiliar na interpretação dos achados subsequentes, pois um VE com disfunção associado a um padrão pulmonar normal sugere a presença de comorbidades cardíacas (ou seja, função ventricular esquerda cronicamente deprimida) e não choque cardiogênico puro, principalmente na presença de uma VCI pequena.

Por outro lado, em casos de choque misto (cardiogênico e distributivo) em um paciente com disfunção miocárdica associada à sepse, pode haver uma disfunção cardíaca aparentemente discreta associada a um estado vasoplégico ou hipovolêmico, o que explica a ocorrência do choque na ausência de disfunção importante do VE.^18^ É preciso lembrar que um coração normal se apresenta discretamente hiperdinâmico em um quadro de choque séptico, dada a redução da resistência vascular sistêmica. Caso um ventrículo tenha função sistólica limítrofe durante o choque séptico, deve-se considerar a possibilidade de disfunção miocárdica preexistente ou nova por disfunção miocárdica da sepse, que pode estar mascarada pela baixa resistência vascular. Nesses pacientes, a disfunção miocárdica pode se tornar mais evidente após o aumento da dose de vasopressor.

Uma condição que pode cursar com choque e VE hiperdinâmico é a regurgitação mitral aguda grave, como observado na ruptura de uma corda tendínea. Profissionais não especialistas e com experiência limitada na técnica Doppler podem interpretar essa condição erroneamente como hipovolemia. No entanto, em contraste com a hipovolemia, a regurgitação mitral aguda grave provavelmente estaria associada a um perfil B e a uma VCI menos complacente (pois associada a pressões de enchimento mais elevadas). Nesse exemplo, como mencionado anteriormente, os achados da USP podem influenciar a interpretação do POCUS cardíaco. Sugere-se que, na presença dessa suspeita, seja solicitada avaliação por um especialista em imagem (ecocardiografista).

#### d. Há sinais de distensão do ventrículo direito?

Deve-se investigar sinais de dilatação moderada a importante das câmaras direitas e aplainamento do septo interventricular (*D-shape*).

Em caso de choque obstrutivo, a VCI encontra-se dilatada. A dilatação (superior a 20 mm) e a perda da variabilidade respiratória na VCI sugerem pressão venosa central elevada.^4,18^

Se a causa do choque obstrutivo for *cor pulmonale* agudo, é obrigatória a presença de sinais de dilatação das câmaras direitas e aplainamento do septo interventricular (*D-shape*). Nessa condição, o VE adquire o formato em "D" na janela paraesternal transversal (Figura 7).^19,20^ A janela A4C é a melhor forma de avaliar a proporção VD/VE, o que permite estimar a gravidade do *cor pulmonale*:

- *Cor pulmonale* leve: VD > 60% do VE;
- *Cor pulmonale* moderado: VD = VE;
- *Cor pulmonale* grave: VD > VE.

#### e. Há pneumotórax?^21^

Deve-se investigar a ausência de deslizamento pleural e a presença de *lung point* (ponto pulmonar).A presença de deslizamento pulmonar exclui o pneumotórax na região examinada e requer treinamento mínimo para ser reconhecido. Embora não seja específica (Figura 20), a ausência do deslizamento pleural, no contexto apropriado, pode ser altamente sugestiva de pneumotórax. O ponto pulmonar, descrito no capítulo sobre USP, representa o achado mais específico para esse diagnóstico.

A ausência de ponto pulmonar não exclui a presença de pneumotórax. Na presença de pneumotórax circunferencial, esse sinal não está presente. A presença de padrão B exclui a possibilidade de pneumotórax na região examinada.

### 6.4. Fluido-Responsividade e Fluido-Tolerância

Dependendo do tipo de choque provável, outras informações complementares podem ser realizadas. Estas dependem do grau de experiência do examinador e da causa provável do choque.

Apesar das diferentes metodologias e definições presentes na literatura, a concepção mais aceita de fluido-responsividade (FR) é o aumento do débito cardíaco (DC) maior que 10-15% induzido pelo aumento da pré-carga.^22–24^

#### 6.4.1. Estimativa do Débito Cardíaco

O principal componente da estimativa de DC pelo POCUS cardíaco é a TVI da VSVE.^22^ O conceito é abordado na Seção 2 do capítulo sobre POCUS cardíaco avançado. O valor normal da TVI da VSVE situa-se em torno de 18 a 22 cm para pacientes com frequência cardíaca entre 55 e 95 bpm.^51^ Se o paciente apresenta função sistólica normal e TVI baixa, deve-se suspeitar choque hipovolêmico. Caso o paciente apresente função sistólica normal e TVI elevada, deve-se considerar o choque distributivo. A avaliação da TVI da VSVE também é descrita na seção sobre avaliação da FR, a seguir.

#### 6.4.2. Veia Cava Inferior/Superior

A avaliação do diâmetro máximo e da variabilidade da VCI pode ser utilizada para a estimativa da PAD e de FR. Para a estimativa da PAD, a VCI tem maior acurácia nos pacientes em ventilação espontânea.^147^ Para a estimativa de FR, os índices baseados na variabilidade da VCI em pacientes sob ventilação mecânica controlada, sem esforço respiratório, com volumes correntes e PEEP próximos do normal, apresentaram boa acurácia nos estudos iniciais (90%). Contudo, esse desempenho não se confirmou nas publicações posteriores (acurácia de 65%).^148–150^ A acurácia da VCS (acurácia de 75%) teve desempenho superior ao da VCI na predição da FR. Contudo, a avaliação da VCS exige a realização de ecocardiografia esofágica.^150^ Em pacientes sob ventilação espontânea, estudos mostram que a acurácia da VCI também tem baixa acurácia, com especificidade maior do que sensibilidade.

Assim, é possível afirmar que a avaliação da VCI pode contribuir para a avaliação do estado volêmico e da FR, sobretudo nos casos mais extremos:^8^

- Muito fina (≤10 mm) ou em colabamento ➔ provável fluido-responsividade e fluido-tolerância;
- Dilatada (>25 mm) e com variação respiratória mínima ➔ provável não fluido-responsividade e não fluido-tolerância.

A variação respiratória da VCI é frequentemente alterada em pacientes sob ventilação mecânica, portadores de cirrose e em doenças pulmonares crônicas, e deve ser interpretada com cautela. Embora a variação respiratória da VCI possa ser um bom preditor de FR em pacientes hipotensos sob ventilação mecânica, uma VCI pletórica sem variação respiratória, por si só, não contraindica a administração de fluidos. A ausência de variação respiratória deve ser interpretada com cuidado e considerada à luz do contexto clínico, pois, assim como valores elevados de PVC, não implica necessariamente falta de fluido-responsividade.

#### 6.4.3. Manobra de Elevação Passiva dos Membros – Passive Leg Raising

Entre os parâmetros de avaliação da FR com POCUS, o *passive leg raising* (PLR – elevação passiva dos membros) é o mais estudado.^151,152^ A manobra consiste em medir a TVI da VSVE com o paciente em decúbito com o tronco elevado a 45° e as pernas estendidas sobre o leito. Em seguida, abaixa-se a cabeceira do paciente para 0°, eleva-se os membros inferiores a 45° e mede-se a TVI cerca de 1 minuto após a elevação dos membros inferiores. Essas manobras devem ser realizadas de forma passiva, por meio do ajuste da inclinação da maca e não pela movimentação ativa do paciente. Um aumento maior ou igual a 10% da TVI provocado pelo PLR é capaz de prever o aumento do DC após infusão volêmica com acurácia de 84%, conforme estudos mais recentes.^153^

Contudo, a estimativa da TVI da VSVE está sujeita a variabilidade intraobservador e interobservador. Um estudo recente em pacientes de UTI mostrou que para um mesmo avaliador, duas medidas realizadas em tempos diferentes, sem deslocar o transdutor do ponto de insonação no tórax do paciente, a variabilidade na medida da TVI da VSVE foi de cerca de 11% (5 a 18%). Quando as duas medidas foram realizadas por profissionais diferentes, a variabilidade aumentou para 14% (8 a 26%).^154^ Assim, é preciso muita cautela na tomada de decisões terapêutica baseadas nesse parâmetro como estimativa de FR. Uma diferença entre as estimativas antes e após a manobra que esteja próxima do valor de variabilidade intraobservador (11%) pode representar um falso positivo. Logo, quando ocorre uma variação mais significativa da TVI induzida pela manobra (>20%), é mais provável que o paciente realmente seja fluido-responsivo.

Uma vantagem do PLR é que pode ser realizado em pacientes sob ventilação espontânea ou mecânica, com ou sem esforço respiratório, mesmo com volume corrente baixo. Todavia, a manobra requer maior experiência por parte do examinador para que as medidas da TVI tenham rapidez e precisão. Recomenda-se calcular a média de três ou mais medidas obtidas ao final da expiração em pacientes em ritmo sinusal e a média de cinco ou mais medidas em pacientes com fibrilação atrial.^154^ Alguns fatores prejudicam a utilização do PLR: hipertensão intra-abdominal, impossibilidade de mobilização dos membros inferiores (por exemplo, devido a fraturas), trombose dos membros inferiores e hipertensão intracraniana.

#### 6.4.4. Manobra da Pausa Expiratória e Inspiratória

Estudos identificaram que uma pausa expiratória de 12 a 15 segundos está associada a um aumento da TVI ou do DC em pacientes fluido-responsivos, enquanto uma pausa inspiratória de 12 a 15 segundos está associada à redução da TVI ou do DC no mesmo tipo de paciente.^33^ Essa avaliação pode ser realizada por ultrassonografia ou com o uso de outro equipamento de monitorização do DC. Todavia, como a margem de variação do DC para detectar a resposta à manobra está próxima da margem da variabilidade intraobservador das medidas realizadas com POCUS, sugere-se utilizar uma estratégia integrada que combine a pausa expiratória e a inspiratória.

A soma da variação absoluta de 13% ou mais da TVI da VSVE, com as manobras combinadas de teste de oclusão expiratória final (TOEF) e teste de oclusão inspiratória final (TOIF), com duração aproximada de 12 a 15 segundos cada, apresentou acurácia de 93% para a predição de FR. Contudo, essa avaliação não deve ser realizada por profissionais com pouca experiência.

É importante reforçar que nenhum parâmetro de estimativa de FR é perfeito. Diversos fatores podem interferir na sua acurácia, sejam eles relacionados ao paciente, ao equipamento ou ao operador.^153^ Além disso, nem todo paciente fluido-responsivo é fluido-tolerante. A decisão de ofertar fluidos deve sempre considerar s riscos individualizados do choque e da hipoxemia e congestão para o paciente específico.^155^

A fluido-tolerância (FT) é a capacidade do organismo de receber uma infusão de fluidos sem evoluir com disfunção orgânica. A FT pode ser avaliada por meio de USP para a pesquisa de linhas B (congestão pulmonar) e do protocolo VExUS (congestão venosa sistêmica). O protocolo VExUS, sua metodologia e sua interpretação estão descritos na Seção 4 deste posicionamento. Para a avaliação de FT, pode-se utilizar esse protocolo da seguinte forma:

- VExUS 0 e 1 (sem congestão significativa): fluido-tolerantes;
- VExUS 2 e 3 (congestão significativa): fluido-intolerantes.

Uma vez que o paciente é identificado como fluido-responsivo, é possível prosseguir com a infusão de fluidos, monitorando a resposta pela USP e o protocolo VExUS, conforme o fluxograma a seguir (Figura 21).^146^

**Figura 21:**
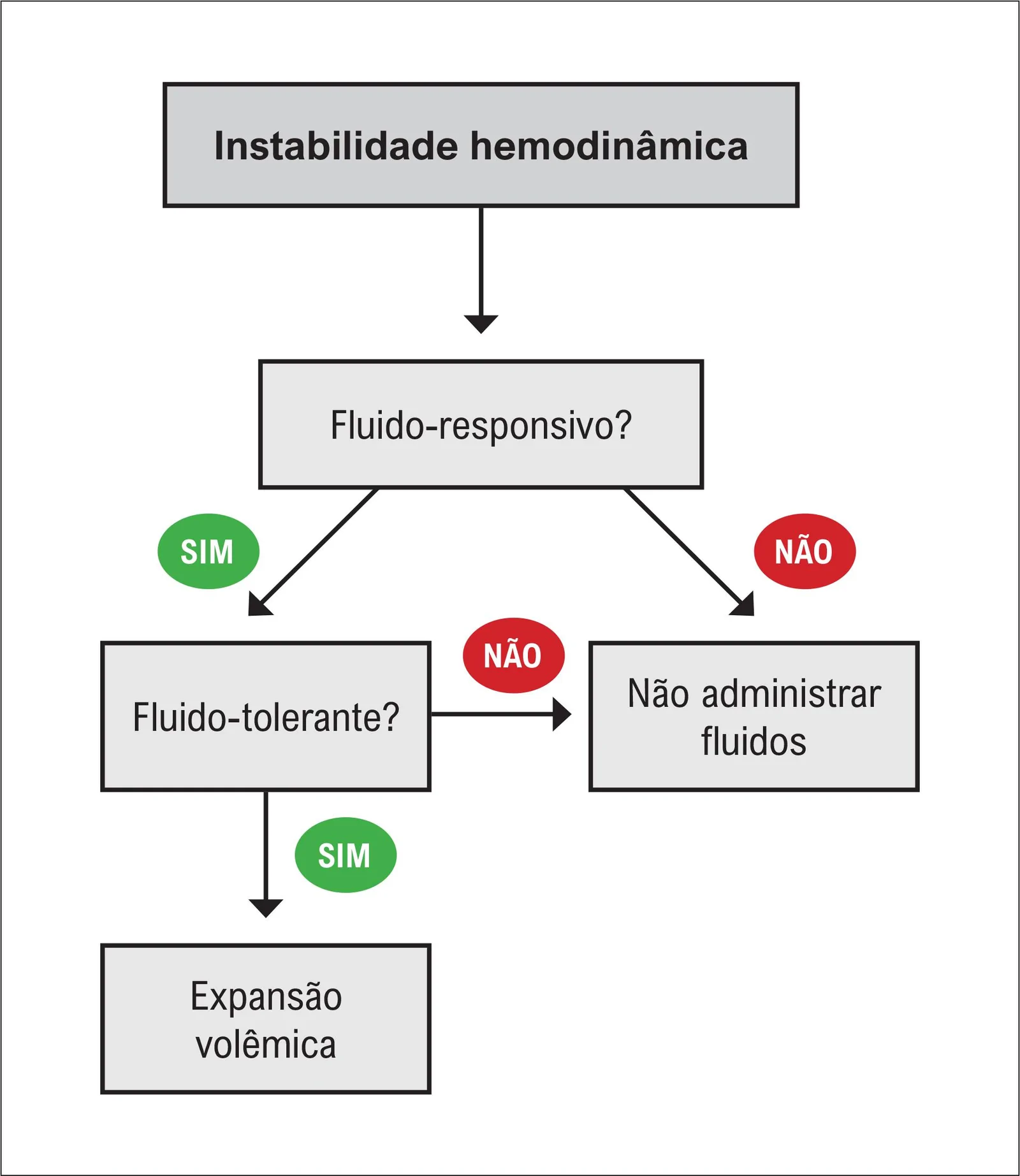
Abordagem integrada de fluido-responsividade e fluido-tolerância.^156^

O médico que atende o paciente à beira do leito deve sempre utilizar as informações obtidas pelo POCUS para auxiliar seu raciocínio e integrá-las às informações colhidas com a anamnese, exame físico e demais exames.

Considerando todos os tópicos discutidos neste capítulo, recomenda-se o uso de um algoritmo inicial para a abordagem do choque circulatório, com base nas informações obtidas pelo POCUS, que podem ser complementadas de acordo com o grau de experiência do observador (Figura 22).

**Figura 22:**
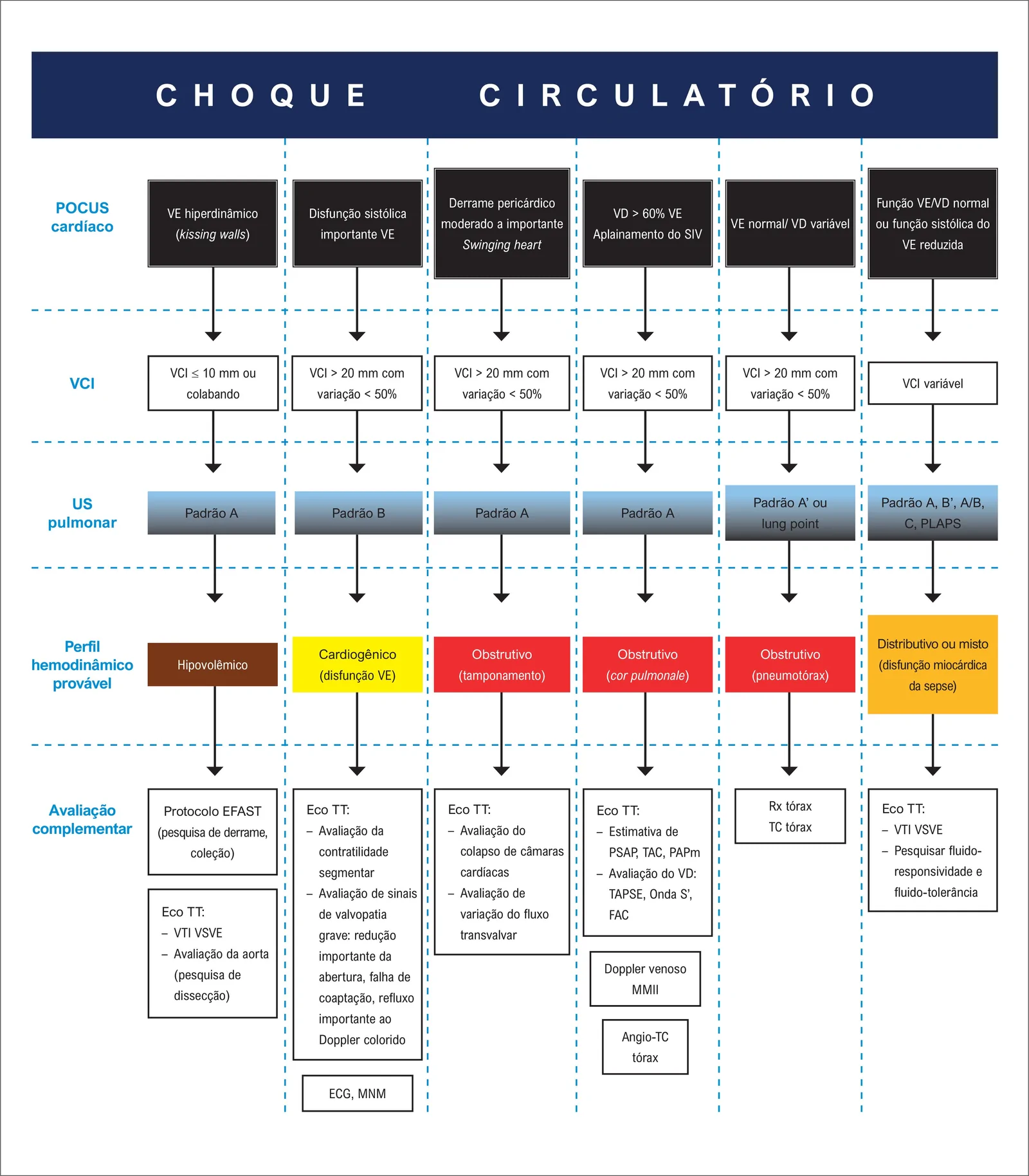
Algoritmo diagnóstico inicial do choque circulatório baseado no POCUS. VE: ventrículo esquerdo; VD: ventrículo direito; VCI: veia cava inferior; VTI; integral da velocidade no tempo; VCI: veia cava inferior; Eco TT: ecocardiograma transtorácico; Rx: radiografia; MNM: marcador de necrose miocárdica; PSAP: pressão sistólica de artéria pulmonar; TAC: tempo de aceleração pulmonar; PAPm: pressão média de artéria pulmonar; TAPSE: excursão sistólica do plano do anel tricúspide; FAC: variação da área fracional; TC: tomografia computadorizada; VExUS: *venous excess ultrasound score*; PLAPS: *posterolateral alveolar and/or pleural syndrome*. Adaptado dos protocolos EGLS, BLUE e EFAST.

## 7. POCUS no Suporte Circulatório Mecânico de Curto Prazo

### 7.1. Introdução

O choque cardiogênico (CC) é caracterizado por disfunção cardíaca que leva à hipoperfusão e à disfunção dos órgãos-alvo. A causa mais comum de CC é o IAM. Apesar dos avanços na revascularização precoce, a incidência de CC associado a IAM permanece entre 6,5 e 10,1%.^157^ Outras causas comuns incluem a piora da disfunção ventricular prévia e a disfunção cardíaca após cirurgia cardíaca. O CC está associado a mortalidade elevada, com uma taxa estimada de mortalidade hospitalar de 50%.^158^ A terapia clínica isolada muitas vezes não é suficiente, tornando necessário o uso de suporte circulatório mecânico de curto prazo (SCMCP).

Os dispositivos de SCMCP podem fornecer suporte hemodinâmico em dois cenários clínicos: (1) durante procedimentos cardíacos de alto risco em pacientes com baixo DC e (2) em CC decorrente de falência ventricular esquerda ou direita. Dispositivos de SCMCP, incluindo balão intra-aórtico (BIA), oxigenação por membrana extracorpórea venoarterial (ECMO-VA) e dispositivos de assistência ventricular percutânea (DAVP) Impella^®^, oferecem suporte vital para pacientes com CC. Além disso, os dispositivos Impella^®^ podem auxiliar na redução da carga ventricular esquerda durante a ECMO-VA. Um estudo recente mostrou o benefício do uso de Impella^®^ comparado à terapia médica padrão em pacientes com CC relacionado ao infarto.^159^ Os principais objetivos dos dispositivos de SCMCP são fornecer suporte circulatório e reduzir a disfunção dos órgãos-alvo no contexto de DC inadequado.

O POCUS revolucionou o diagnóstico e o manejo à beira do leito em diversos cenários médicos. Sua aplicação no SCMCP é particularmente notável devido à natureza crítica dos pacientes que necessitam desse tipo de intervenção. A integração do POCUS ao manejo desses pacientes fornece informações em tempo real e não invasivas que são cruciais para a tomada de decisões clínicas oportunas e eficazes.

### 7.2. O Papel do POCUS no Suporte Circulatório Mecânico de Curto Prazo

O POCUS é uma ferramenta inestimável e seu uso é recomendado para clínicos que manejam pacientes em SCMCP. A técnica fornece *feedback* visual imediato sobre a função cardíaca, o posicionamento do dispositivo e possíveis complicações. Seu papel abrange diversos aspectos, incluindo a avaliação inicial do paciente, a colocação do dispositivo e o monitoramento contínuo.

### 7.3. Avaliação Inicial do Paciente

No contexto do SCMCP, a avaliação inicial é fundamental para determinar a adequação do seu emprego e para planejar a intervenção. O POCUS permite a avaliação rápida da função cardíaca, do estado volêmico e de outros parâmetros hemodinâmicos. Por exemplo, em pacientes com CC, a ecocardiografia permite avaliar rapidamente a função ventricular esquerda e direita, anormalidades valvares e a presença de derrame pericárdico. Essas informações são essenciais para decidir o tipo e a urgência do suporte mecânico (SM) necessário.

### 7.4. Colocação e Confirmação da Posição do Dispositivo

O posicionamento correto do dispositivo de SM é essencial para a sua eficácia e para evitar complicações. O POCUS desempenha um papel importante na orientação e na confirmação do posicionamento de dispositivos como BIA, cânulas de ECMO e DAVP.

### 7.5. Balão Intra-Aórtico

#### 7.5.1. Introdução

Embora diretrizes mais recentes da Sociedade Europeia de Cardiologia (ESC) para IC não recomendem o uso rotineiro de BIA,^160^ ele continua a ser o dispositivo de suporte circulatório mecânico percutâneo de curto prazo mais utilizado, com mais de 60.000 implantes realizados por ano.^161^ O cateter do BIA geralmente é inserido através da artéria femoral ou, com menos frequência, pelas artérias axilar ou subclávia. Na abordagem femoral, o cateter é avançado retrogradamente pela aorta descendente até que a extremidade esteja posicionada logo distalmente à artéria subclávia esquerda (ASCE). O BIA funciona por contrapulsação na aorta torácica descendente, com insuflação durante a diástole e desinsuflação durante a sístole. Esse mecanismo reduz a pós-carga do VE na sístole e melhora a perfusão coronariana na diástole. A ecocardiografia transesofágica (ETE) apresenta vantagens em relação à fluoroscopia, pois permite a visualização em tempo real das estruturas, é portátil, não utiliza radiação ionizante e também permite avaliar tanto as indicações para o uso do BIA quanto as suas possíveis complicações. Além disso, a ETE permite excluir contraindicações primárias antes da inserção, como regurgitação aórtica (RA) e dissecção aórtica.

A ecocardiografia também pode ser utilizada para monitorar a resposta ao BIA. Ntalianis et al. demonstraram, por meio da ecocardiografia transtorácica, que pacientes tratados com BIA apresentaram remodelamento reverso do VD e melhora da função ventricular.^162^ Quando usado em conjunto com a ECMO-VA para CC, o BIA diminui marginalmente a pós-carga do VE, aumenta a pulsatilidade do fluxo na aorta, ajuda a prevenir a distensão do VE e pode reduz o edema pulmonar hidrostático.^163^ O posicionamento adequado do BIA é fundamental para a sua eficácia e para prevenir complicações, como isquemia de membros e dissecção aórtica.

#### 7.5.2. Sequência de Exames de Ecocardiografia Transesofágica para Orientar a Inserção do Balão Intra-Aórtico

Uma ETE abrangente deve ser realizada primeiro, seguida de uma avaliação direcionada da válvula aórtica, raiz aórtica, aorta ascendente, arco aórtico e aorta descendente para excluir ateromas, dilatação aneurismática e dissecção. A válvula aórtica deve ser avaliada nos planos do eixo longo e eixo curto ao nível do esôfago médio e qualquer grau de RA deve ser documentado. Em seguida, confirma-se a presença do fio-guia na aorta descendente e, por meio da visualização em eixo curto da aorta descendente, identifica-se a extremidade do balão. Ainda nesse plano, pode-se estimar a distância entre a extremidade do balão e a origem da ASCE. Fixa-se a ponta dos dedos ao nível dos dentes do paciente e, a partir desse ponto, retira-se gradualmente a sonda até o aparecimento da ASCE. A distância entre os dedos do ecocardiografista e os dentes do paciente corresponde à distância entre a extremidade do balão e a origem da ASCE. A seguir, o cateter pode ser avançado para a distância apropriada, garantindo que a extremidade do BIA seja visualizada cerca de 2 cm distal à origem da ASCE.

#### 7.5.3. Lista de Verificação Pós-Implantação

- Determine a presença de complicações como lesão aórtica, ruptura de placa de ateroma, trombose ou tromboembolismo e ruptura do balão;
- Avalie a eficácia do BIA: dimensões do VE, função, TVI da VSVE;
- Avalie a melhoria do fluxo coronariano usando Doppler colorido/PW, indicada por um aumento da velocidade diastólica com a insuflação do balão.

### 7.6. Oxigenação por Membrana Extracorpórea Venoarterial (ECMO-VA)

#### 7.6.1. Introdução

A ECMO é uma forma crítica de suporte à vida utilizada em pacientes com IC e insuficiência respiratória grave. A ecocardiografia, e particularmente o POCUS, tem um papel multifacetado na ECMO, abrangendo seleção de pacientes, orientação da canulação, monitoramento hemodinâmico e detecção de complicações. Esta seção explora cada um desses papéis, enfatizando a importância da ecocardiografia e do POCUS em otimizar a terapia com ECMO e potencialmente melhorar os resultados dos pacientes.

A ecocardiografia é útil para identificar ou excluir condições reversíveis de declínio hemodinâmico, como tamponamento cardíaco, lesões valvares não diagnosticadas e disfunção do VE, o que elimina a necessidade de suporte com ECMO. Ela também fornece informações críticas sobre contraindicações; por exemplo, a ECMO não deve ser utilizada em casos de dissecção aórtica. A presença de RA importante é uma contraindicação relativa para ECMO-VA, pois aumenta a pós-carga do VE, o que pode exacerbar a RA. Além disso, a ecocardiografia pode detectar a presença de aterosclerose aórtica, o que informa a decisão sobre os locais (central versus periférica) e métodos (cirúrgico versus percutâneo) de canulação. A avaliação da morfologia do coração direito ajuda a identificar anormalidades estruturais que dificultariam a colocação de uma cânula venosa para ECMO venovenosa (ECMO-VV) ou ECMO-VA. A Figura 23 sintetiza os principais pontos de atenção relacionados à ECMO-VV e ECMO-VA nas fases de pré-canulação, durante o período de ECMO e no processo de desmame, assim como possíveis causas de mal funcionamento.

**Figura 23:**
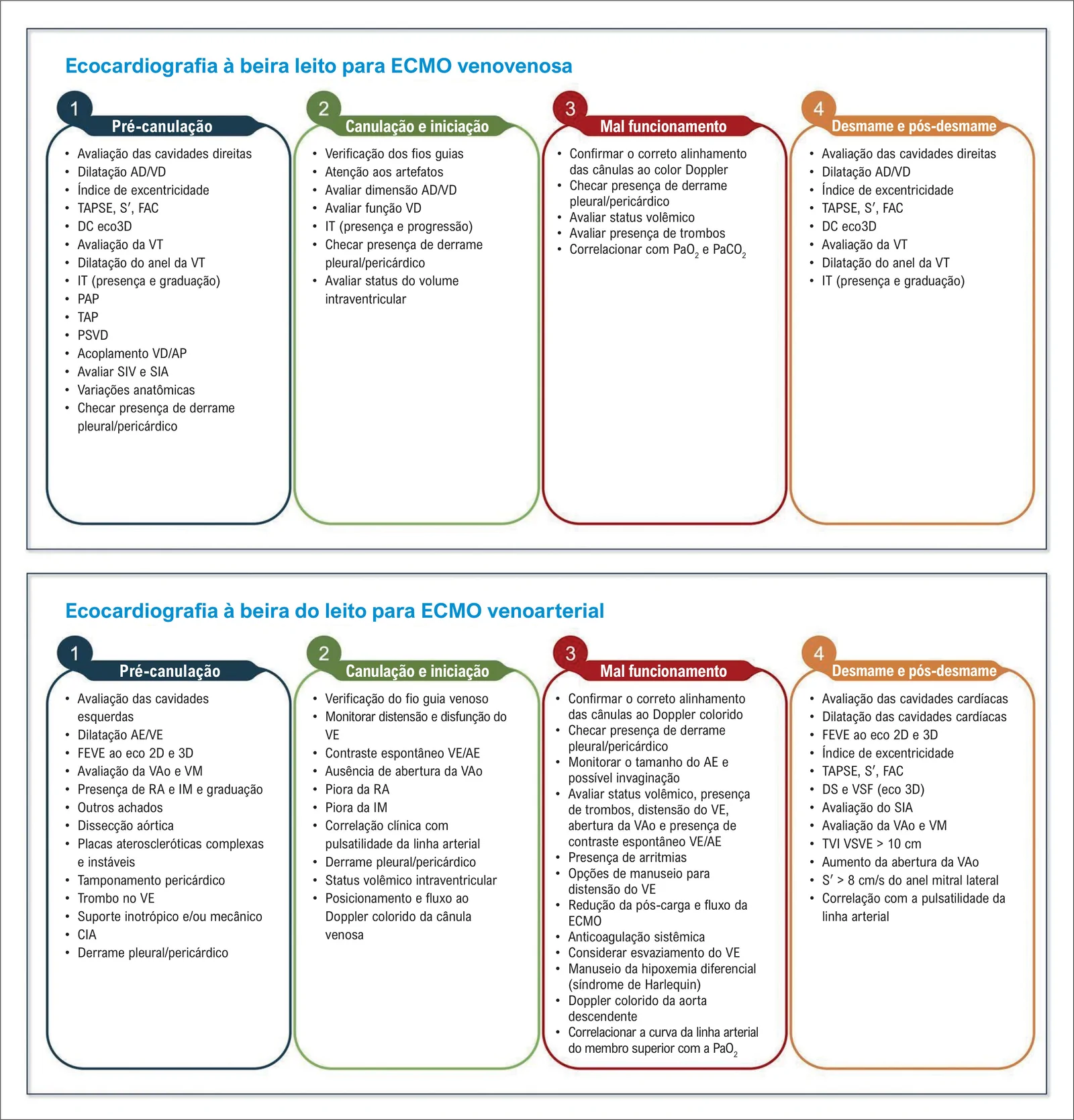
Ecocardiografia à beira do leito na ECMO-VV e VA. AD: átrio direito; AP: artéria pulmonar; DS: débito sistólico; FAC: alteração fracionária da área; FEVE: fração de ejeção do ventrículo esquerdo; IT: insuficiência tricúspide; PAP: pressão da artéria pulmonar; PSVD: pressão sistólica do ventrículo direito; RA: regurgitação aórtica; SIA: septo interatrial; SIV: septo interventricular; TAP: tempo de aceleração do fluxo pulmonar; TAPSE: excursão sistólica do plano do anel tricúspide; TVI: integral tempo-velocidade; VAo: válvula aórtica; VD: ventrículo direito; VM: válvula mitral; VS: volume sistólico; VSF: volume sistólico final; VT: válvula tricúspide. Observação: A ETE deve ser realizada em todos os pacientes quando o tempo e a situação clínica permitirem. Alternativamente, a ETT pode oferecer informações importantes durante a avaliação pré-ECMO e o processo de desmame e deve ser considerada como uma opção não invasiva.

#### 7.6.2. Seleção de Pacientes para ECMO

A avaliação inicial e a seleção de pacientes para ECMO são fundamentais para o sucesso da intervenção. A ecocardiografia fornece informações vitais sobre a estrutura e a função cardíacas, ajudando a determinar a adequação da ECMO.^164^ Antes de iniciar a ECMO, é essencial avaliar a função cardíaca do paciente para determinar a gravidade da IC e o possível benefício do tratamento. A ecocardiografia oferece informações detalhadas sobre a função do VE e do VD, a integridade valvar e a presença de doenças cardíacas estruturais.

A disfunção do VD pode complicar o manejo de pacientes em ECMO. A ecocardiografia permite avaliar o tamanho, a função e as pressões do VD, cruciais para o planejamento da estratégia de ECMO. A ecocardiografia é essencial para identificar anomalias valvares, como regurgitação mitral grave e estenose aórtica, que podem influenciar a decisão de iniciar a ECMO. A identificação e o manejo adequados dessas condições são fundamentais para otimizar a terapia com ECMO.

A instabilidade hemodinâmica é uma indicação frequente para ECMO. A ecocardiografia fornece informações em tempo real sobre o estado hemodinâmico, incluindo DC, pressões de enchimento e status volêmico. A ecocardiografia pode estimar o débito cardíaco pela medição do volume sistólico e da frequência cardíaca. Essas informações são essenciais para determinar a necessidade de ECMO e monitorar a eficácia do suporte. Avaliar o status de volume intravascular por ecocardiografia ajuda a orientar o manejo de fluidos, o que é essencial para estabilização hemodinâmica antes e durante a ECMO. Parâmetros como o diâmetro a variação respiratória da VCI e são indicadores úteis do status volêmico.

#### 7.6.3. Orientação para Canulação

A canulação precisa é uma etapa fundamental para o início da ECMO, e a ecocardiografia é de suma importância na orientação e confirmação do posicionamento das cânulas de ECMO. Na ECMO-VA periférica, as cânulas são inseridas em uma veia (em geral, a veia femoral) e em uma artéria (em geral, a artéria femoral) para fornecer suporte circulatório e respiratório. A ETE é o método de escolha, uma vez que a ecocardiografia transtorácica (ETT) pode não oferecer resolução espacial suficiente. A comunicação eficaz entre o operador de ECMO e o ecocardiografista em relação ao local pretendido para o posicionamento das cânulas é fundamental. Por exemplo, na ECMO-VV, quando uma cânula é usada para drenagem e outra para o retorno do sangue, a extremidade da cânula de drenagem deve ser posicionada na porção proximal da VCI, imediatamente antes de seu deságue no AD. Já a cânula de retorno deve ser posicionada no meio do AD, afastada do septo interatrial e da válvula tricúspide.

O uso da ecocardiografia para monitorar a ECMO é particularmente importante para avaliar a hemodinâmica e o status volêmico.^165^ Em pacientes com disfunção grave do VE associado a regurgitação mitral grave, o VE pode se tornar substancialmente dilatado e a válvula aórtica pode não se abrir devido à redução acentuada do DC. O resultado pode ser estase sanguínea e consequente trombose na aorta ascendente, nas cavidades esquerdas e nas veias pulmonares. Nessas situações, o esvaziamento do VE ou uma atriosseptostomia percutânea por balão podem ser benéficos. A ETE é o método ideal para guiar o posicionamento do cateter da septostomia e a insuflação do balão.

A ausência de abertura da válvula aórtica (VAo) durante o suporte periférico da ECMO-VA representa um problema sério. Nesses casos, deve-se aumentar a dose de medicamentos anticoagulantes e recorrer a inodilatadores para reduzir a pós-carga, otimizar o DC nativo do VE e, com isso, facilitar a abertura da VAo. Nesse cenário, a transição do circuito de ECMO para um dispositivo de assistência ventricular também deve ser considerada.

Na ECMO-VV, a hemodinâmica é mais simples e mais vantajosa para o paciente. Nesse modo de suporte, o sangue é drenado e devolvido ao coração direito, sem alterar substancialmente a pré-carga do VD e sem impacto negativo na hemodinâmica do coração esquerdo. A ECMO-VV também aumenta a saturação de oxigênio venoso misto, o que contribui para uma diminuição da resistência vascular pulmonar e, consequentemente, menor pós-carga do VD. A melhora da oxigenação sanguínea também contribui indiretamente para a função do VE ao aumentar a oferta de oxigênio para as artérias coronárias.

Na ECMO-VA, a canulação pode ser realizada à beira do leito em situações de emergência ou na sala de hemodinâmica se as condições clínicas do paciente o permitirem. Nesses casos, deve-se optar por uma avaliação ecocardiográfica abrangente, incluindo ETT e/ou ETE associada a fluoroscopia, se necessário.

Nos casos de extrema urgência, em pacientes sem condições clínicas para transporte, a canulação deve ser realizada à beira do leito. Nesses casos, a ecocardiografia focada deve ser utilizada para guiar o procedimento, desde que realizada por um profissional experiente. As etapas da ultrassonografia focada para o procedimento incluem:

- Acesso vascular guiado por US em tempo real;
- Confirmação ultrassonográfica das posições dos fios-guia na VCI e na aorta por meio da janela trans-hepática na linha médio-axilar;
- Monitoramento contínuo para garantir que os fios permaneçam posicionados corretamente durante a troca das cânulas;
- Verificação de que o fio venoso não migrou para o VD ou se estendeu até a VCS;
- Confirmação do posicionamento da cânula venosa direcionada para a VCS;
- Confirmação do posicionamento da cânula arterial na aorta;
- Exclusão de complicações imediatas, como derrame pericárdico, dissecção aórtica ou posicionamento incorreto das cânulas (Figura 24).

**Figura 24:**
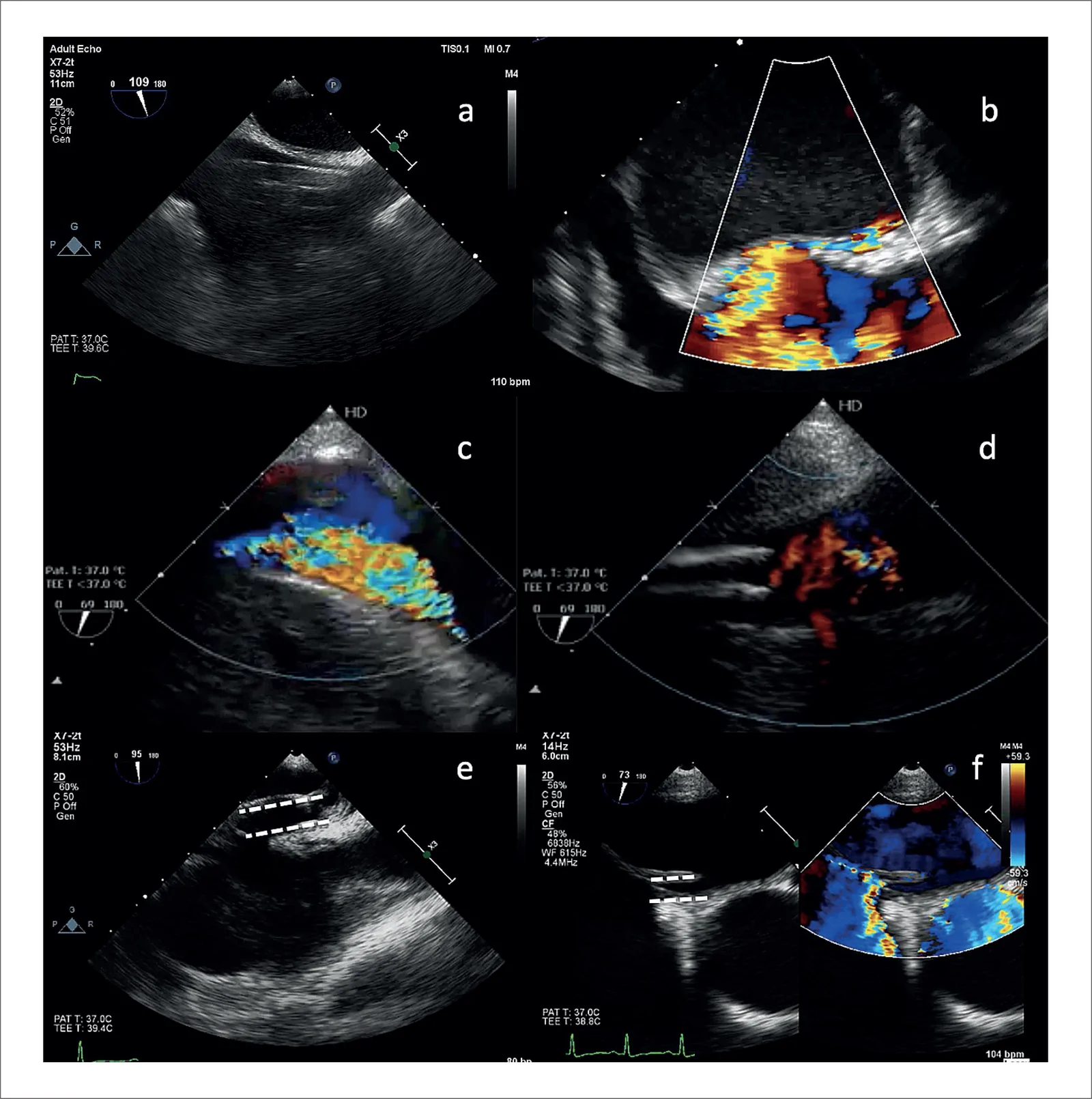
Exemplos de situações que demonstram o papel da ecocardiografia transesofágica na avaliação de pacientes em oxigenação por membrana extracorpórea venoarterial (ECMO-VA). (a) Corte bicaval mostrando a cânula de drenagem venosa em posição adequada com a extremidade na junção da veia cava superior com o átrio direito. (b) Corte bicaval modificado em paciente com dificuldade de oxigenação durante o processo de desmame. O Doppler colorido mostra a presença de forame oval patente com um shunt da direita para a esquerda justificando a hipóxia. (c) Corte transgástrico da veia cava inferior com Doppler colorido mostrando sinais de recirculação da oxigenação por membrana extracorpórea venovenosa (ECMO-VV), com fluxo turbulento devido à curta distância entre as extremidades das cânulas de drenagem e de retorno, o que faz com que a cânula de drenagem aspire sangue da extremidade da cânula de retorno). (d) Corte transgástrico da veia cava superior no mesmo paciente em ECMO-VV após o reposicionamento da cânula de drenagem alguns centímetros abaixo. O Doppler colorido mostra um fluxo linear, indicando menor recirculação. (e) e (f) Corte bicaval ao nível do esôfago médio em paciente em ECMO-VA, mostrando a cânula de drenagem atravessando o septo interatrial através do túnel da fossa oval, gerando pequena comunicação interatrial iatrogênica.

Deve-se enfatizar que não se trata de uma ultrassonografia básica e sim de uma ecocardiografia focada, a qual deve ser realizada por ecocardiografista, uma vez que requer um protocolo bem definido e preciso. Ela não substitui uma avaliação ecocardiográfica abrangente, que deve sempre ser realizada antes e depois da canulação. Se o tempo permitir, uma combinação de US, ETT e ETE é a alternativa ideal, e recomenda-se uma ETE completa durante ou após a canulação para confirmar o posicionamento da cânula venosa e detectar possíveis patologias cardíacas não diagnosticadas pela ETT.

#### 7.6.4. Efeitos Imediatos da ECMO-VA Periférica Observados pela Ecocardiografia à Beira do Leito

Os efeitos imediatos da ECMO-VA podem incluir um aumento gradual do volume do VE. Se a função do VE estiver comprometida, a válvula aórtica pode abrir de forma intermitente ou mesmo não abrir, com aparecimento de contraste espontâneo no interior do VE. Esses achados devem ser comunicados ao consultor de ECMO, pois podem exigir estratégias adicionais para descompressão do VE. Embora a situação não represente uma emergência, deve ser realizada uma avaliação ecocardiográfica abrangente para confirmar o diagnóstico e a patologia, avaliar as pressões de enchimento e determinar a melhor estratégia de alívio.

Todo paciente em suporte circulatório mecânico (SCM) agudo deve ser submetido a uma ecocardiografia diária. Em caso de deterioração hemodinâmica aguda ou falha no circuito, a equipe de cuidados intensivos pode solicitar um exame focado para identificar rapidamente ou excluir complicações específicas, como:

- Efusão pericárdica: Pode ser grande sem repercussão hemodinâmica ou pequena com repercussão hemodinâmica se a drenagem cardíaca estiver prejudicada;
- Deslocamento ou migração da cânula (com ou sem obstrução);
- Trombo relacionado à cânula (com ou sem obstrução);
- Descompressão ventricular excessiva;
- Trombose valvar ou intracavitária;
- Agravamento da regurgitação valvar (volume e/ou duração);
- Alívio inadequado do VE caracterizado por contraste espontâneo no VE, fluxo transmitral retrógrado bifásico ou fluxo sistólico pulmonar retrógrado;
- Encurtamento do período pós-ejeção (medido pelo TAPSE/MAPSE).

Esses achados devem ser identificados ou excluídos por meio de protocolos de ecocardiografia focada, executados por um ecocardiografista experiente. Como em toda ecocardiografia de cuidados intensivos, os achados devem ser interpretados à luz do contexto clínico e debatidos com o médico responsável. Se as imagens de ETT forem inadequadas, e a menos que haja contraindicações, uma ETE deve ser realizada. O uso de contraste no lado esquerdo, com objetivo de melhorar a interpretação das imagens em pacientes em SCM, deve ser feito apenas por profissionais experientes, pois a detecção de bolhas pode interromper imediatamente o funcionamento de alguns circuitos de SCM (por exemplo, Maquet^®^ ou Cardiohelp^®^). Após qualquer intervenção corretiva (por exemplo, ajustes no fluxo do SCM ou na posição da cânula), o coração e a circulação devem ser reavaliados em tempo real.

Para pacientes em suporte univentricular, o impacto no ventrículo contralateral e possíveis deslocamentos do septo interventricular também devem ser avaliados. Esse tipo de avaliação é altamente especializada, exigindo um entendimento aprofundado de fisiologia cardíaca, SCM e técnicas avançadas, como a deformação longitudinal global, que estão além do escopo do POCUS básico.

#### 7.6.5. Avaliação do Alívio e Desmame

O desmame da ECMO-VA e do suporte percutâneo do lado esquerdo envolve protocolos complexos, geralmente combinados com monitoramento hemodinâmico invasivo e adaptados à condição clínica do paciente.^166^ Esse processo está além do escopo dos protocolos de ultrassonografia focada e requer o uso de técnicas ecocardiográficas avançadas. No entanto, à medida que o conhecimento sobre o desmame do SCM agudo avança, a imagem e os estudos focados podem se tornar mais padronizados e desempenhar um papel maior no futuro.

O desmame da ECMO requer uma avaliação cuidadosa da função cardíaca e respiratória para garantir que o paciente consegue manter uma hemodinâmica adequada sem suporte mecânico. A ecocardiografia é essencial para esse processo. Ela avalia a recuperação da função cardíaca e fornece informações essenciais para determinar o melhor momento para o desmame. A melhora da função global e da fração de ejeção do VE geral indica recuperação e o momento adequado para o desmame da ECMO-VA. Avaliações ecocardiográficas seriadas ajudam a monitorar essa evolução. A recuperação da função do VD é igualmente importante. A ecocardiografia avalia o tamanho, a função e as pressões do VD, orientando o processo de desmame. Em pacientes em ECMO-VV, a ecocardiografia auxilia na avaliação da função pulmonar e prontidão para o desmame.^167^ A USP, como complemento da ecocardiografia, ajuda a avaliar a congestão pulmonar e a orientar o manejo de fluidos. A presença de linhas B à USP indica edema intersticial, o que pode influenciar as decisões de manejo de fluidos. A melhora nos achados da USP apoia a decisão de desmame da ECMO-VA e VV.

#### 7.6.6. Monitoramento Durante Testes de Desmame

A ecocardiografia permite monitoramento em tempo real durante os testes de desmame, o que ajuda a garantir que o paciente consiga manter uma hemodinâmica adequada sem o suporte da ECMO. Também desempenha um papel fundamental na detecção precoce e no manejo das complicações associadas à ECMO:

- Trombose: A trombose representa um risco grave em pacientes em ECMO devido à presença de materiais estranhos e à hemodinâmica alterada. A ecocardiografia pode detectar trombos intracardíacos ou intravasculares e permitir uma intervenção precoce. Ela pode identificar trombos dentro das câmaras cardíacas, o que é crucial para prevenir eventos embólicos. A detecção precoce orienta o início de terapia anticoagulante ou outras intervenções. Trombos também podem se formar nos locais de inserção das cânulas, o que cria um risco de embolização ou obstrução do fluxo. A ecocardiografia pode detectar esses trombos e orientar seu manejo;
- Sangramentos e hematomas: O sangramento é uma complicação comum em pacientes em ECMO, muitas vezes relacionado à terapia anticoagulante. A ecocardiografia pode identificar derrames pericárdicos e hematomas, que exigem manejo imediato. Ela pode detectar derrames pericárdicos, que podem ocorrer devido a sangramentos ou inflamações. A detecção e drenagem rápidas de derrames substanciais são críticos para a estabilidade do paciente. Hematomas ao redor dos locais de inserção das cânulas podem ser identificados por meio da ultrassonografia focada, possibilitando o manejo apropriado e a prevenção de complicações adicionais;
- Distensão ventricular: Em pacientes em ECMO-VA, a distensão ventricular pode ocorrer devido ao alívio inadequado do VE. A ecocardiografia permite avaliar as dimensões e a função ventricular e orienta intervenções para aliviar a distensão. A ecocardiografia permite monitorar o grau de distensão do VE e orienta medidas terapêuticas, como o uso de um BIA, Impella^®^ ou esvaziamento cirúrgico do VE. A distensão do VD pode ser manejada pelo ajuste dos parâmetros da ECMO ou pela utilização de suporte mecânico adicional. O monitoramento ecocardiográfico é fundamental para detectar e manejar essa complicação.

### 7.7. Desafios e Direções Futuras

Embora ofereça inúmeros benefícios para o manejo do SCMCP, o POCUS também apresenta desafios que precisam ser enfrentados. A precisão e a eficácia do POCUS dependem muito da habilidade e da experiência do operador. O treinamento inadequado ou a falta de experiência podem levar à interpretação equivocada das imagens e a decisões clínicas inadequadas. Para enfrentar esse problema, programas de treinamento padronizados e certificações para clínicos que usam POCUS no manejo da SCM são essenciais para melhorar a acurácia diagnóstica e os resultados clínicos em ambientes de cuidados intensivos.

Embora seja uma ferramenta poderosa, o POCUS deve ser integrado a outras modalidades de imagem, como a ecocardiografia, tomografia computadorizada e a ressonância magnética, para uma avaliação abrangente do paciente. A combinação de POCUS com outras modalidades proporciona um entendimento mais detalhado de casos complexos, o que aprimora a acurácia diagnóstica e o planejamento terapêutico. O futuro do POCUS no SCMCP reside em avanços tecnológicos que melhoram a qualidade da imagem, a portabilidade e a facilidade de uso. O desenvolvimento de dispositivos de ultrassonografia portáteis de alta resolução e o uso de IA na análise de imagens prometem melhorar a acurácia e a eficiência do POCUS.

### 7.8. Conclusão

O POCUS se tornou uma ferramenta crítica para o manejo de pacientes que necessitam de SCMCP. Sua capacidade de fornecer avaliações em tempo real e não invasivas da função cardíaca, do posicionamento de dispositivos e da ocorrência de complicações em potencial o torna extremamente valioso em ambientes de cuidados intensivos. Embora existam desafios, como a dependência do operador e a necessidade de integração com outras modalidades, os avanços tecnológicos e a capacitação profissional tendem a mitigá-los. À medida que o POCUS evolui, seu papel no aprimoramento dos cuidados e dos desfechos para pacientes em SCMCP deve se expandir, consolidando seu lugar na medicina intensiva moderna.

## 8. POCUS na Unidade de Emergência

O POCUS se estabeleceu como ferramenta essencial para a tomada rápida de decisões na avaliação inicial de pacientes críticos nas unidades de emergência. Apesar dos seus benefícios, recomenda-se que o POCUS não seja interpretado isoladamente nem substitua a anamnese cuidadosa e o exame físico convencional. O método pode fornecer informações valiosas no manejo, especialmente de pacientes críticos e/ou com possíveis diagnósticos de doenças tempo-dependentes.

A seguir, são apresentadas as aplicações do POCUS em diferentes cenários nas unidades de emergência.

### 8.1. POCUS na Dor Torácica

A dor torácica é uma queixa comum no pronto-socorro e, muitas vezes, representa um desafio. O POCUS é uma ferramenta valiosa para auxiliar no diagnóstico diferencial de condições potencialmente fatais, permitindo uma avaliação rápida e à beira do leito. Alguns diagnósticos que implicam risco para o paciente com dor torácica podem ser confirmados ou excluídos com o uso de POCUS, incluindo TEP, pneumotórax e pericardiopatias.

É fundamental enfatizar que a avaliação da aorta com suspeita de dissecção e a análise da contratilidade segmentar do VE para a identificação de síndrome coronariana aguda são técnicas que demandam treinamento avançado e experiência considerável em ecocardiografia. Dada a complexidade e a importância desses diagnósticos, sugere-se que essas avaliações sejam realizadas por especialistas em imagem cardiovascular para garantir a acurácia diagnóstica e a segurança do paciente.^168^

Quando utilizado de forma apropriada e por profissionais devidamente treinados, o POCUS é uma ferramenta poderosa para auxiliar na tomada de decisões clínicas.

As seções a seguir resumem o papel do POCUS nos principais diagnósticos relacionados à dor torácica.

#### 8.1.1. Avaliação de Pericardiopatias na Unidade de Emergência

A avaliação do pericárdio integra a análise sistemática do coração pelo POCUS e é muito útil em pacientes com instabilidade hemodinâmica, dispneia e/ou dor torácica.^169,170^

O exame tem grande valor na identificação rápida e precisa de derrames pericárdicos e de sinais de restrição ao enchimento ventricular à beira do leito (Figura 8), e é muito útil para guiar a pericardiocentese (Figuras 25 e 26), procedimento detalhado na Seção 10.2.^169,170^ A identificação de derrame pericárdico tem alta acurácia (acima de 95%), mesmo quando o POCUS é realizado por emergencistas (não cardiologistas), desde que tenham recebido treinamento adequado.^169^

**Figura 25:**
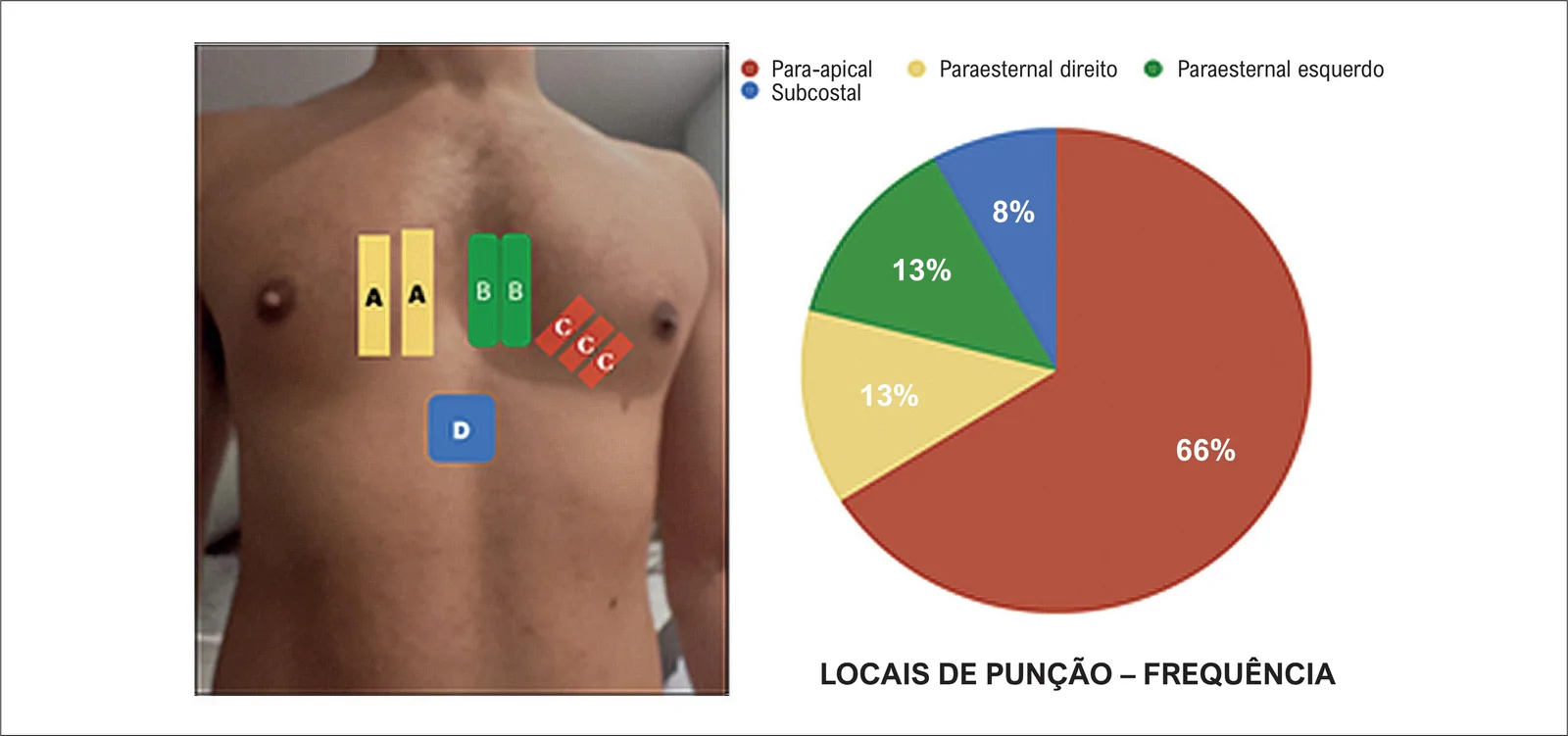
Janelas ecocardiográficas utilizadas para pericardiocentese guiada por ultrassonografia e a frequência de uso na série da Clínica Mayo.^188^

**Figura 26:**
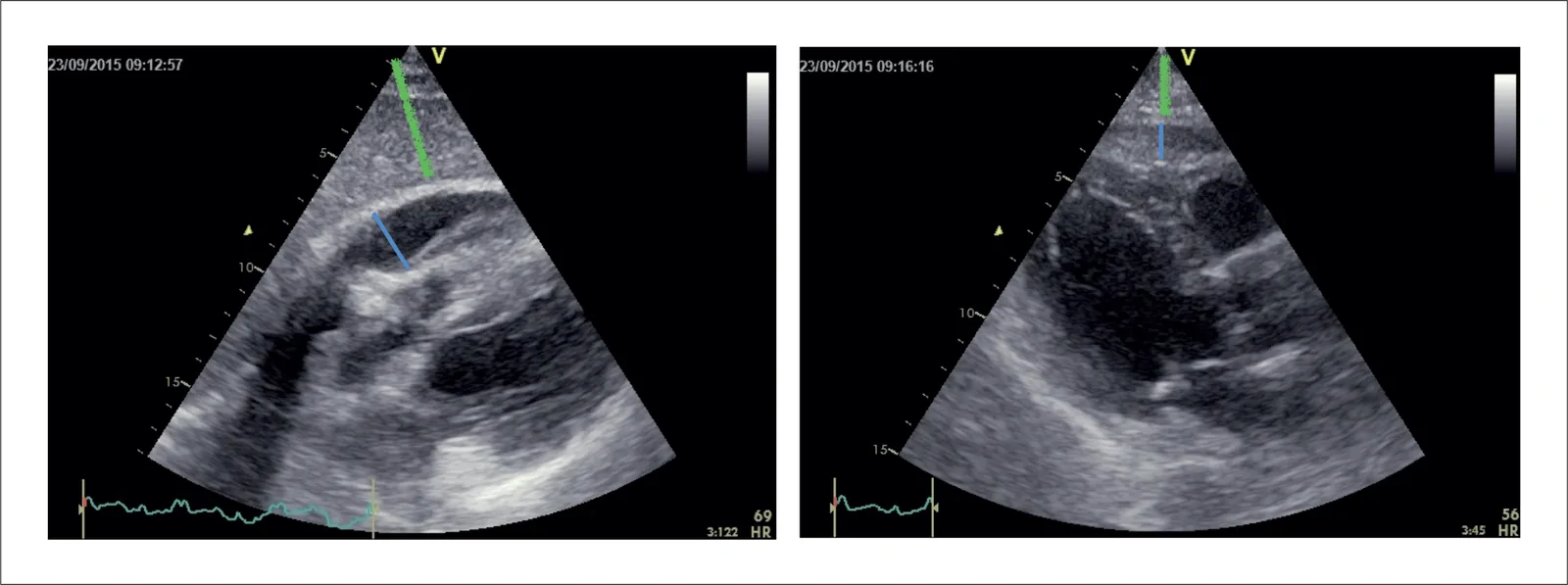
Identificação do ponto de maior acúmulo de líquido (linha azul) e do ponto com menor distância entre a pele e o espaço pericárdico (linha verde).

A medida do derrame pericárdico (Tabela 3) pode ser realizada nas janelas ecocardiográficas principais (paraesternal eixo longo, paraesternal eixo curto, A4C e subcostal) e permite classificar o derrame pericárdico.^49^

Além do volume de líquido anormal, a velocidade de progressão e a localização do derrame podem ultrapassar a capacidade de elasticidade do pericárdio em contê-lo e, por consequência, levar ao aumento da pressão intrapericárdica e à restrição ao enchimento atrial e ventricular.^171^ Nesses casos, alguns achados ecocardiográficos identificados ao POCUS (Tabela 6) podem corroborar o diagnóstico de tamponamento cardíaco (na presença de um quadro clínico compatível).^49,169^

**Tabela 6:** Achados de restrição ao enchimento das câmaras cardíacas e seu significado clínico^49^

Achado	Significado
**Colapso diastólico tardio do átrio direito**	Ocorre durante o relaxamento atrial (momento em que o volume do átrio direito [AD] é mínimo e a pressão pericárdica atinge seu pico), levando à invaginação da parede atrial para o interior da câmara. Tem maior valor quando persiste por mais de um terço do ciclo cardíaco. É um dos sinais mais precoces no tamponamento cardíaco. Apesar da alta sensibilidade, sua especificidade fica em torno de 82%, uma vez que colapsos breves do AD são observados em outras situações, como aumento da pressão intratorácica (o colapso sustentado do AD, por outro lado, permanece como um forte indicativo de tamponamento cardíaco).
**Colapso diastólico do ventrículo direito**	Geralmente ocorre durante a diástole precoce, quando o volume ventricular ainda é reduzido. Apresenta alta especificidade quando presente; entretanto, em determinadas situações clínicas - como nos casos de hipertrofia do ventrículo direito (VD) ou de elevação importante da pressão diastólica dessa câmara (por exemplo, em *cor pulmonale* aguda ou crônica) - o colapso das câmaras direitas pode não estar evidente, mesmo na presença de tamponamento. Dessa forma, embora tenha sensibilidade > 90%, esse sinal é menos sensível do que o colapso do AD para o diagnóstico de tamponamento cardíaco.
**Veia cava inferior (VCI) dilatada, com variação respiratória mínima ou ausente**	No tamponamento cardíaco, o AD sofre compressão externa (maior pressão intrapericárdica) no início da diástole, o que gera limita a capacidade de acomodar a pré-carga. Dessa forma, a VCI permanece dilatada, com variação respiratória mínima (diâmetro > 2,1 cm com redução inspiratória inferior a 50%). Esse sinal apresenta elevado valor preditivo negativo (ou seja, a ausência do sinal reduz substancialmente a probabilidade de tamponamento). Seu valor preditivo positivo é baixo porque a condição também pode ser observada em outras condições, como doenças pulmonares crônicas, insuficiência cardíaca, insuficiência tricúspide e outras cardiopatias. A visualização geralmente é obtida no plano sagital, abaixo do processo xifoide, e a mensuração do diâmetro é realizada aproximadamente 2–3 cm antes da junção da VCI com o AD, ao nível da confluência da veia hepática. Em alguns casos, pode-se utilizar o modo M para obter medidas específicas, posicionando o cursor nesse ponto anatômico.
**Swinging heart**	Movimento oscilante do coração. Presente em casos de derrames pericárdicos volumosos, muitas vezes associado à variação da amplitude do complexo QRS em batimentos alternados (alternância elétrica)

#### 8.1.2. Avaliação da Pleura e Pulmão na Unidade de Emergência

Entre as causas de dor torácica que podem ser identificadas com o POCUS, há situações relacionadas ao pulmão (pneumonia, embolia pulmonar) e à pleura (pneumotórax, derrame pleural). O POCUS pulmonar permite a visualização direta da linha pleural e a avaliação da presença do "sinal do deslizamento pulmonar" (*lung sliding*), ausente no pneumotórax. A ausência de linhas B e a presença do "ponto pulmonar" (*lung point*) também corroboram o diagnóstico.^49,172^ A rápida exclusão ou confirmação do pneumotórax é crucial para o manejo imediato.

A avaliação de achados sugestivos de TEP está descrita no tópico abaixo.

### 8.2. Avaliação do Paciente com Dispneia

A USP é uma das principais aplicações do POCUS na avaliação rápida, à beira do leito e em tempo real de pacientes com queixa de dispneia na unidade de emergência. Seu uso sempre deve ser associado à história clínica e ao exame físico completo e tem o potencial de acelerar o diagnóstico apropriado e, por consequência, o tratamento adequado.

#### 8.2.1. Congestão Pulmonar, Pleuropatias e Outros Diagnósticos Avaliados pela Ultrassonografia Pulmonar

A USP é uma ferramenta excelente para a avaliação rápida e não invasiva da congestão pulmonar. Ele se baseia na identificação de artefatos ultrassonográficos chamados linhas B, que são um forte indicativo de acúmulo de líquido nos septos interlobulares.

As linhas B, também chamadas de *"comet tails"* ou "linhas em cauda de cometa", são artefatos verticais, hiperecogênicos, longos e bem definidos que se originam da linha pleural e se estendem até o final da tela e acompanham o deslizamento pulmonar (Figura 13b). Elas se formam quando há espessamento dos septos interlobulares ou acúmulo de líquido, como na congestão pulmonar. A presença de três ou mais linhas B em um único espaço intercostal sugere congestão pulmonar importante. A quantidade e a distribuição das linhas B se correlacionam com a gravidade do quadro.^63^

Na Seção 3, são descritas as informações essenciais sobre a aplicação da USP na unidade de emergência, incluindo:

- Informações técnicas da USP;
- Achados mais frequentes e seus significados;
- O papel do POCUS no diagnóstico de congestão pulmonar, derrame pleural, síndromes intersticiais, consolidação pneumônica, atelectasia, pneumotórax e TEP.

Considerando que os diversos achados no TEP vão além da USP, esse diagnóstico será detalhado a seguir.

#### 8.2.2. Tromboembolismo Pulmonar

Assim como em outras causas potencialmente fatais de dor torácica, o diagnóstico precoce de TEP é essencial. Entretanto, trata-se de um diagnóstico desafiador, pois pode mimetizar diversas outras condições. Nesse sentido, o POCUS é uma ferramenta diagnóstica de grande utilidade à beira do leito, permitindo uma avaliação rápida e integrada de achados cardíacos, pulmonares e vasculares (Tabela 7).

**Tabela 7:** POCUS no auxílio ao diagnóstico de tromboembolismo pulmonar^131^

Achado	Significado	Observações
**Trombose venosa profunda (TVP) (** **Figura 19** **)**	Visualização de trombo não compressível em veias profundas (femoral, poplítea, etc.)	Achado altamente sugestivo de TEP se associado a sintomas respiratórios.
**Dilatação do VD** **(****Figura 6a e 6b****)**	Diâmetro do VD > VE (VD/VE > 1) em corte paraesternal ou apical	Sugere sobrecarga de pressão do VD (sinal de embolia maciça/submaciça).
**Sinal de McConnell**	Hipocinesia da porção basal e média da parede livre do VD com ápice preservado	Altamente sugestivo de embolismo pulmonar (especificidade ~94%).
**Septo achatado/aplainado (** **Figura 7** **)**	Desvio do septo interventricular para o VE em diástole	Indica sobrecarga pressórica do VD.
**Hipertensão pulmonar**	Estimativa de PSAP	Pode ser calculada a partir do pico de regurgitação tricúspide.
**Trombo em trânsito**	Visualização direta de trombo nas cavidades direitas	Específico, mas de baixa sensibilidade. Diagnóstico diferencial com massas emboligênicas, como tumores e vegetações.
**Trombo em artéria pulmonar**	Visualização direta de trombo no tronco da artéria pulmonar ou ramos (raro)	Específico, mas de baixa sensibilidade (mais comum em TEP central).
**Derrame pleural**	Líquido na cavidade pleural (geralmente pequeno a moderado)	Inespecífico, mas comum em TEP.
**Consolidações subpleurais triangulares (** **Figura 13k** **)**	Sugerem infarto pulmonar	Pacientes com TEP sem infarto pulmonar podem ter um padrão normal à USP

PSAP: pressão sistólica da artéria pulmonar; TEP: tromboembolismo pulmonar; USP: ultrassonografia pulmonar; VD: Ventrículo direito; VE: Ventrículo esquerdo

A combinação desses três locais principais (coração, pulmões e sistema venoso profundo dos membros inferiores) apresenta especificidade superior a 95%. Em pacientes hemodinamicamente instáveis, o POCUS cardíaco à beira do leito deve ser a primeira ferramenta empregada, dado que a ausência de sinais de sobrecarga aguda e insuficiência do VD podem excluir um TEP maciço como causa possível. Além disso, a avaliação fornecer informações valiosas sobre outras etiologias possíveis, como insuficiência do VE, pericardiopatias e status volêmico.

### 8.3. Avaliação do Paciente com Instabilidade Hemodinâmica e/ou Disfunção Orgânica

Em pacientes com instabilidade hemodinâmica e/ou disfunção orgânica, é fundamental assegurar uma pressão de perfusão adequada. Nesse contexto, a avaliação da responsividade à fluidoterapia (Figura 21) representa uma informação essencial para a definição da etiologia do choque e para a escolha da melhor intervenção de suporte hemodinâmico.

Por outro lado, pacientes com insuficiência do VD, hipertensão pulmonar ou sobrecarga hídrica são propensos a desenvolver congestão venosa sistêmica importante, o que leva a redução do gradiente arteriovenoso, elevação da pressão hidrostática capilar e edema intersticial. O resultado pode ser uma disfunção orgânica (especialmente renal) e, por consequência, uma maior retenção hídrica, o que cria um ciclo vicioso.^90–93^ A Seção 6 apresenta uma descrição completa do papel do POCUS no choque.

Dado que a insuficiência renal aguda (IRA) é uma das primeiras manifestações de disfunção orgânica em pacientes hipo e hipervolêmicos, o POCUS é útil na avaliação de pacientes com IRA (Figura 17), pois avaliações clínicas da volemia estão sujeitas a limitações importantes.^94–96^ O uso do POCUS à beira do leito representa uma ferramenta valiosa para a avaliação do estado volêmico e para a quantificação da congestão venosa sistêmica em serviços de emergência.

Na Seção 4, são descritas as principais informações sobre a aplicação do VExUS em unidades de emergência, incluindo:

- Evidências científicas (Tabela 5);
- Aspectos técnicos do VExUS para a análise da VCI e, quando necessário, dos demais componentes do escore (veia supra-hepática, veia porta e veia interlobar renal) (Figura 15).

É importante frisar que o VExUS não avalia a necessidade de reposição volêmica. Contudo, ele é um parâmetro importante na definição da tolerância à fluidoterapia, pois ajuda a identificar os casos em que a interrupção de reposição volêmica (ou a remoção de fluidos) tende a ser benéfica.

### 8.4. Avaliação do Paciente com Edema Periférico

Além da USP para avaliar congestão pulmonar e do VExUS para avaliar o estado volêmico e quantificar a congestão venosa, o POCUS pode ser útil na investigação de causas específicas de edema, como TVP.^131^

A Seção 5 apresenta as informações essenciais relacionadas à aplicação do POCUS na avaliação de TVP, incluindo:

- Evidências científicas;
- Aspectos técnicos (protocolos de compressão) (Figura 16).

## 9. POCUS na Parada Cardiorrespiratória

### 9.1. Introdução

O POCUS durante a parada cardiorrespiratória (POCUS-PCR), quando realizado por um operador capacitado, possibilita verificar a qualidade das compressões torácicas, diagnosticar prontamente causas tratáveis de parada cardíaca com ritmos não chocáveis (hipovolemia, pneumotórax hipertensivo, TEP e tamponamento cardíaco), monitorar intervenções e avaliar a resposta ao tratamento (Figura 27).^174^ O POCUS também oferece dados prognósticos relevantes sobre a chance de retorno da circulação espontânea e as perspectivas de sobrevida.^175^

**Figura 27:**
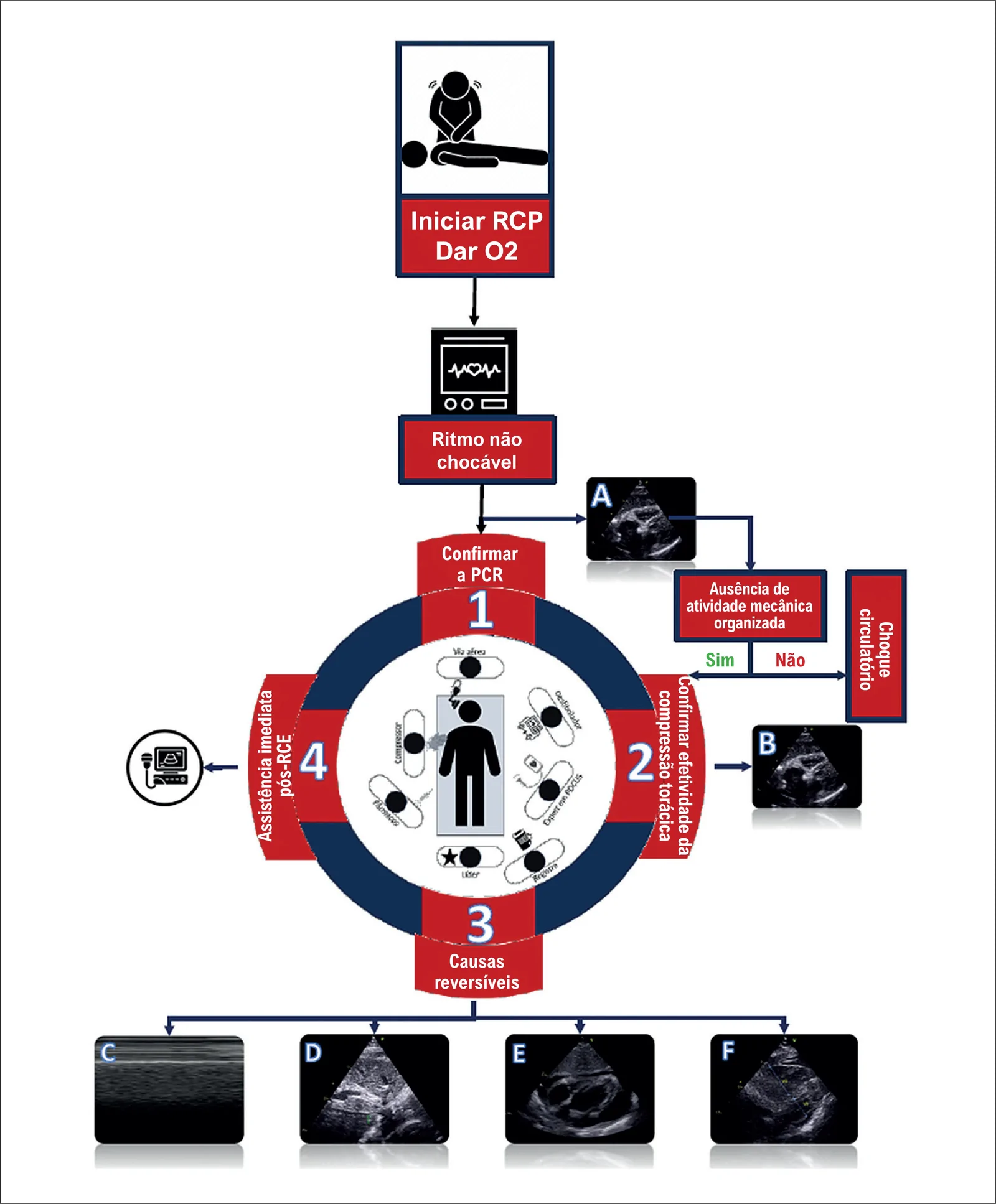
Utilidade do POCUS durante uma parada cardiorrespiratória (POCUS-PCR). A ilustração central representa a disposição da equipe sugerida neste posicionamento durante a realização do POCUS-PCR. A janela subcostal permite a confirmação rápida da PCR versus contração cardíaca, de ritmo chocável versus não chocável (painel 1) e da eficácia da compressão torácica (painel 2B). É possível identificar causas reversíveis, como pneumotórax hipertensivo (painel 3C), choque hipovolêmico (painel 3D), tamponamento cardíaco (painel 3E) e sinais de tromboembolismo pulmonar (painel 3F). Após o retorno do ritmo cardíaco espontâneo, é possível realizar uma avaliação direcionada para investigar outras causas (painel 4). RCP: ressuscitação cardiopulmonar; RCE: retorno da circulação espontânea.

O uso do POCUS durante a parada cardiorrespiratória (PCR) ainda é cercado de incertezas, diferentemente de outros contextos em que seu uso está mais consolidado. A principal preocupação é que o exame poderia atrasar a retomada das compressões cardíacas, fundamentais na ressuscitação cardiopulmonar, e que, até o momento, não demonstrou um impacto significativo no prognóstico dos pacientes. Essas incertezas se devem principalmente à falta de um protocolo universal que possa incorporado de forma definitiva ao suporte avançado de vida em cardiologia (ACLS).^176^

### 9.2. Aspectos Técnicos

A Figura 27 ilustra a organização da equipe durante o atendimento da PCR, destacando a posição do operador de POCUS à esquerda do paciente.

Recomenda-se que seja utilizado apenas o transdutor setorial (3,5-5 MHz), pois permite avaliar adequadamente o pulmão, coração e o abdome, dependendo do contexto quadro clínico. As imagens devem ser adquiridas durante os 10 segundos de pausa para checagem do ritmo e analisadas ao longo do ciclo de reanimação. Durante as compressões torácicas, podem ser adquiridas pela janela subcostal para avaliar a eficácia da massagem cardíaca.^177^ Embora existam diversos protocolos estabelecidos para POCUS-PCR, recomenda-se o CASA por ser o único que reduz o tempo das interrupções para checagem do ritmo, que consiste em avaliar a atividade cardíaca e possíveis causas, como TEP e tamponamento cardíaco.^178^ Durante a PCR, o operador de POCUS pode investigar o diagnóstico de pneumotórax e de hipovolemia.^178^

### 9.3. Execução do POCUS-PCR

Caso o ritmo identificado na PCR seja não desfibrilável, recomenda-se começar o POCUS-PCR pela janela subcostal. As imagens devem ser analisadas posteriormente, no início do ciclo seguinte, para não prejudicar a ressuscitação cardiopulmonar (RCP). Durante o intervalo designado para a verificação do pulso e análise do ritmo cardíaco, é possível examinar as outras janelas ecográficas, como a janela pulmonar para a avaliação do deslizamento pleural, a janela apical com visualização das quatro câmaras, a janela paraesternal de eixo curto e, por fim, o eixo longo paraesternal. Dependendo do cenário clínico, o protocolo FAST para pesquisa de líquido livre em cavidades pode ser incorporado ao POCUS-PCR, independentemente das manobras de RCP, e fornecer dados adicionais para o diagnóstico.^179^ O POCUS-PCR é fundamental para identificar as causas reversíveis de uma parada cardíaca e deve ser empregado em conjunto com a investigação sistemática de condições como hipovolemia, tamponamento cardíaco, pneumotórax hipertensivo e TEP (Figura 27, painel 3).^174^

### 9.4. Confirmação da Parada Cardiorrespiratória, Avaliação da Eficácia das Compressões Torácicas e da Função Prognóstica da Presença da Atividade Cardíaca na Atividade Elétrica sem Pulso

Embora a distinção entre ritmos chocáveis e não chocáveis dependa da eletrocardiografia, a US pode detectar uma falsa PCR porque permite o diagnóstico diferencial entre assistolia e fibrilação ventricular fina com contração demonstrável, achado esse presente em 10 a 35% dos pacientes com suposta assistolia.^174^

Outra função do POCUS-PCR é avaliar a eficácia das compressões, proporcionando observação direta e em tempo real da contração e relaxamento das câmaras cardíacas durante a massagem. Estudos mostram que a posição inadequada da mão durante as manobras de ressuscitação pode resultar na compressão da aorta ascendente, da raiz da aorta ou da VSVE, excluindo o próprio VE. O POCUS-PCR possibilita o ajuste da força aplicada e da posição das mãos.^174^

### 9.5. Diagnósticos de Causas Reversíveis pelo POCUS-PCR

#### 9.5.1. Tamponamento Cardíaco

O tamponamento cardíaco pode ocorrer devido tanto a eventos traumáticos quanto a causas não traumáticas. Mesmo pequenos volumes de líquido acumulados de forma rápida no espaço pericárdico influenciam a hemodinâmica cardiovascular (enchimento ventricular/débito cardíaco) e podem desencadear o quadro de tamponamento cardíaco.^174^

A diferenciação entre derrame pericárdico e pleural nem sempre é simples. Na janela paraesternal eixo longo, o derrame pericárdico aparece na região anterior à aorta descendente e acima da dobra posterior do pericárdio. O derrame pleural, por sua vez, se localiza atrás da aorta descendente e abaixo dessa dobra pericárdica (Figura 9).^21^

É importante salientar que identificar um derrame pericárdico não basta para diagnosticar o tamponamento cardíaco. E os sinais clássicos de tamponamento, como colapso sistólico do AD e colapso diastólico do VD, não são visíveis em pacientes já em PCR. A avaliação da VCI pode ajudar nessa definição quando esta se encontra dilatada, indicando uma elevação das pressões no AD.^174^

A ecocardiografia também desempenha um papel em guiar a pericardiocentese. Várias técnicas têm índice de sucesso próximo a 95% e de complicações em menos de 2% dos casos. Dá-se preferência à janela subcostal para orientar o trajeto da agulha durante o procedimento.^180^

#### 9.5.2. Tromboembolismo Pulmonar

O TEP pode ser identificado na ultrassonografia por evidências diretas ou indiretas. A evidência direta é a visualização do próprio trombo. Os sinais indiretos envolvem, por exemplo, a dilatação súbita do VD, que se torna maior do que o VE, com relação VD/VE de pelo menos 1:1. Além disso, a configuração em forma de "D" do VD (paraesternal de eixo curto) também é indicativa de dilatação do VD (Figura 7).

Diante da suspeita de TEP, o protocolo de US focada para a investigação de TVP pode ser introduzido sem prejudicar a massagem cardíaca (Figura 17).^174^

#### 9.5.3. Pneumotórax Hipertensivo

O pneumotórax hipertensivo, uma das principais causas de PCR, pode decorrer de doenças pulmonares preexistentes, ser uma complicação da ventilação com pressão positiva e ocorrer após acidente de punção venosa profunda.^181^

Os sinais de pneumotórax podem ser observados pela USP na região do 4° ao 5° espaço intercostal ao longo da linha médio-clavicular. Os sinais incluem a ausência de deslizamento pleural, a presença do sinal da estratosfera (ou código de barras; Figura 27, painel 3C) e a ausência de movimento pulmonar ou pulso pulmonar. O sinal mais específico é o ponto de pulmonar, que, quando identificado, tem especificidade muito alta para a presença de pneumotórax.^174^

#### 9.5.4. Hipovolemia

Durante uma PCR, a ultrassonografia pode revelar o colabamento completo dos ventrículos e o colapso da VCI. Esses sinais, ao lado da evolução clínica do paciente e outros dados, podem sugerir hipovolemia como causa subjacente da PCR (Figura 25, painel 3D). Em situações de trauma, é essencial que o médico identifique imediatamente a presença de sangue na cavidade abdominal ou no tórax, uma possível causa de choque hipovolêmico.^21^

## 10. Procedimentos Guiados pela Ultrassonografia

### 10.1. Acesso Vascular Guiado pelo Ultrassom

A prática da punção guiada por ultrassonografia tem se consolidado na maioria dos centros médicos, sobretudo por sua superioridade comprovada em termos de segurança. A eficácia do procedimento depende diretamente do domínio da anatomia regional pelo operador, da sua experiência clínica e da qualidade do equipamento.^182,183^

A ocorrência de complicações é multifatorial e envolve aspectos como a experiência e habilidade do profissional, o biotipo e o estado clínico do paciente (incluindo nível de colaboração, agitação e status volêmico) e a complexidade anatômica da região a ser puncionada. Além disso, o número de tentativas de punção está diretamente associado a um maior risco de eventos adversos.

Historicamente, esses procedimentos eram realizados "às cegas", com base apenas em referências anatômicas superficiais. Atualmente, há fortes evidências de que o posicionamento de cateteres guiado por US é mais seguro, transformando-a em uma ferramenta extremamente útil para a punção e na monitorizarão de procedimentos intervencionistas.^184^

#### 10.1.1. Técnica de Punção

Seldinger descreveu a técnica de punção percutânea para a inserção de cateteres vasculares. Ela pode ser resumida como "cateter sobre fio-guia": após a punção percutânea do vaso, introduz-se o fio-guia pela agulha. O fio-guia serve como um trilho e guia o cateter para dentro do vaso da forma mais eficaz e menos traumática possível.

Basicamente, existem duas técnicas de punção guiada por ultrassonografia:

1. **Técnica estática**: Consiste no mapeamento ultrassonográfico prévio da anatomia vascular e na marcação do local ideal de punção na pele. Uma das principais vantagens é que não requer manutenção de um campo estéril durante o exame, sendo útil em situações de menor complexidade;
2. **Técnica dinâmica ou em "tempo real"**: Permite a visualização contínua em tempo real da estrutura vascular (veia ou artéria), da progressão da agulha através dos tecidos e de sua entrada no lúmen do vaso. Recomenda-se o uso dessa técnica sempre que possível, pois aumenta a precisão do procedimento, reduz o número de tentativas e está associada a uma menor taxa de complicações, especialmente em pacientes com anatomia complexa ou acesso venoso difícil.

#### 10.1.2. Passo a Passo da Punção (Utilizar Material Estéril)

1. Posicione o paciente em decúbito dorsal, preferencialmente em posição de Trendelenburg reverso, para aumentar o calibre da veia e facilitar a punção. A manobra de Valsalva também ser útil;
2. Avalie o vaso a ser puncionado e as estruturas vizinhas (vasculares ou não); É importante saber diferenciar entre artérias e veias, o que pode ser feito com base nas características ultrassonográficas (ver Tabela 8);
3. Realize ampla assepsia do local com clorexidina a 2%;
4. Posicione os campos estéreis;
5. Realize anestesia local com xilocaína a 2% ("botão" anestésico com agulha de insulina);
6. Encape o transdutor com material estéril, colocando gel no interior da capa de modo que ele fique em contato com a face do transdutor. Fixe a capa com bandas elásticas para facilitar o manuseio do transdutor;
7. Após girar o rosto do paciente 45° para o lado contralateral, visualize o vaso em corte transversal ou longitudinal e meça a distância entre a parede anterior da veia e a pele (no ponto de entrada da agulha). Tome cuidado para não pressionar demais o transdutor contra a pele, pois isso pode comprimir a veia.

**Tabela 8:** Diferenciação ultrassonográfica entre artérias e veias

Artérias	Veias
Paredes mais grossas	Paredes mais finas
Não compressíveis	Compressíveis
Pulsáteis	Não pulsáteis
Direção do fluxo ao Doppler colorido	Direção do fluxo ao Doppler colorido

#### 10.1.3. Punção em Corte Transversal (Eixo Curto)

Nessa abordagem, a veia é visualizada em uma seção transversal e posicionada no centro da tela do ultrassom. A agulha deve ser introduzida exatamente no plano médio do transdutor, perpendicular à pele (Figura 28). Esse método é considerado mais acessível do ponto de vista técnico, pois permite a visualização simultânea da veia e da artéria no mesmo campo de imagem, o que favorece a diferenciação entre estruturas vasculares e evita punções inadvertidas.

**Figura 28:**
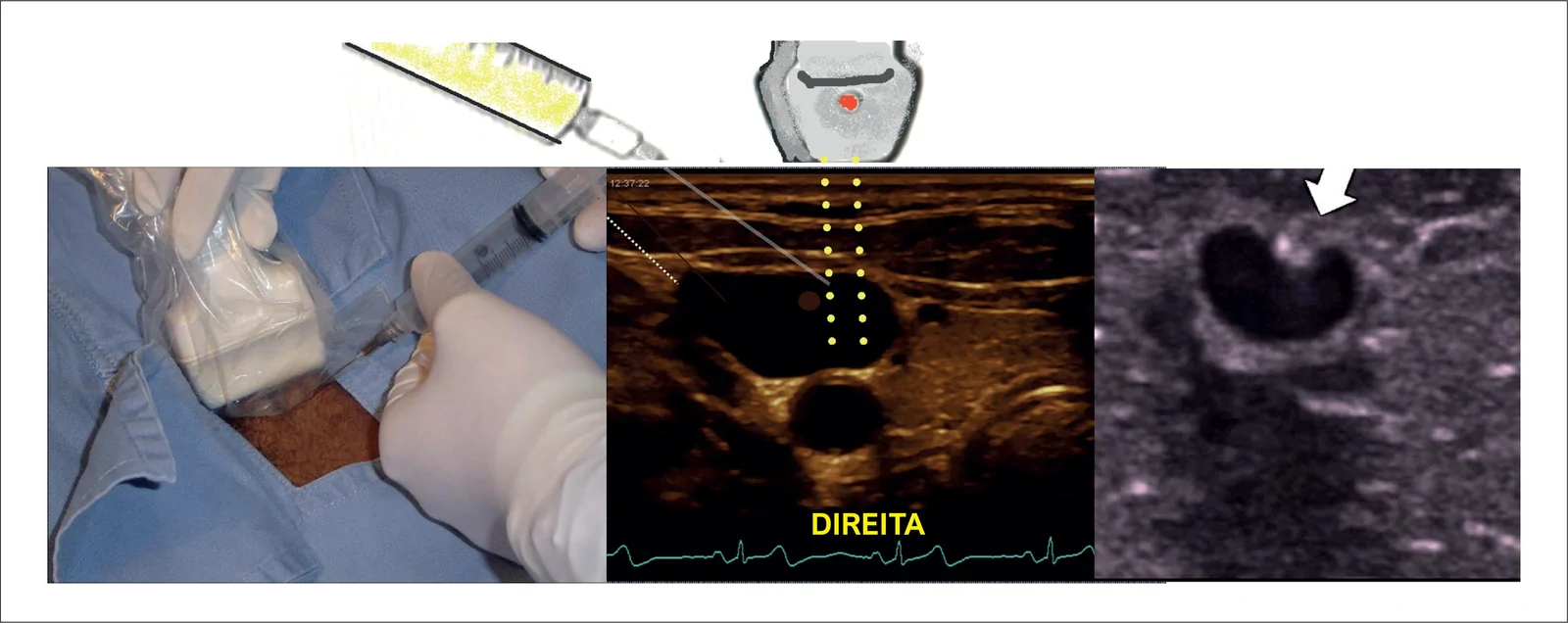
Posicionamento do transdutor e da agulha na técnica de punção transversal. A agulha é visualizada somente como um ponto ecogênico quando cruza o feixe ultrassonográfico. A movimentação dos tecidos ao redor da agulha ajuda a identificar a sua posição, enquanto a indentação da parede da veia confirma o posicionamento correto.

No entanto, sua principal limitação é a impossibilidade de visualizar continuamente a ponta da agulha. Como cruza obliquamente o feixe ultrassonográfico, a agulha aparece como um ponto hiperecogênico (brilhante) intermitente, acompanhado de um artefato ecogênico distal. Esse artefato pode induzir interpretações equivocadas, sendo confundido com a ponta da agulha.

A posição da agulha deve ser inferida pela movimentação dos tecidos adjacentes durante a inserção, que pode ser facilitada por movimentos suaves e controlados de avanço e recuo. Como a visualização direta da ponta da agulha é limitada, é fundamental conhecer previamente a profundidade da parede anterior da veia - medida no modo B da pele até o lúmen vascular. Essa distância serve como referência para evitar avanços excessivos, que aumentam o risco de complicações, como punções da parede posterior, lesões arteriais e hematomas.

A confirmação do posicionamento correto da agulha dentro do vaso ocorre pela observação da compressão da parede anterior e, posteriormente, o retorno de sangue pela seringa conectada. Essa etapa é essencial antes da introdução do fio-guia.

#### 10.1.4. Punção em Corte Longitudinal (Eixo Longo)

A abordagem longitudinal oferece ao operador uma visualização contínua da trajetória da agulha em tempo real, desde a entrada nos tecidos até a penetração na luz do vaso. Isso proporciona um controle mais refinado do procedimento, com mais segurança e precisão (Figura 29).

**Figura 29:**
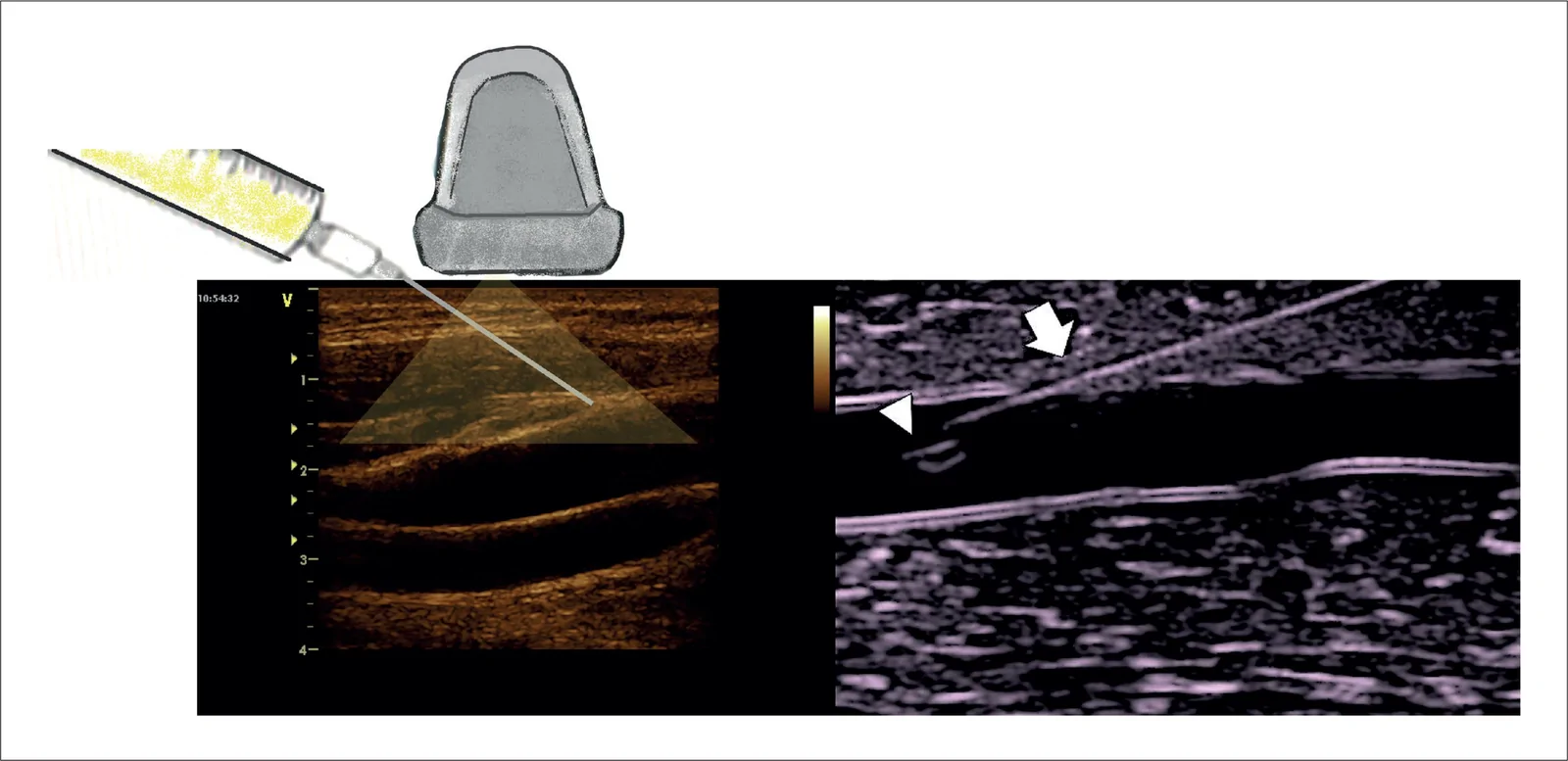
Posicionamento do transdutor e posição da agulha na técnica de punção longitudinal. A agulha pode ser visualizada em toda sua extensão quando completamente dentro do feixe de ultrassom. A ponta da agulha pode ser reconhecida pelo seu aspecto em bisel.

Ao contrário da punção em eixo curto, a técnica em corte longitudinal permite a visualização direta da ponta da agulha ao longo de todo o trajeto, inclusive no momento exato em que entra no vaso. Isso elimina a dependência do retorno sanguíneo para confirmar o posicionamento correto, pois a agulha pode ser monitorada visualmente durante toda a punção. Por consequência, o fio-guia pode ser introduzido sob visão ultrassonográfica direta, o que reduz o risco de complicações, como lesões da parede posterior do vaso e inserções inadequadas.

Apesar dessas vantagens, a punção em corte longitudinal é mais desafiadora do ponto de vista técnico, especialmente para iniciantes. Isso ocorre porque é necessário o plano do feixe de ultrassom precisamente com o eixo da agulha, o que exige destreza manual e familiaridade com a imagem ultrassonográfica. Pequenos desvios no alinhamento podem fazer com que a agulha desapareça do campo de visão, o que torna o procedimento frustrante em mãos menos experientes.

#### 10.1.5. Locais de Punção Guiada por Ultrassonografia

Diversas veias centrais podem ser acessadas com auxílio da ultrassonografia. A escolha do local ideal depende de fatores anatômicos e clínicos e da experiência do operador. As principais abordagens são descritas a seguir:

##### a. Veia jugular interna

Com frequência, a veia jugular interna é considerada o local de escolha para punção guiada por ultrassonografia, principalmente devido à sua acessibilidade e à visibilidade favorável com o transdutor. O ponto de punção mais utilizado se situa entre as duas cabeças do músculo esternocleidomastoideo, embora qualquer ponto onde com menor espessura de tecido mole entre a pele e o vaso possa ser adequado. A ultrassonografia permite a visualização clara da relação entre a veia e a artéria carótida, o que reduz substancialmente o risco de punção arterial inadvertida.

##### b. Veia femoral

A veia femoral é uma alternativa segura, especialmente em situações de emergência. Para localizá-la, o transdutor deve ser posicionado imediatamente abaixo do ligamento inguinal. Na maioria dos pacientes, a veia se encontra medialmente à artéria femoral, mas pode haver sobreposição (*overlap*) entre os dois vasos em até 50% dos casos. Quando a artéria está completamente sobre a veia, recomenda-se avaliar o lado contralateral para minimizar o risco de complicações.

##### c. Veia subclávia

O acesso ecoguiado à veia subclávia tem se mostrado eficaz, com maior taxa de sucesso e menor incidência de complicações como punção arterial e hematomas. Além disso, a abordagem está associada a uma menor taxa de infecção e bacteremia, bem como menor variabilidade anatômica em comparação com a veia jugular interna.^185^

Entretanto, o trajeto da veia subclávia sob a clavícula impõe desafios técnicos. A insonação do vaso pode ser difícil para profissionais pouco experientes, especialmente devido a variações anatômicas individuais e à interferência óssea.

Para a punção infraclavicular ecoguiada, recomenda-se:

- Iniciar com a avaliação da região infraclavicular em corte transversal;
- Identificar a veia e a artéria no triângulo clavipeitoral;
- Centralizar a imagem da veia na tela;
- Rotacionar o transdutor para obter a visualização longitudinal (eixo longo) da veia.

Essa orientação permite:

- Visualizar a ponta da agulha em tempo real;
- Evitar a punção da parede posterior do vaso;
- Em alguns casos, identificar a pleura parietal, aumentando a segurança do procedimento.

Por esses motivos, a punção em eixo curto (corte transversal) não é recomendada nessa região, especialmente devido ao risco de pneumotórax.^183^

Embora meta-análises não tenham demonstrado diferenças significativas na taxa de sucesso da punção da veia subclávia guiada por US em comparação com a técnica baseada em referências anatômicas (insucesso de 9% vs. 13%, respectivamente), Caballero et al. recomendam o uso da ultrassonografia em situações com maior risco de complicação ou dificuldade técnica.^186^ Mesmo após a falha da técnica baseada em marcos anatômicos, a punção guiada por ultrassonografia demonstra maior probabilidade de sucesso.

#### 10.1.6. Outras Punções Vasculares Guiadas por Ultrassonografia

A ultrassonografia também pode guiar a punção das veias cefálica ou basílica no braço, locais comumente utilizados para a inserção de cateteres do tipo PICC.

As punções arteriais são utilizadas para cateterismos diagnósticos e terapêuticos e para medida da pressão arterial média (PAM). Quando guiados por ultrassonografia, esses procedimentos obedecem às mesmas regras aplicadas às veias, sendo a técnica longitudinal utilizada com mais frequência.

### 10.2. Pericardiocentese Guiada por Ultrassom

O acúmulo anormal de líquido entre as membranas pericárdicas é uma condição cardíaca comum, com diversas etiologias possíveis. A punção pericárdica pode ser realizada de forma eletiva, para investigação diagnóstica do derrame pericárdico, ou de forma emergencial, para alívio imediato do tamponamento cardíaco. A ecocardiografia é o método de escolha para o diagnóstico, localização, quantificação e avaliação dos efeitos hemodinâmicos.^187^

Tradicionalmente, a pericardiocentese era realizada pela via subxifoide, "às cegas", com morbidade em torno de 20% e mortalidade de 6%. Quando guiada por US e executada por profissionais experientes que receberam treinamento adequado, apresenta taxa de eventos maiores de 0,3 a 3,9% e de eventos menores de 0,4 a 20%.^189^ As seções a seguir apresentam um guia passo a passo para a técnica de punção guiada por US recomendada por este posicionamento.

#### 10.2.1. Técnica de Punção Guiada por Ultrassonografia

- Normalmente, utiliza-se o transdutor setorial de baixa frequência, usado na ecocardiografia. Quando é necessário maior detalhamento das estruturas, principalmente nas janelas paraesternais (avaliação do curso das artérias torácicas internas) ou em janelas muito lateralizadas (proximidade à pleura), utiliza-se o transdutor linear de alta frequência;
- A Figura 25 ilustra as janelas ecocardiográficas utilizadas e a frequência com que foram empregadas em uma série da Clínica Mayo;^188^
- Deve se identificar onde o acúmulo de líquido é maior. Apesar de ser circunferencial na maioria das vezes, a distribuição do líquido acumulado em torno do coração não é uniforme;
- Deve-se localizar o ponto em que a distância entre a pele e o espaço pericárdico é mais curta e identificar estruturas adjacentes ou no trajeto da agulha (Figura 26);
- O paciente deve ser posicionado em decúbito dorsal, com cabeceira elevada entre 30° e 60°;
- Deve-se realizar anestesia local, com sedação leve, utilizando o kit de canulação venosa central;
- Na punção, a agulha deve ser introduzida no local previamente marcado e sempre pelo bordo superior da costela (para evitar o risco de lesão dos vasos intercostais). Nas janelas apicais e subcostal, deve-se verificar a possível interposição de tecido hepático;
- Após alcançar o espaço pericárdico (drenagem de líquido ou sangue), deve-se introduzir o cateter usando a técnica de Seldinger e confirmar seu posicionamento por meio da injeção de 5 mL de solução salina agitada. Em seguida, deve-se fixar o cateter e o curativo estéril conforme a rotina padrão.

#### 10.2.2. Contraindicações

Embora não existam contraindicações absolutas à pericardiocentese, algumas situações exigem cautela. Quando o derrame pericárdico está associado à dissecção aguda da aorta ou à ruptura da parede ventricular esquerda, como pode ocorrer no IAM ou em traumas torácicos, a drenagem pericárdica apenas deve ser considerada na ausência de acesso imediato à cirurgia cardíaca. Nesses casos, a punção deve ser realizada de maneira lenta e controlada, apenas o suficiente para estabilizar temporariamente a hemodinâmica do paciente até a intervenção cirúrgica.

Outra contraindicação relativa é a trombocitopenia importante, especialmente quando a contagem plaquetária é inferior a 50.000/mm³, dado o maior risco de sangramento.^189^

#### 10.2.3. Complicações

Embora seja um procedimento potencialmente salvador em situações de tamponamento cardíaco, a pericardiocentese à beira do leito não está isenta de riscos. A realização segura exige conhecimento anatômico preciso, técnica adequada e uso preferencial da orientação ecocardiográfica. As complicações durante o procedimento variam em gravidade, desde efeitos vagais autolimitados até lesões cardíacas ou vasculares graves. A Tabela 9 resume as possíveis complicações associadas ao procedimento.

**Tabela 9:** Possíveis complicações durante a punção pericárdica

**Maiores**	Lesão das câmaras cardíacas Laceração das artérias coronárias, intercostais ou torácica interna Punção visceral Pneumotórax Arritmias Síndrome da descompressão pericárdica
**Menores**	Hipotensão e bradicardia vasovagal Arritmias supraventriculares Fístula pleuropericárdica

### 10.3. Avaliação do Estômago Cheio

A ecocardiografia transesofágica é realizada sob sedação e, para a segurança do procedimento, é essencial avaliar o estômago cheio para evitar o risco broncoaspiração e suas consequências. Na prática, isso se limita a perguntar ao paciente sobre o tempo de jejum em horas. Essa avaliação subjetiva pode criar um risco de broncoaspiração, principalmente em condições que cursam com gastroparesia (gravidez, diabetes, obesidade, instabilidade hemodinâmica, etc.). Neste posicionamento, adota-se a recomendação da Sociedade Americana de Anestesiologia, que preconiza os tempos de jejum a seguir^190^ (Tabela 10).

**Tabela 10:** Recomendação dos tempos de jejum de acordo com a última refeição^190^

Dieta	Tempo jejum
Líquida sem resíduo	2 horas
Leite materno	4 horas
Líquida com resíduo e fórmulas infantis	6 horas
Alimentos leves e baixo volume	6 horas
Ingestão habitual de alimentos gordurosos	8 horas ou mais

O POCUS gástrico permite avaliar de forma simples e direta a quantidade e a qualidade do resíduo gástrico antes do procedimento, aumentando a sua segurança.

#### 10.3.1. Técnica de Avaliação

Em geral, a avaliação do estômago por POCUS é realizada com o paciente em posição supina ou em decúbito lateral direito. Utiliza-se um transdutor curvilíneo de baixa frequência (2-5 MHz) para obter imagens da região epigástrica e do antro gástrico. A sonda é posicionada longitudinalmente no plano sagital, logo abaixo do apêndice xifoide e orientada para a esquerda do paciente. A identificação do antro gástrico é essencial, pois é o local mais sensível para detectar a presença de conteúdo gástrico (Figura 30a).^191^

**Figura 30:**
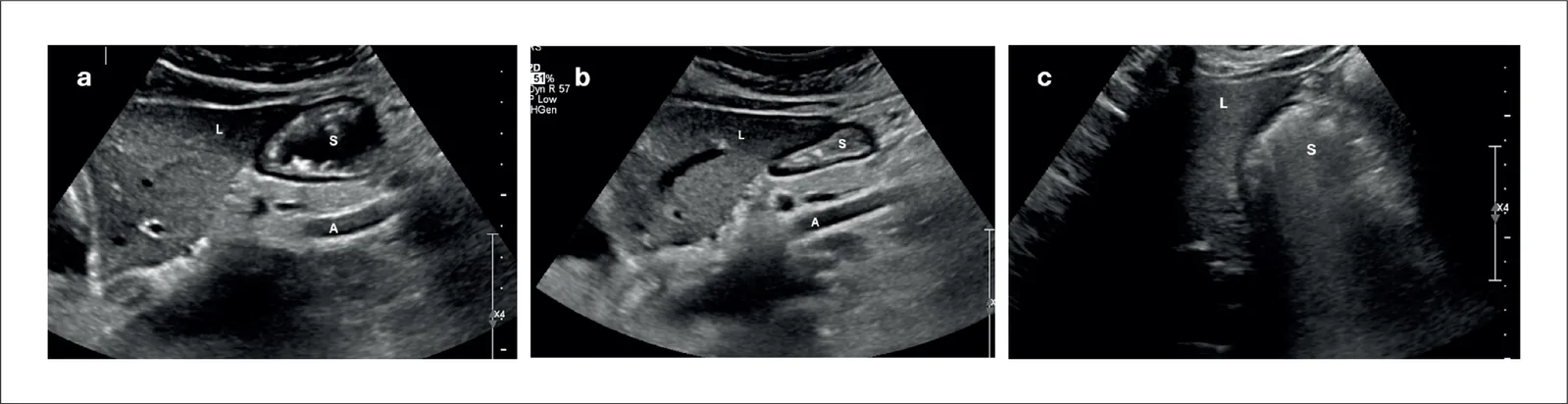
a. Antro gástrico com conteúdo anecoico, indicando estômago cheio (S) com conteúdo líquido. A: aorta; L: fígado b Estômago vazio com antro gástrico colapsado (S) c. Estômago cheio com conteúdo sólido caracterizado por áreas hiperecogênicas brancas (S).

#### 10.3.2. Achados Sonográficos

- **Estômago vazio:** Um estômago vazio geralmente apresenta o antro gástrico colapsado, com paredes finas e sem líquido ou conteúdo sólido discernível. A mucosa gástrica pode estar visível como uma linha hiperecogênica, enquanto o lúmen do estômago aparece praticamente sem conteúdo (Figura 30b);
- **Estômago com conteúdo líquido:** Quando o estômago está cheio de líquido, o antro gástrico se expande e apresenta uma aparência anecoica (preta) à ultrassonografia. Essa característica se deve à presença de líquido que não reflete o som, o que cria uma área escura na imagem. Em alguns casos, é possível observar movimentos peristálticos (Figura 30a);
- **Estômago com conteúdo sólido:** Um estômago com conteúdo sólido ou espesso apresenta uma aparência mais complexa à ultrassonografia. O antro gástrico pode ter aspecto heterogêneo, com áreas hiperecogênicas (brancas) que indicam a presença de partículas sólidas misturadas ao líquido. O padrão é típico após a ingestão de refeições sólidas ou alimentos de difícil digestão (Figura 30c).

A quantificação do conteúdo gástrico pelo POCUS emerge como ferramenta útil agregando segurança ao procedimento notadamente em cenários onde o a história clínica e o tempo de jejum não podem ser bem definidos. Outra possível vantagem é antecipar o procedimento uma vez que os achados ultrassonográficos indiquem um estômago vazio abordagem essa ainda sem validação segura.

## 11. Considerações Finais sobre este Posicionamento

### 11.1. Realidade Atual e Perspectivas Futuras

O uso do POCUS representa uma extensão natural do exame físico moderno, oferecendo informações em tempo real que impactam diretamente a tomada de decisões clínicas. Sua aplicação estruturada permite a avaliação rápida e eficaz de condições críticas em ambientes de emergência, terapia intensiva e até mesmo no âmbito ambulatorial. No entanto, como vimos na Seção 1, seu uso seguro e eficiente depende da regulamentação legal, do treinamento padronizado, da supervisão adequada e da atualização constante das habilidades técnicas e interpretativas.

A incorporação do POCUS aos fluxos assistenciais deve ser acompanhada de programas de capacitação, certificação de competências e estratégias de monitoramento contínuo da qualidade. Dessa forma, o método pode concretizar seu potencial como ferramenta diagnóstica complementar, promovendo cuidados mais precisos, ágeis e centrados no paciente.

### 11.2. Contextualização do POCUS Cardiovascular no Âmbito da Saúde Pública no Brasil

A incorporação do POCUS cardiovascular no SUS ocorre em paralelo à expansão da telessaúde, especialmente após a consolidação do Programa Telessaúde Brasil Redes^192^ e da Ação Estratégica SUS Digital,^193^ que busca reduzir as desigualdades de acesso e apoiar a qualificação da atenção em saúde, embora ainda existam importantes assimetrias regionais de infraestrutura e conectividade.^192,193^ A adoção do POCUS enfrenta barreiras como disponibilidade limitada de equipamentos, capacitação irregular, ausência de fluxos assistenciais padronizados e desafios logísticos relativos à manutenção, ao suporte técnico e à integração com sistemas de informação. Esses aspectos são discutidos em revisões nacionais sobre o tema.^13,194^ Equipamentos portáteis de ultrassonografia, como dispositivos *handheld*, apresentam custo internacional de USD 4.000–6.000, conforme catálogos de fabricantes,^195,196^ acrescido de despesas com assinaturas anuais de software e serviços. Como oportunidades de financiamento, destacam-se os programas federais vinculados ao SUS Digital,^192,193^ além de linhas de apoio à inovação da FINEP, como o Inova Saúde e o Apoio Direto à Inovação,^197,198^ além de editais de fomento estaduais, como os da FAPERJ,^199^ voltados à aquisição de equipamentos, infraestrutura tecnológica e pesquisa aplicada. Esses fatores sugerem um cenário futuro desafiador, mas com boas perspectivas, para a aplicação dessa metodologia em larga escala, não somente no âmbito da saúde privada, mas também no da saúde pública.

## Data Availability

os conteúdos subjacentes ao texto do Posicionamento estão contidos no manuscrito.

## Lista de abreviaturas e siglas

+LR: Positive likelihood ratio
A4C: Apical 4 câmaras
ACLS: Suporte avançado de vida em cardiologia
AD: Átrio direito
angio-TC: Angiotomografia
AP: Artéria pulmonar
ASCE: Artéria subclávia esquerda
AUC: Area under the curve
BIA: Balão intra-aórtico
BLUE: *Bedside Lung Ultrasound in Emergency*
CATE: Cateterismo cardíaco
CC: Choque cardiogênico
CHEST: *Critical Care Ultrasonography Certificate of Completion Certificate of Completion Program - American College of Chest Physicians*
DAVP: Dispositivo de assistência ventricular percutânea
DC: Débito cardíaco
DHE: Distúrbio hidroeletrolítico
DICOM: *Digital Imaging and Communications in Medicine*
DPer: Derrame Pericárdico
DPOC: Doença pulmonar obstrutiva crônica
DS: Débito sistólico
ECG: Eletrocardiograma
ECMO: Oxigenação por membrana extracorpórea
ECMO-VA: Oxigenação por membrana extracorpórea venoarterial
ECMO-VV: Oxigenação por membrana extracorpórea venovenosa
EPSS: *E-point septal separation*
ESC: Sociedade Europeia de Cardiologia
Esp ou Sp: Especificidade
ETE: Ecocardiografia transesofágica
ETT: Ecocardiografia transtorácica
FAC: Alteração fracionária da área
FC: Frequência cardíaca
FEVE: Fração de ejeção do ventrículo esquerdo
FR: Fluido-responsividade
FS: Encurtamento fracional
FT: Fluido-tolerância
HR: *Hazard ratio*
IA: Inteligência artificial
IAM: Infarto agudo do miocárdio
IC: Insuficiência cardíaca
IC95%: Intervalo de confiança de 95%
ICFEp: Insuficiência cardíaca com fração de ejeção preservada
ICFEr: Insuficiência cardíaca com fração de ejeção reduzida
IRA: Insuficiência renal aguda
IT: Insuficiência tricúspide
LRA: Lesão renal aguda
MAPSE: *Mitral annular plane systolic excursion*
OR: *Odds ratio*
OSAUS: *Objective Structured Assessment of Ultrasound Skills*
PAD: Pressão atrial direita
PAE: Pressão atrial esquerda
PAM: Pressão arterial média
PAP: Pressão da artéria pulmonar
PCR: Parada cardiorrespiratória
PLAPS: *Posterolateral alveolar and/or pleural syndrome*
PLAX: Paraesternal eixo longo
PLR: *Passive leg raising*
POCUS: *Point of care ultrasound*
POCUS-PCR: POCUS durante a parada cardiorrespiratória
PSAX: Paraesternal eixo curto
PSVD: Pressão sistólica do ventrículo direito
PVC: Pressão venosa central
RA: Regurgitação aórtica
RCP: Ressuscitação cardiopulmonar
SCA: Síndrome coronariana aguda
SCASSST: Síndrome coronariana aguda sem supradesnível do segmento ST
SCM: Suporte circulatório mecânico
SCMCP: Suporte circulatório mecânico de curto prazo
SDRA: Síndrome do desconforto respiratório agudo
Sen ou Sn: Sensibilidade
SIA: Septo interatrial
SIV: Septo interventricular
SM: Suporte mecânico
SUS: Sistema Único de Saúde
TAP: Tempo de aceleração do fluxo pulmonar
TAPSE: Excursão sistólica do anel tricúspide lateral
TEP: Tromboembolismo pulmonar
TOEF: Teste de oclusão expiratória final
TOIF: Teste de oclusão inspiratória final
TVI: Integral velocidade-tempo
TVP: Trombose venosa profunda
US: Ultrassonografia
USC: Ultrassonografia de compressão
USP: Ultrassonografia pulmonar
USV: Ultrassonografia vascular
UTI: Unidade de terapia intensiva
VAo: Válvula aórtica
VCI: Veia cava inferior
VCS: Veia cava superior
VD: Ventrículo Direito
VE: Ventrículo Esquerdo
VExUS: *Venous excess ultrasound*
VM: Válvula mitral
VPN: Valor preditivo negativo
VPP: Valor preditivo positivo
VS: Volume sistólico
VSF: Volume sistólico final
VSVE: Via de saída do ventrículo esquerdo
VT: Válvula tricúspide

## Suplemento

Suplemento 1

Suplemento 2
