# Supplementary material for: Posicionamento sobre o Uso do Ultrassom "Point of Care" (POCUS) na Medicina Cardiovascular – 2026
Source: Arq Bras Cardiol. 2026 May 4;123(4):e20260222. [Article in Portuguese] doi: 10.36660/abc.20260222 (PMC13671151; doi:10.36660/abc.20260222)
Supplement: Supplementary file 1 [file 0066-782x-abc-123-4-e20260222-Suppl01-en.pdf]

Supplement 1

Checklist for internal evaluation and certification of CV POCUS training and for granting institutional clinical privileges.

Name: ________ **Date:** / /

Number of examinations: 30+ ☐ 50+ ☐ 70+ ☐

Certified experience by: ________

1. **Preparation for the examination**
   - Approaches the patient appropriately, correctly identifies the patient, and demonstrates an adequate and professional attitude: 0☐ 1☐ 2☐ 3☐
   - Demonstrates knowledge of the basic indications: 0☐ 1☐ 2☐ 3☐
2. **Examination**
   - Applies ultrasound knowledge to adjust the equipment and select the most appropriate transducer: 0☐ 1☐ 2☐ 3☐
   - Image optimization: 0☐ 1☐ 2☐ 3☐
   - Sequential evaluation of windows and structures (systematic examination):
     - *Cardiac (parasternal, subcostal, and apical):* 0☐ 1☐ 2☐ 3☐
     - *Lung (pneumothorax, B-lines):* 0☐ 1☐ 2☐ 3☐
     - *Systemic veins and femoral and popliteal veins:* 0☐ 1☐ 2☐ 3☐
   - Image interpretation (based on binary responses of YES or NO [Level 2], or with severity quantification when applicable [Level 3])
     - *Is there LV enlargement?* 0☐ 1☐ 2☐ 3☐
     - *Is there RV enlargement?* 0☐ 1☐ 2☐ 3☐
     - *Is there pulmonary congestion (B-lines)?* 0☐ 1☐ 2☐ 3☐
     - *Is there systemic venous congestion (VExUS score)?* 0☐ 1☐ 2☐ 3☐
     - *Is there deep vein thrombosis?* 0☐ 1☐ 2☐ 3☐
     - *Is there a change in global LV systolic function?* 0☐ 1☐ 2☐ 3☐
     - *Is there a change in global RV systolic function?* 0☐ 1☐ 2☐ 3☐
     - *Is there pericardial effusion?* 0☐ 1☐ 2☐ 3☐
     - *Is there inferior vena cava plethora?* 0☐ 1☐ 2☐ 3☐
     - *Are there signs of hypovolemia?* 0☐ 1☐ 2☐ 3☐
3. **Post-examination**
   - Critical and appropriate interpretation of results: 0☐ 1☐ 2☐ 3☐
   - Results correctly integrated into clinical decision-making: 0☐ 1☐ 2☐ 3☐
   - Documentation of imaging findings and clinical outcome based on cardiac ultrasound results: 0☐ 1☐ 2☐ 3☐
   - Recognizes when the examination is inconclusive: 0☐ 1☐ 2☐ 3☐
4. **Video interpretation**
   - Multiple-choice questions: 0☐ 1☐ 2☐ 3☐
   - Open-ended questions: 0☐ 1☐ 2☐ 3☐

Form adapted from the "Objective Structured Assessment of Ultrasound Skills (OSAUS)" (Tolsgaard MG et al.[19]) and the "Point-of-Care Ultrasound Competence Entrustment Scale" (Mani N et al.[20]). If the trainee does not achieve 70% of the score required for the desired level, according to the AcQUIRE protocol, additional training and a new assessment must be performed. If the trainee is approved, the certification must be validated by the Training Coordinator in conjunction with the Education Committee and the Hospital Safety Unit, in order to grant institutional privileges to perform POCUS. However, this certification does not define full professional competency and does not qualify the individual to serve as a trainer. Certification of competency should be granted by the relevant specialty societies, which remains a future challenge. The privileges granted should be restricted to the institution or service where the professional practices. CV: cardiovascular; LV: left ventricle; POCUS: point-of-care ultrasound; RV: right ventricle; VExUS: Venous Excess Ultrasound Score.
