# Supplementary material for: Posicionamento sobre o Uso do Ultrassom "Point of Care" (POCUS) na Medicina Cardiovascular – 2026
Source: Arq Bras Cardiol. 2026 May 4;123(4):e20260222. [Article in Portuguese] doi: 10.36660/abc.20260222 (PMC13671151; doi:10.36660/abc.20260222)
Supplement: Supplementary file 2 [file 0066-782x-abc-123-4-e20260222-Suppl01.pdf]

Suplemento 1

Checklist para avaliação e certificação interna da competência em POCUS CV e concessão de privilégio institucional de atuação.

Nome: ______________________ **Data:** ___ / ___ / ___

Número de exames: 30+ ☐ 50+ ☐ 70+ ☐

Experiência certificada por: ______________________

1. Preparação para o exame

- Aborda o paciente adequadamente, identifica corretamente o paciente e demonstra atitude adequada e profissional: 0☐ 1☐ 2☐ 3☐
- Demonstra conhecimento das indicações básicas: 0☐ 1☐ 2☐ 3☐

2. Exame

- Aplicação dos conhecimentos de ultrassom no ajuste do equipamento e seleção do transdutor mais adequado: 0☐ 1☐ 2☐ 3☐
- Otimização da imagem: 0☐ 1☐ 2☐ 3☐
- Avaliação sequencial de janelas e estruturas (exame sistemático)
  - *Cardíacas (Paraesternal, Subcostal e Apical):* 0☐ 1☐ 2☐ 3☐
  - *Pulmão (pneumotórax, linhas B):* 0☐ 1☐ 2☐ 3☐
  - *Veias sistêmicas e veias femoral e poplítea:* 0☐ 1☐ 2☐ 3☐
- Interpretação da imagem (baseado em respostas binárias do tipo SIM ou NÃO (nível 2) ou com quantificação da gravidade quando aplicável (nível 3)
  - *Há aumento do tamanho do VE?* 0☐ 1☐ 2☐ 3☐
  - *Há aumento do tamanho do VD?* 0☐ 1☐ 2☐ 3☐
  - *Há congestão pulmonar (linhas B)?* 0☐ 1☐ 2☐ 3☐
  - *Há congestão venosa sistêmica (VEXUS score)?* 0☐ 1☐ 2☐ 3☐
  - *Há trombose venosa profunda?* 0☐ 1☐ 2☐ 3☐
  - *Há alteração na função sistólica global do VE?* 0☐ 1☐ 2☐ 3☐
  - *Há alteração na função sistólica global do VD?* 0☐ 1☐ 2☐ 3☐
  - *Há derrame pericárdico?* 0☐ 1☐ 2☐ 3☐
  - *Há pletora da veia cava inferior?* 0☐ 1☐ 2☐ 3☐
  - *Há sinais de hipovolemia?* 0☐ 1☐ 2☐ 3☐

3. Pós-exame

- Interpretação crítica e apropriada dos resultados: 0☐ 1☐ 2☐ 3☐
- Resultados corretamente integrados na tomada de decisão: 0☐ 1☐ 2☐ 3☐
- Documentação dos resultados de imagem e do desfecho clínico embasado nos resultados do ultrassom cardíaco: 0☐ 1☐ 2☐ 3☐
- Reconhece que o exame não foi conclusivo: 0☐ 1☐ 2☐ 3☐

4. Interpretação de vídeos

- Questões de múltipla escolha: 0☐ 1☐ 2☐ 3☐
- Questões abertas: 0☐ 1☐ 2☐ 3☐

Ficha adaptada do "Objective Structured Assessment of Ultrasound Skills (OSAUS). Tolsgaard, MG et al.[19]" e "Point of Care Ultrasound Competence Entrustment Scale. Mani et al.[20]". Caso não atinja 70% do escore para o nível desejado, conforme PROTOCOLO AcQUIRE,o treinamento e um novo teste deverão ser realizados. Se o treinando for aprovado, a certificação deve ser validada pelo Coordenador do treinamento junto à Comissão de Ensino e ao Núcleo de Segurança do Hospital, para concessão dos privilégios institucionais para atuação em POCUS. Entretanto, essa certificação não define competência e não habilita a ser um profissional formador. A certificação de competência deverá ser conferida pelas Sociedades de Especialidades e esse é um desafio futuro. Essa concessão deve ser restrita apenas ao serviço onde atua.
