# Supplementary material for: Posicionamento sobre o Uso do Ultrassom "Point of Care" (POCUS) na Medicina Cardiovascular – 2026
Source: Arq Bras Cardiol. 2026 May 4;123(4):e20260222. [Article in Portuguese] doi: 10.36660/abc.20260222 (PMC13671151; doi:10.36660/abc.20260222)
Supplement: Supplementary file 3 [file 0066-782x-abc-123-4-e20260222-Suppl02-en.pdf]

Statement

Supplement 2

Compiled Videos

SECTION 2 – Cardiac POCUS

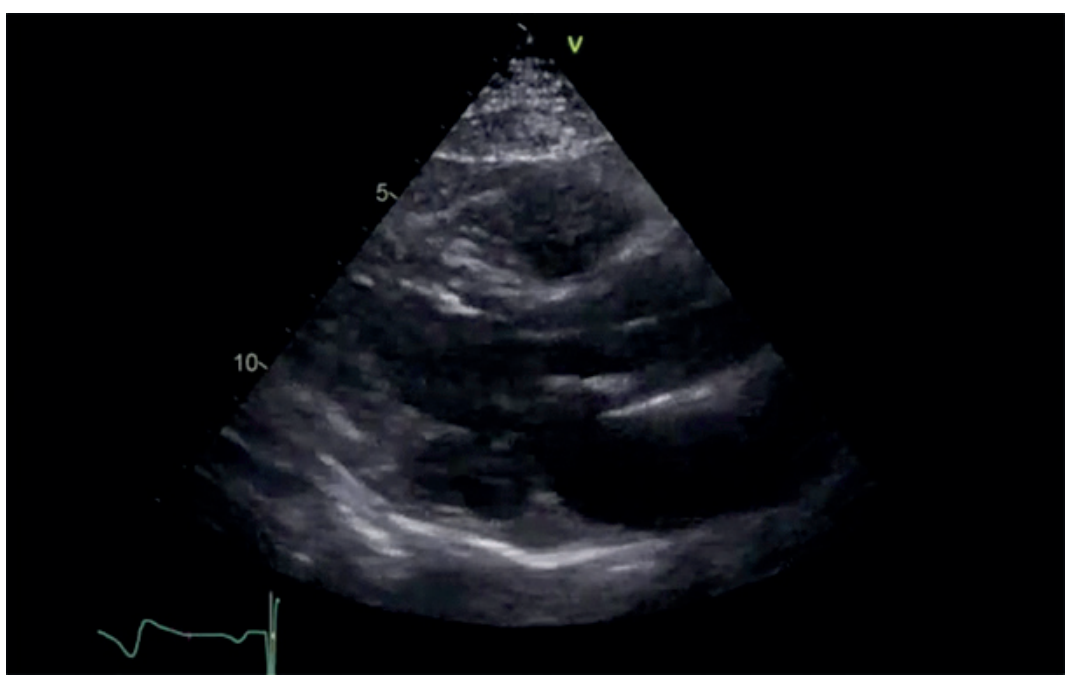

Video 01 – *Parasternal long-axis view showing normal global left ventricular systolic function. Link: http://abccardiol.org/supplementary-material/2026/12304/PosicionamentoPOCUS/Video 01 PLAX normal.mp4*

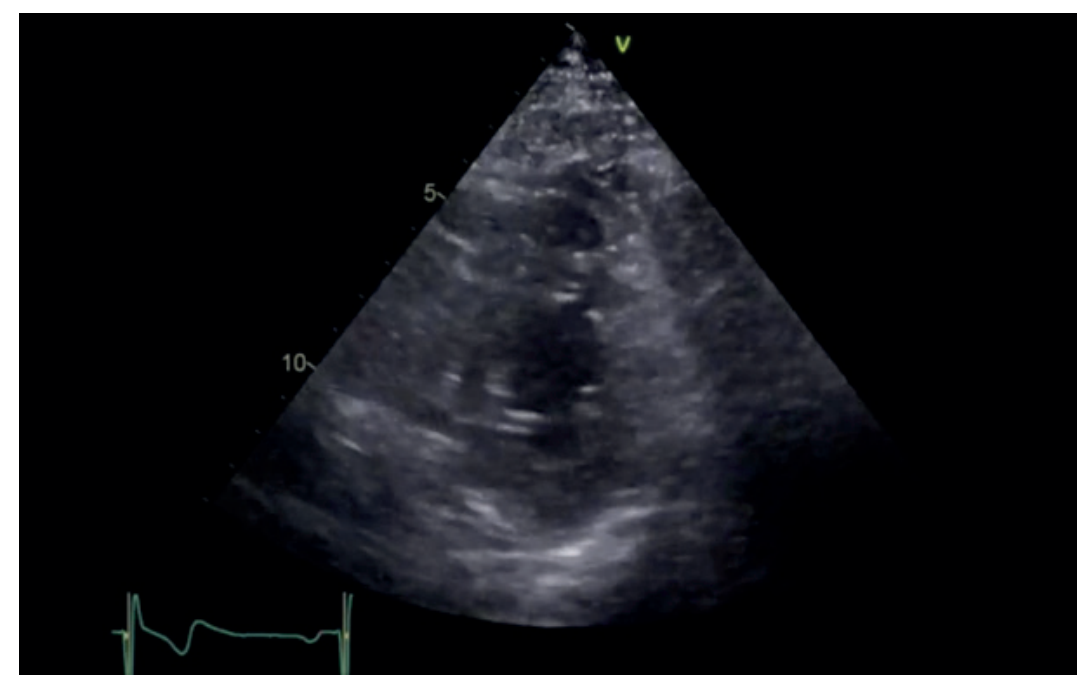

Video 02 – *Parasternal short-axis view showing normal global left ventricular systolic function. Link: http://abccardiol.org/supplementary-material/2026/12304/PosicionamentoPOCUS/Video 02 PSAX normal.mp4*

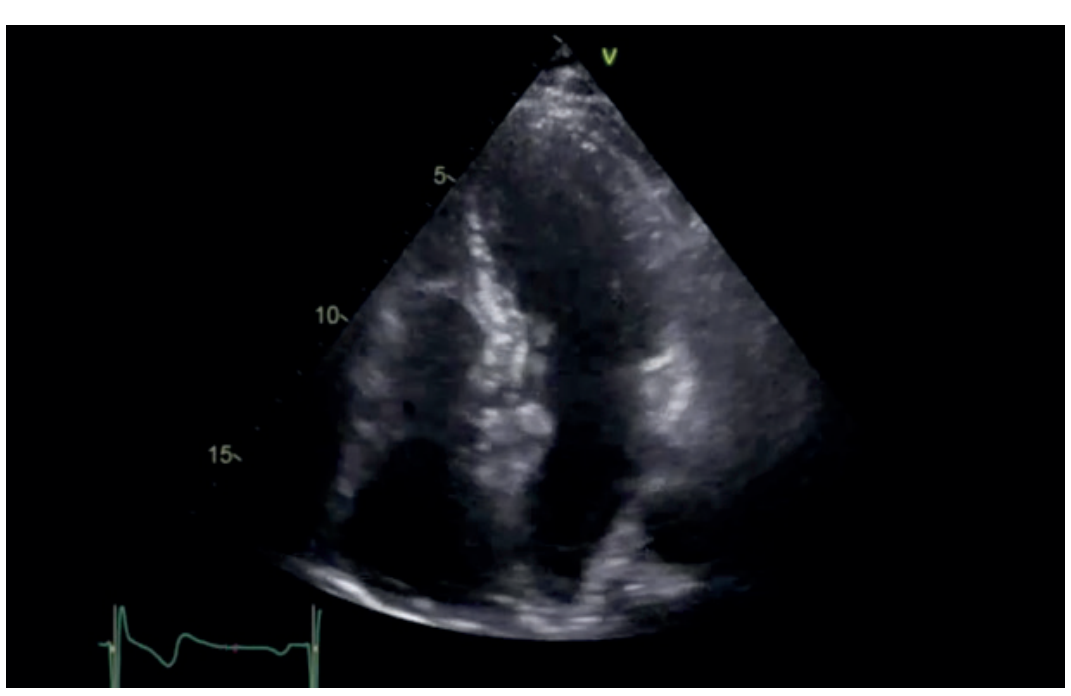

Video 03 – *Apical 4-chamber view showing normal global left ventricular systolic function. Link: http://abccardiol.org/supplementary-material/2026/12304/PosicionamentoPOCUS/Video 03 A4C normal.mp4*

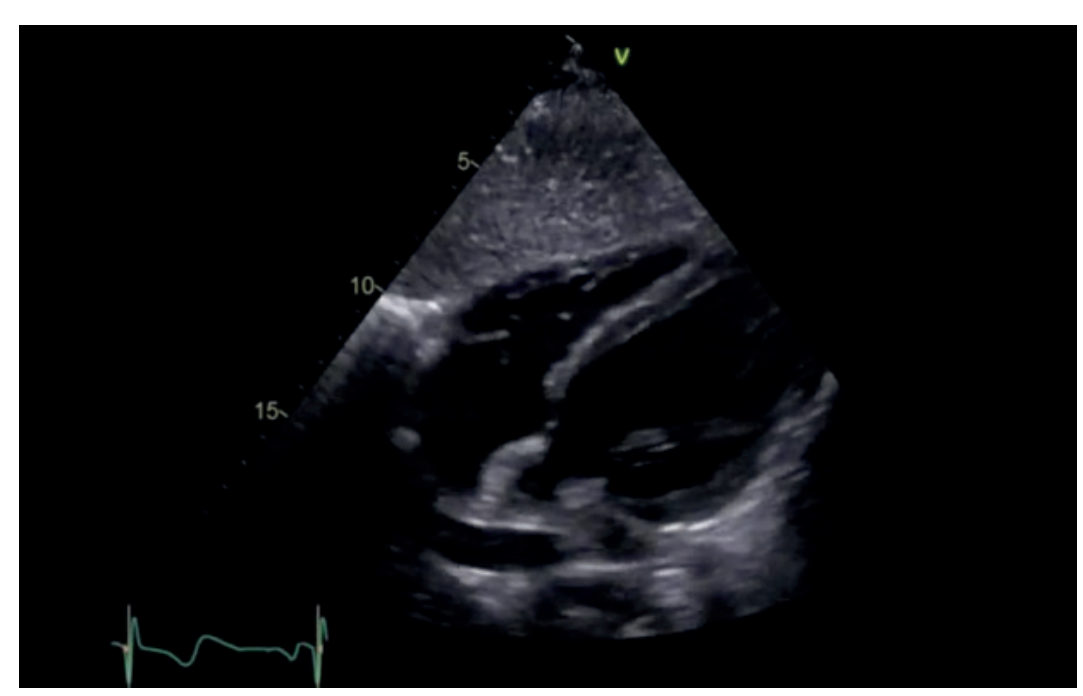

Video 04 – *Subcostal 4-chamber view showing normal global left ventricular systolic function. Link: http://abccardiol.org/supplementary-material/2026/12304/PosicionamentoPOCUS/Video 04 Subcostal Normal.mp4*

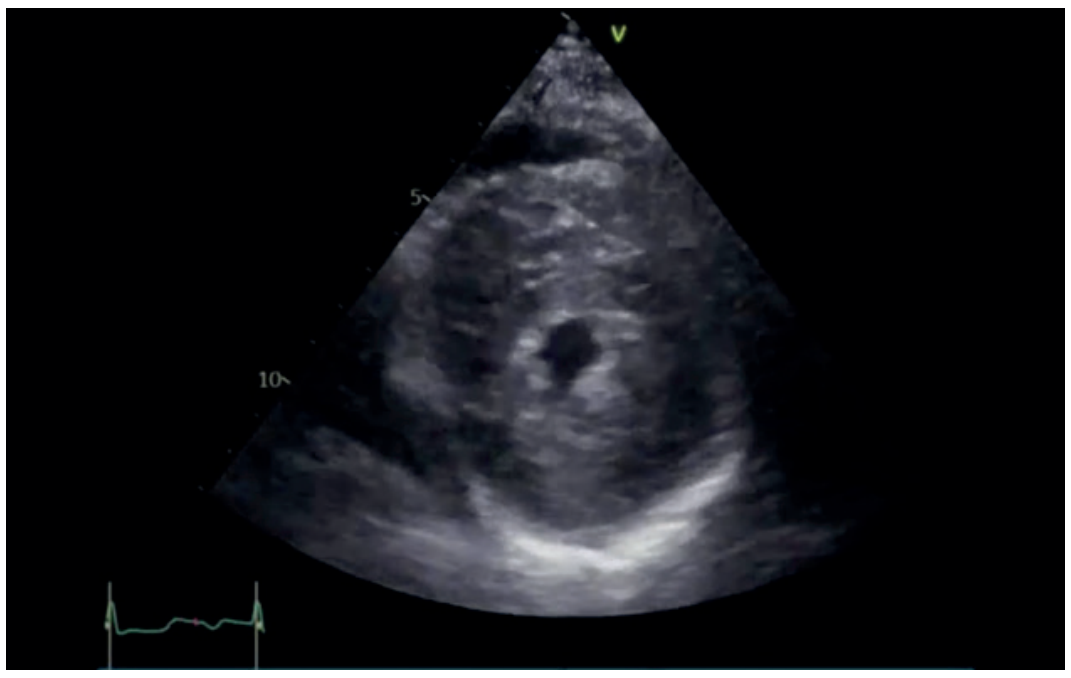

Video 05 – *Parasternal short-axis view showing hyperdynamic global left ventricular systolic function. Link: http://abccardiol.org/supplementary-material/2026/12304/PosicionamentoPOCUS/Video 05 PSAX hiperdinamico.mp4*

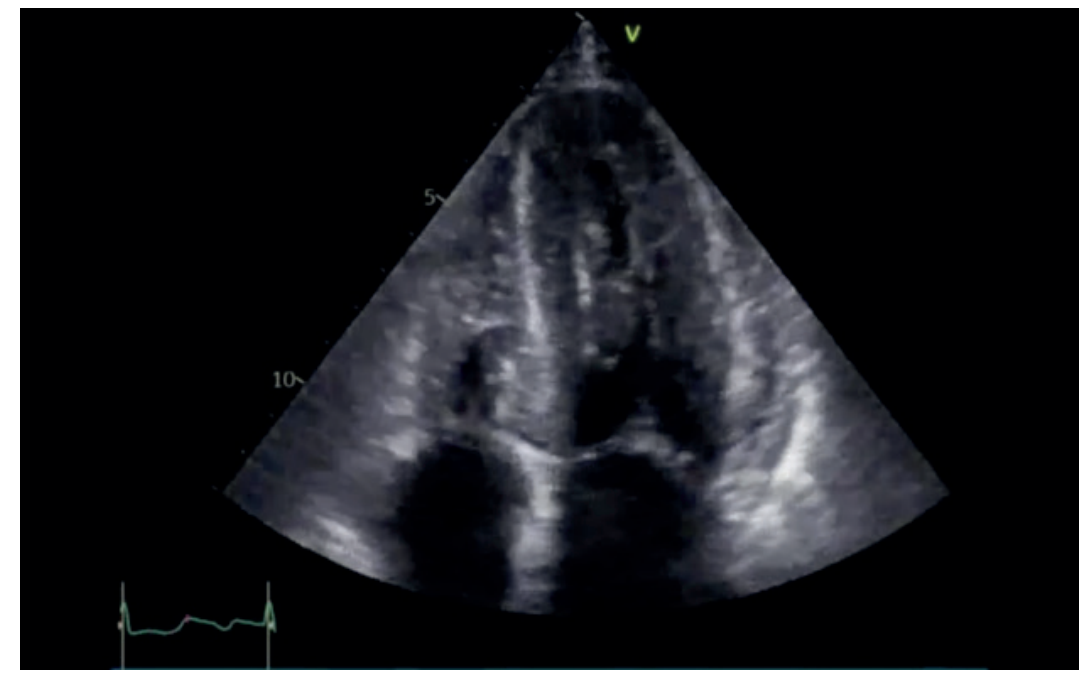

Video 06 – *Apical 4-chamber view showing hyperdynamic global left ventricular systolic function. Link: http://abccardiol.org/supplementary-material/2026/12304/PosicionamentoPOCUS/Video 06 A4C hiperdinamico.mp4*

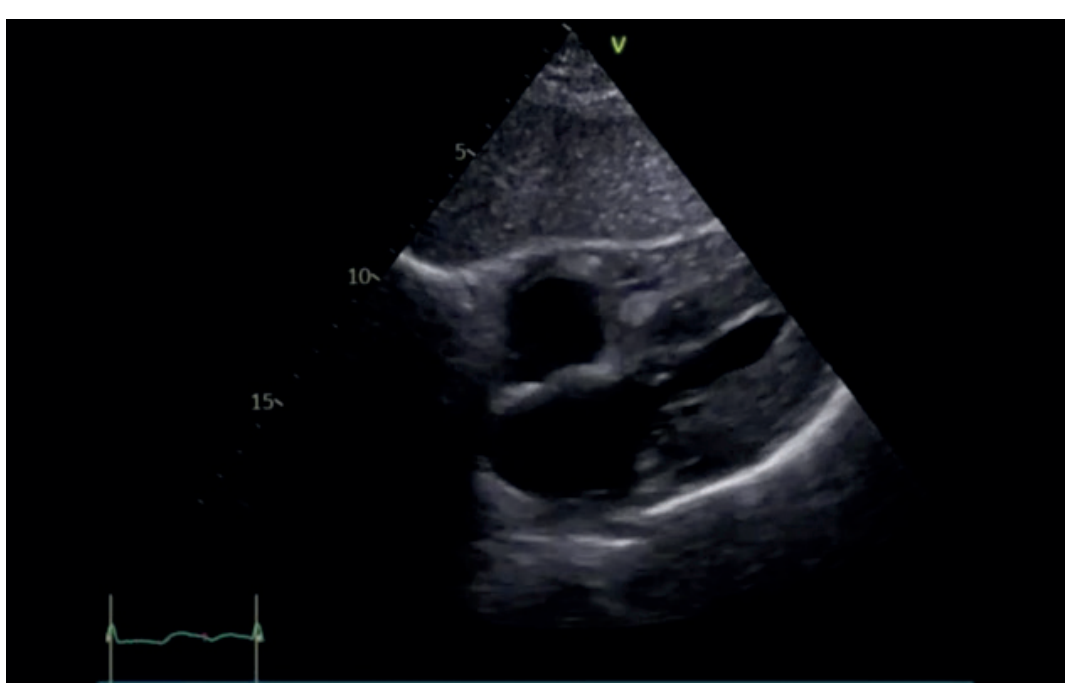

Video 07 – *Subcostal 4-chamber view showing hyperdynamic global left ventricular systolic function. Link: http://abccardiol.org/supplementary-material/2026/12304/PosicionamentoPOCUS/Video 07 Subcostal hiperdinamico.mp4*

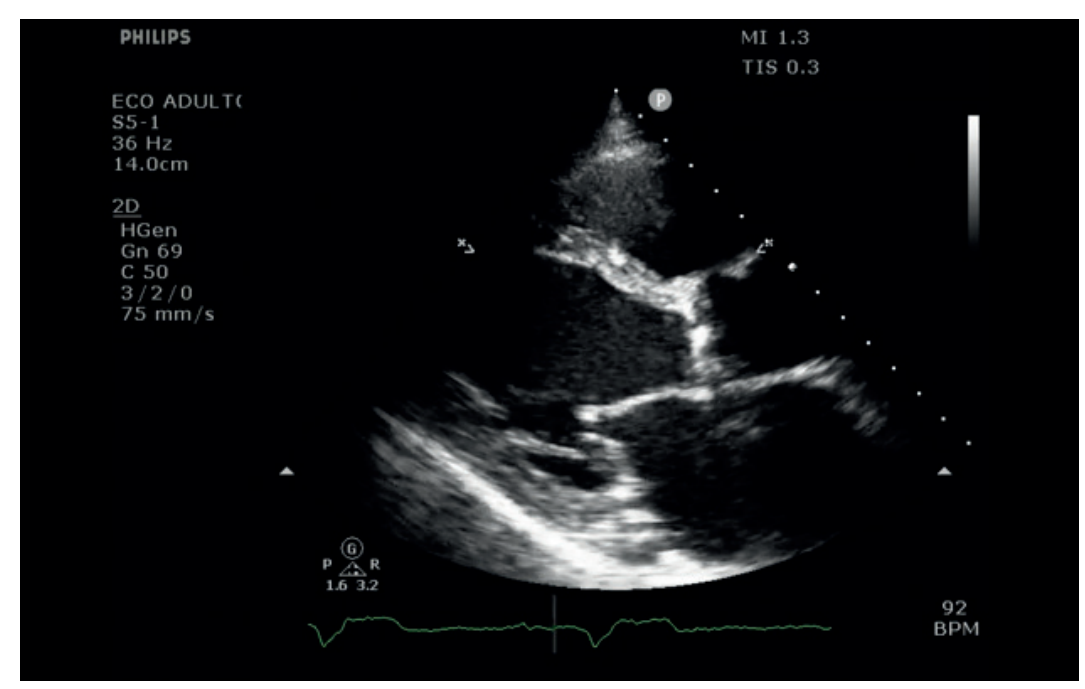

Video 08 – *Parasternal long-axis view showing reduced global left ventricular systolic function. Link: http://abccardiol.org/supplementary-material/2026/12304/PosicionamentoPOCUS/Video 08 PLAX FS reduzida.mp4*

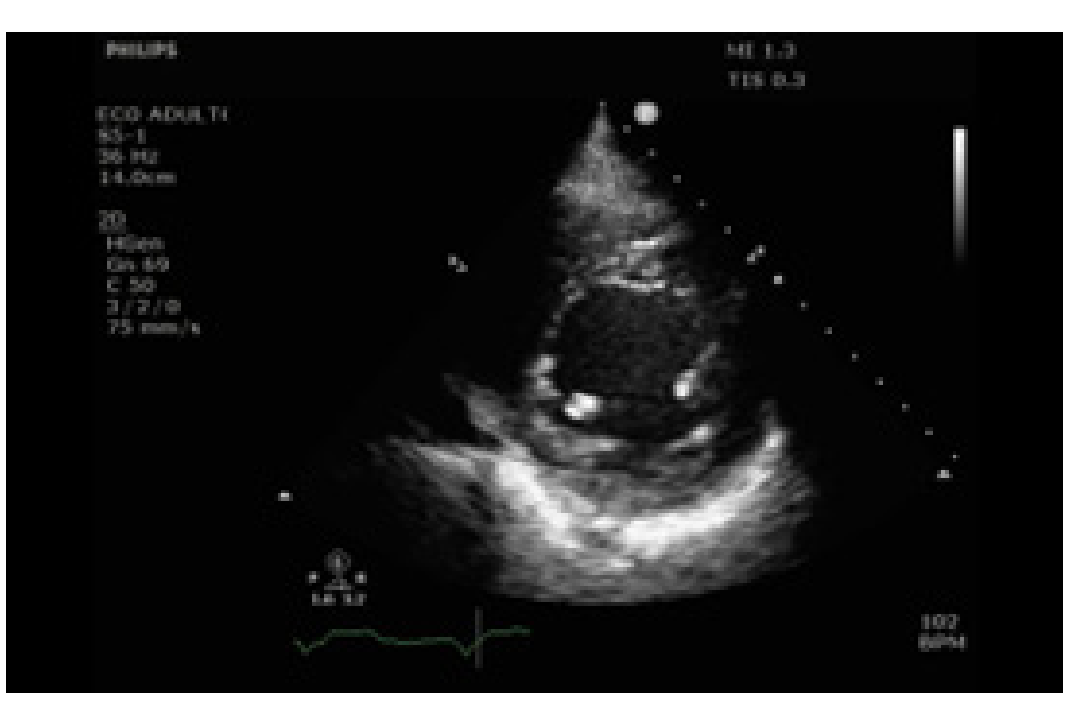

Video 09 – *Parasternal short-axis view showing reduced global left ventricular systolic function. Link: http://abccardiol.org/supplementary-material/2026/12304/PosicionamentoPOCUS/Video 09 PSAX FS reduzida.mp4*

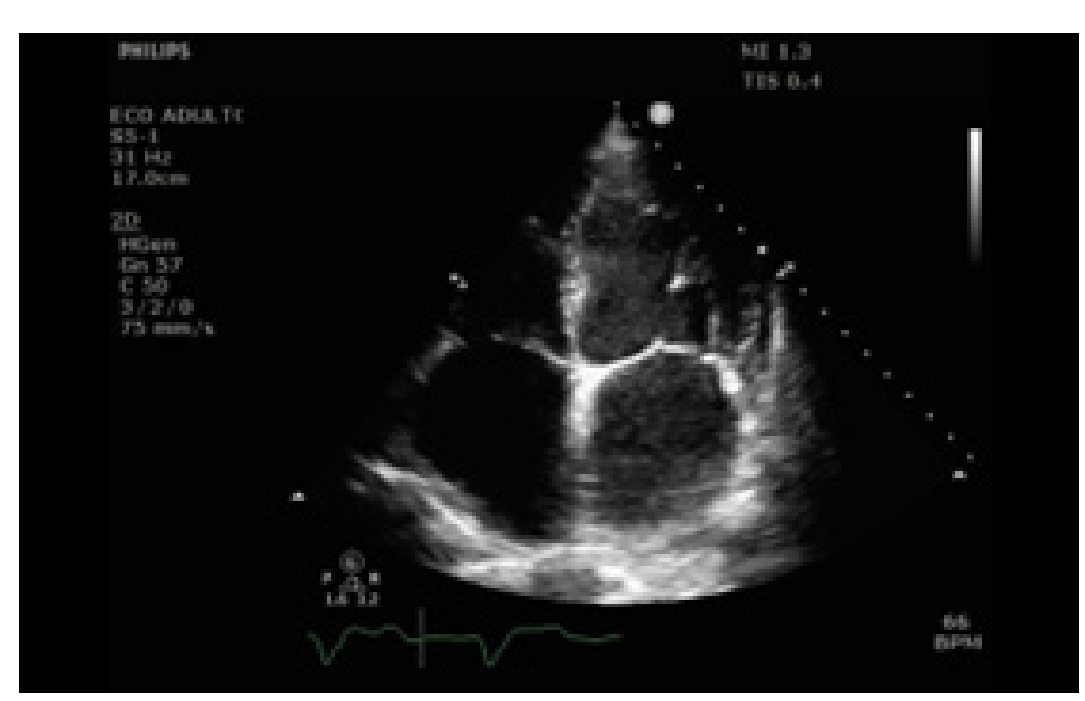

Video 10 – *Apical 4-chamber view showing reduced global left ventricular systolic function. Link: http://abccardiol.org/supplementary-material/2026/12304/PosicionamentoPOCUS/Video 10 APICAL 4C FS reduzida.mp4*

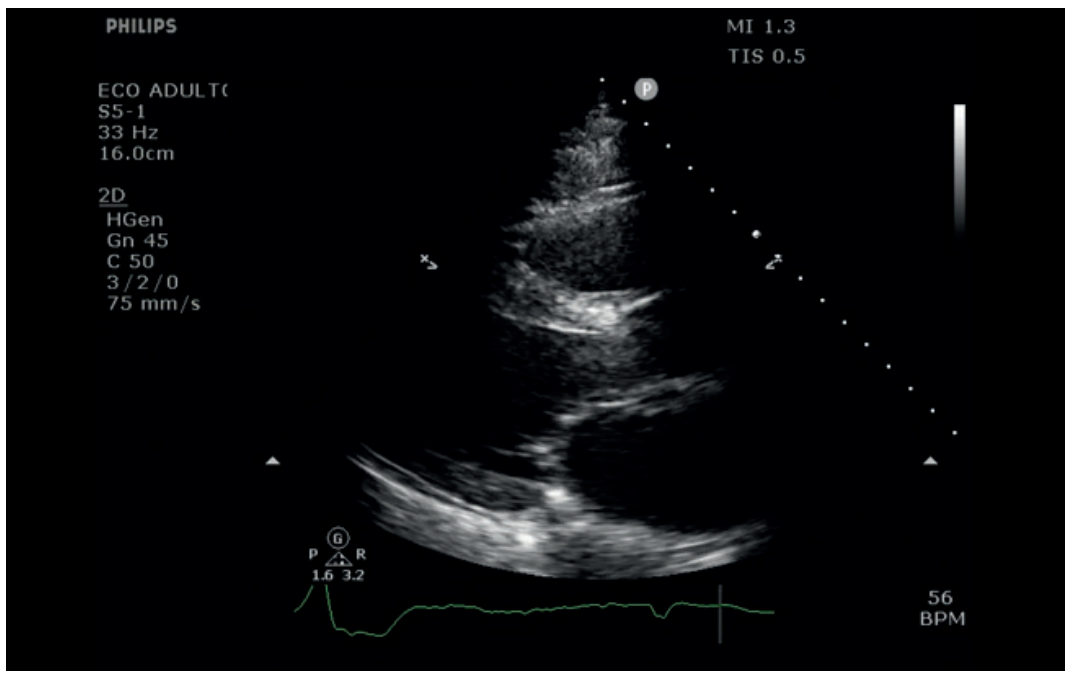

Video 11 – *Parasternal long-axis view showing severe global left ventricular systolic dysfunction. Link: http://abccardiol.org/supplementary-material/2026/12304/PosicionamentoPOCUS/Video 11 PLAX Disfuncao grave.mp4*

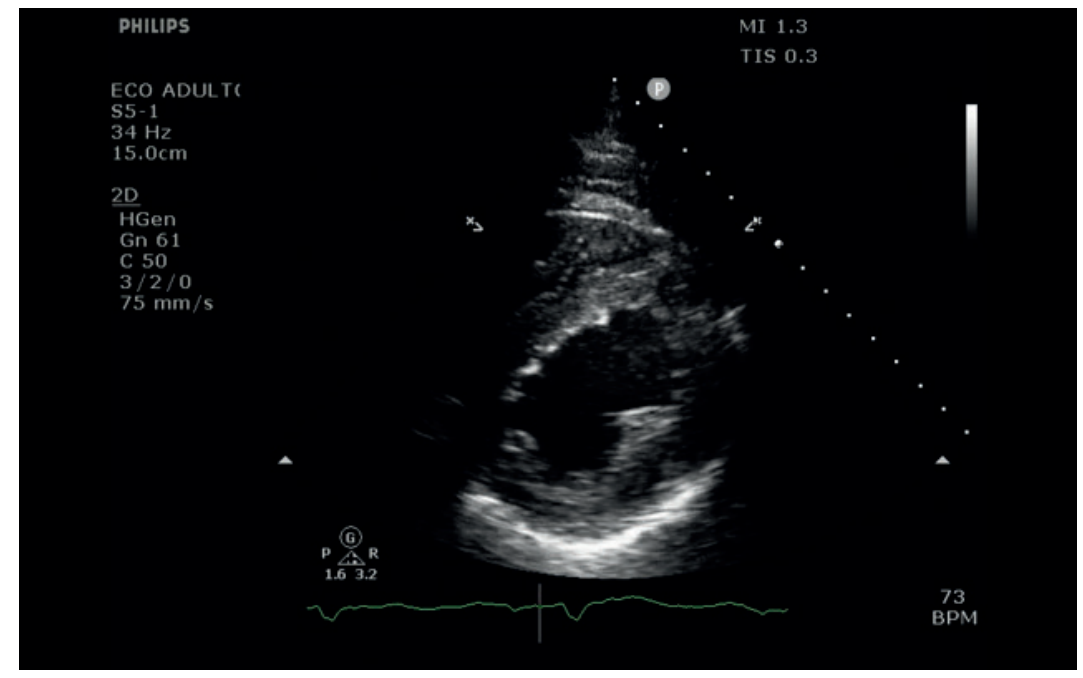

Video 12 – *Parasternal short-axis view showing severe global left ventricular systolic dysfunction. Link: http://abccardiol.org/supplementary-material/2026/12304/PosicionamentoPOCUS/Video 12 PSAX disfuncao grave.mp4*

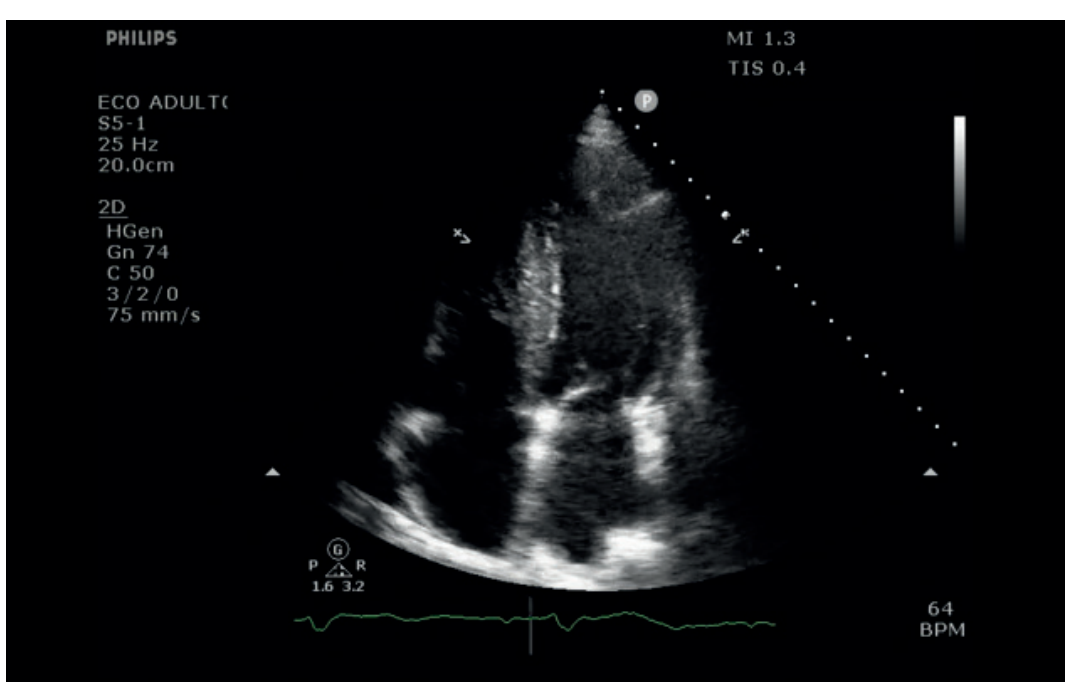

Video 13 – *Apical 4-chamber view showing severe global left ventricular systolic dysfunction. Link: http://abccardiol.org/supplementary-material/2026/12304/PosicionamentoPOCUS/Video 13 Apical 4C disfuncao grave.mp4*

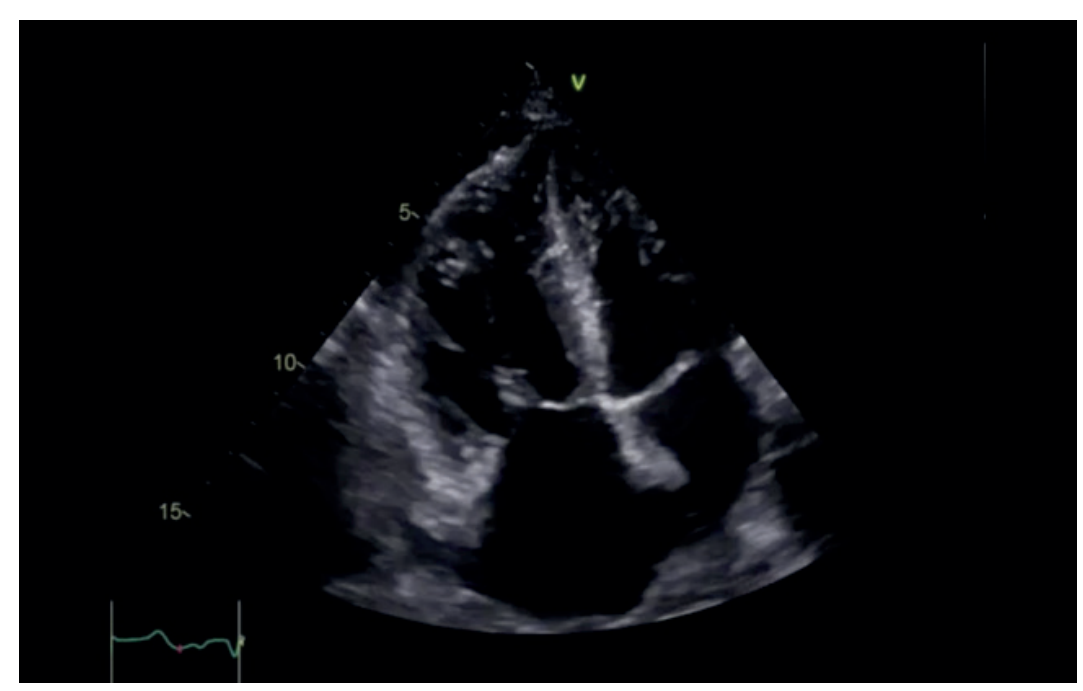

Video 14 – *Apical 4-chamber view showing McConnell sign in pulmonary embolism. Link: http://abccardiol.org/supplementary-material/2026/12304/PosicionamentoPOCUS/Video 14 TEP McConnell.mp4*

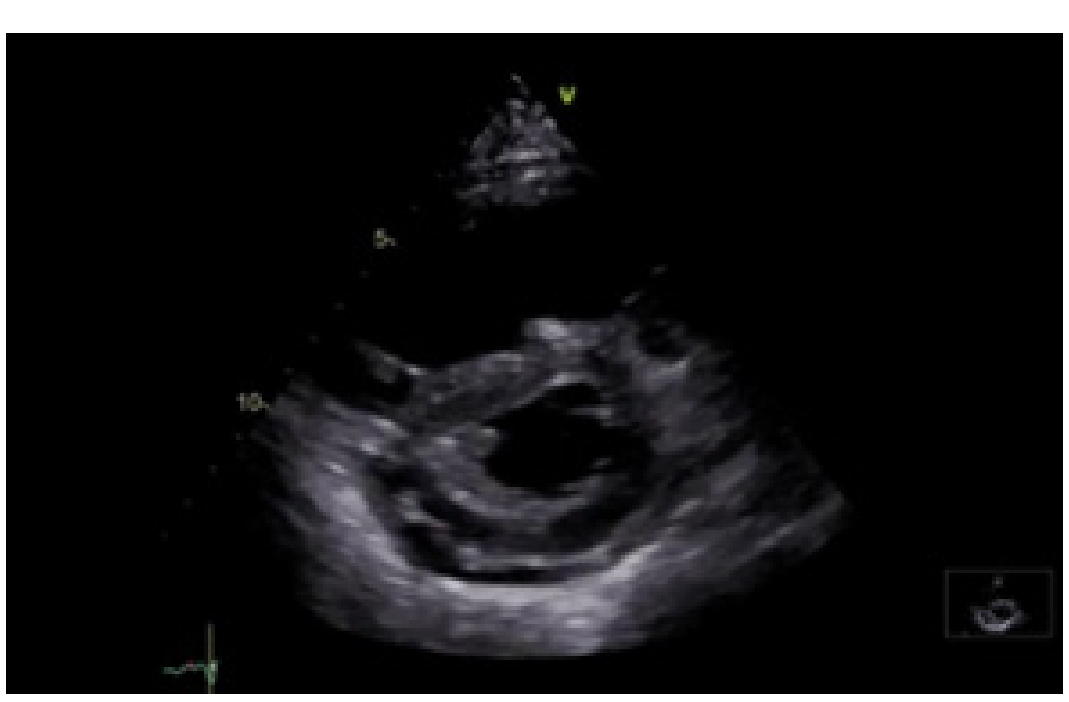

Video 15 – *Parasternal short-axis view showing interventricular septal flattening (D-shape) in pulmonary embolism. Link: http://abccardiol.org/supplementary-material/2026/12304/PosicionamentoPOCUS/Video 15 TEP- D shape.mp4*

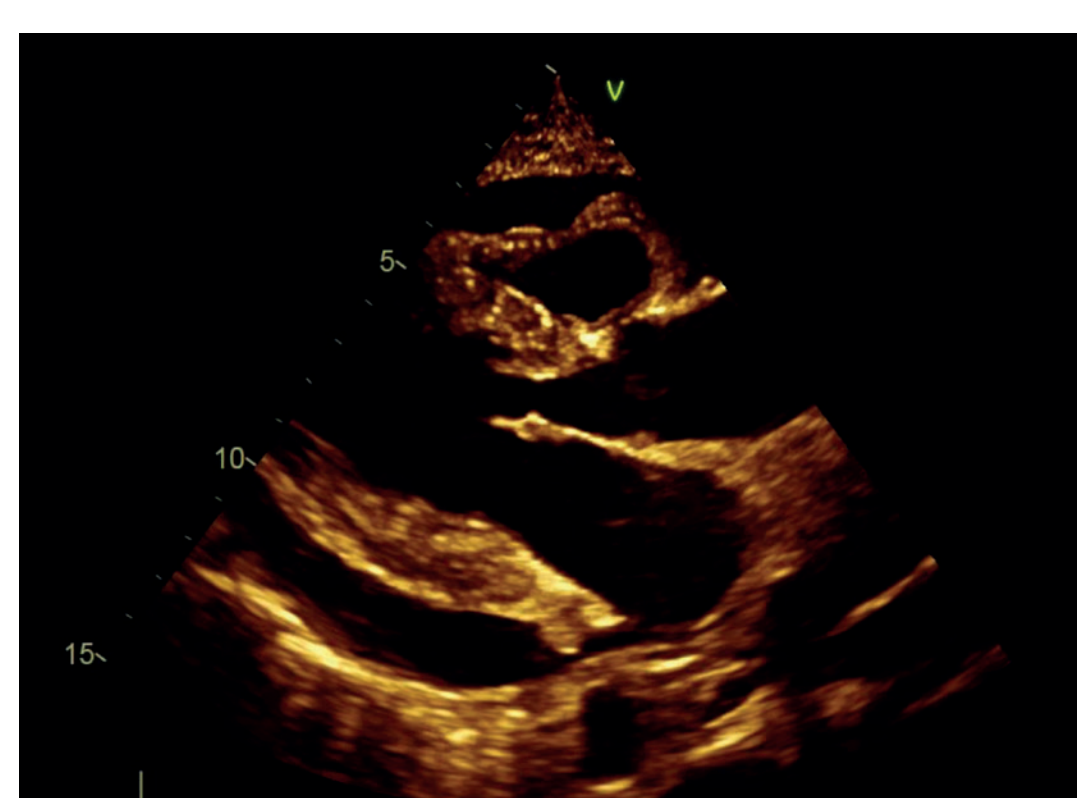

Video 16 – *Parasternal long-axis view showing large pericardial effusion with right ventricular diastolic collapse. Link: http://abccardiol.org/supplementary-material/2026/12304/PosicionamentoPOCUS/Video 16 Tamponamento PLAX.mp4*

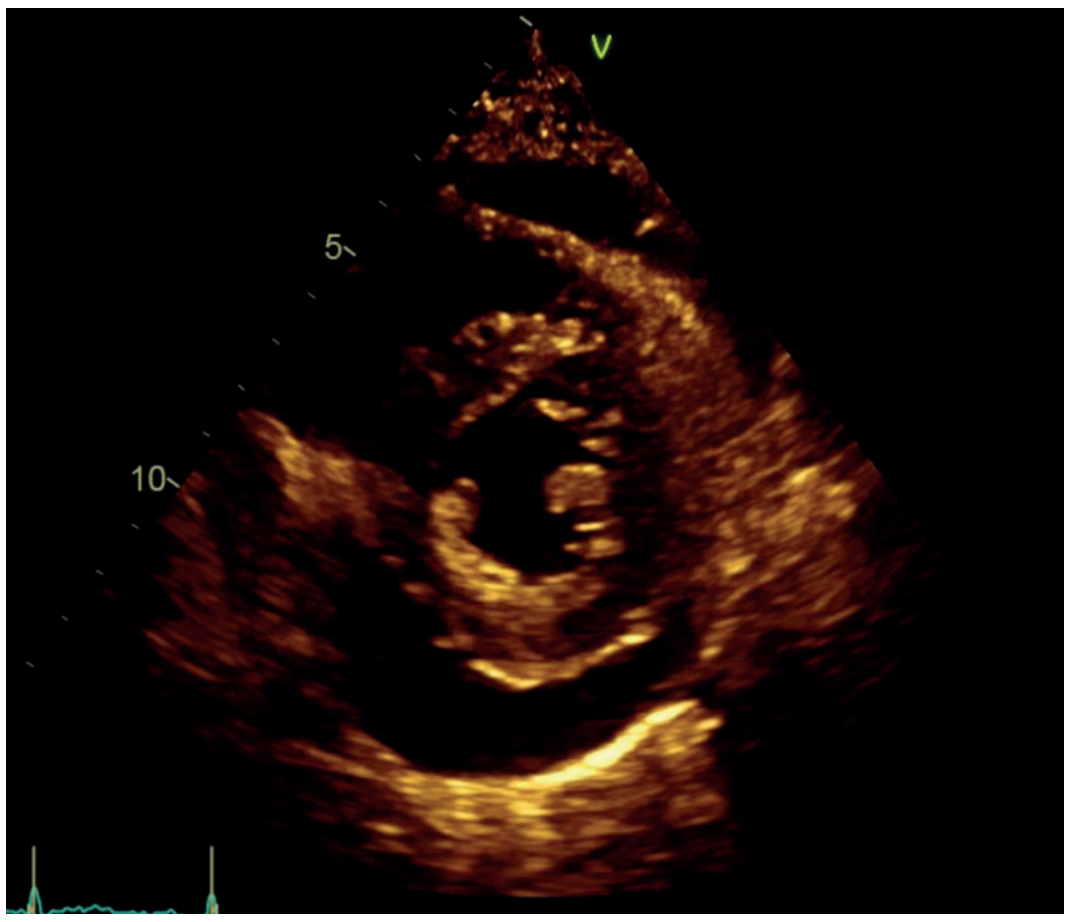

Video 17 – *Parasternal short-axis view showing large pericardial effusion with right ventricular diastolic collapse. Link: http://abccardiol.org/supplementary-material/2026/12304/PosicionamentoPOCUS/Video 17 Tamponamento PSAX.mp4*

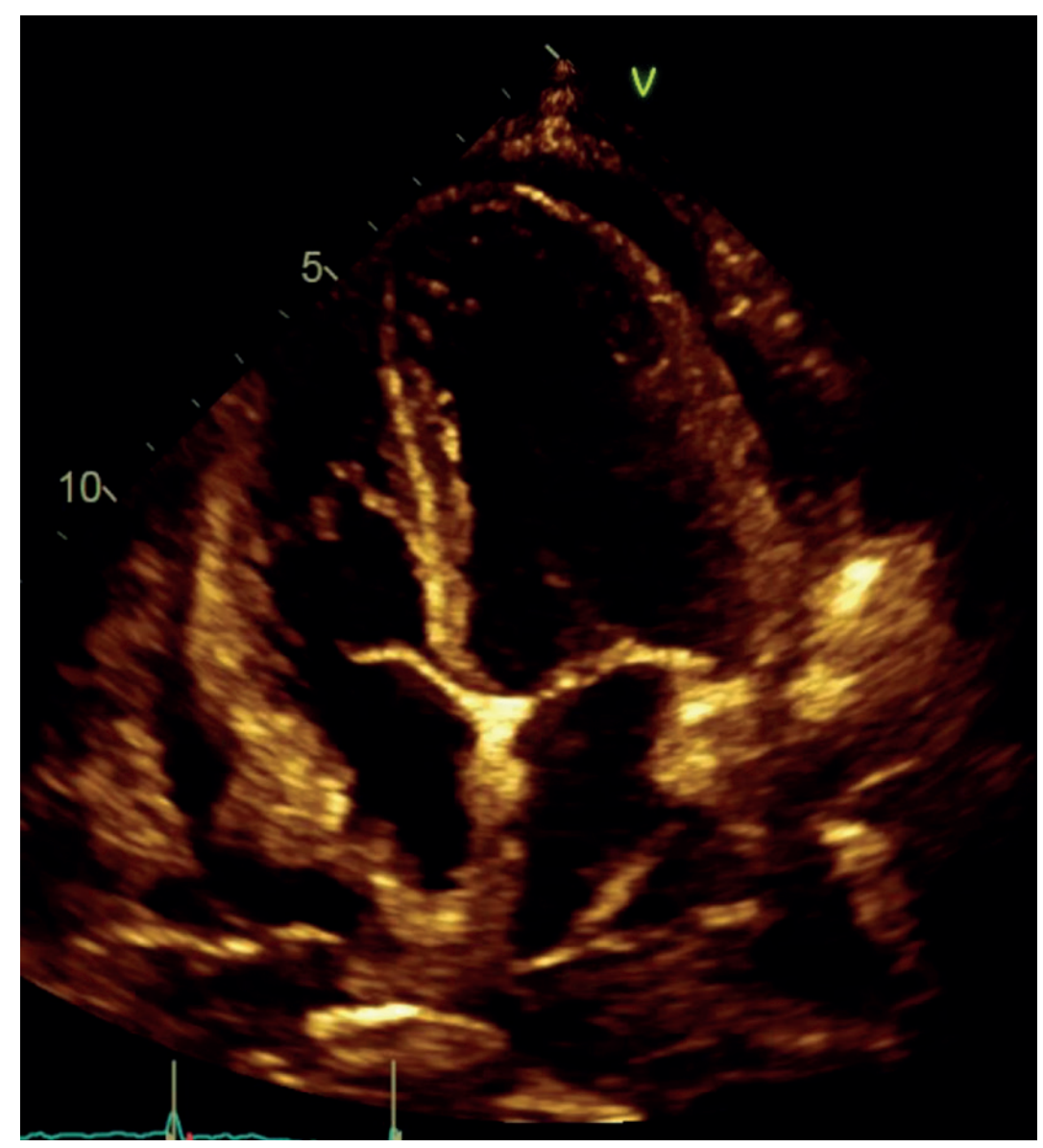

Video 18 – *Apical 4-chamber view showing large pericardial effusion with diastolic restriction. Link: http://abccardiol.org/supplementary-material/2026/12304/PosicionamentoPOCUS/Video 18 Tamponamento A4C.mp4*

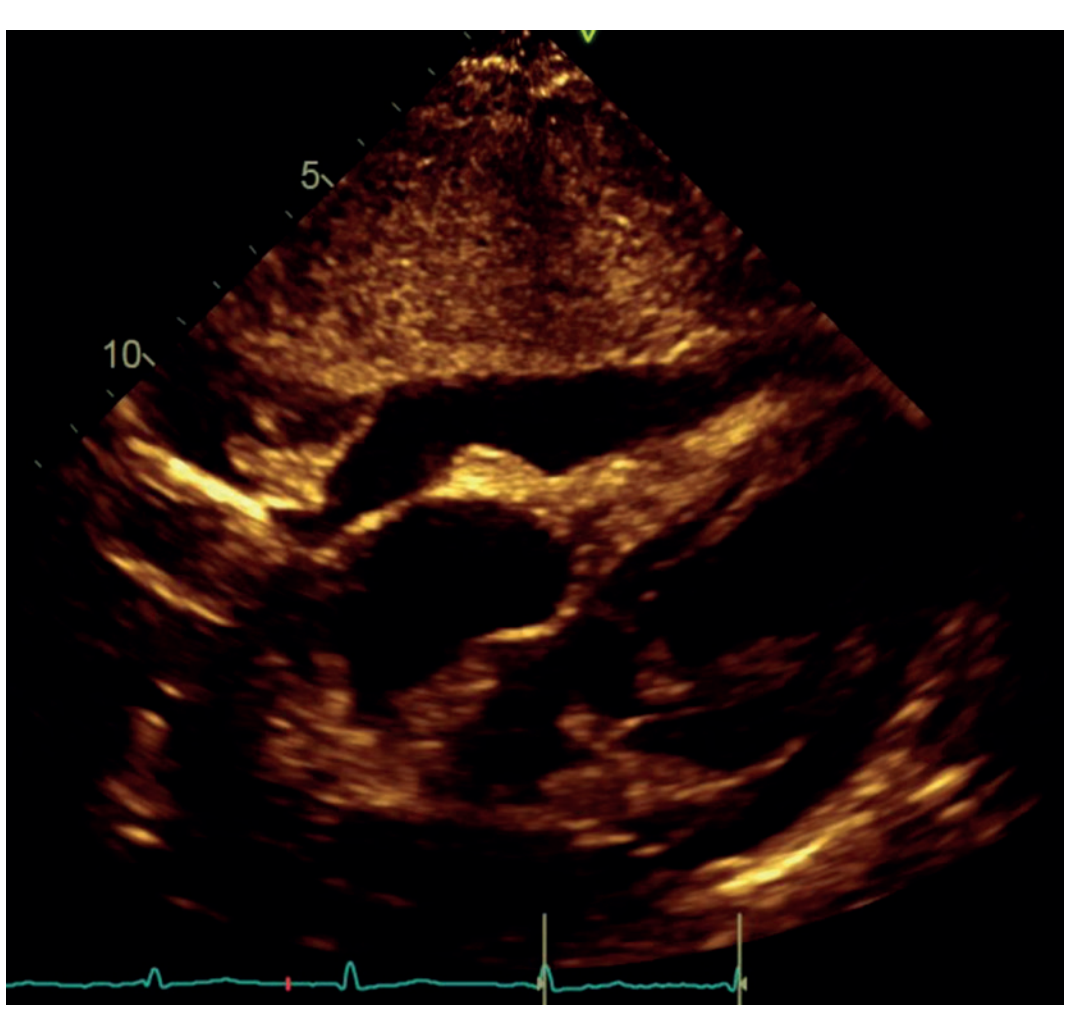

Video 19 – *Subcostal view showing large pericardial effusion with right ventricular diastolic collapse. Link: http://abccardiol.org/supplementary-material/2026/12304/PosicionamentoPOCUS/Video 19 Tamponamento subcostal.mp4*

Supplement 2

Compiled Videos

SECTION 3 – Lung ultrasound

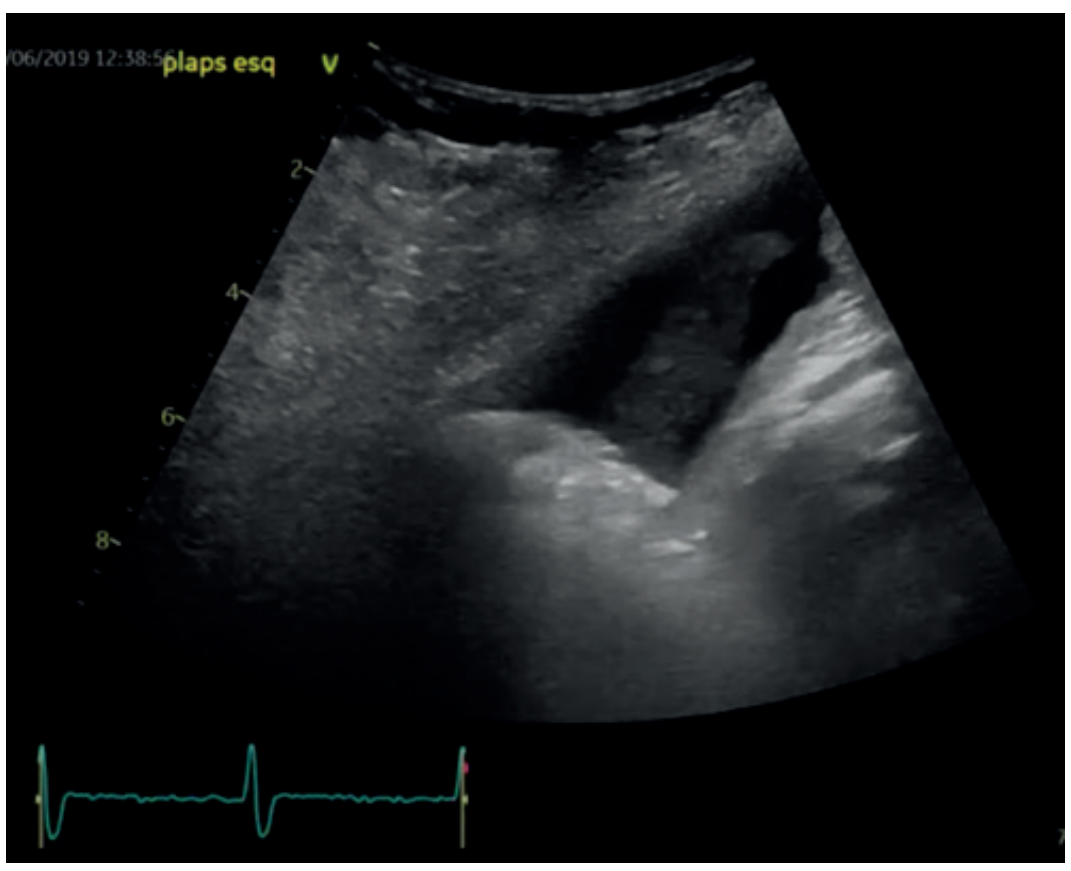

Video 20 – *Pulmonary atelectasis characterized by a sheet of consolidated lung tissue with sinusoidal movements (jellyfish/medusa sign) within pleural fluid. Link: http://abccardiol.org/supplementary-material/2026/12304/PosicionamentoPOCUS/Video 20 US pulm atelectasia.mp4*

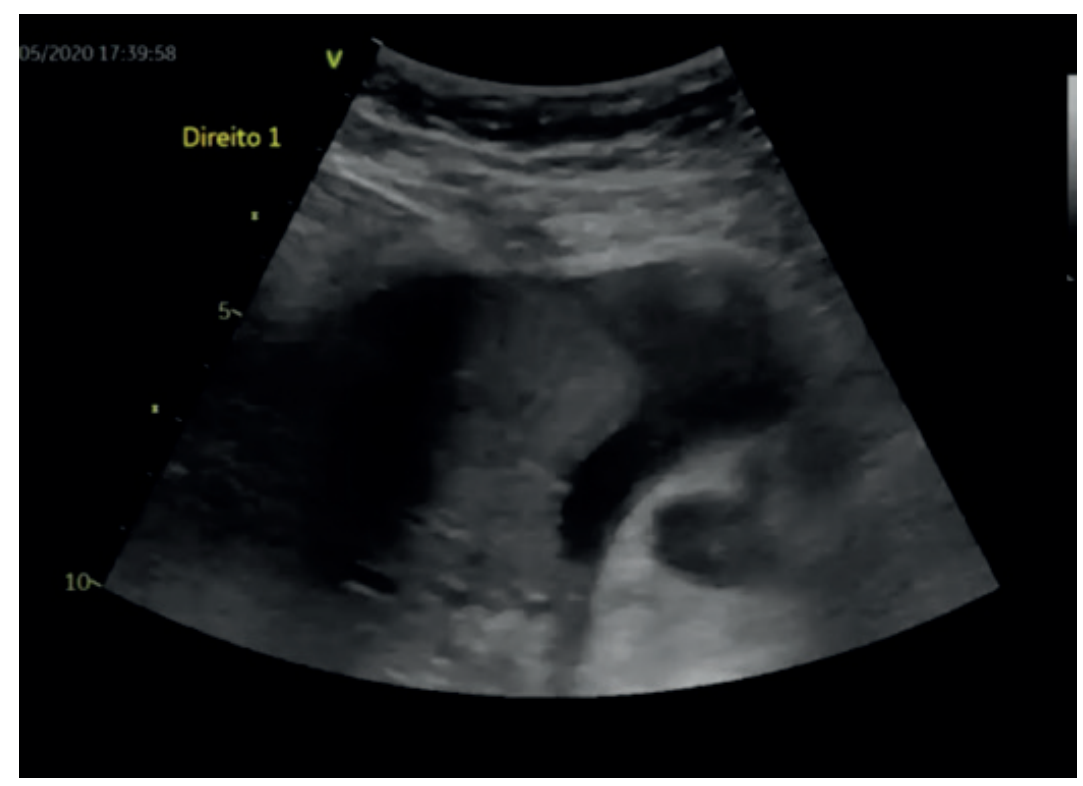

Video 21 – *Translobar consolidation with loss of lung aeration. The lung parenchyma is filled with infectious fluid, giving it a solid appearance (hepatization) extending throughout the entire ultrasound field. Link: http://abccardiol.org/supplementary-material/2026/12304/PosicionamentoPOCUS/Video 21 US pulm consolidação translobar.mp4*

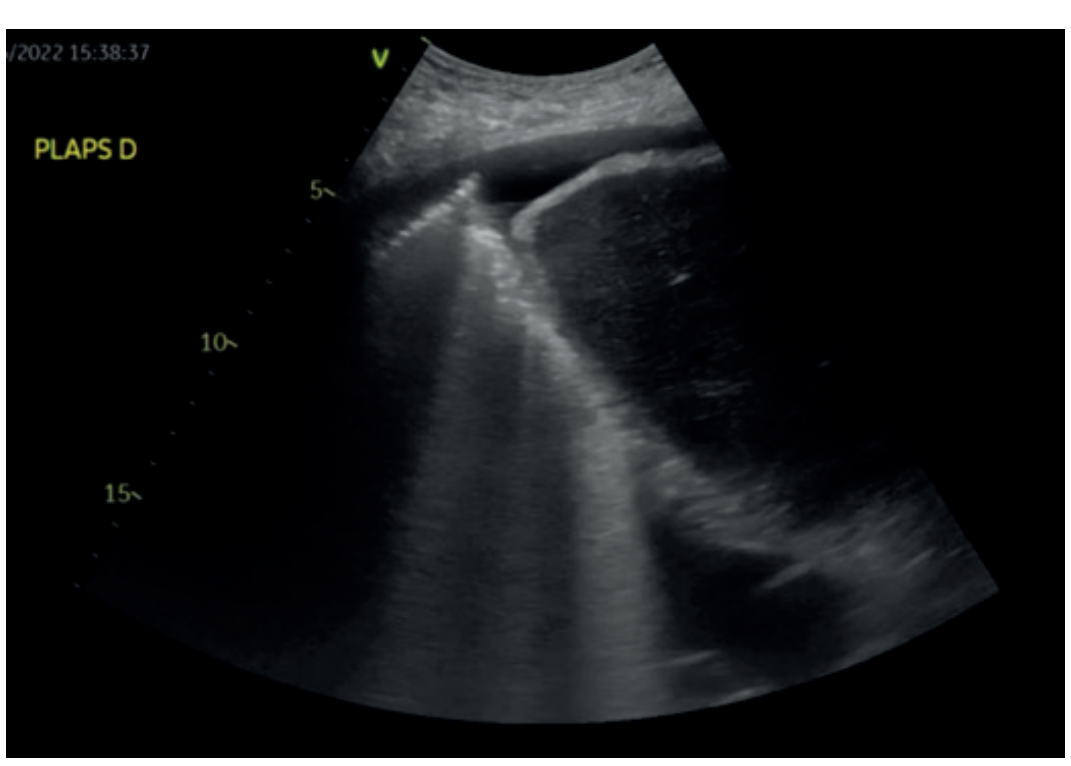

Video 22 – *Mild pleural effusion associated with pleural thickening and pleural adhesions near the diaphragmatic dome. Link: http://abccardiol.org/supplementary-material/2026/12304/PosicionamentoPOCUS/Video 22 US pulm derrame pl perdadeslizamento.mp4*

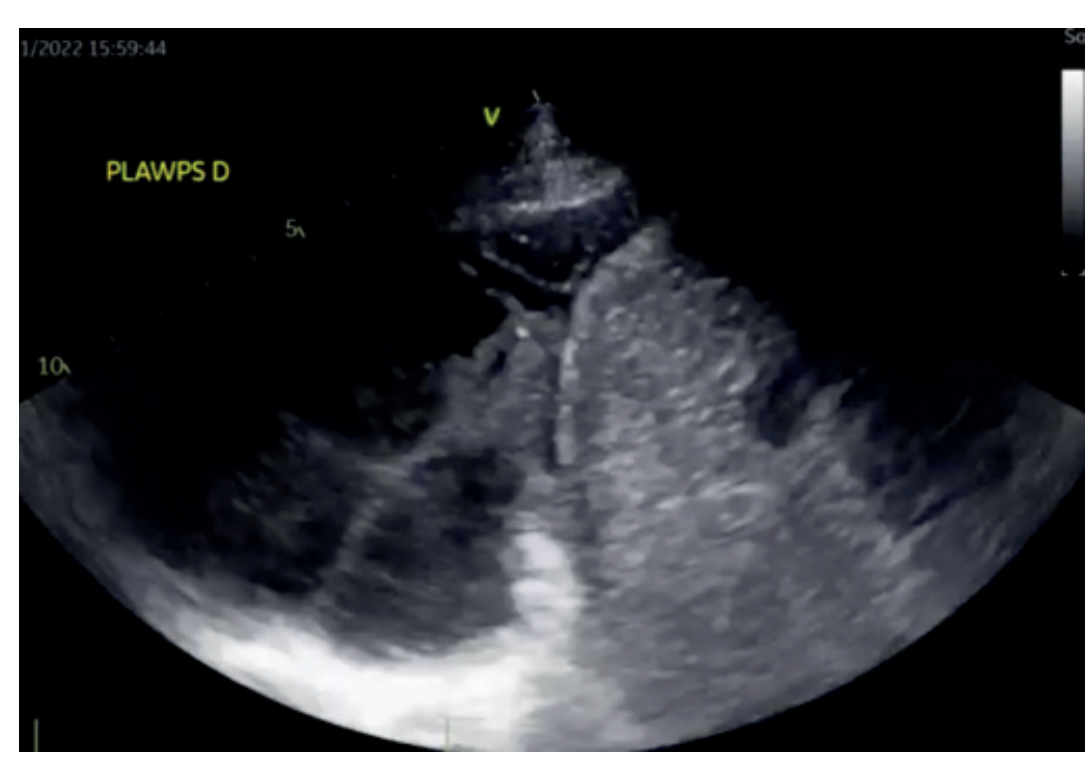

Video 23 – *Complex pleural effusion with debris and septations, suggestive of infectious origin. Link: http://abccardiol.org/supplementary-material/2026/12304/PosicionamentoPOCUS/Video 23 US pulm derrame septado.mp4*

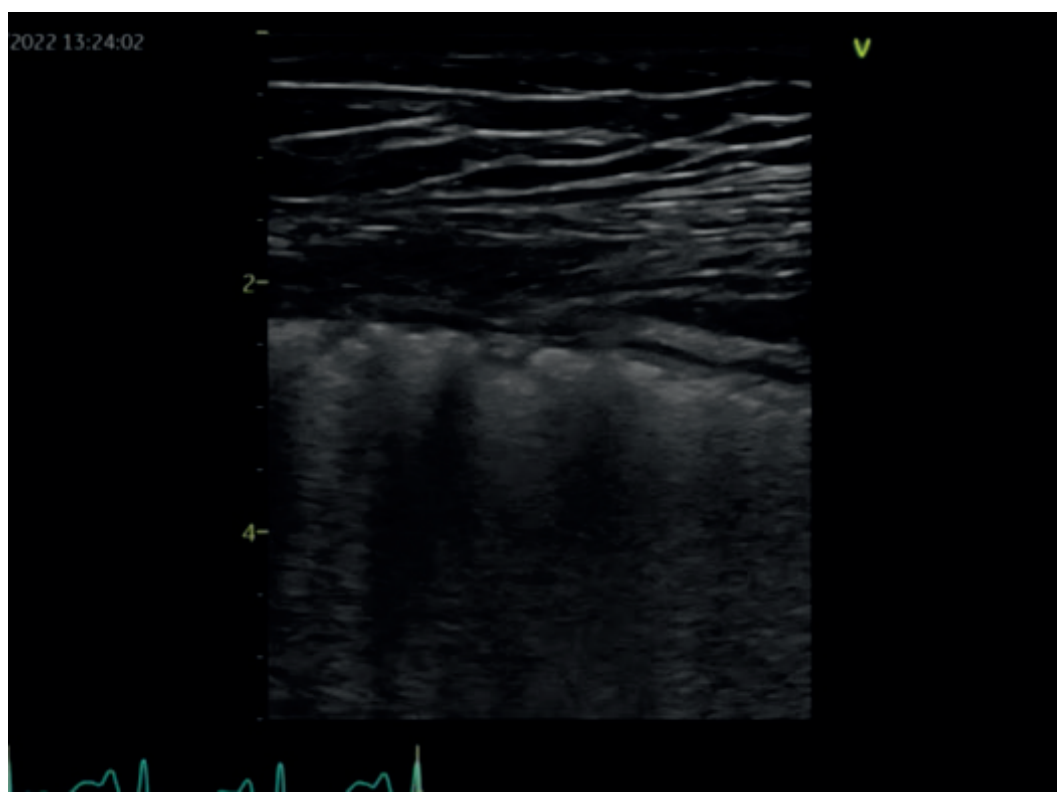

Video 24 – *Pleural line thickening and irregularity with B-lines and subpleural cysts in a patient with scleroderma-related pulmonary fibrosis. Best visualized with a linear probe. Link: http://abccardiol.org/supplementary-material/2026/12304/PosicionamentoPOCUS/Video 24 US pulm fibrose pulm esclerodermia.mp4*

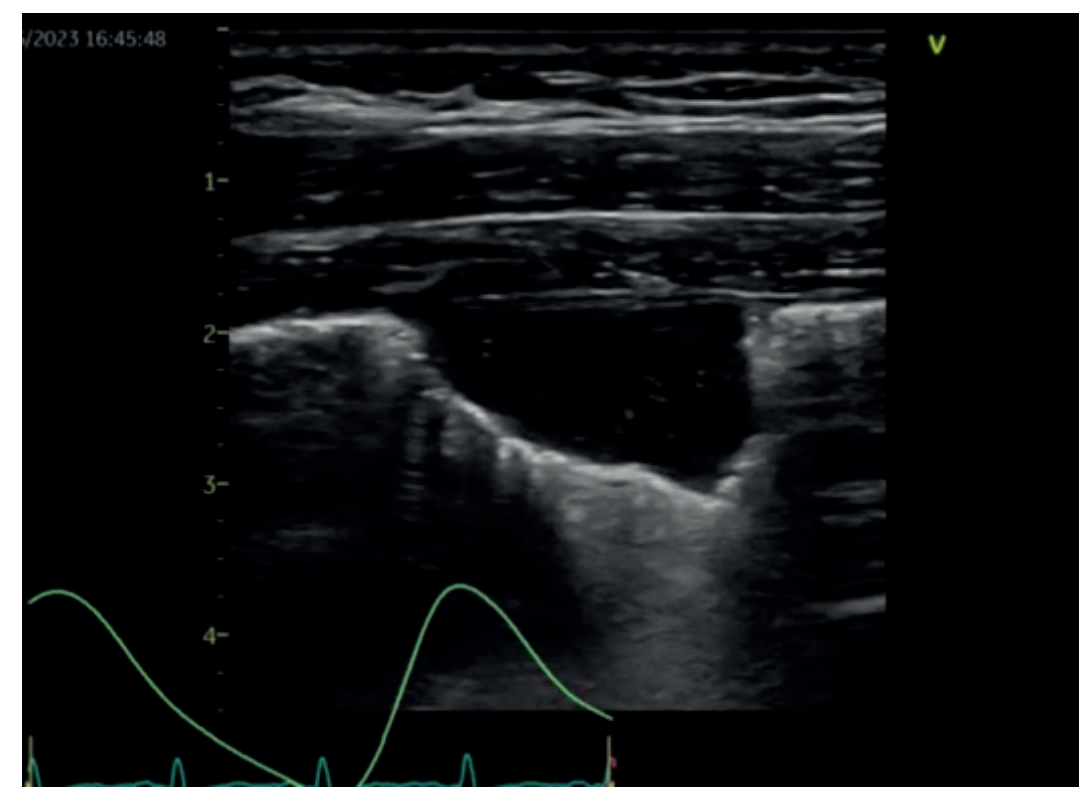

Video 25 – *Pulmonary infarction characterized by a small triangular hypoechoic subpleural lesion in a patient with pulmonary embolism. Image obtained with a linear probe. Link: http://abccardiol.org/supplementary-material/2026/12304/PosicionamentoPOCUS/Video 25 US pulm infarto pulm.mp4*

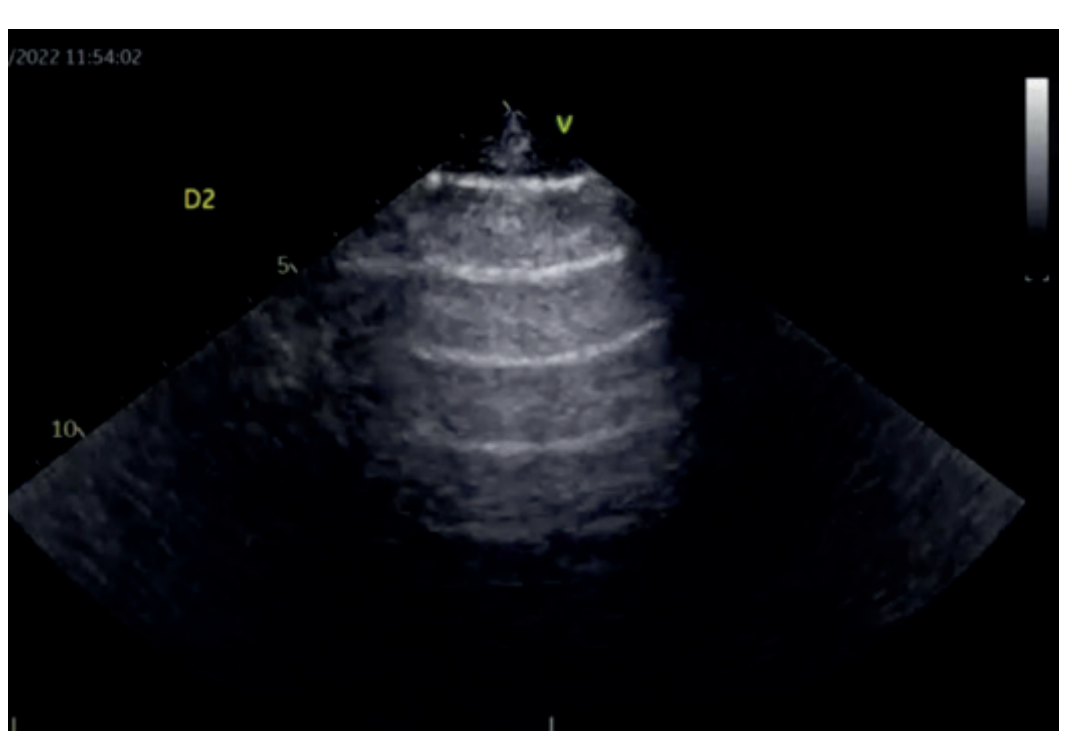

Video 26 – *A-lines characterized by horizontal reverberation artifacts arising from the pleural line, indicating aerated lung. Link: http://abccardiol.org/supplementary-material/2026/12304/PosicionamentoPOCUS/Video 26 US pulm padrao A.mp4*

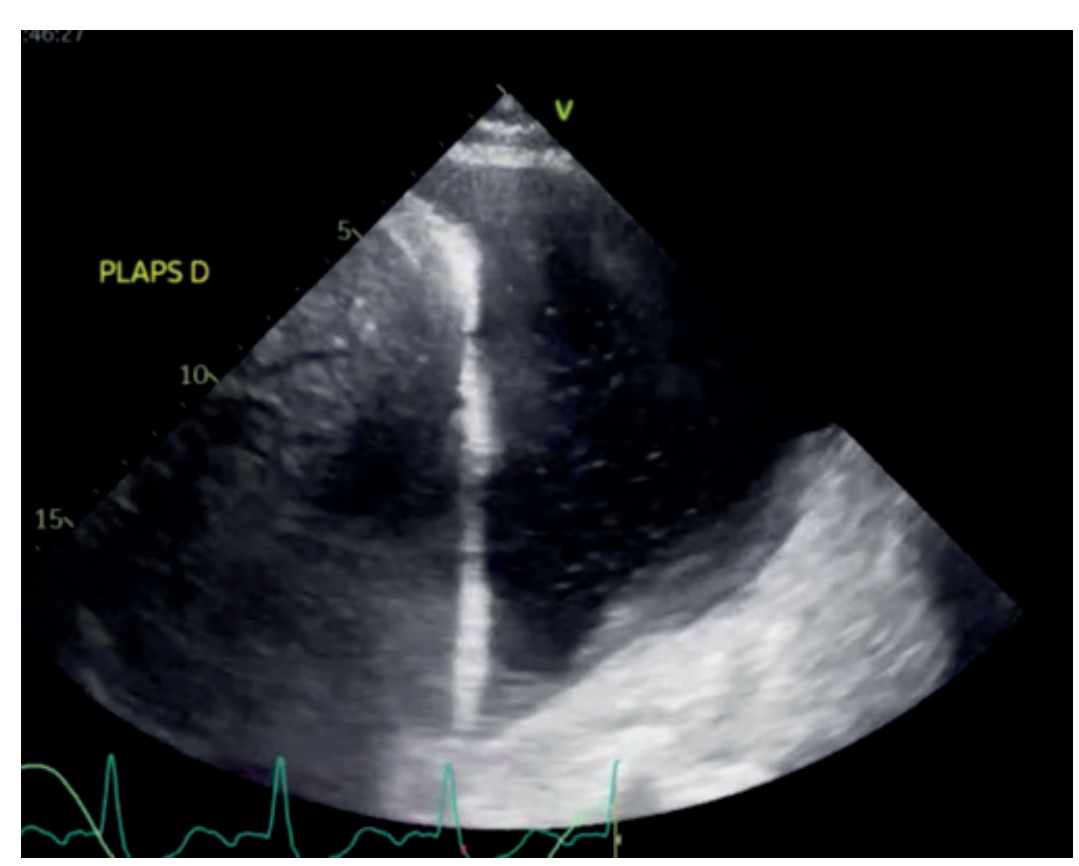

Video 27 – *Plankton sign – punctate, mobile echoes within pleural fluid. May indicate infection, hemothorax, or post-aggressive diuretic therapy. Link: http://abccardiol.org/supplementary-material/2026/12304/PosicionamentoPOCUS/Video 27 US pulm sinal plancton.mp4*

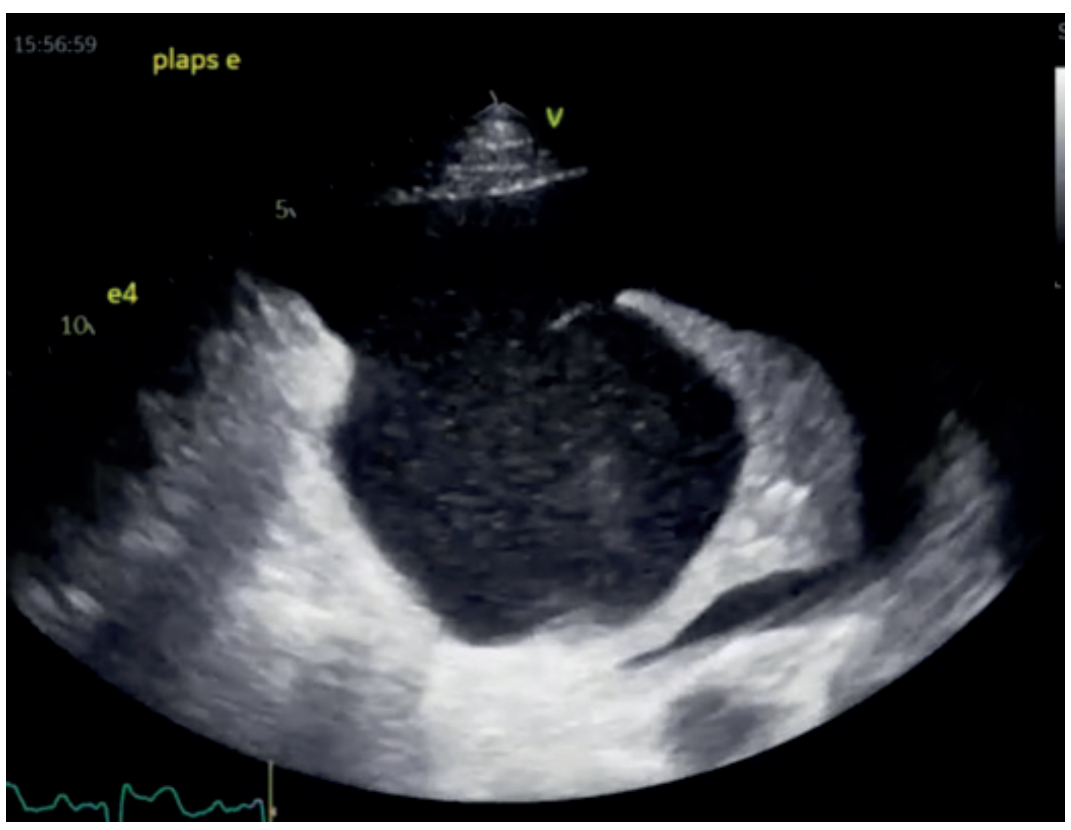

Video 28 – *Plankton sign associated with compressive atelectasis in a large pleural effusion. Link: http://abccardiol.org/supplementary-material/2026/12304/PosicionamentoPOCUS/Video 28 US pulm sinal do plancton.mp4*

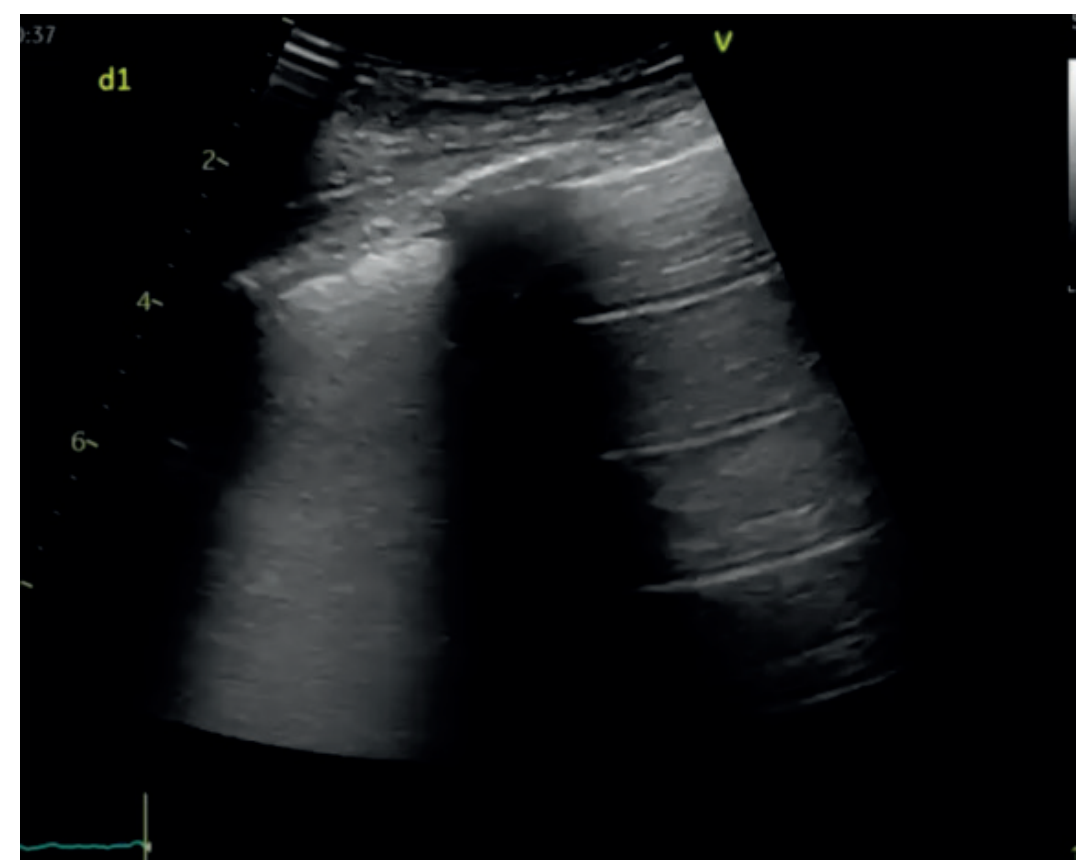

Video 29 – *B-lines with spared areas (A-lines) within the same ultrasound field in a patient with SARS-CoV-2 infection. Link: http://abccardiol.org/supplementary-material/2026/12304/PosicionamentoPOCUS/Video 29 US pulm zona poupada covid.mp4*

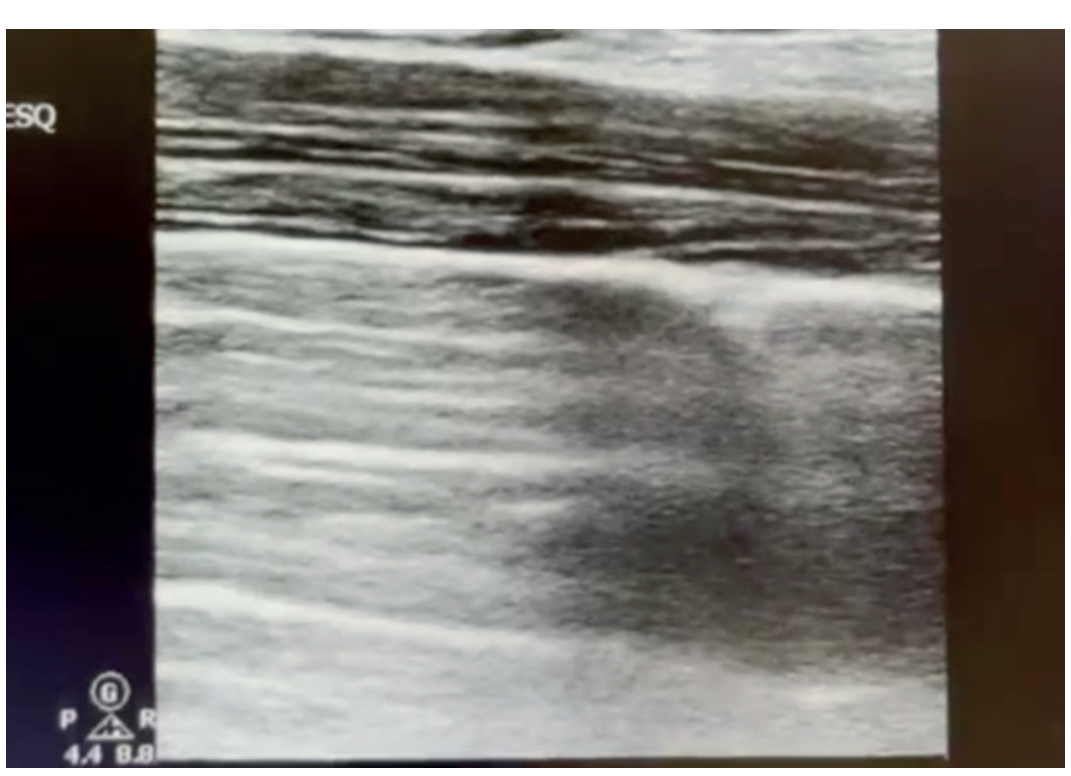

Video 30 – *Lung point – exact location of transition between collapsed lung (absent sliding) and normal lung (present sliding). Pathognomonic finding for pneumothorax. Link: http://abccardiol.org/supplementary-material/2026/12304/PosicionamentoPOCUS/Video 30 US pulm ponto pulmonar.mp4*

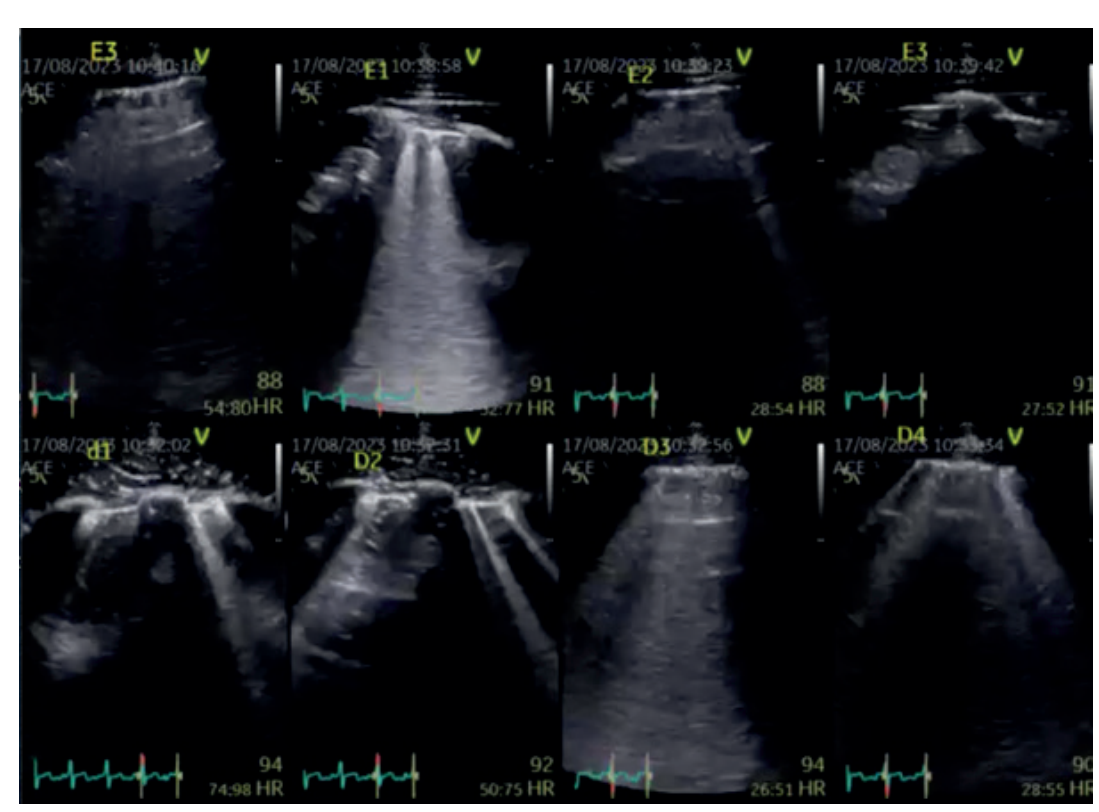

Video 31 – *Patient with decompensated heart failure presenting diffuse B-lines in all examined quadrants. Link: http://abccardiol.org/supplementary-material/2026/12304/PosicionamentoPOCUS/Video 31 US pulm linhas B difusas em todos quadrantes.mp4*

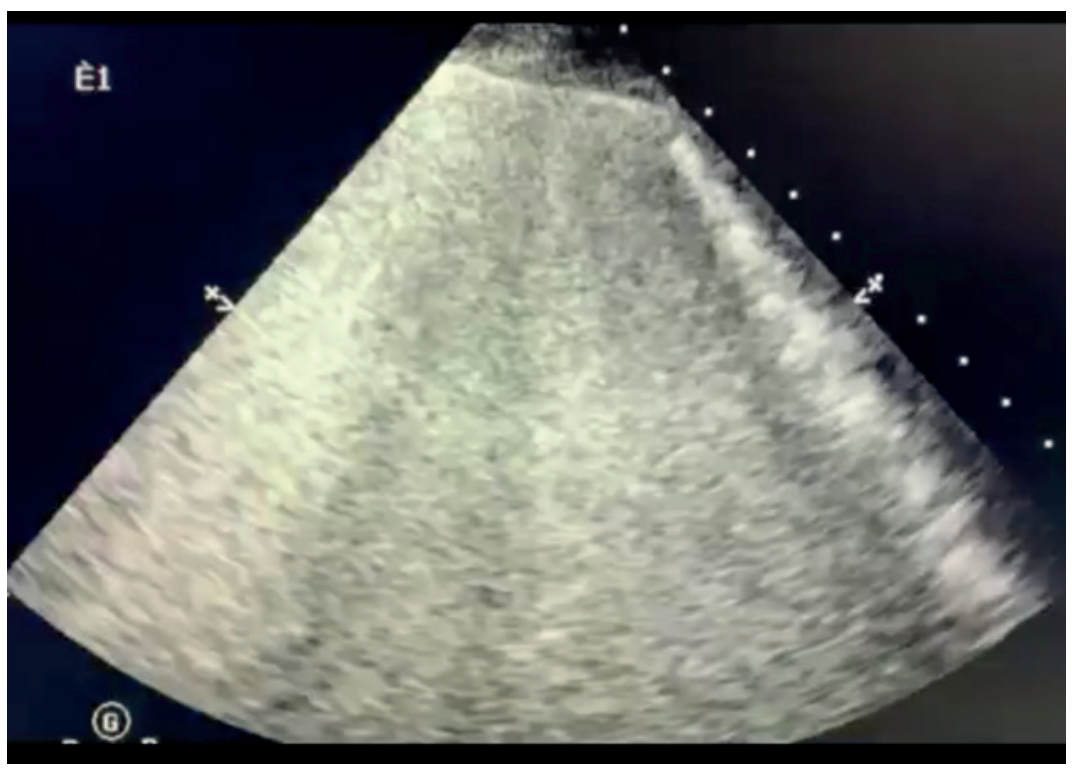

Video 32 – *Patient with decompensated heart failure presenting diffuse B-lines in the upper left quadrant, obscuring A-lines. Link: http://abccardiol.org/supplementary-material/2026/12304/PosicionamentoPOCUS/Video 32 US pulm Linhas B difusas.mp4*

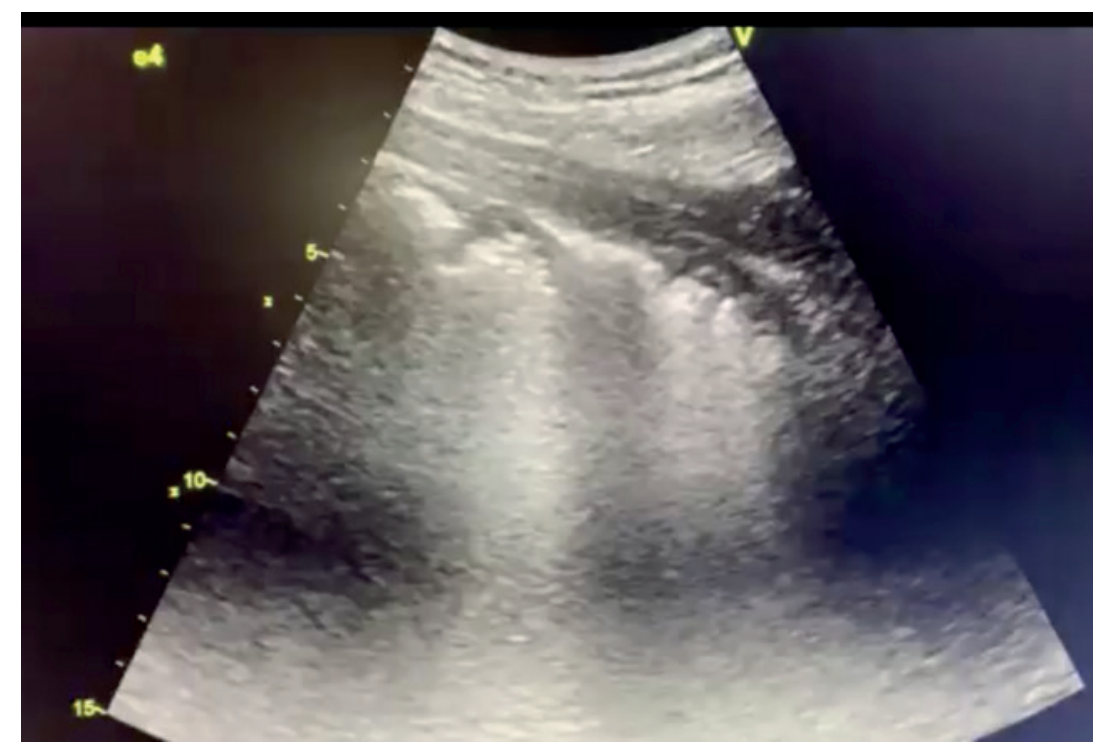

Video 33 – *Subpleural consolidations in a patient with SARS-CoV-2 infection. Link: http://abccardiol.org/supplementary-material/2026/12304/PosicionamentoPOCUS/Video 33 US pulm consolidações subpleurais.mp4*

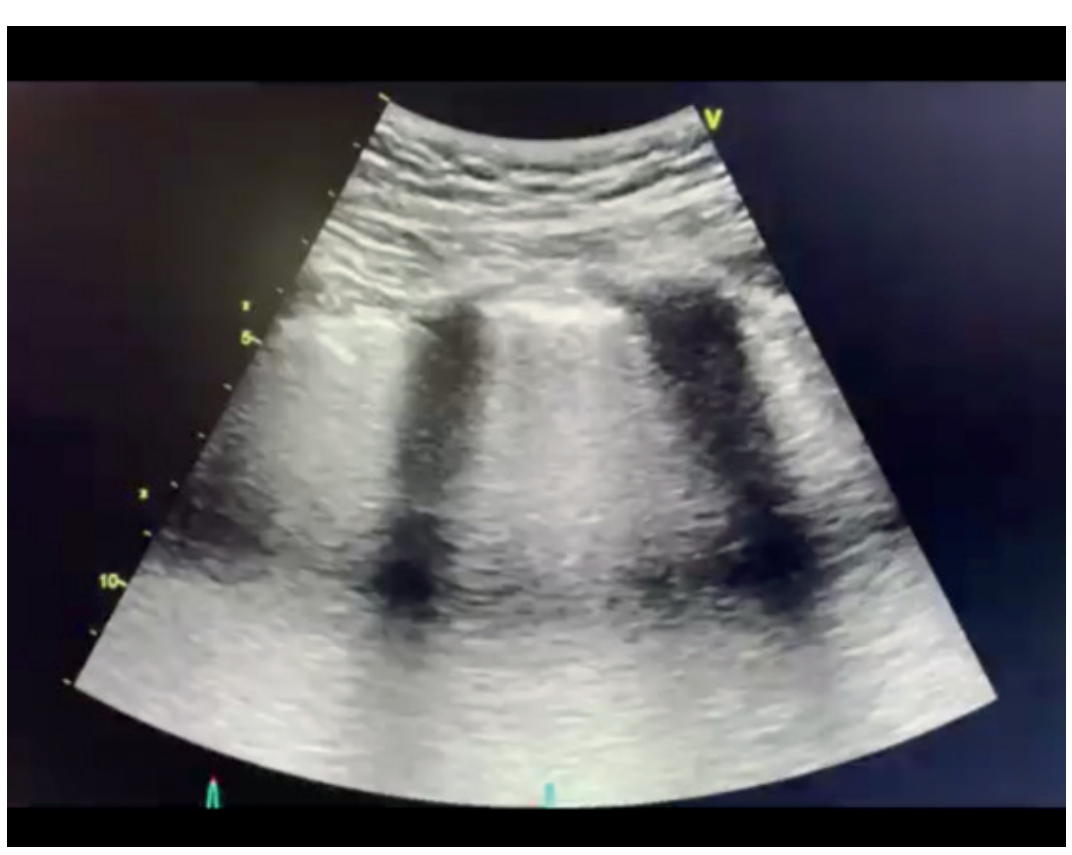

Video 34 – *Coalescent B-lines in a patient with SARS-CoV-2 infection. Link: http://abccardiol.org/supplementary-material/2026/12304/PosicionamentoPOCUS/Video 34 US pulm linhas B coalescentes.mp4*

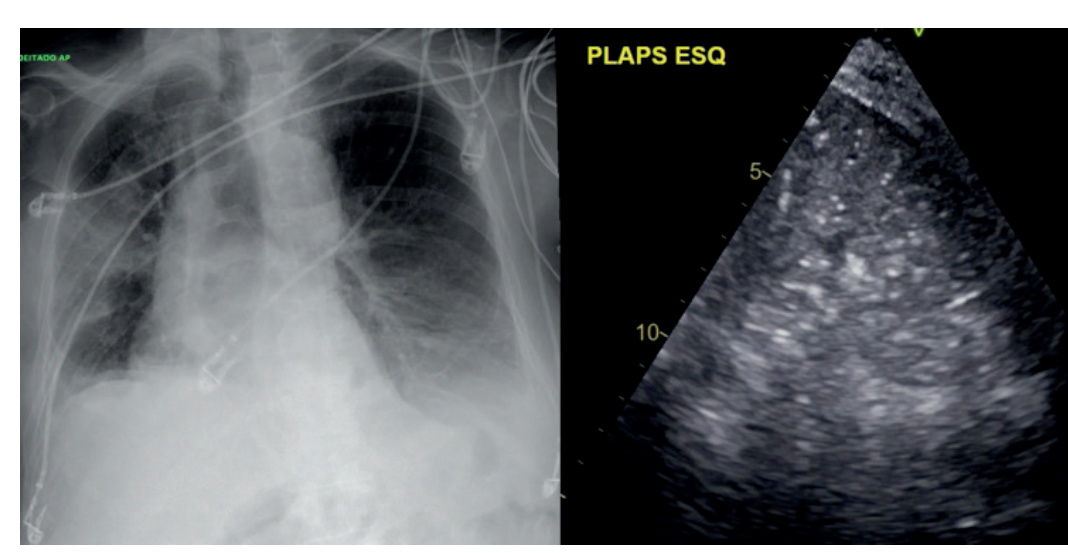

Video 35 – *Consolidation of the left lung base with air bronchogram and its radiologic equivalent. Link: http://abccardiol.org/supplementary-material/2026/12304/PosicionamentoPOCUS/Video 35 US pulm consolidação pulm com broncograma aereo.mp4*

Statement

Supplement 2

Compiled Videos

SECTION 4 – VExUS

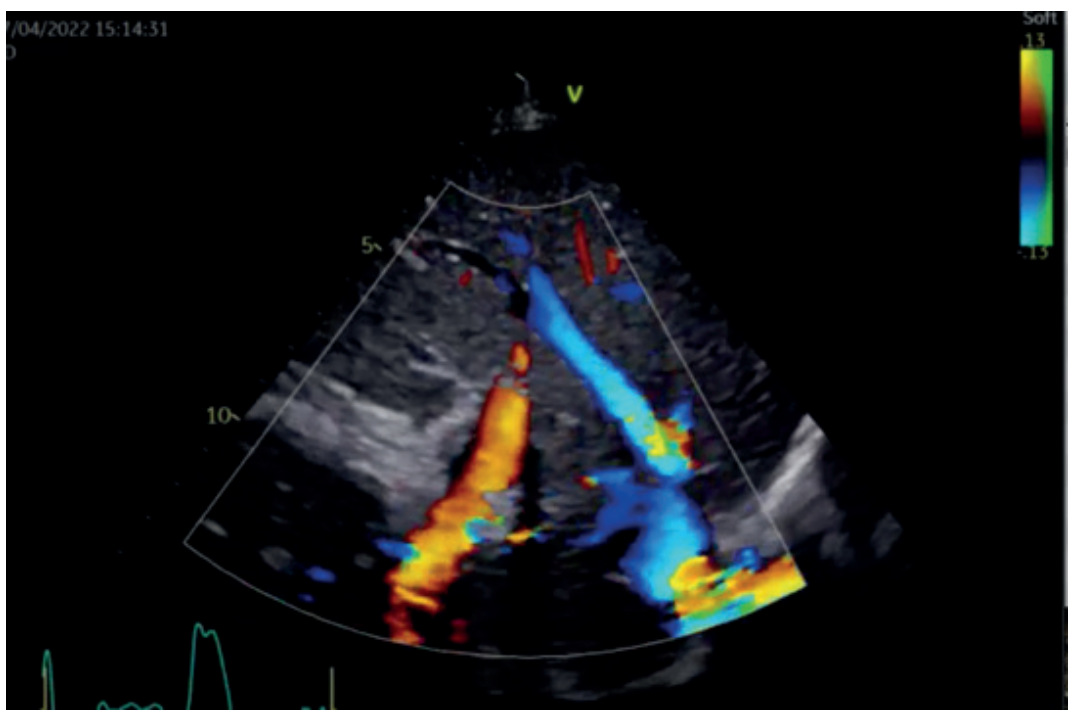

Video 36 – *Venous Excess Ultrasound Score transhepatic window normal pattern. Hepatic vein (blue flow) and portal vein (red flow). Link: http://abccardiol.org/supplementary-material/2026/12304/PosicionamentoPOCUS/Video 36 VEXUS Janela transhepática padrão normal.mp4*

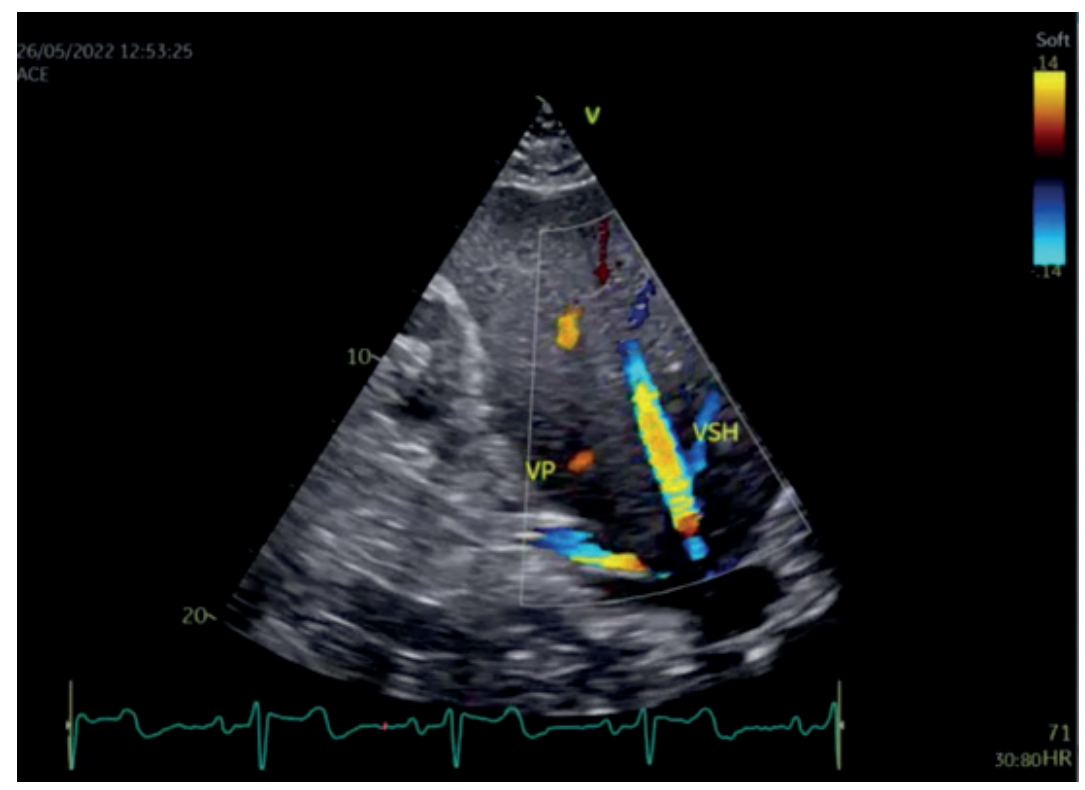

Video 37 – *Venous Excess Ultrasound Score transhepatic window normal pattern with tilting movement. Normal transhepatic window with anterior tilting of the transducer to visualize the portal vein (red). Link: http://abccardiol.org/supplementary-material/2026/12304/PosicionamentoPOCUS/Video 37 VEXUS Janela transhepática padrão normal.mp4*

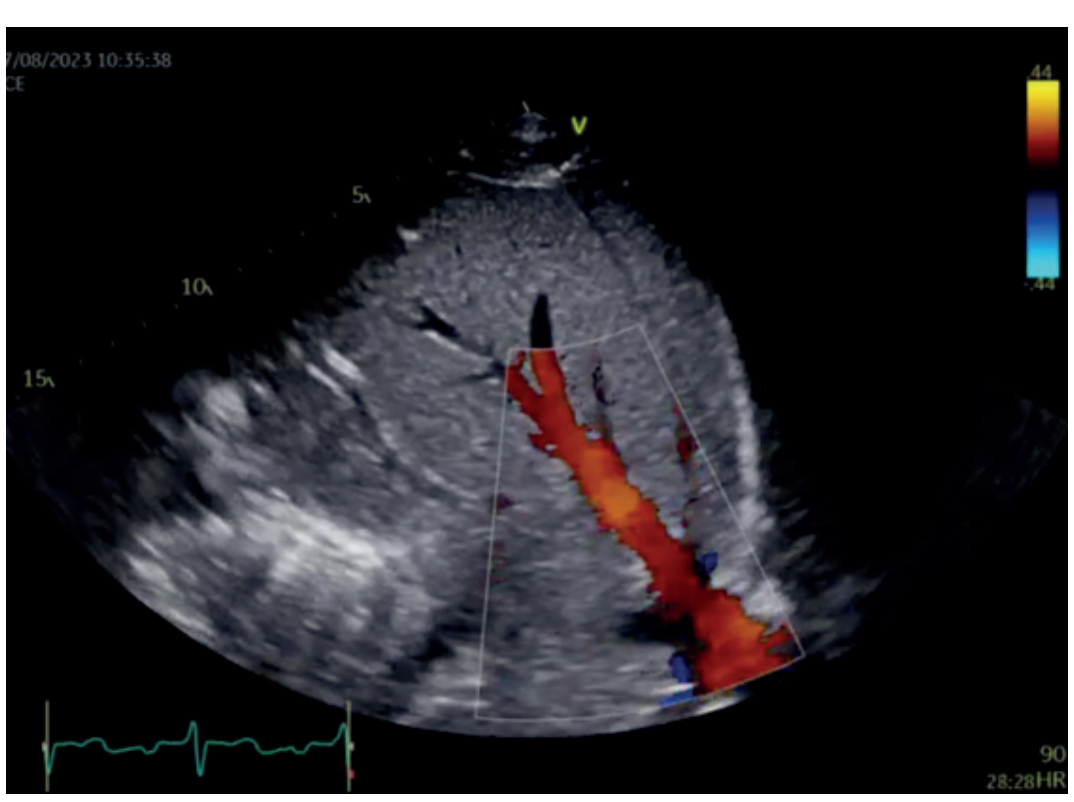

Video 38 – *Venous Excess Ultrasound Score (VExUS) transhepatic window abnormal pattern. Abnormal transhepatic VExUS pattern with hepatic vein showing systolic flow reversal (red), equivalent to systolic reversal on spectral Doppler. Link: http://abccardiol.org/supplementary-material/2026/12304/PosicionamentoPOCUS/Video 38 VEXUS Janela transhepática padrão alterado.mp4*

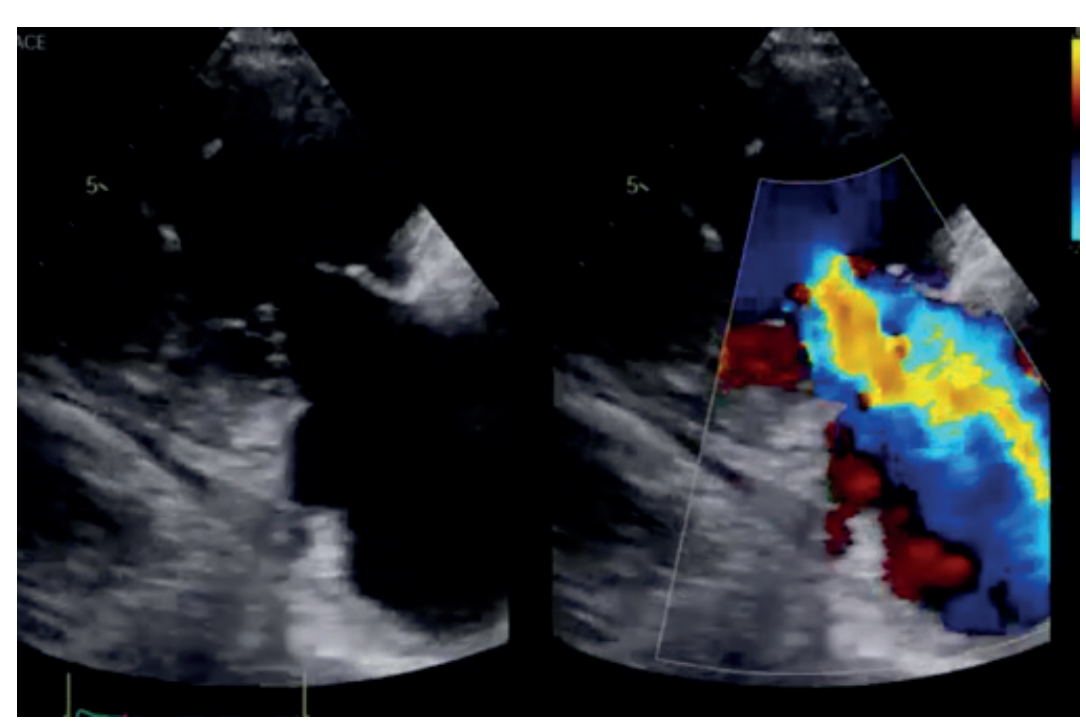

Video 39 – *Venous Excess Ultrasound Score torrential tricuspid regurgitation 1. Severe tricuspid regurgitation causing significant systemic venous congestion. Link: http://abccardiol.org/supplementary-material/2026/12304/PosicionamentoPOCUS/Video 39 VEXUS IT torrencial1.mp4*

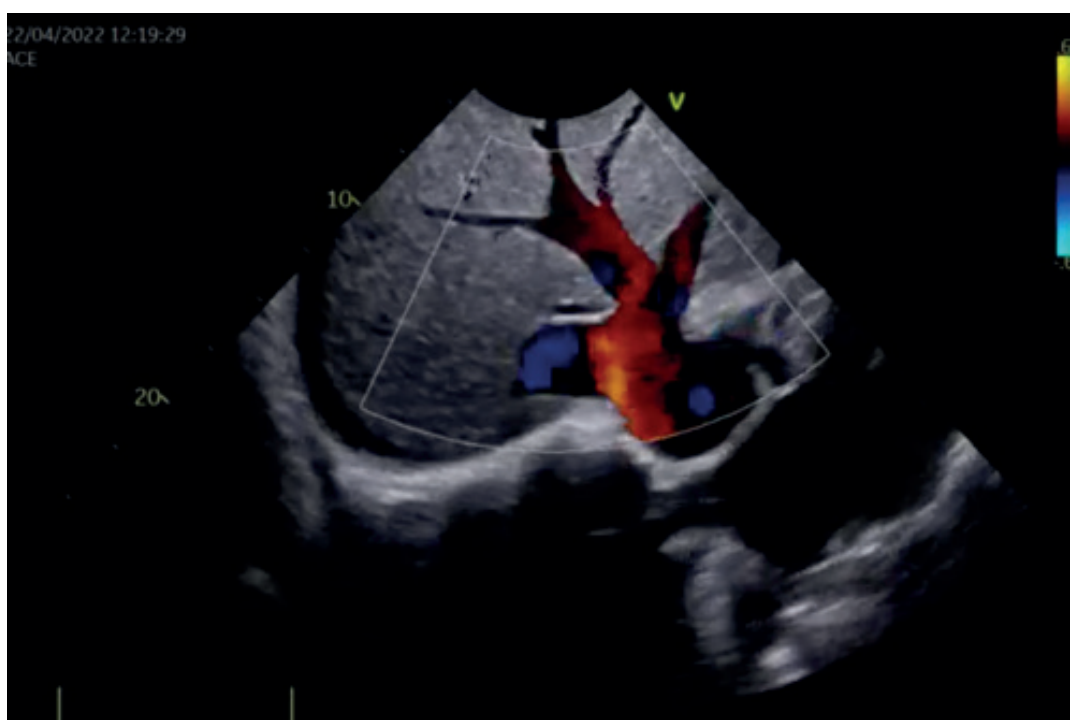

Video 40 – *Venous Excess Ultrasound Score torrential tricuspid regurgitation 2. Severe tricuspid regurgitation causing marked systemic venous congestion, characterized by markedly dilated inferior vena cava and hepatic veins with reversed flow, along with ascites. Link: http://abccardiol.org/supplementary-material/2026/12304/PosicionamentoPOCUS/Video 40 VEXUS IT torrencial2.mp4*

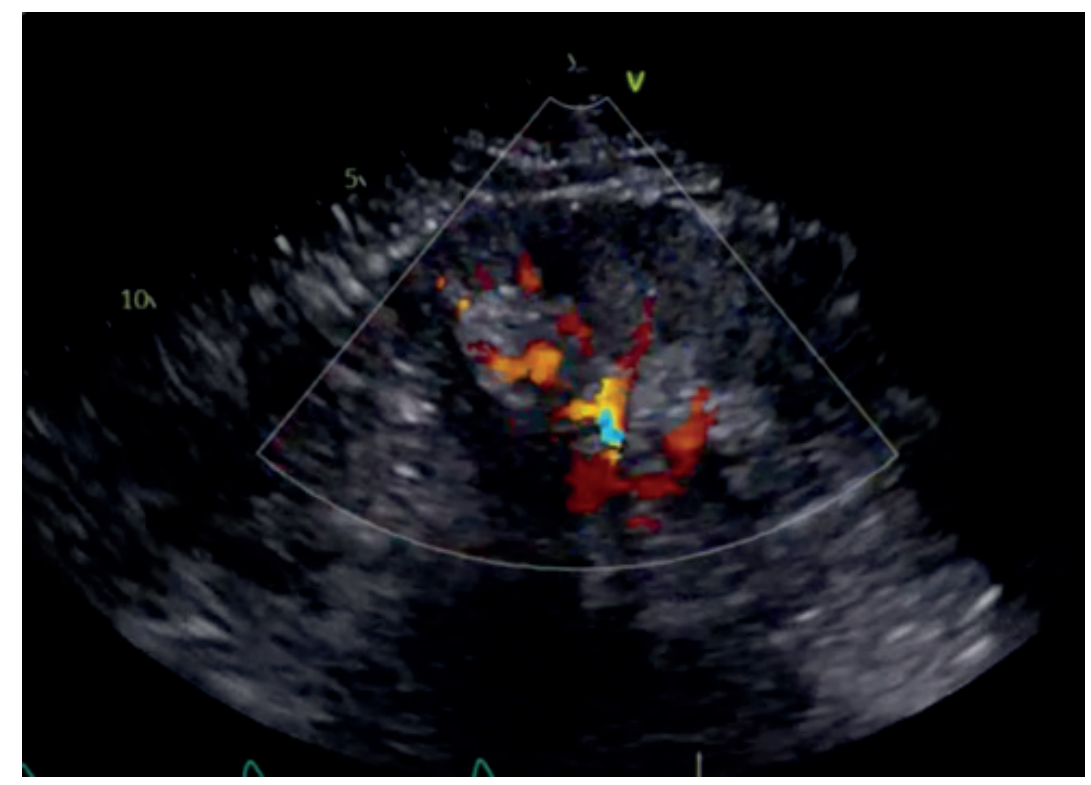

Video 41 – *Venous Excess Ultrasound Score renal lobar vein flow. Renal intraparenchymal flow with recording of lobar renal vein flow at the corticomedullary junction. Link: http://abccardiol.org/supplementary-material/2026/12304/PosicionamentoPOCUS/Video 41 VEXUS Fluxo veia lobar renal.mp4*

Supplement 2

Compiled Videos

SECTION 5 – POCUS in deep vein thrombosis

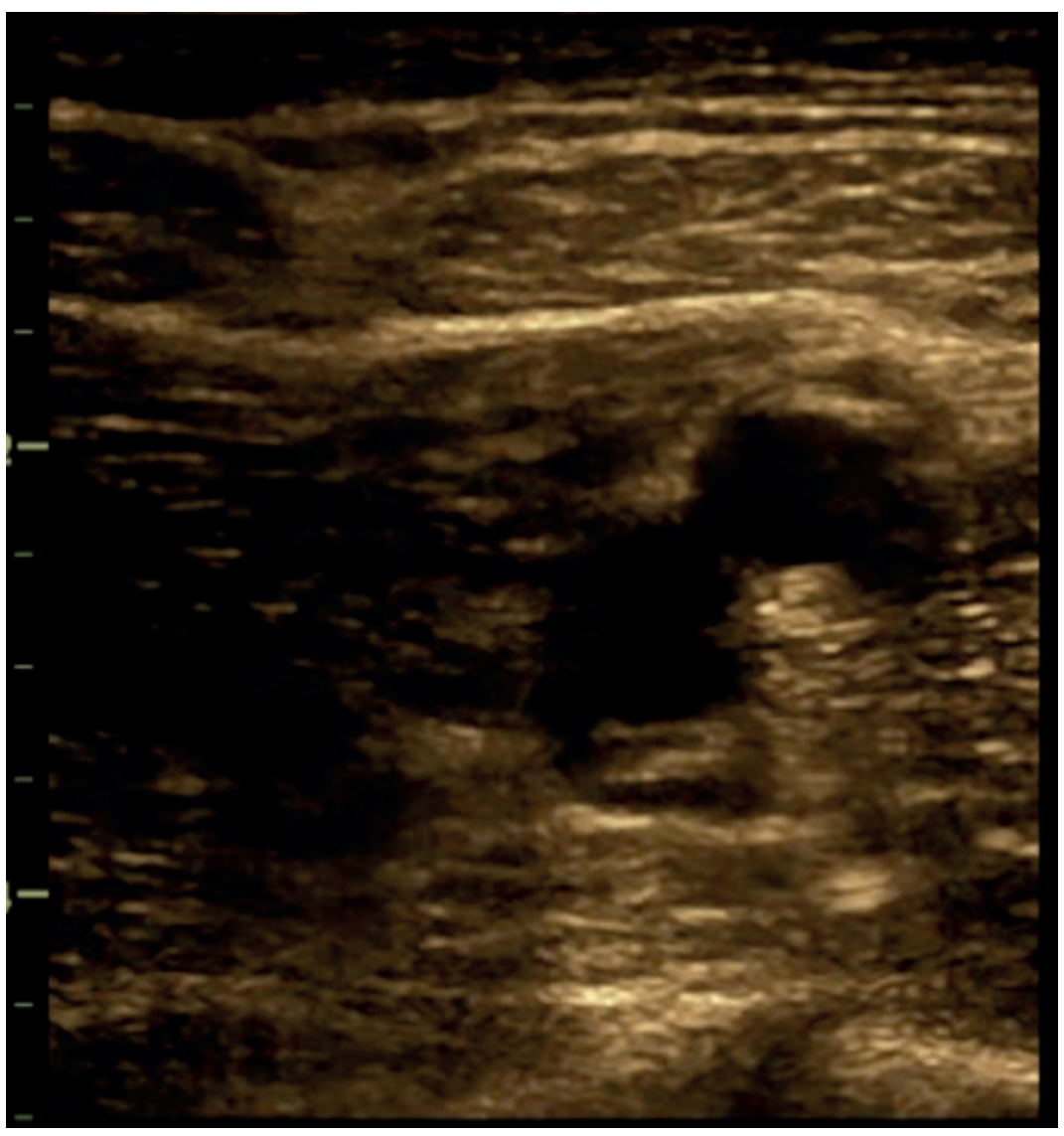

Video 42 – *Femoral vein compression. Compression protocol for deep vein thrombosis investigation. Link: http://abccardiol.org/supplementary-material/2026/12304/PosicionamentoPOCUS/Video 42 veia femoral com compressão.mp4*

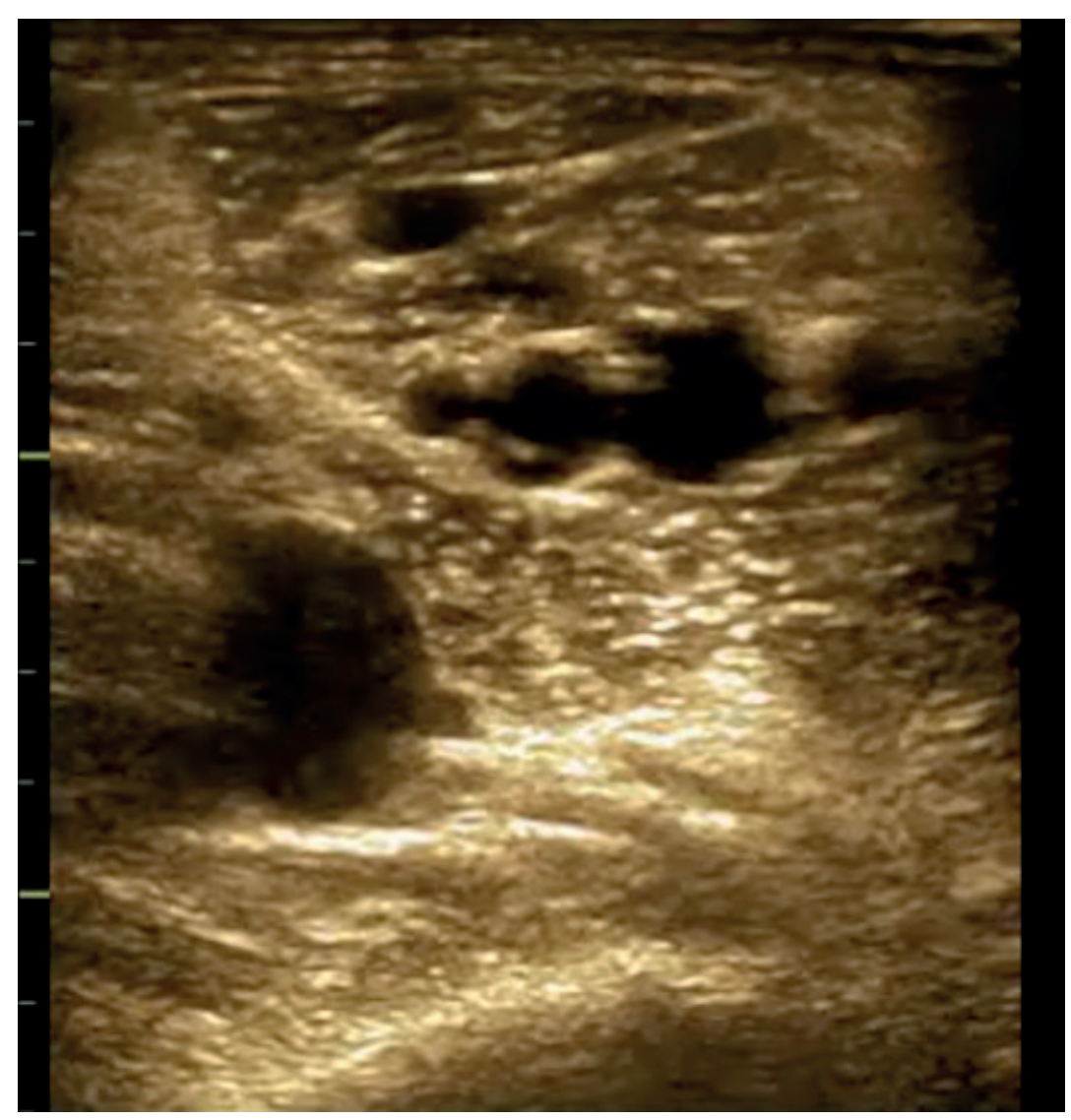

Video 43 – *Gastrocnemius compression popliteal deep vein thrombosis (DVT). Compression protocol for DVT investigation. Compression of calf muscular veins without thrombosis and, in a deeper plane, a non-compressible popliteal vein with thrombosis. Link: http://abccardiol.org/supplementary-material/2026/12304/PosicionamentoPOCUS/Video 43 gastrocnemia com compressão TVP poplitea.mp4*

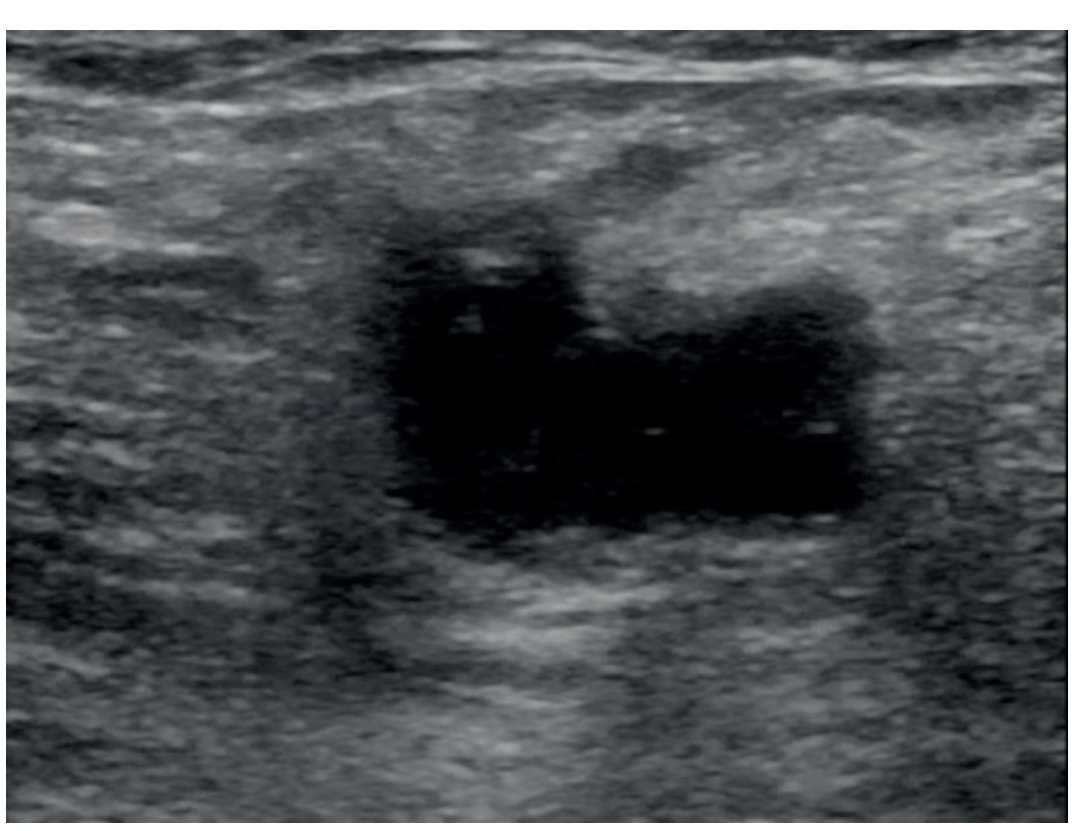

Video 44 – *Femoral deep vein thrombosis compression. Compression protocol showing a femoral vein non-compressible due to thrombus. Link: http://abccardiol.org/supplementary-material/2026/12304/PosicionamentoPOCUS/Video 44 TVP femoral compressão.mp4*

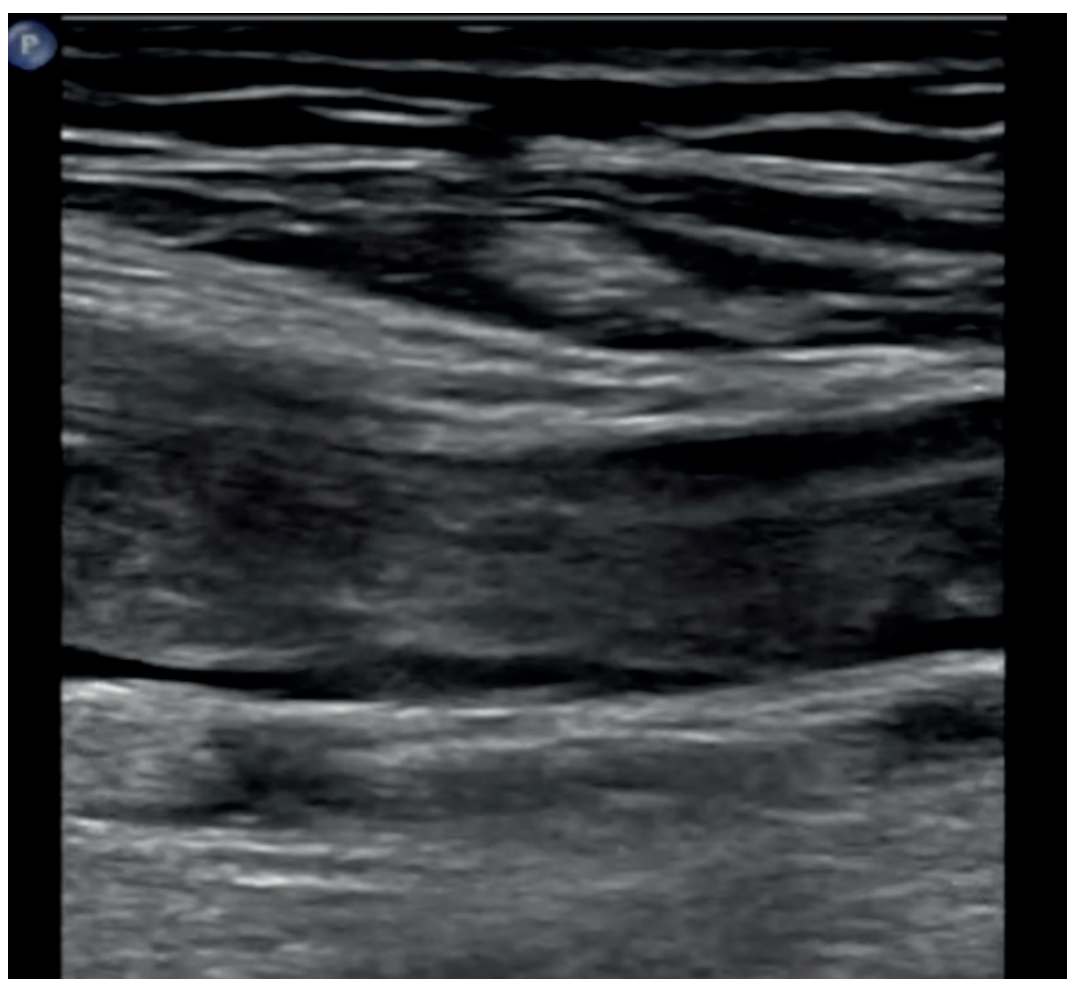

Video 45 – *Floating thrombus femoral vein. Floating thrombus in the femoral vein, longitudinal axis. Link: http://abccardiol.org/supplementary-material/2026/12304/PosicionamentoPOCUS/Video 45 Trombo flutuante veia femoral.mp4*

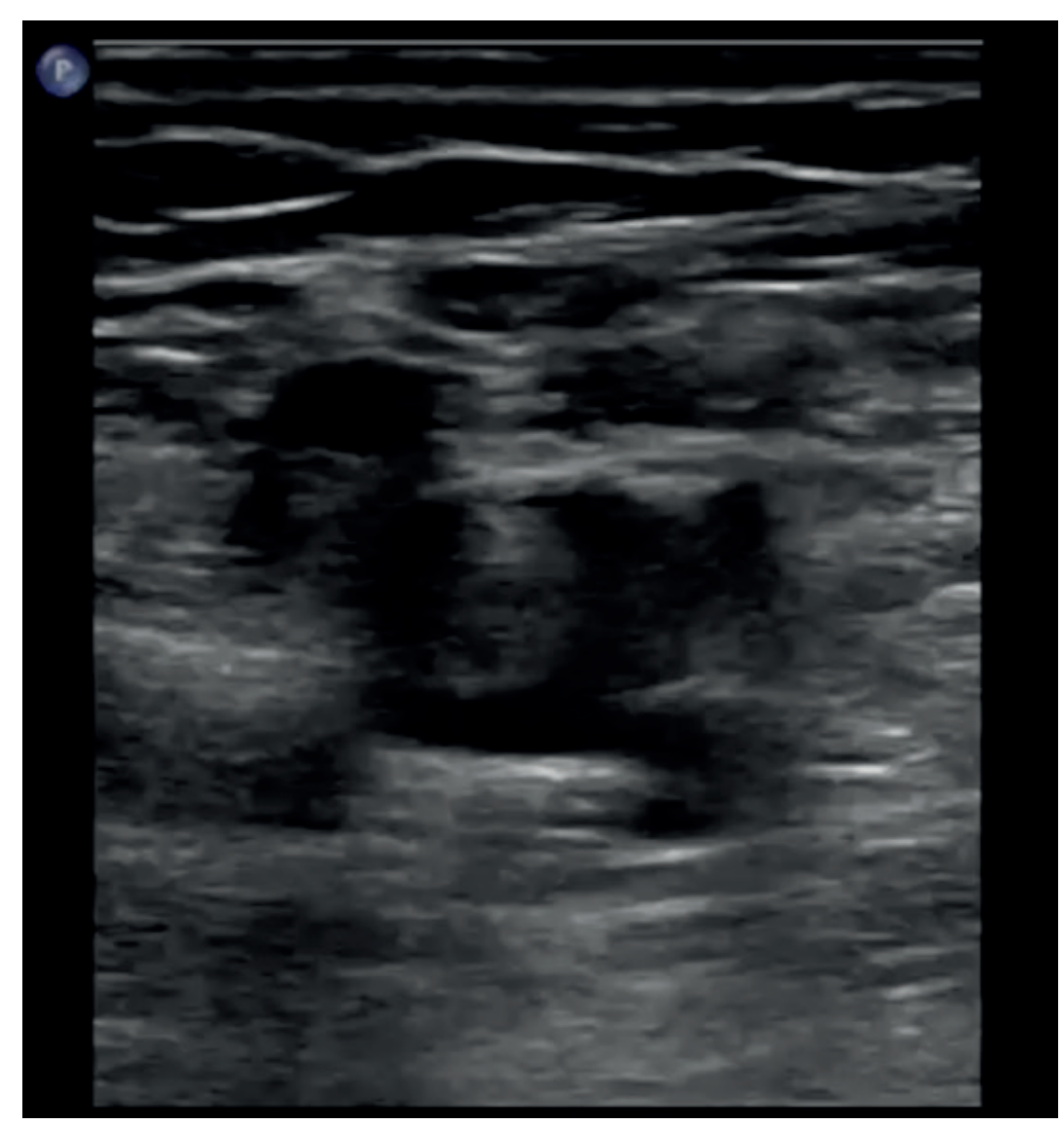

Video 46 – *Floating thrombus femoral vein short axis. Floating thrombus in the femoral vein, short-axis view. Link: http://abccardiol.org/supplementary-material/2026/12304/PosicionamentoPOCUS/Video 46 Trombo flutuante veia femoral eixo curto.mp4*

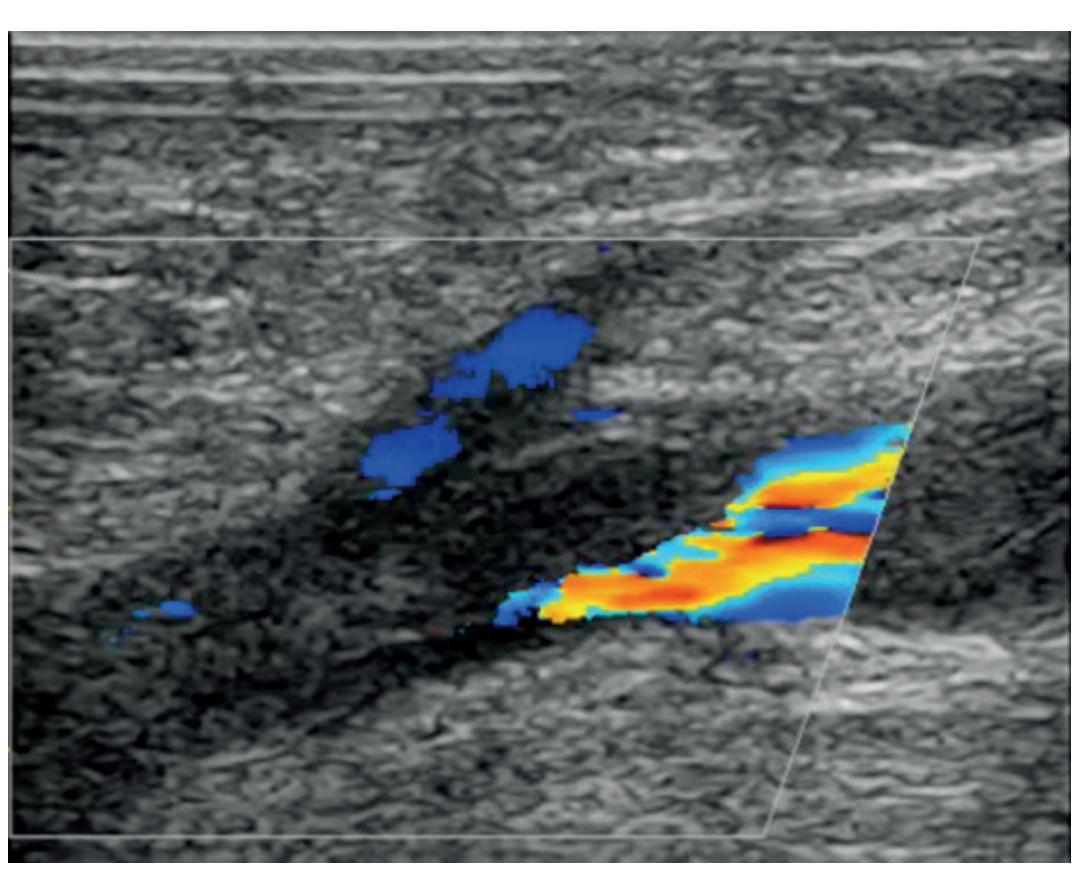

Video 47 – *Femoral deep vein thrombosis color Doppler. Mobile thrombus in the femoral vein with flow seen around it on color Doppler. Link: http://abccardiol.org/supplementary-material/2026/12304/PosicionamentoPOCUS/Video 47 TVP veia femoral color.mp4*

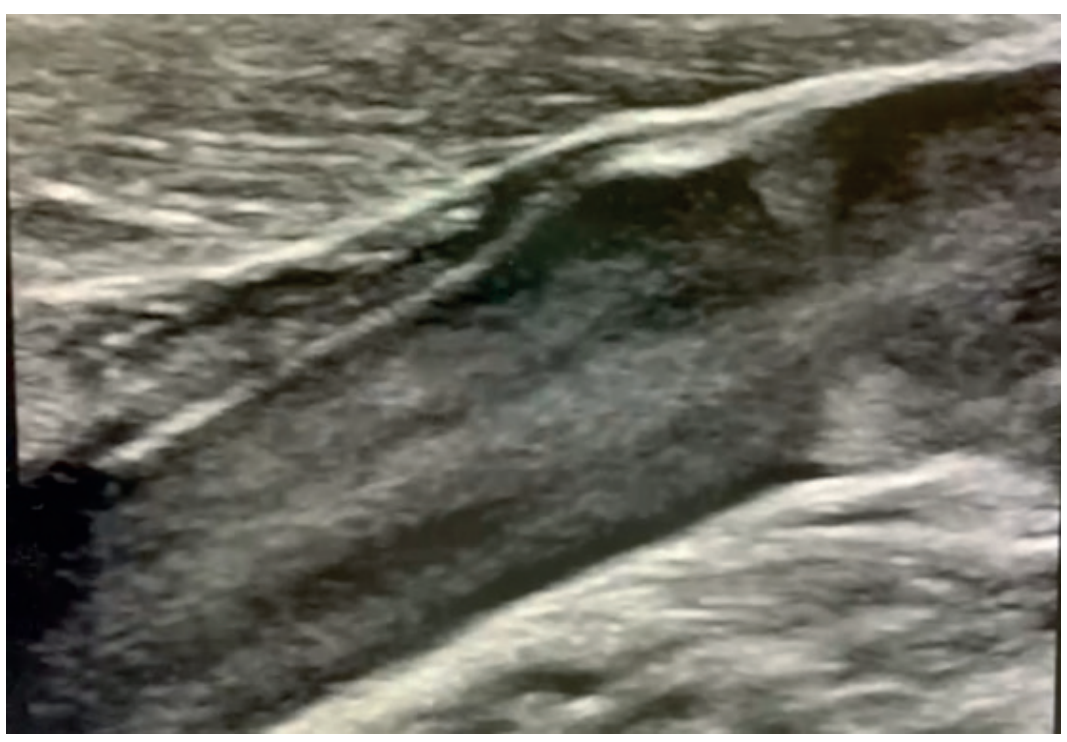

Video 48 – *Venous duplex with venous stasis. Venous stasis mimicking thrombus, especially at the valve level. Link: http://abccardiol.org/supplementary-material/2026/12304/PosicionamentoPOCUS/Video 48 Duplex venoso com estase venosa.MP4*

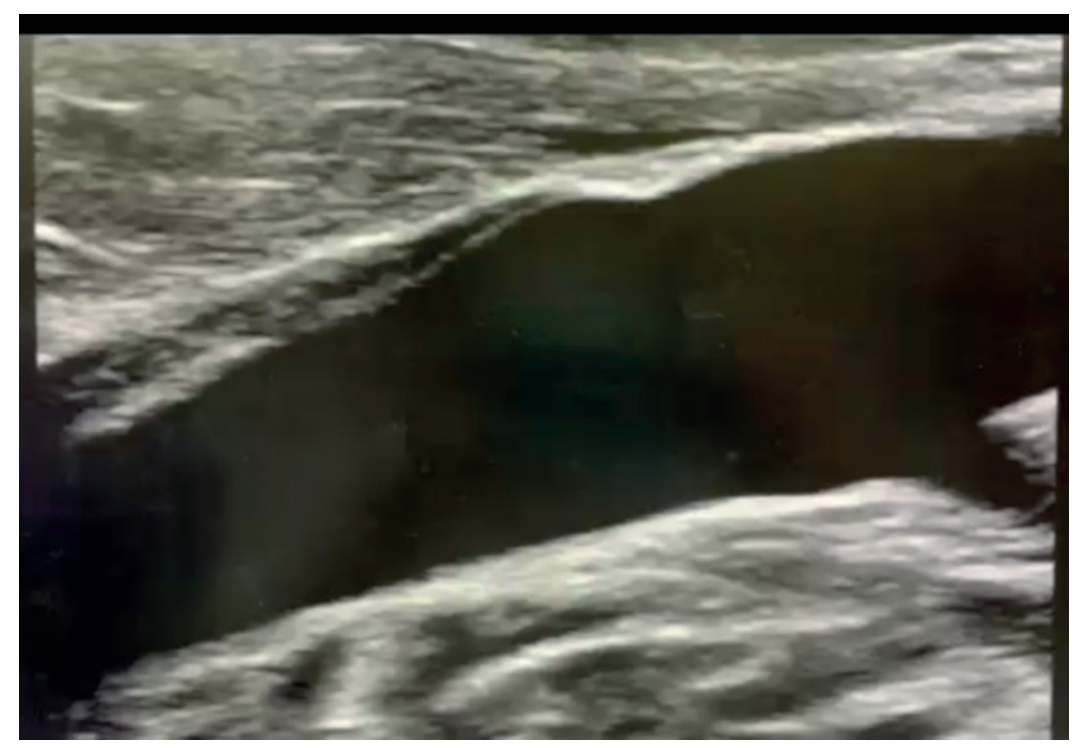

Video 49 – *Duplex after compression without venous stasis. Disappearance of venous stasis after compression with the transducer. Link: http://abccardiol.org/supplementary-material/2026/12304/PosicionamentoPOCUS/Video 49 Duplex apos compressão sem estase venosa.mp4*

Supplement 2

Compiled Videos

SECTION 6 – POCUS in shock

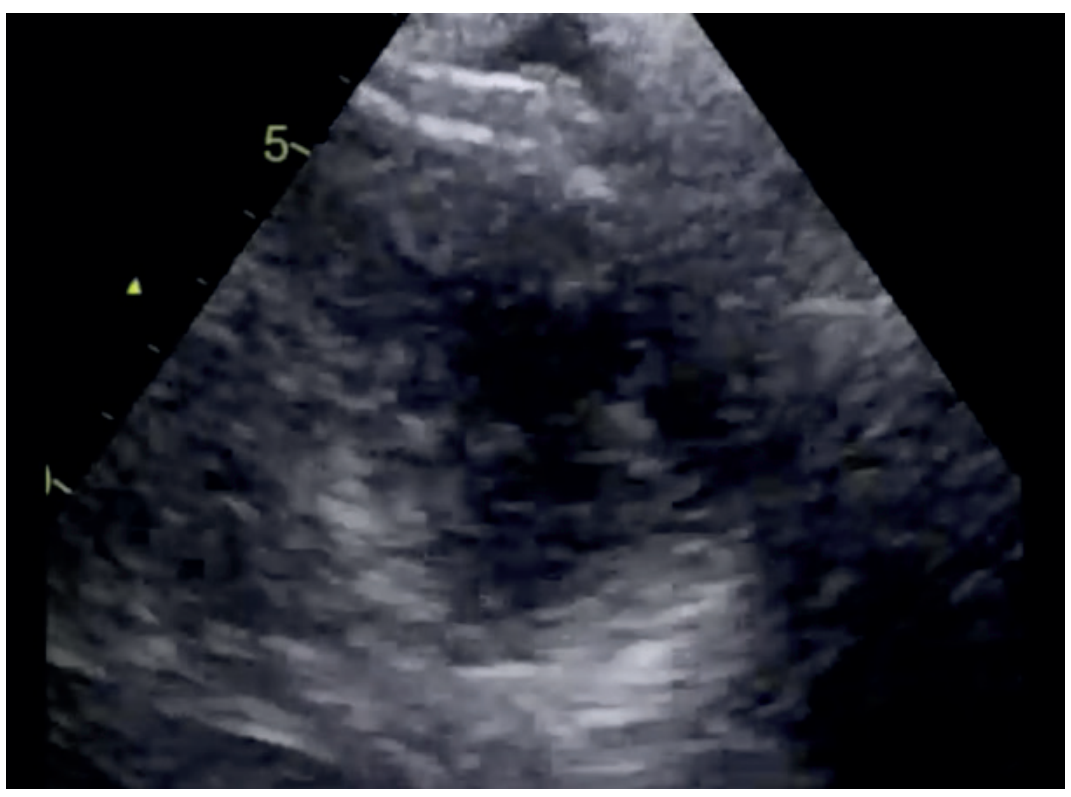

Video 50 – *Septic shock hyperdynamic left ventricle short axis kissing walls. Hyperdynamic left ventricle in the early phase of septic shock, with reduced ventricular cavities and approximation of papillary muscles and walls (“*kissing walls*”). Link: http://abccardiol.org/supplementary-material/2026/12304/PosicionamentoPOCUS/Video 50 Choque septico VE hiperdinamico eixo curto kissing walls.mp4*

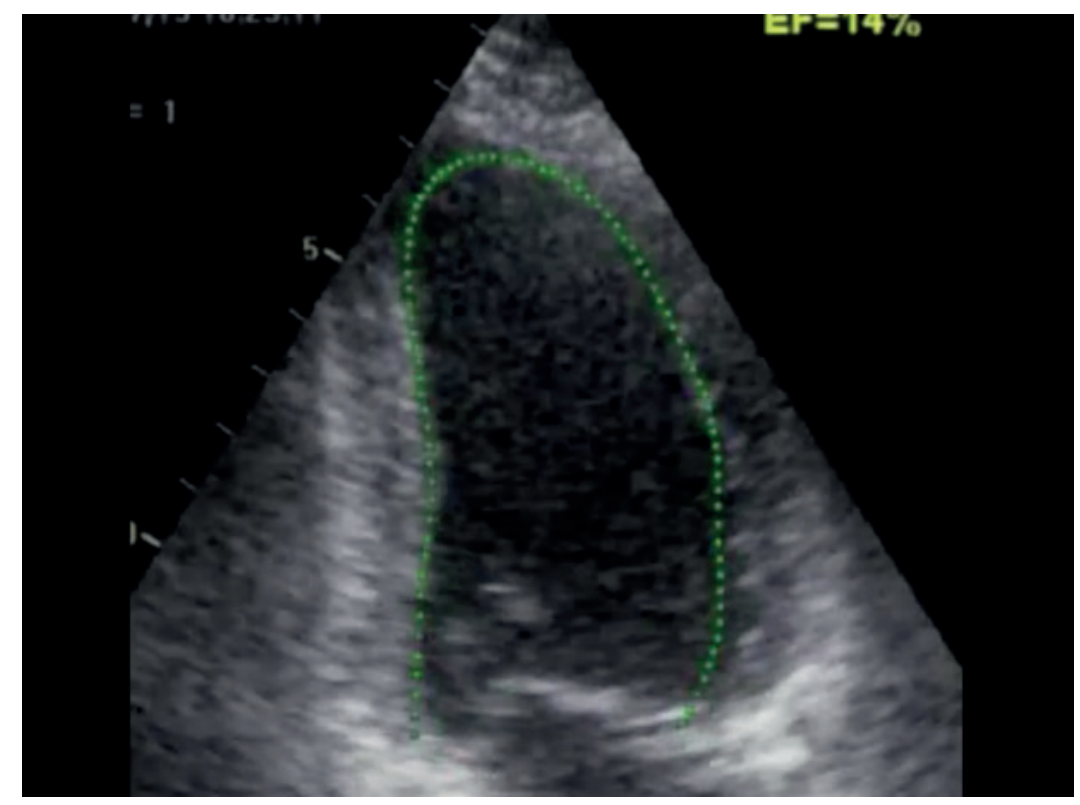

Video 51 – *Cardiogenic shock severe left ventricular dysfunction. Patient admitted with SARS-CoV-2 presenting cardiogenic shock due to acute myocardial infarction complicated by stress cardiomyopathy (Takotsubo). Examination performed prior to venoarterial extracorporeal membrane oxygenation initiation. Link: http://abccardiol.org/supplementary-material/2026/12304/PosicionamentoPOCUS/Video 51 Choque cardiogênico disfunção grave VE.mp4*

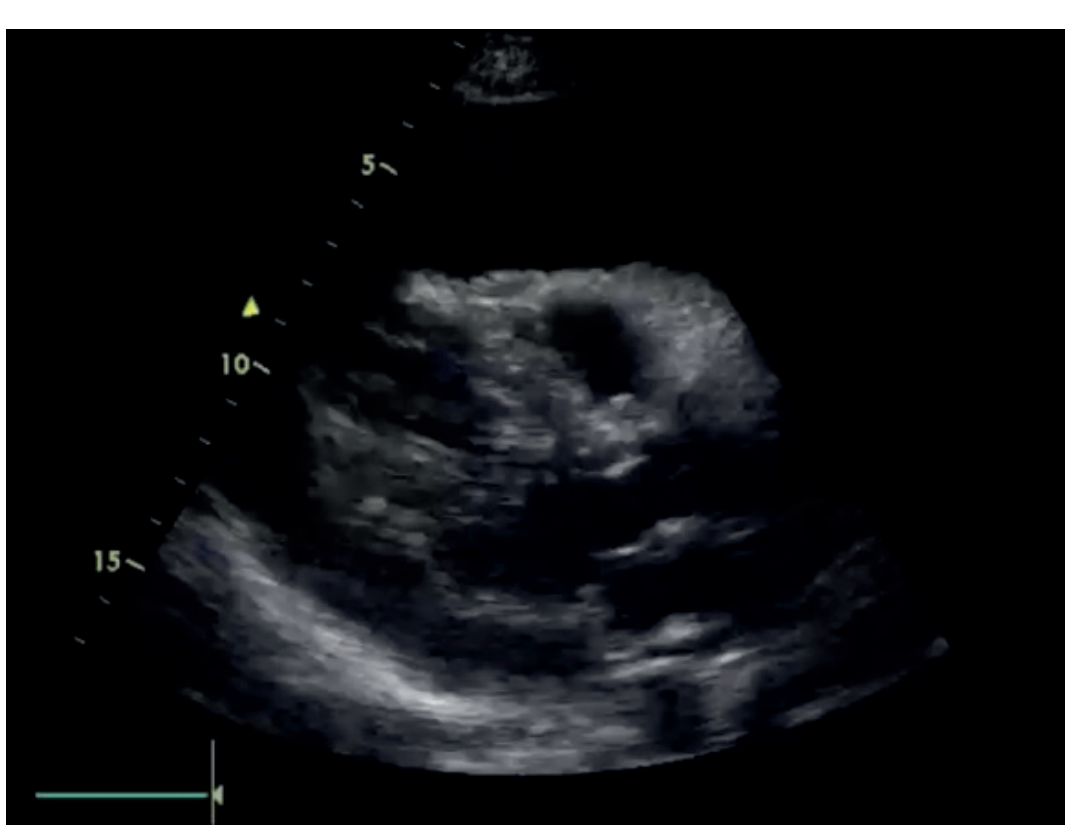

Video 52 – *Large pericardial effusion with swinging heart. Large pericardial effusion producing oscillatory cardiac motion within the pericardial sac (*swinging heart*). Link: http://abccardiol.org/supplementary-material/2026/12304/PosicionamentoPOCUS/Video 52 Derrame pericardico volumoso com swinging heart.mp4*

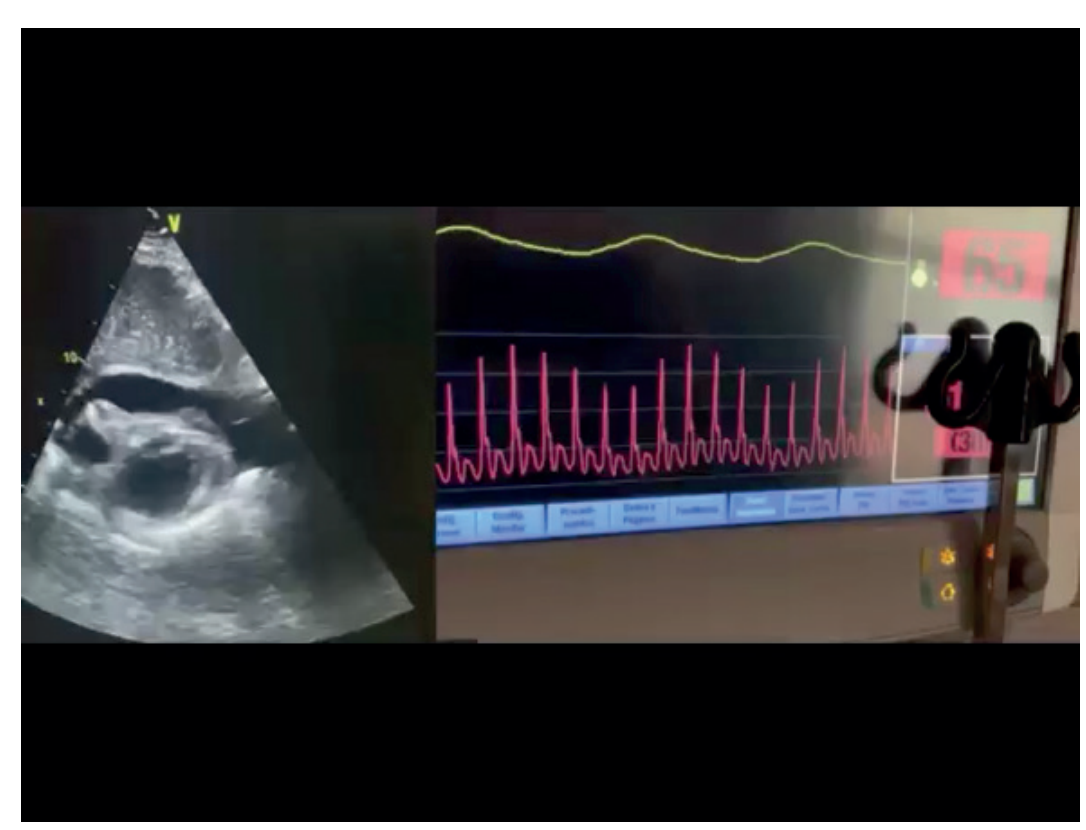

Video 53 – *Obstructive shock cardiac tamponade. Patient hospitalized with SARS-CoV-2 evolves to shock. Echocardiography shows large pericardial effusion with collapse of right-sided chambers and inferior vena cava distension. Electrical alternans observed on monitoring due to swinging heart, with systolic blood pressure < 40 mm Hg. Link: http://abccardiol.org/supplementary-material/2026/12304/PosicionamentoPOCUS/Video 53 Choque obstrutivo tamponamento cardiaco.mp4*

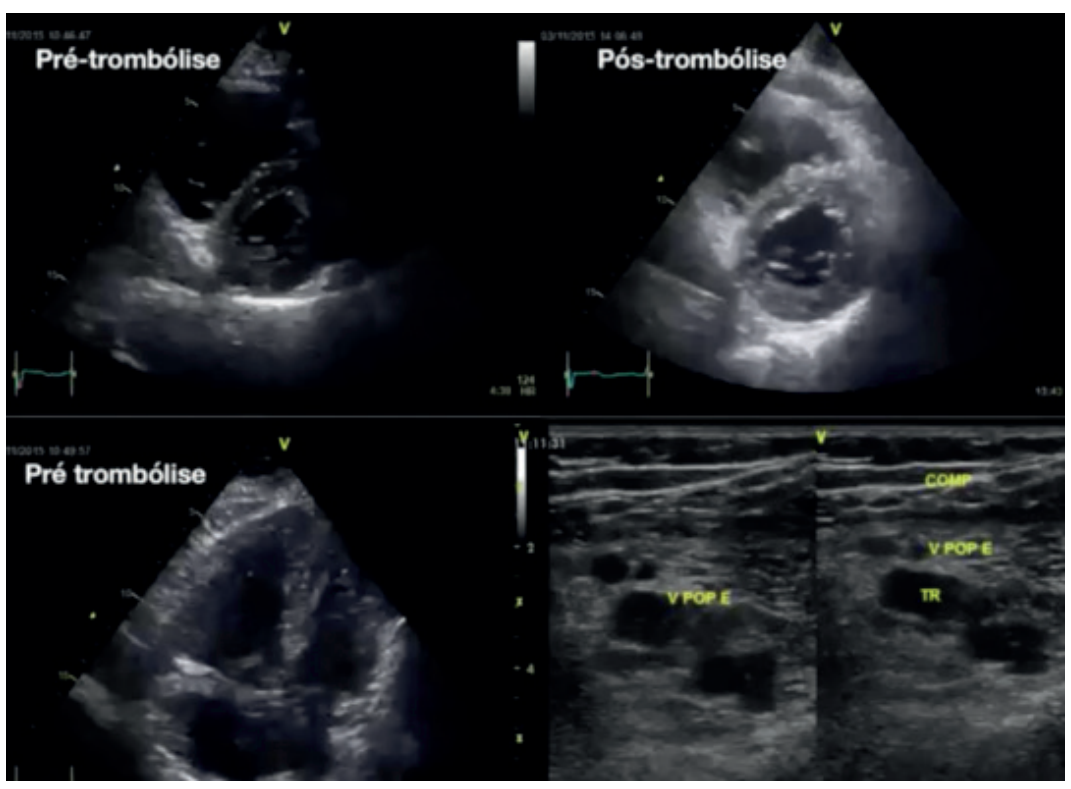

Video 54 – *Obstructive shock massive pulmonary embolism thrombolysis. Patient admitted to the emergency department with tachydyspnea, diaphoresis, and cold extremities. Blood pressure: 80×50 mm Hg; heart rate: 120 bpm; SpO_2: 89%; Troponin: 0.337 ng/mL. Echocardiography shows right ventricular dilation and dysfunction with septal flattening and McConnell sign. Computed tomography angiography confirmed massive pulmonary embolism. Patient underwent systemic thrombolysis with alteplase, with resolution of findings. Link: http://abccardiol.org/supplementary-material/2026/12304/PosicionamentoPOCUS/Video 54 Choque obstrutivo TEP maciço trombólise.mp4*

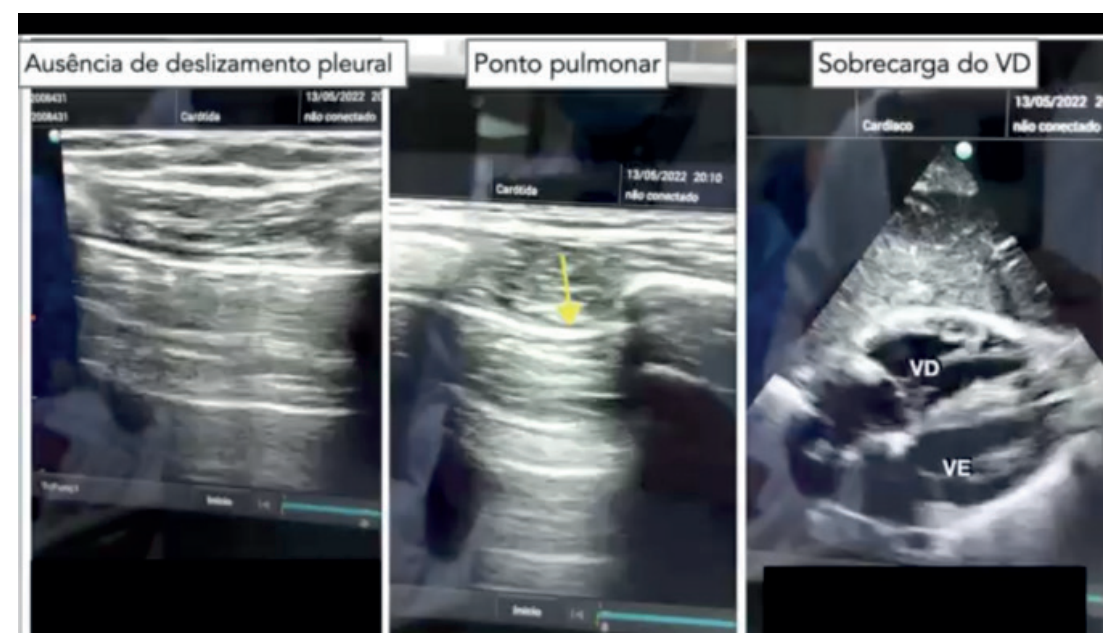

Video 55 – *Obstructive tension pneumothorax. Patient admitted for drainage of spontaneous pneumothorax due to subpleural blebs develops dyspnea and hypotension. Cardio-pulmonary point-of-care ultrasound on the contralateral hemithorax shows the classic triad: absence of lung sliding, lung point, and right ventricular overload. Link: http://abccardiol.org/supplementary-material/2026/12304/PosicionamentoPOCUS/Video 55 choque obstrutivo pneumotorax hipertensivo.mp4*

Supplement 2

Compiled Videos

SECTION 7 – Short-term circulatory support devices

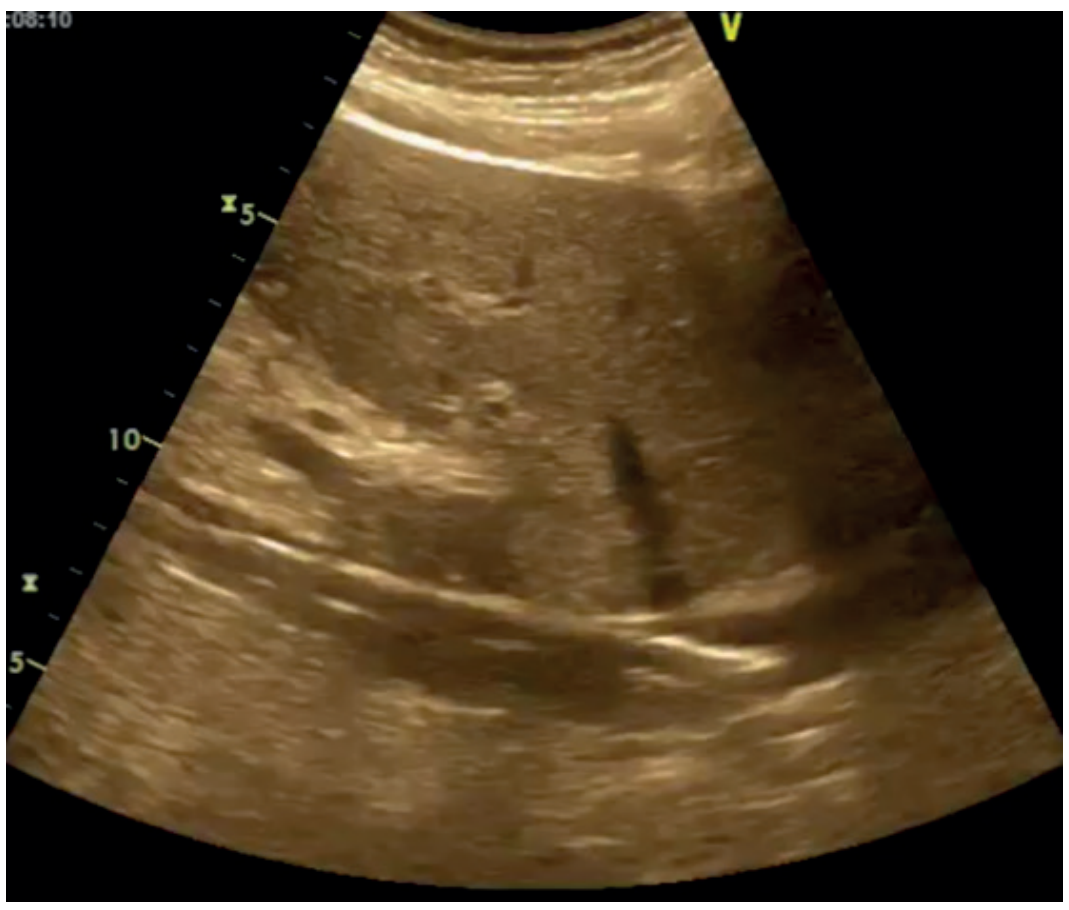

Video 56 – *Extracorporeal membrane oxygenation (ECMO) cannula inferior vena cava. ECMO cannula in the inferior vena cava near the confluence of the hepatic veins. Link: http://abccardiol.org/supplementary-material/2026/12304/PosicionamentoPOCUS/Video 56 Cânula ECMO VCI.mp4*

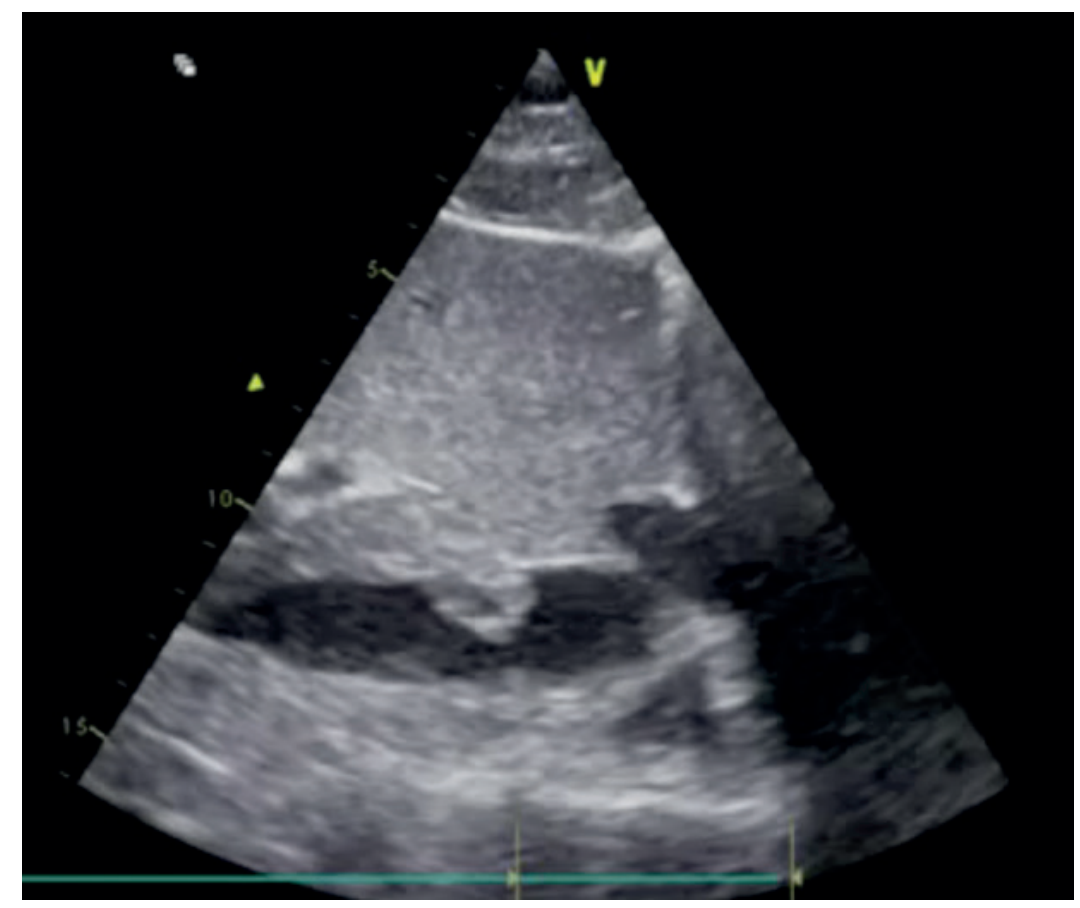

Video 57 – *Inferior vena cava thrombus after extracorporeal membrane oxygenation (ECMO) cannula removal. Thrombus attached to the inferior vena cava wall after ECMO cannula removal Link: http://abccardiol.org/supplementary-material/2026/12304/PosicionamentoPOCUS/Video 57 Trombo VCI pos retirada cânula ECMO.mp4*

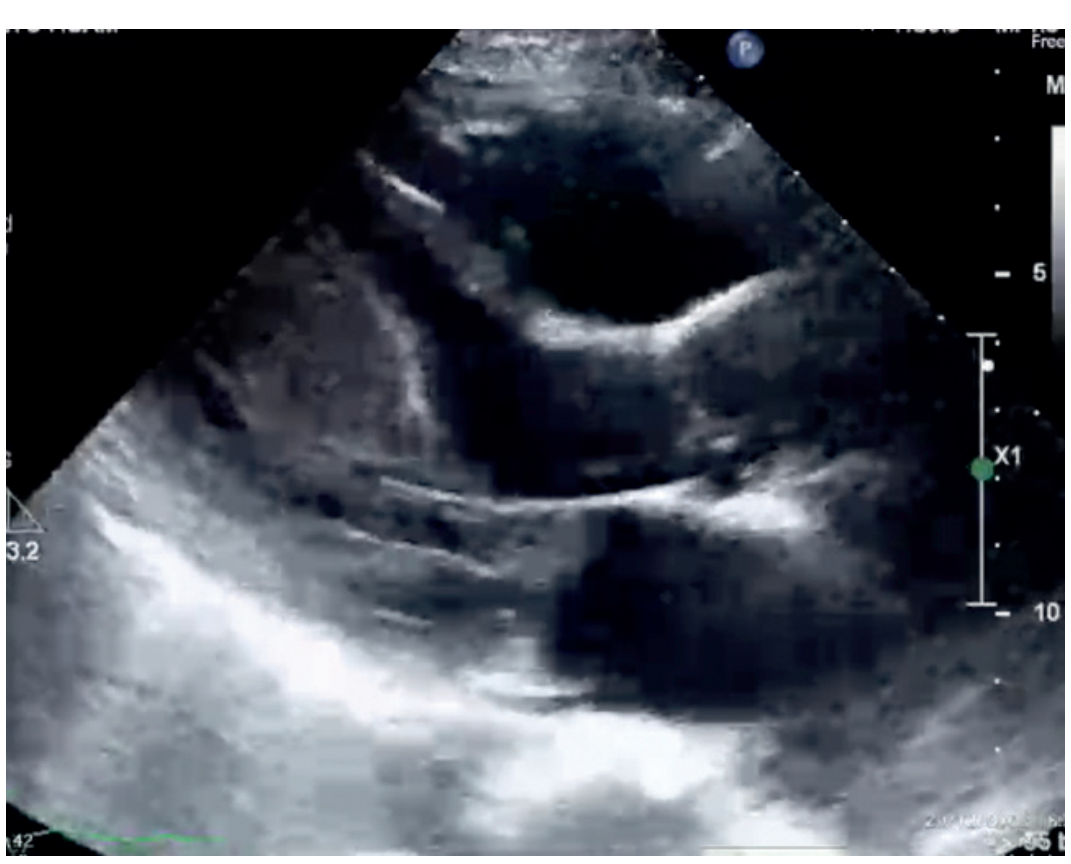

Video 58 – *Venoarterial extracorporeal membrane oxygenation (VA-ECMO) intraventricular thrombosis – parasternal long-axis view. Intraventricular thrombus in a patient on VA-ECMO. Link: http://abccardiol.org/supplementary-material/2026/12304/PosicionamentoPOCUS/Video 58 ECMO VA trombose intraventricular PLAX.mp4*

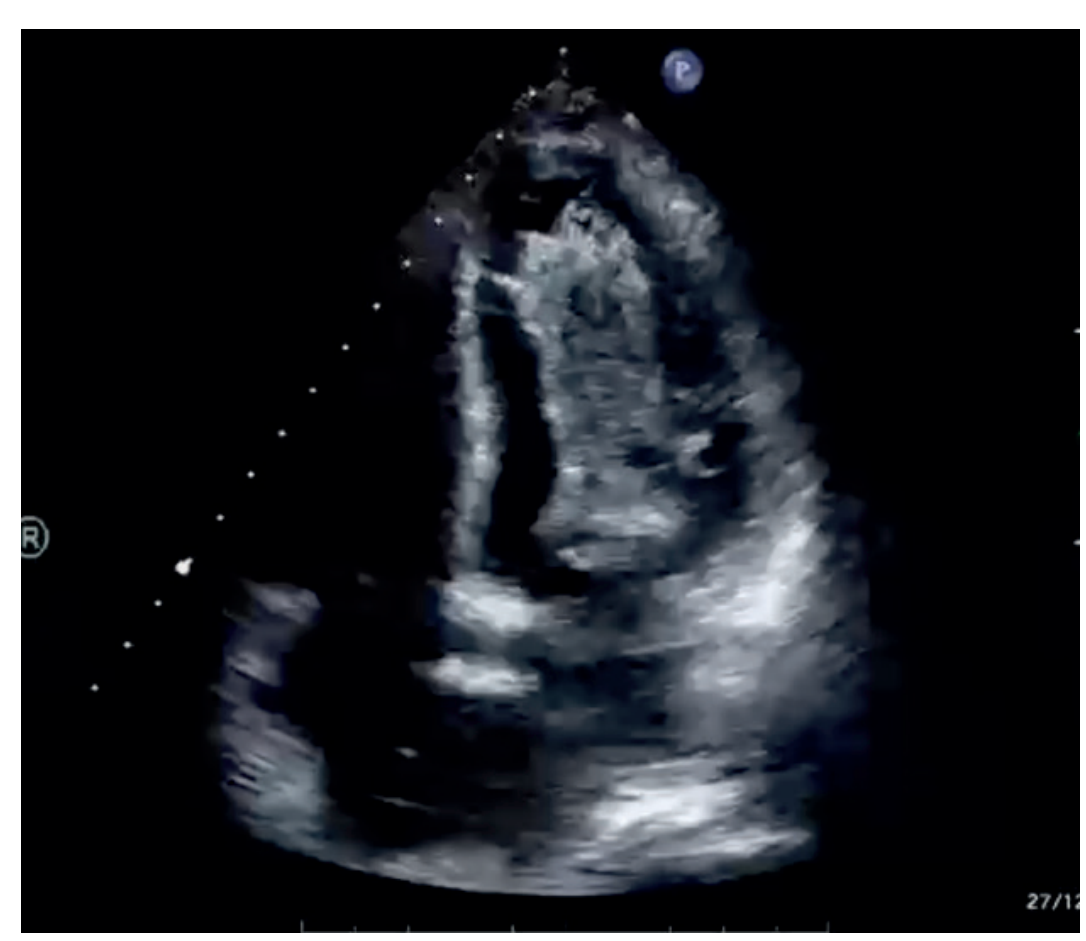

Video 59 – *Venoarterial extracorporeal membrane oxygenation (VA-ECMO) intraventricular thrombosis – apical 4-chamber view. Intraventricular thrombus in a patient on VA-ECMO. Link: http://abccardiol.org/supplementary-material/2026/12304/PosicionamentoPOCUS/Video 59 ECMO VA trombose intraventricular 4c.mp4*

Supplement 2t

Compiled Videos

SECTION 8 – POCUS in the emergency department

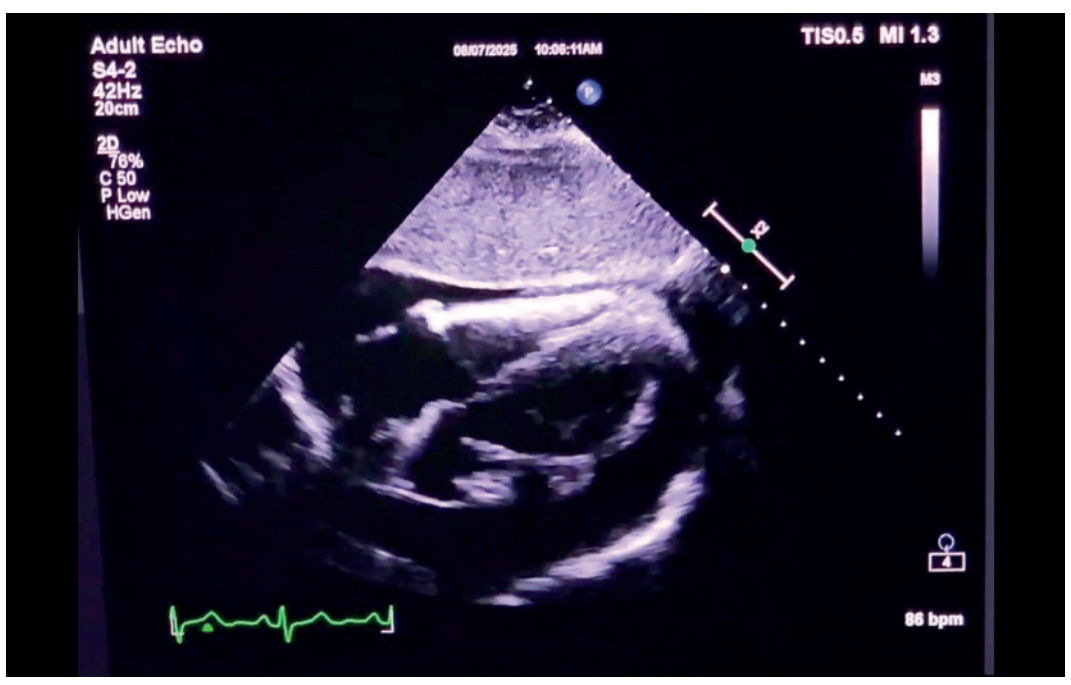

Video 60 – *Tamponade right ventricle and atrium collapse. Subcostal 4-chamber view – large pericardial effusion with right ventricular diastolic collapse. Link: http://abccardiol.org/supplementary-material/2026/12304/PosicionamentoPOCUS/Video 60 Tamponamento colabamento VD e AD.mp4*

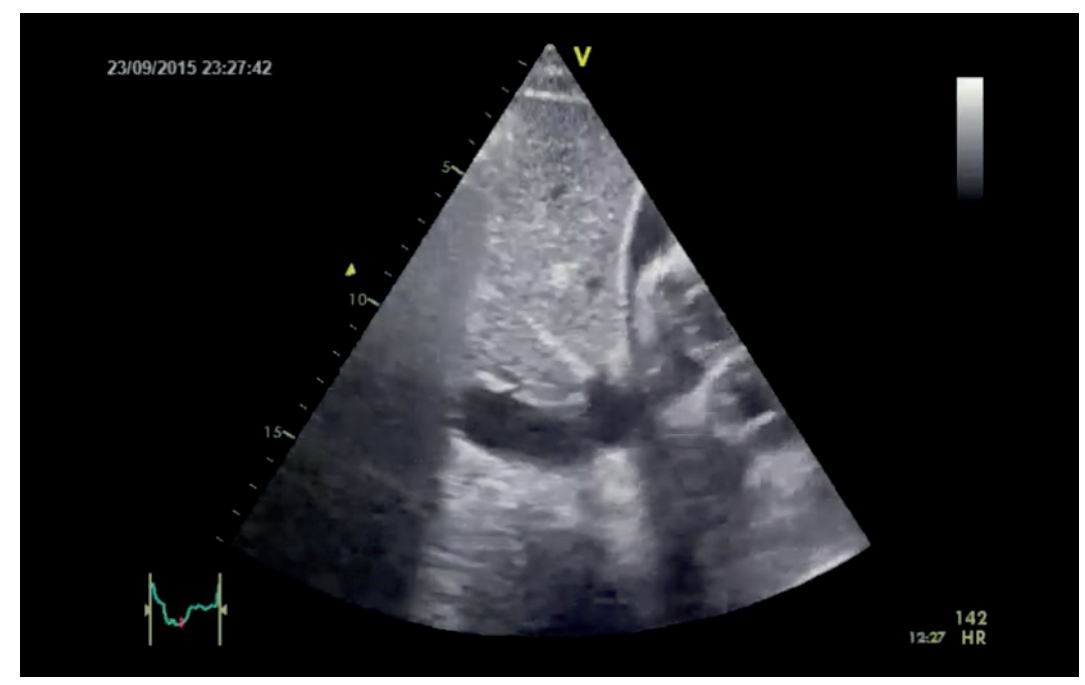

Video 61 – *Cardiac tamponade inferior vena cava dilation. Subcostal view of the inferior vena cava – large pericardial effusion causing inferior vena cava distension. Link: http://abccardiol.org/supplementary-material/2026/12304/PosicionamentoPOCUS/Video 61 Tamponamento cardiaco dilatação de VCI.mp4*

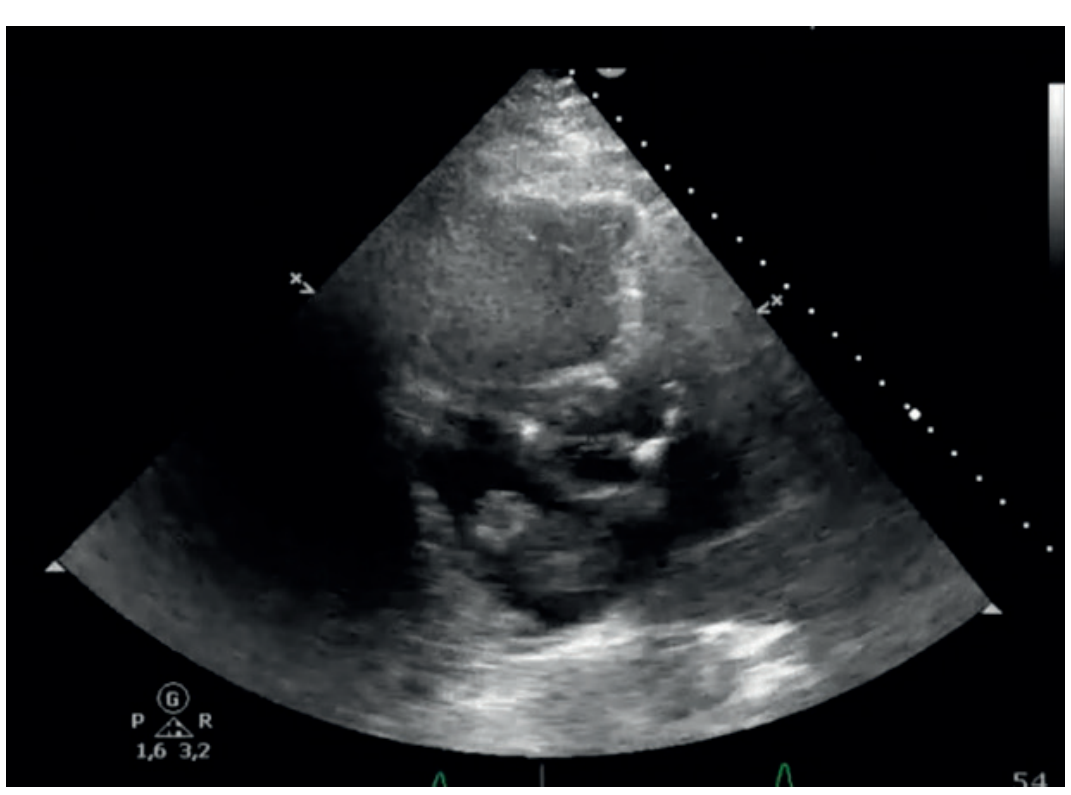

Video 62 – *Thrombus in transit – apical 4-chamber view. Apical view showing thrombus in the right-sided chambers in a patient with submassive pulmonary embolism. Link: http://abccardiol.org/supplementary-material/2026/12304/PosicionamentoPOCUS/Video 62 Trombo em transito A4C.mp4*

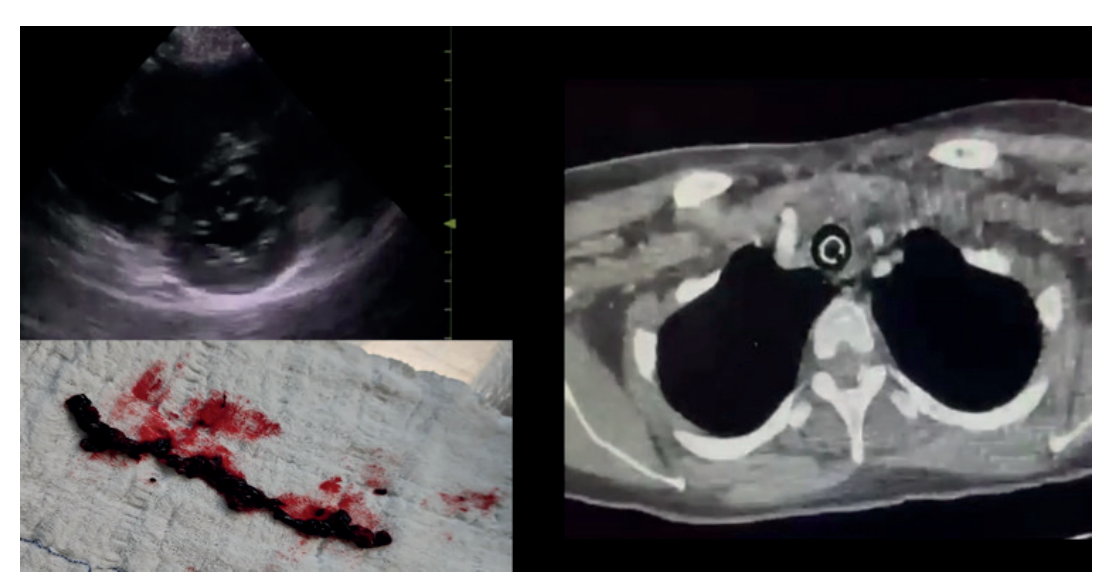

Video 63 – *Massive pulmonary embolism progressing to cardiac arrest reversed. Patient admitted in shock. Echocardiography shows acute right ventricular overload. Progresses to cardiac arrest with return of spontaneous circulation; computed tomography angiography confirms massive pulmonary embolism. Patient underwent mechanical thrombectomy. Link: http://abccardiol.org/supplementary-material/2026/12304/PosicionamentoPOCUS/Video 63 TEP maciço evoluindo com PCR revertida.mp4*

Supplement 2

Compiled Videos

SECTION 9 – POCUS in cardiac arrest

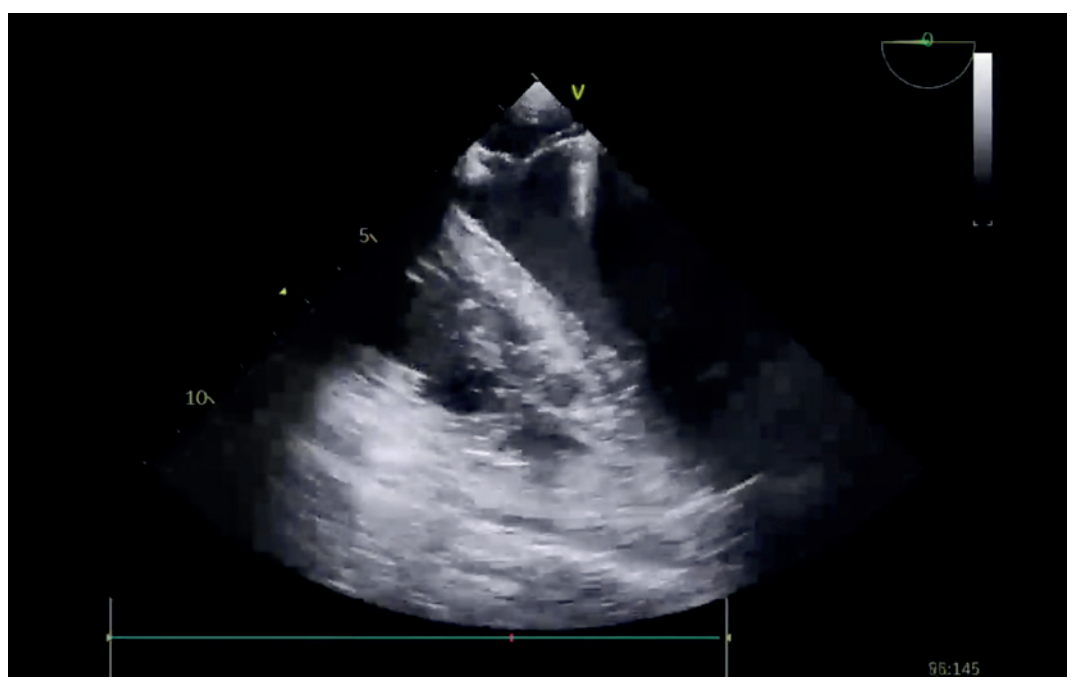

Video 64 – *Transesophageal echocardiography and chest compressions. Transesophageal mid-esophageal – 4-chamber view. Patient with biological mitral prosthesis undergoing cardiopulmonary resuscitation. Link: http://abccardiol.org/supplementary-material/2026/12304/PosicionamentoPOCUS/Video 64 Eco transesofágico e massagem cardiaca.mp4*

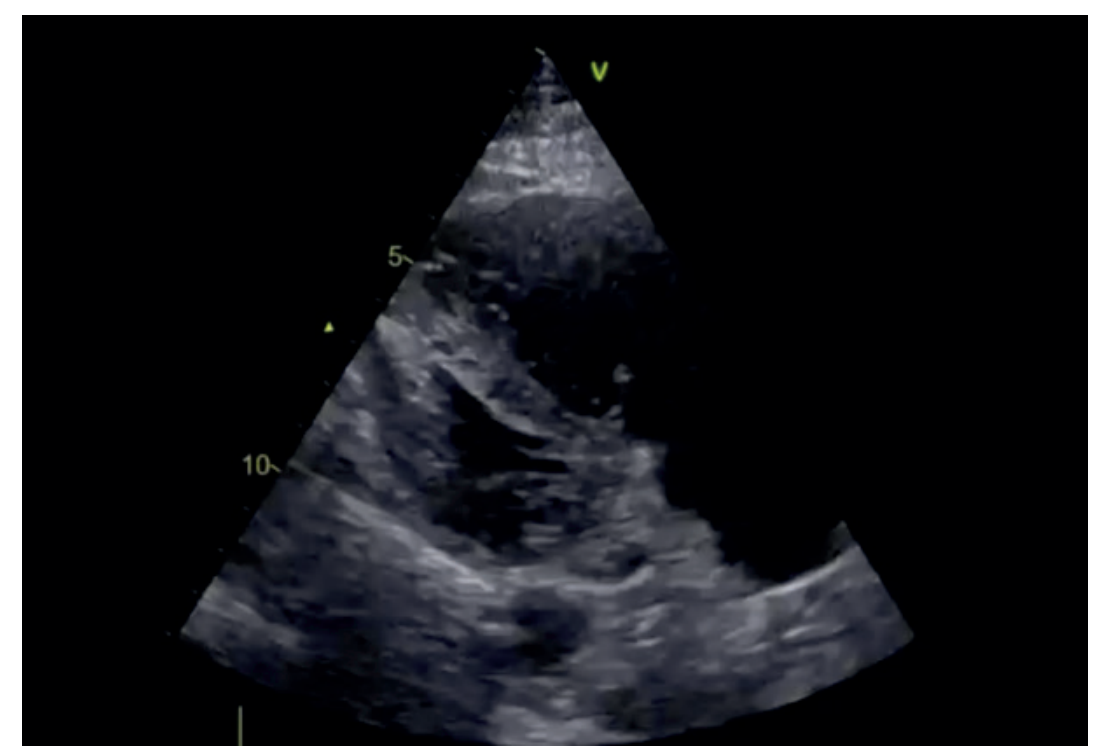

Video 65 – *Massive pulmonary embolism progressing to cardiac arrest 2 – parasternal long-axis view. Massive pulmonary embolism causing acute right ventricular overload in a patient evolving to cardiac arrest. Link: http://abccardiol.org/supplementary-material/2026/12304/PosicionamentoPOCUS/Video 65 TEP macico evoluindo com PCR2.mp4*

Supplement 2

Compiled Videos

SECTION 10 – Ultrasound-guided procedures

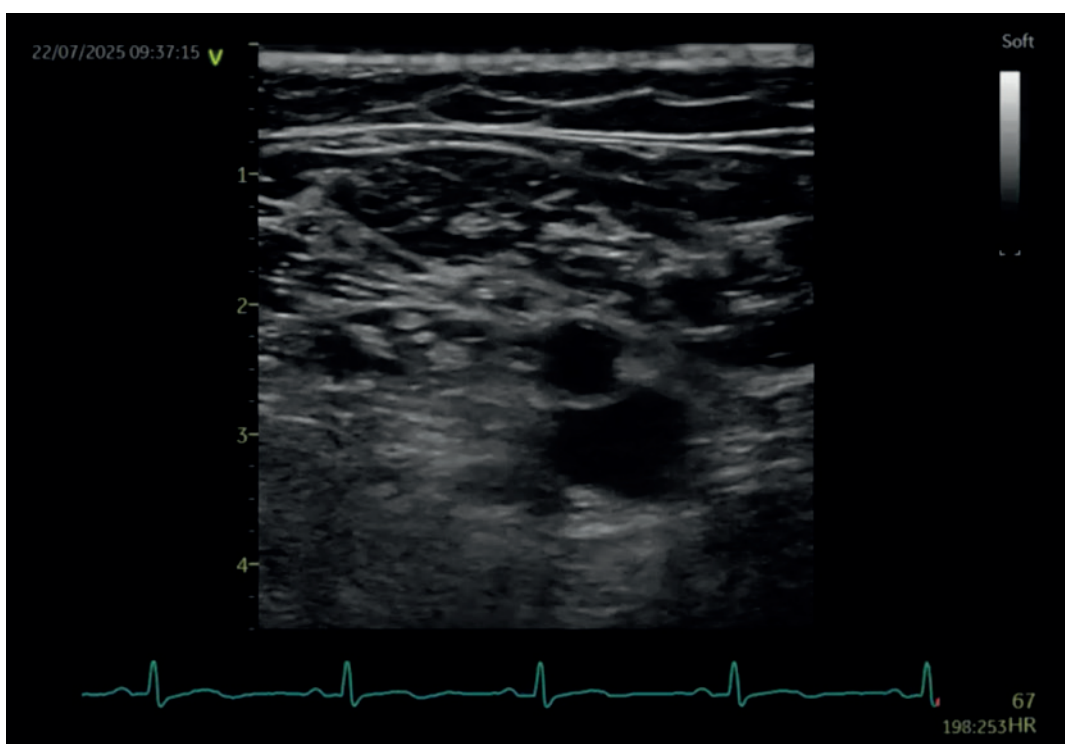

Video 66 – *Ultrasound-guided venous puncture – short axis view. Ultrasound-guided femoral vein access using the transverse technique. Link: http://abccardiol.org/supplementary-material/2026/12304/PosicionamentoPOCUS/Video 66 Punçao venosa ecoguida eixo curto.mp4*

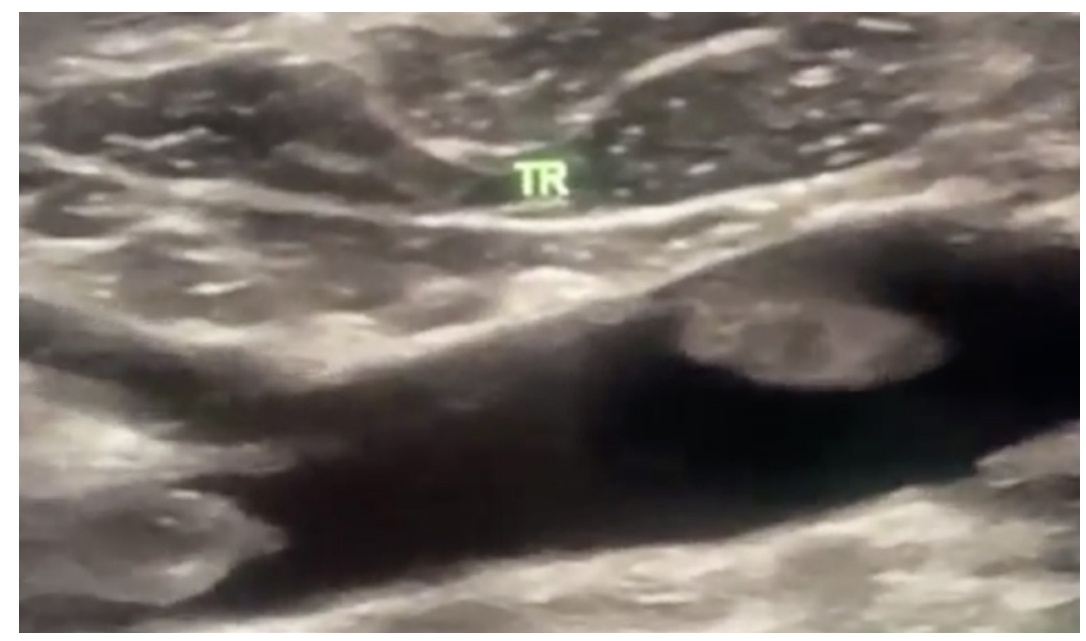

Video 67 – *Mobile catheter-related thrombus. Subclavian vein thrombosis with a highly mobile thrombotic component related to a previous catheter in the region. Link: http://abccardiol.org/supplementary-material/2026/12304/PosicionamentoPOCUS/Video 67 Trombo móvel relacionado a cateter.mp4*

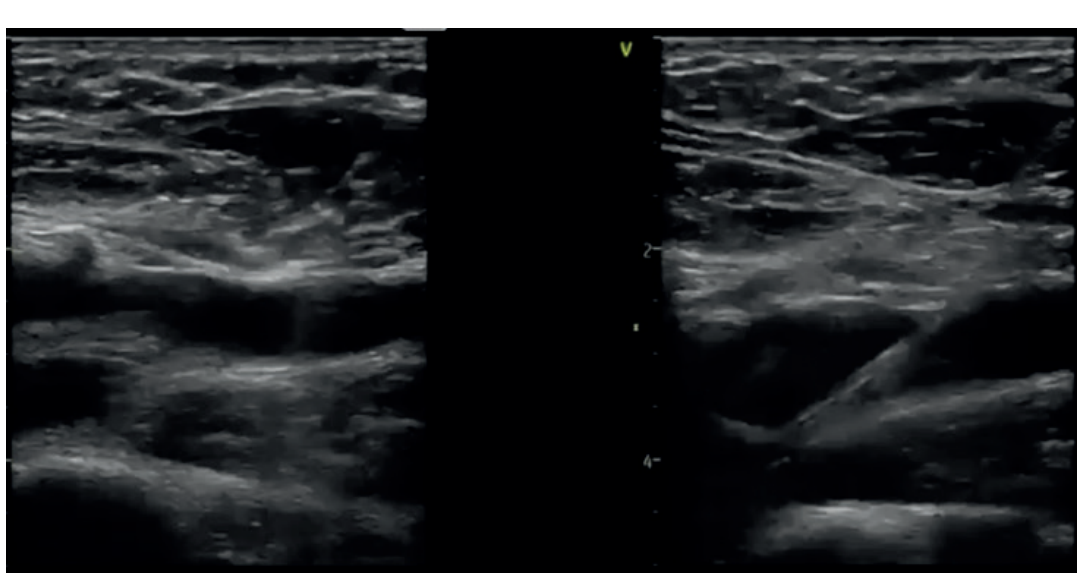

Video 68 – *Ultrasound-guided longitudinal puncture. Visualization of the needle trajectory along its entire length, including the exact point of vessel entry. The second clip shows guidewire advancement. Link: http://abccardiol.org/supplementary-material/2026/12304/PosicionamentoPOCUS/Video 68 Punção guiada longitudinal.mp4*
