# Supplementary material for: Posicionamento sobre o Uso do Ultrassom "Point of Care" (POCUS) na Medicina Cardiovascular – 2026
Source: Arq Bras Cardiol. 2026 May 4;123(4):e20260222. [Article in Portuguese] doi: 10.36660/abc.20260222 (PMC13671151; doi:10.36660/abc.20260222)
Supplement: Supplementary file 4 [file 0066-782x-abc-123-4-e20260222-Suppl02.pdf]

Suplemento 2

Posicionamento POCUS – VIDEOS Reunidos

SESSÃO 2 – POCUS cardíaco

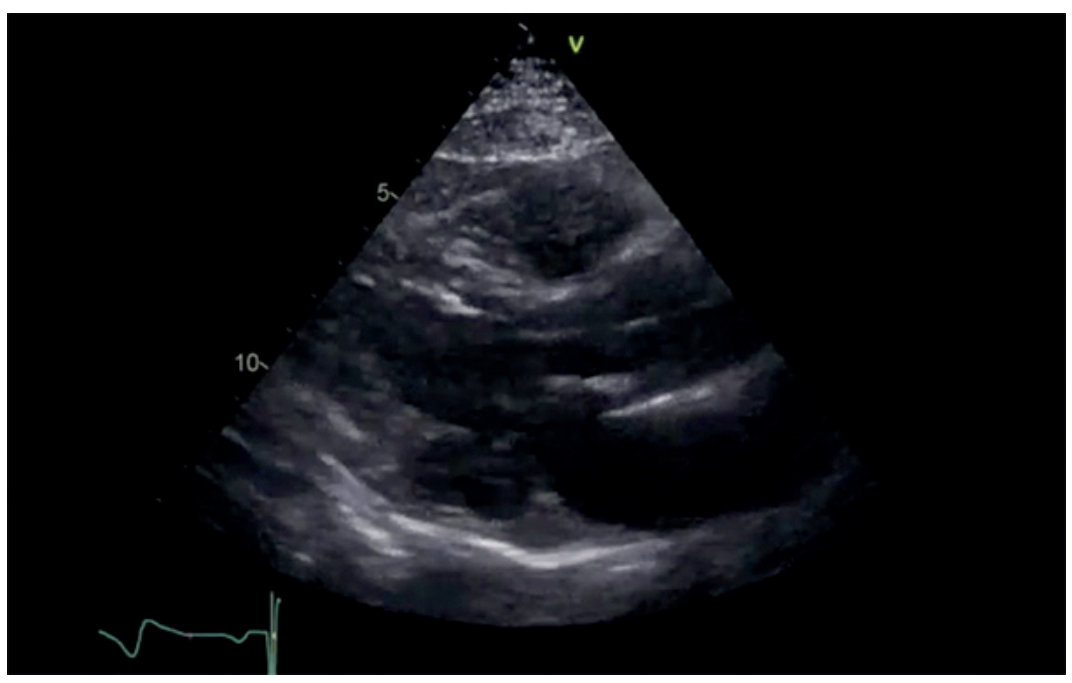

Vídeo 01 – *Corte paraesternal longitudinal – Função sistólica global do VE normal. Em: http://abccardiol.org/supplementary-material/2026/12304/PosicionamentoPOCUS/Video 01 PLAX normal.mp4*

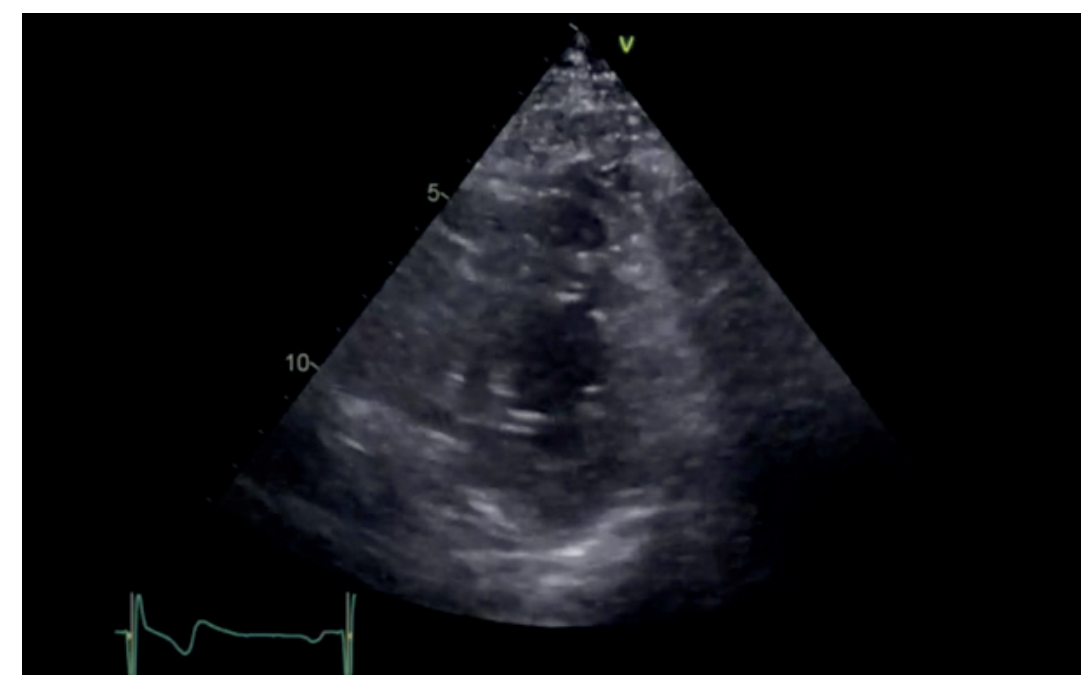

Vídeo 02 – *Corte paraesternal transversal (eixo curto) – Função sistólica global do VE normal. Em: http://abccardiol.org/supplementary-material/2026/12304/PosicionamentoPOCUS/Video 02 PSAX normal.mp4*

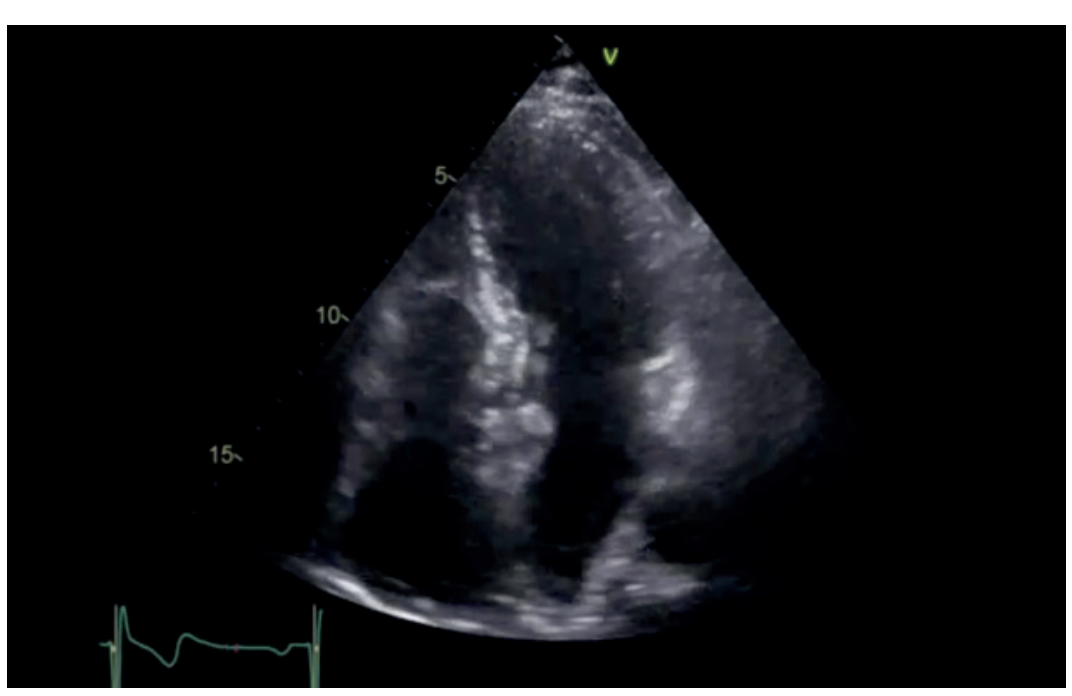

Vídeo 03 – *Corte apical 4 câmaras – Função sistólica global do VE normal. Em: http://abccardiol.org/supplementary-material/2026/12304/PosicionamentoPOCUS/Video 03 A4C normal.mp4*

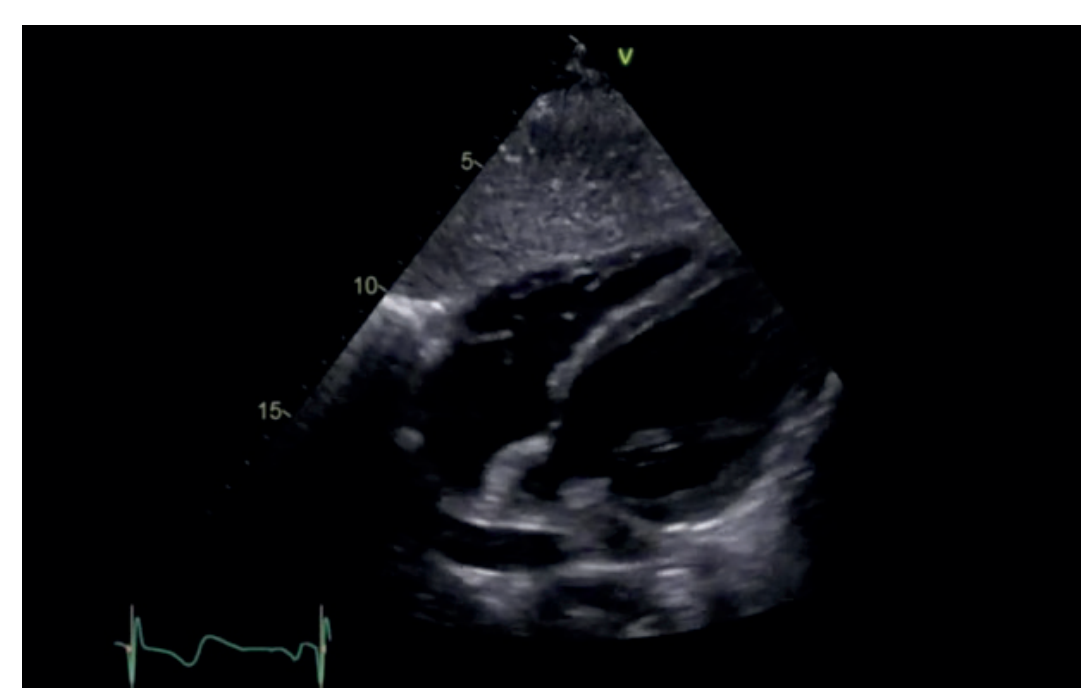

Vídeo 04 – *Corte subcostal 4 câmaras – Função sistólica global do VE normal. Em: http://abccardiol.org/supplementary-material/2026/12304/PosicionamentoPOCUS/Video 04 Subcostal Normal.mp4*

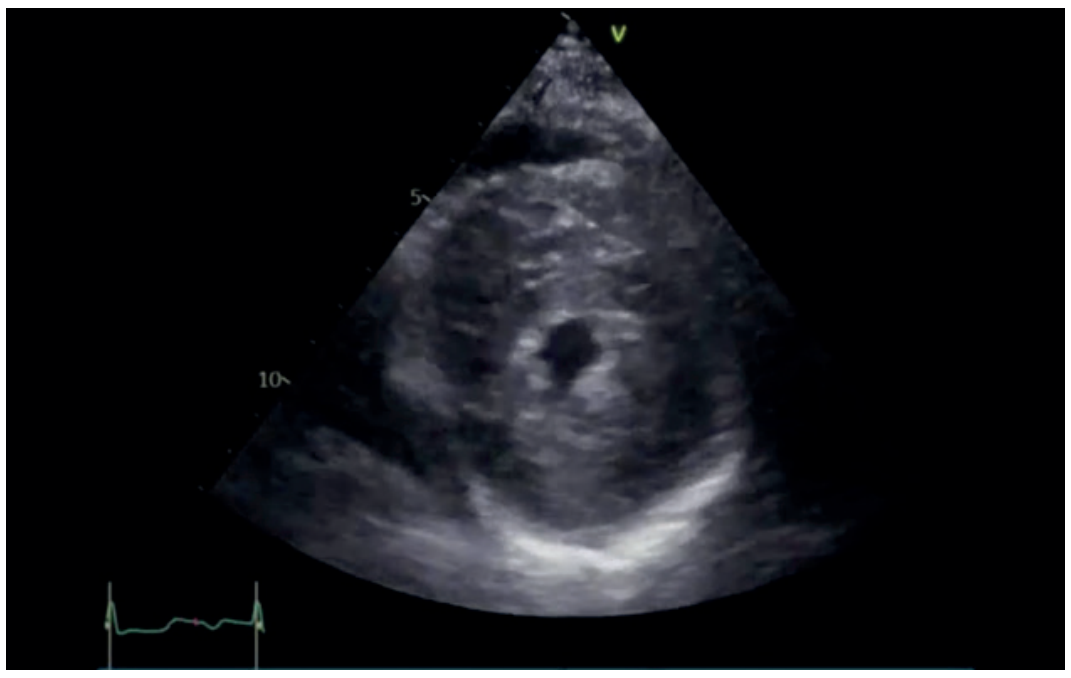

Vídeo 05 – *Corte paraesternal transversal – Função sistólica global do VE hiperdinâmica. Em: http://abccardiol.org/supplementary-material/2026/12304/PosicionamentoPOCUS/Video 05 PSAX hiperdinamico.mp4*

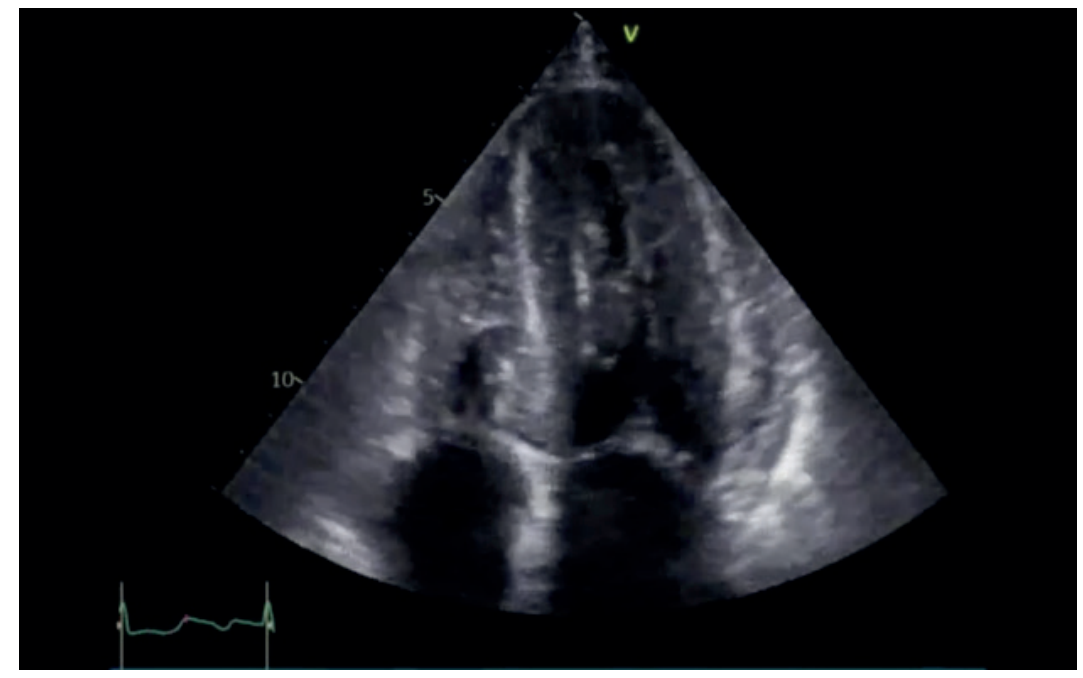

Vídeo 06 – *Corte apical 4 câmaras – Função sistólica global do VE hiperdinâmica. Em: http://abccardiol.org/supplementary-material/2026/12304/PosicionamentoPOCUS/Video 06 A4C hiperdinamico.mp4*

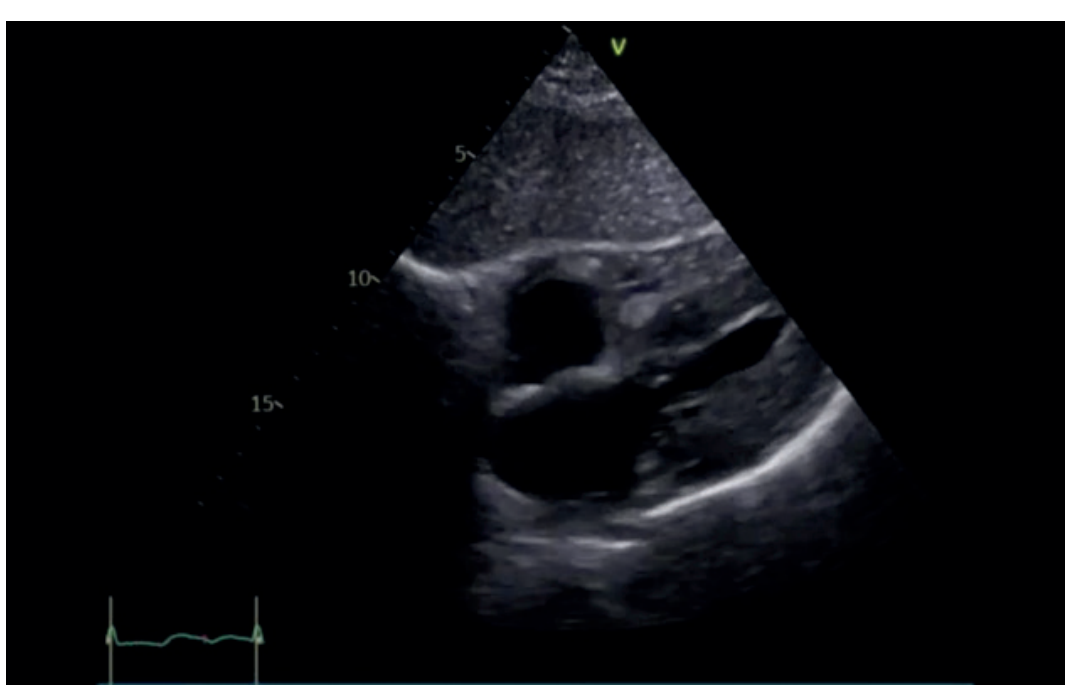

Vídeo 07 – *Corte subcostal 4 câmaras – Função sistólica global do VE hiperdinâmica. Em: http://abccardiol.org/supplementary-material/2026/12304/PosicionamentoPOCUS/Video 07 Subcostal hiperdinamico.mp4*

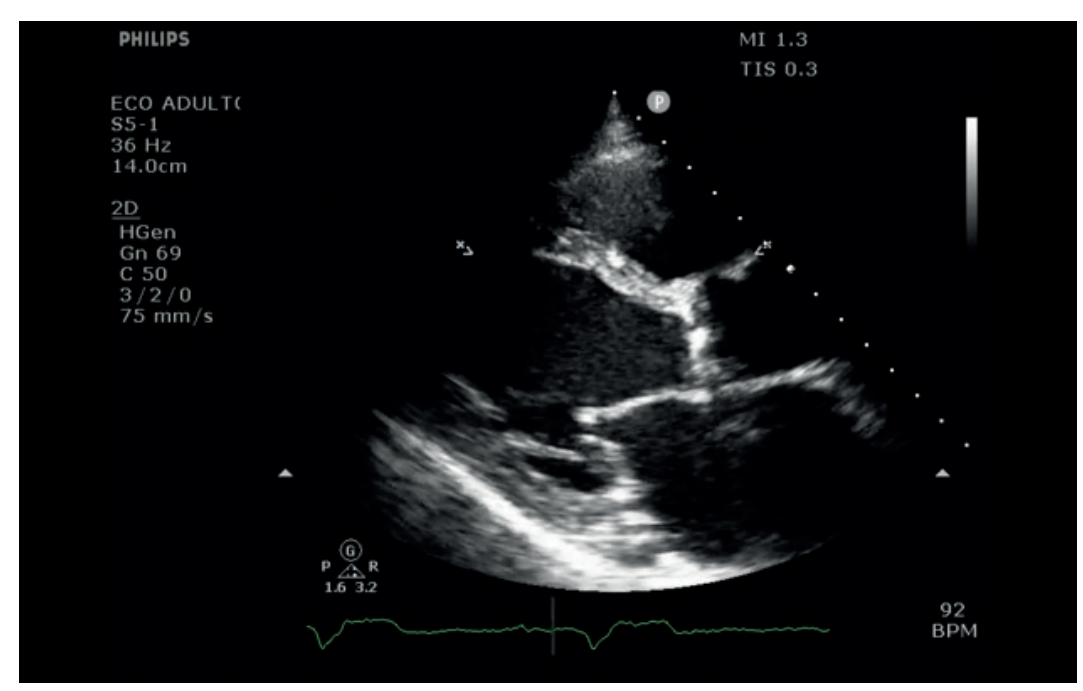

Vídeo 08 – *Corte paraesternal longitudinal – Função sistólica global do VE reduzida. Em: http://abccardiol.org/supplementary-material/2026/12304/PosicionamentoPOCUS/Video 08 PLAX FS reduzida.mp4*

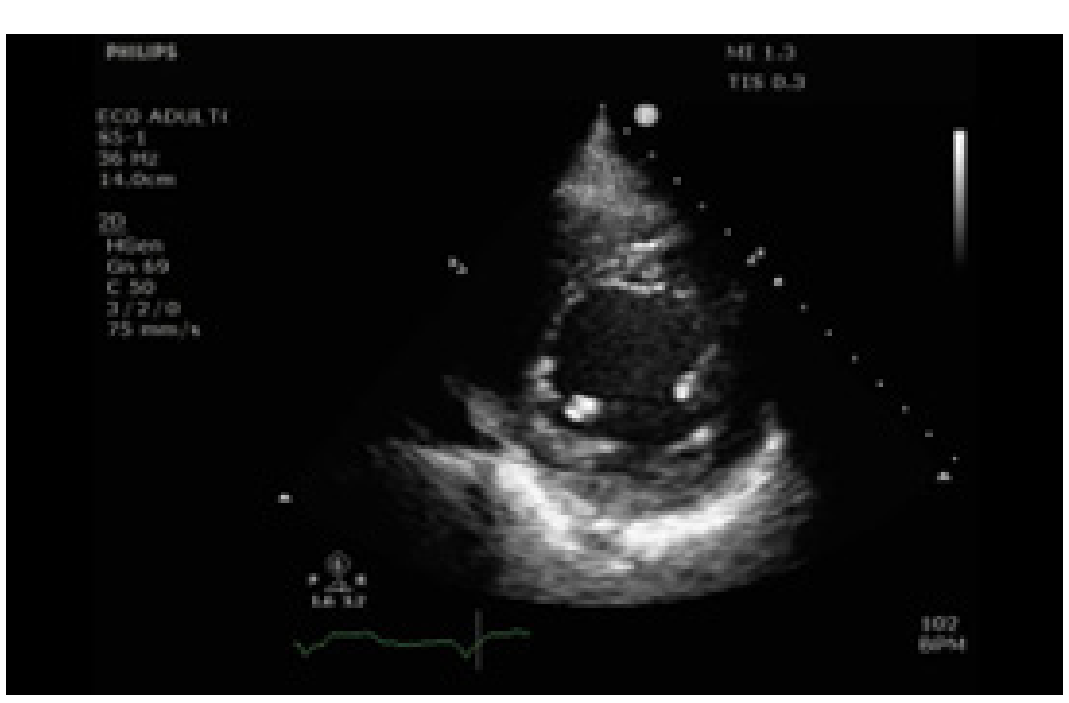

Vídeo 09 – *Corte paraesternal transversal (eixo curto) – Função sistólica global do VE reduzida. Em: http://abccardiol.org/supplementary-material/2026/12304/PosicionamentoPOCUS/Video 09 PSAX FS reduzida.mp4*

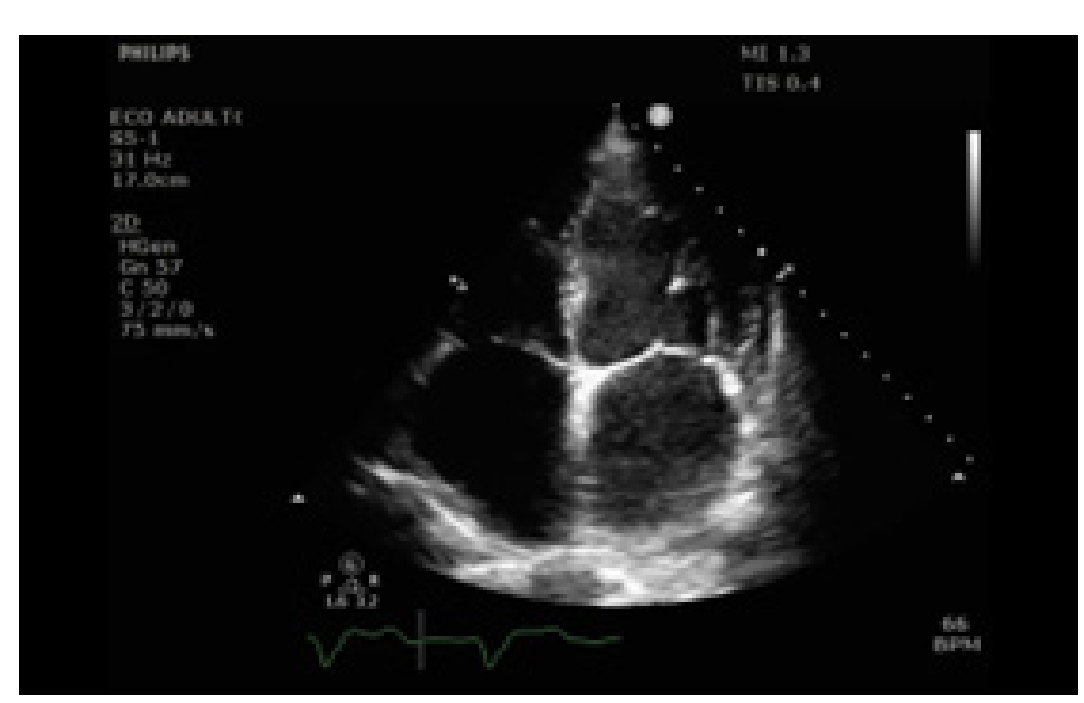

Vídeo 10 – *Corte apical 4 câmaras – Função sistólica global do VE reduzida. Em: http://abccardiol.org/supplementary-material/2026/12304/PosicionamentoPOCUS/Video 10 APICAL 4C FS reduzida.mp4*

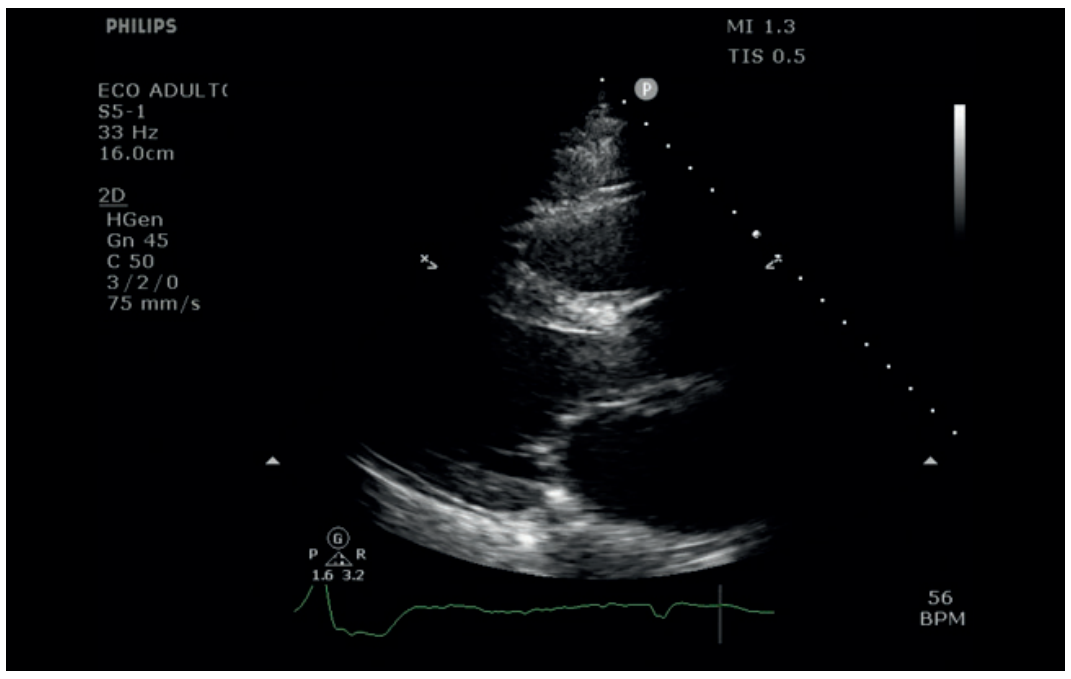

Vídeo 11 – *Corte paraesternal longitudinal – Disfunção sistólica global grave do VE. Em: http://abccardiol.org/supplementary-material/2026/12304/PosicionamentoPOCUS/Video 11 PLAX Disfuncao grave.mp4*

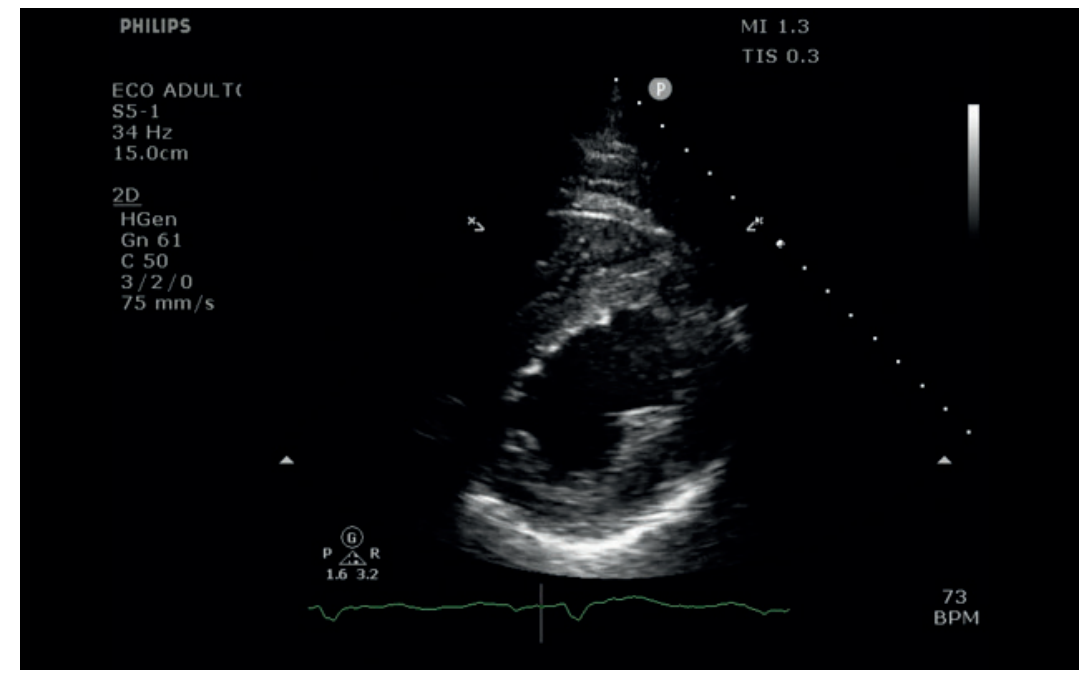

Vídeo 12 – *Corte paraesternal transversal – Disfunção sistólica global grave do VE. Em: http://abccardiol.org/supplementary-material/2026/12304/PosicionamentoPOCUS/Video 12 PSAX disfuncao grave.mp4*

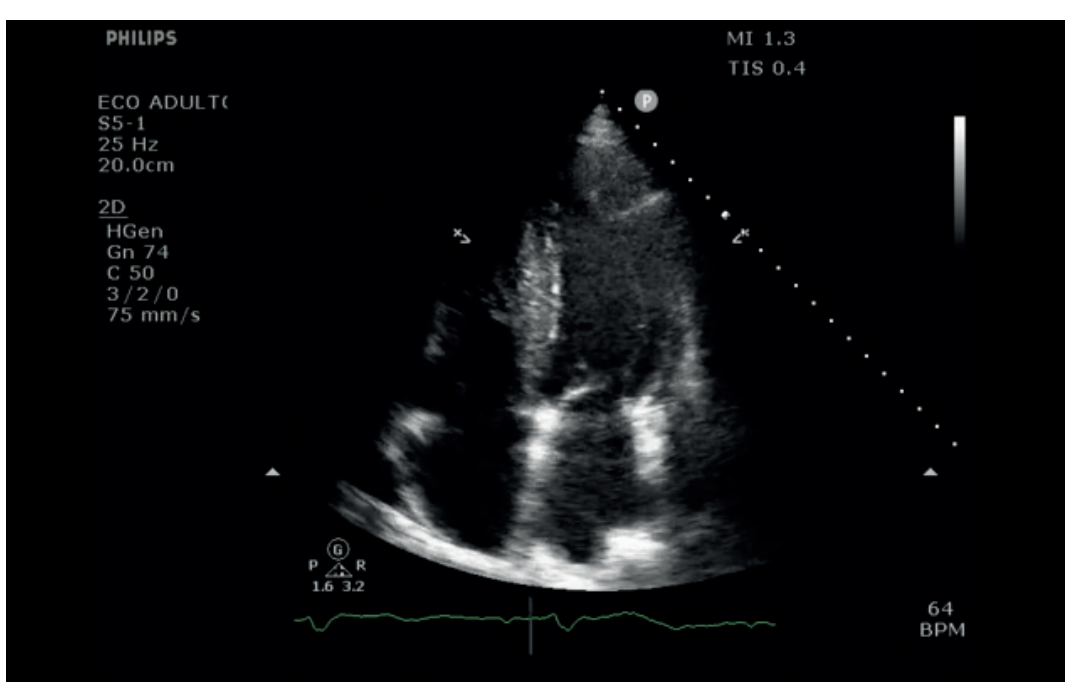

Vídeo 13 – *Corte paraesternal transversal – Disfunção sistólica global grave do VE. Em: http://abccardiol.org/supplementary-material/2026/12304/PosicionamentoPOCUS/Video 13 Apical 4C disfuncao grave.mp4*

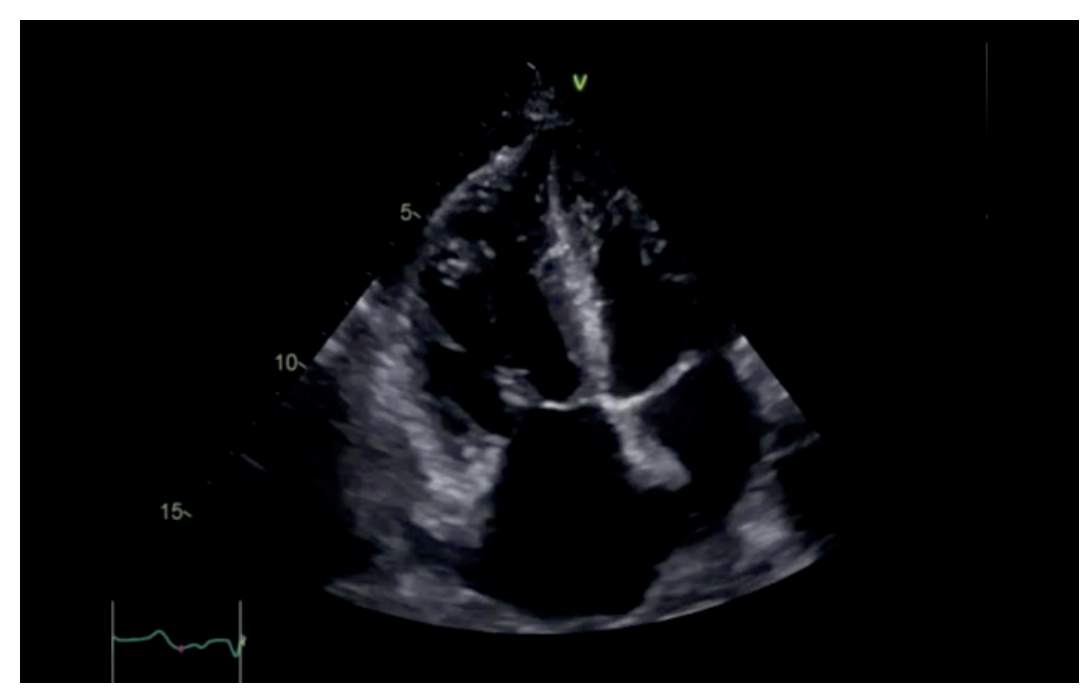

Vídeo 14 – *Corte apical 4 câmaras – Sinal de McConnell na embolia pulmonar. Em: http://abccardiol.org/supplementary-material/2026/12304/PosicionamentoPOCUS/Video 14 TEP McConnell.mp4*

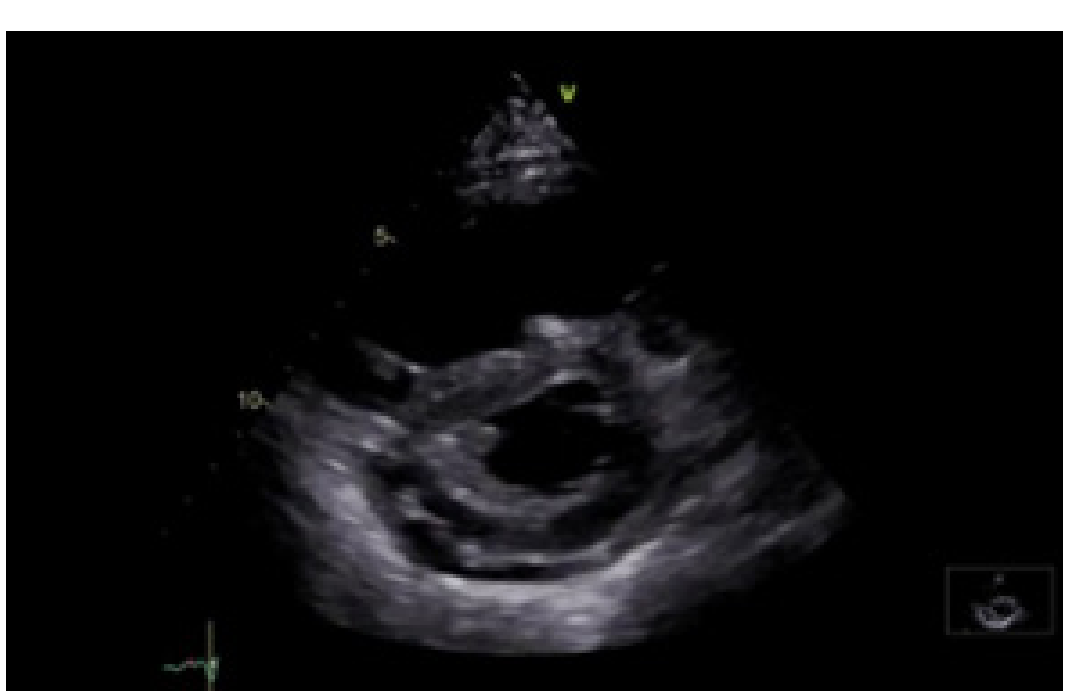

Vídeo 15 – *Corte paraesternal transversal – Aplainamento do septo interventricular (D shape) na embolia pulmonar. Em: http://abccardiol.org/supplementary-material/2026/12304/PosicionamentoPOCUS/Video 15 TEP- D shape.mp4*

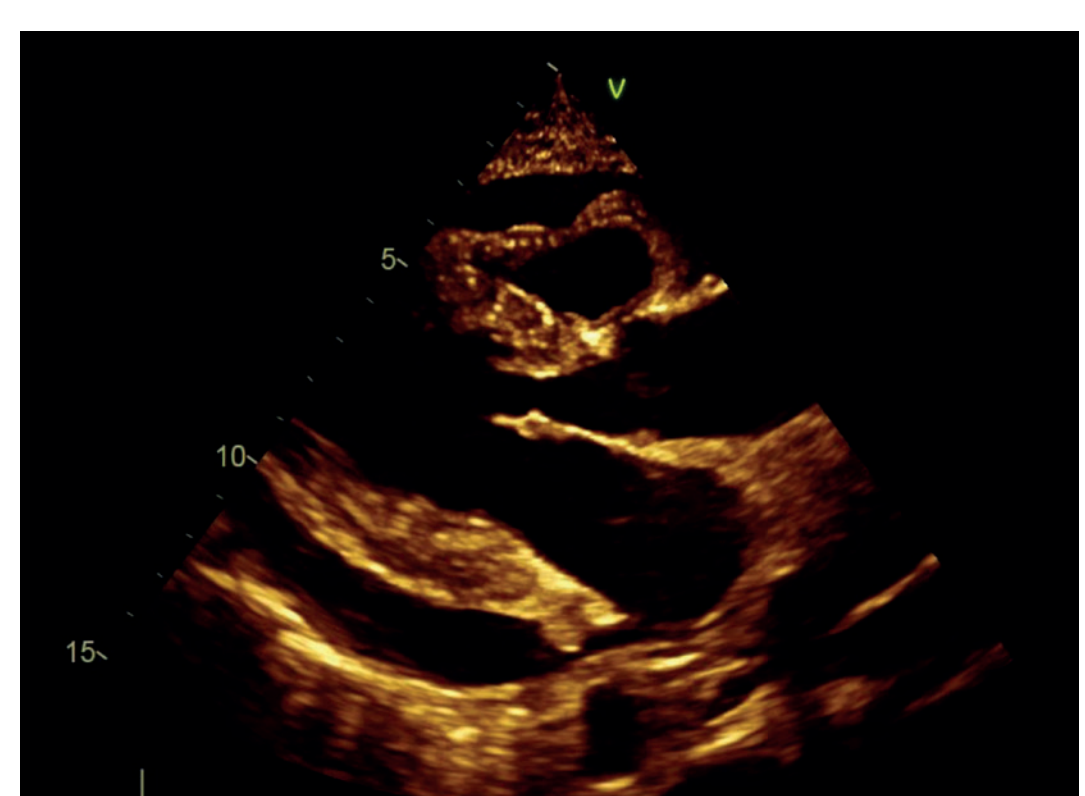

Vídeo 16 – *Corte paraesternal longitudinal – Derrame pericárdico grave com colabamento diastólico do ventrículo direito. Em: http://abccardiol.org/supplementary-material/2026/12304/PosicionamentoPOCUS/Video 16 Tamponamento PLAX.mp4*

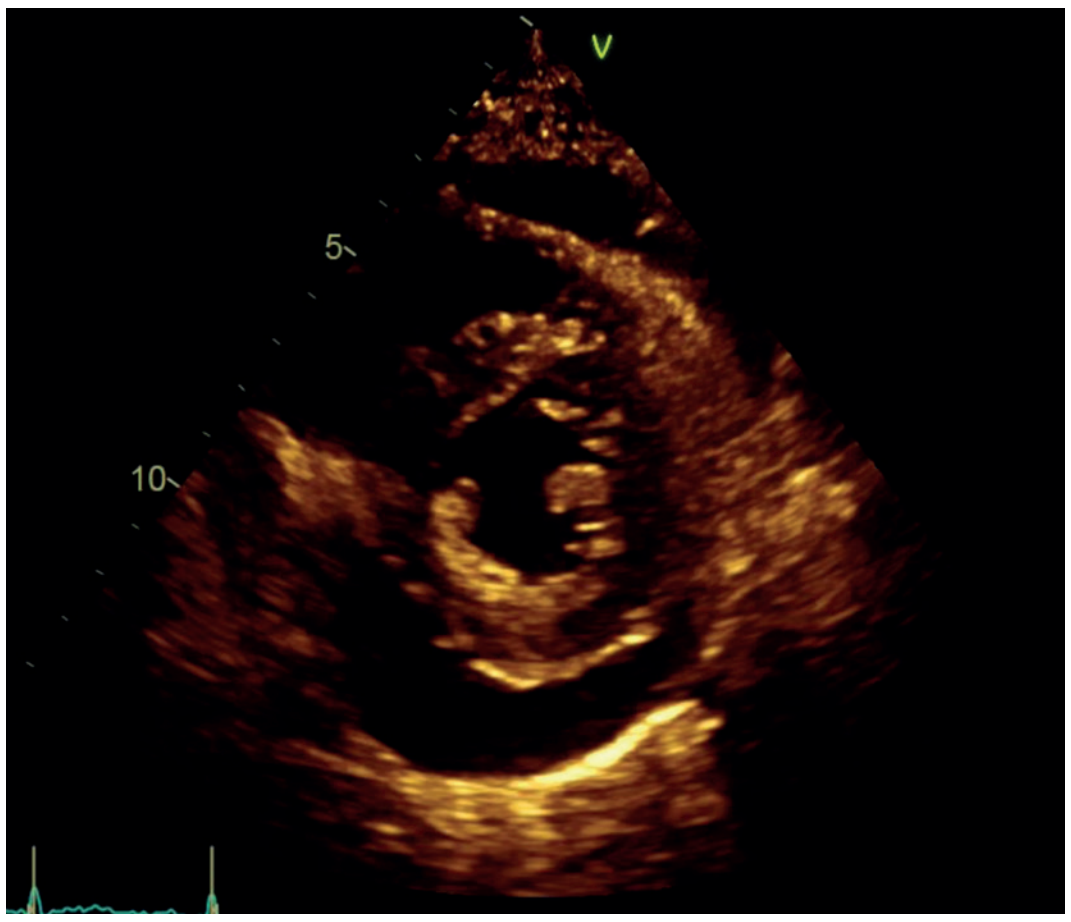

Vídeo 17 – *Corte paraesternal transversal – Derrame pericárdico grave com colabamento diastólico do ventrículo direito. Em: http://abccardiol.org/supplementary-material/2026/12304/PosicionamentoPOCUS/Video 17 Tamponamento PSAX.mp4*

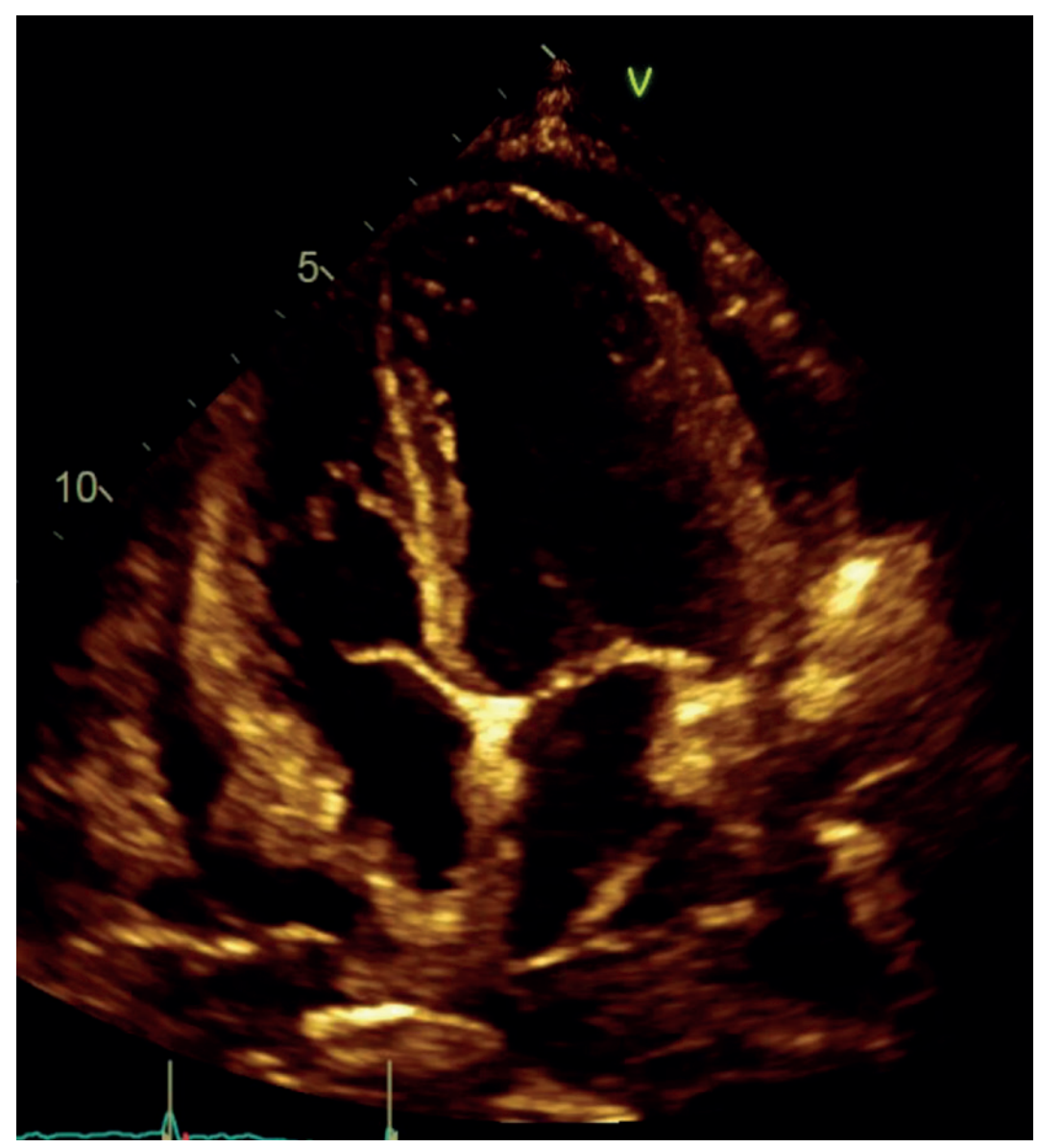

Vídeo 18 – *Corte apical 4 câmaras – Derrame pericárdico grave com restrição diastólica. Em: http://abccardiol.org/supplementary-material/2026/12304/PosicionamentoPOCUS/Video 18 Tamponamento A4C.mp4*

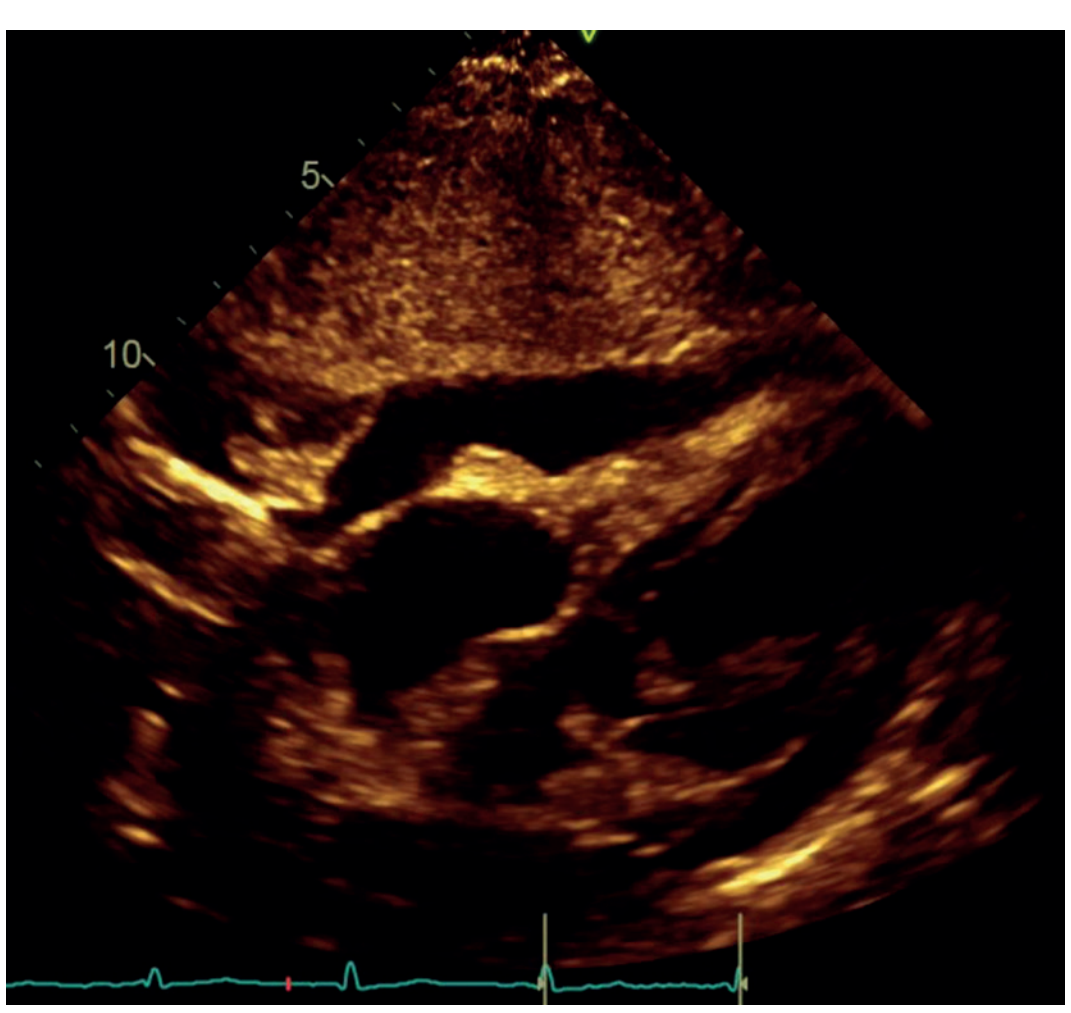

Vídeo 19 – *Corte subcostal – Derrame pericárdico grave com colabamento diastólico do ventrículo direito. Em: http://abccardiol.org/supplementary-material/2026/12304/PosicionamentoPOCUS/Video 19 Tamponamento subcostal.mp4*

Suplemento 2

Posicionamento POCUS – VIDEOS Reunidos

SESSÃO 3 – Ultrassom Pulmonar

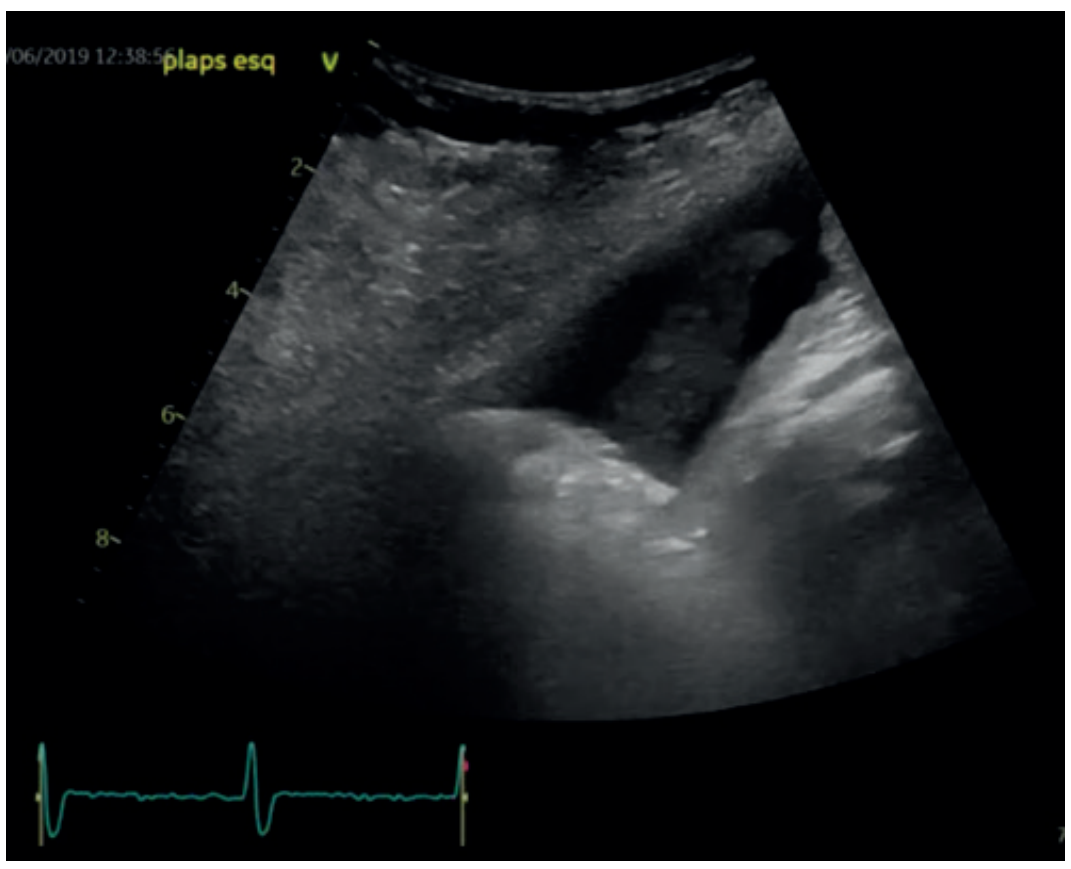

Vídeo 20 – *Atelectasia pulmonar caracterizada por lâmina de tecido pulmonar consolidado com movimentos sinusoides (sinal da medusa) em meio ao líquido pleural. Em: http://abccardiol.org/supplementary-material/2026/12304/PosicionamentoPOCUS/Video 20 US pulm atelectasia.mp4*

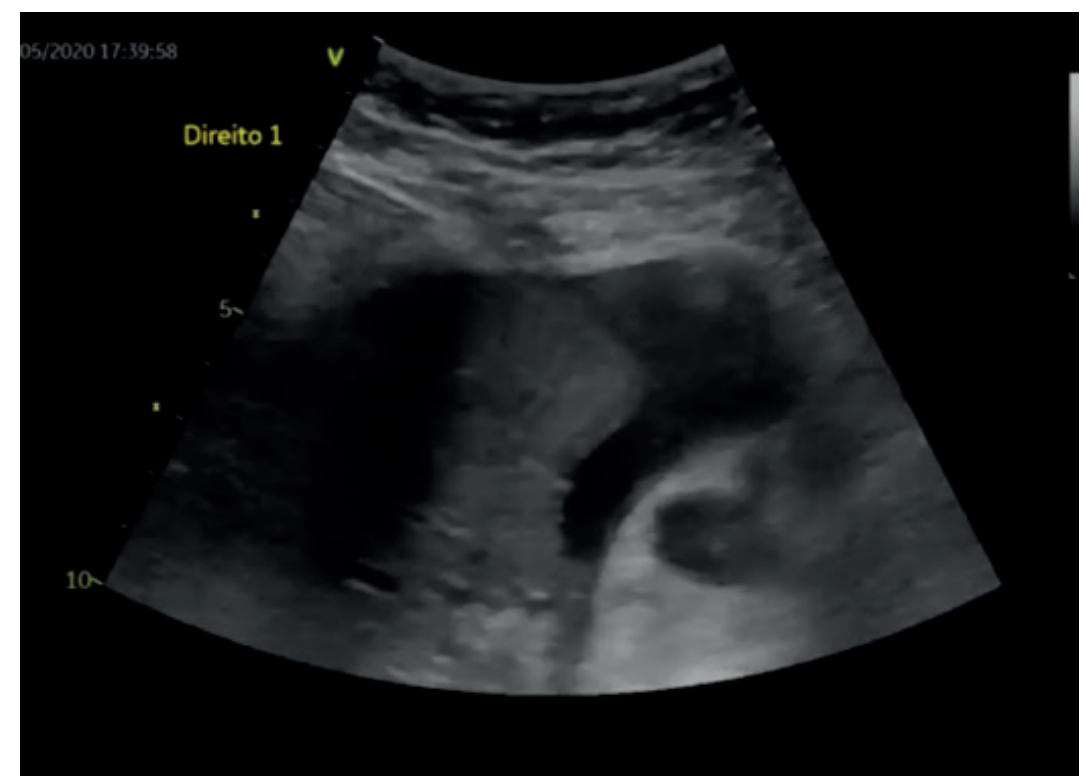

Vídeo 21 – *Consolidação translobar perda da aeração do tecido pulmonar que é preenchido por fluido infeccioso conferindo-lhe aspecto de estrutura sólida (hepatização) estendendo-se por todo campo ultrassônico. Em: http://abccardiol.org/supplementary-material/2026/12304/PosicionamentoPOCUS/Video 21 US pulm consolidação translobar.mp4*

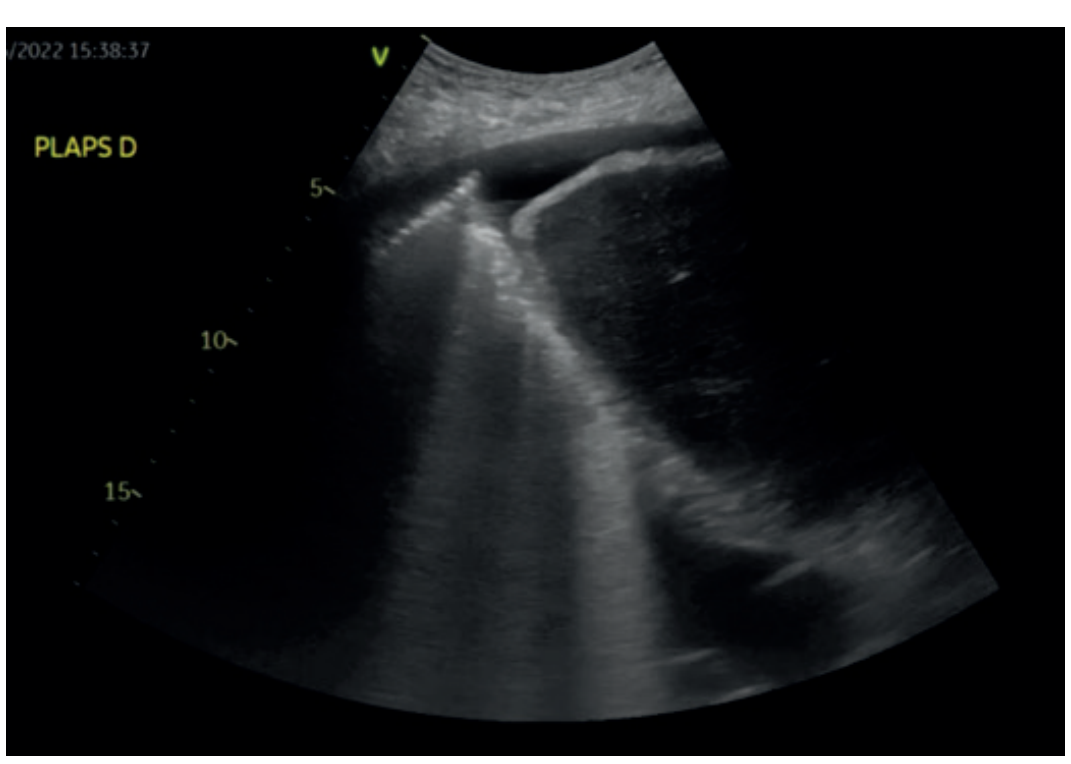

Vídeo 22 – *Derrame pleural leve associado a espessamento pleural e aderência pleural junto a cúpula diafragmática. Em: http://abccardiol.org/supplementary-material/2026/12304/PosicionamentoPOCUS/Video 22 US pulm derrame pl perdadeslizamento.mp4*

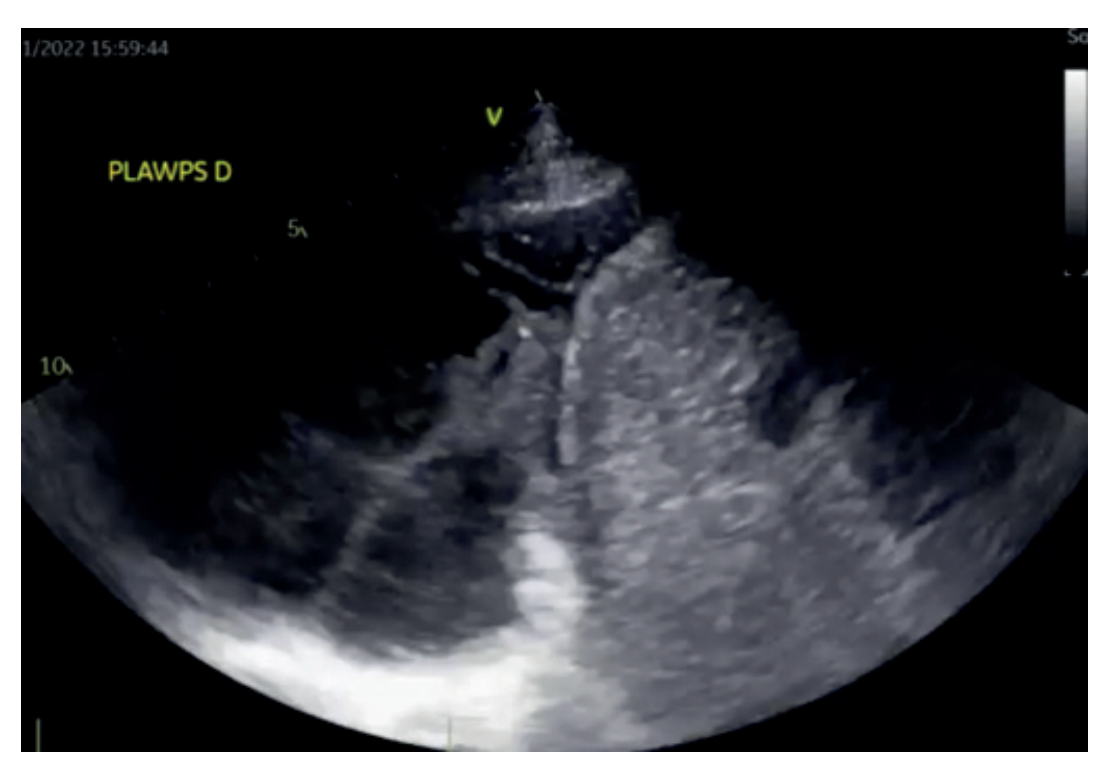

Vídeo 23 – *Derrame pleural complexo com a presença de debris e septações indica de origem infecciosa. Em: http://abccardiol.org/supplementary-material/2026/12304/PosicionamentoPOCUS/Video 23 US pulm derrame septado.mp4*

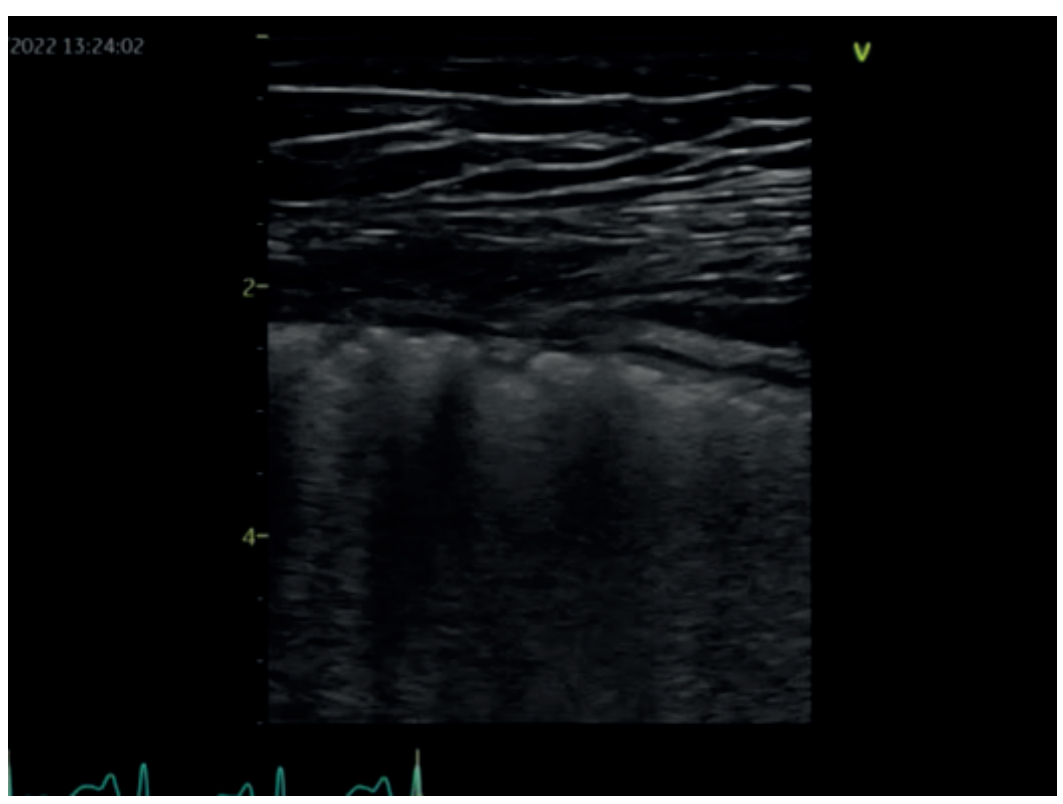

Vídeo 24 – *Espessamento e irregularidade da linha pleural com linhas B e cistos subpleurais em paciente com fibrose pulmonar por esclerodermia. Achado melhor evidenciado com a sonda linear. Em: http://abccardiol.org/supplementary-material/2026/12304/PosicionamentoPOCUS/Video 24 US pulm fibrose pulm esclerodermia.mp4*

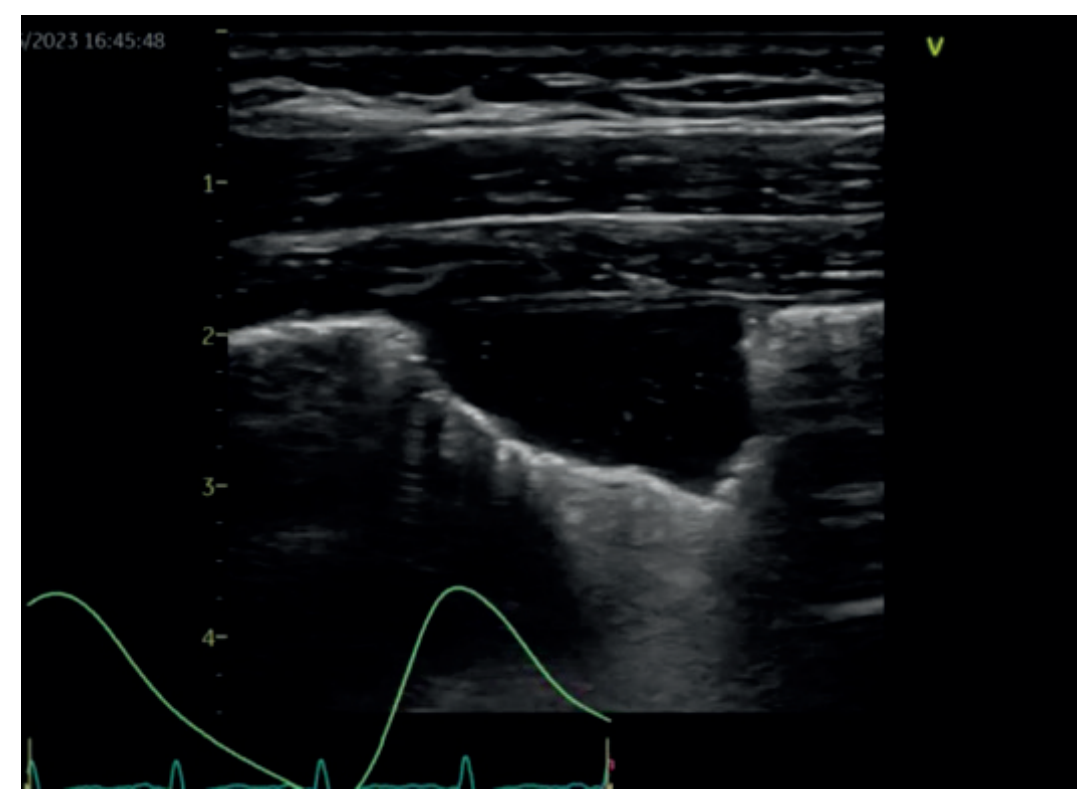

Vídeo 25 – *Infarto pulmonar caracterizado por pequena coleção hipoecoica subpleural e triangular em paciente com embolia pulmonar. Imagem obtida com sonda linear. Em: http://abccardiol.org/supplementary-material/2026/12304/PosicionamentoPOCUS/Video 25 US pulm infarto pulm.mp4*

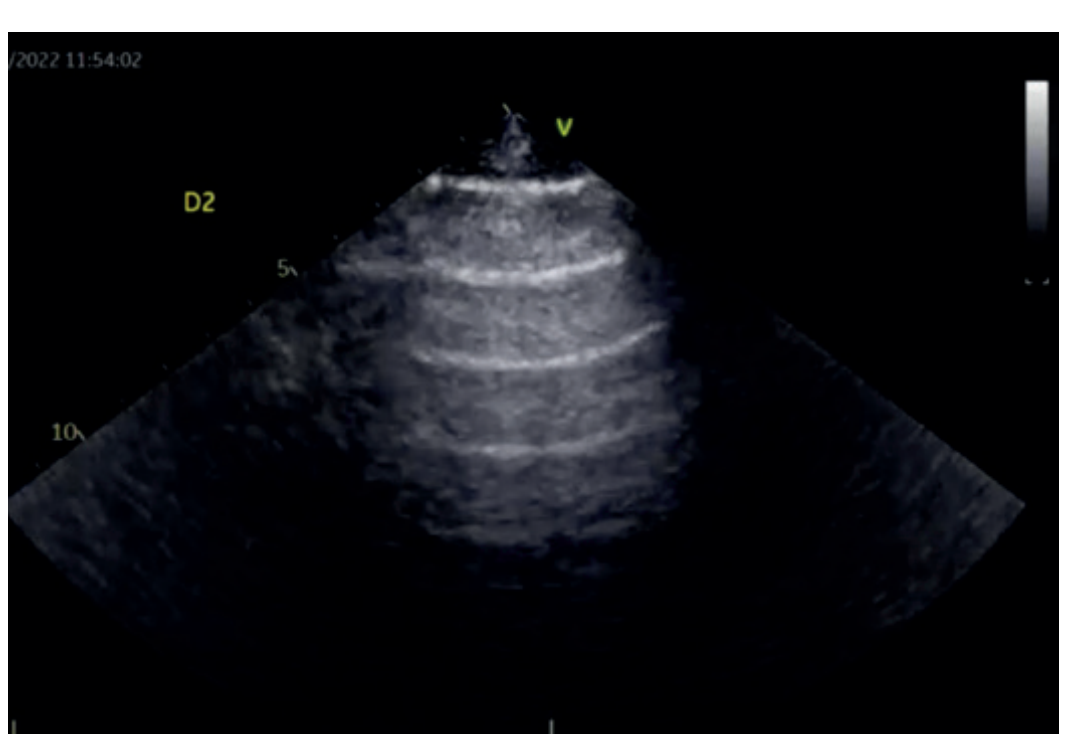

Vídeo 26 – *Linhas A caracterizadas por artefatos de reverberação horizontais a partir da linha pleural indicando pulmão aerado. Em: http://abccardiol.org/supplementary-material/2026/12304/PosicionamentoPOCUS/Video 26 US pulm padrao A.mp4*

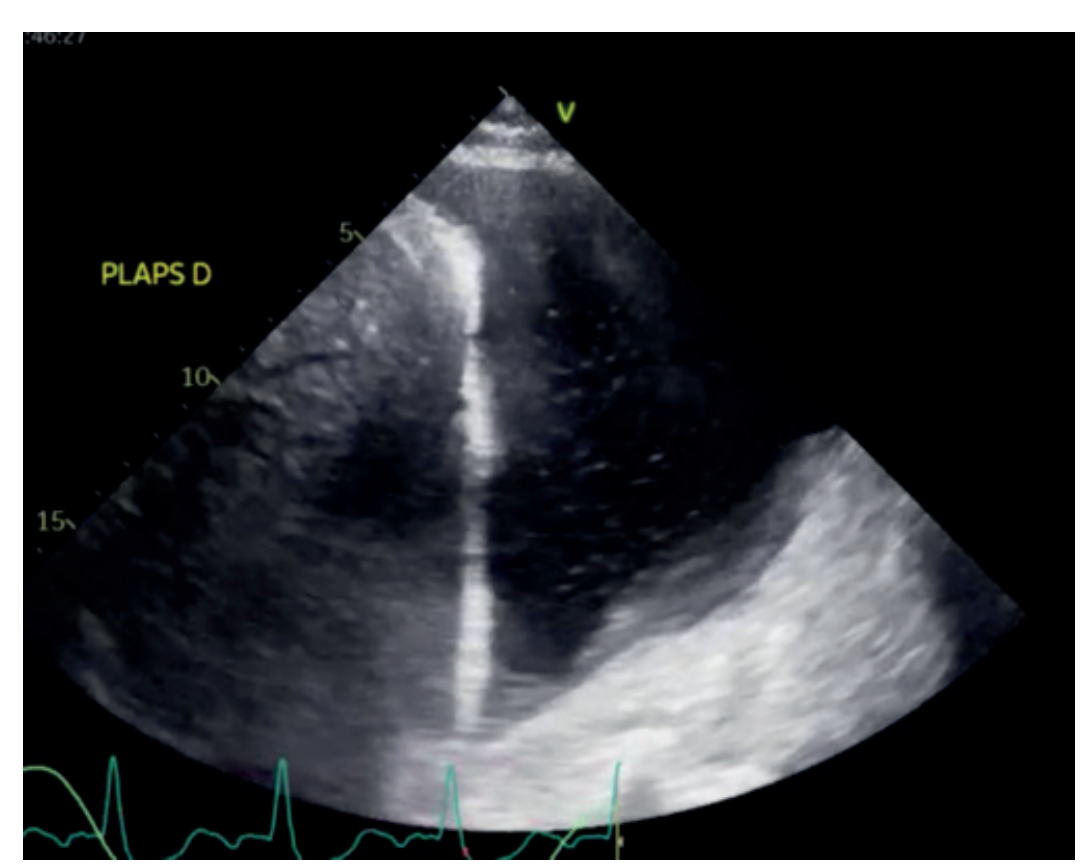

Vídeo 27 – *Sinal do plancton – ecos puntiformes e moveis no interior do líquido pleural. Podem indicar infecção , hemotórax ou pós terapia agressiva com diuréticos. Em: http://abccardiol.org/supplementary-material/2026/12304/PosicionamentoPOCUS/Video 27 US pulm sinal plancton.mp4*

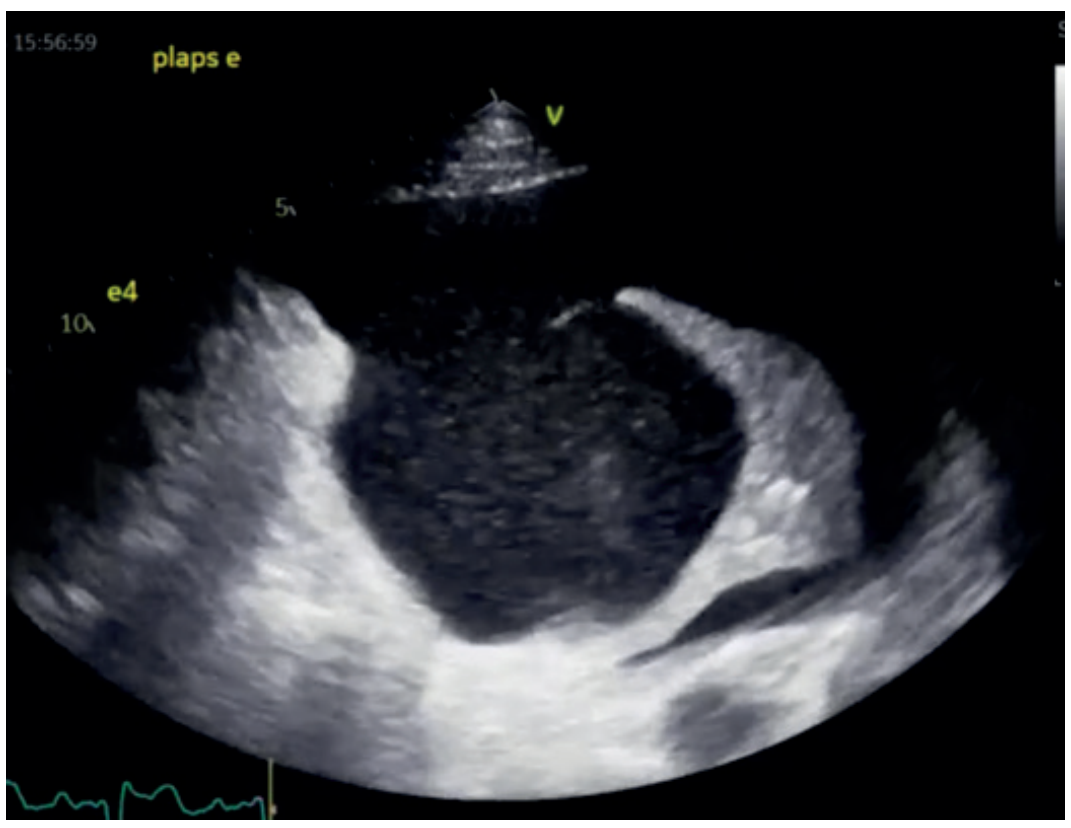

Vídeo 28 – *Sinal do plancton associado a atelectasia compressiva em volumoso derrame pleural. Em: http://abccardiol.org/supplementary-material/2026/12304/PosicionamentoPOCUS/Video 28 US pulm sinal do plancton.mp4*

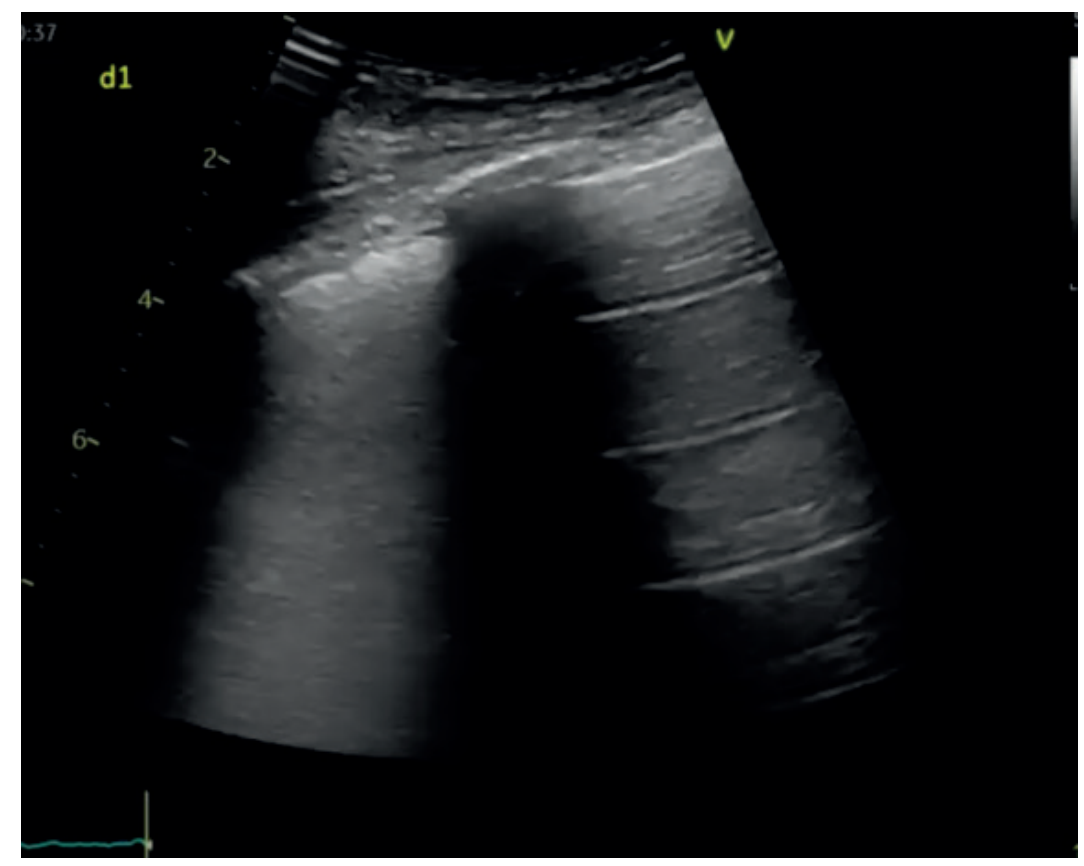

Vídeo 29 – *Linhas B com áreas poupadas (linhas A) em um mesmo campo ultrassônico em paciente com Sars-Cov2. Em: http://abccardiol.org/supplementary-material/2026/12304/PosicionamentoPOCUS/Video 29 US pulm zona poupada covid.mp4*

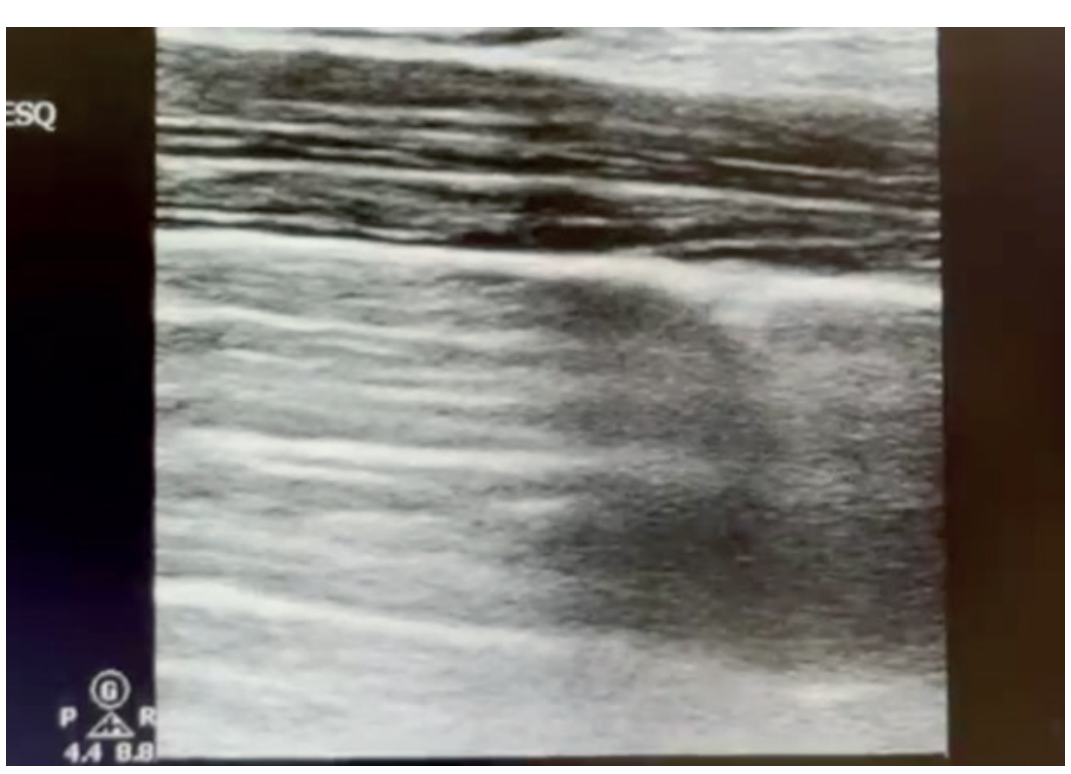

Vídeo 30 – *Ponto pulmonar. Indica o local exato da transição entre pulmão colapsado (deslizamento abolido) e pulmão normal (deslizamento presente). Achado patognomônico para pneumotórax. Em: http://abccardiol.org/supplementary-material/2026/12304/PosicionamentoPOCUS/Video 30 US pulm ponto pulmonar.mp4*

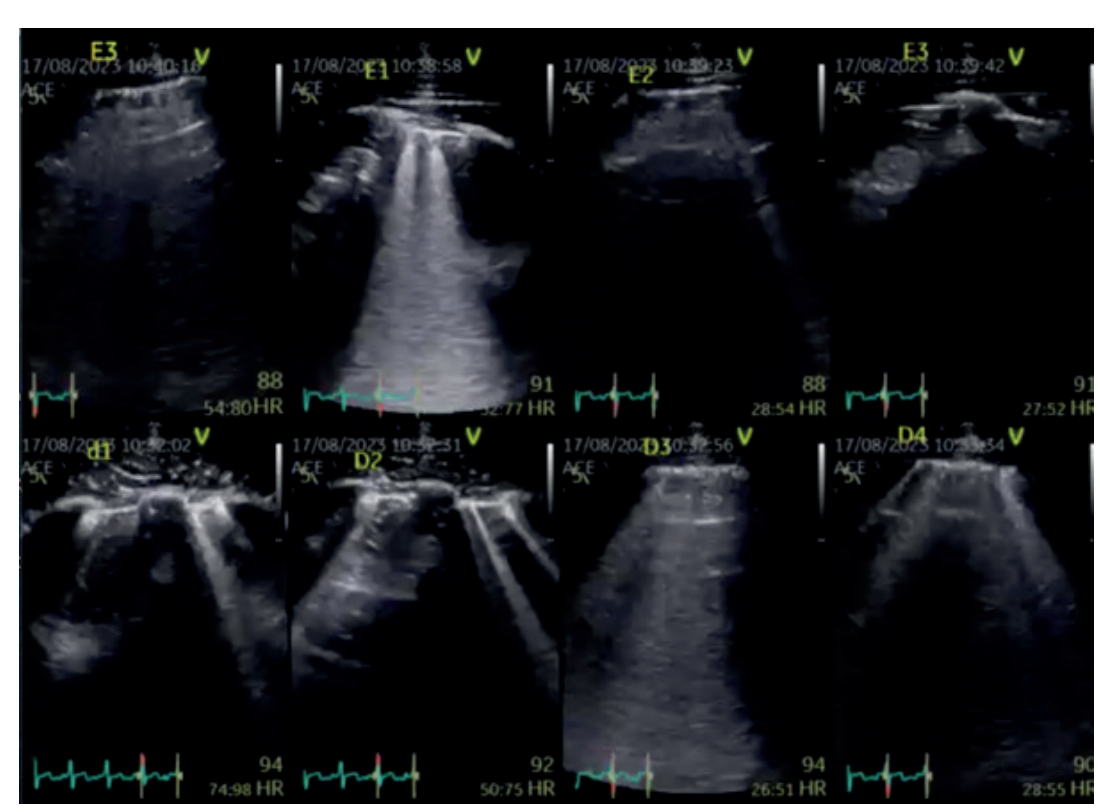

Vídeo 31 – *Paciente com quadro de IC descompensada apresentando linhas B difusas em todos os quadrantes estudados. Em: http://abccardiol.org/supplementary-material/2026/12304/PosicionamentoPOCUS/Video 31 US pulm linhas B difusas em todos quadrantes.mp4*

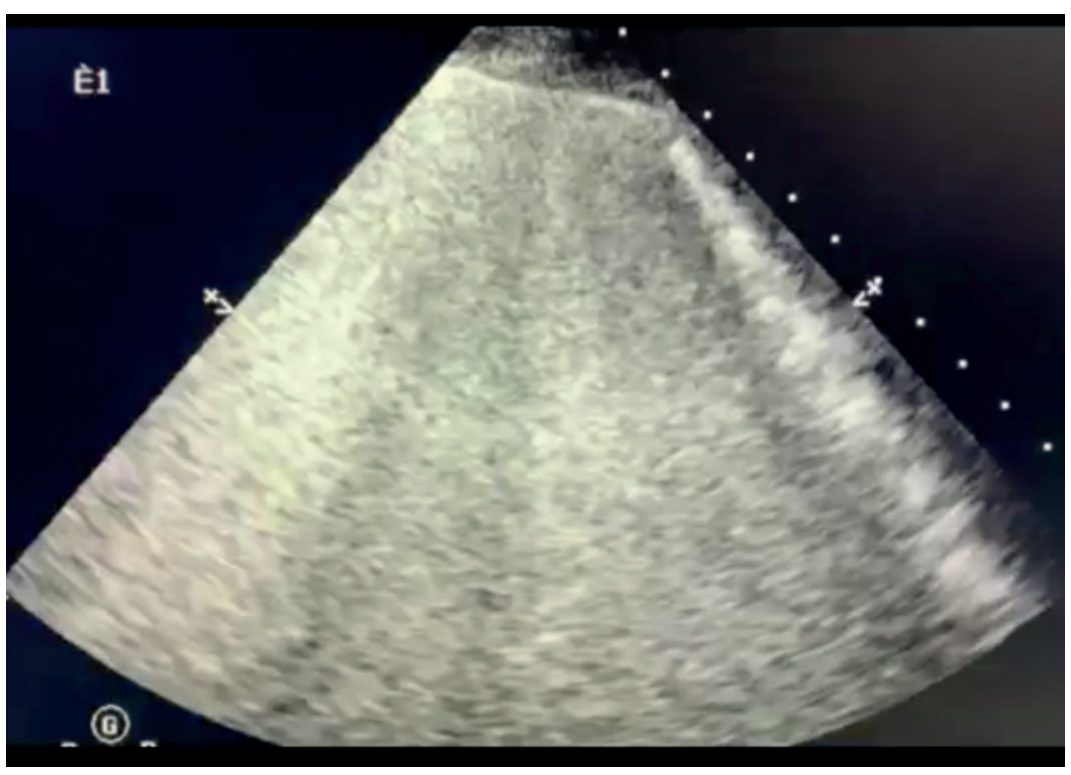

Vídeo 32 – *Paciente com quadro de IC descompensada apresentando linhas B difusas no quadrante superior esquerdo apagando as linhas A. Em: http://abccardiol.org/supplementary-material/2026/12304/PosicionamentoPOCUS/Video 32 US pulm Linhas B difusas.mp4*

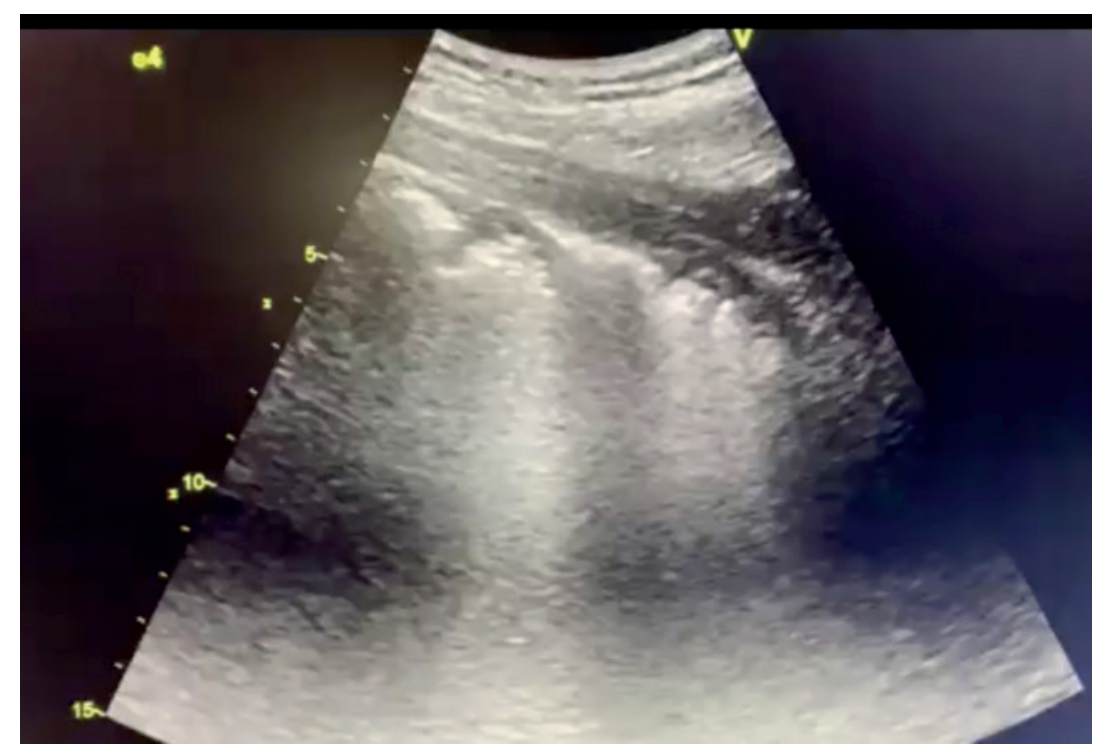

Vídeo 33 – *Consolidações subpleurais em paciente com Sars-Cov2. Em: http://abccardiol.org/supplementary-material/2026/12304/PosicionamentoPOCUS/Video 33 US pulm consolidações subpleurais.mp4*

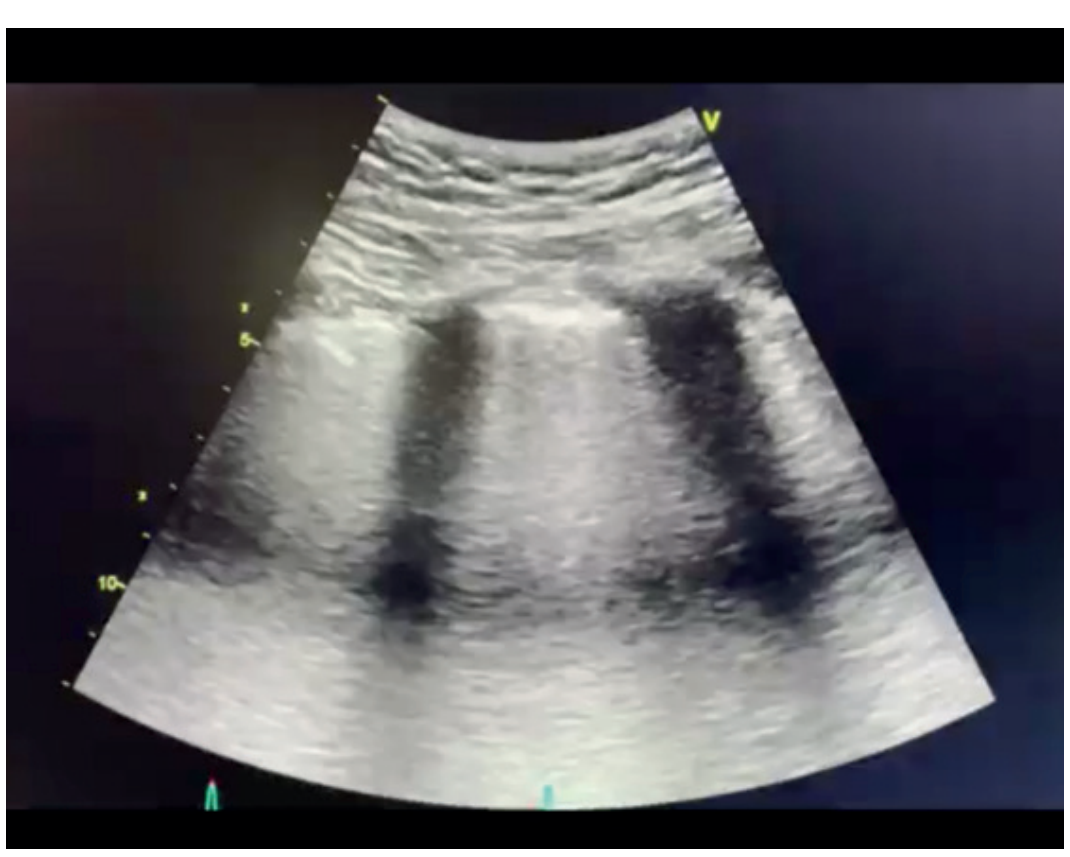

Vídeo 34 – *Linhas B coalescentes em paciente com Sars-Cov2. Em: http://abccardiol.org/supplementary-material/2026/12304/PosicionamentoPOCUS/Video 34 US pulm linhas B coalescentes.mp4*

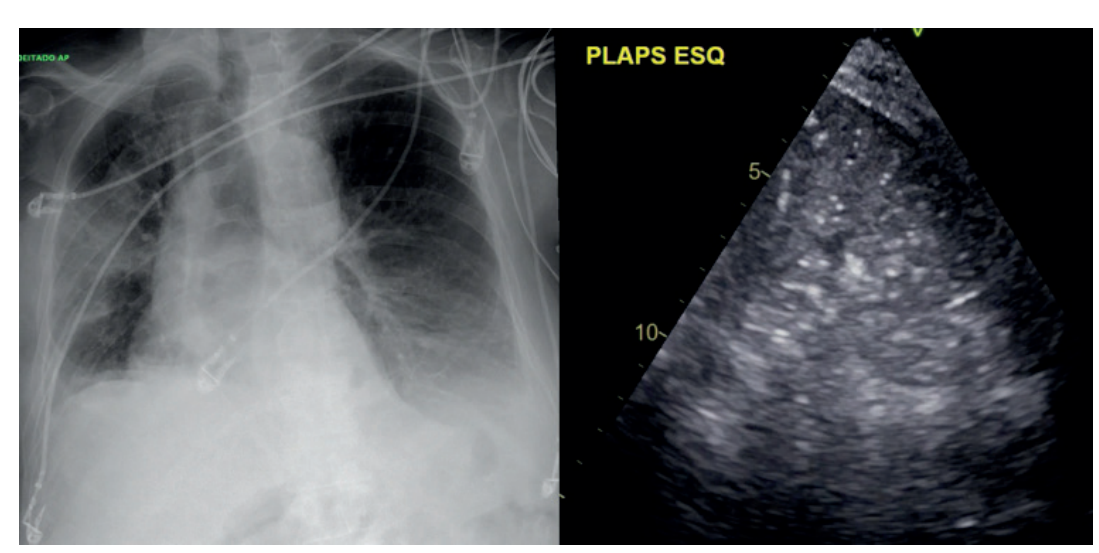

Vídeo 35 – *Consolidação da base pulmonar esquerda com broncograma aéreo e seu equivalente radiológico. Em: http://abccardiol.org/supplementary-material/2026/12304/PosicionamentoPOCUS/Video 35 US pulm consolidação pulm com broncograma aereo.mp4*

Suplemento 2

Posicionamento POCUS – VIDEOS Reunidos

SESSÃO 4 – VExUS

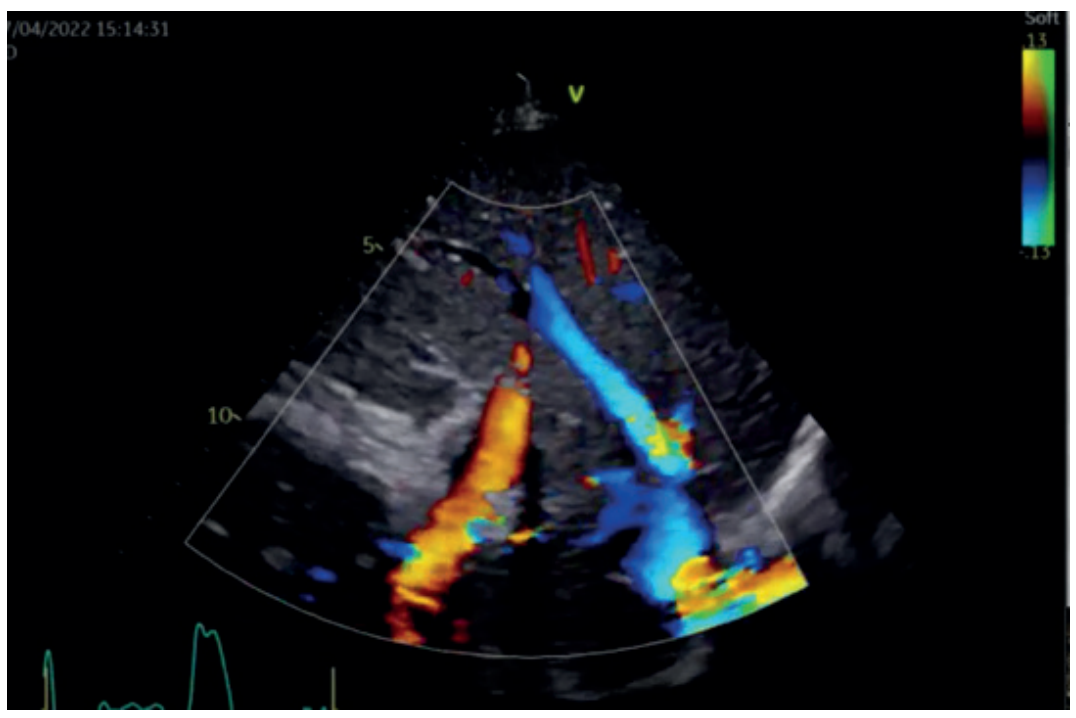

Vídeo 36 – *Veia suprahepatica (fluxo em azul) e veia porta (fluxo em vermelho). Em: http://abccardiol.org/supplementary-material/2026/12304/PosicionamentoPOCUS/Video 36 VEXUS Janela transhepática padrão normal.mp4*

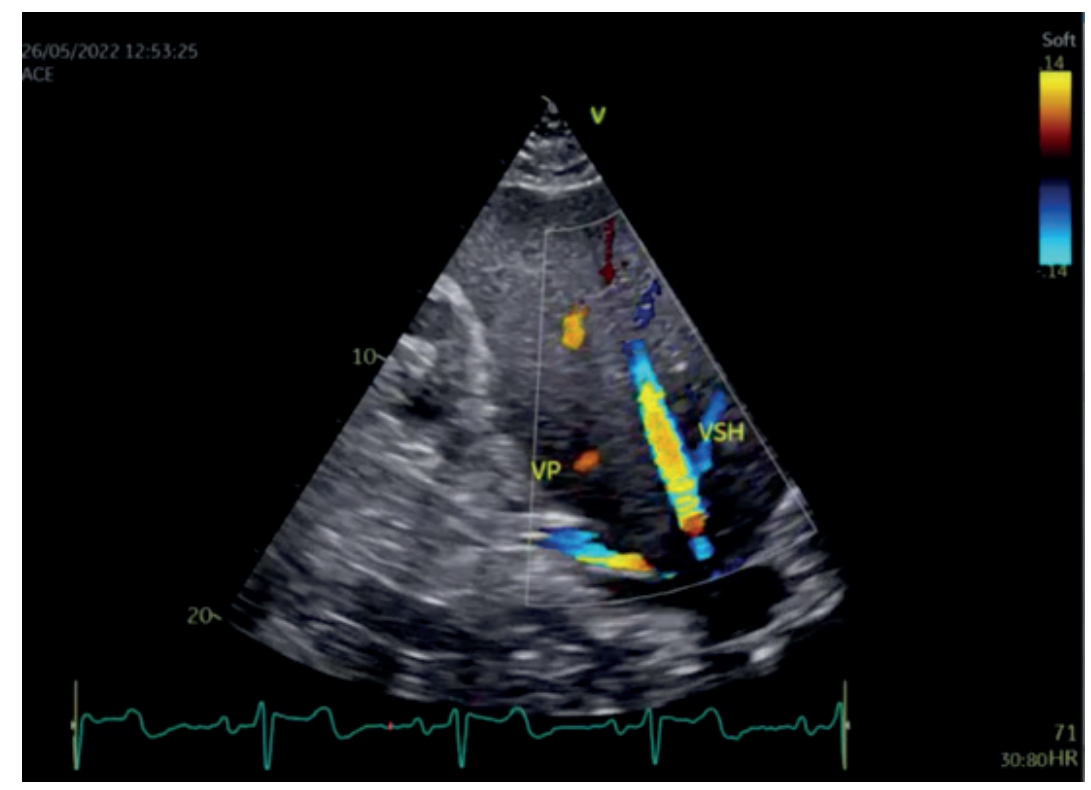

Vídeo 37 – *Janela transhepática padrão normal com movimento de báscula anteriorizando o transdutor para visualizar a veia porta (vermelho). Em: http://abccardiol.org/supplementary-material/2026/12304/PosicionamentoPOCUS/Video 37 VEXUS Janela transhepática padrão normal.mp4*

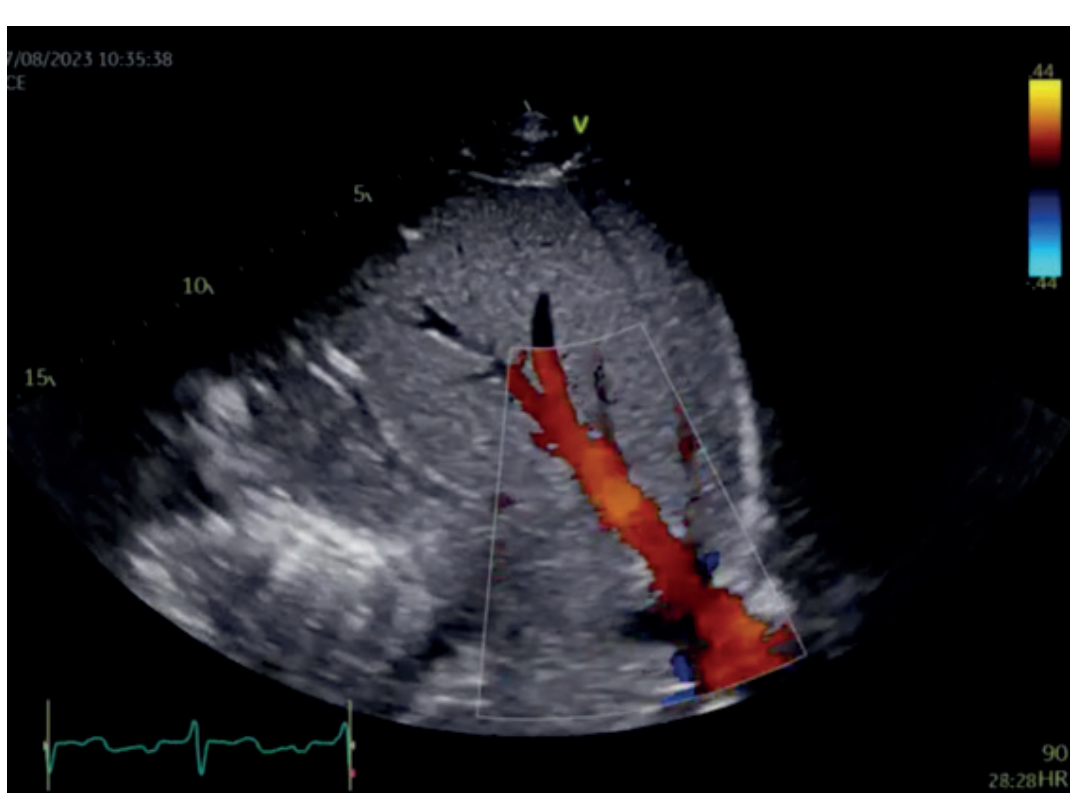

Vídeo 38 – *VEXUS Janela transhepática padrão alterado com de veia suprahepatica apresentando inversão sistólica(vermelho) equivalente ao reverso sistólico na curva espectral. Em: http://abccardiol.org/supplementary-material/2026/12304/PosicionamentoPOCUS/Video 38 VEXUS Janela transhepática padrão alterado.mp4*

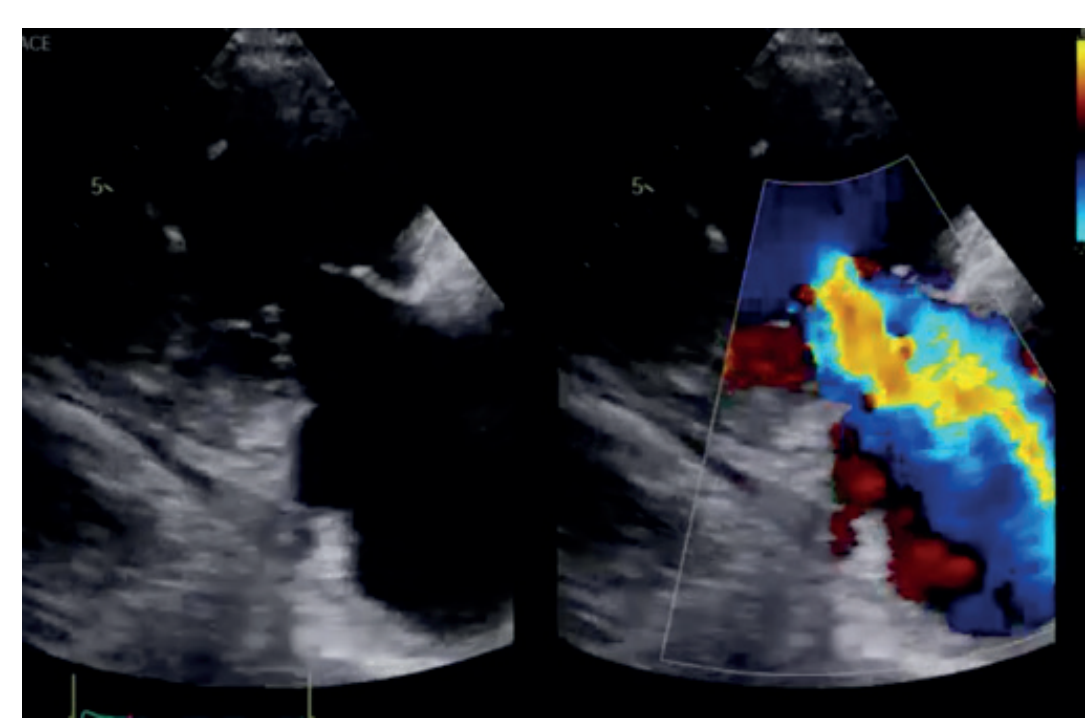

Vídeo 39 – *Regurgitação tricúspide torrencial gerando congestão venosa sistêmica importante. Em: http://abccardiol.org/supplementary-material/2026/12304/PosicionamentoPOCUS/Video 39 VEXUS IT torrencial1.mp4*

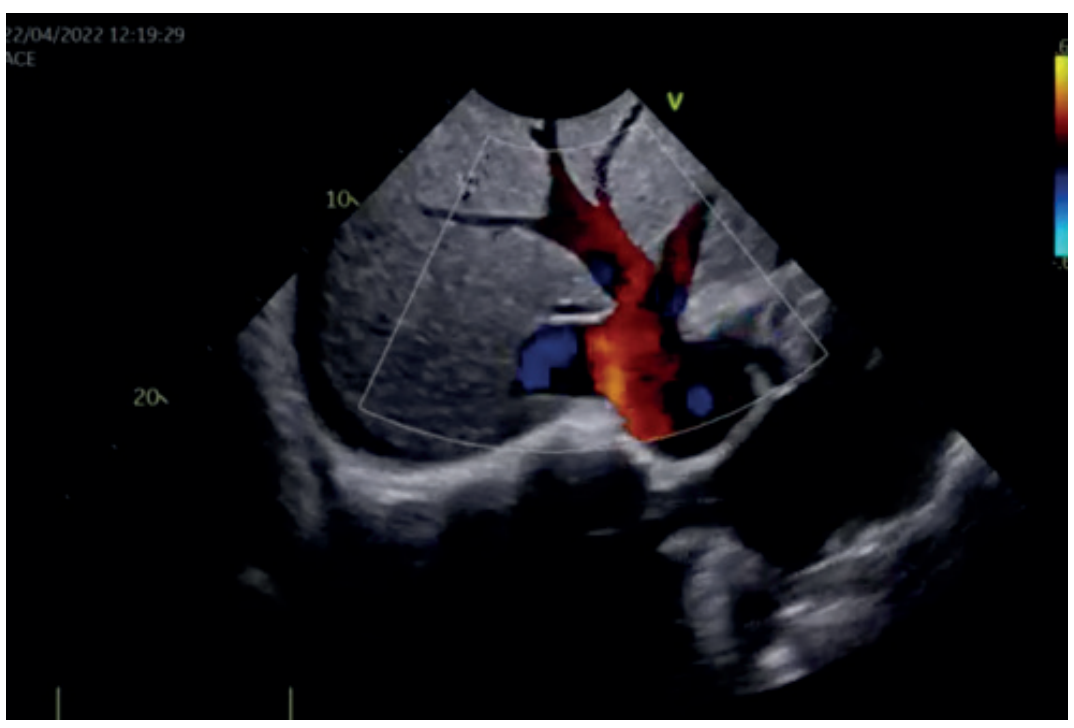

Vídeo 40 – *Regurgitação tricúspide torrencial gerando congestão venosa sistêmica importante caracterizado por veias cava inferior e suprahepaticas marcadamente dilatadas e com fluxo reverso assim como a presença de ascite. Em: http://abccardiol.org/supplementary-material/2026/12304/PosicionamentoPOCUS/Video 40 VEXUS IT torrencial2.mp4*

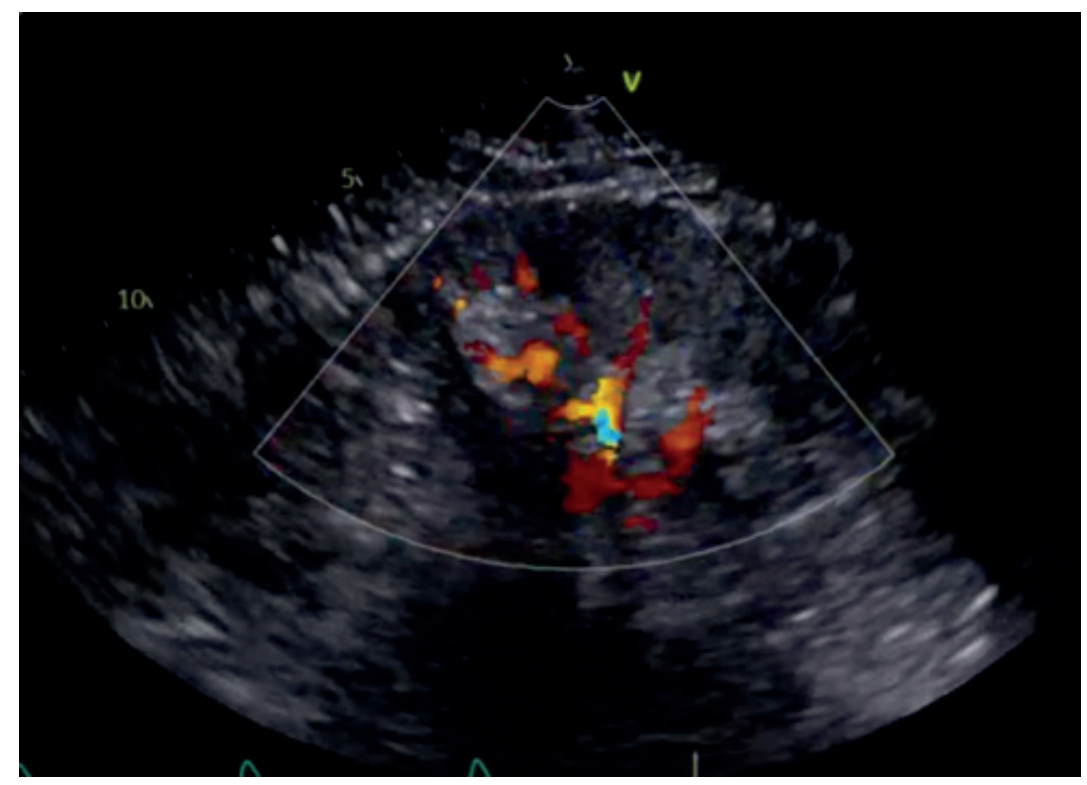

Vídeo 41 – *Fluxo intraparenquimatoso renal com obtenção do fluxo da veia lobar renal ao nível da transição da medula para a córtex do rim. Em: http://abccardiol.org/supplementary-material/2026/12304/PosicionamentoPOCUS/Video 41 VEXUS Fluxo veia lobar renal.mp4*

Suplemento 2

Posicionamento POCUS – VIDEOS Reunidos

SESSÃO 5 – POCUS na TVP

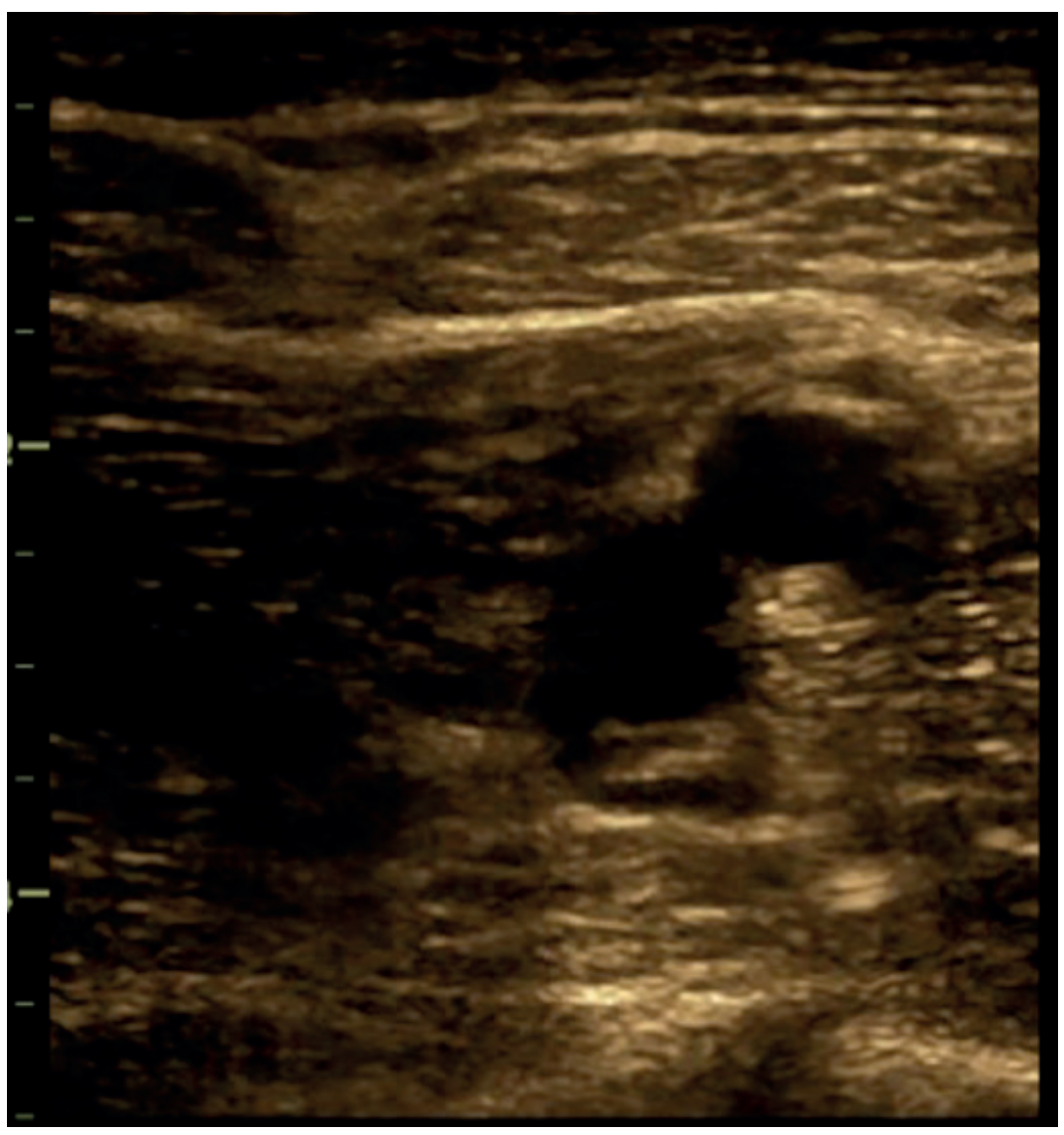

Vídeo 42 – *Protocolo de compressão para pesquisa de TVP. Compressão da veia femoral. Em: http://abccardiol.org/supplementary-material/2026/12304/PosicionamentoPOCUS/Video 42 veia femoral com compressão.mp4*

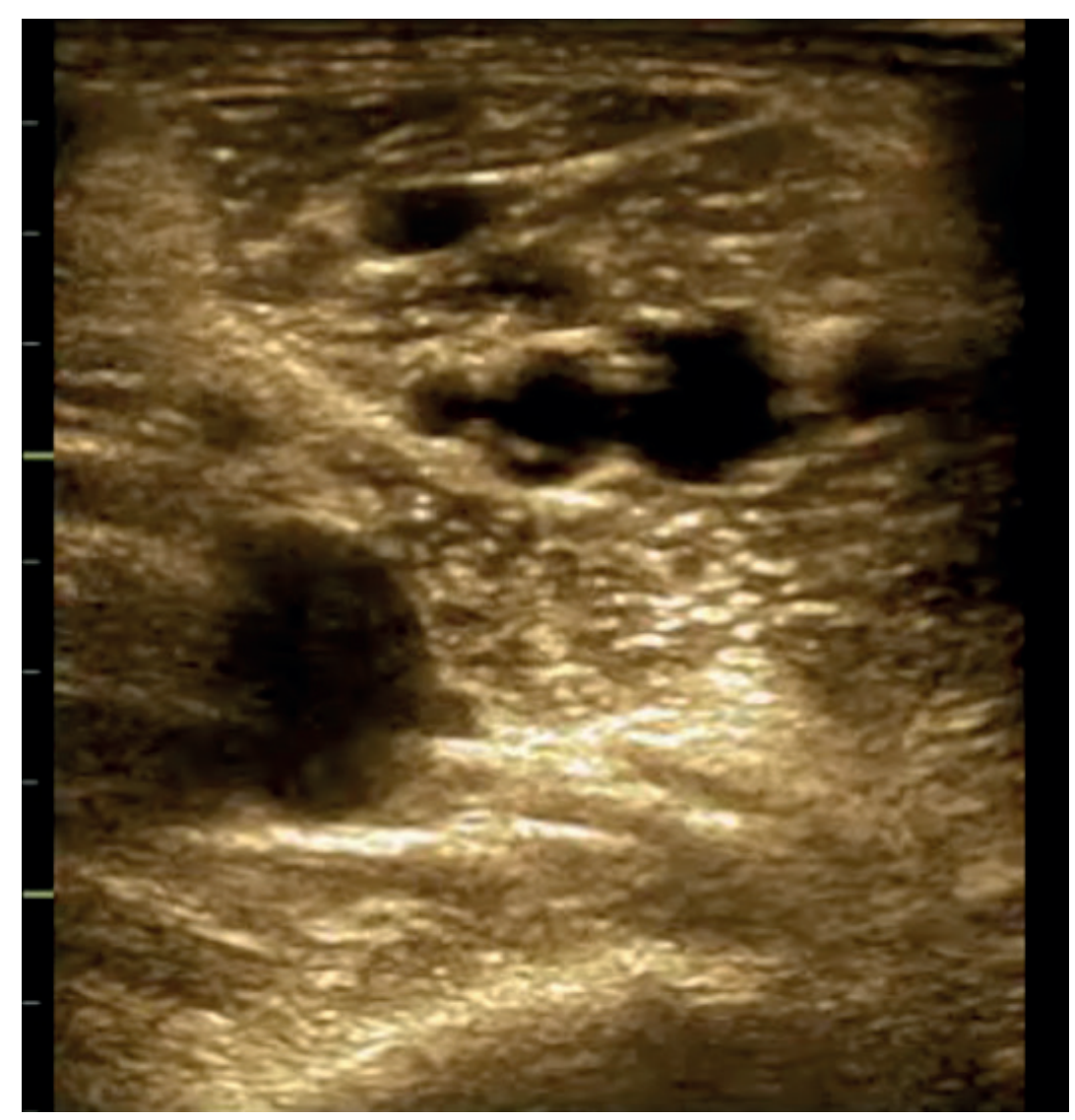

Vídeo 43 – *Protocolo de compressão para pesquisa de TVP. Compressão das veias musculares da panturrilha sem trombose e em um plano mais profundo da veia poplítea incompressível com trombose. Em: http://abccardiol.org/supplementary-material/2026/12304/PosicionamentoPOCUS/Video 43 gastrocnemia com compressão TVP poplitea.mp4*

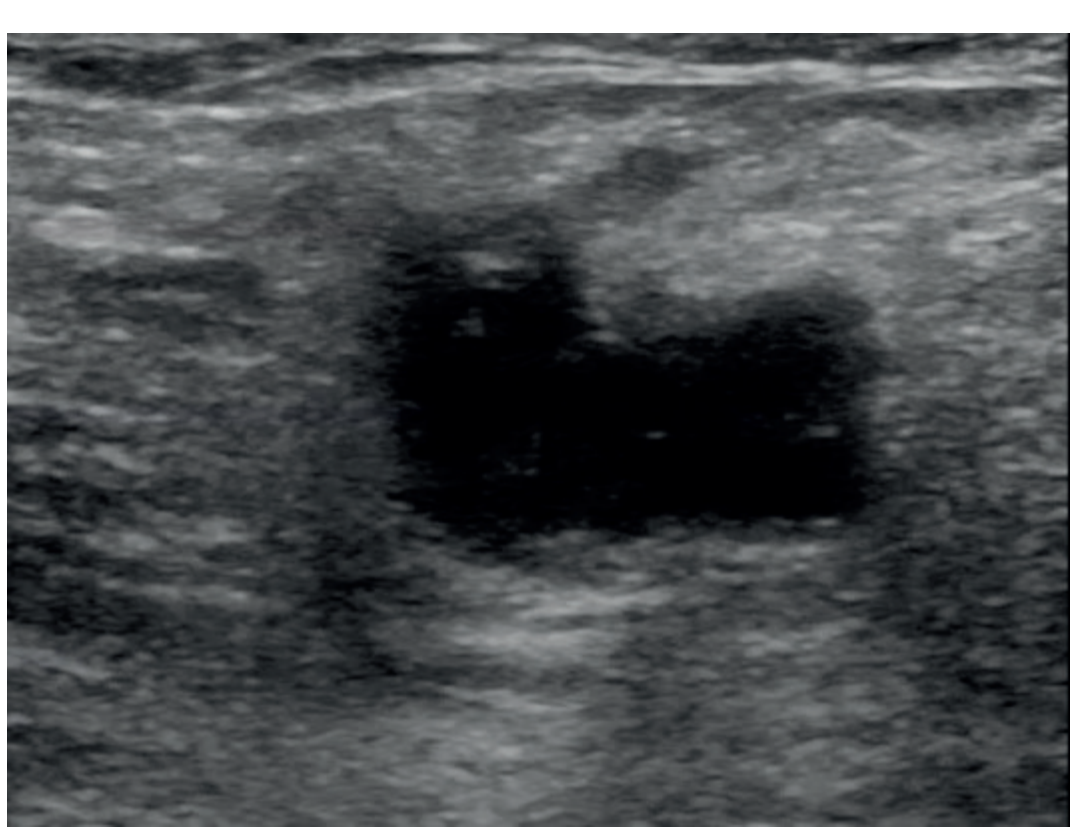

Vídeo 44 – *Protocolo de compressão para pesquisa de TVP. Veia femoral incompressível por trombo. Em: http://abccardiol.org/supplementary-material/2026/12304/PosicionamentoPOCUS/Video 44 TVP femoral compressão.mp4*

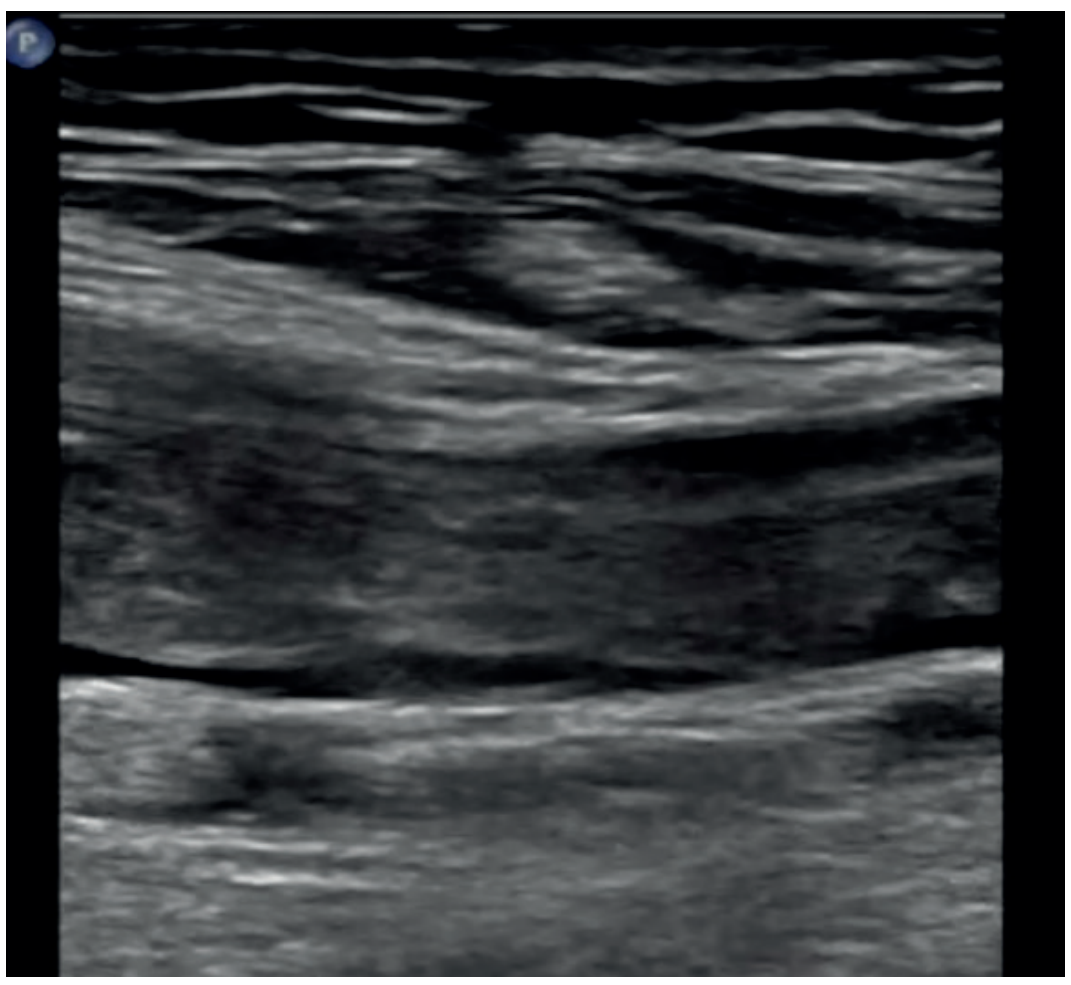

Vídeo 45 – *Trombo flutuante em veia femoral eixo longitudinal. Em: http://abccardiol.org/supplementary-material/2026/12304/PosicionamentoPOCUS/Video 45 Trombo flutuante veia femoral.mp4*

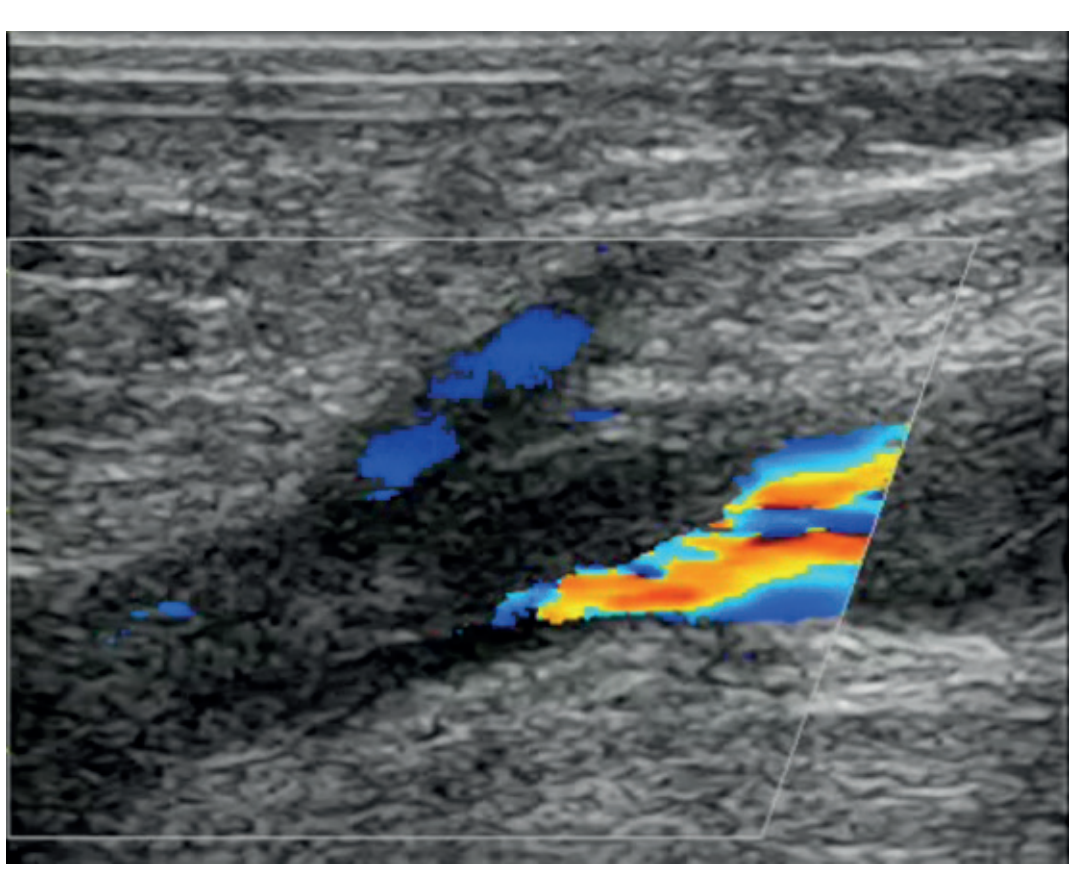

Vídeo 47 – *Trombo móvel em veia femoral com fluxo de permeio ao color Doppler. Em: http://abccardiol.org/supplementary-material/2026/12304/PosicionamentoPOCUS/Video 47 TVP veia femoral color.mp4*

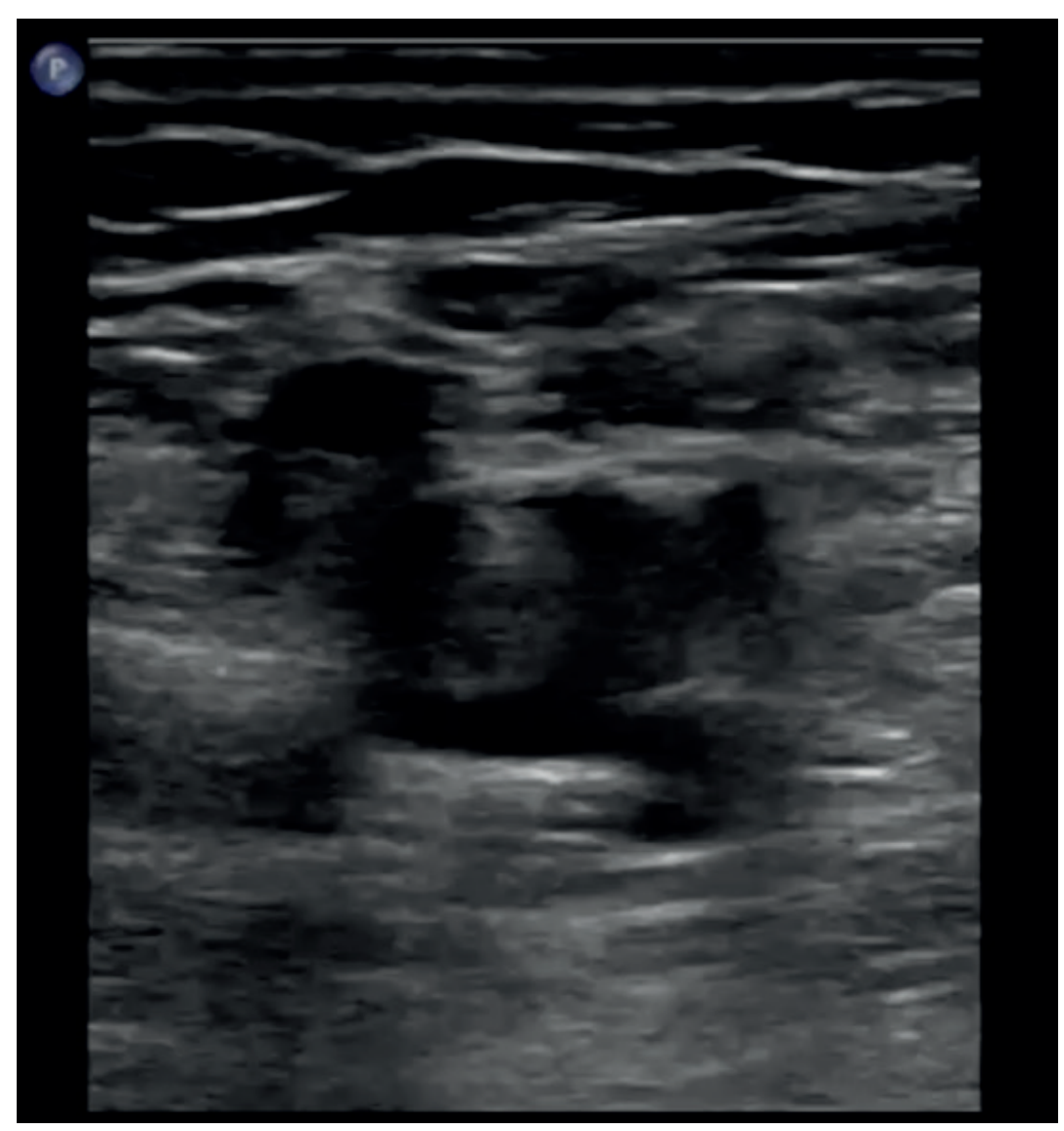

Vídeo 46 – *Trombo flutuante em veia femoral eixo curto. Em: http://abccardiol.org/supplementary-material/2026/12304/PosicionamentoPOCUS/Video 46 Trombo flutuante veia femoral eixo curto.mp4*

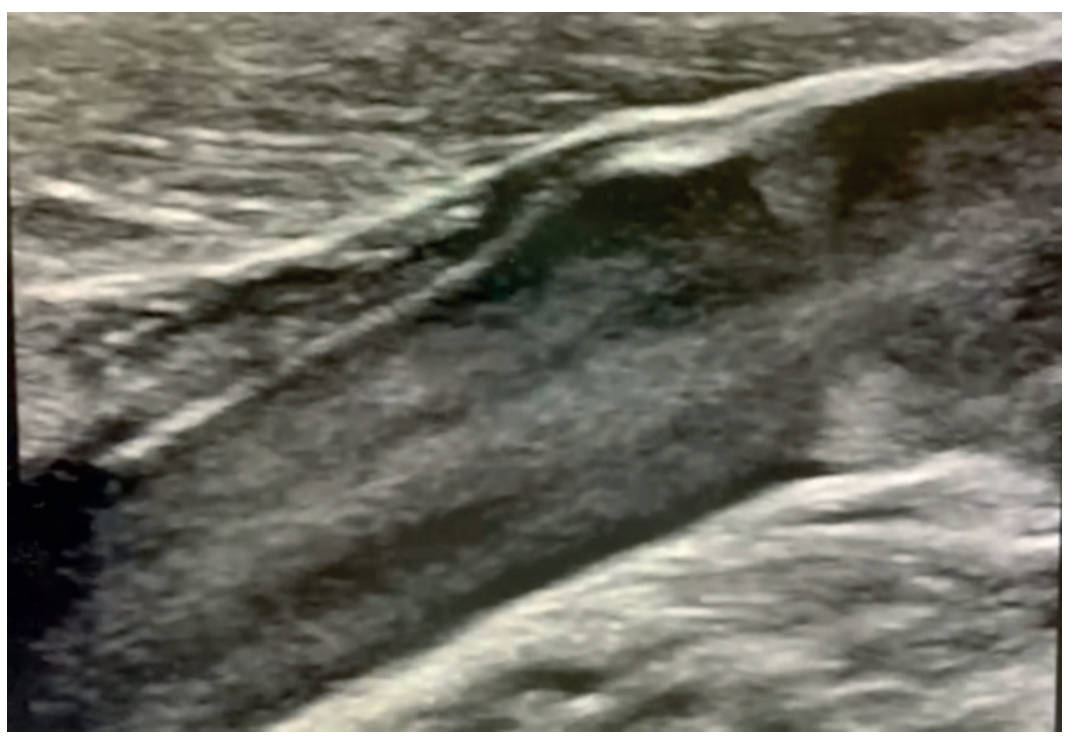

Vídeo 48 – *Estase venosa mimetizando trombo especialmente ao nível das valvas. Em: http://abccardiol.org/supplementary-material/2026/12304/PosicionamentoPOCUS/Video 48 Duplex venoso com estase venosa.MP4*

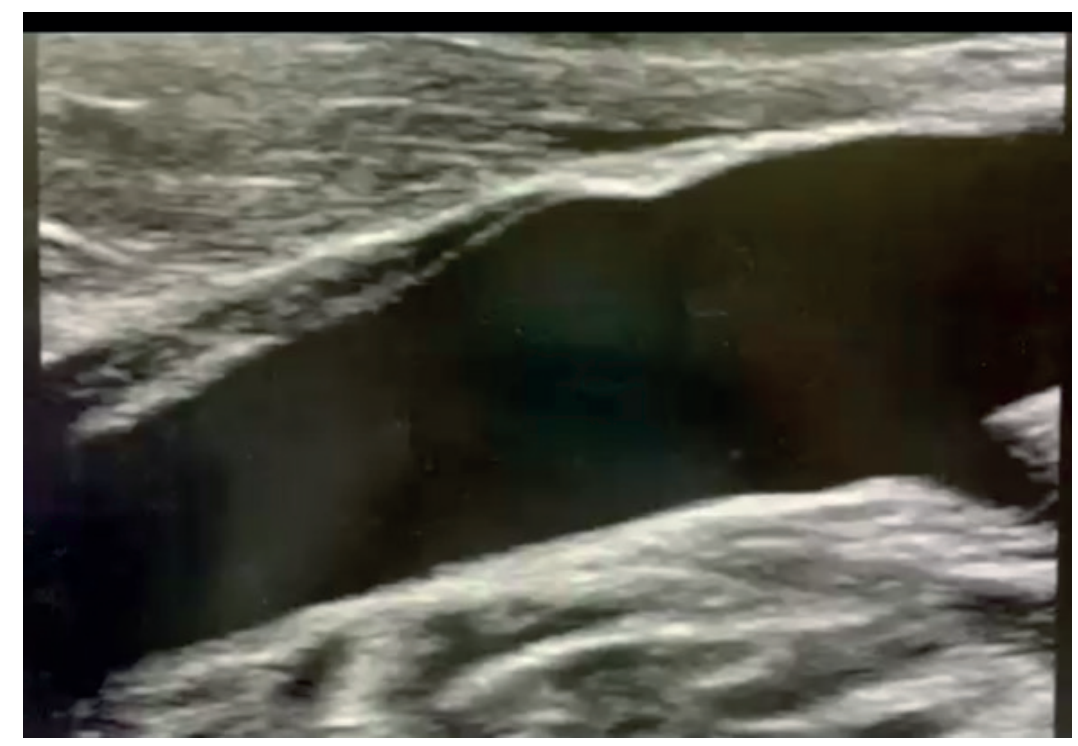

Vídeo 49 – *Desaparecimento da estase venosa após compressão com o transdutor. Em: http://abccardiol.org/supplementary-material/2026/12304/PosicionamentoPOCUS/Video 49 Duplex apos compressão sem estase venosa.mp4*

Suplemento 2

Posicionamento POCUS – VIDEOS Reunidos

SESSÃO 6 – POCUS no Choque

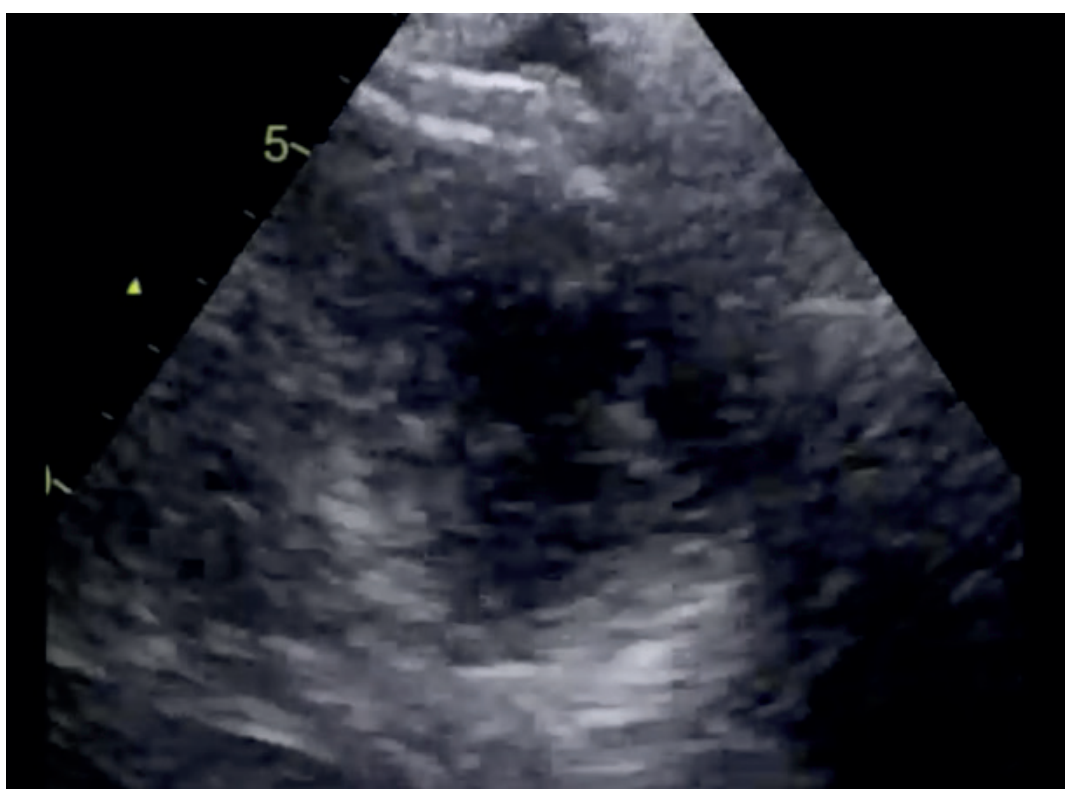

Vídeo 50 – *VE hiperdinâmico na fase precoce de um paciente com choque séptico apresentando cavidades ventriculares reduzidas com aproximação dos músculos papilares e paredes* Kissing Walls*. Em: http://abccardiol.org/supplementary-material/2026/12304/PosicionamentoPOCUS/Video 50 Choque septico VE hiperdinamico eixo curto kissing walls.mp4*

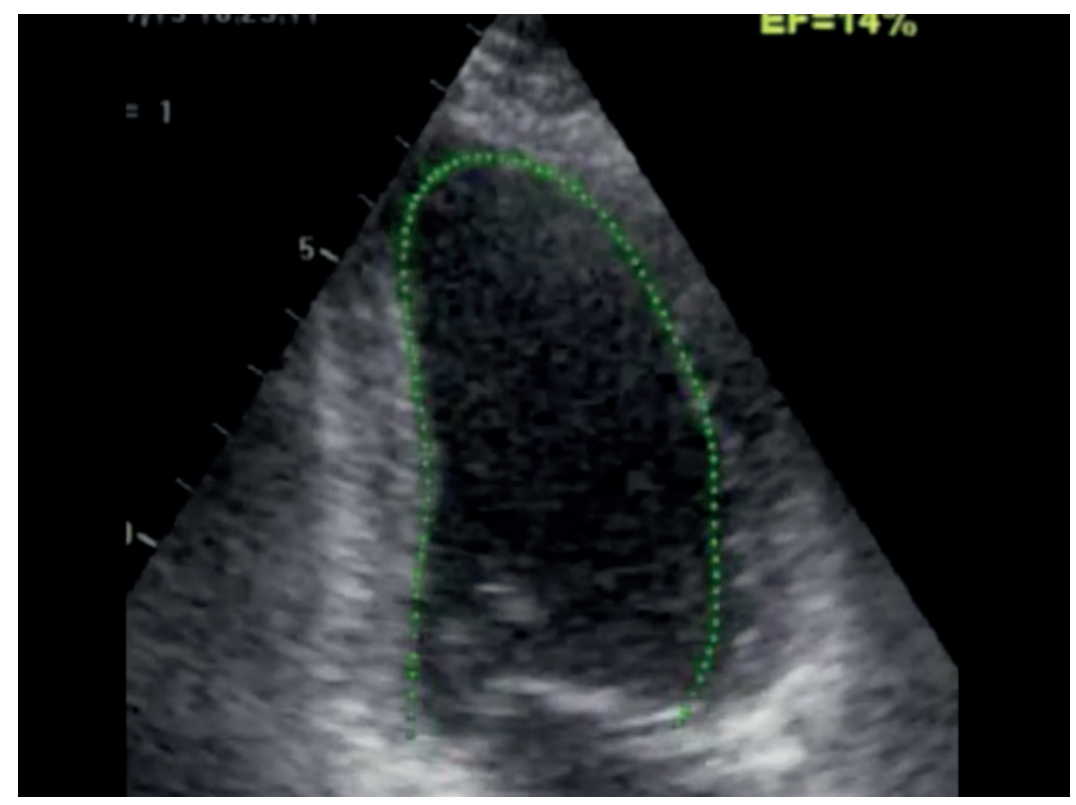

Vídeo 51 – *Paciente admitida por SARS-Cov-2 apresentando quadro de choque cardiogênico por IAM complicado por Cardiomiopatia Adrenérgica (Tako Tsubo). Exame pré instalação de ECMO-VA. Em: http://abccardiol.org/supplementary-material/2026/12304/PosicionamentoPOCUS/Video 51 Choque cardiogênico disfunção grave VE.mp4*

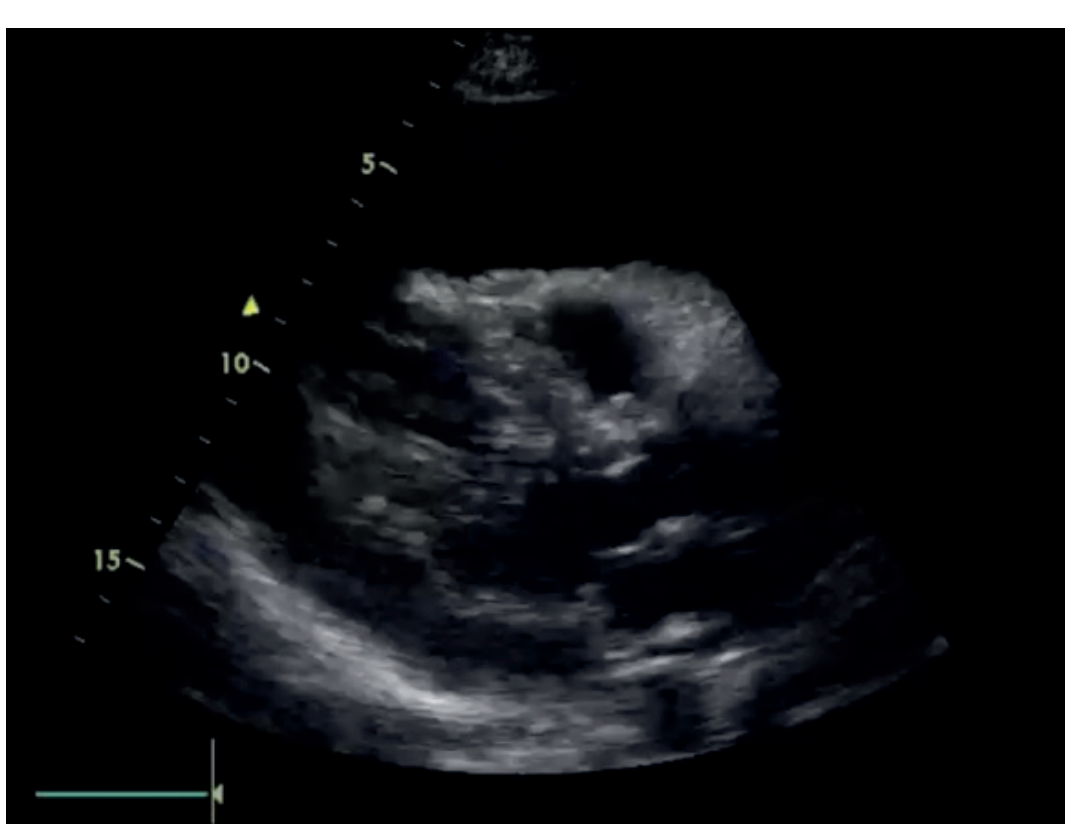

Vídeo 52 – *Volumoso derrame pericárdico gerando movimento oscilante do coração no interior do saco pericárdico* swinging heart*. Em: http://abccardiol.org/supplementary-material/2026/12304/PosicionamentoPOCUS/Video 52 Derrame pericardico volumoso com swinging heart.mp4*

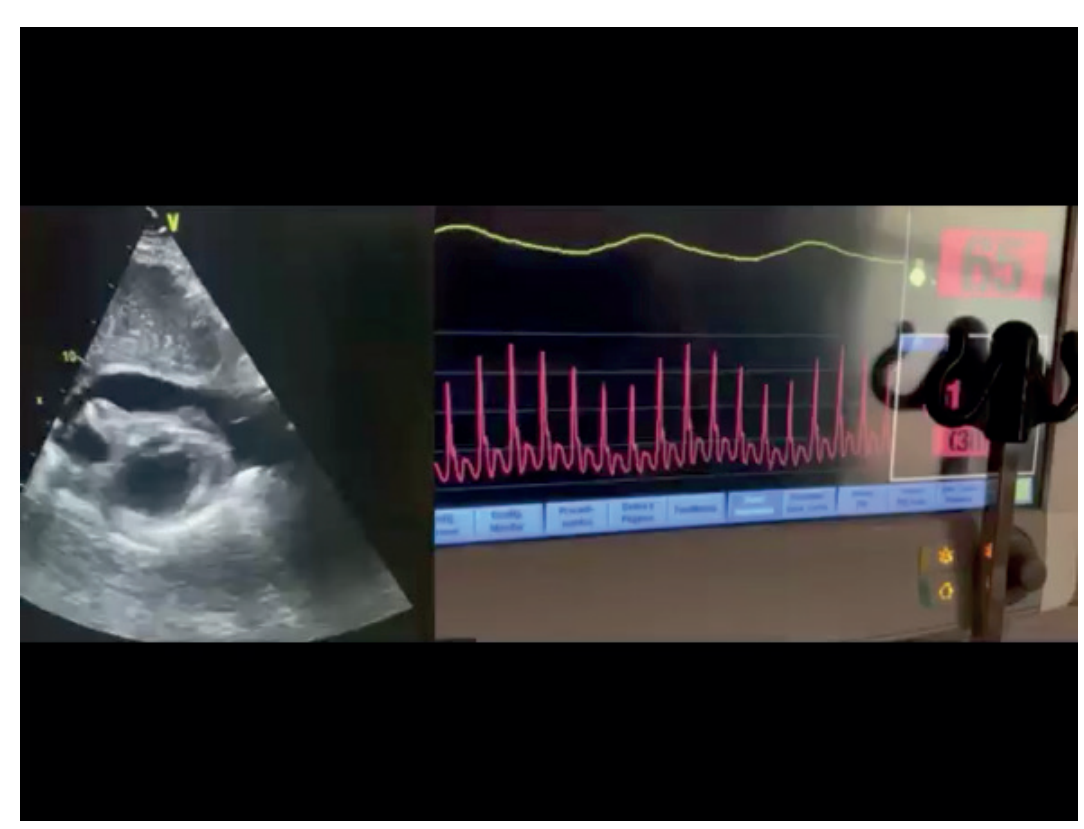

Vídeo 53 – *Paciente internada com quadro de SARS-Cov-2 evolui com choque. Eco demonstra volumoso derrame pericárdio gerando colabamento das cavidades direitas e distensão da veia cava inferior. Ao monitor observa-se alternância elétrica gerada pelo swinging heart e PAS<40mmHg. Em: http://abccardiol.org/supplementary-material/2026/12304/PosicionamentoPOCUS/Video 53 Choque obstrutivo tamponamento cardiaco.mp4*

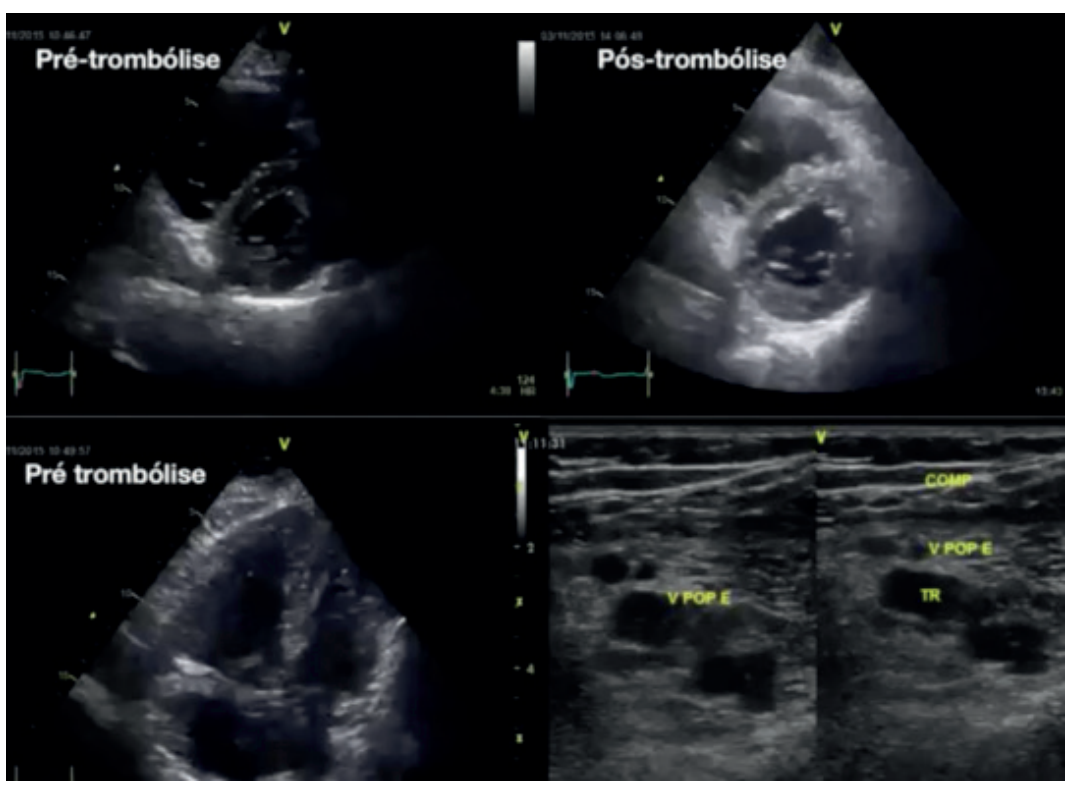

Vídeo 54 – *Paciente admitido na emergência com taquidispneia, sudorese e extremidades frias. PA: 80x50mmHg - FC:120bpm - Sat 89% -Troponina 0,337ng/ml. Eco mostra dilatação e disfunção do VD com achatamento do septo e sinal de McConnell . AngioTC confirmou TEP maciço submetido a trombólise sistêmica com ATPlase com resolução dos achados. Em: http://abccardiol.org/supplementary-material/2026/12304/PosicionamentoPOCUS/Video 54 Choque obstrutivo TEP maciço trombólise.mp4*

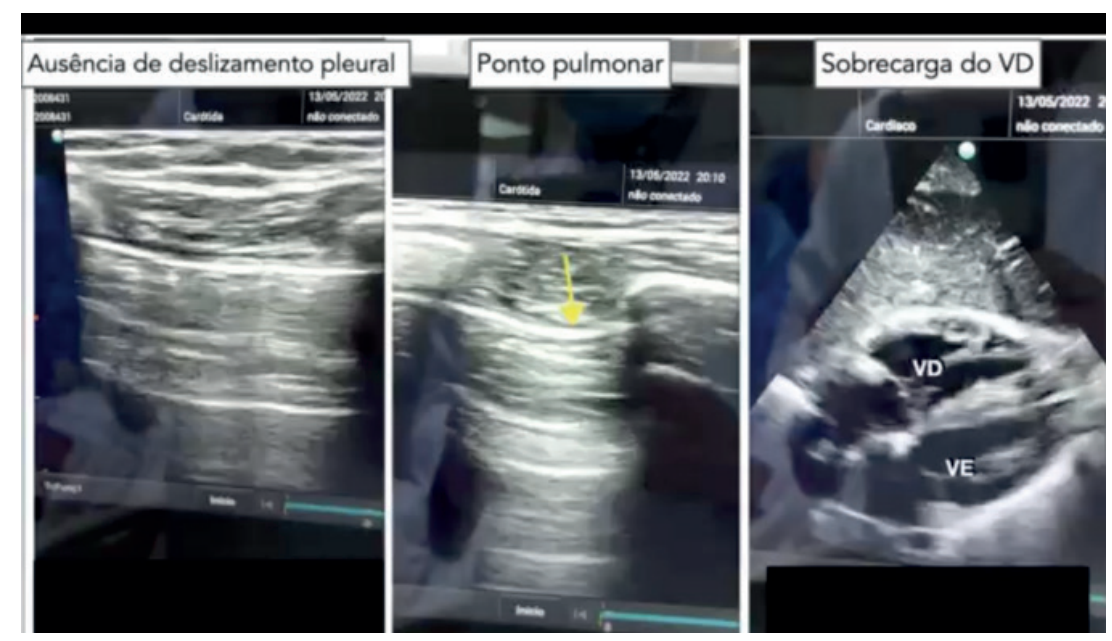

Vídeo 55 – *Paciente internado na enfermaria para drenagem de pneumotórax espontâneo por bolhas subpleurais apresenta quadro de dispneia e hipotensão POCUS cardio-pulmonar no hemitórax contralateral ao já drenado demonstrando a tríade clássica com ausência de deslizamento pleural, ponto pulmonar (*lung point*) e sobrecarga do VD. Em: http://abccardiol.org/supplementary-material/2026/12304/PosicionamentoPOCUS/Video 55 choque obstrutivo pneumotorax hipertensivo.mp4*

Suplemento 2

Posicionamento POCUS – VIDEOS Reunidos

SESSÃO 7 – Dispositivos circulatórios de curto prazo

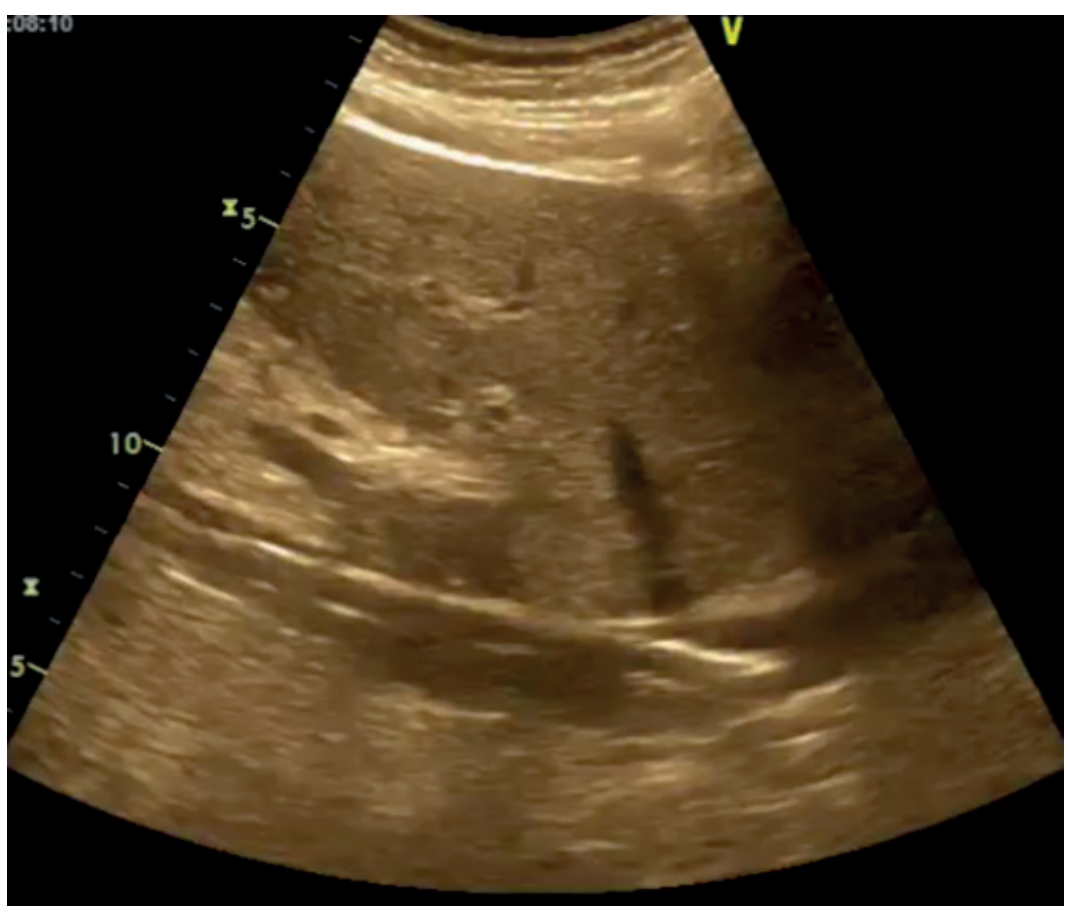

Vídeo 56 – *Cânula de ECMO na VCI próxima ao desague da veia supra-hepática. Em: http://abccardiol.org/supplementary-material/2026/12304/PosicionamentoPOCUS/Video 56 Cânula ECMO VCI.mp4*

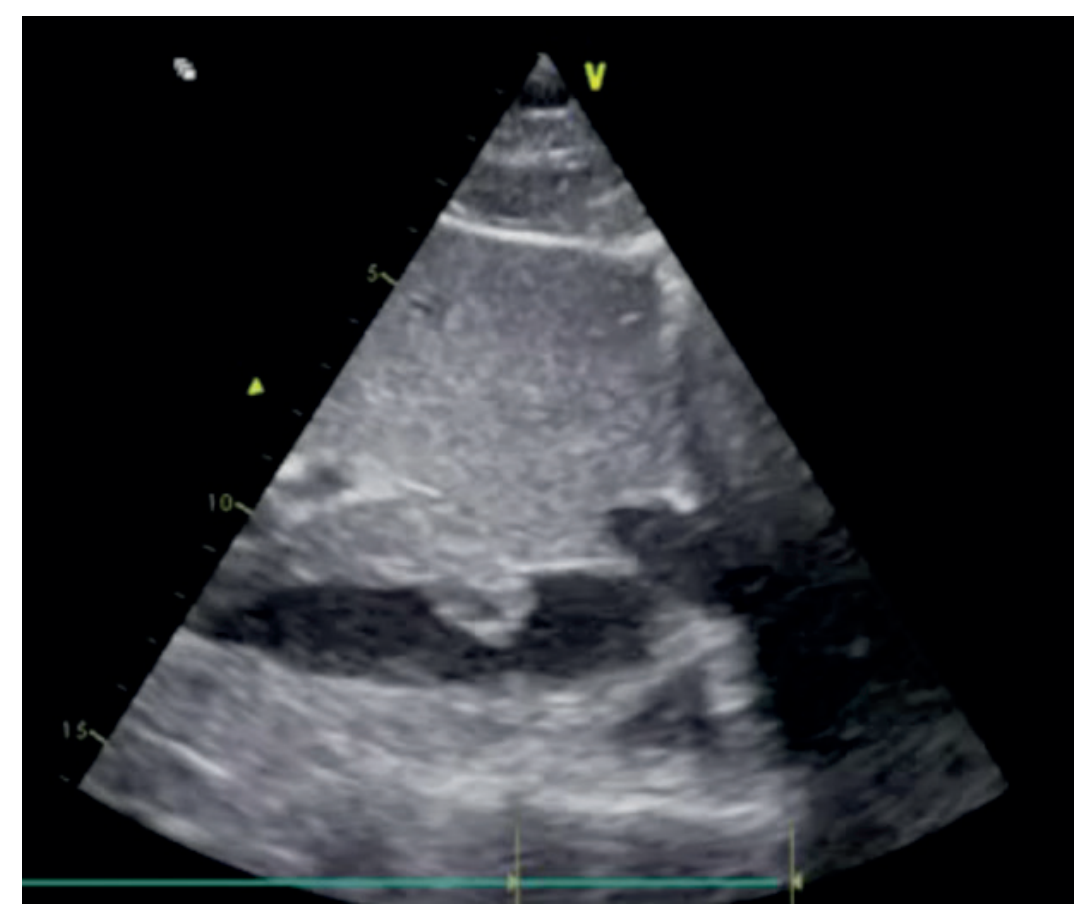

Vídeo 57 – *Trombo na parede da VCI após retirada da cânula de ECMO. Em: http://abccardiol.org/supplementary-material/2026/12304/PosicionamentoPOCUS/Video 57 Trombo VCI pos retirada cânula ECMO.mp4*

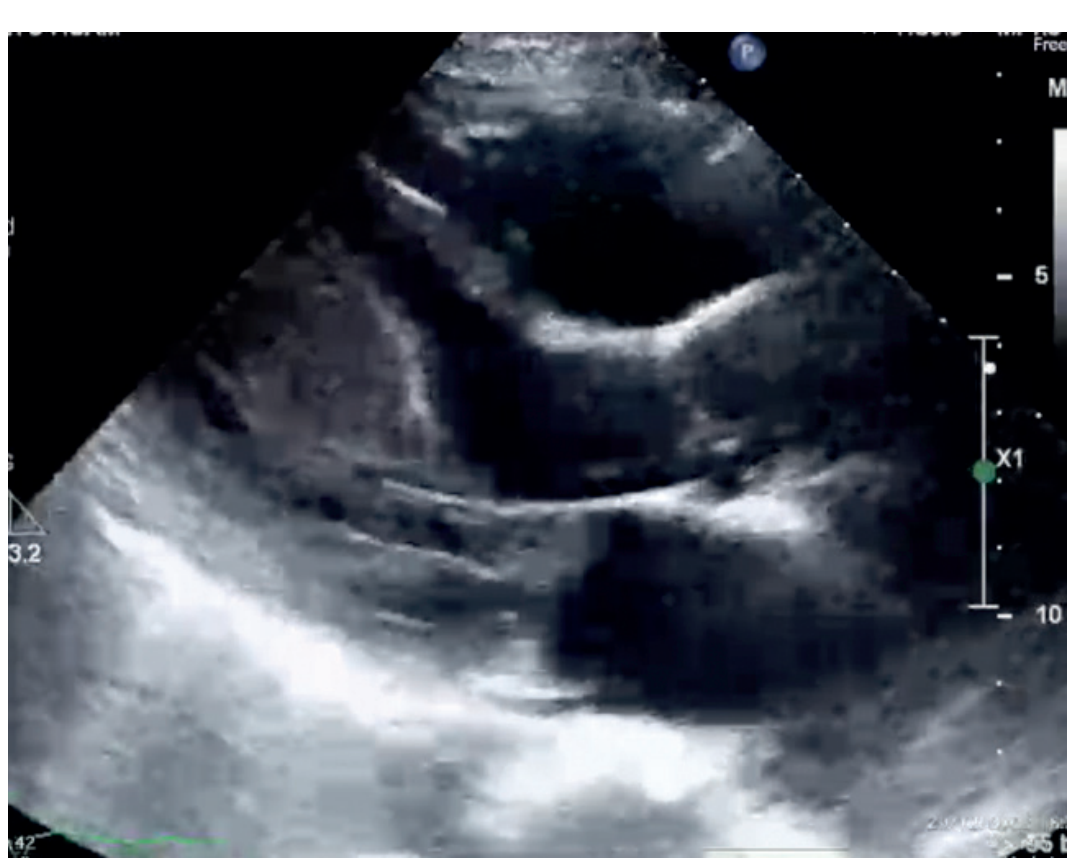

Vídeo 58 – *Trombo intraventricular em paciente em ECMO VA. Corte longitudinal. Em: http://abccardiol.org/supplementary-material/2026/12304/PosicionamentoPOCUS/Video 58 ECMO VA trombose intraventricular PLAX.mp4*

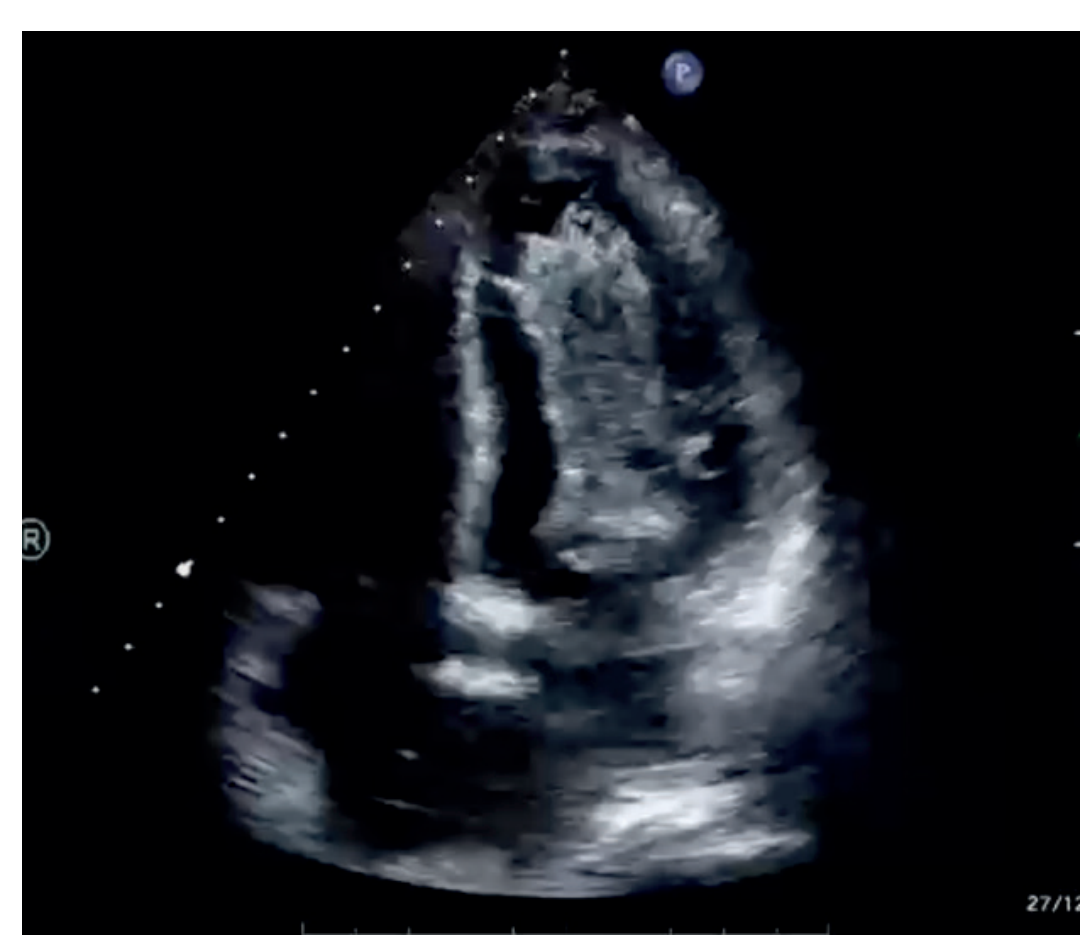

Vídeo 59 – *Trombo intraventricular em paciente em ECMO VA. Corte apical. Em: http://abccardiol.org/supplementary-material/2026/12304/PosicionamentoPOCUS/Video 59 ECMO VA trombose intraventricular 4c.mp4*

Suplemento 2

Posicionamento POCUS – VIDEOS Reunidos

SESSÃO 8 – POCUS na emergência

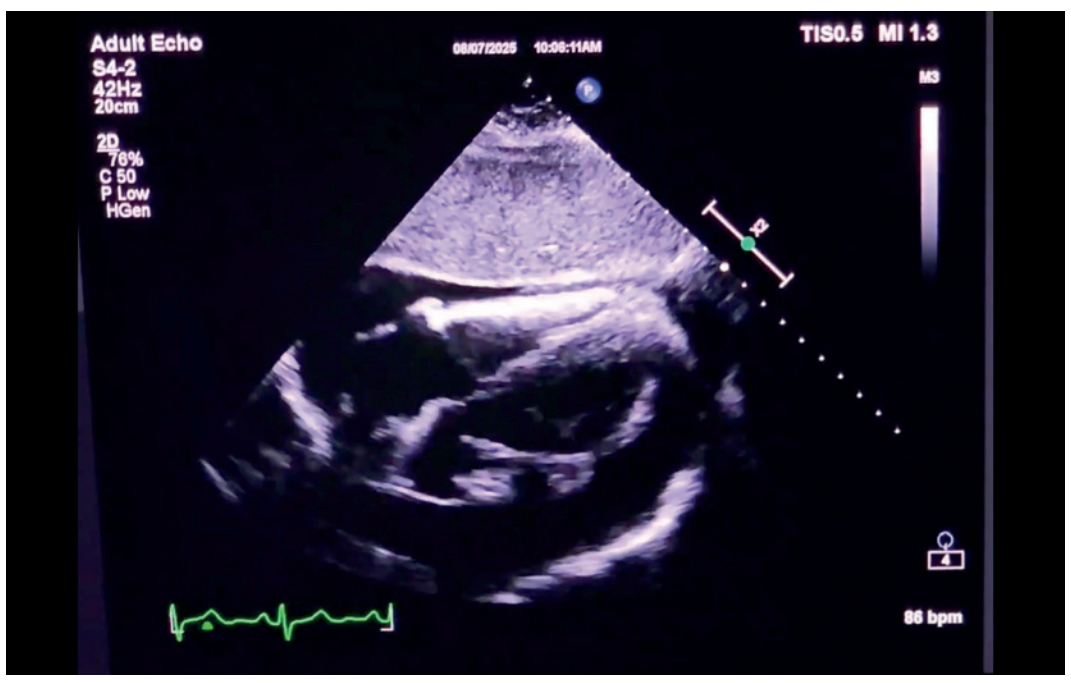

Vídeo 60 – *Corte subcostal 4 câmaras – Derrame pericárdico importante com colabamento diastólico do ventrículo direito. Em: http://abccardiol.org/supplementary-material/2026/12304/PosicionamentoPOCUS/Video 60 Tamponamento colabamento VD e AD.mp4*

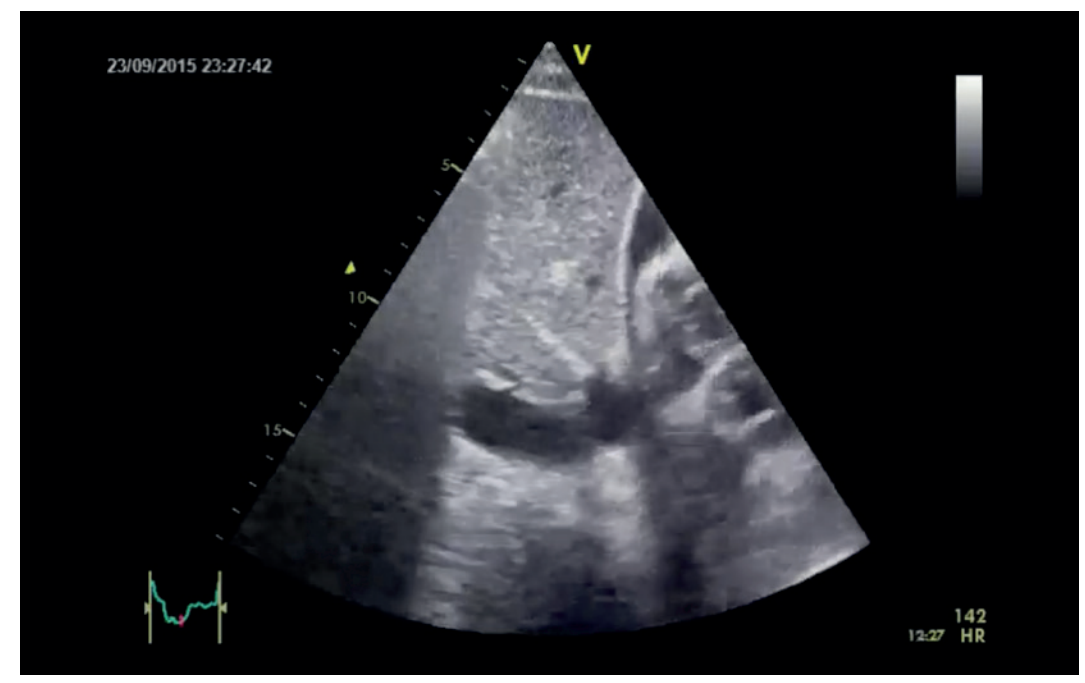

Vídeo 61 – *Corte subcostal da VCI– Derrame pericárdico importante gerando distensão da veia cava inferior. Em: http://abccardiol.org/supplementary-material/2026/12304/PosicionamentoPOCUS/Video 61 Tamponamento cardiaco dilatação de VCI.mp4*

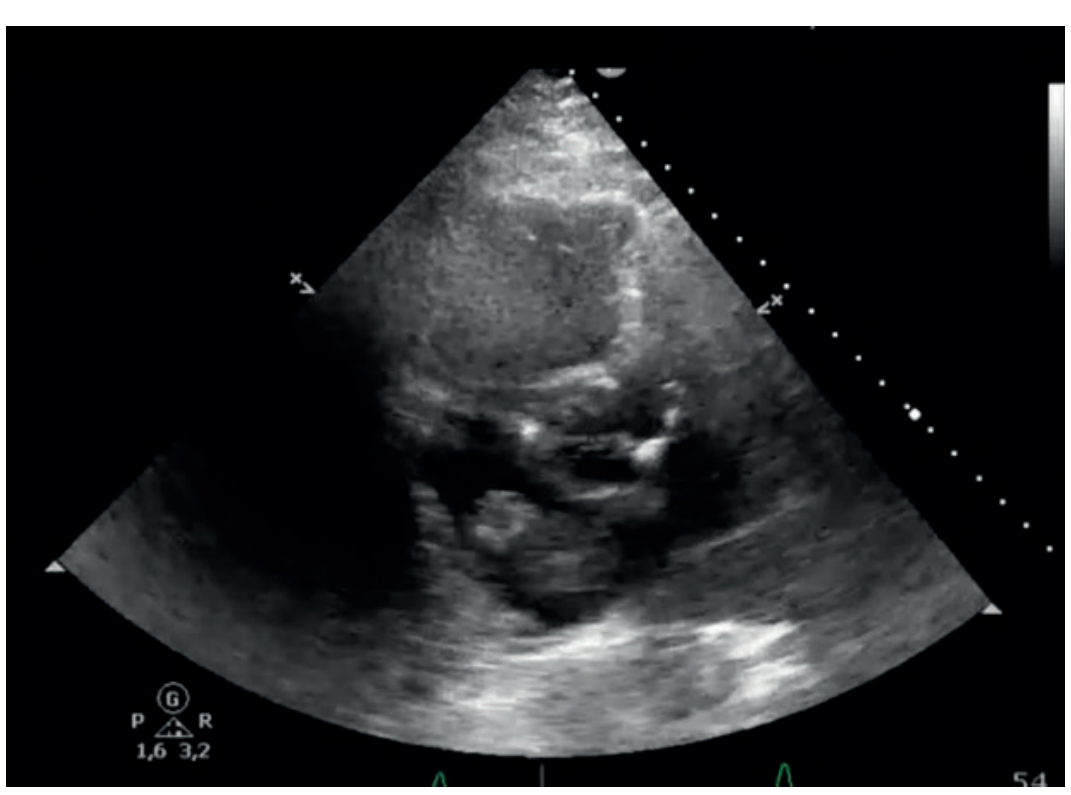

Vídeo 62 – *Corte apical com trombo em cavidades direitas em paciente com TEP submaciço. Em: http://abccardiol.org/supplementary-material/2026/12304/PosicionamentoPOCUS/Video 62 Trombo em transito A4C.mp4*

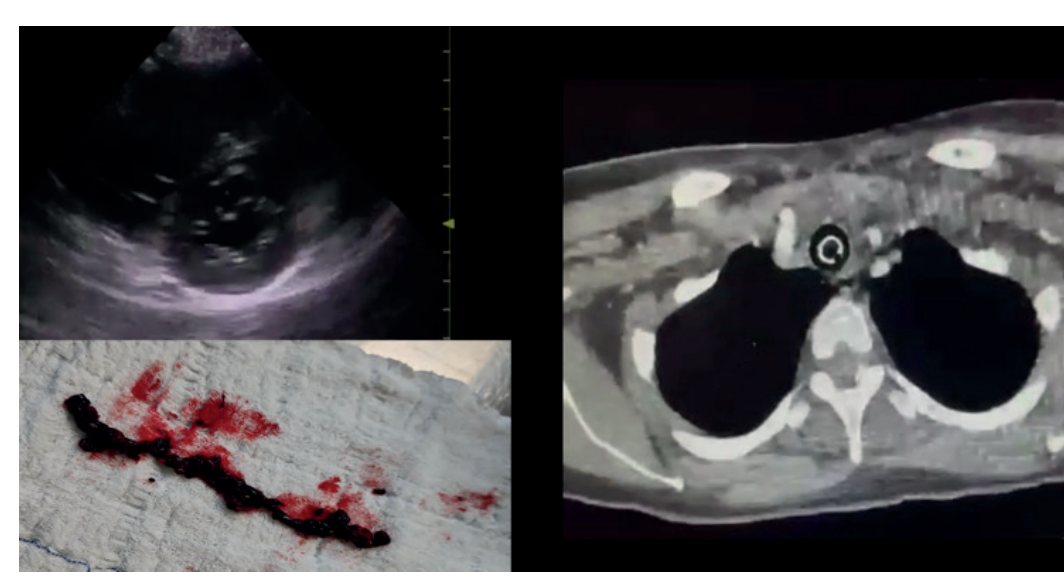

Vídeo 63 – *Pct admitida em choque na emergência eco mostra sobrecarga aguda do VD. Evolui com PCR revertida, angioTC mostra TEP maciço. Submetida a trombectomia mecânica. Em: http://abccardiol.org/supplementary-material/2026/12304/PosicionamentoPOCUS/Video 63 TEP maciço evoluindo com PCR revertida.mp4*

Suplemento 2

Posicionamento POCUS – VIDEOS Reunidos

SESSÃO 9 – POCUS na PCR

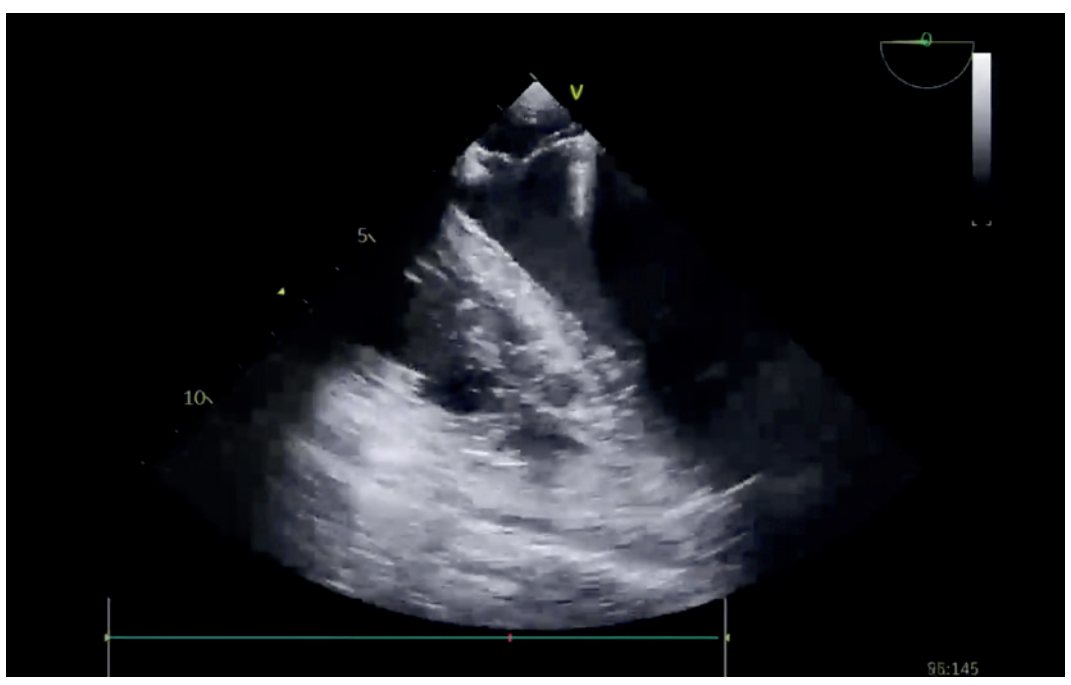

Vídeo 64 – *Corte transesofágico ao nível do esófago médio 4 câmaras – Paciente com prótese mitral biológica sendo submetido à reanimação cardiopulmonar. Em: http://abccardiol.org/supplementary-material/2026/12304/PosicionamentoPOCUS/Video 64 Eco transesofágico e massagem cardiaca.mp4*

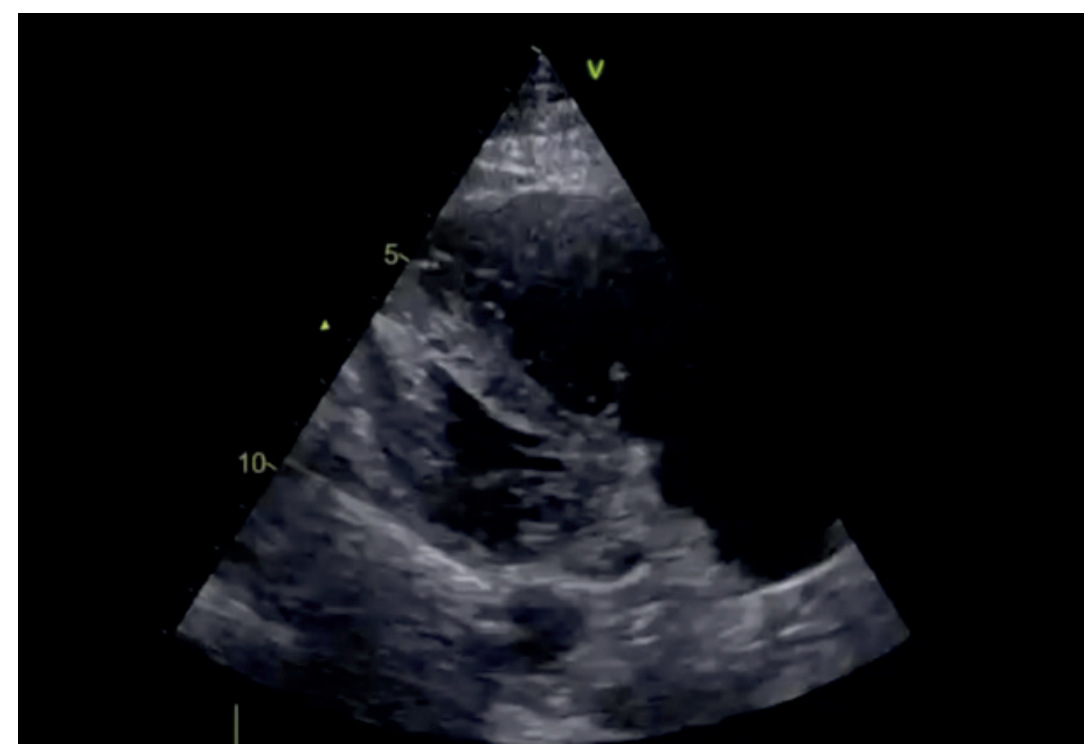

Vídeo 65 – *Corte paraesternal eixo longo – TEP maciço gerando sobrecarga aguda do VD em paciente evoluindo para PCR. Em: http://abccardiol.org/supplementary-material/2026/12304/PosicionamentoPOCUS/Video 65 TEP macico evoluindo com PCR2.mp4*

Suplemento 2

Posicionamento POCUS – VIDEOS Reunidos

SESSÃO 10 – Procedimentos Guiados pelo US

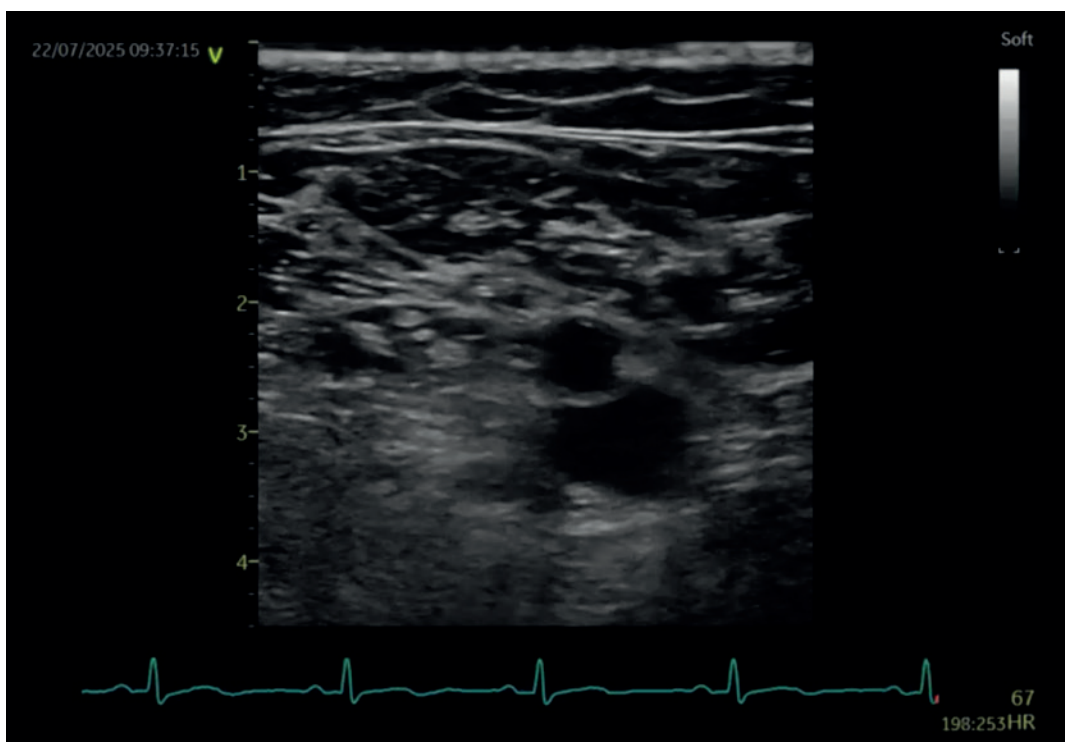

Vídeo 66 – *Acesso guiado por ultrassom da veia femoral pela técnica transversal. Em: http://abccardiol.org/supplementary-material/2026/12304/PosicionamentoPOCUS/Video 66 Punçao venosa ecoguida eixo curto.mp4*

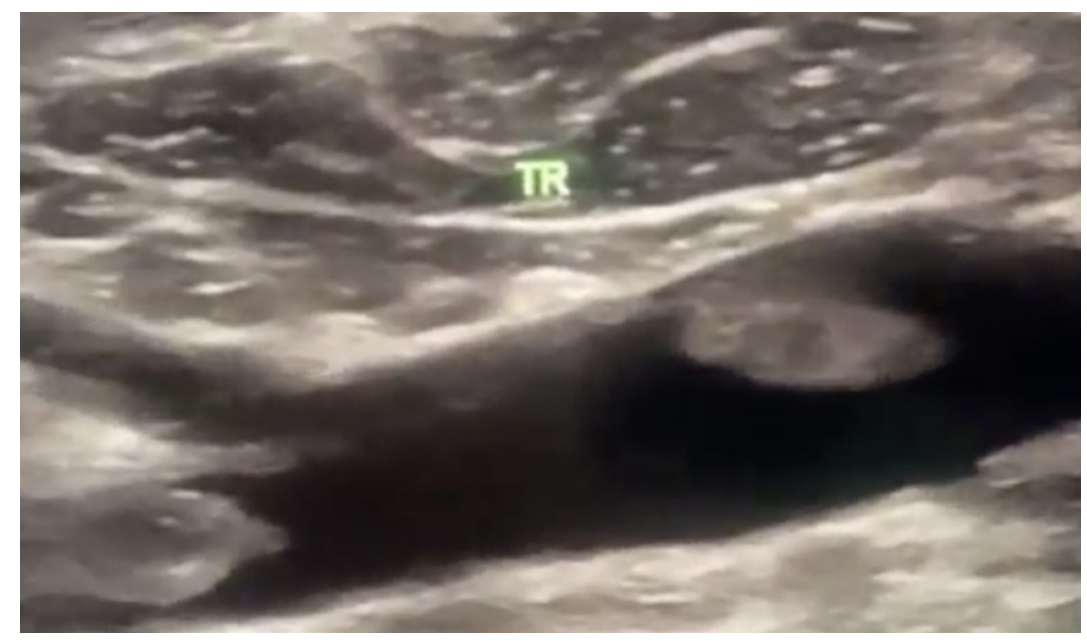

Vídeo 67 – *Trombose de veia subclávia com componente apresentando alta mobilidade relacionado a cateter prévio nesta região. Em: http://abccardiol.org/supplementary-material/2026/12304/PosicionamentoPOCUS/Video 67 Trombo móvel relacionado a cateter.mp4*

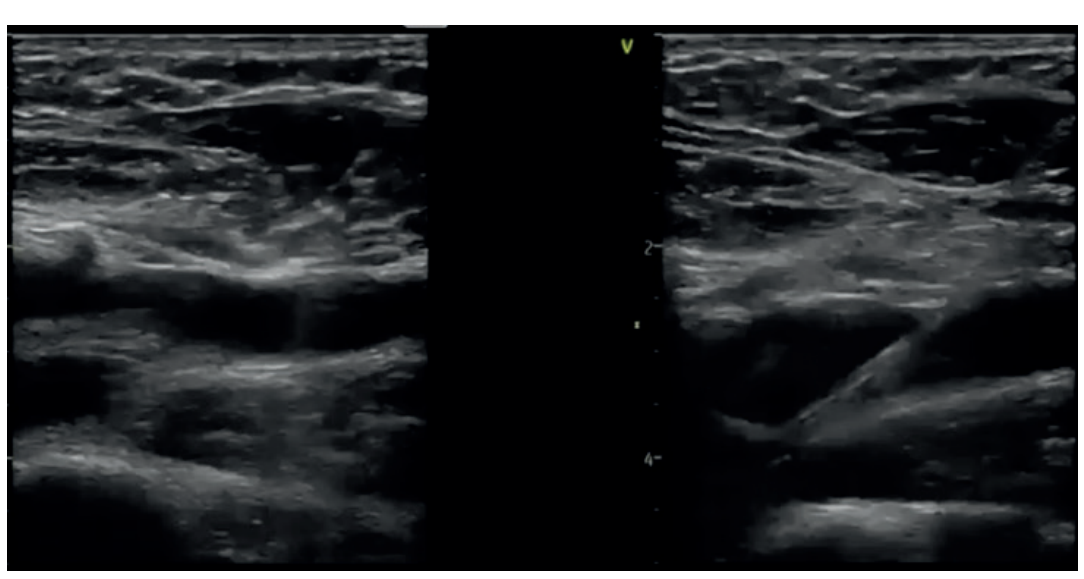

Vídeo 68 – *Visualiza-se o trajeto da agulha em toda a sua extensão inclusive no ponto exato de entrada no vaso. 2º clipe mostra a passagem do fio guia. Em: http://abccardiol.org/supplementary-material/2026/12304/PosicionamentoPOCUS/Video 68 Punção guiada longitudinal.mp4*
