# Supplementary material for: Posicionamento sobre o Uso do Ultrassom "Point of Care" (POCUS) na Medicina Cardiovascular – 2026
Source: Arq Bras Cardiol. 2026 May 4;123(4):e20260222. [Article in Portuguese] doi: 10.36660/abc.20260222 (PMC13671151; doi:10.36660/abc.20260222)
Supplement: Translation

# Translation: Positioning Statement on the Use of Point-of-Care Ultrasound in Cardiovascular Medicine – 2026

**DOI:** 10.36660/abc.20260222i

**Authors:** Monica Luiza de Alcantara, Marcos Paulo Lacerda Bernardo, Adenalva Lima de Souza Beck, Simone Nascimento Santos, Samira Saady Morhy, Edgar Bezerra de Lira, Alex dos Santos Felix, Daniel Goldwasser, Maria Estefânia Bosco Otto, Angelo Antunes Salgado, Ana Paula dos Reis Velloso Siciliano, Fernando Melo, Claudia Maria Vilas Freire, Ana Cristina Lopes Albricker, Rafael Modesto Fernandes, Frederico José Neves Mancuso, Dalton de Souza Barros, Hatem Soliman Aboumarie, Marcelo Haertel Miglioranza, Salomon Israel do Amaral, Marcelo Dantas Tavares de Melo, Marcio Mendes Pereira, Antonio Tito Paladin, Lidia Ana Zytynski Moura, Pedro Gabriel Melo de Barros e Silva, Alexandre de Matos Soeiro, Carlos Eduardo Lucena Montenegro, Luiz Cláudio Danzmann, João Ricardo Cordeiro Fernandes, Silvio Henrique Barberato

**Affiliations:** 1 Rio de Janeiro RJ Brazil Hospital Quinta D’Or RDSL, Rio de Janeiro, RJ – Brazil; 2 Rio de Janeiro RJ Brazil Hospital Universitário Pedro Ernesto (HUPE) da Universidade Estadual do Rio de Janeiro, Rio de Janeiro, RJ – Brazil; 3 Rio de Janeiro RJ Brazil Hospital Copa Star, Rio de Janeiro, RJ – Brazil; 4 Brasília DF Brazil Instituto de Cardiologia e Transplantes do Distrito Federal, Brasília, DF – Brazil; 5 Brasília DF Brazil Hospital Sirio Libanês, Brasília, DF – Brazil; 6 Brasília DF Brazil Eccos Diagnóstico CardioVascular Avançado, Brasília, DF – Brazil; 7 São Paulo SP Brazil Einstein Hospital Istaelita, São Paulo, SP – Brazil; 8 Rio de Janeiro RJ Brazil Instituto Nacional de Cardiologia, Rio de Janeiro, RJ – Brazil; 9 Rio de Janeiro RJ Brazil Complexo Hospitalar Americas, Rio de Janeiro, RJ – Brazil; 10 Rio de Janeiro RJ Brazil Hospital Copa D’Or, Rio de Janeiro, RJ – Brazil; 11 Brasília DF Brazil Universidade de Brasília, Brasília, DF – Brazil; 12 Rio de Janeiro RJ Brazil Rede D’Or, Rio de Janeiro, RJ – Brazil; 13 Brasília DF Brazil Hospital DF Star - Rede D’Or, Brasília, DF – Brazil; 14 Belo Horizonte MG Brazil Empresa Brasileira de Serviços Hospitalares (EBSERH), Belo Horizonte, MG – Brazil; 15 Belo Horizonte MG Brazil Hospital das Clínicas da Universidade Federal de Minas Gerais (HC-UFMC), Belo Horizonte, MG – Brazil; 16 Belo Horizonte MG Brazil Centro Universitário UniBh-Inspirali, Belo Horizonte, MG – Brazil; 17 Salvador BA Brazil Hospital Aliança Rede D’Or, Salvador, BA – Brazil; 18 São Paulo SP Brazil Universidade Federal de São Paulo - Escola Paulista de Medicina (UNIFESP/EPM), São Paulo, SP – Brazil; 19 Salvador BA Brazil Hospital Cardio Pulmonar, Salvador, BA – Brazil; 20 Londres UK Harefield Hospital, Royal Brompton and Harefield Hospitals, Londres, UK – England; 21 Porto Alegre RS Brazil EcoHaertel - Hospital Mae de Deus, Porto Alegre, RS – Brazil; 22 Rio de Janeiro RJ Brazil Hospital Samaritano Botafogo, Rio de Janeiro, RJ – Brazil; 23 João Pessoa PB Brazil Universidade Federal da Paraíba (UFPB), João Pessoa, PB – Brazil; 24 São Luís MA Brazil UDI Hospital Rede D'or São Luiz, São Luís, MA – Brazil; 25 São Paulo SP Brazil Instituto Dante Pazzanese de Cardiologia, São Paulo, SP – Brazil; 26 Curitiba PR Brazil Pontifícia Universidade Católica do Paraná (PUC-PR), Curitiba, PR – Brazil; 27 São Paulo SP Brazil Hospital do Coração, São Paulo, SP – Brazil; 28 São Paulo SP Brazil Brazilian Clinical Research Institute, São Paulo, SP – Brazil; 29 São Paulo SP Brazil Centro Universitário São Camilo, São Paulo, SP – Brazil; 30 São Paulo SP Brazil Instituto do Coração do Hospital das Clínicas da Faculdade de Medicina da Universidade de São Paulo (InCor-HCFMUSP), São Paulo, SP – Brazil; 31 Recife PE Brazil Hospital do Coração de Pernambuco (PROCAPE), Recife, PE – Brazil; 32 Recife PE Brazil Universidade de Pernambuco, Recife, PE – Brazil; 33 Recife PE Brazil Brasil Hospital Esperança Recife - Rede D'or, Recife, PE – Brazil; 34 Porto Alegre RS Brazil Universidade Luterana do Brasil, Porto Alegre, RS – Brazil; 35 Porto Alegre RS Brazil Santa Casa de Porto Alegre, Porto Alegre, RS – Brazil; 36 Curitiba PR Brazil Cardioeco - Centro de Diagnóstico Cardiovascular, Curitiba, PR – Brazil

**Table:** 

Positioning Statement on the Use of Point-of-Care Ultrasound in Cardiovascular Medicine – 2026	
The report below lists declarations of interest as reported to the SBC by the experts during the period of the development of these statement, 2025/2026.	
Expert	Type of relationship with industry
Adenalva Lima de Souza Beck	Nothing to be declared
Alex dos Santos Felix	Nothing to be declared
Alexandre de Matos Soeiro	Financial declaration B - Research funding under your direct/personal responsibility (directed to the department or institution) from the brazilian or international pharmaceutical, orthosis, prosthesis, equipment and implants industry: - Biomerieux.
Ana Cristina Lopes Albriker	Nothing to be declared
Ana Paula dos Reis Veloso Siciliano	Nothing to be declared
Angelo Salgado	Nothing to be declared
Antonio Tito Paladino Filho	Nothing to be declared
Carlos Eduardo Lucena Montenegro	Financial declaration A - Economically relevant payments of any kind made to (i) you, (ii) your spouse/partner or any other person living with you, (iii) any legal person in which any of these is either a direct or indirect controlling owner, business partner, shareholder or participant; any payments received for lectures, lessons, training instruction, compensation, fees paid for participation in advisory boards, investigative boards or other committees, etc. from the brazilian or international pharmaceutical, orthosis, prosthesis, equipment and implants industry: - Bayer: Firialta (HF); Pfizer: Vyndaquel/Vynkella (Amyloidosis); Alnylan: Amvuttra (Amyloidosis) Servier: Vastarel (CAD); Novartis: Entresto, Sybrava (HF, DLP); AstraZeneca: Forxiga, Lokelma, Breztri (HF, CKD, COPD); Viatris: Inspra (HF); Merck: Concor HF, CAD); Novo Nordisk: Wegovy (Obesity, CAD); EMS: Vynaxa (CAD). Other relationships Funding of continuing medical education activities, including travel, accommodation and registration in conferences and courses, from the brazilian or international pharmaceutical, orthosis, prosthesis, equipment and implants industry: - Novo Nordisk: Wegovy (Obesity, HF).
Claudia Maria Vilas Freire	Nothing to be declared
Dalton de Souza Barros	Nothing to be declared
Daniel Goldwasser	Nothing to be declared
Edgar Bezerra de Lira Filho	Nothing to be declared
Fernando Mello Netto	Financial declaration A - Economically relevant payments of any kind made to (i) you, (ii) your spouse/partner or any other person living with you, (iii) any legal person in which any of these is either a direct or indirect controlling owner, business partner, shareholder or participant; any payments received for lectures, lessons, training instruction, compensation, fees paid for participation in advisory boards, investigative boards or other committees, etc. from the brazilian or international pharmaceutical, orthosis, prosthesis, equipment and implants industry: - St. Jude – Abbott: MitraClip (TEER); Edwards Lifesciences: PASCAL (TEER); Abbott: Amplatzer (PFO occluder, ASD occluder); Amulet (left atrial appendage occlusion – LAAO).
Frederico José Neves Mancuso	Nothing to be declared
Hatem Soliman Aboumarie	Nothing to be declared
João Ricardo Cordeiro Fernandes	Nothing to be declared
Lidia Ana Zytynski Moura	Financial declaration A - Economically relevant payments of any kind made to (i) you, (ii) your spouse/partner or any other person living with you, (iii) any legal person in which any of these is either a direct or indirect controlling owner, business partner, shareholder or participant; any payments received for lectures, lessons, training instruction, compensation, fees paid for participation in advisory boards, investigative boards or other committees, etc. from the brazilian or international pharmaceutical, orthosis, prosthesis, equipment and implants industry: - Bayer, Merck, Novartis, Novo Nordisk, Lilly, Viatris. Other relationships Funding of continuing medical education activities, including travel, accommodation and registration in conferences and courses, from the brazilian or international pharmaceutical, orthosis, prosthesis, equipment and implants industry: - Novo Nordisk, Astra.
Luiz Claudio Danzmann	Financial declaration A - Economically relevant payments of any kind made to (i) you, (ii) your spouse/partner or any other person living with you, (iii) any legal person in which any of these is either a direct or indirect controlling owner, business partner, shareholder or participant; any payments received for lectures, lessons, training instruction, compensation, fees paid for participation in advisory boards, investigative boards or other committees, etc. from the brazilian or international pharmaceutical, orthosis, prosthesis, equipment and implants industry: - AstraZeneca: Lokelma; Bristol Myers Squibb: Camzyos; Bayer: Firialta. Other relationships Funding of continuing medical education activities, including travel, accommodation and registration in conferences and courses, from the brazilian or international pharmaceutical, orthosis, prosthesis, equipment and implants industry: - AstraZeneca: Lokelma. Any other interest - financial or other - that should be declared considering the position taken in SBC that has not been expressly listed above: - Health area; medical education.
Marcelo Dantas Tavares de Melo	Nothing to be declared
Marcelo Haertel Miglioranza	Nothing to be declared
Marcio Mendes Pereira	Financial declaration A - Economically relevant payments of any kind made to (i) you, (ii) your spouse/partner or any other person living with you, (iii) any legal person in which any of these is either a direct or indirect controlling owner, business partner, shareholder or participant; any payments received for lectures, lessons, training instruction, compensation, fees paid for participation in advisory boards, investigative boards or other committees, etc. from the brazilian or international pharmaceutical, orthosis, prosthesis, equipment and implants industry: - Abbott: MitraClip. Other relationships Funding of continuing medical education activities, including travel, accommodation and registration in conferences and courses, from the brazilian or international pharmaceutical, orthosis, prosthesis, equipment and implants industry: - Abbott: MitraClip.
Marcos Paulo Lacerda Bernardo	Nothing to be declared
Maria Estefânia Bosco Otto	Nothing to be declared
Monica Luiza de Alcantara	Financial declaration A - Economically relevant payments of any kind made to (i) you, (ii) your spouse/partner or any other person living with you, (iii) any legal person in which any of these is either a direct or indirect controlling owner, business partner, shareholder or participant; any payments received for lectures, lessons, training instruction, compensation, fees paid for participation in advisory boards, investigative boards or other committees, etc. from the brazilian or international pharmaceutical, orthosis, prosthesis, equipment and implants industry: - Boston Medical: proctoria Watchman; Edwards Lifesciences: proctoria PASCAL; Vitória Hospitalar: proctoria MitraClip (Abbott), Amplatzer (Abbott)
Pedro Gabriel Melo de Barros e Silva	Financial declaration B - Funding for research under your direct/personal responsibility (directed to the department or institution) from the pharmaceutical, orthosis, prosthetic, equipment, and implant industries, Brazilian or foreign: - Bayer, Novartis, Idorsia, Roche Diagnostics
Rafael Modesto Fernandes	Other relationships Funding of continuing medical education activities, including travel, accommodation and registration in conferences and courses, from the brazilian or international pharmaceutical, orthosis, prosthesis, equipment and implants industry: - Edwards.
Salomon Israel do Amaral	Nothing to be declared
Samira Saady Morhy	Nothing to be declared
Silvio Henrique Barberato	Financial declaration A - Economically relevant payments of any kind made to (i) you, (ii) your spouse/partner or any other person living with you, (iii) any legal person in which any of these is either a direct or indirect controlling owner, business partner, shareholder or participant; any payments received for lectures, lessons, training instruction, compensation, fees paid for participation in advisory boards, investigative boards or other committees, etc. from the brazilian or international pharmaceutical, orthosis, prosthesis, equipment and implants industry: - Pfizer: Amyloidosis; AstraZeneca: HFpEF. Other relationships Funding of continuing medical education activities, including travel, accommodation and registration in conferences and courses, from the brazilian or international pharmaceutical, orthosis, prosthesis, equipment and implants industry: - Pfizer: Amyloidosis.
Simone Nascimento Santos	Nothing to be declared

## Table of contents

**1. POCUS: Methodology, Concept, Training, and Certification** 8
  **1.1. Methodology** 8
  **1.2. Concept and Scope of Application** 8
  **1.3. Equipment** 8
  **1.4. Training, Certification, and Maintenance of Competence** 9
  **1.5. Ethical and Legal Aspects of Cardiovascular POCUS use in Brazil** 9
**2. Cardiac POCUS** 12
  **2.1. Main Windows in Cardiac POCUS** 12
  **2.2. Assessment of Global Left Ventricular Systolic Function** 13
    **2.2.1. General Considerations** 13
    **2.2.2. Methods for Assessing Left Ventricular Systolic Function** 14
    **2.2.3. Other Techniques for Assessing Left Ventricular Systolic Function** 14
  **2.3. Assessment of Right Ventricular Overload and Right Ventricular Systolic Function** 14
    **2.3.1. Pulmonary Embolism** 14
    **2.3.2. POCUS for Assessing Right Ventricular Myocardial Infarction** 15
    **2.3.3. Other Applications** 16
    **2.3.4. Limitations** 16
  **2.4. POCUS in Cardiac Tamponade** 16
  **2.5. Advanced POCUS** 16
    **2.5.1. Assessment of Left Ventricular Outflow Tract Velocity-Time Integral and Determination of Stroke Volume and Cardiac Output** 16
    **2.5.2. E/e′ ratio as a measure of left atrial pressure and fluid tolerance** 18
  **2.6. Pediatric cardiac POCUS** 19
**3. Lung Ultrasound** 20
  **3.1. Introduction** 20
  **3.2. Technical Aspects** 20
    **3.2.1. Equipment and Transducers** 20
  **3.3. Examination Protocols** 20
  **3.4. Quantification of B-Lines** 21
  **3.5. Main Applications of Lung Ultrasound** 21
    **3.5.1. Pulmonary Congestion** 21
    **3.5.2. Pleural Effusion** 21
    **3.5.3. Interstitial Syndromes** 21
    **3.5.4. Pneumonic Consolidation** 23
    **3.5.5. Atelectasis** 23
    **3.5.6. Pneumothorax** 24
    **3.5.7. Pulmonary Embolism** 24
  **3.6. Conclusions and Future Perspectives** 24
**4. Ultrasound Focused on the Assessment of Systemic Venous Congestion - VExUS** 24
  **4.1. Introduction** 24
  **4.2. Clinical Studies and Scientific Evidence** 24
  **4.3. Venous Excess Ultrasound Components** 25
  **4.4. Venous Excess Ultrasound Grading** 28
  **4.5. Clinical Applications** 28
  **4.6. Limitations and Pitfalls** 28
  **4.7. Conclusion** 29
**5. POCUS in Deep Vein Thrombosis** 29
  **5.1. Introduction** 29
  **5.2. Scientific Evidence for POCUS in Assessment of Deep Vein Thrombosis** 30
  **5.3. Technique of the Focused Ultrasound Protocol for Assessment of Deep Vein Thrombosis** 30
**6. POCUS in the Patient with Circulatory Shock** 31
  **6.1. Introduction** 31
  **6.2. Diagnostic Protocols** 31
  **6.3. Key Questions to Identify the Type of Shock** 34
  **6.4. Fluid Responsiveness and Fluid Tolerance** 36
    **6.4.1. Estimation of Cardiac Output** 36
    **6.4.2. Inferior and Superior Vena Cava** 36
    **6.4.3. Passive Leg Raising Maneuver** 36
    **6.4.4. Expiratory and Inspiratory Occlusion Maneuvers** 36
**7. POCUS in Short-Term Mechanical Circulatory Support** 37
  **7.1. Introduction** 37
  **7.2. The Role of POCUS in Short-Term Mechanical Circulatory Support** 37
  **7.3. Initial Patient Assessment** 37
  **7.4. Device Placement and Position Confirmation** 39
  **7.5. Intra-Aortic Balloon Pump** 39
    **7.5.1. Introduction** 39
    **7.5.2. Transesophageal Echocardiography Examination Sequence to Guide Intra-Aortic Balloon Pump Insertion** 39
    **7.5.3. Post-Implantation Checklist** 39
  **7.6. Veno-Arterial Extracorporeal Membrane Oxygenation** 39
    **7.6.1. Introduction** 39
    **7.6.2. Patient Selection for Extracorporeal Membrane Oxygenation** 40
    **7.6.3. Cannulation Guidance** 41
    **7.6.4. Immediate Effects of Peripheral Venoarterial Extracorporea Membrane Oxygenation Observed by Bedside Echocardiography** 41
    **7.6.5. Assessment of Unloading and Weaning** 43
    **7.6.6. Monitoring During Weaning Trials** 43
  **7.7. Challenges and Future Directions** 44
  **7.8. Conclusion** 44
**8. POCUS in the Emergency Department** 44
  **8.1. POCUS in Chest Pain** 44
    **8.1.1. Evaluation of Pericardial Diseases in the Emergency Department** 44
    **8.1.2. Pleural and Pulmonary Evaluation in the Emergency Department** 44
  **8.2. Evaluation of the Patient with Dyspnea** 45
    **8.2.1 Pulmonary Congestion, Pleural Diseases, and Other Diagnoses Assessed by Lung Ultrasound** 45
    **8.2.2. Pulmonary Embolism** 46
  **8.3. Evaluation of the Patient with Hemodynamic Instability or Organ Dysfunction** 46
  **8.4. Evaluation of the Patient with Peripheral Edema** 47
**9. POCUS in Cardiac Arrest** 47
  **9.1. Introduction** 47
  **9.2. Technical Aspects** 48
  **9.3. Performance of POCUS in Cardiac Arrest** 48
  **9.4. Confirmation of Cardiac Arrest, Assessment of Chest Compression Effectiveness, and Prognostic Role of Cardiac Activity in Pulseless Electrical Activity** 48
  **9.5. Diagnosis of Reversible Causes Using POCUS in Cardiac Arrest** 48
    **9.5.1. Cardiac Tamponade** 48
    **9.5.2. Pulmonary Embolism** 48
    **9.5.3. Tension Pneumothorax** 50
    **9.5.4. Hypovolemia** 50
**10. Ultrasound-Guided Procedures** 50
  **10.1. Ultrasound-Guided Vascular Access** 50
    **10.1.1. Cannulation Technique** 50
    **10.1.2. Step-by-Step Cannulation (Use Sterile Supplies)** 50
    **10.1.3. Transverse (Short-Axis) Cannulation** 51
    **10.1.4. Longitudinal (Long-Axis) Cannulation** 51
    **10.1.5. Ultrasound-Guided Cannulation Sites** 51
    **10.1.6. Other Ultrasound-Guided Vascular Punctures** 52
  **10.2. Ultrasound-Guided Pericardiocentesis** 53
    **10.2.1. Ultrasound-Guided Puncture Technique** 53
    **10.2.2. Contraindications** 53
    **10.2.3. Complications** 53
  **10.3. Evaluation of Gastric Fullness** 53
    **10.3.1. Assessment Technique** 53
    **10.3.2. Sonographic Findings** 54
**11. Final Considerations of this Positioning Statement** 54
  **11.1. Current Reality and Future Perspectives** 54
  **11.2. Contextualization of Cardiovascular Point-of-Care Ultrasound within Public Health in Brazil** 54
**References** 55
**Suplement 1** 62
**Suplement 2** 63

## 1. POCUS: Methodology, Concept, Training, and Certification

### 1.1. Methodology

This positioning statement was developed through online meetings involving a multidisciplinary group of specialists. Each participant prepared their respective section based on clinical experience and a review of relevant research. Collective discussions enabled the consolidation of the recommendations presented in this document.

### 1.2. Concept and Scope of Application

The term point-of-care ultrasound (POCUS) refers to the use of ultrasound as an adjunct to the physical examination, focused on answering a specific, urgent clinical question. In this positioning statement, POCUS is applied to the anatomical and functional assessment of heart, lungs, and blood vessels at the bedside, supporting diagnosis and therapeutic decision-making in hospitalized and outpatient settings, ranging from neonates to adults.

The clinical relevance of POCUS lies in its portability, relatively low cost, and accessibility. It can be performed by frontline physicians, including cardiologists and non-cardiologists, in emergency departments, intensive care units (ICUs), operating rooms, and in remote or resource-limited environments where conventional imaging examinations are not readily available. However, several prerequisites must be fulfilled before a POCUS is adopted in clinical practice (Figure 1).

**Figure 1:** Prerequisites for POCUS implementation in a health care service. *Cardiac abnormalities identified in this age group should raise suspicion of congenital heart disease, and a comprehensive examination by a pediatric echocardiographer should be performed as soon as possible. DCI: Department of Cardiovascular Imaging; POCUS: point-of-care ultrasound; SBC: Brazilian Society of Cardiology.

**Figure:** PE: Pulmonary embolism; POCUS: Point-of-care ultrasound; TEE: Transesophageal echocardiography; TTE: Transthoracic echocardiography; US: Ultrasound; VA-ECMO: Venoarterial extracorporeal membrane oxygenation; VExUS: Venous Excess Ultrasound Score; VV-ECMO: Venovenous extracorporeal membrane oxygenation.

### 1.3. Equipment

Although POCUS can be performed using different ultrasound systems, the ideal equipment should be capable of acquiring one- and two-dimensional images, spectral Doppler, and color flow mapping. It should include transducers with variable frequencies suitable for adult and pediatric cardiac imaging as well as for assessment of lungs and vessels. Image settings should be standardized or adjustable according to the structure being examined (presets), and the system should allow image storage in a format compatible with Digital Imaging and Communications in Medicine (DICOM) standards. Equipment miniaturization, with devices featuring screens as small as those of smartphones, along with the incorporation of artificial intelligence (AI) tools, such as transducer positioning guidance, has played a significant role in the expanding use of POCUS.

### 1.4. Training, Certification, and Maintenance of Competence

Training methodologies for POCUS are highly variable. However, the primary objectives of training, according to the desired level of competence, are summarized in Table 1,^1–8^ and an acronym is proposed in Figure 2.^1,2^ The required training duration and number of supervised examinations may vary depending on prior experience, the targeted level of proficiency, and supervisor assessment.

**Table 1:** Main objectives of POCUS training for image acquisition, interpretation, and documentation of results according to the desired level (1, 2, and 3)

Skills	Level 1	Level 2	Level 3
**Acquisition**			
**1.** Ability to identify the main cardiac structures on ultrasound (pericardium, cardiac chambers, valves, aorta, and inferior vena cava).			
**2.** Understanding basic ultrasound concepts, including physical principles, image generation, and equipment adjustment (gain, focus, and depth), with the aim of improving image resolution when changing acoustic windows.	**(1)**		
**3.** Acquire the cardiac POCUS windows (parasternal long-axis and short-axis, apical four-chamber, subcostal, and inferior vena cava in longitudinal and transverse views); the lung windows (anterior and posterior chest); the abdominal aorta in transverse view along its entire course; the femoral and popliteal veins; and the visceral veins (hepatic, portal, and renal veins).	**(2)**		
**Interpretation**			
**4.** Supervised practice under the guidance of a trained professional, with subsequent review.			
**5.** Ability to recognize the presence or absence of pathology according to the clinical suspicion, in order to answer basic clinical questions, including chamber size, wall thickening, left ventricular dysfunction, right ventricular dysfunction, pericardial effusion, hypovolemia, pulmonary congestion (B-lines), systemic venous congestion, deep vein thrombosis, pneumothorax, or pleural effusion.	**(3)**	**(5)**	**(6)**
**6.** Knowledge of cardiovascular POCUS application protocols for the differential diagnosis of shock, respiratory failure, and cardiac arrest (SHOCK, RUSH, EGLS).			
**7.** Assessment of fluid responsiveness and systemic congestion.			
**8.** Ability to perform a basic evaluation of cardiac valves, cardiac output, and filling pressures.			
**9.** In neonates, identification of patent foramen ovale, patent ductus arteriosus, and estimation of pulmonary systolic pressure.			
**10.** Use of POCUS for ultrasound-guided vascular access, pericardial drainage, orotracheal intubation, assessment of bladder volume, and gastric residual volume. Recognition of POCUS limitations, associated risks, and required precautions.			
**Outcomes**			
**11.** Ability to store images on the ultrasound system and document ultrasound findings in the medical record as well as to integrate ultrasound findings with the patient's clinical presentation.	**(4)**		
**12.** Development of the ability to critically interpret ultrasound findings in the context of emergency clinical scenarios.			
**13.** Documentation of clinical decision-making in direct patient care based on integrated analysis.			
**14.** Storage of images on the ultrasound system and transmission in DICOM format to the hospital PACS.			
**15.** Ability to recognize whether the pathology suggested by POCUS is compatible with the clinical scenario and, in case of uncertainty, promptly indicate a comprehensive diagnostic examination. Likewise, to pursue further investigation when clinical suspicion persists despite the absence of POCUS findings.			

(1) Learning limited to appropriate patient positioning, transducer selection, preset selection, and correct transducer orientation.

(2) Limited to the subcostal window and at least one additional cardiac window, longitudinal inferior vena cava view, anterior pleural windows, transverse aortic view up to the proximal abdominal segment, femoral and popliteal veins, and FAST examination.

(3) Limited to recognizing the presence or absence of ultrasonographic signs of cardiac abnormalities (visual assessment of chamber size, B-lines, pleural effusion, and pericardial effusion).

(4) Limited to reporting ultrasound findings in conjunction with the physical examination.

(5) Except for assessment of visceral venous congestion, all evaluations performed qualitatively as YES or NO.

(6) With semiquantitative assessment categorized as mild, moderate, or severe, when applicable.

DICOM: Digital Imaging and Communications in Medicine; FAST: Focused Assessment with Sonography for Trauma; PACS: Picture Archiving and Communication System; POCUS: point-of-care ultrasound.

**Figure 2:** Stages of POCUS training. The online test is not exclusionary but should be a mandatory prerequisite for participation in the next stage, as it allows the teaching plan to be tailored to the trainee's needs.^1^ At least 25% of the case mix should consist of pathological cases, which may be supplemented with videos.^2^ See Figures 2 and 3.^3^ Ideally, though not mandatory, POCUS images should be uploaded in DICOM format to the hospital imaging system, with an average of 10 dynamic clips.^4^ These videos should be made available on the institution's website, accompanied by multiple-choice questions, answer keys, and comments; access to educational videos from specialized societies is also recommended.^5^

Figure 3 presents a suggested training protocol that should ideally be tested, validated, and endorsed by relevant specialty societies. The training stages proposed by this group are described in Figure 2 and include, at the conclusion of training, an internal assessment and certification of competence (Supplement 1). To minimize risks and overcome the challenges associated with POCUS performance (Figure 4), attention must also be given to the legal considerations and safeguards outlined below (Figure 1).

**Figure 3:** Level-based POCUS training protocol, including the minimum recommended number of examinations and training duration for each level. For example, Level 1 is expected to acquire skills in image AcQUIsition, Interpretation, and documentation of REsults (AcQUIRE), corresponding to items 1, 4, 5, and 11 described in Table 1. N: minimum number of examinations; ΔT: training weeks.

**Figure 4:** Risks and challenges involved in POCUS. The scope of POCUS extends beyond cardiac assessment and may require collaboration with other specialists, in addition to echocardiographers, such as vascular specialists, radiologists, intensivists, and pediatric cardiologists.^1^ At present, no reimbursement mechanisms for POCUS have been established, and dedicated funding from training centers remains unavailable.^2^

### 1.5. Ethical and Legal Aspects of Cardiovascular POCUS Use in Brazil

Cardiovascular POCUS should be understood as a medical act performed at the bedside with the purpose of answering specific clinical questions and supporting immediate decision-making. It does not replace a comprehensive diagnostic echocardiographic examination when such an examination is indicated.^9,10^ Within this context, the physician performing POCUS assumes direct civil liability for the acts undertaken, including test indication, appropriate technical execution, accurate interpretation of findings, and proper documentation in the medical record, in accordance with the principles established by the Brazilian Code of Medical Ethics.^9,11^ Professional accountability arises not only from potential technical errors but also from the use of POCUS outside the practitioner's scope of competence, without adequate training, or in disagreement with institutional protocols.^11,12^

In Brazil, formal training in POCUS remains limited within undergraduate medical curricula and residency programs. As a result, professional qualification often depends on extracurricular courses, which leads to heterogeneity in training quality.^13,14^ The implementation of structured cardiovascular POCUS programs requires investments in equipment, digital infrastructure, supervised training, and clinical governance mechanisms.^15^ Despite initial costs, national studies have demonstrated a positive impact on diagnostic efficiency and more rational use of complementary examinations, suggesting favorable cost-effectiveness, particularly in emergency care, intensive care settings, and regions with limited access to conventional echocardiography.^13,15^ In this context, integration with telehealth strategies strengthens Tele-POCUS as a tool to expand access to specialized cardiovascular assessment within the Brazilian Unified Health System (SUS), promoting greater equity in health care delivery.^16^

Nevertheless, significant structural challenges remain. These include the absence of standardized coding and clear reimbursement policies for cardiovascular POCUS in both the public health care system and the supplementary private sector, which compromises the financial sustainability of institutional programs.^17,18^ Additionally, specific attention must be given to professional liability insurance coverage since the lack of formal regulation defining scope of practice, minimum training requirements, and certification criteria. This regulatory gap contributes to legal uncertainty, particularly in increasingly judicialized health care environments.^11,12^

Although health care institutions may establish internal policies for training, supervision, and documentation, regulation on the use of POCUS by the Brazilian Federal Council of Medicine, in collaboration with medical specialty societies, is a priority. Such regulation should clearly define scope of practice, minimum training and certification requirements, and differentiation from comprehensive diagnostic echocardiographic examinations.^10,11^ This regulatory framework is essential to ensure legal security for professionals, patient safety, standardization of clinical practice, and the responsible and sustainable incorporation of cardiovascular POCUS into the Brazilian health care system.

## 2. Cardiac POCUS

### 2.1. Main Windows in Cardiac POCUS

Unlike conventional echocardiography, cardiac POCUS relies on images that are generally easy to obtain and interpret. It is used as an extension of the physical examination to support diagnosis and clinical decision-making in several emergency situations, such as the evaluation of circulatory shock and volume management in critically ill patients.^21^

Five main echocardiographic windows are used in cardiac POCUS (Figure 5), as follows:^22^

**Figure 5:** Main windows used in cardiac POCUS, with corresponding transducer positioning and assessed structures.

1. Parasternal long-axis window (long-axis view of the heart), obtained with the transducer positioned along the left parasternal line, between the 3rd and 4th intercostal spaces, with the transducer marker oriented toward the patient's right shoulder;
2. Parasternal short-axis window (short-axis view of the heart), obtained at the same location as the parasternal long-axis window, but with a 90° rotation of the transducer, such that the marker points toward the patient's left shoulder. With slight angulation of the transducer, the two-dimensional imaging plane can be modified, allowing "tomographic" views at the level of the great vessels, papillary muscles, and apical segments of the left ventricle (LV);
3. Apical 4-chamber window (A4C): obtained with the transducer positioned along the left midclavicular line, between the 4th and 5th intercostal spaces, below the nipple or inframammary line, at the level of the apical impulse (point of maximal impulse), with the transducer marker oriented toward the patient's left side;
4. Subcostal window, obtained with the transducer positioned on the midline, just below the xiphoid process, forming an acute angle with the abdominal wall, and with the transducer marker oriented toward the patient's left side;
5. Subcostal window focused on the inferior vena cava (IVC), obtained at the same location as the subcostal window, but with the transducer positioned perpendicular to the abdominal wall and the marker oriented toward the patient's head.

### 2.2. Assessment of Global Left Ventricular Systolic Function

#### 2.2.1. General Considerations

LV ejection fraction (LVEF) is the most validated and most commonly used measure for the assessment of systolic function.^23^ LV dysfunction may cause or significantly complicate acute clinical conditions. In undifferentiated shock, focused assessment of LV function has been shown to improve diagnostic accuracy and guide treatment decisions when a qualitative approach is used.^24^

In this document, a simplified and objective assessment is recommended since it has a direct impact on clinical decision-making.

#### 2.2.2. Methods for Assessing Left Ventricular Systolic Function

Assessment of LV function should be performed using all cardiac windows available in the individual patient, as described in the section on cardiac POCUS windows.

Traditional methods for determining LV function, including biplane Simpson's method and the Teichholz method, are not commonly used in cardiac POCUS. These techniques require additional training, are time-consuming, are prone to measurement errors, and do not necessarily provide greater accuracy in this setting. Therefore, cardiac POCUS has adopted a qualitative approach.

Unlüer et al.,^25^ as well as other authors, have demonstrated good correlation between visual assessment performed by emergency physicians and examinations conducted by specialists.

In this qualitative assessment, attention should be given to endocardial excursion (inward movement of the ventricular walls toward the center of the LV) and myocardial thickening (increase in wall thickness during contraction). These parameters allow classification of LV systolic function into four broad categories: i) normal, ii) hyperdynamic, iii) reduced systolic function, and iv) severe dysfunction (Table 2). The category of reduced systolic function includes patients with mild to moderate dysfunction on formal echocardiography.

**Table 2:** Qualitative assessment of LV systolic function

**Normal**	Myocardial thickening of most LV segments, with endocardial displacement toward the center of the LV.
**Hyperdynamic**	Increased endocardial excursion and myocardial thickening, resulting in partial or complete systolic obliteration of the LV cavity.
**Reduced systolic function**	Reduced endocardial excursion and myocardial thickening.
**Severe dysfunction**	Severely reduced endocardial excursion and myocardial thickening.

See examples in Videos 1 to 13 in Supplement 2.

This classification is intended to be intuitive and clinically relevant in patient care.^26^

#### 2.2.3. Other Techniques for Assessing Left Ventricular Systolic Function

Other semiquantitative methods, such as mitral annular plane systolic excursion (MAPSE) and E-point septal separation, may be used; however, each has inherent limitations.^27,28^ For more detailed information on these parameters, the relevant bibliographic references should be consulted.

Recent studies have demonstrated good correlation between automated ejection fraction measurements obtained using AI and conventional assessments, provided that image acquisition quality is adequate. This approach represents a promising tool in this field.^29^

### 2.3. Assessment of Right Ventricular Overload and Right Ventricular Systolic Function

Findings from cardiac POCUS may assist in the rapid diagnosis of pathological conditions that primarily or secondarily affect the right ventricle (RV), providing valuable information for the early initiation of appropriate hemodynamic support and/or specific therapies.

In the evaluation of patients presenting with chest pain and/or acute dyspnea, particularly when accompanied by signs of hemodynamic instability or heart failure (HF), it is essential to exclude potential differential diagnoses such as hypovolemia, severe LV systolic dysfunction, cardiac tamponade, acute valvular dysfunction, or aortic dissection.^30^

In unstable patients with acute RV overload and/or dysfunction, the following diagnoses should be considered: i) acute pulmonary embolism (PE); ii) acute RV acute myocardial infarction (AMI); or iii) RV dysfunction in the context of acute HF due to various etiologies, including AMI, myocarditis, sepsis, peripartum cardiomyopathy, acute valvular heart disease, and stress-induced cardiomyopathy (Takotsubo syndrome).

RV enlargement may be identified in the parasternal long-axis view (PLAX; Figure 6A) or in the A4C view (Figure 6B). In the apical view, the RV area is normally ≤ two-thirds of the LV area; a ratio exceeding 1.0 is suggestive of significant RV enlargement and overload.^31^

**Figure 6:** Assessment of RV overload using the RV/LV ratio (A and B); assessment of RV systolic function using lateral tricuspid annular plane systolic excursion measured in M-mode (C and D). LV: left ventricle; RV: right ventricle.

Assessment of RV systolic function is inherently complex, limiting the accuracy of subjective analysis due to the RV's unique crescent-shaped geometry, with inflow and outflow tracts located in different planes as well as the significant influence of preload, afterload, and ventricular interdependence.^32^ In addition to subjective evaluation, we recommend the use of simple objective measures to assess RV systolic function, such as tricuspid annular plane systolic excursion (TAPSE), measured at the lateral tricuspid annulus (RV free wall). TAPSE is reduced in patients with acute pulmonary embolism and hemodynamic overload and has prognostic value.^33^ TAPSE is obtained from the A4C view and is considered abnormal when <1.7 cm (Figures 6C and 6D).

#### 2.3.1. Pulmonary Embolism

The use of cardiac POCUS in suspected acute pulmonary embolism provides substantial added value due to the rapid acquisition of clinically relevant diagnostic and prognostic information. This is particularly important in hemodynamically unstable patients who cannot be easily transported for computed tomography pulmonary angiography (CTPA) or who cannot await a comprehensive echocardiographic examination. Echocardiographic findings suggestive of pulmonary embolism are typically present only when there are signs of acute RV overload, which usually occurs in hemodynamically significant pulmonary embolism (massive or submassive). In this context, cardiac POCUS is useful for risk stratification and for guiding therapeutic strategy.^34^ Findings such as RV dilation with septal flattening (the "D-shaped" septum) (Figures 7A and 7B) and, in some cases, regional RV wall motion abnormalities characterized by preserved apical contractility with hypokinesia of the mid and basal segments (McConnell sign) may support the diagnosis. Although classically described, the McConnell sign is present in only 12%-20% of patients.^35,36^ In patients with acute pulmonary embolism, systemic venous congestion due to RV failure is also common, frequently manifested by a dilated IVC with reduced respiratory variation, reflecting elevated estimated right atrial pressure.

**Figure 7:** Right ventricular dilation with septal flattening (D-shaped septum).

Approximately 5% of patients admitted with cardiac arrest in the emergency department may have acute pulmonary embolism as the underlying cause. Rapid diagnosis may improve prognosis by enabling earlier thrombolytic therapy or percutaneous pulmonary thromboendarterectomy.^37^

In acute pulmonary embolism, intracardiac thrombi within the right-sided chambers^10^ or saddle thrombi at the pulmonary artery bifurcation are rarely visualized (< 5%). When present, these findings not only confirm the diagnosis but are also associated with poor prognosis, particularly in patients who develop RV systolic dysfunction.^38–40^

Echocardiography has a reported sensitivity of approximately 60%-70%; therefore, a negative examination does not exclude the diagnosis of pulmonary embolism.^41^ The combined use of cardiac POCUS, lower-extremity venous ultrasound, and lung ultrasound (LUS), referred to as "triple POCUS," increases diagnostic accuracy for pulmonary embolism detection.

In a multicenter study by Nazerian et al.^42^ involving 357 patients with clinical suspicion of pulmonary embolism, the diagnostic accuracy of triple POCUS was compared with CTPA findings. Triple POCUS demonstrated a sensitivity of 90% and a specificity of 86%, markedly higher than the use of individual modalities alone (LUS: sensitivity 61%, specificity 96%; cardiac POCUS: sensitivity 33%, specificity 91%; lower-extremity venous ultrasound: sensitivity 53%, specificity 98%).

In hemodynamically unstable patients, the use of cardiac POCUS combined with lower-extremity venous ultrasound, even before CTPA, is capable of reducing diagnostic time and improving therapeutic outcomes.^43,44^ The European Society of Cardiology recommends that in patients with a high clinical suspicion of acute pulmonary embolism and echocardiographic evidence suggestive of the diagnosis, immediate thrombolytic therapy may be justified when additional diagnostic tests are not readily available or when delays may increase patient risk.^41^

#### 2.3.2. POCUS for Assessing Right Ventricular Myocardial Infarction

RV AMI may occur in up to 30%-50% of patients with inferior AMI and can complicate hemodynamic management, potentially leading to bradycardia, need for pacemaker implantation, hypotension, and death. The presence of segmental wall motion abnormalities in the inferior and inferolateral walls, along with a characteristic pattern of RV segmental dysfunction, supports this diagnostic possibility.^45^

Important differential diagnoses include i) stress-induced cardiomyopathy (Takotsubo syndrome), which may also cause RV segmental dysfunction, typically presenting as apical akinesia accompanied by a similar pattern of apical ballooning in the LV; and ii) segmental contractile abnormalities known as the McConnell sign, characterized by preserved apical contractility with mid and basal dysfunction, as described in acute pulmonary embolism.

#### 2.3.3. Other Applications

The use of cardiac POCUS may be helpful in critically ill patients as a tool for serial assessment of RV systolic function and to facilitate hemodynamic management. Examples include i) patients undergoing pulmonary recruitment maneuvers as part of mechanical ventilation strategies, in whom ventriculo-arterial coupling may be affected^46^; ii) patients with RV failure, for serial evaluation of chamber dimensions and cardiac output; and iii) monitoring of patients receiving extracorporeal membrane oxygenation (ECMO) support who present with signs of instability, allowing assessment of chamber decompression, cannula positioning, and potential obstruction.^47^

#### 2.3.4. Limitations

The use of cardiac POCUS for assessing RV dimensions and systolic function has important limitations. Obtaining an adequate acoustic window can be challenging and is highly dependent on examiner expertise. In addition, the RV is extremely sensitive to changes in preload and afterload as well as to ventricular interdependence with LV function. These factors must be considered in the context of serial and longitudinal assessments.

### 2.4. POCUS in Cardiac Tamponade

Although uncommon, cardiac tamponade, a condition in which the accumulation of fluid within the pericardial space compresses the heart and impairs adequate filling, constitutes a medical emergency.^48^ In advanced stages, restriction of diastolic filling leads to a marked reduction in cardiac output and systemic and coronary perfusion, ultimately resulting in abrupt hypotension and bradycardia.^49^

Because the classic clinical signs of cardiac tamponade, known as Beck's triad (arterial hypotension, muffled heart sounds, and jugular venous distension), are not always present or may appear only in advanced stages, POCUS becomes a valuable tool for early detection.^50^

Quantification of pericardial fluid volume based on the distance between the parietal and visceral pericardium at end-diastole (Table 3), although easily performed using parasternal and subcostal windows, does not allow definitive diagnosis of cardiac tamponade. Other factors, such as the rate of fluid accumulation and the nature of the fluid (transudate, exudate, or blood), may result in a rapid increase in intrapericardial pressure even with relatively small effusion volumes.^49^

**Table 3:** Semiquantitative assessment of pericardial effusion according to end-diastolic measurement and estimated fluid volume

End-diastolic measurement	Estimated fluid volume
5-10 mm	100-250 mL (small)
10-20 mm	250-500 mL (moderate)
>20 mm	> 500 mL (large)
>25 mm	Very large

Adapted from Klein et al.^49^

Distension of the IVC and collapse of the right-sided cardiac chambers in the presence of pericardial effusion are key findings for confirming the physiology of cardiac tamponade (Figure 8) when there is clinical suspicion, although their sensitivity and specificity vary considerably.^50^ IVC and hepatic vein dilation, as well as right atrial collapse during ventricular systole, are findings with high sensitivity, whereas diastolic right ventricular collapse is the most specific finding of diastolic restriction on POCUS.

**Figure 8:** Key POCUS findings in cardiac tamponade. *Inferior vena cava dilation may be absent in cardiac tamponade associated with marked hypovolemia. Other findings, such as leftward interventricular septal shift during inspiration (part of the physiology of pulsus paradoxus), may also be observed.^49^

Differentiation between pericardial effusion and pleural effusion may be challenging and requires careful attention during POCUS examination. In the PLAX view, effusions located anterior to the descending aorta are typically pericardial, whereas those located posterior to the vessel are of pleural origin (Figure 9).

**Figure 9:** Differentiation between pleural effusion (posterior to the aorta) and pericardial effusion (anterior to the aorta).

### 2.5. Advanced POCUS

The concept of advanced POCUS encompasses progressive skills acquired throughout POCUS training, involving the assessment of hemodynamic parameters obtained by ultrasound. Each of such skills has a learning curve, which should be followed to ensure the highest possible accuracy in clinical application. In this section, we address the assessment of LV outflow tract velocity-time integral (LVOT VTI), determination of stroke volume and cardiac output, as well as evaluation of the E/e′ ratio and its role in determining fluid tolerance (FT).

#### 2.5.1. Assessment of Left Ventricular Outflow Tract Velocity-Time Integral and Determination of Stroke Volume and Cardiac Output

Stroke volume is calculated as the product of the LVOT VTI and the LVOT cross-sectional area. The LVOT area is derived from its diameter, which is measured in the PLAX view, as illustrated in Figure 10. The LVOT VTI is typically obtained from the apical five-chamber view. After acquiring the A4C view, slight anterior angulation of the transducer is required to obtain this window.

**Figure 10:** Calculation of stroke volume by multiplying the area of the left ventricular outflow tract obtained from the parasternal window by the left ventricular outflow tract flow integral obtained from the apical five-chamber view.^51^ A: area; D: diameter; SV: stroke volume; VTI: velocity time integral.

For this measurement, pulsed-wave Doppler is used, with the sample volume positioned within the LVOT. After spectral Doppler acquisition, the flow envelope is traced, yielding a value expressed in centimeters. When the LVOT is considered a cylindrical structure, the VTI corresponds to the distance traveled by blood from the LVOT into the aorta during each cardiac cycle (Figure 10).

The use of LVOT area has potential drawbacks. Because this calculation is derived from the formula D^2^ × 0.785, where D represents the LVOT diameter, any measurement error is squared, resulting in a significant error in the calculated stroke volume. Unfortunately, measurement errors at this step are not uncommon. For these reasons, an alternative to stroke volume measurement is required in emergency settings.

Since the LVOT diameter (and consequently the LVOT area) is essentially constant, any change in stroke volume reflects a change in LVOT VTI. Therefore, LVOT VTI represents a potential surrogate for stroke volume and an important hemodynamic parameter for both initial assessment and serial monitoring during treatment, allowing evaluation of therapeutic responses and guidance of clinical management.

Cardiac output is calculated by multiplying stroke volume by the patient's heart rate.^52^

In healthy individuals, LVOT VTI typically ranges from 18 to 22 cm at a heart rate between 55 and 95 beats per minute (bpm). When heart rate is below 55 bpm, the expected LVOT VTI should be greater than 18 cm. Similarly, in patients with heart rates exceeding 95 bpm, LVOT VTI should be less than 22 cm; otherwise, a high–cardiac output state should be considered. The concept of "trend tracking" underlies functional hemodynamic monitoring, in which changes in cardiac function in response to therapy are more informative than isolated static measurements.^53^ The application of LVOT VTI within a diagnostic and therapeutic algorithm will be addressed in the section on POCUS in the patient with shock.

#### 2.5.2. E/e′ Ratio as a Measure of Left Atrial Pressure and Fluid Tolerance

An increase in left atrial pressure (LAP) has important consequences for gas exchange, pulmonary hemodynamic afterload, and right ventricular performance.^54^

Increased LAP may result from pre-existing conditions such as LV systolic and diastolic dysfunction as well as mitral and aortic valve disease. However, acute increases in LAP may also be observed in critically ill patients with sepsis, myocardial ischemia, stress-induced cardiomyopathy, and volume overload states.^55^

The relationship between the transmitral E-wave and the e′ wave obtained at the medial and lateral mitral annulus (mean E/e′ ratio) has been extensively studied in cardiology populations and has gained increasing interest in the critical care literature. The E/e′ ratio is less load dependent and may be used to assess elevated LAP across various clinical scenarios (Figure 11). Although a normal E/e′ ratio does not exclude elevated LAP, a mean E/e′ value > 14 has high specificity for increased LAP. This finding may be particularly useful in pragmatic bedside decision-making when further fluid resuscitation is being considered: in this context, an E/e′ > 14 strongly supports the presence of "fluid intolerance." At the other end of the spectrum, an E/e′ < 8 has good accuracy for predicting LAP < 18 mm Hg.^56^

**Figure 11:** Calculation of the E/e′ ratio derived from the relationship between the transmitral E-wave and the average e′ wave obtained by tissue PWD imaging at the medial and lateral mitral annulus. PWD: pulsed-wave Doppler.

### 2.6. Pediatric Cardiac POCUS

As defined for adult patients, cardiac POCUS in the pediatric population (≤ 18 years) is an extension of the physical examination, performed at the bedside to rapidly support clinical decision-making and patient management. The main indications are summarized in Chart 1.

**Chart 1:** Main indications for pediatric POCUS

**1.** Assessment of ventricular systolic function
**2.** Assessment of pulmonary hypertension
**3.** Assessment of pericardial effusion and guidance for pericardiocentesis
**4.** Assessment of volume status and fluid responsiveness
**5.** Assessment of patent ductus arteriosus in neonates

Cardiac POCUS does not include detailed assessment of cardiac anatomy nor the diagnosis of congenital heart disease. It is also distinct from so-called targeted neonatal echocardiography, which is an examination that uses multiple echocardiographic parameters to assess systolic and diastolic function and intravascular volume status.^4,57^

## 3. Lung Ultrasound

### 3.1. Introduction

LUS, which was described later than other POCUS applications,^58^ has become essential for rapid, bedside, real-time assessment of patients presenting with dyspnea. When combined with clinical history and physical examination, LUS can accelerate accurate diagnosis and appropriate treatment.^59,60^ The presence of extravascular lung water in pulmonary congestion is readily detected by LUS, with diagnostic performance comparable to B-type natriuretic peptide (BNP) measurement and superior to chest radiography.^61,62^ Other conditions associated with pulmonary involvement, such as pleural effusion, interstitial syndromes, pneumonia, atelectasis, pneumothorax, and pulmonary embolism, can also be assessed with LUS and are addressed below.^63^

LUS is based on pleural artifacts, as the pleura is easily visualized. Under normal conditions, the aerated lung reflects ultrasound energy, resulting in reverberation artifacts of the pleural line projected at regular intervals in the ultrasound field, known as A-lines. Thickening of the interalveolar spaces or alveolar filling by fluid or inflammatory processes causes progressive blurring of A-lines and the appearance of B-lines, which are vertical artifacts that move synchronously with pleural sliding and propagate throughout the ultrasound field.^64^

### 3.2. Technical Aspects

A comprehensive description of technical aspects of POCUS has been provided earlier in this document. However, some specific considerations related to LUS deserve emphasis.

#### 3.2.1. Equipment and Transducers

Basic ultrasound systems, including handheld devices, are capable of obtaining adequate LUS images. When a lung preset is not available, the abdominal preset is considered the most appropriate due to its higher persistence. However, the adult cardiac preset, despite lower persistence, may also be used without loss of diagnostic information.^65^ Four types of transducers may be used for LUS: a 5-MHz convex transducer, a 3-5-MHz phased-array transducer, a 5-8-MHz microconvex transducer, and a 5-12-MHz linear transducer. The latter is particularly useful for detailed assessment of the pleural line and pleural sliding.^65^

#### 3.3. Examination Protocols

Among the various examination protocols described in the literature, we recommend three protocols (Figure 12), which may be selected according to the clinical scenario. The most commonly used protocol is the Bedside Lung Ultrasound in Emergency (BLUE) protocol (Figure 12A). BLUE divides each hemithorax into three quadrants: two anterior quadrants, focused on the detection of B-lines and pneumothorax, and a third posterolateral basal quadrant, aimed at identifying pleural effusion and pulmonary consolidations.^66^

**Figure 12:** Lung ultrasound protocols recommended in this position statement. (A) BLUE protocol (ref. 56); (B) 8-quadrant protocol (ref. 60); (C) 12-quadrant protocol (ref. 62). For detailed descriptions of each protocol, please refer to the cited references. BLUE: Bedside Lung Ultrasound in Emergency.

With imaging depth set at approximately 15-18 cm, the transducer should be positioned sagittally with the marker oriented cranially. With the patient in the supine position, careful scanning of each quadrant should be performed throughout the full respiratory cycle, identifying the point with the highest concentration of B-lines. The anterior chest quadrants are referred to as the upper and lower BLUE points and, for identification purposes, may be labeled cranio-caudally as R1 and R2 for the right hemithorax and L1 and L2 for the left hemithorax. At these points, the objective is to identify a B-profile indicative of pulmonary congestion, with a reported sensitivity of 97% and specificity of 95%.^67^ A B-profile is defined by the presence of three or more B-lines per anterior quadrant in each hemithorax, with preserved pleural sliding.^68,69^

The third quadrant, known as the postero-lateral alveolar and/or pleural syndrome (PLAPS) point, may be difficult to obtain in patients unable to assume a semilateral position. Optimal positioning allows visualization of the costophrenic recess, diaphragm, and liver or spleen. In this quadrant, the primary goal is the assessment of pleural effusion and possible pneumonic consolidations (Figure 13F).

**Figure 13:** Lung ultrasound findings (detailed descriptions in Table 4): (a) A-lines; (b) B-lines; (c) coalescent B-lines with spared areas; (d) coalescent B-lines; (e) subpleural consolidation; (f) translobar consolidation; (g) compressive atelectasis; (h) shred sign; (i) plankton sign; (j) septated effusion; (k) triangular subpleural consolidation on linear probe (pulmonary embolism); (l) barcode sign (left lung pneumothorax).

Although the BLUE protocol is widely used in POCUS, it is qualitative in nature and limited to the anterior chest. More recently, aiming for a quantitative approach and longitudinal follow-up, particularly in the context of HF with reduced ejection fraction (HFrEF) or HF with preserved ejection fraction (HFpEF), an 8-quadrant protocol has been recommended (Figure 12B), without compromising examination time or diagnostic accuracy,^70^ without compromising examination time or diagnostic accuracy.^71^ For conditions involving posterior lung regions, such as interstitial syndromes, including SARS-CoV-2 pneumonia, protocols incorporating posterior lung zones may be used (Figure 12C).^72^

### 3.4. Quantification of B-Lines

There is no consensus regarding the optimal method for B-line quantification. A growing trend involves automated, real-time counting of B-lines using dedicated software.^73,74^

Currently, two main approaches with satisfactory reproducibility are commonly used:

- Binary scoring by zone, in which the presence of three or more B-lines defines a positive zone;^70^
- Total B-line count, performed either by counting individual B-lines or by estimating the percentage of the screen occupied by B-lines and dividing by 10. This method is particularly useful for coalescent B-lines (for example, if 80% of the screen is occupied by B-lines, this corresponds to 8 B-lines) (Figure 13B);^71^
- Although not yet validated in the literature, a third approach is suggested to reduce intra- and interobserver variability without increasing examination time. For longitudinal follow-up, B-lines may be categorized into ranges (e.g., 0, < 3, 3-5, 5-7, and ≥ 8 B-lines), assigning a score from 0 to 4 to each range for every quadrant examined.^54^ The scores from all quadrants are then summed to obtain a total score according to the number of quadrants assessed (e.g., a score of 20 out of a maximum of 32 in an 8-quadrant protocol). This method provides a semiquantitative assessment of B-lines that can be compared over the course of decongestive therapy.

### 3.5. Main Applications of Lung Ultrasound

For better understanding throughout the text, Table 4 summarizes the terminology most commonly used in LUS, with selected findings illustrated in Figure 13.

**Table 4:** Description of the most frequent lung ultrasound findings and their clinical significance. Letters in bold correspond to Figure 13

Finding	Description	Clinical Significance
Pleural line	Curvilinear hyperechoic line beneath the ribs	Interface between parietal and visceral pleura
Pleural sliding	Movement of the vis-ceral pleura over the parietal pleura during respiration	Excludes pneumothorax and pleural adhesions
A-lines **(a)**	Horizontal reverberation artifacts arising from the pleural line	Aerated lung (normal), COPD, or pneumothorax (when pleural sliding is absent)
B-lines **(b)**	Vertical artifacts arising from the pleural line extending to the bot-tom of the ultrasound field	Accumulation of hydrostatic and/or inflammatory fluid or fibro-sis in interlobular septa/alveoli
Z-lines	Static vertical artifacts that do not reach the bottom of the screen	No pathological significance; should be differentiated from B-lines
Lung point	Transition point between normal pleural sliding and loss of pleural movement	Pathognomonic for pneumothorax; the more lateral the lung point, the larger the pneumothorax
Lung pulse	Rhythmic pleural movement synchronized with cardiac activity	Excludes pneumothorax in the presence of absent pleural sliding
Barcode sign (strato-sphere sign)	Horizontal lines throughout the ultra-sound field on M-mode	Suggestive of pneumothorax
Plankton sign	Mobile punctate ech-oes within pleural fluid	May indicate infection, hemothorax, or post-aggressive diuretic therapy
Septated (loculated) effusion	Debris, fibrin strands, and septations	Complex effusion suggesting infection, abscess, empyema, or fibrosis, potentially hindering pleural drainage
Air bronchogram **(f)**	Hyperechoic punctate echoes within bronchi surrounded by consolidation	Dynamic air bronchograms favor pneumonia; static air bronchograms favor atelectasis
Shred sign **(h)**	Irregular hyperechoic deep subpleural line separating consolidated from partially aerated lung	Seen in nontranslobar consolidations, more commonly associated with pneumonia
Curtain sign	Overlapping of aerated lung during respiration obscuring the liver or spleen image	Aerated lung bases at the point of examination
Jellyfish sign **(g)**	Collapsed lung seg-ment with undulating motion within a large pleural effusion	Compressive atelectasis
Subpleural consolidation **(e) (k)**	Small hypoechoic subpleural collection (triangular, circular, or polygonal)	Pulmonary embolism, atelectasis, infection, hemorrhage, or neoplasm
Translobar consolidation **(f)**	Absence of air in the lung, giving it a solid organ-like (hepatization) appearance	Pneumonia or atelectasis

COPD: chronic obstructive pulmonary disease.

#### 3.5.1. Pulmonary congestion

The diagnosis of cardiogenic pulmonary congestion can only be suggested when associated with a clinical picture of HFrEF or HFpEF, in its acute or chronic forms. The chronic form may at times be subclinical due to adaptive mechanisms such as thickening of the alveolar–capillary membrane, increased lymphatic drainage, and pulmonary hypertension.^75^ In the context of decompensated HF, LUS signs of pulmonary congestion often precede clinical findings, such as crackles, a third heart sound, peripheral edema, jugular venous distension, and dyspnea, by several days.^75^ The Brazilian Guideline for Chronic and Acute Heart Failure – 2018 recommends, as a Class I, Level of Evidence C indication, the integrated use of LUS and echocardiography for the assessment of pulmonary congestion in patients admitted with acute HF, adding only a few minutes to the examination time while providing substantial additional diagnostic information.^76^

We recommend using the BLUE protocol in emergencies because of its simplicity and level of validation.^66^ Identification of a B-profile indicates the presence of extravascular lung water. When combined with a diagnosis of HF (HFrEF or HFpEF) and echocardiographic evidence of hemodynamic congestion, it confirms the presence of cardiogenic pulmonary congestion. When the goal is grading and longitudinal follow-up of congestion, the 8-quadrant protocol is preferred.^71^ At the level of the third quadrant (PLAPS point), pleural effusion and possible associated pneumonic consolidation, as potential triggers of decompensation, should be assessed.

#### 3.5.2. Pleural Effusion

LUS has high accuracy for the detection of pleural effusion (93%) compared with chest radiography (47%),^67^ allowing identification of small effusions starting at volumes as low as 20 mL. The optimal technique involves positioning the patient in a semilateral decubitus position, slightly elevated (45 degrees), or seated when thoracentesis or drainage is planned, with insonation of the PLAPS point.

Pleural effusion is present in 56%-90% of cases of decompensated HF and in approximately 25% of cases of isolated right-sided HF. It may persist in up to 60% of patients during the pre-discharge phase^63^ without implying a worse prognosis.^77^ In left-sided HF, pleural effusion develops as a consequence of increased LAP and only after pulmonary congestion has occurred. In right-sided HF, effusion results from transmission of increased right atrial pressure to the thoracic duct, impairing lymphatic drainage from the pleural space to the superior vena cava (SVC),^63^ and is more frequently right-sided when unilateral. Several methods for pleural effusion quantification have been described. The most commonly used is the method proposed by Balik et al.,^78^ in which, during expiration, the maximal distance (in centimeters) between the pleural dome and the lung tissue is multiplied by 200 to estimate effusion volume in milliliters (Figure 14). Based on fluid echogenicity, LUS allows qualitative assessment of the likelihood of transudative, exudative (plankton sign; Figure 13I), or hemorrhagic effusion. The presence of debris and septations indicates a complex effusion of likely infectious origin, warranting further investigation^79^ (Figure 13J).

**Figure 14:** Example of pleural effusion volume estimation obtained by measuring, during expiration, the distance between the diaphragmatic dome and lung tissue.

#### 3.5.3. Interstitial Syndromes

Acute respiratory distress syndrome (ARDS) represents a form of pulmonary edema secondary to inflammatory processes caused by various pulmonary or systemic diseases, most commonly viral or bacterial pneumonia, sepsis, aspiration, and severe trauma, including barotrauma secondary to mechanical ventilation. ARDS may coexist with HF, further complicating LUS interpretation.^80^ Certain LUS findings help differentiate cardiogenic from inflammatory pulmonary edema, including heterogeneous and asymmetric distribution of B-lines, spared areas within the same ultrasound field (Figure 13C), thickening and irregularity of the pleural line, and the presence of subpleural (Figure 13E) or translobar consolidations, all findings observed, for example, in SARS-CoV-2 pneumonia.^72^

Idiopathic pulmonary fibrosis or fibrosis secondary to toxins, organic dusts, medications, or connective tissue diseases is characterized by progressive thickening and fibrosis of lung tissue. In milder forms, pleural line thickening is observed. As the disease progresses, pleural thickening increases, pleural sliding is reduced, B-lines - some of them coalescent - appear, and subpleural cysts develop, which on LUS resemble subpleural consolidations.^81^

#### 3.5.4. Pneumonic Consolidation

Bacterial pneumonia is characterized by loss of lung aeration, with alveoli filled by infectious fluid, giving the lung a solid, tissue-like appearance (hepatization). Consolidation may vary in extent, being classified as translobar when it occupies the entire ultrasound field, or nontranslobar or subpleural when an irregular hyperechoic line divides consolidated from partially aerated lung tissue^82^ (shred sign) (Figure 13H).

Pneumonic consolidations are most commonly located at the lung bases (PLAPS point), often accompanied by small pleural effusions restricted to the costophrenic recess, inflammatory B-lines, and occasional dynamic air bronchograms (Figure 13F). The pleura typically appears thickened and irregular, with reduced or absent pleural sliding.^83^

#### 3.5.5. Atelectasis

Atelectasis refers to loss of lung volume and aeration due either to bronchial obstruction by mucus plugging or to compression of the parenchyma secondary to large pleural effusions. Extensive atelectasis leads to ventilation–perfusion mismatch, intrapulmonary shunt, increased pulmonary vascular resistance, and hypoxemia. Differentiating atelectasis from pneumonic consolidation by LUS is challenging and must be interpreted in the clinical context. Findings such as static air bronchograms and localized rather than diffuse B-lines^84^ aid in this differentiation. In cases of compressive atelectasis, a thin layer of consolidated lung tissue with sinusoidal movement (jellyfish sign) may be observed within pleural fluid (Figure 13G). Notably, atelectasis and pneumonic consolidation may coexist.^85^

#### 3.5.6. Pneumothorax

LUS has a sensitivity of approximately 90% and a specificity of 98% for the diagnosis of pneumothorax.^65^ Air accumulation between the parietal and visceral pleura occurs predominantly in the anterior chest. Therefore, when pneumothorax is clinically suspected, whether due to trauma or positive-pressure mechanical ventilation, evaluation should begin in the anterior thorax. In this region, the most sensitive diagnostic finding is abolition of pleural sliding (lung sliding). On M-mode ultrasonography, this condition appears as the barcode (stratosphere) sign, characterized by multiple horizontal parallel lines extending throughout the ultrasound field. This pattern contrasts with the physiological seashore sign, in which the granular appearance of the lung parenchyma ("sand") is distinguished from the static horizontal lines of the chest wall ("sea") above the pleural line^65^ (Figure 13L).

Absence of pleural sliding, however, is not specific to pneumothorax and may also be observed in selective intubation, pleurodesis, pulmonary contusions, and pulmonary fibrosis.^67^

Although not always easy to demonstrate, the lung point is the only pathognomonic sign of pneumothorax, indicating the exact transition between collapsed lung (absent sliding) and normal lung (present sliding). The more lateral the lung point, the larger the pneumothorax.

#### 3.5.7. Pulmonary Embolism

Although LUS is not considered the first-line diagnostic modality for the investigation of pulmonary embolism,^34^ it represents a valuable alternative for patients who are unable to undergo CTPA. Integrated analysis of LUS with cardiac and venous POCUS, aimed at identifying signs of right-sided chamber overload and/or deep vein thrombosis (DVT), may increase the sensitivity and specificity of the method.^86^ Findings related to pulmonary embolism are most frequently located in the posterobasal regions of the thorax, where patients often report pleuritic chest pain. The most suggestive LUS finding is a triangular subpleural consolidation (Figure 13K), which may also appear circular or polygonal. These consolidations result from parenchymal necrosis or atelectasis due to disruption of the surfactant layer with local blood extravasation.^87^

### 3.6. Conclusions and Future Perspectives

Lung ultrasound (LUS), previously underutilized, has recently become an essential tool for outpatient follow-up and for the management of patients with dyspnea in intensive care units, and emergency departments. LUS is a rapid, simple, and highly informative imaging modality, capable of providing critical diagnostic information. In addition it is cost-effective and suitable for bedside application.

With the advent of AI applied to image interpretation, automated B-line quantification, and the development of integrated diagnostic algorithms, the use of LUS is expected to expand further in emergency medicine, critical care, cardiology, and other specialties in which this form of POCUS imaging adds value to clinical decision-making. Nevertheless, it is important to emphasize that LUS does not replace careful clinical assessment and must always be interpreted within the context of the patient's clinical history and overall presentation.

## 4. Ultrasound Focused on the Assessment Of Systemic Venous Congestion – VExUS

### 4.1. Introduction

The management of critically ill patients has long focused on ensuring adequate perfusion pressure, with attention primarily directed toward the arterial side of the vascular system through assessment of fluid responsiveness (FR). However, the venous system, often overlooked, plays a crucial role in this context. Patients with right ventricular failure, pulmonary hypertension, or fluid overload are prone to developing significant systemic venous congestion, resulting in a reduced arteriovenous pressure gradient, increased capillary hydrostatic pressure, and interstitial edema, frequently exacerbated by endothelial dysfunction.^88,89^ These factors adversely affect tissue perfusion and contribute to organ dysfunction, particularly kidney impairment. Acute kidney injury (AKI), in turn, worsens fluid retention, perpetuating a vicious cycle and significantly increasing morbidity and mortality.^90–93^

In this setting, early detection of venous congestion is of paramount importance for the development of appropriate patient monitoring and management strategies. However, assessment based on physical examination and cumulative fluid balance has important limitations.^94–96^ Moreover, central venous pressure (CVP) measurements are invasive, prone to error, and do not always reliably reflect preload, particularly in patients receiving mechanical ventilation.^97–101^

Because of the scarcity of reliable clinical signs, additional, more precise, and objective information is required to guide therapeutic decision-making. In this context, POCUS has emerged as a useful, effective tool for assessing volume status and quantifying systemic venous congestion.

### 4.2. Clinical Studies and Scientific Evidence

Ultrasonographic markers of systemic venous congestion are not new. More than 20 years ago, portal vein pulsatility was described in the context of HF,^102^ although it was never fully incorporated into routine clinical practice.

More recently, venous excess ultrasound (VExUS) has proven to be a reliable and reproducible method, demonstrating good interobserver agreement among trained operators and correlation with other measures of volume status, such as CVP. The technique has gained increasing attention in the literature, and several studies have explored its application across different clinical contexts (Table 5).

**Table 5:** Studies evaluating the VExUS score in different clinical contexts

Study	Objective	Results
**Beaubien-Souligny et al. (2020)**^103^ Single-center prospective study; 145 patients undergoing cardiac surgery	Initial VExUS grading system and its association with AKI	Association with subse-quent AKI: HR = 3.69; 95% CI 1.65-8.24; p = 0.001; +LR 6.37 (95% CI 2.19-18.50) when detected at ICU admission, outperforming CVP
**Bhardwaj et al. (2020)**^104^ Prospective ICU cohort; 30 patients	Application of VExUS score for AKI staging in cardiorenal syndrome	AKI resolution significantly correlated with improvement in VExUS grade (p = 0.003); significant association between VExUS changes and fluid balance (p = 0.006)
**Varudo et al. (2022)**^105^ ICU case report	Use of VExUS as a rapid tool to assess volume status in complex hyponatremia	Overall VExUS grade 2 prompted a diuretic strategy with clinical improvement
**Rolston et al. (2022)**^106^ Single-center observational study; 150 septic patients	VExUS grading in septic patients prior to fluid therapy	Composite outcome (mortality, ICU admission, or rapid response activation): VExUS 0: 31.6%; VExUS 1: 47.6%; VExUS > 1: 67.7% (p = 0.0015)
**Guinot et al. (2022)**^107^ Prospective ICU observational study; 81 patients starting loop diuretics	Comparison of portal vein pulsatility index, kidney venous impedance index, and VExUS	Portal vein pulsatility and kidney venous impedance indices outperformed VExUS; baseline VExUS had limited predictive value (AUC 0.66; 95% CI 0.53-0.79; p = 0.012)
**Menéndez-Suso et al. (2023)**^108^ Pediatric ICU cross-sectional pilot study; 33 children	Association between VExUS score and CVP	VExUS severity strongly associated with CVP (p < 0.001)
**Longino et al. (2023)**^109^ Single-center prospective study; 81 patients undergoing right heart catheterization	Validation of association between VExUS grade and right atrial pressure	Favorable AUC for predict-ing right atrial pressure > 10 mm Hg (AUC 0.90; 95% CI 0.83-0.97)
**Torres-Arrese et al. (2023)**^110^ Multicenter prospective study; 74 patients with acute heart failure	Evaluation of ultrasound parameters, including VExUS, as predictors of clinical outcomes	VExUS grade 3 predicted in-hospital mortality (AUC 0.885; sensitivity 80%; specificity 75%; PPV 33%; NPV 96%)
**Magin et al. (2023)**^111^ Single-center prospective pilot study; 69 patients undergoing noncardiac surgery	Feasibility of VExUS assessment in perioperative settings	VExUS feasible for as-sessing venous congestion after noncardiac surgery; trend toward increased congestion after abdominal surgery.
**Viana-Rojas et al. (2023)**^112^ Prospective study; 77 patients with acute coronary syndrome	Association between VExUS and AKI	VExUS ≥ 1 associated with AKI (OR 6.15; 95% CI 1.26-29.94; p = 0.02); VExUS ≥ 1 more prevalent in inferior acute myocardial infarction

+LR: positive likelihood ratio; AKI: acute kidney injury; AUC: area under the curve; CVP: central venous pressure; HR: hazard ratio; ICU: intensive care unit; NPV: negative predictive value; OR: odds ratio; PPV: positive predictive value; VExUS: venous excess ultrasound.

### 4.3. Venous Excess Ultrasound Components

For VExUS assessment, either a phased-array or convex transducer may be used, with reduction of wall filters and adjustment of the velocity scale to venous flow settings. We recommend the following step-by-step sequence for each vessel evaluated, detailing the optimal approach to obtain flow waveforms and thereby minimizing potential interpretation errors. Final grading of systemic venous congestion should follow the model proposed in Figure 15.

**Figure 15:** VExUS grading: when the IVC diameter exceeds 2 cm, three grades of venous congestion are defined according to the severity of abnormalities observed in the flow patterns of the hepatic, portal, and renal interlobar veins, which should ideally be recorded during end-expiratory apnea. IVC: inferior vena cava; VExUS: venous excess ultrasound. Adapted from Koratala et al.^121^

#### Step 1: Inferior vena cava assessment

IVC assessment is preferably performed via the subcostal approach, with the transducer index oriented toward the 12 o'clock position. In this view, the IVC drains longitudinally into the right atrium (RA). Less ideally, IVC assessment may be performed from the mid-axillary line with the index also oriented toward 12 o'clock (transhepatic window). This latter approach frequently overestimates IVC diameter and should therefore be avoided.

IVC diameter is measured 2 cm proximal to its junction with the RA, with the patient in end-expiratory apnea. The left hepatic vein often drains near the measurement site and should not be included in the measurement.

If the IVC diameter is < 2 cm, systemic venous congestion is considered absent, and VExUS is graded as 0 (no congestion), with no need to evaluate the remaining components (hepatic vein, portal vein, and kidney interlobar vein).

If the diameter is ≥ 2 cm, venous congestion may be present and further assessment of the remaining components should be performed. Use of this parameter helps eliminate potential false-positive diagnoses of systemic venous congestion.^103^

#### Step 2: Hepatic vein assessment using pulsed-wave Doppler

Hepatic vein assessment may be performed via the subcostal or transhepatic approach (mid-axillary line, with anterior angulation of the transducer if needed), adjusting the color Doppler velocity scale to 30-40 cm/s. The vein selected for analysis should be the one most vertically aligned with the transducer, allowing optimal acquisition of flow waveforms.

Because the hepatic veins are directly connected to a central vein (IVC), they transmit venous pulsatility through four Doppler waveforms identifiable on pulsed-wave Doppler:^113^ **S wave**, negative and dominant, resulting from right atrial relaxation and downward displacement of the tricuspid annulus toward the cardiac apex during ventricular systole; **V wave**, not always present, usually positive, occurring when the tricuspid annulus returns to its diastolic position; **D wave**, negative and smaller than the S wave, resulting from tricuspid valve opening and blood inflow from the RA to the right ventricle; **A wave**, positive, caused by atrial contraction.

The S wave occurs immediately after the R wave of the electrocardiogram (ECG), the D wave follows the T wave, and the A wave correlates directly with the P wave of the ECG and is absent in atrial fibrillation.^114^ Therefore, ECG monitoring is essential for accurate differentiation of venous waveforms.

According to RA pressure (RAP), hepatic vein Doppler waveforms may be classified into three patterns:^115^

1. Normal (type 1): S wave larger than D wave.
2. Increased RAP (type 2): S wave smaller than D wave, reflecting predominant atrial emptying during diastole.^116^
3. Markedly increased RAP (type 3): inversion of the S wave, which becomes positive, while the D wave remains negative.

#### Step 3: Portal vein assessment using pulsed-wave Doppler

Portal vein assessment is ideally performed via the transhepatic window (mid-axillary line), with the transducer index oriented toward 12 o'clock and the color Doppler velocity scale adjusted to 20-30 cm/s. It may also be visualized using the subcostal approach. In this orientation, portal vein flow is ascending, appears red on color Doppler, and exhibits a continuous or mildly pulsatile pattern. Due to its thicker and hyperechoic walls, the portal vein is easily distinguished from the hepatic veins, which lack visible walls and exhibit flow directed toward the IVC, appearing blue on color Doppler.

Because it is separated from the central systemic venous circulation by hepatic sinusoids, portal vein flow is typically continuous or mildly pulsatile, with a positive waveform generated by atrial contraction.^117^ As systemic venous pressure increases, sinusoidal dilation occurs due to congestion, and venous pulsatility is increasingly transmitted to the portal vein. Thus, the greater the systemic venous congestion, the greater the portal vein pulsatility, quantified by the pulsatility fraction (pulsatility fraction = [peak velocity − minimum velocity] / peak velocity × 100).

Similar to hepatic veins, RAP alters portal vein Doppler morphology, which can be classified into three patterns:^103^

1. Normal (type 1): preserved sinusoids with flow oscillation < 30%;
2. Increased RAP (type 2): sinusoidal dilation with flow oscillation between 31% and 50%;
3. Markedly increased RAP (type 3): severe sinusoidal dilation with marked transmission of systemic venous pulsatility to the portal vein, with oscillation > 50%; in some cases, flow velocity may cross below the baseline (negative velocity).^118^

#### Step 4: Kidney interlobar vein assessment using pulsed-wave Doppler

Assessment of the kidney interlobar vein is of critical importance in VExUS grading, as it shows the strongest isolated correlation with progression to kidney failure in patients with severe systemic venous congestion.

Because the kidney is an encapsulated organ, it does not normally transmit central venous pulsatility, and venous flow is typically continuous. However, as systemic venous congestion develops (congestive nephropathy), interstitial kidney edema occurs, venous compliance decreases, and flow becomes biphasic or, in more severe cases, monophasic.^119,120^

Due to the close proximity of arteries and veins, parenchymal arterial flow is visualized above the Doppler baseline, whereas venous flow appears below the baseline.

Kidney veins at the renal hilum should not be confused with intrarenal veins located in the cortical region, as the renal vein is a central vein with pulsatile flow and represents a frequent source of error.

For correct acquisition of interlobar vein flow, Doppler sampling should be performed in the kidney cortex, with the color Doppler velocity scale reduced to approximately 20 cm/s to enhance flow visualization.

According to systemic venous pressure, kidney interlobar vein flow patterns are classified into three types:

1. Normal (type 1): continuous venous flow throughout the cardiac cycle;
2. Increased RAP (type 2): biphasic venous flow with one systolic and one diastolic peak;
3. Markedly increased RAP (type 3): with further elevation of venous pressure and kidney edema, only the diastolic peak remains, resulting in monophasic flow.

For optimal acquisition of venous waveforms in the VExUS protocol, respiratory apnea should be performed whenever possible.

#### Step 5: data integration and VExUS grading

Once the step-by-step analysis as described has been performed, the data should be integrated to allow appropriate grading of the degree of visceral venous congestion.

### 4.4. Venous Excess Ultrasound Grading

Quantification of the degree of visceral venous congestion is based on the original study by Beaubien-Souligny et al.,^103^ in which five prototypes of a grading system, termed the VExUS score, were validated in a population of 145 postoperative cardiac surgery patients with respect to the risk of developing kidney failure. A post hoc analysis of this single-center prospective study identified the prototype with the best diagnostic performance, leading to the proposal of a four-grade classification of venous congestion based on the abnormalities observed in the VExUS components (Figure 15).

The study included patients receiving mechanical ventilation, thereby expanding the practical applicability of the VExUS score. This grading system, when used in isolation, is not capable of distinguishing visceral venous congestion caused by volume overload from that caused by pressure overload. Therefore, findings must be interpreted in an integrated manner, taking into account the clinical context and other POCUS findings.^121^

### 4.5. Clinical Applications

Overall, the VExUS score should not be interpreted in isolation nor considered a substitute for careful history taking and conventional physical examination. However, it can provide valuable information in the management of critically ill patients, particularly in conditions or states associated with elevated venous pressure, which increase the risk of significant systemic venous congestion, impaired tissue perfusion, and subsequent renal dysfunction.

VExUS plays an important role in fluid balance management. Although it does not provide direct information regarding the need for volume expansion, it serves as a key indicator of FT, helping to identify situations in which discontinuation of fluid resuscitation or active fluid removal is likely to be beneficial, especially in complex scenarios associated with AKI (Figure 16).

**Figure 16:** Algorithm incorporating venous excess ultrasound into the management of critically ill patients. AKI: acute kidney injury; IV: intravenous; VExUS: venous excess ultrasound score. Adapted from Bhardwaj et al.^104^ e Argaiz et al.^122^

Across various clinical contexts, recent studies have demonstrated potentially useful applications of this tool (Table 5): prediction of AKI risk in the perioperative period of cardiac surgery; monitoring of decongestive therapy in patients with cardiorenal syndrome; assessment of volume status in complex hyponatremia; evaluation of AKI risk in ischemic coronary syndromes; estimation of CVP in critically ill children; and assessment of volume status in pulmonary hypertension, right HF, or mechanically ventilated patients, in whom CVP may not reliably reflect preload.

### 4.6. Limitations and Pitfalls

The first step in the assessment of venous congestion should always be evaluation of the IVC. However, isolated assessment of the IVC has several limitations, beginning with its modest correlation with right atrial pressure measured by catheterization studies.^121^ In addition, assessment of IVC collapsibility depends on the patient's inspiratory effort and may vary considerably according to clinical conditions. Athletes and young individuals may present with a persistently dilated IVC without elevated right atrial pressure, whereas patients with increased intra-abdominal pressure may exhibit a collapsed IVC despite elevated right atrial pressure. Furthermore, IVC assessment using only the long-axis view is subject to the cylinder effect, which occurs when a two-dimensional ultrasound beam intersects a three-dimensional structure peripherally rather than centrally, potentially underestimating its true diameter.

Regarding hepatic vein assessment, failure to use simultaneous ECG tracing may lead to significant interpretation errors,^103^ particularly in the presence of arrhythmias. Atrial fibrillation may cause reduction of the S wave in patients with normal right atrial pressure, as this wave partially depends on atrial relaxation. In patients with tricuspid regurgitation or pulmonary arterial hypertension, a chronic pattern of S wave smaller than D wave or S wave reversal may be observed regardless of volume status. Right ventricular dysfunction, due to reduced downward displacement of the tricuspid annulus during ventricular systole, may also reduce or abolish the S wave.

Portal vein assessment also has important limitations.^121,123^ Lean individuals with low body mass index may exhibit high portal vein pulsatility even in the presence of normal right atrial pressure. In patients with chronic liver disease, the spectral waveform reflects local pressure changes more than CVPs, such that flow may appear pulsatile even in euvolemic patients due to the presence of portoarterial shunts. Patients with portal hypertension may exhibit reversed (hepatofugal) flow, whereas in those with liver cirrhosis the waveform may appear relatively normal despite tricuspid regurgitation and elevated right atrial pressure, owing to increased sinusoidal resistance caused by hepatic fibrosis.

Finally, the main limitation of renal interlobar vein flow assessment is its technical difficulty.^121^ Intrarenal vessels are small and frequently move out of the imaging plane as the kidney shifts with respiration. Respiratory breath-holding may facilitate acquisition but is not always feasible depending on the patient's clinical condition. Obesity and dyspnea may preclude adequate Doppler tracing. In addition, factors other than right atrial pressure may alter venous flow patterns, such as structural renal abnormalities and chronic kidney disease.^123^

### 4.7. Conclusion

The VExUS score has demonstrated high specificity for the diagnosis of systemic venous congestion and prognostic value for AKI following cardiac surgery. It is a method that can be easily performed by trained professionals and represents a potentially useful tool in the clinical management of critically ill patients.

## 5. POCUS in Deep Vein Thrombosis

### 5.1. Introduction

DVT is a common vascular condition, occurring in approximately 30% of hospitalized patients.^124–126^ Pulmonary embolism is a complication of DVT and represents the third most frequent cause of acute cardiovascular syndrome, surpassed only by AMI and stroke. In Brazil, between 2016 and 2020, more than 34,000 deaths were attributed to venous thromboembolism.^127^ Mortality from pulmonary embolism may reach 30% if not properly treated, and it remains one of the most frequent unsuspected findings in autopsy studies of critically ill patients.^128–130^ Rapid and accurate diagnosis is therefore essential for timely treatment of DVT and prevention of complications. The Joint Guideline on Venous Thromboembolism – 2022 was developed by a task force of Brazilian medical societies (Department of Cardiovascular Imaging, Brazilian Society of Cardiology; Brazilian College of Radiology; Brazilian Society of Angiology and Vascular Surgery; Brazilian Society of Nuclear Medicine) with the aim of guiding the diagnostic approach to DVT and pulmonary embolism.^131^

In DVT, thrombi form in areas of reduced venous flow and subsequently propagate along the vessel lumen. During the acute phase, an inflammatory response develops in the venous wall, with partial or complete luminal obstruction and associated clinical symptoms such as pain, edema, and erythema of the affected limb. However, in approximately 50% of cases, clinical signs of DVT are nonspecific, making diagnosis challenging. Complementary diagnostic tests are therefore required to confirm or exclude DVT. According to the 2022 Brazilian Guideline, in patients with suspected DVT, use of the simplified Wells score as a clinical prediction model is recommended, in combination with D-dimer measurement and vascular ultrasonography (VUS).^131–133^

VUS is the diagnostic test of choice in suspected DVT, providing anatomical and functional venous information. Its advantages include being noninvasive, not requiring nephrotoxic contrast agents, being reproducible, and having low cost.^134,135^

In this chapter, we present the different POCUS protocols for assessment of DVT. Based on the 2022 Brazilian Guideline, we recommend use of the extended compression protocol, also known as the 3-point compression ultrasound (CUS) protocol.^131^ This recommendation is supported by the most recent European Society of Intensive Care Medicine consensus, the latest Emergency Ultrasound Guideline of the American College of Emergency Physicians, and its inclusion in the Critical Care Ultrasonography Certificate of Completion Program of the American College of Chest Physicians.^7,136^

### 5.2. Scientific Evidence for POCUS in Assessment of Deep Vein Thrombosis

Regarding the diagnostic accuracy of bedside CUS performed by emergency physicians, a meta-analysis by Pomero et al.^137^ reported a sensitivity of 96.1% (95% CI 90.6%-98.5%) and a specificity of 96.8% (95% CI 94.6%-98.1%). However, the included studies showed substantial heterogeneity, likely related to limited operator training, presence of distal or intra-abdominal thrombosis, and patients with challenging body habitus.^139^

In a study by Garcia et al.,^139^ the accuracy of CUS for diagnosing DVT in patients with high clinical suspicion, performed in the emergency department, was 91.7% compared with comprehensive examinations performed by specialists within 48 hours. In critically ill patients, Kory et al.^140^ demonstrated a diagnostic accuracy of 95% for CUS performed by intensivists with less than 2 years of ultrasound experience. Moreover, diagnosis was established, on average, 13 hours earlier than by specialist-performed examinations. A multicenter study evaluating the accuracy of CUS performed by emergency physicians in patients with high suspicion of pulmonary embolism/DVT reported a high negative predictive value (100%) and a moderate positive predictive value (61.5%), with sensitivity of 100% and specificity of 95.8%, compared with formal ultrasound performed by specialists. A reduction of approximately 50% in the time between POCUS and formal ultrasound was also observed.^141^

Collectively, these studies demonstrate the utility of CUS in the management of patients with suspected DVT in emergency and intensive care settings. Additionally, the rapid execution of these examinations - typically in less than 5 minutes - may prevent unnecessary comprehensive studies and diagnostic delays, particularly outside regular working hours or in settings with limited specialist availability. Regarding comparison between 2-point and 3-point CUS protocols, a recent meta-analysis including 17 studies found that both protocols exhibited excellent performance, with sensitivities and specificities of approximately 90% and false-positive and false-negative rates of about 4%. Diagnostic accuracy tended to be higher when examinations were performed by emergency physicians rather than residents.^142^

Due to its lower prevalence, studies evaluating POCUS for upper extremity thrombosis in emergency settings remain scarce, and standardized protocols are less well established.

### 5.3. Technique of the Focused Ultrasound Protocol for Assessment of Deep Vein Thrombosis

Application of concise bedside ultrasound protocols, as opposed to full examinations, offers several advantages in emergency and urgent care settings, including rapid diagnosis enabling early treatment initiation, continuous monitoring with therapy adjustments as needed, and reduced length of hospital stay, benefiting both patients and health care systems.

Nevertheless, although POCUS provides substantial benefits, its application must be appropriately integrated into the clinical context, taking into account patient-specific needs and ensuring result reliability.

Ultrasound equipment ranges from portable handheld devices to advanced machines with varying costs. Chart 2 summarizes key technical tips for performing the POCUS protocol for assessment of DVT.^143^

**Chart 2:** Technical tips for performing the POCUS protocol for assessment of DVT

Guidelines for POCUS protocol in the investigation of DVT
•Become familiar with the ultrasound equipment available in the unit.
•Select the appropriate transducer: (1) linear, 5-10 MHz; (2) convex, 2-5 MHz (occasionally in obese or edematous patients).
•Choose the venous examination preset, if available.
•Adjust basic two-dimensional imaging parameters, including gain, depth, and focus.
•Observe the transducer index, aligning it with the screen marker.
•Ensure adequate availability of ultrasound gel.
•Record images for follow-up comparison.

DVT: deep vein thrombosis; POCUS: point-of-care ultrasound.

Patients should preferably be examined in the supine position in bed, with the head elevated at approximately 30°. For lower limb assessment, external rotation with slight knee flexion is recommended to allow evaluation of the inguinal region and popliteal fossa without repositioning the limb.^143^

For both 2-point and 3-point CUS protocols, grayscale imaging is used to identify intraluminal echogenic material suggestive of thrombus. Compression of the vein must be performed with the transducer positioned transversely.^143,144^ Figure 17 schematically illustrates the CUS protocols.

**Figure 17:** Extended compression ultrasound (CUS) protocol (3-point; A); CUS protocol (2-point; B).

The 2-point protocol consists of evaluation of the common femoral vein and the popliteal vein. Examination begins in the inguinal region, encompassing the segment 1-2 cm above and below the saphenofemoral junction, respectively. The popliteal fossa is then assessed, where the vein runs parallel to the popliteal artery until the confluence of the calf veins. The 3-point protocol evaluates the common femoral and popliteal veins in a manner similar to the 2-point protocol, with the addition of compression of the femoral vein at proximal and mid-distal thigh segments.

Regardless of the protocol used, within the POCUS framework, DVT diagnosis is established by visualization of thrombus (intraluminal echogenic material) or by identification of venous incompressibility. In acute thrombosis, venous incompressibility often precedes direct visualization of the thrombus. Therefore, adequate transducer pressure must be applied to collapse the venous wall, avoiding overly gentle compression that may result in false-negative findings.^143^ Figure 18 illustrates the ultrasound scan sequence for the extended 3-point compression protocol.

**Figure 18:** Scan sequence for extended 3-point compression ultrasound protocol.

Figure 19 displays ultrasound findings in normal veins and in veins with acute thrombosis, comparing venous caliber, compressibility, luminal characteristics, and thrombus echogenicity.

**Figure 19:** Ultrasound findings in normal veins and veins with signs of acute.

Clinical signs and symptoms of thrombotic events may overlap with those of several acute or chronic conditions. Therefore, it is essential to consider differential diagnoses, including ruptured synovial cysts, chronic venous insufficiency, muscle ruptures and hematomas, abscesses, synovial cysts, lymphadenopathy, tumors, and Baker cyst, among others.^131^

Health professionals must become familiar with the devices and specific techniques employed in the POCUS protocols they use, thereby ensuring accuracy and reliability of bedside results. Interdisciplinary collaboration and ongoing training are also fundamental to the successful implementation of POCUS protocols for DVT assessment.

Recommendation: POCUS for proximal assessment of DVT, when performed by adequately trained professionals in emergency and urgent care settings, carries a Grade I recommendation, Level B evidence.

## 6. POCUS in the Patient with Circulatory Shock

### 6.1. Introduction

Shock can be defined as a severe state of generalized circulatory failure that leads to inadequate oxygen delivery to tissues. The four basic hemodynamic types of shock are hypovolemic, cardiogenic, obstructive, and distributive. Each has a different treatment approach, which makes rapid diagnosis and therapy essential. POCUS is the method of choice for the initial hemodynamic evaluation and monitoring of patients with circulatory shock. It allows rapid identification of the hemodynamic profile, often within 2 minutes, even when performed by non-specialists.^145^

For clinicians with basic training, the main focus should be qualitative image assessment. Making clinical decisions based on quantitative measurements without adequate proficiency may compromise patient management.

Several bedside ultrasound protocols have been proposed for cardiac arrest (Focused Echocardiographic Evaluation in Resuscitation, Pulseless Electrical Activity, Cardiac Arrest Sonographic Assessment [CASA]),^3^ shock (Ultrasound Hypotension Protocol, Trinity, Rapid Ultrasound for Shock and Hypotension, Focus-Assessed Transthoracic Echocardiography, Echo Guided Life Support [EGLS]),^4,5^ and respiratory failure (BLUE).^6^

The goal of this chapter is to present a diagnostic algorithm for circulatory shock based on POCUS, similar to an adapted EGLS protocol, designed to be feasible at the bedside by non-imaging specialists working in emergency or critical care settings.

### 6.2. Diagnostic Protocols

This section presents an integrated approach that combines the principles of BLUE, cardiac POCUS, and the Extended Focused Assessment with Sonography in Trauma (EFAST)^7^ and EGLS protocols.^8^ We believe that, given the close relationship between pulmonary extravascular water, LV function, and volume status in shock states, the combined use of lung and cardiac ultrasound together with the EFAST examination offers a clear advantage over isolated cardiac POCUS or EFAST in patients with undifferentiated shock.

### 6.3. Key questions to Identify the Type of Shock

We propose a sequence of five key questions, aligned with their corresponding ultrasound findings and implicit therapeutic implications, designed to rapidly identify and manage conditions that present a characteristic "ultrasound signature"^2,9^ (Figure 20). These questions are:

**Figure 20:** Adapted EGLS protocol.^8^ CT: Computed tomography EFAST: Extended Focused Assessment with Sonography in Trauma; EGLS: Echo Guided Life Support; LV: Left ventricle; LVOT: LV outflow tract; VTI: Velocity-time integral; RA: Right Atrium; TTE: Transthoracic echocardiography; VExUS: Venous Excess Ultrasound Score.

- Is there cardiac tamponade?
- Is the patient hypovolemic?
- If left ventricular systolic dysfunction is present, is it the primary cause of shock?
- Are there signs of right ventricular dilation?
- Is there pneumothorax?

Each of these questions is discussed below.

#### a . Is there cardiac tamponade?

The evaluation should focus on the presence of moderate to large pericardial effusion, a dilated IVC with minimal respiratory variation, diastolic collapse of cardiac chambers, and the presence of a swinging heart.^9^

The pathophysiology of tamponade is suggested by a pericardial effusion causing atrial or right ventricular diastolic collapse. In cases of massive effusion, a pendular motion of the heart ("swinging heart") may be observed. Cardiac tamponade should be associated with increased CVP, which can be demonstrated in the subcostal view by a plethoric IVC (≥ 20 mm) without respiratory variation.

These findings are discussed in greater detail in Section 4 of Chapter 2 (POCUS in cardiac tamponade).

#### b. Is the patient hypovolemic?

The assessment should include identification of a hyperdynamic or hypovolemic LV pattern ("kissing walls"), a narrow IVC (< 10 mm), or marked IVC collapse throughout the respiratory cycle.^146^

In hypovolemic shock, LUS is expected to show normal aeration (absence of B-lines), in association with a hyperdynamic LV and a collapsible IVC. In this context, investigation of potential causes of hypovolemia is warranted, and an EFAST examination may be considered.

A small IVC (diameter < 10 mm) has been shown to correlate with hypovolemia in trauma patients. Therefore, significant respiratory variation or collapse of the IVC in patients with shock should always raise suspicion of hypovolemia,^10,11^ particularly when associated with a hyperdynamic LV.

In the early phases of sepsis, patients may also present with a hyperdynamic cardiac profile. Accordingly, sepsis should be actively investigated as a potential underlying cause.^12^

#### c. If left ventricular systolic dysfunction is present, is it the primary cause of shock?

The evaluation should focus on identifying significant LV systolic dysfunction and signs of pulmonary and systemic venous congestion.

For shock to be purely cardiogenic, marked LV systolic dysfunction must be present. These patients typically also exhibit signs of pulmonary congestion (B-line pattern on LUS) and/or systemic venous congestion (IVC dilation).^13^

Pulmonary interstitial syndrome is defined by the presence of at least three B-lines within the width of a single intercostal space (the "B profile"). Although this pattern may represent cardiogenic pulmonary edema,^13,14^ it is not specific and may also be observed in other interstitial lung diseases,^16^ such as pulmonary fibrosis, ARDS, or pulmonary contusions. A cardiogenic origin is more likely when B-lines are homogeneously distributed, bilateral, and gravity dependent, with greater involvement of the lung bases.

Pure cardiogenic shock (CS) is unlikely to be the primary cause of hemodynamic instability in the presence of a normal LUS pattern, suggesting that fluid administration is probably safe in such cases.^17^

Prior knowledge of baseline echocardiographic and LUS findings may assist in interpretation. For example, LV systolic dysfunction combined with normal lung findings suggests pre-existing cardiac disease rather than acute CS, particularly when the IVC is small.

In mixed shock states (cardiogenic and distributive), such as septic shock with sepsis-induced myocardial dysfunction, mild cardiac dysfunction may coexist with vasoplegia or hypovolemia, explaining the presence of shock in the absence of severe LV dysfunction.^18^ In septic shock, a normal heart is expected to be somewhat hyperdynamic due to reduced systemic vascular resistance. Therefore, borderline LV systolic function in this context should raise suspicion of underlying or sepsis-related myocardial dysfunction, which may become more apparent after escalation of vasopressor therapy.

Severe acute mitral regurgitation, such as that caused by chordae tendineae rupture, is another condition that may present with shock and a hyperdynamic LV. This scenario may be misinterpreted as hypovolemia by nonexperts with limited Doppler experience. However, unlike hypovolemia, severe acute mitral regurgitation is typically associated with a B-line pattern and a less compliant IVC, reflecting elevated filling pressures. This illustrates how LUS findings can directly influence the interpretation of cardiac POCUS. In such cases, evaluation by an imaging specialist (echocardiographer) is recommended.

#### d. Are there signs of right ventricular dilation?

The assessment should include identification of moderate to severe right-sided chamber dilation and flattening of the interventricular septum (D-shape).

In obstructive shock, the IVC is expected to be dilated. An IVC diameter > 20 mm with loss of respiratory variability suggests elevated CVP.^4,18^

If acute cor pulmonale is the cause of obstructive shock, right-sided chamber dilation and interventricular septal flattening must be present, resulting in a D-shaped LV on parasternal short-axis view (PSAX; Figure 7).^19,20^ The A4C view is the best window for assessing RV-to-LV size ratios. The severity of cor pulmonale may be estimated as follows:

- *Mild: RV > 60% of LV;*
- *Moderate: RV = LV;*
- *Severe: RV > LV.*

#### e. Is there pneumothorax?^21^

The assessment should focus on the absence of pleural sliding and the presence of the lung point. The presence of pleural sliding excludes pneumothorax beneath the probe and requires only minimal training to be recognized. Although not specific (Figure 20), the absence of pleural sliding in the appropriate clinical context may be highly suggestive of pneumothorax. The lung point represents a more specific finding for this diagnosis and is described in detail in the LUS chapter.

The absence of a lung point does not exclude pneumothorax. In cases of circumferential pneumothorax, this finding will not be observed. The presence of a B-line pattern excludes pneumothorax in the scanned region.

### 6.4. Fluid Responsiveness and Fluid Tolerance

Depending on the most likely type of shock, additional complementary assessments may be performed according to the examiner's level of experience and the suspected underlying cause.

Despite variations in methodology and definitions across the literature, the most widely accepted concept of FR is an increase in cardiac output greater than 10%-15% induced by an increase in preload.^22–24^

#### 6.4.1. Estimation of Cardiac Output

The primary component for estimating cardiac output using cardiac POCUS is the LVOT VTI.^22^ This concept is addressed in Section 2 on advanced cardiac POCUS. The normal LVOT VTI ranges from approximately 18-22 cm in patients with a heart rate between 55 and 95 bpm.^51^ When systolic function is preserved and the VTI is low, hypovolemic shock should be considered. Conversely, preserved systolic function with an elevated VTI suggests distributive shock. The assessment of LVOT VTI is also discussed below in the evaluation of FR.

#### 6.4.2. Inferior and Superior Vena Cava

Assessment of the maximum diameter and respiratory variability of IVC may be used to estimate RAP and FR. For RAP estimation, the IVC has greater accuracy in spontaneously breathing patients.^147^ Regarding FR estimation, indices based on IVC variability in patients under controlled mechanical ventilation, without spontaneous respiratory effort and with near-normal tidal volumes and positive end-expiratory pressure (PEEP), showed good accuracy in early studies (approximately 90%). However, this performance was not confirmed in subsequent publications, in which accuracy decreased to about 65%.^148–150^ Assessment of SVC demonstrated better accuracy (approximately 75%) than the IVC for predicting FR, although SVC evaluation requires transesophageal echocardiography (TEE).^150^ In spontaneously breathing patients, several studies have shown that IVC accuracy is also limited, with higher specificity than sensitivity.

Thus, the assessment of IVC may assist in evaluating volume status and FR, particularly in extreme scenarios:^8^

- A very small IVC (≤ 10 mm) or marked collapse ➔ likely FR and FT;
- A markedly dilated IVC (> 25 mm) with minimal respiratory variation ➔ likely absence of FR and FT.

Respiratory variation of the IVC is frequently altered in patients receiving mechanical ventilation, in cirrhosis, and in chronic lung disease, and should therefore be interpreted with caution. Although IVC respiratory variation may predict FR in hypotensive patients under mechanical ventilation, a plethoric IVC without respiratory variation is not, by itself, a contraindication to fluid administration in this population. As with elevated CVP, the absence of respiratory variation does not necessarily indicate lack of FR and must be interpreted within the clinical context.

#### 6.4.3. Passive Leg Raising Maneuver

Among POCUS-based methods for estimating FR, the passive leg raising (PLR) maneuver is the most extensively studied.^151,152^ PLR involves measuring LVOT VTI with the patient in a semi-recumbent position (head of bed elevated at 45°) and the legs extended. The head of the bed is then lowered to the supine position while the lower limbs are elevated to 45°, and the VTI is reassessed approximately 1 minute after leg elevation. These maneuvers must be performed passively by adjusting the bed position rather than through active patient movement. An increase in LVOT VTI of ≥ 10% induced by PLR predicts an increase in cardiac output after volume infusion with an accuracy of approximately 84%, according to recent studies.^153^

However, both intra- and interobserver variability affect LVOT VTI measurements. A recent ICU study showed that when the same examiner performed two measurements at different times without moving the transducer, variability reached approximately 11% (range 5%-18%). When measurements were performed by different operators, variability increased to approximately 14% (range 8%-26%).^154^ Therefore, therapeutic decisions based solely on this parameter require caution. Changes in VTI close to the intraobserver variability threshold (11%) may represent false-positive results. Consequently, a more substantial VTI increase (> 20%) following PLR more reliably indicates true FR.

An advantage of PLR is that it can be performed in both spontaneously breathing and mechanically ventilated patients, with or without respiratory effort, even at low tidal volumes. However, the maneuver requires greater operator experience to ensure rapid and accurate VTI measurements. It is recommended to average at least three measurements at end-expiration in patients with sinus rhythm and at least five measurements in patients with atrial fibrillation.^154^ Factors that limit the applicability of PLR include intra-abdominal hypertension, inability to mobilize the lower limbs (e.g., fractures), lower limb thrombosis, and intracranial hypertension.

#### 6.4.4. Expiratory and Inspiratory Occlusion Maneuvers

Some studies have shown that an expiratory hold lasting 12-15 seconds is associated with an increase in VTI or cardiac output in fluid-responsive patients. Similarly, an inspiratory hold of 12-15 seconds is associated with a reduction in VTI or cardiac output in these patients.^33^ These assessments may be performed using ultrasound or other cardiac output monitoring devices. However, because the magnitude of cardiac output variation induced by these maneuvers approaches the limits of intraobserver variability when assessed by POCUS, an integrated approach combining both maneuvers has been proposed.

A combined absolute LVOT VTI variation of at least 13%, obtained using end-expiratory occlusion test and end-inspiratory occlusion test, each lasting approximately 12-15 seconds, demonstrated an accuracy of 93% for predicting FR. This approach should not be performed by operators with limited experience.

It must be emphasized that no single parameter for estimating FR is perfect. Multiple factors related to the patient, equipment, and operator can influence accuracy.^153^ Moreover, FR does not necessarily imply FT. Decisions regarding fluid administration must always balance the individualized risks of shock against the risks of hypoxemia and congestion.^155^

FT is defined as the ability of the organism to receive fluid infusion without developing organ dysfunction. FT can be assessed using LUS to detect B-lines (pulmonary congestion) and the VExUS protocol to evaluate systemic venous congestion. The VExUS protocol, methodology, and interpretation are described in Section 4 of this document. For FT assessment, the protocol may be applied as follows:

- VExUS grades 0-1 (no significant congestion): fluid tolerant;
- VExUS grades 2-3 (significant congestion): fluid intolerant.

Once FR is confirmed, fluid infusion may be initiated, with response monitoring guided by LUS and VExUS, according to the algorithm shown in Figure 21.^146^

**Figure 21:** Integrated approach to fluid responsiveness and fluid tolerance.^156^

Clinicians managing patients at the bedside should always integrate POCUS findings into their clinical reasoning, combining them with history taking, physical examination, and other diagnostic tests.

Based on the topics discussed throughout this chapter, we recommend the use of an initial POCUS-based algorithm for the evaluation of circulatory shock, which may be further expanded according to the observer's level of expertise (Figure 21).

## 7. POCUS in Short-Term Mechanical Circulatory Support

### 7.1. Introduction

CS is characterized by cardiac dysfunction leading to systemic hypoperfusion and end-organ failure. The most common cause of CS is AMI. Despite advances in early revascularization, the incidence of CS complicating AMI remains between 6.5% and 10.1%.^157^ Other frequent causes include worsening of pre-existing ventricular dysfunction and postoperative cardiac dysfunction. CS is associated with high mortality, with an estimated in-hospital mortality rate of approximately 50%.^158^ Medical therapy alone is often insufficient, making short-term mechanical circulatory support (ST-MCS) necessary.

ST-MCS devices provide hemodynamic support in two main clinical scenarios: i) during high-risk cardiac procedures in patients with low cardiac output, and ii) in CS due to left or right ventricular failure. ST-MCS devices include the intra-aortic balloon pump (IABP), venoarterial (VA) ECMO (VA-ECMO), and percutaneous ventricular assist devices (pVADs), such as Impella^®^. In addition, Impella^®^ devices may help unload the LV during VA-ECMO. Recently, the use of Impella^®^ has demonstrated a mortality benefit compared with standard medical therapy in patients with AMI-related CS.^159^ The primary goals of ST-MCS devices are to provide circulatory support and to mitigate end-organ dysfunction in the setting of inadequate cardiac output.

POCUS has transformed bedside diagnosis and management across multiple clinical scenarios. Its application in ST-MCS is particularly relevant due to the critical condition of these patients. Integrating POCUS into patient management provides real-time, noninvasive information that is essential for timely and effective clinical decision-making.

### 7.2. The Role of POCUS in Short-Term Mechanical Circulatory Support

POCUS is an invaluable tool and is recommended for clinicians managing patients on ST-MCS since it provides immediate visual feedback on cardiac function, device positioning, and potential complications. Its role encompasses several stages of care, including initial patient assessment, device placement, and ongoing monitoring.

### 7.3. Initial Patient Assessment

In the context of ST-MCS, the initial assessment is critical to determine appropriateness for support and to guide procedural planning. POCUS allows rapid evaluation of cardiac function, volume status, and other hemodynamic parameters. For example, in patients with CS, echocardiography can quickly assess left and right ventricular function, valvular abnormalities, and the presence of pericardial effusion. These findings are essential for determining the type and urgency of mechanical support required.

**Figure 22:** Initial diagnostic algorithm for circulatory shock based on POCUS. LV: left ventricle; RV: right ventricle; IVC: inferior vena cava; VTI: velocity time integral; TTE: transthoracic echocardiography; X-ray: radiography; MNM: myocardial necrosis marker; PASP: pulmonary artery systolic pressure; PAT: pulmonary acceleration time; mPAP: mean pulmonary artery pressure; TAPSE: tricuspid annular plane systolic excursion; FAC: fractional area change; CT: computed tomography; VExUS: Venous Excess Ultrasound Score; PLAPS: posterolateral alveolar–pleural syndrome. Adapted from the EGLS, BLUE, and EFAST protocols.

### 7.4. Device Placement and Position Confirmation

Accurate positioning of mechanical support devices is essential for optimal performance and for preventing complications. POCUS plays a key role in guiding and confirming the placement of devices such as the IABP, ECMO cannulas, and pVADs.

### 7.5. Intra-Aortic Balloon Pump

#### 7.5.1. Introduction

Although more recent European Society of Cardiology guidelines on HF discourage the routine use of the IABP,^160^ it remains the most widely used short-term percutaneous mechanical circulatory support device, with more than 60,000 implants performed annually.^161^ The IABP catheter is typically inserted via the femoral artery or, less commonly, via the axillary or subclavian artery. With a femoral approach, the catheter is advanced retrogradely into the descending aorta until the tip is positioned just distal to the left subclavian artery (LSA). The IABP operates by counterpulsation in the descending thoracic aorta, inflating during diastole and deflating during systole, thereby reducing LV afterload during systole and improving coronary perfusion during diastole. TEE is superior to fluoroscopy for this purpose, as it allows real-time visualization, is portable, free of ionizing radiation, and enables assessment not only of indications but also of complications related to IABP use. TEE also allows exclusion of primary contraindications, such as aortic regurgitation (AR) and aortic dissection, before device insertion.

Echocardiography can also be used to monitor response to IABP support. Ntalianis et al. demonstrated, using transthoracic echocardiography (TTE), that patients treated with IABP showed right ventricular reverse remodeling and improved ventricular function.^162^ When used in combination with VA-ECMO for CS, the IABP modestly reduces LV afterload, increases aortic flow pulsatility, helps prevent LV distension, and may reduce hydrostatic pulmonary edema.^163^ Proper IABP positioning is crucial for device efficacy and for preventing complications such as limb ischemia or aortic dissection.

#### 7.5.2. Transesophageal Echocardiography Examination Sequence to Guide Intra-Aortic Balloon Pump Insertion

A comprehensive TEE examination should be performed initially, followed by a focused assessment of the aortic valve, aortic root, ascending aorta, aortic arch, and descending aorta to exclude atheroma, aneurysmal dilation, or dissection. The aortic valve should be evaluated in both long- and short-axis views at the mid-esophageal level, and any degree of AR should be documented. Subsequently, the guidewire should be confirmed within the descending aorta. In the short-axis view of the descending aorta, the balloon tip can be identified. Still in the short-axis view, the distance between the balloon tip and the origin of the LSA can be estimated by placing the examiner's fingers at the level of the patient's teeth and gradually withdrawing the probe until the LSA becomes visible. The distance between the examiner's fingers and the patient's teeth corresponds to the distance between the balloon tip and the LSA origin. The catheter can then be advanced to the appropriate position, ensuring that the IABP tip is visualized approximately 2 cm distal to the origin of the LSA.

#### 7.5.3. Post-Implantation Checklist

- Assess for complications, including aortic injury, atheromatous plaque rupture, thrombosis or thromboembolism, and balloon rupture;
- Evaluate IABP effectiveness by assessing LV dimensions, function, and LVOT-VTI;
- Improvement in coronary flow can be assessed using color or pulsed-wave Doppler, indicated by increased diastolic flow velocity during balloon inflation.

### 7.6. Veno-arterial Extracorporeal Membrane Oxygenation

#### 7.6.1. Introduction

ECMO is a critical life-support intervention used in patients with severe cardiac and respiratory failure. The role of echocardiography, particularly POCUS, in ECMO is multifaceted and includes patient selection, cannulation guidance, hemodynamic monitoring, and complication detection. This overview highlights each of these roles, emphasizing the importance of echocardiography and POCUS in optimizing ECMO therapy and potentially improving patient outcomes.

Echocardiography is useful for identifying or excluding reversible conditions that may be responsible for hemodynamic deterioration, such as cardiac tamponade, previously unrecognized valvular lesions, and LV dysfunction, thereby potentially obviating the need for ECMO. It also provides critical information regarding contraindications; for example, ECMO should not be used in cases of aortic dissection. Significant AR is a relative contraindication to VA-ECMO, as it increases LV afterload and may exacerbate AR. In addition, echocardiography can detect aortic atherosclerosis, assisting intensivists in selecting cannulation sites (central versus peripheral) or techniques (surgical versus percutaneous). It also aids in evaluating right heart morphology to identify structural abnormalities that may hinder venous cannula placement for venovenous ECMO (VV-ECMO) or VA-ECMO. Figure 23 summarizes the main points of attention for VV-ECMO and VA-ECMO during the pre-cannulation phase, throughout ECMO support, and during the weaning process, as well as potential causes of device malfunction.

**Figure 23:** Bedside echocardiography for VV and VA ECMO. Note: TEE should be performed in all patients when time and clinical conditions allow. Alternatively, TTE may provide important information during the pre-ECMO assessment and the weaning process and should be considered as a noninvasive option. ECMO: extracorporeal membrane oxygenation; RA: right atrium; RV: right ventricle; TAPSE: tricuspid annular plane systolic excursion; FAC: fractional area change; IVS: interventricular septum; IAS: interatrial septum; TTE: transthoracic echocardiography; VV: venovenous; VA: venoarterial.

#### 7.6.2. Patient Selection for Extracorporeal Membrane Oxygenation

Initial assessment and patient selection for ECMO are critical determinants of procedural success. Echocardiography provides essential information on cardiac structure and function and plays a key role in determining patient suitability for ECMO.^164^ Prior to ECMO initiation, a comprehensive evaluation of cardiac function is required to assess the severity of HF and the potential benefit of mechanical circulatory support. Echocardiography offers detailed assessment of LV and RV function, valvular integrity, and the presence of structural heart disease.

RV dysfunction may significantly complicate management of ECMO. Echocardiographic evaluation of RV size, systolic function, and filling pressures is therefore essential for planning the ECMO strategy. Echocardiography is also indispensable for identifying significant valvular abnormalities, such as severe mitral regurgitation or aortic stenosis, which may influence the decision to initiate ECMO. Appropriate recognition and management of these conditions are fundamental to optimizing ECMO therapy.

Hemodynamic instability is a common indication for ECMO support. Echocardiography provides real-time hemodynamic information, including cardiac output, filling pressures, and volume status. Cardiac output can be estimated by measuring stroke volume and heart rate, which is crucial for determining the need for ECMO and for monitoring the effectiveness of mechanical support. Assessment of intravascular volume status using echocardiography guides fluid management and is vital for hemodynamic stabilization before and during ECMO support. Parameters such as IVC diameter and respiratory variation are useful indicators of volume status.

#### 7.6.3. Cannulation Guidance

Accurate cannulation is a fundamental step in ECMO initiation, and echocardiography plays a pivotal role in guiding and confirming correct cannula positioning. In peripheral VA-ECMO, cannulas are typically inserted into a vein (usually the femoral vein) and an artery (most commonly the femoral artery) to provide combined cardiac and respiratory support. TEE is the preferred modality, as TTE may not provide sufficient spatial resolution for this purpose. Clear communication between the ECMO operator and the echocardiographer is essential regarding the intended cannula positions. For example, in VV-ECMO, when separate cannulas are used for drainage and reinfusion, the drainage cannula tip should be positioned in the proximal IVC, just before its entry into the RA. The return cannula should ideally be positioned in the mid-RA, away from the interatrial septum and tricuspid valve.

Echocardiographic monitoring during ECMO is particularly important for assessing hemodynamics and volume status.^165^ In cases of severe LV dysfunction associated with significant mitral regurgitation, the LV may become markedly dilated, and the aortic valve may fail to open because of severely reduced native cardiac output. This condition can lead to blood stasis and subsequent thrombosis in the ascending aorta, left-sided chambers, and pulmonary veins. In such situations, LV unloading or percutaneous balloon atrial septostomy may be beneficial. TEE is the optimal modality for guiding septostomy catheter placement and balloon inflation.

Failure of aortic valve opening during peripheral VA-ECMO represents a serious complication. In these cases, anticoagulation intensity should be increased, and inodilators may be used to reduce afterload, optimize native LV output, and facilitate aortic valve opening. Transitioning from ECMO to a ventricular assist device should also be considered.

In VV-ECMO, hemodynamics are simpler and generally more favorable. Blood is drained from and returned to the right heart without significantly altering RV preload or adversely affecting left heart hemodynamics. VV-ECMO increases mixed venous oxygen saturation, leading to reduced pulmonary vascular resistance and decreased RV afterload. Improved systemic oxygenation may also indirectly enhance LV function by increasing coronary oxygen delivery.

VA-ECMO cannulation may be performed at the bedside in emergency situations or in the cardiac catheterization laboratory if the patient's condition allows. In these settings, a comprehensive echocardiographic assessment, including TTE and/or TEE, should be performed, supplemented by fluoroscopy when appropriate.

In cases of extreme urgency where patient transport is not feasible, bedside cannulation should be performed using focused echocardiography guidance by an experienced operator. Focused ultrasound steps for bedside cannulation include:

- Real-time ultrasound-guided vascular access;
- Ultrasound confirmation of guidewire position in the IVC and aorta using the transhepatic mid-axillary window;
- Continuous monitoring to ensure guidewire stability during cannula exchange;
- Verification that the venous guidewire has not migrated into the RV or SVC;
- Confirmation of venous cannula positioning directed toward the SVC;
- Confirmation of arterial cannula positioning within the aorta;
- Exclusion of immediate complications, such as pericardial effusion, aortic dissection, or cannula malposition (Figure 24).

**Figure 24:** Various situations demonstrating the role of TEE in the evaluation of patients on VA-ECMO. (a) Bicaval view showing the venous drainage cannula in the correct position, with the tip at the junction of the SVC and the RA. (b) Modified bicaval view in a patient presenting with difficulty in oxygenation during the weaning process. Color Doppler shows the presence of a patent foramen ovale with a right-to-left shunt explaining the hypoxia. (c) Transgastric view of the IVC with color Doppler showing signs of VV-ECMO recirculation (turbulent flow due to the short distance between the tips of the drainage and return cannulas, causing the drainage cannula to aspirate blood from the return cannula tip). (d) Transgastric view of the IVC in the same patient on VV-ECMO after repositioning the drainage cannula a few centimeters lower. Color Doppler shows linear flow, indicating less recirculation. (e) and (f) Bicaval view at the mid-esophageal level in a patient on VA-ECMO showing the drainage cannula crossing the interatrial septum through the fossa ovalis tunnel, creating a small iatrogenic ASD. ASD: atrial septal defect; IVC: inferior vena cava; RA: right atrium; SVC: superior vena cava; TEE: transesophageal echocardiography; VA-ECMO: venoarterial extracorporeal membrane oxygenation; VV-ECMO: venovenous extracorporeal membrane oxygenation.

It should be emphasized that this is not basic ultrasound, but rather focused echocardiography, which should be performed by an echocardiographer, as it requires a well-defined and precise protocol. It does not replace comprehensive echocardiography, which remains the preferred approach before and after cannulation. When time permits, a combined approach using ultrasound, TTE, and TEE is ideal, and a complete TEE examination during or after cannulation is recommended to confirm venous cannula positioning and detect previously unrecognized cardiac pathology.

#### 7.6.4. Immediate Effects of Peripheral Venoarterial Extracorporeal Membrane Oxygenation Observed by Bedside Echocardiography

Immediate effects of VA-ECMO may include progressive LV dilation. When LV function is severely impaired, the aortic valve may open intermittently or fail to open entirely, with the appearance of spontaneous echocardiographic contrast within the LV. These findings should be promptly communicated to the ECMO consultant, as they may necessitate additional strategies for LV decompression. Although not an immediate emergency, a comprehensive echocardiographic evaluation is required to confirm the diagnosis, assess filling pressures, and determine the optimal unloading strategy.

All patients with acute CS should undergo daily echocardiographic assessment. In cases of acute hemodynamic deterioration or circuit failure, the critical care team may request a focused study to rapidly identify or exclude specific complications, including:

- Pericardial effusion, which may be large without hemodynamic impact or small with significant compromise if cardiac drainage is impaired;
- Cannula displacement or migration, with or without obstruction;
- Cannula-related thrombosis, with or without obstruction;
- Excessive ventricular unloading;
- Valvular or intracavitary thrombosis;
- Worsening valvular regurgitation (severity and/or duration);
- Inadequate LV unloading characterized by spontaneous LV contrast, biphasic retrograde transmitral flow, or retrograde systolic pulmonary venous flow;
- Shortening of the post-ejection period, assessed by TAPSE or MAPSE.

These findings should be identified or excluded using focused echocardiography protocols performed by experienced echocardiographer. As with all critical care echocardiography, findings must be interpreted within the clinical context and discussed with the treating physician. If TTE images are inadequate and no contraindications exist, TEE should be performed. The use of left-sided contrast agents to enhance image interpretation in patients receiving mechanical circulatory support should be limited to experienced practitioners, as detection of air bubbles may cause some ECMO circuits (e.g., Maquet^®^ or Cardiohelp^®^) to immediately stop. Following any corrective intervention, such as adjustment of ECMO flow or cannula position, real-time reassessment of cardiac function and circulation is mandatory.

In patients receiving univentricular support, the impact on the contralateral ventricle and any interventricular septal shift should also be evaluated. This type of evaluation is highly specialized and requires advanced knowledge of cardiac physiology, mechanical circulatory support, and techniques such as global longitudinal strain, which are beyond the scope of basic POCUS.

#### 7.6.5. Assessment of Unloading and Weaning

Weaning from VA-ECMO and left-sided percutaneous support involves complex protocols that are typically combined with invasive hemodynamic monitoring and tailored to the patient's clinical status.^166^ These processes exceed the scope of focused ultrasound protocols and require the full use of advanced echocardiographic techniques. However, as understanding of acute mechanical circulatory support weaning improves, focused imaging protocols may become more standardized and play an expanded role in the future.

ECMO weaning requires careful assessment of both cardiac and respiratory function to ensure that adequate hemodynamics can be maintained without mechanical support. Echocardiography is central to this evaluation, as it assesses recovery of cardiac function and provides essential information for determining the optimal timing of weaning. Improvement in global LV function and LV ejection fraction indicates recovery and readiness for VA-ECMO weaning. Serial echocardiographic evaluations are critical for monitoring this progression. Recovery of RV function is equally important. Echocardiography assesses RV size, systolic performance, and filling pressures, guiding the weaning process. In patients supported with VV-ECMO, echocardiography may assist in evaluating pulmonary function and readiness for decannulation.^167^ LUS, as an extension of echocardiography, aids in assessing pulmonary congestion and guiding fluid management. The presence of B-lines indicates interstitial edema and may influence fluid management decisions. Improvement in LUS findings supports the decision to wean from both VA-ECMO and VV-ECMO.

#### 7.6.6. Monitoring During Weaning Trials

Echocardiography provides real-time monitoring during weaning trials, ensuring that the patient maintains adequate hemodynamics without ECMO support. It also plays a critical role in the early detection and management of ECMO-related complications, including:

- Thrombosis: Thrombosis is a major risk in patients receiving ECMO because of exposure to foreign surfaces and altered hemodynamics. Echocardiography can detect intracardiac or intravascular thrombi, enabling timely intervention. Identification of thrombi within the cardiac chambers is essential to prevent embolic events. Early detection may prompt initiation or escalation of anticoagulation and other targeted interventions. Thrombi may also form at cannulation sites, posing a risk of embolization or cannula obstruction; echocardiography can identify these thrombi and guide management;
- Bleeding and hematoma: Bleeding is a common complication in ECMO patients, often related to anticoagulation therapy. Echocardiography can identify pericardial effusions and hematomas that require prompt management. Pericardial effusions may develop due to bleeding or inflammation, and rapid detection with timely drainage of clinically significant effusions is critical for patient stability. Focused ultrasound can also detect hematomas surrounding cannulation sites, supporting appropriate management and prevention of further complications;
- Ventricular distension: In patients supported with VA-ECMO, ventricular distension may occur due to inadequate LV unloading. Echocardiography can assess ventricular size and function and guide interventions to relieve distension. Serial assessment can quantify the degree of LV distension and inform therapeutic strategies, including IABP support, Impella^®^ placement, or surgical LV venting. RV distension may be managed by adjusting ECMO settings or adding additional mechanical support. Continuous echocardiographic monitoring is essential for early detection and management of this complication.

### 7.7. Challenges and Future Directions

Although POCUS offers numerous advantages in the management of patients requiring mechanical circulatory support, several challenges must be addressed. Diagnostic accuracy and clinical effectiveness are highly dependent on operator skill and experience. Inadequate training or limited expertise may result in image misinterpretation and inappropriate clinical decisions. Standardized training programs and certification for clinicians using POCUS in this setting are therefore essential to improve diagnostic accuracy and patient outcomes in critical care environments.

While POCUS is a powerful tool, it should be integrated with other imaging modalities, such as echocardiography, CT and magnetic resonance imaging, to provide a comprehensive patient assessment. Combining POCUS with advanced imaging techniques can enhance diagnostic precision and improve treatment planning in complex cases. Future developments in POCUS are expected to include technological advances that improve image quality, portability, and ease of use. High-resolution handheld ultrasound devices and AI–based image analysis hold promise for increasing the accuracy and efficiency of POCUS in mechanical circulatory support.

### 7.8. Conclusion

POCUS has emerged as a critical tool in the management of patients requiring mechanical circulatory support. Its ability to provide real-time, noninvasive assessment of cardiac function, device positioning, and potential complications makes it invaluable in critical care settings. Despite challenges related to operator dependency and the need for integration with other imaging modalities, ongoing advances in technology and training are likely to address these limitations. As POCUS continues to evolve, its role in improving patient care and outcomes in mechanical circulatory support will undoubtedly expand, firmly establishing its place in modern critical care medicine.

## 8. POCUS in the Emergency Department

The use of POCUS is well established as an essential tool for rapid clinical decision-making during the initial assessment of critically ill patients in the emergency department. Despite its well-recognized benefits, POCUS should not be interpreted in isolation nor considered a substitute for careful history taking and a comprehensive physical examination. Rather, it provides valuable complementary information, particularly in the management of critically ill patients and those with potentially time-sensitive diagnoses.

Below, we outline the main applications of POCUS across different clinical scenarios encountered in the emergency department.

### 8.1. POCUS in Chest Pain

Chest pain is a common and often challenging presentation in the emergency setting. POCUS serves as a valuable adjunct for the differential diagnosis of life-threatening conditions, allowing rapid bedside evaluation. Several high-risk diagnoses associated with chest pain can be identified using POCUS, including pulmonary embolism, pneumothorax, and pericardial diseases.

Evaluation of the aorta for dissection and assessment of regional LV wall motion abnormalities for the diagnosis of acute coronary syndromes require advanced training and substantial expertise in echocardiography. Because of the complexity and clinical relevance of these diagnoses, such assessments should preferably be performed by physicians specialized in cardiovascular imaging to ensure diagnostic accuracy and patient safety.^168^

When appropriately applied by adequately trained professionals, POCUS represents a powerful tool to support clinical decision-making.

Below, we summarize the role of POCUS in the main diagnostic considerations.

#### 8.1.1. Evaluation of Pericardial Diseases in the Emergency Department

Pericardial assessment is an integral component of the systematic cardiac evaluation performed with POCUS and is particularly useful in patients presenting with hemodynamic instability, dyspnea, and/or chest pain.^169,170^

POCUS has high diagnostic value for the rapid bedside identification of pericardial effusion and signs of impaired ventricular filling (Figure 8), and it is especially useful for guiding pericardiocentesis (Figures 25 and 26), a procedure detailed in a dedicated section.^169,170^ The detection of pericardial effusion demonstrates high diagnostic accuracy (exceeding 95%), even when performed by emergency physicians without formal cardiology training, provided adequate training has been completed.^169^

Quantification of pericardial effusion (Table 3) can be performed using standard echocardiographic windows, including parasternal long-axis, parasternal short-axis, A4C, and subcostal views, allowing classification of effusion size.^49^

Beyond effusion volume, the rate of fluid accumulation and its anatomical distribution may exceed the pericardium's elastic capacity, resulting in increased intrapericardial pressure and impaired atrial and ventricular filling.^171^ In such cases, specific echocardiographic findings detectable by POCUS (Table 6) support the diagnosis of cardiac tamponade when correlated with a compatible clinical presentation.^49,169^

**Table 6:** Echocardiographic findings of impaired cardiac chamber filling and their clinical significance^49^

Finding	Clinical significance
**Late diastolic** **right atrial collapse**	Occurs during atrial relaxation, when right atrial volume is minimal and pericardial pressure peaks, causing inward invagination of the atrial wall. Diagnostic value increases when collapse persists for more than one third of the cardiac cycle. This is one of the earliest signs of cardiac tamponade. Despite high sensitivity, specificity is approximately 82%, as brief right atrial collapse may occur in other conditions such as increased intrathoracic pressure. Sustained right atrial collapse remains a strong indicator of tamponade.
**Right ventricular** **diastolic collapse**	Typically occurs during early diastole, when ventricular volume is still low. This finding has high specificity when present; however, in certain clinical scenarios – such as right ventricular hypertrophy or markedly elevated right ventricular diastolic pressure (e.g., acute or chronic cor pulmonale) – right-sided chamber collapse may be absent even in tamponade. Although sensitivity exceeds 90%, this sign is less sensitive than right atrial collapse.
**Dilated inferior vena cava with minimal or absent respiratory variation**	In cardiac tamponade, external compression of the right atrium during early diastole limits preload accommodation, resulting in a dilated inferior vena cava with reduced inspiratory collapse (diameter > 2.1 cm with < 50% inspiratory reduction). This finding has a high negative predictive value, whereas its positive predictive value is limited, as it may also be observed in chronic lung disease, heart failure, tricuspid regurgitation, and other cardiac conditions. Visualization is usually obtained in the sagittal plane below the xiphoid process, with diameter measured approximately 2-3 cm proximal to the junction with the right atrium, at the level of the hepatic vein confluence. M-mode may be used for more precise measurements.
**"Swinging heart"**	Oscillatory motion of the heart, typically observed in large pericardial effusions and often associated with beat-to-beat QRS amplitude variation (electrical alternans).

#### 8.1.2. Pleural and Pulmonary Evaluation in the Emergency Department

Among the causes of chest pain identifiable with POCUS are pulmonary conditions, such as pneumonia and pulmonary embolism, and pleural conditions, including pneumothorax and pleural effusion. Lung Ultrasound (LUS) enables direct visualization of the pleural line and assessment of the lung sliding sign, which is absent in pneumothorax. The absence of B-lines and the presence of the lung point further support the diagnosis.^49,172^ Rapid exclusion or confirmation of pneumothorax is critical for immediate clinical management.

Ultrasound findings suggestive of pulmonary embolism are discussed in the following section.

### 8.2. Evaluation of the Patient with Dyspnea

LUS is one of the primary applications of POCUS for rapid, real-time bedside assessment of patients presenting with dyspnea in the emergency department. It should always be interpreted in conjunction with clinical history and a thorough physical examination since it can significantly expedite diagnostic clarification and appropriate treatment initiation.

#### 8.2.1 Pulmonary Congestion, Pleural Diseases, and Other Diagnoses Assessed by Lung Ultrasound

LUS is an excellent tool for rapid, noninvasive assessment of pulmonary congestion. It is based on the identification of ultrasound artifacts known as B-lines, which strongly indicate fluid accumulation within the pulmonary interlobular septa.

B-lines, also referred to as "comet-tail artifacts," are vertical, hyperechoic, well-defined reverberation artifacts that arise from the pleural line, extend to the bottom of the screen, and move synchronously with lung sliding (Figure 13b). They occur in the presence of interlobular septal thickening or fluid accumulation, as seen in pulmonary congestion. The presence of three or more B-lines in a single intercostal space suggests clinically significant pulmonary congestion, and both their number and distribution correlate with congestion severity.^63^

Section 3 provides a detailed description of the essential aspects of LUS application in the emergency department, including:

- Technical considerations of LUS;
- Most common findings and their clinical significance;
- The role of POCUS in the diagnosis of pulmonary congestion, pleural effusion, interstitial syndromes, pneumonic consolidation, atelectasis, pneumothorax, and pulmonary embolism.

Because pulmonary embolism involves multiple ultrasound findings beyond LUS alone, this diagnosis is discussed in detail in the following section.

#### 8.2.2. Pulmonary Embolism

As with other potentially life-threatening causes of chest pain, early diagnosis of pulmonary embolism is essential. However, pulmonary embolism remains a challenging diagnosis because it may mimic several other clinical conditions. In this context, POCUS is a highly valuable bedside diagnostic tool, enabling rapid, integrated assessment of cardiac, pulmonary, and vascular findings (Table 7).

**Table 7:** POCUS findings supporting the diagnosis of pulmonary embolism^131^

Finding	Description	Comments
**DVT (Figure 19)**	Visualization of a non-compressible thrombus in deep veins (femoral, popliteal, etc.)	Highly suggestive of pulmonary embolism when associated with respiratory symptoms
**Right ventricular dilation (Figures 6a and 6b)**	RV diameter greater than LV diameter (RV/LV > 1) on paras-ternal or apical views	Suggests RV pressure overload (massive or submassive pulmonary embolism)
**McConnell sign**	Hypokinesia of the basal and mid free wall of the RV with preserved apical con-tractility	Highly suggestive of pulmonary embolism (specificity ≈ 94%)
**Flattened interventricular septum (Figure 7)**	Septal shift toward the LV during diastole	Indicates RV pressure overload
**Pulmonary hypertension**	Estimated pulmonary artery systolic pressure	Can be calculated from peak tricuspid regurgitation velocity
**Thrombus in transit**	Direct visualization of thrombus in right-sided cardiac chambers	Highly specific, but low sensitivity; differential diagnosis includes tumors and vegetations
**Thrombus in** **pulmonary artery**	Direct visualization of thrombus in the pulmonary artery trunk or branches (rare)	Highly specific, low sensitivity; more common in central pulmonary embolism
**Pleural effusion**	Fluid in the pleural space (usually small to moderate)	Nonspecific, but frequently observed in pulmonary embolism
**Triangular subpleural con-solidations (Figure 13k)**	Suggest pulmonary infarction	Patients with pulmonary em-bolism without pulmonary infarction may have normal LUS findings

DVT: deep vein thrombosis; LUS: lung ultrasound; LV: left ventricle; RV: right ventricle.

The combined evaluation of these three key anatomical sites – the heart, lungs, and deep venous system of the lower limbs – achieves a specificity greater than 95%. In hemodynamically unstable patients, bedside cardiac POCUS should be the first diagnostic modality employed. The absence of signs of acute right ventricular pressure overload or right ventricular failure may help exclude massive pulmonary embolism as the underlying cause. Additionally, cardiac POCUS provides valuable information regarding alternative etiologies, such as LV failure, pericardial disease, and intravascular volume status.

#### 8.3. Evaluation of the Patient with Hemodynamic Instability or Organ Dysfunction

Ensuring adequate perfusion pressure is essential in patients presenting with hemodynamic instability and/or organ dysfunction. Assessment of FR (Figure 21) provides critical information for determining the etiology of shock and guiding the most appropriate hemodynamic support strategy.

Conversely, patients with RV failure, pulmonary hypertension, or volume overload are prone to developing significant systemic venous congestion. This process leads to a reduction in the arteriovenous pressure gradient, elevation of capillary hydrostatic pressure, and development of interstitial edema, which may result in organ dysfunction, particularly AKI, and subsequent fluid retention, creating a vicious cycle.^90–93^ Section 6 provides a comprehensive description of the role of POCUS in shock states.

Because AKI is often one of the earliest manifestations of organ dysfunction in both hypovolemic and hypervolemic patients, POCUS is particularly useful for evaluating patients with AKI (Figure 17), given the well-known limitations of clinical volume assessment.^94–96^ Bedside use of POCUS therefore represents a valuable tool for assessing volume status and quantifying systemic venous congestion in the emergency department.

In Section 4, the essential information regarding the application of VExUS in the emergency department is described, including:

- Scientific evidence (Table 5);
- Technical aspects of VExUS for assessment of the IVC and, when necessary, analysis of additional components (hepatic veins, portal vein, and intrarenal interlobar veins) (Figure 15).

Notably, VExUS does not determine the need for fluid administration. Instead, it serves as a key parameter for assessing tolerance to fluid therapy, helping identify patients in whom interruption of fluid administration or active fluid removal is likely to be beneficial.

### 8.4. Evaluation of the Patient with Peripheral Edema

In addition to LUS for the assessment of pulmonary congestion and VExUS for evaluation of volume status and quantification of systemic venous congestion, POCUS is highly useful in the investigation of specific causes of peripheral edema, such as DVT.^131^

Section 5 provides a detailed description of the essential information regarding the application of POCUS in the evaluation of DVT, including:

- Scientific evidence;
- Technical aspects (compression protocols) (Figure 16).

## 9. POCUS in Cardiac Arrest

### 9.1. Introduction

POCUS in cardiac arrest (POCUS-CA), when performed by a trained operator, enables assessment of chest compression quality, rapid diagnosis of treatable causes of cardiac arrest with non-shockable rhythms (hypovolemia, tension pneumothorax, pulmonary embolism, and cardiac tamponade), monitoring of interventions, and evaluation of response to treatment (Figure 27).^174^ It also provides relevant prognostic information regarding the likelihood of return of spontaneous circulation and survival outcomes.^175^

**Figure 27:** Usefulness of POCUS-CA. Central illustration of figure represents the suggested team organization based on positioning during POCUS-CA. The subcostal view allows rapid confirmation of cardiac arrest versus cardiac contraction, differentiation between a shockable and a non-shockable rhythm (panel 1), and confirmation of the effectiveness of chest compressions (panel 2B). It is possible to identify reversible causes such as tension pneumothorax (panel 3C), hypovolemic shock (panel 3D), cardiac tamponade (panel 3E), and signs of pulmonary embolism (panel 3F). After return of spontaneous cardiac rhythm, a focused evaluation can be performed to identify other causes (panel 4).

During cardiac arrest, the use of POCUS remains surrounded by uncertainties, unlike other clinical contexts in which its role is more firmly established. The main concern is that POCUS may delay the resumption of chest compressions, which are essential for cardiopulmonary resuscitation (CPR), and that, to date, its use has not demonstrated a significant impact on patient prognosis. These uncertainties are largely due to the lack of a universal protocol that can be definitively incorporated into Advanced Cardiac Life Support (ACLS).^176^

### 9.2. Technical Aspects

Figure 27 illustrates team organization during cardiac arrest management, highlighting the position of the POCUS operator on the patient's left side.

We recommend the exclusive use of a phased-array transducer (3.5–5 MHz), as it allows adequate evaluation of the lungs, heart, and abdomen according to the clinical scenario. Images should be acquired during the 10-second pause for rhythm analysis, with interpretation performed during the subsequent resuscitation cycle. During chest compressions, subcostal views may be obtained to assess the effectiveness of cardiac massage.^177^ Several POCUS-CA protocols have been described; however, we recommend the CASA protocol, as it was the only one shown to reduce interruption time for rhythm checks. This protocol focuses on the assessment of cardiac activity and potential causes such as pulmonary embolism and cardiac tamponade.^178^ During cardiac arrest, the POCUS operator may also actively search for pneumothorax and hypovolemia.^178^

### 9.3. Performance of POCUS in Cardiac Arrest

When a cardiac arrest rhythm is identified as non-shockable, it is advisable to initiate POCUS-CA evaluation using the subcostal window. Images should be reviewed during the beginning of the next resuscitation cycle to avoid interference with CPR. During the interval designated for pulse check and rhythm analysis, additional echocardiographic windows may be examined. These include the lung window for assessment of pleural sliding, the A4C view, the PSAX view, and finally the PLAX view. Depending on the clinical scenario, the FAST protocol for detection of free intraperitoneal fluid may be incorporated into POCUS-CA without interrupting resuscitation maneuvers, providing additional diagnostic information.^179^ POCUS-CA is fundamental for identifying reversible causes of cardiac arrest and should be used in conjunction with systematic evaluation for conditions such as hypovolemia, cardiac tamponade, tension pneumothorax, and pulmonary embolism (Figure 27, panel 3).^174^

### 9.4. Confirmation of Cardiac Arrest, Assessment of Chest Compression Effectiveness, and Prognostic Role of Cardiac Activity in Pulseless Electrical Activity

Although the distinction between shockable and non-shockable rhythms depends on ECG, ultrasound may identify false cardiac arrest by allowing differentiation between true asystole and fine ventricular fibrillation with demonstrable myocardial contraction. This finding is present in approximately 10%-35% of patients initially diagnosed with asystole.^174^

Assessment of chest compression effectiveness is another important function of POCUS-CA, as it provides direct, real-time visualization of cardiac chamber contraction and relaxation during cardiac massage. Prior research emphasizes that improper hand positioning during resuscitation may result in compression of the ascending aorta, aortic root, or LVOT, rather than the LV itself. POCUS-CA allows adjustment of both hand position and compression force.^174^

### 9.5. Diagnosis of Reversible Causes Using POCUS in Cardiac Arrest

#### 9.5.1. Cardiac Tamponade

Cardiac tamponade may result from both traumatic and non-traumatic causes. Even small volumes of rapidly accumulating pericardial fluid can significantly affect cardiovascular hemodynamics by impairing ventricular filling and cardiac output, potentially leading to tamponade physiology.^174^

Differentiation between pericardial and pleural effusion is not always straightforward. In the PLAX view, pericardial effusion appears anterior to the descending aorta and above the posterior pericardial reflection. In contrast, pleural effusion is located posterior to the descending aorta and below this pericardial reflection (Figure 9).^21^

Importantly, simple detection of pericardial effusion is insufficient to diagnose cardiac tamponade. Classic tamponade signs, such as systolic right atrial collapse and diastolic right ventricular collapse, are not visible in patients already in cardiac arrest. Assessment of IVC may assist in this differentiation when it is dilated, indicating elevated right atrial pressure.^174^

Echocardiography also plays a key role in guiding pericardiocentesis in cardiac tamponade. Several techniques have reported success rates close to 95% with complication rates below 2%. The subcostal window is the preferred approach, guiding needle trajectory during the procedure.^180^

#### 9.5.2. Pulmonary Embolism

Pulmonary embolism may be identified by ultrasound through both direct and indirect findings. Direct evidence consists of visualization of the thrombus itself. Indirect signs include acute right ventricular dilation, in which the right ventricle becomes larger than the LV, with a right-to-left ventricular ratio of at least 1:1. In addition, a D-shaped LV in the PSAX view is another indicator of right ventricular dilation (Figure 7).

When pulmonary embolism is suspected, a focused ultrasound protocol for detection of DVT may be incorporated without compromising chest compressions (Figure 17).^174^

#### 9.5.3. Tension Pneumothorax

Tension pneumothorax is a major cause of cardiac arrest and may result from pre-existing lung disease, complications of positive-pressure ventilation, or venous access procedures.^181^

Signs of pneumothorax can be identified using LUS at the fourth to fifth intercostal spaces along the midclavicular line. These include absence of pleural sliding, presence of the stratosphere or barcode sign (Figure 27, panel 3C), and absence of lung pulse. The most definitive sign is the lung point, which, when identified, has very high specificity for confirming pneumothorax.^174^

#### 9.5.4. Hypovolemia

During cardiac arrest, ultrasound may reveal complete collapse of the ventricles and collapse of IVC. These findings, when interpreted alongside clinical evolution and other indicators, may suggest hypovolemia as an underlying cause of cardiac arrest (Figure 27, panel 3D). In trauma settings, rapid identification of blood within the abdominal cavity or thorax is essential, as hemorrhage may be the primary cause of hypovolemic shock.^21^

## 10. Ultrasound-Guided Procedures

### 10.1. Ultrasound-Guided Vascular Access

Ultrasound-guided cannulation has become a widely adopted practice in most medical centers, largely due to its demonstrated superiority in terms of safety. The effectiveness of the procedure is directly related to the operator's knowledge of regional anatomy, clinical experience, and the quality of the equipment used.^182,183^

The occurrence of complications is multifactorial and depends on factors such as operator expertise, patient body habitus, and clinical status (including level of cooperation, agitation, and volume status) as well as the anatomical complexity of the site to be cannulated. Notably, the number of cannulation attempts is directly associated with a higher risk of adverse events.

Historically, most of these procedures were performed "blind," relying solely on superficial anatomical landmarks. Currently, there is strong evidence that ultrasound-guided catheter placement is safer, making ultrasound an extremely useful tool for guiding cannulation and monitoring interventional procedures.^184^

#### 10.1.1. Cannulation Technique

Seldinger described the percutaneous vessel cannulation technique for catheter insertion. It can be summarized as a "catheter-over-guidewire" approach: the vessel is punctured percutaneously, followed by introduction of a guidewire through the needle. The guidewire then serves as a rail, allowing the catheter to be advanced into the vessel as effectively and atraumatically as possible.

There are two main ultrasound-guided cannulation techniques:

1. **Static technique:** This approach consists of a preliminary ultrasound survey of vascular anatomy and marking the ideal puncture site on the skin. One of its main advantages is that it does not require maintenance of a sterile field during the ultrasound examination, making it useful in less complex situations;
2. **Dynamic or "real-time" technique:** This approach enables continuous, live visualization of the vascular structure (vein or artery), the needle trajectory through the tissues, and entry into the vessel lumen. Whenever possible, we recommend this technique, as it improves procedural accuracy, reduces the number of attempts, and is associated with lower complication rates, particularly in patients with complex anatomy or difficult venous access.

#### 10.1.2. Step-by-Step Cannulation (Use Sterile Supplies)

1. Position the patient supine, preferably in reverse Trendelenburg to increase venous caliber and facilitate cannulation. The Valsalva maneuver may also help;
2. Assess the target vessel and adjacent structures (vascular and nonvascular). It is important to differentiate arteries from veins, which can be readily done based on sonographic characteristics as described in Table 8;
3. Perform wide skin antisepsis with 2% chlorhexidine;
4. Place sterile drapes;
5. Administer local anesthesia with 2% lidocaine (a small wheal using an insulin needle);
6. Cover the transducer with a sterile sheath, placing gel at the bottom of the sheath so that it remains in contact with the transducer tip. Securing the sheath with elastic bands facilitates transducer handling;
7. After turning the patient's face 45° to the contralateral side, visualize the vessel in transverse or longitudinal view and measure the distance from the anterior vein wall to the skin (at the planned needle entry site).

**Table 8:** Ultrasonographic differentiation between arteries and veins

Arteries	Veins
Thicker walls	Thinner walls
Non-compressible	Compressible
Pulsatile	Non-pulsatile
Direction of flow on color Doppler	Direction of flow on color Doppler

#### 10.1.3. Transverse (Short-Axis) Cannulation

In this approach, the vein is identified in a transverse section and centered on the ultrasound screen. Needle insertion should be performed exactly in the midline of the transducer, perpendicular to the skin (Figure 28). This technique is considered technically more accessible because it allows simultaneous visualization of the vein and artery within the same imaging field, facilitating differentiation between vascular structures and helping to avoid inadvertent puncture.

**Figure 28:** Needle positioning and orientation in the transverse cannulation technique. The needle can be visualized only as an echogenic dot when crossing the ultrasound beam. Movement of the surrounding tissues helps identify needle location, and indentation of the venous wall confirms correct positioning.

However, its main limitation is the inability to continuously visualize the needle tip. As the needle traverses the ultrasound beam obliquely, it appears as an intermittent hyperechoic (bright) dot, accompanied by a distal echogenic artifact. This artifact may lead to misinterpretation and be incorrectly identified as the needle tip.

Needle position must therefore be inferred from movement of the surrounding tissues during insertion, which can be facilitated by gentle, controlled in-and-out motions. Because direct visualization of the needle tip is limited, prior knowledge of the depth of the anterior venous wall, measured in B-mode from the skin to the vascular lumen, is essential. This depth serves as a reference to avoid excessive advancement, which increases the risk of complications such as posterior wall puncture, arterial injury, or hematoma.

Confirmation of correct needle positioning within the vessel is achieved by observing indentation of the anterior wall and, subsequently, by blood return through the attached syringe. This step is essential before guidewire insertion.

#### 10.1.4. Longitudinal (Long-Axis) Cannulation

The longitudinal approach provides continuous, real-time visualization of the needle trajectory from tissue entry to penetration of the vessel lumen. This allows the operator more refined procedural control, with greater safety and precision (Figure 29).

**Figure 29:** Needle positioning and orientation in the longitudinal cannulation technique. The needle can be visualized along its entire length when fully aligned within the ultrasound beam, with the needle tip recognized by its beveled appearance.

Unlike the short-axis approach, this technique enables direct visualization of the needle tip throughout its entire course, including the exact moment of vessel entry. This eliminates reliance on blood return to confirm correct positioning, as the needle can be visually monitored during the entire puncture. Consequently, the guidewire can be introduced under direct ultrasound guidance, reducing the risk of complications such as posterior wall injury or improper placement.

Despite these advantages, this technique is more technically demanding, particularly for beginners. Precise alignment of the ultrasound beam with the needle axis is required, which demands manual dexterity and familiarity with ultrasound imaging. Minor misalignment may cause the needle to disappear from the field of view, making the procedure challenging for less experienced operators.

#### 10.1.5. Ultrasound-Guided Cannulation Sites

Several central veins may be accessed with ultrasound guidance, and selection of the optimal site depends on anatomical and clinical factors as well as operator experience. The main approaches are described below.

##### a. Internal jugular vein

The internal jugular vein is often considered the site of choice for ultrasound-guided cannulation, primarily due to its accessibility and favorable visualization with the transducer. The most commonly used puncture site is located between the two heads of the sternocleidomastoid muscle, although any location with minimal soft tissue thickness between the skin and the vessel may be suitable. Ultrasound allows clear visualization of the relationship between the vein and the carotid artery, significantly reducing the risk of inadvertent arterial puncture.

##### b. Femoral vein

The femoral vein is a reliable alternative, particularly in emergency situations. To locate it, the transducer is positioned immediately below the inguinal ligament. In most patients, the vein lies medial to the femoral artery; however, in up to 50% of cases, overlap between the two vessels may occur. If the artery completely overlies the vein, assessment of the contralateral side is recommended to avoid complications.

##### c. Subclavian vein

Ultrasound-guided access to the subclavian vein has proven effective, with higher success rates and a lower incidence of complications such as arterial puncture and hematoma formation. In addition, this approach is associated with lower rates of infection and bacteremia as well as less anatomical variability compared with the internal jugular vein.^185^

However, the course of the subclavian vein beneath the clavicle poses technical challenges. Vessel insonation may be difficult for less experienced operators, particularly because of individual anatomical variations and bony interference. For ultrasound-guided infraclavicular cannulation, the following steps are recommended:

- Begin with transverse imaging of the infraclavicular region;
- Identify the vein and artery within the clavipectoral triangle;
- Center the venous image on the screen;
- Rotate the transducer to obtain a longitudinal (long-axis) view of the vein.

This approach allows:

- Real-time visualization of the needle tip;
- Avoidance of posterior wall puncture;
- In some cases, identification of the parietal pleura, thereby increasing procedural safety.

For these reasons, short-axis (transverse) cannulation should be avoided in this region, particularly because of the risk of pneumothorax.^183^

Although meta-analyses have not demonstrated significant differences in success rates for subclavian vein cannulation using ultrasound compared with landmark-based techniques (failure rates of 9% versus 13%, respectively), Caballero et al. recommend ultrasound use in situations associated with higher risk of complications or technical difficulty.^186^ Even when landmark-based techniques fail, ultrasound-guided cannulation demonstrates a higher likelihood of success.

#### 10.1.6. Other Ultrasound-Guided Vascular Punctures

Ultrasound may also be used to guide puncture of the cephalic or basilic veins in the arm, which are commonly used for insertion of peripherally inserted central catheters.

Arterial punctures are performed for diagnostic and therapeutic catheterization procedures and for invasive mean arterial pressure monitoring. When guided by ultrasound, they follow the same principles described for venous access, with the longitudinal technique being the most commonly used approach.

### 10.2. Ultrasound-Guided Pericardiocentesis

Abnormal accumulation of fluid between the pericardial layers is a common cardiac condition with multiple etiologies. Pericardial puncture may be performed electively for diagnostic investigation of pericardial effusion or emergently for immediate relief of cardiac tamponade. Echocardiography is the method of choice for diagnosis, localization, quantification, and assessment of hemodynamic effects.^187^

Traditionally, pericardiocentesis was performed using a blind subxiphoid approach, with reported morbidity of approximately 20% and mortality of 6%. When guided by ultrasound and performed by experienced operators with adequate training, reported rates of major complications range from 0.3% to 3.9%, and minor complications from 0.4% to 20%.^189^ Below, we present the step-by-step technique for ultrasound-guided pericardiocentesis as recommended in this positioning statement.

#### 10.2.1. Ultrasound-guided puncture technique

- A low-frequency phased-array transducer, commonly used in echocardiography, is typically employed. When greater structural detail is required, particularly in parasternal windows (to evaluate the course of the internal thoracic arteries) or in very lateral windows (due to proximity to the pleura), a high-frequency linear transducer is used;
- Figure 25 details the echocardiographic windows used and their frequency of use in a series reported by the Mayo Clinic;^188^
- The area with the largest fluid collection should be identified (although effusions are often circumferential, fluid is not uniformly distributed around the heart);
- The point where the distance between the skin and the pericardial space is shortest should be identified as well as adjacent structures along the needle trajectory (Figure 26);
- The patient should be positioned supine with the head of the bed elevated between 30° and 60°;
- Local anesthesia with mild sedation using a central venous cannulation kit;
- Puncture: needle insertion at the previously marked site, always along the upper border of the rib (to avoid intercostal vessel injury); in apical windows and in the subcostal window, interposition of hepatic tissue should be excluded;
- After reaching the pericardial space (with drainage of fluid or blood), the catheter is introduced using the Seldinger technique, and correct positioning is confirmed by injection of 5 mL of agitated saline. The catheter is then secured and a sterile dressing applied as usual.

**Figure 25:** Echocardiographic windows used for ultrasound-guided pericardial puncture and their frequency of use in a series reported by the Mayo Clinic.^188^

**Figure 26:** Identification of the site of greatest fluid accumulation (blue line) and the site where the distance between the skin and the pericardial space is shortest (green).

#### 10.2.2. Contraindications

Although there are no absolute contraindications to pericardiocentesis, particular caution is required in specific situations. When pericardial effusion is associated with acute aortic dissection or rupture of the LV free wall, as may occur after AMI or thoracic trauma, pericardial drainage should only be considered in the absence of immediate availability of cardiac surgery. In such cases, puncture should be performed slowly and in a controlled manner, draining only enough fluid to temporarily stabilize hemodynamics until surgical intervention becomes available.

Another relative contraindication is significant thrombocytopenia, particularly when the platelet count is below 50,000/mm^3^, which increases the risk of bleeding.^189^

#### 10.2.3. Complications

Bedside pericardial puncture, although potentially life-saving in cases of cardiac tamponade, is not without risk. Safe execution requires precise anatomical knowledge, appropriate technique, and preferential use of echocardiographic guidance. A range of complications may occur, varying in severity from self-limited vagal reactions to serious cardiac or vascular injuries. Table 9 describes potential complications associated with this procedure.

**Table 9:** Potential complications during pericardial puncture

**Major**	Cardiac chamber injury Laceration of coronary, intercostal, or internal thoracic arteries Visceral puncture Pneumothorax Arrhythmias Pericardial decompression syndrome
**Minor**	Vasovagal hypotension and bradycardia Supraventricular arrhythmias Pleuropericardial fistula

### 10.3. Evaluation of Gastric Fullness

TEE is performed under sedation, and for procedural safety it is essential to assess gastric contents in order to avoid the risk of aspiration and its consequences. In routine practice, patients are often asked only about fasting duration. This subjective assessment may increase the risk of aspiration, particularly in conditions associated with gastroparesis (pregnancy, diabetes, obesity, hemodynamic instability, etc.). For this positioning statement, we adopt the recommendations of the American Society of Anesthesiologists regarding fasting times^190^ (Table 10).

**Table 10:** Recommended fasting times according to characteristics of the last meal^190^

DIET	Fasting time
Clear liquids	2 hours
Breast milk	4 hours
Non-clear liquids and infant formula	6 hours
Light meals and low volume	6 hours
Fatty meals	8 hours or more

Gastric POCUS allows simple, direct assessment of the quantity and quality of gastric contents prior to the procedure, thereby increasing procedural safety.

#### 10.3.1. Assessment Technique

Gastric assessment with POCUS is generally performed with the patient in the supine position or right lateral decubitus. A low-frequency curvilinear transducer (2-5 MHz) is used to obtain images of the epigastric region and the gastric antrum. The probe is positioned longitudinally in the sagittal plane just below the xiphoid process and oriented toward the patient's left side. Identification of the gastric antrum is essential, as it is the most sensitive site for detecting gastric contents (Figure 30a).^191^

**Figure 30:** A) Gastric antrum with anechoic content indicating a full stomach (S) with liquid content. A: aorta; L: liver. B) Empty stomach with collapsed gastric antrum (S). C) Full stomach with solid content characterized by hyperechoic white areas (S).

#### 10.3.2. Sonographic Findings

- **Empty stomach:** an empty stomach typically shows a collapsed gastric antrum with thin walls and no discernible liquid or solid content. The gastric mucosa may appear as a hyperechoic line, and the lumen is virtually content-free (Figure 30b);
- **Liquid-filled stomach:** when the stomach contains liquid, the gastric antrum becomes distended and appears anechoic (black) on ultrasound. This is due to the presence of fluid, which does not reflect sound waves, creating a dark image. Peristaltic movements may be observed in some cases (Figure 30a);
- **Solid-content stomach:** a stomach containing solid or thick material presents a more complex sonographic appearance. The gastric antrum may appear heterogeneous, with hyperechoic (white) areas indicating solid particles mixed with fluid. This pattern is typical after ingestion of solid meals or foods that are difficult to digest (Figure 30c).

Quantification of gastric contents using POCUS emerges as a useful tool that adds procedural safety, particularly in scenarios where clinical history and fasting time cannot be reliably determined. Another potential advantage is anticipation of the procedure when sonographic findings indicate an empty stomach, although this approach has not yet been safely validated.

## 11. Final Considerations of this Positioning Statement

### 11.1. Current Reality and Future Perspectives

The use of POCUS represents a natural extension of the modern physical examination, providing targeted, real-time data that directly impact clinical decision-making. Its structured application allows rapid and effective assessment of critical conditions in emergency settings, ICUs, and even outpatient environments. However, as described in Section 1, safe and effective use requires appropriate legal regulation, standardized training, adequate supervision, and continuous updating of technical and interpretative skills.

Incorporation of POCUS into clinical workflows should be accompanied by training programs, competency certification, and continuous quality-monitoring strategies. In this way, the method can reach its full potential as a complementary diagnostic tool, promoting more precise, agile, and patient-centered care.

### 11.2. Contextualization of Cardiovascular Point-of-Care Ultrasound within Public Health in Brazil

Incorporation of cardiovascular POCUS into the Brazilian Unified Health System (SUS) occurs in the context of expansion of the national telehealth strategy, particularly following consolidation of the Telessaúde Brasil Redes Program^192^ and the SUS Digital Strategic Action,^193^ aimed at reducing access inequalities and supporting care qualification. Despite this progress, significant regional asymmetries in infrastructure and connectivity remain.^192,193^ Adoption of POCUS faces barriers such as limited equipment availability, uneven training, absence of standardized clinical workflows, and logistical challenges related to maintenance, technical support, and integration with information systems, as discussed in national reviews on the topic.^13,194^ Portable ultrasound equipment, including handheld devices, typically costs between US$ 4,000 and US$ 6,000 on the international market, according to global manufacturer catalogs,^195,196^ with additional annual software and service subscription fees. Potential funding opportunities include federal programs linked to SUS Digital,^192,193^ as well as innovation support lines from Financiadora de Estudos e Projetos (FINEP), such as Inova Saúde and Direct Support for Innovation,^197,198^ and state-level funding calls, such as those from Fundação Carlos Chagas Filho de Amparo à Pesquisa do Estado do Rio de Janeiro (FAPERJ),^199^ aimed at equipment acquisition, technological infrastructure, and applied research. A challenging yet promising future scenario thus emerges for large-scale implementation of this methodology, not only in private health care but also within the public health system.

## Data Availability

The data underlying the text of the Position Statement are included in the manuscript.

## List of abbreviations and acronyms

A4C: Apical 4-chamber view
AI: Artificial intelligence
AKI: Acute kidney injury
AMI: Acute myocardial infarction
AR: Aortic regurgitation
ARDS: Acute respiratory distress syndrome
BLUE: Bedside Lung Ultrasound in Emergency
CASA: Cardiac Arrest Sonographic Assessment
COPD: Chronic obstructive pulmonary disease
CPR: Cardiopulmonary resuscitation
CS: Cardiogenic shock
CT: Computed tomography
CTPA: CT pulmonary angiography
CUS: Compression ultrasound
CVP: Central venous pressure
DICOM: Digital Imaging and Communications in Medicine
DVT: Deep vein thrombosis
ECMO: Extracorporeal membrane oxygenation
ECG: Electrocardiography
EFAST: Extended Focused Assessment with Sonography in Trauma
EGLS: Echo Guided Life Support
EIOT: End-inspiratory occlusion test
FAST: Focused Assessment with Sonography for Trauma
FR: Fluid responsiveness
FT: Fluid tolerance
HF: Heart failure
HFrEF: HF with reduced ejection fraction
HFpEF: HF with preserved ejection fraction
HR: Hazard ratio
IABP: Intra-aortic balloon pump
ICU: Intensive care unit
IVC: Inferior vena cava
LAP: Left atrial pressure
LR: Likelihood ratio
LSA: Left subclavian artery
LUS: Lung ultrasound
LV: Left ventricle
LVEF: LV ejection fraction
LVOT: LV outflow tract
MAPSE: Mitral annular plane systolic excursion
NPV: Negative predictive value
OR: Odds ratio
OSAUS: Objective Structured Assessment of Ultrasound Skills
PE: Pulmonary embolism
PEff: Pericardial effusion
PLAPS: Postero-lateral alveolar and/or pleural syndrome
PLAX: Parasternal long-axis view
PLR: Passive leg raising
POCUS: Point-of-care ultrasound
POCUS-CA: POCUS in cardiac arrest
PPV: Positive predictive value
PSAX: Parasternal short-axis view
RA: Right atrium
RAP: RA pressure
ROSC: Return of spontaneous circulation
ST-MCS: Short-term mechanical circulatory support
SUS: Sistema Único de Saúde
SVC: Superior vena cava
TAPSE: Tricuspid annular plane systolic excursion
TEE: Transesophageal echocardiography
TTE: Transthoracic echocardiography
VA: Venoarterial
VExUS: Venous Excess Ultrasound Score
VV: Venovenous
VTI: Velocity-time integral
VUS: Vascular ultrasonography
